# Vanadium-Dependent Haloperoxidase Gene Evolution in Brown Algae: Evidence for Horizontal Gene Transfer

**DOI:** 10.3390/ijms26020716

**Published:** 2025-01-16

**Authors:** Zihao Yuan, Jie Zhang, Delin Duan

**Affiliations:** 1 Key Lab of Breeding Biotechnology and Sustainable Aquaculture, Shandong Province Key Laboratory of Experimental Marine Biology, Institute of Oceanology, Chinese Academy of Sciences, Qingdao 266071, China; 2 Laboratory for Marine Biology and Biotechnology, Qingdao Marine Science and Technology Centre, Qingdao 266237, China

**Keywords:** brown algae Phaeophyceae, vanadium-dependent haloperoxidase, horizontal gene transfer

## Abstract

Compared with green plants, brown algae are characterized by their ability to accumulate iodine, contributing to their ecological adaptability in high-iodide coastal environments. Vanadium-dependent haloperoxidase (V-HPO) is the key enzyme for iodine synthesis. Despite its significance, the evolutionary origin of V-HPO genes remains underexplored. This study investigates the genomic and evolutionary dynamics of V-HPOs in brown algae, focusing on Laminariales species, particularly *Saccharina japonica*. Genomic analyses revealed the extensive expansion of the V-HPO gene family in brown algae, with 88 V-HPOs identified in *S. japonica*, surpassing the number in red algae. Phylogenetic analysis demonstrated distinct evolutionary divergence between brown and red algal V-HPOs, with the brown algal clade closely related to bacterial V-HPOs. These findings suggest horizontal gene transfer (HGT) played a key role in acquiring V-HPO genes, particularly from Acidobacteriota, a bacterial phylum known for genomic plasticity. Additionally, enriched active transposable elements were identified around V-HPO genomic clusters, highlighting their role in tandem gene duplications and rapid HGT processes. Expression profiling further revealed dynamic regulation of V-HPOs in response to environmental conditions. This study provides new insights into how HGT has driven kelp genomic adaptations and enhances understanding of marine ecological success and evolutionary processes.

## 1. Introduction

Laminariales kelp, a large brown algal group, holds a vital ecological role in marine ecosystems and contributes significantly to the global aquaculture industry [1]. As a primary producer in coastal environments, Laminariales kelp forests support diverse marine species by providing food, habitat, and shelter. Beyond its ecological significance, kelp is an abundant source of bioactive compounds, among which iodine stands out because of its remarkable concentration and biological importance [2]. As one of the richest natural sources of iodine, kelp has drawn attention for its potential health benefits, particularly in supporting thyroid function and preventing iodine deficiency disorders [3]. Iodine deficiency remains a global health issue, affecting millions of people particularly in regions with limited access to iodine-rich foods or iodized salt. Iodine deficiency disorders, including goiter, intellectual impairment, and developmental issues, are preventable but persist because of dietary limitations [4]. Kelp’s high iodine content has led to its utilization as a dietary supplement in regions vulnerable to iodine deficiency disorders.

However, understanding kelp’s iodine synthesis pathway is not only crucial for human health but also provides a genomic foundation for exploring the adaptation of lower plants. Unlike green algae and land plants, red and brown algae are distinct from the green plant lineage (Viridiplantae), differing significantly in pigmentation, cell wall composition, and evolutionary lineage [5,6,7]. These divergences indicate an ecological specialization and evolutionary adaptation that enhances the algae’s resilience in their dynamic marine habitats. For instance, in the intertidal and shallow coastal zones where brown algae typically thrive, environmental conditions fluctuate drastically with intense sunlight, dramatic temperature change, and variable salinity. In response to stress, kelp synthesizes iodine as a natural antioxidant, which protects cellular structures from oxidative damage by neutralizing the effects of reactive oxygen species (ROS) [2]. This unique iodine synthesis pathway has evolved as a biological shield against environmental challenges, providing kelp with an adaptive advantage in marine ecosystems.

Additionally, brown algae, especially kelp, utilize iodine as part of their defense mechanisms against pathogens and herbivores. Studies have shown that the iodine that accumulates in the cells of kelp is highly reactive and exhibits antimicrobial properties, making it toxic to pathogens and herbivores that threaten the algae [2,8]. This dual function, protection from oxidative stress and microbial defense, illustrates how the iodine synthesis pathway likely provides kelp with a significant evolutionary advantage in adapting to the challenging marine environment [9]. Additionally, the iodine compounds released from brown algae are also involved in atmospheric chemistry by participating in cloud formation and influencing the marine iodine cycle, which further underscores its ecological significance in marine ecosystems [10,11].

A key enzyme involved in iodine synthesis in kelp is vanadium-dependent haloperoxidase (V-HPO). V-HPOs are primarily found in marine macroalgae such as brown and red algae, e.g., *Laminaria* or *Corallina* species [12,13]. They catalyze the halogenation of organic substrates by activating halides, primarily iodide or bromide, in the presence of hydrogen peroxide. Such activity is pivotal in producing halogenated organic compounds for the algae’s environmental adaptation, as mentioned above [14]. Moreover, V-HPOs have potential applications in green chemistry due to their catalytic stability and specificity [15]. However, the evolutionary origin of V-HPOs in algae remain poorly understood, sparking interest in potential sources, including horizontal gene transfer (HGT). Brown algae are known to host a diverse microbiome on their surfaces and in surrounding waters, with some researchers proposing that some algae genes may have originated in marine bacteria and were transferred to brown algae via HGT [16,17,18]. Such HGT events would have provided the algae with a new metabolic capability and a distinct advantage in adapting to challenging coastal environments.

Although these events in eukaryotes remain rare, HGT events in which bacterial genes were transferred into plant genomes have been reported, often facilitated by transposable elements. For example, some plants acquired *Agrobacterium*-derived genes through HGT, facilitated by transposons, which allowed for the stable integration of bacterial DNA into the plant genome and provided adaptive advantages in various environments [19]. This acquisition exemplifies how HGT has facilitated evolutionary innovations in plants, enabling them to thrive in challenging environments. In brown algae, however, the investigation of HGT has been limited, partly due to the scarcity of genomic resources.

As genomic sequencing of kelp and related algae has greatly advanced [20,21], new opportunities to explore the genetic basis of iodine synthesis and its mechanism of evolution have emerged. In this study, based on the newly available genomic data, we investigated the genomic foundation of iodine synthesis in kelp and examined the evolutionary dynamics that may have led to the acquisition and development of iodine pathways. By elucidating the genomic evolution of iodine synthesis in brown algae, especially kelp, our research provides deeper insights into the complex interactions between marine organisms and their rapidly changing environment, as well as enhances our understanding of marine adaptation throughout the long course of evolution.

## 2. Result

### 2.1. The V-HPO Gene Family Is Extensively Expanded in the Brown Algae Genome

**Figure 1:**
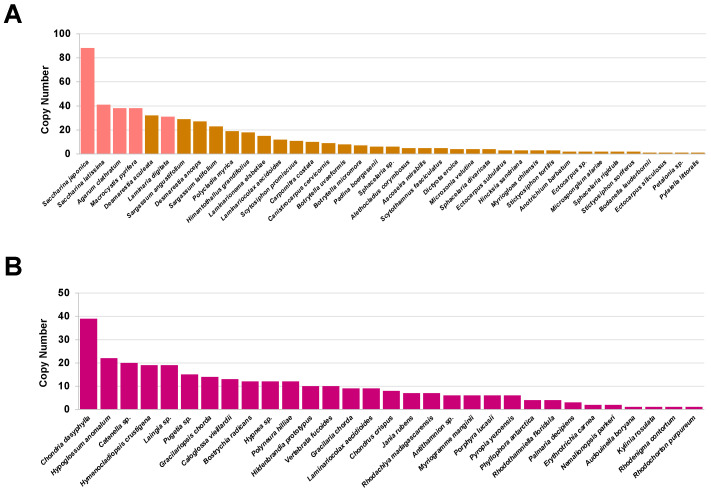
V-HPO copy number landscape in algae. Copy numbers of the V-HPO gene across (**A**) brown algae and (**B**) red algae species. Species are color-coded, with Laminariales species highlighted in red.

Based on genome data mining, we identified a total of 521 and 299 V-HPOs across 39 brown algae and 31 red algae genomes, respectively (Supplementary Tables S1 and S2). In particular, brown algae from the Laminariales order, usually referred to as kelp, contain the highest number of V-HPO genes, with an especially high abundance in the genus *Saccharina*. Specifically, *S. japonica* harbors 88 V-HPO genes, forming large gene clusters, mainly on LG3, LG4, and LG21 (Supplementary Table S3), followed by *S. latissima* with 41, *Agarum clathratum* and *Macrocystis pyrifera* with 38 each, and *Laminaria digitata* with 31 genes. This extensive gene duplication probably underlies the robust iodine synthesis capacity observed in kelp (Figure 1A). In contrast, V-HPO genes are less abundant in red algae, with *Chondria dasyphylla* showing the highest copy number at 39, followed by *Hypoglossuma nomalum* with 22 copies (Figure 1B). The expansion of V-HPO genes in kelp may suggest the genomic mechanisms underlying iodine accumulation in kelp.

### 2.2. The V-HPOs in Brown Algae and Red Algae Exhibit Distinct Differences

**Figure 2:**
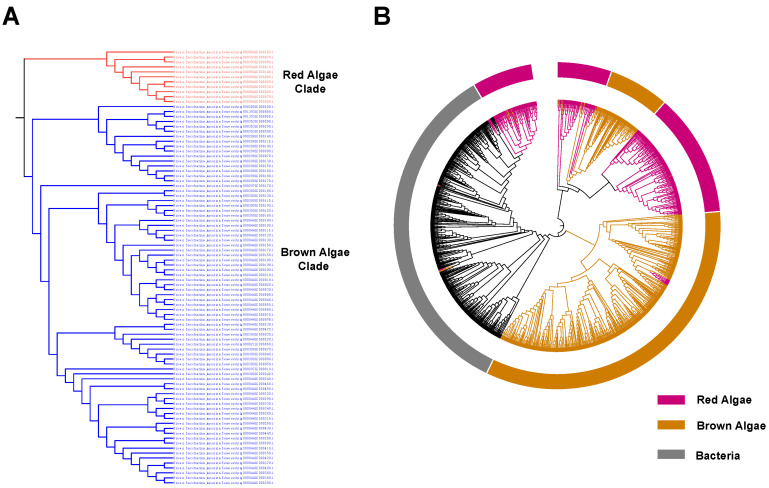
Phylogenetic clade analysis of V-HPOs in algae and their relationship with bacteria. (**A**) The phylogenetic analysis of V-HPOs from *S. japonica*. The red algae and brown algae clades are colored accordingly in red and blue. (**B**) Phylogenetic analysis of algal and bacterial V-HPOs. Different classes are represented by distinct colors for clarity.

Referring to the phylogenetic analysis, the identified V-HPOs in brown algae and red algae can be divided into two main clades, representing the V-HPOs from brown algae and red algae (Figure 2A,B (Supplementary Figures S1 and S2)). These two clades show distinct evolutionary divergences, suggesting a significant evolutionary split. In *S. japonica*, the 88 identified V-HPOs can be further divided into brown algae and red algae clades; one group, consisting of 77 V-HPOs, aligns more closely with the brown algae clade, while the remaining 11 show greater similarity to the red algae clade (Figure 2A). This divergence of V-HPOs within *S. japonica* suggests a bifurcation in halogen synthesis capability. Notably, the V-HPOs in the brown algae clade exhibit a closer phylogenetic relationship with bacterial V-HPOs, potentially indicating an evolutionary origin of brown algae V-HPOs from bacteria (Figure 2B, Supplementary Figure S3).

### 2.3. The Brown Algae Clade V-HPOs in S. japonica Are Probably Horizontally Transferred from Bacteria

**Figure 3:**
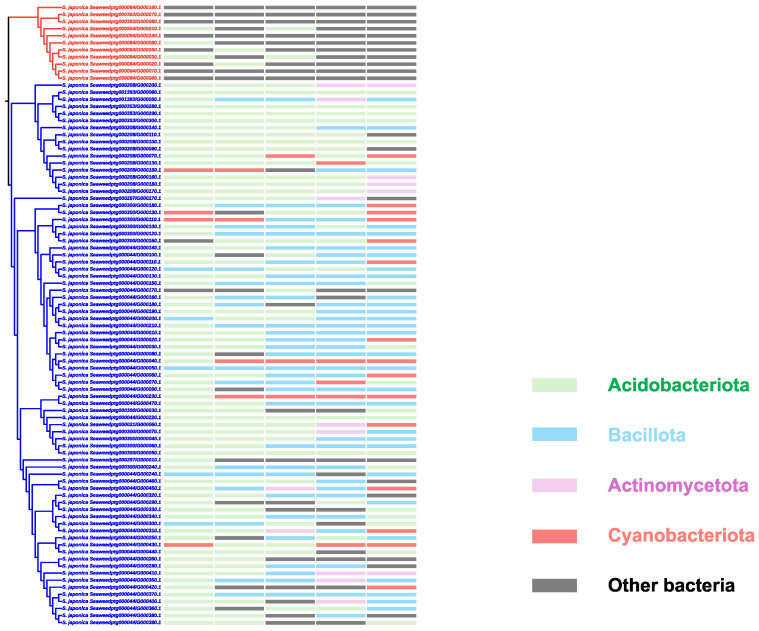
Taxonomic distribution of the top V-HPO hits against the bacterial non-redundant database. The top five taxonomic hits from the sequence search against the non-redundant database are color-coded. Specific bacterial phyla corresponding to these hits are highlighted in detail.

As noted in Section 2.2, the V-HPOs in the brown algae clade exhibit a closer phylogenetic relationship to bacterial V-HPOs. A more detailed search of the RefSeq non-redundant protein database revealed that in *S. japonica* the V-HPOs in the brown algae clade show the highest similarity to bacterial V-HPOs (Supplementary Figure S3). This finding provides further evidence supporting the hypothesis that V-HPOs in the brown algae clade may have been horizontally transferred from bacteria. A comparative BLAST search against bacterial sequences showed that the highest-scoring matches for the brown algae clade V-HPOs are found with the V-HPOs from the *Thermoan aerobaculia* genus bacteria in the Acidobacteria phylum, as well as those from the phyla Bacillota and Cyanobacteriota (Figure 3). Some proteins from the *T. aerobaculia* genus have high similarity with the *S. japonica* genome (Supplementary Table S4), indicating the genomic plasticity of both kelp and *T. aerobaculia* and implying hidden horizontal gene transfers events between *S. japonica* and bacteria.

### 2.4. Tandem Duplication of V-HPOs in the Genome Is Mediated by Active Transposable Elements

**Figure 4:**
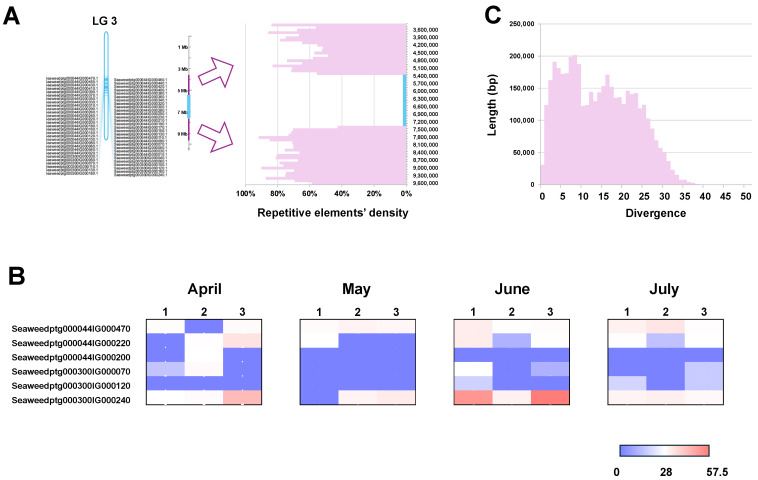
The cluster of V-HPOs on LG3, their transposable element landscapes, and their expression patterns. (**A**) Genomic location of the V-HPO cluster on LG3, along with surrounding transposable elements. The Y-axis represents the total length of repetitive elements within 10,000 bp bins. (**B**) Divergence of repetitive elements: the X-axis represents the divergence of the repetitive elements, and the Y-axis represents its total length. (**C**) The expression pattern of the V-HPOs from April to July; the colors, from blue to red, indicate an increasing expression level.

**Figure 5:**
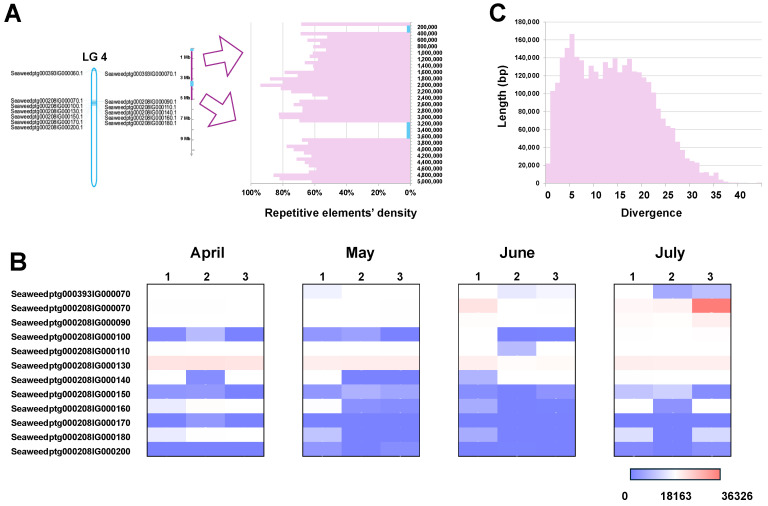
The cluster of V-HPOs on LG4, their transposable element landscapes, and their expression patterns. (**A**) Genomic location of the V-HPO cluster on LG4, along with the surrounding transposable elements. The Y-axis represents the total length of the repetitive elements within 10,000 bp bins. (**B**) Divergence of repetitive elements: the X-axis represents the divergence of the repetitive elements, and the Y-axis represents its total length. (**C**) The expression pattern of the V-HPOs from April to July, the colors, from blue to red, indicate an increasing expression level.

**Figure 6:**
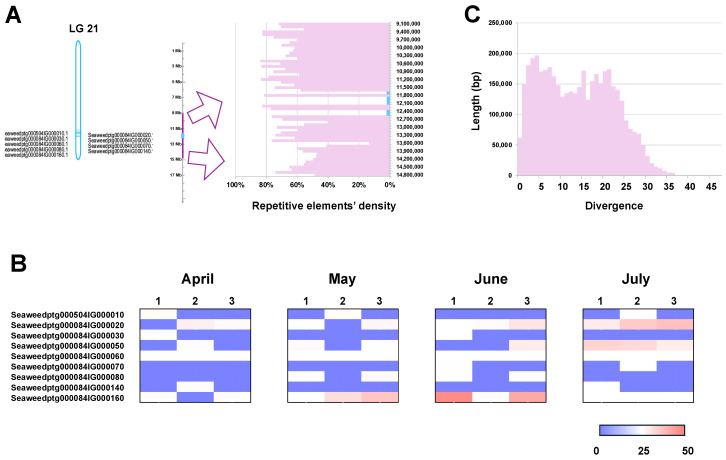
The clusters of V-HPO on LG21, their transposable element landscapes, and their expression patterns. (**A**) Genomic location of the V-HPO cluster on LG21, along with surrounding transposable elements. The Y-axis represents the total length of the repetitive elements within 10,000 bp bins. (**B**) Divergence of repetitive elements: the X-axis represents the divergence of the repetitive elements, the Y-axis represents its total length. (**C**) The expression pattern of the V-HPOs from April to July; the colors, from blue to red, indicate an increasing expression level.

Genomic analysis revealed that the V-HPOs in *S. japonica* are aggregated on chromosomes LG3, LG4, and LG21, where they have undergone tandem duplication 58, 13, and 9 times, respectively, forming three major V-HPO clusters (Figure 4A, Figure 5A, Figure 6A). The V-HPO clusters on LG3 and LG4 predominantly belong to the brown algae clade, while the cluster on LG21 is associated with the red algae clade. Transcriptome analysis further indicates a dose–compensation pattern in V-HPO expression, with a few genes exhibiting the highest expression levels (Figure 4B, Figure 5B, Figure 6B). Furthermore, the expression of V-HPOs also varies with time, peaking in summer (June and July). This unbalanced expression pattern may provide kelp with an adaptive advantage in fluctuating marine iodine environments.

To investigate the evolutionary dynamics underlying this clustering, we analyzed the distribution of repetitive elements, which are mainly composed of transposable elements around the V-HPO clusters. This analysis reveals a high abundance of transposable elements surrounding the V-HPO loci (Figure 4A, Figure 5A, Figure 6A, Supplementary Tables S5–S7); meanwhile, those repetitive elements had low divergence (Figure 4C, Figure 5C, Figure 6C), suggesting that abundant active TE may play roles in the formation and evolutionary diversification of V-HPO clusters. The high density of transposable elements adjacent to the V-HPO loci likely drives the rapid evolution of these genes, potentially contributing to the ecological success of kelp in iodine-rich marine habitats.

## 3. Discussion

As the largest reservoir of natural iodine, the ocean contains approximately 70% of the Earth’s iodine inventory, enabling brown algae, such as kelp, to absorb and store substantial amounts of this iodine [22,23]. Once absorbed, iodide is converted into reactive iodine species, which neutralize ROS. Iodine-derived compounds in algae serve as antioxidants, scavenging excess ROS and protecting cells from oxidative stress [2,8,10]. This adaptation likely evolved as a response to oxidative stressors in intertidal and shallow waters, where kelp, especially those from the Laminariales order, face intense sunlight and fluctuating conditions, such as in oxygen-rich water, both of which elevate ROS levels, risking cellular damage [24]. In addition to forming an algae forest and enriching the local ecology, the iodine released from algae, furthermore, influences the marine environment, impacting local chemical cycles and interacting with microbial communities [25]. This release can also affect atmospheric processes, contributing to aerosol formation and potentially catalyzing ozone destruction in the troposphere [26,27].

In addition to environmental roles, iodine that accumulates in kelp has implications for human health, as it is an essential micronutrient, supporting thyroid function and hormonal balance [9]. The strong antioxidant properties of kelp-derived iodine make it valuable in nutraceuticals and dietary supplements [8,28]. Its potential antimicrobial and anti-inflammatory effects also offer promising insights for future applications in food preservation and pharmaceuticals [28,29]. Moreover, iodine functions as a “beneficial element” that stimulates growth in higher plants, creating potential applications in the fertilizer industry [30].

Notably, brown algae, especially kelp from the genus *Saccharina*, are the only eukaryotes with a robust iodine synthesis capacity, with iodine sometimes accounting for over 4% of their dry weight [2]. Investigating the genomic mechanisms of iodine synthesis in brown algae enhances our understanding of marine plant physiology and provides insights into plant adaptation to environmental changes. From the perspective of evolution, both red and brown algae are not part of the green plant lineage but evolved independently, exhibiting diverse structural and functional adaptations to their environments [5]. Prior research indicates that certain members of the Laminariales order within brown algae exhibit intense genome duplications, with genes often clustered in tandem arrays, which likely occurred early in their evolutionary history. Additionally, the integration of viral genes into brown algal genomes may have significantly influenced their evolutionary trajectory [31]. These findings highlight the remarkable genome plasticity and rapid evolution observed in brown algae, characterized by extensive gene duplications and recombination [21,32]. This adaptability is further supported by evidence that brown algae may have acquired genes via horizontal gene transfer. The HGT process is more commonly studied in bacteria, where it enables bacterial adaptation by introducing novel phenotypes such as antibiotic resistance [33]. Recent studies suggest that some genes in brown algae have bacterial origins, especially those associated with metabolic functions essential for survival in nutrient-poor or variable environments, such as carbohydrate metabolism and stress response. In eukaryotes, HGT is less understood but can have significant impacts on evolution by introducing novel genes or genetic pathways from unrelated species. Recent studies have shown that, in the genome of brown algae, a proportion of their genes appear to have a bacterial origin [21,32]. These genes of bacterial origin in brown algae are often associated with metabolic functions that enhance the ability of algae to survive in nutrient-poor or variable environments, including those involved in carbohydrate metabolism and stress response [34]. Kelp has also acquired genes for polysaccharide degradation, enhancing its ability to utilize complex carbohydrates, a potential advantage in marine environments, where polysaccharide resources are rich [35,36]. Studies also revealed that the kelp genomes had active interactions with the microbiome residing on its surface or surrounding waters [16,17,18]. Thus, the horizontal gene transfer events in brown algae, especially those *Saccharina* species, likely occurred through interactions with its surface bacteria [37]. As a species that undergoes rapid evolution, the transfer of functional genes from bacteria to kelp highlights the evolutionary flexibility of kelp and underscores the importance of HGT in eukaryotic adaptation [38].

Decades of biochemical research reveals that vanadium-dependent haloperoxidases (V-HPOs) are key enzymes for iodine accumulation in kelp, catalyzing iodide oxidation in the presence of hydrogen peroxide to produce hypoiodous acid and other iodinated compounds, which are essential for several physiological and ecological functions in kelp. In this study, using a data-driven approach, we characterized the expansion of V-HPOs in the *S. japonica* genome, which appears to have occurred through tandem duplications on the chromosome. Previous research suggests that V-HPO activation, as revealed by transcriptomic analysis, is life-stage dependent and serves as an adaptive response to changing environmental stressors [21]. Consistent with these findings, our study observed that V-HPO expression increased with period of growth, reaching its peak during summer. This seasonal elevation is likely a response to rising temperatures and heightened pathogenic pressures in the environment. Meanwhile, further phylogenetic analysis suggests that the V-HPO gene in kelp likely originated from bacteria, particularly from the Acidobacteriota phylum, a group known for its metabolic versatility and role in nutrient cycling, inhabiting a range of environments, from acidic soils to marine sediments [39,40]. Studies indicate that Acidobacteriota engage in HGT, sharing genomics traits with Proteobacteria or Cyanobacteria and acquiring genes that enhance resilience across various environments [41,42]. This genetic exchange likely facilitates the incorporation of functions crucial for environmental survival, such as genes involved in stress responses and specialized metabolic pathways, underscoring HGT as a fundamental driver of evolution and ecological success in Acidobacteriota and other bacteria [43,44]. In our study here, in addition to the incorporation of V-HPOs into the kelp genome, we also observed that a significant amount of Acidonbacteria genomic elements share high similarity with the *S. japonica* genome, adding further evidence that there is likely active bacteria–algae genomic interactions

Transposable elements (TEs), a category of repetitive genetic elements, likely facilitated HGT from bacteria to brown algae by mobilizing foreign DNA into host genomes. TEs can integrate bacterial genes into eukaryotic genomes through transposition, enabling novel gene acquisition that may confer adaptive advantages under environmental pressures. In plants, for instance, TEs have mediated the transfer of nitrogen fixation genes, aiding in adaptation to diverse ecological niches [45,46]. This process highlights the evolutionary significance of TEs in enabling eukaryotes to acquire advantageous genes from bacteria, supporting its environmental adaptability to new metabolic pathways [47,48]. Our findings indicate a high density of TEs around the V-HPO loci in *S. japonica*, with relatively low sequence divergence, suggesting recent or active transposition events. This supports the hypothesis that V-HPO genes were transferred horizontally from bacteria to brown algae, mediated by transposable elements, enabling kelp’s iodine synthesis capability. Additionally, TEs are known to drive tandem gene duplications [49], which may explain the observed V-HPO tandem duplications forming genomic clusters on the kelp genome.

Our study provides new insights into kelp genome evolution and the genesis of novel genes through HGT. While research on HGT in kelp is still emerging, our findings suggest frequent genomic exchanges between kelp and bacteria. In particular, the acquisition of V-HPO genes has likely enhanced the defensive and metabolic systems of kelp, giving it a distinct advantage in high-ROS coastal environments. Given the increasing evidence of HGT in both bacteria and eukaryotes, further studies could illuminate the importance of HGT as an evolutionary strategies of kelp and the prevalence HGT events in the marine environment [50].

## 4. Materials and Methods

### 4.1. Sequence Collection and Phylogenetic Analysis

A total of 38 brown algae and 31 red algae genomes were obtained from publicly accessible genome repositories (https://zenodo.org/records/7758534, accessed 11 April 2024) [20], while the *S. japonica* genome was retrieved from Bioproject MEHQ00000000. Protein sequences were analyzed using the conserved domain database (https://www.ncbi.nlm.nih.gov/cdd/, accessed 11 April 2024) [51], and only those with intact PAP2_haloperoxidase domains were selected as V-HPOs. Multiple sequence alignments were performed with Clustal Omega (--full --force) [52]. Phylogenetic analyses of *S. japonica* V-HPOs were conducted using the maximum likelihood method with IQ-TREE 2 v2.1.2 [53], with a bootstrap of 1000 replications. The WAG+F+G4 substitution models were selected based on the Bayesian information criterion (BIC) score via ModelFinder (http://www.iqtree.org/ModelFinder/, accessed 1 October 2024) [54]. Phylogenetic trees were visualized with iTOL (https://itol.embl.de/, accessed 6 October 2024) [55].

### 4.2. Comparative Analyses of Algae and Bacteria V-HPOs

A total of 820 V-HPO sequences from brown and red algae were used as query sequences. These V-HPOs were searched against the NCBI Refseq database via BLASTP, using an E-value threshold of 1 × 10^−5^ to enhance, in part, the accuracy. The sequences were manually curated using the conserved domains database (https://www.ncbi.nlm.nih.gov/cdd/). Phylogenetic analysis of V-HPO proteins from both algae and bacterial sources was performed using the maximum likelihood method in IQ-TREE 2 v2.1.2 [53], with a bootstrap of 1000 replications. The VT+R10 substitution models were chosen based on the Bayesian information criterion (BIC) score using ModelFinder [54]. The final phylogenetic trees were visualized using iTOL (https://itol.embl.de/) [55].

### 4.3. Repetitive Element Analysis

Repetitive elements in the *S. japonica* genome were identified using RepeatModeler v1.0.8, which incorporates RECON [56] and RepeatScout with default parameters (-engine ncbi, -pa 1, -LTRStruct no) [57]. The derived repetitive elements were further annotated by comparison with Repbase [58]. The distribution of repetitive elements surrounding V-HPO loci on each chromosome was assessed and quantified in 10,000 bp bins.

### 4.4. Characterization of the Gene Expression

*S. japonica*, cultured from April to July under controlled conditions at Gaolv Aquaculture Co. (Weihai, China), was subjected to sequencing using Oxford Nanopore Technologies Long-Read sequencing, with three biological replicates. Raw sequencing reads were initially filtered to retain those with a minimum average quality score of 6 and a minimum read length of 350 bp. The filtered reads were then mapped to the reference genome using Minimap2-2.24 (r1122) [59], and only reads with a mapping quality score above 5 were retained for further analysis. Gene expression levels were quantified as counts per million (CPM). Additionally, the location of the V-HPOs on the *S. japonica* linkage group was visualized using MG2C (v2.1) [60].

## 5. Conclusions

Horizontal gene transfer has been recognized as a crucial force in bacterial evolution, and there is growing evidence that it plays a significant role in the evolution of eukaryotes such as plants or brown algae. By acquiring genes from other organisms in the environment, kelp may rapidly boost its adaptability and resilience, contributing to its ecological success in marine ecosystems. In our study, based on the genome sequence availability, we discovered that the V-HPOs in kelp were horizontally transferred from bacteria, which provide kelp with a greater advantage in adapting to challenging environments. As the number of sequenced algae genomes increases, we anticipate that additional instances of HGT will be uncovered, and we will further explore the impact on kelp and other marine organisms. The incorporation of V-HPOs into the kelp genome exemplifies how HGT has facilitated evolutionary innovations in marine algae, enabling them to thrive in challenging marine environments, particularly in the context of global climate change.

## Data Availability

The data presented in this study can be accessed in the article/Supplemental Materials.

## Supplementary Materials

The supporting information can be downloaded at: https://www.mdpi.com/article/10.3390/ijms26020716/s1.
