# Supplementary material for: Vanadium-Dependent Haloperoxidase Gene Evolution in Brown Algae: Evidence for Horizontal Gene Transfer
Source: Int J Mol Sci. 2025 Jan 16;26(2):716. doi: 10.3390/ijms26020716 (PMC11765636; doi:10.3390/ijms26020716)
Supplement: Supplementary file 1 [file ijms-26-00716-s001.zip › Supplemental Table S1. Accessions of V-HPOs identified from brown algae species.pdf]

Supplemental Table S1. Accessions of V-HPOs identified from brown algae species.

Species	**Accessions**
Agarum clathratum	Seq11480-renamed-S20_DDSW200007839-1a_HJLNVDSXY_L4_1_contigs_40w_500min.10
Agarum clathratum	Seq11480-renamed-S20_DDSW200007839-1a_HJLNVDSXY_L4_1_contigs_40w_500min.11
Agarum clathratum	Seq115217-renamed-S20_DDSW200007839-1a_HJLNVDSXY_L4_1_contigs_40w_500min.1
Agarum clathratum	Seq121812-renamed-S20_DDSW200007839-1a_HJLNVDSXY_L4_1_contigs_40w_500min.3
Agarum clathratum	Seq12473-renamed-S20_DDSW200007839-1a_HJLNVDSXY_L4_1_contigs_40w_500min.2
Agarum clathratum	Seq12474-renamed-S20_DDSW200007839-1a_HJLNVDSXY_L4_1_contigs_40w_500min.7
Agarum clathratum	Seq14456-renamed-S20_DDSW200007839-1a_HJLNVDSXY_L4_1_contigs_40w_500min.15
Agarum clathratum	Seq18461-renamed-S20_DDSW200007839-1a_HJLNVDSXY_L4_1_contigs_40w_500min.1
Agarum clathratum	Seq2043-renamed-S20_DDSW200007839-1a_HJLNVDSXY_L4_1_contigs_40w_500min.4
Agarum clathratum	Seq2043-renamed-S20_DDSW200007839-1a_HJLNVDSXY_L4_1_contigs_40w_500min.7
Agarum clathratum	Seq23059-renamed-S20_DDSW200007839-1a_HJLNVDSXY_L4_1_contigs_40w_500min.1
Agarum clathratum	Seq25044-renamed-S20_DDSW200007839-1a_HJLNVDSXY_L4_1_contigs_40w_500min.3
Agarum clathratum	Seq25045-renamed-S20_DDSW200007839-1a_HJLNVDSXY_L4_1_contigs_40w_500min.10
Agarum clathratum	Seq25410-renamed-S20_DDSW200007839-1a_HJLNVDSXY_L4_1_contigs_40w_500min.3
Agarum clathratum	Seq25460-renamed-S20_DDSW200007839-1a_HJLNVDSXY_L4_1_contigs_40w_500min.1
Agarum clathratum	Seq28148-renamed-S20_DDSW200007839-1a_HJLNVDSXY_L4_1_contigs_40w_500min.3
Agarum clathratum	Seq28496-renamed-S20_DDSW200007839-1a_HJLNVDSXY_L4_1_contigs_40w_500min.2
Agarum clathratum	Seq30229-renamed-S20_DDSW200007839-1a_HJLNVDSXY_L4_1_contigs_40w_500min.7
Agarum clathratum	Seq31058-renamed-S20_DDSW200007839-1a_HJLNVDSXY_L4_1_contigs_40w_500min.5
Agarum clathratum	Seq32263-renamed-S20_DDSW200007839-1a_HJLNVDSXY_L4_1_contigs_40w_500min.13
Agarum clathratum	Seq32263-renamed-S20_DDSW200007839-1a_HJLNVDSXY_L4_1_contigs_40w_500min.15
Agarum clathratum	Seq35673-renamed-S20_DDSW200007839-1a_HJLNVDSXY_L4_1_contigs_40w_500min.3
Agarum clathratum	Seq37118-renamed-S20_DDSW200007839-1a_HJLNVDSXY_L4_1_contigs_40w_500min.11
Agarum clathratum	Seq37447-renamed-S20_DDSW200007839-1a_HJLNVDSXY_L4_1_contigs_40w_500min.1
Agarum clathratum	Seq38106-renamed-S20_DDSW200007839-1a_HJLNVDSXY_L4_1_contigs_40w_500min.3
Agarum clathratum	Seq41247-renamed-S20_DDSW200007839-1a_HJLNVDSXY_L4_1_contigs_40w_500min.1
Agarum clathratum	Seq48201-renamed-S20_DDSW200007839-1a_HJLNVDSXY_L4_1_contigs_40w_500min.12
Agarum clathratum	Seq49392-renamed-S20_DDSW200007839-1a_HJLNVDSXY_L4_1_contigs_40w_500min.1
Agarum clathratum	Seq5113-renamed-S20_DDSW200007839-1a_HJLNVDSXY_L4_1_contigs_40w_500min.2
Agarum clathratum	Seq51638-renamed-S20_DDSW200007839-1a_HJLNVDSXY_L4_1_contigs_40w_500min.1
Agarum clathratum	Seq51639-renamed-S20_DDSW200007839-1a_HJLNVDSXY_L4_1_contigs_40w_500min.1
Agarum clathratum	Seq5792-renamed-S20_DDSW200007839-1a_HJLNVDSXY_L4_1_contigs_40w_500min.4
Agarum clathratum	Seq64637-renamed-S20_DDSW200007839-1a_HJLNVDSXY_L4_1_contigs_40w_500min.4
Agarum clathratum	Seq66085-renamed-S20_DDSW200007839-1a_HJLNVDSXY_L4_1_contigs_40w_500min.1
Agarum clathratum	Seq70692-renamed-S20_DDSW200007839-1a_HJLNVDSXY_L4_1_contigs_40w_500min.2
Agarum clathratum	Seq71971-renamed-S20_DDSW200007839-1a_HJLNVDSXY_L4_1_contigs_40w_500min.1
Agarum clathratum	Seq77195-renamed-S20_DDSW200007839-1a_HJLNVDSXY_L4_1_contigs_40w_500min.1
Agarum clathratum	Seq83644-renamed-S20_DDSW200007839-1a_HJLNVDSXY_L4_1_contigs_40w_500min.6
Alethocladus corymbosus	Seq11358-2_CCMP111_Dneasy_Alethocladus_corymbosus_Brown_100GB_Genohub_contigs_40w_500m.3
Alethocladus corymbosus	Seq146720-2_CCMP111_Dneasy_Alethocladus_corymbosus_Brown_100GB_Genohub_contigs_40w_500m.1
Alethocladus corymbosus	Seq146720-2_CCMP111_Dneasy_Alethocladus_corymbosus_Brown_100GB_Genohub_contigs_40w_500m.2
Alethocladus corymbosus	Seq62911-2_CCMP111_Dneasy_Alethocladus_corymbosus_Brown_100GB_Genohub_contigs_40w_500m.3
Alethocladus corymbosus	Seq90536-2_CCMP111_Dneasy_Alethocladus_corymbosus_Brown_100GB_Genohub_contigs_40w_500m.1
Anotrichium barbatum	Seq22818-S19_FSFP210375660-2r_HMJ5NDSX2_L1_1_40w_500m.4
Anotrichium barbatum	Seq3129-S19_FSFP210375660-2r_HMJ5NDSX2_L1_1_40w_500m.5
Ascoseira mirabilis	Seq11835-renamed-19106D-06-04_S0_L001_contigs_60w_500min.4
Ascoseira mirabilis	Seq12246-renamed-19106D-06-04_S0_L001_contigs_60w_500min.4
Ascoseira mirabilis	Seq14507-renamed-19106D-06-04_S0_L001_contigs_60w_500min.9
Ascoseira mirabilis	Seq50408-renamed-19106D-06-04_S0_L001_contigs_60w_500min.1
Ascoseira mirabilis	Seq8924-renamed-19106D-06-04_S0_L001_contigs_60w_500min.21
Bodanella lauderbornii	Seq983-renamed-19106D-08-01_S5_L003_contigs_60w_500m.10
Botrytella micromora	Seq19208-renamed-S8_FDSW210282399-1r_HTNYJDSXY_L4_1_contigs_60w_500min.9
Botrytella micromora	Seq2181-renamed-S8_FDSW210282399-1r_HTNYJDSXY_L4_1_contigs_60w_500min.8
Botrytella micromora	Seq25814-renamed-S8_FDSW210282399-1r_HTNYJDSXY_L4_1_contigs_60w_500min.6
Botrytella micromora	Seq33757-renamed-S8_FDSW210282399-1r_HTNYJDSXY_L4_1_contigs_60w_500min.3
Botrytella micromora	Seq41192-renamed-S8_FDSW210282399-1r_HTNYJDSXY_L4_1_contigs_60w_500min.5
Botrytella micromora	Seq43154-renamed-S8_FDSW210282399-1r_HTNYJDSXY_L4_1_contigs_60w_500min.4

Botrytella micromora	Seq43819-renamed-S8_FDSW210282399-1r_HTNYJDSXY_L4_1_contigs_60w_500min.3
Botrytella uvaeformis	Seq26454-S17_FDSW210282408-1b_H722NDSX2_L4_1_contigs_40w_500m.7
Botrytella uvaeformis	Seq26718-S17_FDSW210282408-1b_H722NDSX2_L4_1_contigs_40w_500m.9
Botrytella uvaeformis	Seq30773-S17_FDSW210282408-1b_H722NDSX2_L4_1_contigs_40w_500m.2
Botrytella uvaeformis	Seq30773-S17_FDSW210282408-1b_H722NDSX2_L4_1_contigs_40w_500m.3
Botrytella uvaeformis	Seq34707-S17_FDSW210282408-1b_H722NDSX2_L4_1_contigs_40w_500m.2
Botrytella uvaeformis	Seq48900-S17_FDSW210282408-1b_H722NDSX2_L4_1_contigs_40w_500m.1
Botrytella uvaeformis	Seq51127-S17_FDSW210282408-1b_H722NDSX2_L4_1_contigs_40w_500m.1
Botrytella uvaeformis	Seq6820-S17_FDSW210282408-1b_H722NDSX2_L4_1_contigs_40w_500m.1
Canistrocarpus cervicornis	Seq136920-5_1MIC_S4_contigs_40w_500m.1
Canistrocarpus cervicornis	Seq152849-5_1MIC_S4_contigs_40w_500m.1
Canistrocarpus cervicornis	Seq22973-5_1MIC_S4_contigs_40w_500m.1
Canistrocarpus cervicornis	Seq245320-5_1MIC_S4_contigs_40w_500m.1
Canistrocarpus cervicornis	Seq285575-5_1MIC_S4_contigs_40w_500m.1
Canistrocarpus cervicornis	Seq295160-5_1MIC_S4_contigs_40w_500m.1
Canistrocarpus cervicornis	Seq88756-5_1MIC_S4_contigs_40w_500m.1
Canistrocarpus cervicornis	Seq92091-5_1MIC_S4_contigs_40w_500m.1
Canistrocarpus cervicornis	Seq98916-5_1MIC_S4_contigs_40w_500m.1
Carpomitra costata	Seq24202-renamed-S12_FDSW210282403-2r_HTWJ3DSXY_L1_1_contigs_40w_500m.3
Carpomitra costata	Seq26606-renamed-S12_FDSW210282403-2r_HTWJ3DSXY_L1_1_contigs_40w_500m.6
Carpomitra costata	Seq27075-renamed-S12_FDSW210282403-2r_HTWJ3DSXY_L1_1_contigs_40w_500m.4
Carpomitra costata	Seq27075-renamed-S12_FDSW210282403-2r_HTWJ3DSXY_L1_1_contigs_40w_500m.5
Carpomitra costata	Seq3115-renamed-S12_FDSW210282403-2r_HTWJ3DSXY_L1_1_contigs_40w_500m.3
Carpomitra costata	Seq3756-renamed-S12_FDSW210282403-2r_HTWJ3DSXY_L1_1_contigs_40w_500m.2
Carpomitra costata	Seq49366-renamed-S12_FDSW210282403-2r_HTWJ3DSXY_L1_1_contigs_40w_500m.1
Carpomitra costata	Seq49680-renamed-S12_FDSW210282403-2r_HTWJ3DSXY_L1_1_contigs_40w_500m.1
Carpomitra costata	Seq50747-renamed-S12_FDSW210282403-2r_HTWJ3DSXY_L1_1_contigs_40w_500m.2
Carpomitra costata	Seq51367-renamed-S12_FDSW210282403-2r_HTWJ3DSXY_L1_1_contigs_40w_500m.1
Desmarestia aculeata	Seq10267-renamed-S4_DDSW200007823-1a_HJLY3DSXY_L2_1_contigs_60w_500min.14
Desmarestia aculeata	Seq11861-renamed-S4_DDSW200007823-1a_HJLY3DSXY_L2_1_contigs_60w_500min.4
Desmarestia aculeata	Seq12747-renamed-S4_DDSW200007823-1a_HJLY3DSXY_L2_1_contigs_60w_500min.7
Desmarestia aculeata	Seq14645-renamed-S4_DDSW200007823-1a_HJLY3DSXY_L2_1_contigs_60w_500min.7
Desmarestia aculeata	Seq14646-renamed-S4_DDSW200007823-1a_HJLY3DSXY_L2_1_contigs_60w_500min.10
Desmarestia aculeata	Seq15330-renamed-S4_DDSW200007823-1a_HJLY3DSXY_L2_1_contigs_60w_500min.16
Desmarestia aculeata	Seq17515-renamed-S4_DDSW200007823-1a_HJLY3DSXY_L2_1_contigs_60w_500min.1
Desmarestia aculeata	Seq17725-renamed-S4_DDSW200007823-1a_HJLY3DSXY_L2_1_contigs_60w_500min.6
Desmarestia aculeata	Seq17725-renamed-S4_DDSW200007823-1a_HJLY3DSXY_L2_1_contigs_60w_500min.9
Desmarestia aculeata	Seq17726-renamed-S4_DDSW200007823-1a_HJLY3DSXY_L2_1_contigs_60w_500min.26
Desmarestia aculeata	Seq18202-renamed-S4_DDSW200007823-1a_HJLY3DSXY_L2_1_contigs_60w_500min.2
Desmarestia aculeata	Seq18203-renamed-S4_DDSW200007823-1a_HJLY3DSXY_L2_1_contigs_60w_500min.9
Desmarestia aculeata	Seq21835-renamed-S4_DDSW200007823-1a_HJLY3DSXY_L2_1_contigs_60w_500min.5
Desmarestia aculeata	Seq21836-renamed-S4_DDSW200007823-1a_HJLY3DSXY_L2_1_contigs_60w_500min.4
Desmarestia aculeata	Seq21836-renamed-S4_DDSW200007823-1a_HJLY3DSXY_L2_1_contigs_60w_500min.5
Desmarestia aculeata	Seq21837-renamed-S4_DDSW200007823-1a_HJLY3DSXY_L2_1_contigs_60w_500min.2
Desmarestia aculeata	Seq29443-renamed-S4_DDSW200007823-1a_HJLY3DSXY_L2_1_contigs_60w_500min.9
Desmarestia aculeata	Seq30144-renamed-S4_DDSW200007823-1a_HJLY3DSXY_L2_1_contigs_60w_500min.1
Desmarestia aculeata	Seq30144-renamed-S4_DDSW200007823-1a_HJLY3DSXY_L2_1_contigs_60w_500min.2
Desmarestia aculeata	Seq31005-renamed-S4_DDSW200007823-1a_HJLY3DSXY_L2_1_contigs_60w_500min.6
Desmarestia aculeata	Seq3177-renamed-S4_DDSW200007823-1a_HJLY3DSXY_L2_1_contigs_60w_500min.4
Desmarestia aculeata	Seq3465-renamed-S4_DDSW200007823-1a_HJLY3DSXY_L2_1_contigs_60w_500min.9
Desmarestia aculeata	Seq39845-renamed-S4_DDSW200007823-1a_HJLY3DSXY_L2_1_contigs_60w_500min.2
Desmarestia aculeata	Seq4220-renamed-S4_DDSW200007823-1a_HJLY3DSXY_L2_1_contigs_60w_500min.2
Desmarestia aculeata	Seq4518-renamed-S4_DDSW200007823-1a_HJLY3DSXY_L2_1_contigs_60w_500min.4
Desmarestia aculeata	Seq5578-renamed-S4_DDSW200007823-1a_HJLY3DSXY_L2_1_contigs_60w_500min.1
Desmarestia aculeata	Seq6537-renamed-S4_DDSW200007823-1a_HJLY3DSXY_L2_1_contigs_60w_500min.10
Desmarestia aculeata	Seq8071-renamed-S4_DDSW200007823-1a_HJLY3DSXY_L2_1_contigs_60w_500min.1
Desmarestia aculeata	Seq8278-renamed-S4_DDSW200007823-1a_HJLY3DSXY_L2_1_contigs_60w_500min.9
Desmarestia aculeata	Seq84766-renamed-S4_DDSW200007823-1a_HJLY3DSXY_L2_1_contigs_60w_500min.2
Desmarestia aculeata	Seq87532-renamed-S4_DDSW200007823-1a_HJLY3DSXY_L2_1_contigs_60w_500min.1
Desmarestia aculeata	Seq9609-renamed-S4_DDSW200007823-1a_HJLY3DSXY_L2_1_contigs_60w_500min.6
Desmarestia anceps	Seq10581-renamed-19106D-06-06_S322_L004_contigs_60w_500min.6
Desmarestia anceps	Seq12883-renamed-19106D-06-06_S322_L004_contigs_60w_500min.11
Desmarestia anceps	Seq13162-renamed-19106D-06-06_S322_L004_contigs_60w_500min.11
Desmarestia anceps	Seq14383-renamed-19106D-06-06_S322_L004_contigs_60w_500min.6
Desmarestia anceps	Seq14556-renamed-19106D-06-06_S322_L004_contigs_60w_500min.8
Desmarestia anceps	Seq15393-renamed-19106D-06-06_S322_L004_contigs_60w_500min.12

Desmarestia anceps	Seq17117-renamed-19106D-06-06_S322_L004_contigs_60w_500min.3
Desmarestia anceps	Seq1754-renamed-19106D-06-06_S322_L004_contigs_60w_500min.9
Desmarestia anceps	Seq18808-renamed-19106D-06-06_S322_L004_contigs_60w_500min.3
Desmarestia anceps	Seq20044-renamed-19106D-06-06_S322_L004_contigs_60w_500min.2
Desmarestia anceps	Seq24982-renamed-19106D-06-06_S322_L004_contigs_60w_500min.2
Desmarestia anceps	Seq2910-renamed-19106D-06-06_S322_L004_contigs_60w_500min.8
Desmarestia anceps	Seq29353-renamed-19106D-06-06_S322_L004_contigs_60w_500min.6
Desmarestia anceps	Seq3109-renamed-19106D-06-06_S322_L004_contigs_60w_500min.8
Desmarestia anceps	Seq32368-renamed-19106D-06-06_S322_L004_contigs_60w_500min.3
Desmarestia anceps	Seq37906-renamed-19106D-06-06_S322_L004_contigs_60w_500min.3
Desmarestia anceps	Seq37906-renamed-19106D-06-06_S322_L004_contigs_60w_500min.5
Desmarestia anceps	Seq39081-renamed-19106D-06-06_S322_L004_contigs_60w_500min.1
Desmarestia anceps	Seq44957-renamed-19106D-06-06_S322_L004_contigs_60w_500min.3
Desmarestia anceps	Seq4930-renamed-19106D-06-06_S322_L004_contigs_60w_500min.15
Desmarestia anceps	Seq63718-renamed-19106D-06-06_S322_L004_contigs_60w_500min.1
Desmarestia anceps	Seq6537-renamed-19106D-06-06_S322_L004_contigs_60w_500min.19
Desmarestia anceps	Seq6537-renamed-19106D-06-06_S322_L004_contigs_60w_500min.7
Desmarestia anceps	Seq6537-renamed-19106D-06-06_S322_L004_contigs_60w_500min.8
Desmarestia anceps	Seq7391-renamed-19106D-06-06_S322_L004_contigs_60w_500min.5
Desmarestia anceps	Seq8562-renamed-19106D-06-06_S322_L004_contigs_60w_500min.8
Desmarestia anceps	Seq8694-renamed-19106D-06-06_S322_L004_contigs_60w_500min.9
Dictyota eroica	Seq273962-renamed-S3_FDSW210185064-1r_H73VNDSX2_L2_1_contigs_60w_500min.1
Dictyota eroica	Seq56760-renamed-S3_FDSW210185064-1r_H73VNDSX2_L2_1_contigs_60w_500min.1
Dictyota eroica	Seq68973-renamed-S3_FDSW210185064-1r_H73VNDSX2_L2_1_contigs_60w_500min.1
Dictyota eroica	Seq8011-renamed-S3_FDSW210185064-1r_H73VNDSX2_L2_1_contigs_60w_500min.5
Ectocarpus siliculosus	Seq24-renamed-Ectocarpus_siliculosus_GCA_000310025.1_ASM31002v1_genomic.2451
Ectocarpus sp.	Seq30646-renamed-19106D-08-02_S6_L003_contigs_50w_500m.2
Ectocarpus sp.	Seq65972-renamed-19106D-08-02_S6_L003_contigs_50w_500m.1
Ectocarpus subulatus	Seq28228-renamed-Ectocarpus_subulatus-completeassembly-Esub-CCAP1310-34-CACSHI01.2
Ectocarpus subulatus	Seq51205-renamed-Ectocarpus_subulatus-completeassembly-Esub-CCAP1310-34-CACSHI01.7
Ectocarpus subulatus	Seq53706-renamed-Ectocarpus_subulatus-completeassembly-Esub-CCAP1310-34-CACSHI01.3
Himantothallus grandifolius	Seq10010-renamed-S6_DDSW200007825-1a_HJLY3DSXY_L2_1_contigs_50w_500min.2
Himantothallus grandifolius	Seq10010-renamed-S6_DDSW200007825-1a_HJLY3DSXY_L2_1_contigs_50w_500min.9
Himantothallus grandifolius	Seq11359-renamed-S6_DDSW200007825-1a_HJLY3DSXY_L2_1_contigs_50w_500min.8
Himantothallus grandifolius	Seq11597-renamed-S6_DDSW200007825-1a_HJLY3DSXY_L2_1_contigs_50w_500min.10
Himantothallus grandifolius	Seq11598-renamed-S6_DDSW200007825-1a_HJLY3DSXY_L2_1_contigs_50w_500min.10
Himantothallus grandifolius	Seq1776-renamed-S6_DDSW200007825-1a_HJLY3DSXY_L2_1_contigs_50w_500min.6
Himantothallus grandifolius	Seq20985-renamed-S6_DDSW200007825-1a_HJLY3DSXY_L2_1_contigs_50w_500min.10
Himantothallus grandifolius	Seq23932-renamed-S6_DDSW200007825-1a_HJLY3DSXY_L2_1_contigs_50w_500min.2
Himantothallus grandifolius	Seq26473-renamed-S6_DDSW200007825-1a_HJLY3DSXY_L2_1_contigs_50w_500min.3
Himantothallus grandifolius	Seq3023-renamed-S6_DDSW200007825-1a_HJLY3DSXY_L2_1_contigs_50w_500min.8
Himantothallus grandifolius	Seq44872-renamed-S6_DDSW200007825-1a_HJLY3DSXY_L2_1_contigs_50w_500min.4
Himantothallus grandifolius	Seq4691-renamed-S6_DDSW200007825-1a_HJLY3DSXY_L2_1_contigs_50w_500min.19
Himantothallus grandifolius	Seq50154-renamed-S6_DDSW200007825-1a_HJLY3DSXY_L2_1_contigs_50w_500min.1
Himantothallus grandifolius	Seq5536-renamed-S6_DDSW200007825-1a_HJLY3DSXY_L2_1_contigs_50w_500min.9
Himantothallus grandifolius	Seq55809-renamed-S6_DDSW200007825-1a_HJLY3DSXY_L2_1_contigs_50w_500min.2
Himantothallus grandifolius	Seq6091-renamed-S6_DDSW200007825-1a_HJLY3DSXY_L2_1_contigs_50w_500min.13
Himantothallus grandifolius	Seq9115-renamed-S6_DDSW200007825-1a_HJLY3DSXY_L2_1_contigs_50w_500min.9
Himantothallus grandifolius	Seq9965-renamed-S6_DDSW200007825-1a_HJLY3DSXY_L2_1_contigs_50w_500min.1
Hincksia sandriana	Seq10097-S18_FSFP210375659-1r_HLF3FDSX2_L4_1_40w_500m.2
Hincksia sandriana	Seq35712-S18_FSFP210375659-1r_HLF3FDSX2_L4_1_40w_500m.1
Hincksia sandriana	Seq76122-S18_FSFP210375659-1r_HLF3FDSX2_L4_1_40w_500m.2
Laminaria digitata	Seq10287-renamed-S8_DDSW200007827-1a_HJLNVDSXY_L3_1_contigs_50w_500min.2
Laminaria digitata	Seq12916-renamed-S8_DDSW200007827-1a_HJLNVDSXY_L3_1_contigs_50w_500min.6
Laminaria digitata	Seq12916-renamed-S8_DDSW200007827-1a_HJLNVDSXY_L3_1_contigs_50w_500min.8
Laminaria digitata	Seq13179-renamed-S8_DDSW200007827-1a_HJLNVDSXY_L3_1_contigs_50w_500min.16
Laminaria digitata	Seq15873-renamed-S8_DDSW200007827-1a_HJLNVDSXY_L3_1_contigs_50w_500min.8
Laminaria digitata	Seq20435-renamed-S8_DDSW200007827-1a_HJLNVDSXY_L3_1_contigs_50w_500min.22
Laminaria digitata	Seq20436-renamed-S8_DDSW200007827-1a_HJLNVDSXY_L3_1_contigs_50w_500min.10
Laminaria digitata	Seq21802-renamed-S8_DDSW200007827-1a_HJLNVDSXY_L3_1_contigs_50w_500min.3
Laminaria digitata	Seq21802-renamed-S8_DDSW200007827-1a_HJLNVDSXY_L3_1_contigs_50w_500min.5
Laminaria digitata	Seq23532-renamed-S8_DDSW200007827-1a_HJLNVDSXY_L3_1_contigs_50w_500min.6
Laminaria digitata	Seq24099-renamed-S8_DDSW200007827-1a_HJLNVDSXY_L3_1_contigs_50w_500min.15
Laminaria digitata	Seq2732-renamed-S8_DDSW200007827-1a_HJLNVDSXY_L3_1_contigs_50w_500min.12
Laminaria digitata	Seq28696-renamed-S8_DDSW200007827-1a_HJLNVDSXY_L3_1_contigs_50w_500min.3
Laminaria digitata	Seq30653-renamed-S8_DDSW200007827-1a_HJLNVDSXY_L3_1_contigs_50w_500min.7

Laminaria digitata	Seq30654-renamed-S8_DDSW200007827-1a_HJLNVDSXY_L3_1_contigs_50w_500min.2
Laminaria digitata	Seq38469-renamed-S8_DDSW200007827-1a_HJLNVDSXY_L3_1_contigs_50w_500min.10
Laminaria digitata	Seq39272-renamed-S8_DDSW200007827-1a_HJLNVDSXY_L3_1_contigs_50w_500min.10
Laminaria digitata	Seq45176-renamed-S8_DDSW200007827-1a_HJLNVDSXY_L3_1_contigs_50w_500min.9
Laminaria digitata	Seq49284-renamed-S8_DDSW200007827-1a_HJLNVDSXY_L3_1_contigs_50w_500min.3
Laminaria digitata	Seq49288-renamed-S8_DDSW200007827-1a_HJLNVDSXY_L3_1_contigs_50w_500min.1
Laminaria digitata	Seq49288-renamed-S8_DDSW200007827-1a_HJLNVDSXY_L3_1_contigs_50w_500min.4
Laminaria digitata	Seq4965-renamed-S8_DDSW200007827-1a_HJLNVDSXY_L3_1_contigs_50w_500min.11
Laminaria digitata	Seq52270-renamed-S8_DDSW200007827-1a_HJLNVDSXY_L3_1_contigs_50w_500min.1
Laminaria digitata	Seq55071-renamed-S8_DDSW200007827-1a_HJLNVDSXY_L3_1_contigs_50w_500min.6
Laminaria digitata	Seq56348-renamed-S8_DDSW200007827-1a_HJLNVDSXY_L3_1_contigs_50w_500min.7
Laminaria digitata	Seq57048-renamed-S8_DDSW200007827-1a_HJLNVDSXY_L3_1_contigs_50w_500min.2
Laminaria digitata	Seq59258-renamed-S8_DDSW200007827-1a_HJLNVDSXY_L3_1_contigs_50w_500min.2
Laminaria digitata	Seq65038-renamed-S8_DDSW200007827-1a_HJLNVDSXY_L3_1_contigs_50w_500min.6
Laminaria digitata	Seq674-renamed-S8_DDSW200007827-1a_HJLNVDSXY_L3_1_contigs_50w_500min.3
Laminaria digitata	Seq868-renamed-S8_DDSW200007827-1a_HJLNVDSXY_L3_1_contigs_50w_500min.13
Laminaria digitata	Seq95846-renamed-S8_DDSW200007827-1a_HJLNVDSXY_L3_1_contigs_50w_500min.1
Laminariocolax aecidoides	Seq11254-renamed-S9_DDSW200007828-2a_HKKN3DSXY_L4_1_contigs_60w_500min.13
Laminariocolax aecidoides	Seq1546-renamed-S9_DDSW200007828-2a_HKKN3DSXY_L4_1_contigs_60w_500min.31
Laminariocolax aecidoides	Seq1546-renamed-S9_DDSW200007828-2a_HKKN3DSXY_L4_1_contigs_60w_500min.33
Laminariocolax aecidoides	Seq15861-renamed-S9_DDSW200007828-2a_HKKN3DSXY_L4_1_contigs_60w_500min.1
Laminariocolax aecidoides	Seq1990-renamed-S9_DDSW200007828-2a_HKKN3DSXY_L4_1_contigs_60w_500min.16
Laminariocolax aecidoides	Seq3909-renamed-S9_DDSW200007828-2a_HKKN3DSXY_L4_1_contigs_60w_500min.9
Laminariocolax aecidoides	Seq5306-renamed-S9_DDSW200007828-2a_HKKN3DSXY_L4_1_contigs_60w_500min.17
Laminariocolax aecidoides	Seq5720-renamed-S9_DDSW200007828-2a_HKKN3DSXY_L4_1_contigs_60w_500min.6
Laminariocolax aecidoides	Seq7672-renamed-S9_DDSW200007828-2a_HKKN3DSXY_L4_1_contigs_60w_500min.2
Laminariocolax aecidoides	Seq7672-renamed-S9_DDSW200007828-2a_HKKN3DSXY_L4_1_contigs_60w_500min.4
Laminariocolax aecidoides	Seq805-renamed-S9_DDSW200007828-2a_HKKN3DSXY_L4_1_contigs_60w_500min.13
Laminariocolax aecidoides	Seq9844-renamed-S9_DDSW200007828-2a_HKKN3DSXY_L4_1_contigs_60w_500min.5
Laminarionema elsbetiae	Seq14284-renamed-S13_FDSW210282404-2r_H5KY2DSX2_L1_1_contigs_60w_500m.4
Laminarionema elsbetiae	Seq15973-renamed-S13_FDSW210282404-2r_H5KY2DSX2_L1_1_contigs_60w_500m.11
Laminarionema elsbetiae	Seq17342-renamed-S13_FDSW210282404-2r_H5KY2DSX2_L1_1_contigs_60w_500m.4
Laminarionema elsbetiae	Seq1885-renamed-S13_FDSW210282404-2r_H5KY2DSX2_L1_1_contigs_60w_500m.4
Laminarionema elsbetiae	Seq19960-renamed-S13_FDSW210282404-2r_H5KY2DSX2_L1_1_contigs_60w_500m.6
Laminarionema elsbetiae	Seq24411-renamed-S13_FDSW210282404-2r_H5KY2DSX2_L1_1_contigs_60w_500m.1
Laminarionema elsbetiae	Seq24411-renamed-S13_FDSW210282404-2r_H5KY2DSX2_L1_1_contigs_60w_500m.3
Laminarionema elsbetiae	Seq25644-renamed-S13_FDSW210282404-2r_H5KY2DSX2_L1_1_contigs_60w_500m.3
Laminarionema elsbetiae	Seq2820-renamed-S13_FDSW210282404-2r_H5KY2DSX2_L1_1_contigs_60w_500m.2
Laminarionema elsbetiae	Seq28700-renamed-S13_FDSW210282404-2r_H5KY2DSX2_L1_1_contigs_60w_500m.3
Laminarionema elsbetiae	Seq31631-renamed-S13_FDSW210282404-2r_H5KY2DSX2_L1_1_contigs_60w_500m.7
Laminarionema elsbetiae	Seq34919-renamed-S13_FDSW210282404-2r_H5KY2DSX2_L1_1_contigs_60w_500m.3
Laminarionema elsbetiae	Seq535-renamed-S13_FDSW210282404-2r_H5KY2DSX2_L1_1_contigs_60w_500m.2
Laminarionema elsbetiae	Seq6287-renamed-S13_FDSW210282404-2r_H5KY2DSX2_L1_1_contigs_60w_500m.5
Laminarionema elsbetiae	Seq9549-renamed-S13_FDSW210282404-2r_H5KY2DSX2_L1_1_contigs_60w_500m.3
Macrocystis pyrifera	Seq10306-renamed-S10_DDSW200007829-1a_HJT2VDSXY_L1_1_contigs_60w_500min.22
Macrocystis pyrifera	Seq10306-renamed-S10_DDSW200007829-1a_HJT2VDSXY_L1_1_contigs_60w_500min.6
Macrocystis pyrifera	Seq10845-renamed-S10_DDSW200007829-1a_HJT2VDSXY_L1_1_contigs_60w_500min.1
Macrocystis pyrifera	Seq11844-renamed-S10_DDSW200007829-1a_HJT2VDSXY_L1_1_contigs_60w_500min.7
Macrocystis pyrifera	Seq12059-renamed-S10_DDSW200007829-1a_HJT2VDSXY_L1_1_contigs_60w_500min.12
Macrocystis pyrifera	Seq13142-renamed-S10_DDSW200007829-1a_HJT2VDSXY_L1_1_contigs_60w_500min.2
Macrocystis pyrifera	Seq14236-renamed-S10_DDSW200007829-1a_HJT2VDSXY_L1_1_contigs_60w_500min.8
Macrocystis pyrifera	Seq15064-renamed-S10_DDSW200007829-1a_HJT2VDSXY_L1_1_contigs_60w_500min.7
Macrocystis pyrifera	Seq15064-renamed-S10_DDSW200007829-1a_HJT2VDSXY_L1_1_contigs_60w_500min.8
Macrocystis pyrifera	Seq15891-renamed-S10_DDSW200007829-1a_HJT2VDSXY_L1_1_contigs_60w_500min.3
Macrocystis pyrifera	Seq17154-renamed-S10_DDSW200007829-1a_HJT2VDSXY_L1_1_contigs_60w_500min.10
Macrocystis pyrifera	Seq17710-renamed-S10_DDSW200007829-1a_HJT2VDSXY_L1_1_contigs_60w_500min.8
Macrocystis pyrifera	Seq19483-renamed-S10_DDSW200007829-1a_HJT2VDSXY_L1_1_contigs_60w_500min.3
Macrocystis pyrifera	Seq19629-renamed-S10_DDSW200007829-1a_HJT2VDSXY_L1_1_contigs_60w_500min.3
Macrocystis pyrifera	Seq19629-renamed-S10_DDSW200007829-1a_HJT2VDSXY_L1_1_contigs_60w_500min.4
Macrocystis pyrifera	Seq19629-renamed-S10_DDSW200007829-1a_HJT2VDSXY_L1_1_contigs_60w_500min.6
Macrocystis pyrifera	Seq2179-renamed-S10_DDSW200007829-1a_HJT2VDSXY_L1_1_contigs_60w_500min.5
Macrocystis pyrifera	Seq24140-renamed-S10_DDSW200007829-1a_HJT2VDSXY_L1_1_contigs_60w_500min.11
Macrocystis pyrifera	Seq24140-renamed-S10_DDSW200007829-1a_HJT2VDSXY_L1_1_contigs_60w_500min.13
Macrocystis pyrifera	Seq24979-renamed-S10_DDSW200007829-1a_HJT2VDSXY_L1_1_contigs_60w_500min.12
Macrocystis pyrifera	Seq25482-renamed-S10_DDSW200007829-1a_HJT2VDSXY_L1_1_contigs_60w_500min.8
Macrocystis pyrifera	Seq33019-renamed-S10_DDSW200007829-1a_HJT2VDSXY_L1_1_contigs_60w_500min.1

Macrocystis pyrifera	Seq35437-renamed-S10_DDSW200007829-1a_HJT2VDSXY_L1_1_contigs_60w_500min.7
Macrocystis pyrifera	Seq37345-renamed-S10_DDSW200007829-1a_HJT2VDSXY_L1_1_contigs_60w_500min.2
Macrocystis pyrifera	Seq43219-renamed-S10_DDSW200007829-1a_HJT2VDSXY_L1_1_contigs_60w_500min.5
Macrocystis pyrifera	Seq4788-renamed-S10_DDSW200007829-1a_HJT2VDSXY_L1_1_contigs_60w_500min.7
Macrocystis pyrifera	Seq4790-renamed-S10_DDSW200007829-1a_HJT2VDSXY_L1_1_contigs_60w_500min.1
Macrocystis pyrifera	Seq5274-renamed-S10_DDSW200007829-1a_HJT2VDSXY_L1_1_contigs_60w_500min.37
Macrocystis pyrifera	Seq5274-renamed-S10_DDSW200007829-1a_HJT2VDSXY_L1_1_contigs_60w_500min.7
Macrocystis pyrifera	Seq5907-renamed-S10_DDSW200007829-1a_HJT2VDSXY_L1_1_contigs_60w_500min.8
Macrocystis pyrifera	Seq5907-renamed-S10_DDSW200007829-1a_HJT2VDSXY_L1_1_contigs_60w_500min.9
Macrocystis pyrifera	Seq7123-renamed-S10_DDSW200007829-1a_HJT2VDSXY_L1_1_contigs_60w_500min.5
Macrocystis pyrifera	Seq810-renamed-S10_DDSW200007829-1a_HJT2VDSXY_L1_1_contigs_60w_500min.1
Macrocystis pyrifera	Seq8464-renamed-S10_DDSW200007829-1a_HJT2VDSXY_L1_1_contigs_60w_500min.37
Macrocystis pyrifera	Seq9206-renamed-S10_DDSW200007829-1a_HJT2VDSXY_L1_1_contigs_60w_500min.15
Macrocystis pyrifera	Seq9206-renamed-S10_DDSW200007829-1a_HJT2VDSXY_L1_1_contigs_60w_500min.27
Macrocystis pyrifera	Seq9367-renamed-S10_DDSW200007829-1a_HJT2VDSXY_L1_1_contigs_60w_500min.31
Macrocystis pyrifera	Seq9753-renamed-S10_DDSW200007829-1a_HJT2VDSXY_L1_1_contigs_60w_500min.30
Microspongium alariae	Seq5820-renamed-S25_FDSW210282416-2r_H5L2YDSX2_L1_1_contigs_60w_500m.23
Microspongium alariae	Seq8945-renamed-S25_FDSW210282416-2r_H5L2YDSX2_L1_1_contigs_60w_500m.2
Microzonia velutina	Seq16981-renamed-S26_FDSW210282417-1r_HTTF7DSXY_L3_1_contigs_40w_500m.1
Microzonia velutina	Seq180927-renamed-S26_FDSW210282417-1r_HTTF7DSXY_L3_1_contigs_40w_500m.1
Microzonia velutina	Seq6846-renamed-S26_FDSW210282417-1r_HTTF7DSXY_L3_1_contigs_40w_500m.8
Microzonia velutina	Seq83324-renamed-S26_FDSW210282417-1r_HTTF7DSXY_L3_1_contigs_40w_500m.1
Myriogloea chilensis	Seq1175-renamed-S9_FDSW210282400-2r_HTWJ3DSXY_L4_1__contigs_60w_500min.49
Myriogloea chilensis	Seq1790-renamed-S9_FDSW210282400-2r_HTWJ3DSXY_L4_1__contigs_60w_500min.1
Myriogloea chilensis	Seq6956-renamed-S9_FDSW210282400-2r_HTWJ3DSXY_L4_1__contigs_60w_500min.1
Padina boergesenii	Seq238305-1E_S1_contigs_40w_500m.7
Padina boergesenii	Seq294376-1E_S1_contigs_40w_500m.6
Padina boergesenii	Seq524246-1E_S1_contigs_40w_500m.1
Padina boergesenii	Seq53706-1E_S1_contigs_40w_500m.11
Padina boergesenii	Seq571197-1E_S1_contigs_40w_500m.1
Padina boergesenii	Seq89119-1E_S1_contigs_40w_500m.3
Petalonia sp.	Seq8932-renamed-S27_FDSW210282418-2r_HTWJ3DSXY_L1_1_contigs_40w_500m.16
Polycladia myrica	Seq1009-ICS_S2_contigs_60w_500m.13
Polycladia myrica	Seq10169-ICS_S2_contigs_60w_500m.2
Polycladia myrica	Seq20221-ICS_S2_contigs_60w_500m.10
Polycladia myrica	Seq216-ICS_S2_contigs_60w_500m.1
Polycladia myrica	Seq25424-ICS_S2_contigs_60w_500m.6
Polycladia myrica	Seq28561-ICS_S2_contigs_60w_500m.1
Polycladia myrica	Seq32285-ICS_S2_contigs_60w_500m.4
Polycladia myrica	Seq39121-ICS_S2_contigs_60w_500m.5
Polycladia myrica	Seq42719-ICS_S2_contigs_60w_500m.4
Polycladia myrica	Seq4333-ICS_S2_contigs_60w_500m.14
Polycladia myrica	Seq4333-ICS_S2_contigs_60w_500m.15
Polycladia myrica	Seq46009-ICS_S2_contigs_60w_500m.1
Polycladia myrica	Seq5039-ICS_S2_contigs_60w_500m.6
Polycladia myrica	Seq54774-ICS_S2_contigs_60w_500m.1
Polycladia myrica	Seq552-ICS_S2_contigs_60w_500m.9
Polycladia myrica	Seq659-ICS_S2_contigs_60w_500m.7
Polycladia myrica	Seq7690-ICS_S2_contigs_60w_500m.8
Polycladia myrica	Seq7707-ICS_S2_contigs_60w_500m.4
Polycladia myrica	Seq8298-ICS_S2_contigs_60w_500m.15
Pylaiella littoralis	Seq17412-renamed-19106D-07-05_S0_L001_R1_001_contigs_60w_500min.8
Saccharina japonica	Seaweedptg000021lG000060.1
Saccharina japonica	Seaweedptg000044lG000010.1
Saccharina japonica	Seaweedptg000044lG000020.1
Saccharina japonica	Seaweedptg000044lG000030.1
Saccharina japonica	Seaweedptg000044lG000040.1
Saccharina japonica	Seaweedptg000044lG000050.1
Saccharina japonica	Seaweedptg000044lG000060.1
Saccharina japonica	Seaweedptg000044lG000070.1
Saccharina japonica	Seaweedptg000044lG000080.1
Saccharina japonica	Seaweedptg000044lG000090.1
Saccharina japonica	Seaweedptg000044lG000100.1
Saccharina japonica	Seaweedptg000044lG000110.1
Saccharina japonica	Seaweedptg000044lG000120.1
Saccharina japonica	Seaweedptg000044lG000130.1

Saccharina japonica	Seaweedptg000044lG000140.1
Saccharina japonica	Seaweedptg000044lG000150.1
Saccharina japonica	Seaweedptg000044lG000160.1
Saccharina japonica	Seaweedptg000044lG000170.1
Saccharina japonica	Seaweedptg000044lG000180.1
Saccharina japonica	Seaweedptg000044lG000190.1
Saccharina japonica	Seaweedptg000044lG000200.1
Saccharina japonica	Seaweedptg000044lG000210.1
Saccharina japonica	Seaweedptg000044lG000220.1
Saccharina japonica	Seaweedptg000044lG000230.1
Saccharina japonica	Seaweedptg000044lG000240.1
Saccharina japonica	Seaweedptg000044lG000250.1
Saccharina japonica	Seaweedptg000044lG000260.1
Saccharina japonica	Seaweedptg000044lG000280.1
Saccharina japonica	Seaweedptg000044lG000290.1
Saccharina japonica	Seaweedptg000044lG000300.1
Saccharina japonica	Seaweedptg000044lG000310.1
Saccharina japonica	Seaweedptg000044lG000320.1
Saccharina japonica	Seaweedptg000044lG000330.1
Saccharina japonica	Seaweedptg000044lG000340.1
Saccharina japonica	Seaweedptg000044lG000350.1
Saccharina japonica	Seaweedptg000044lG000360.1
Saccharina japonica	Seaweedptg000044lG000370.1
Saccharina japonica	Seaweedptg000044lG000380.1
Saccharina japonica	Seaweedptg000044lG000390.1
Saccharina japonica	Seaweedptg000044lG000400.1
Saccharina japonica	Seaweedptg000044lG000410.1
Saccharina japonica	Seaweedptg000044lG000420.1
Saccharina japonica	Seaweedptg000044lG000430.1
Saccharina japonica	Seaweedptg000044lG000440.1
Saccharina japonica	Seaweedptg000044lG000450.1
Saccharina japonica	Seaweedptg000044lG000460.1
Saccharina japonica	Seaweedptg000044lG000470.1
Saccharina japonica	Seaweedptg000084lG000020.1
Saccharina japonica	Seaweedptg000084lG000030.1
Saccharina japonica	Seaweedptg000084lG000050.1
Saccharina japonica	Seaweedptg000084lG000060.1
Saccharina japonica	Seaweedptg000084lG000070.1
Saccharina japonica	Seaweedptg000084lG000080.1
Saccharina japonica	Seaweedptg000084lG000140.1
Saccharina japonica	Seaweedptg000084lG000160.1
Saccharina japonica	Seaweedptg000208lG000070.1
Saccharina japonica	Seaweedptg000208lG000090.1
Saccharina japonica	Seaweedptg000208lG000100.1
Saccharina japonica	Seaweedptg000208lG000110.1
Saccharina japonica	Seaweedptg000208lG000130.1
Saccharina japonica	Seaweedptg000208lG000140.1
Saccharina japonica	Seaweedptg000208lG000150.1
Saccharina japonica	Seaweedptg000208lG000160.1
Saccharina japonica	Seaweedptg000208lG000170.1
Saccharina japonica	Seaweedptg000208lG000180.1
Saccharina japonica	Seaweedptg000208lG000200.1
Saccharina japonica	Seaweedptg000297lG000010.1
Saccharina japonica	Seaweedptg000297lG000170.1
Saccharina japonica	Seaweedptg000300lG000030.1
Saccharina japonica	Seaweedptg000300lG000040.1
Saccharina japonica	Seaweedptg000300lG000050.1
Saccharina japonica	Seaweedptg000300lG000060.1
Saccharina japonica	Seaweedptg000300lG000070.1
Saccharina japonica	Seaweedptg000300lG000100.1
Saccharina japonica	Seaweedptg000300lG000110.1
Saccharina japonica	Seaweedptg000300lG000120.1
Saccharina japonica	Seaweedptg000300lG000130.1
Saccharina japonica	Seaweedptg000300lG000160.1
Saccharina japonica	Seaweedptg000300lG000180.1
Saccharina japonica	Seaweedptg000300lG000240.1

Saccharina japonica	Seaweedptg000353lG000280.1
Saccharina japonica	Seaweedptg000353lG000290.1
Saccharina japonica	Seaweedptg000353lG000300.1
Saccharina japonica	Seaweedptg000393lG000060.1
Saccharina japonica	Seaweedptg000393lG000070.1
Saccharina japonica	Seaweedptg000504lG000010.1
Saccharina japonica	Seaweedptg001393lG000050.1
Saccharina japonica	Seaweedptg001393lG000060.1
Saccharina latissima	Seq10267-S14_FDSW210004955-1a_HTVHVDSXY_L3_1_contigs_50w_500m.16
Saccharina latissima	Seq11034-S14_FDSW210004955-1a_HTVHVDSXY_L3_1_contigs_50w_500m.2
Saccharina latissima	Seq11892-S14_FDSW210004955-1a_HTVHVDSXY_L3_1_contigs_50w_500m.11
Saccharina latissima	Seq15514-S14_FDSW210004955-1a_HTVHVDSXY_L3_1_contigs_50w_500m.9
Saccharina latissima	Seq15866-S14_FDSW210004955-1a_HTVHVDSXY_L3_1_contigs_50w_500m.5
Saccharina latissima	Seq1684-S14_FDSW210004955-1a_HTVHVDSXY_L3_1_contigs_50w_500m.6
Saccharina latissima	Seq17133-S14_FDSW210004955-1a_HTVHVDSXY_L3_1_contigs_50w_500m.3
Saccharina latissima	Seq17775-S14_FDSW210004955-1a_HTVHVDSXY_L3_1_contigs_50w_500m.6
Saccharina latissima	Seq17775-S14_FDSW210004955-1a_HTVHVDSXY_L3_1_contigs_50w_500m.8
Saccharina latissima	Seq2138-S14_FDSW210004955-1a_HTVHVDSXY_L3_1_contigs_50w_500m.3
Saccharina latissima	Seq2139-S14_FDSW210004955-1a_HTVHVDSXY_L3_1_contigs_50w_500m.5
Saccharina latissima	Seq23043-S14_FDSW210004955-1a_HTVHVDSXY_L3_1_contigs_50w_500m.13
Saccharina latissima	Seq26351-S14_FDSW210004955-1a_HTVHVDSXY_L3_1_contigs_50w_500m.1
Saccharina latissima	Seq26351-S14_FDSW210004955-1a_HTVHVDSXY_L3_1_contigs_50w_500m.3
Saccharina latissima	Seq27175-S14_FDSW210004955-1a_HTVHVDSXY_L3_1_contigs_50w_500m.2
Saccharina latissima	Seq28011-S14_FDSW210004955-1a_HTVHVDSXY_L3_1_contigs_50w_500m.19
Saccharina latissima	Seq28011-S14_FDSW210004955-1a_HTVHVDSXY_L3_1_contigs_50w_500m.25
Saccharina latissima	Seq28112-S14_FDSW210004955-1a_HTVHVDSXY_L3_1_contigs_50w_500m.11
Saccharina latissima	Seq31010-S14_FDSW210004955-1a_HTVHVDSXY_L3_1_contigs_50w_500m.15
Saccharina latissima	Seq3357-S14_FDSW210004955-1a_HTVHVDSXY_L3_1_contigs_50w_500m.10
Saccharina latissima	Seq3357-S14_FDSW210004955-1a_HTVHVDSXY_L3_1_contigs_50w_500m.8
Saccharina latissima	Seq35083-S14_FDSW210004955-1a_HTVHVDSXY_L3_1_contigs_50w_500m.4
Saccharina latissima	Seq36502-S14_FDSW210004955-1a_HTVHVDSXY_L3_1_contigs_50w_500m.3
Saccharina latissima	Seq3743-S14_FDSW210004955-1a_HTVHVDSXY_L3_1_contigs_50w_500m.20
Saccharina latissima	Seq39436-S14_FDSW210004955-1a_HTVHVDSXY_L3_1_contigs_50w_500m.1
Saccharina latissima	Seq4040-S14_FDSW210004955-1a_HTVHVDSXY_L3_1_contigs_50w_500m.16
Saccharina latissima	Seq45031-S14_FDSW210004955-1a_HTVHVDSXY_L3_1_contigs_50w_500m.15
Saccharina latissima	Seq50163-S14_FDSW210004955-1a_HTVHVDSXY_L3_1_contigs_50w_500m.1
Saccharina latissima	Seq50163-S14_FDSW210004955-1a_HTVHVDSXY_L3_1_contigs_50w_500m.2
Saccharina latissima	Seq5505-S14_FDSW210004955-1a_HTVHVDSXY_L3_1_contigs_50w_500m.1
Saccharina latissima	Seq58782-S14_FDSW210004955-1a_HTVHVDSXY_L3_1_contigs_50w_500m.2
Saccharina latissima	Seq6049-S14_FDSW210004955-1a_HTVHVDSXY_L3_1_contigs_50w_500m.13
Saccharina latissima	Seq60515-S14_FDSW210004955-1a_HTVHVDSXY_L3_1_contigs_50w_500m.8
Saccharina latissima	Seq66199-S14_FDSW210004955-1a_HTVHVDSXY_L3_1_contigs_50w_500m.1
Saccharina latissima	Seq7365-S14_FDSW210004955-1a_HTVHVDSXY_L3_1_contigs_50w_500m.10
Saccharina latissima	Seq76934-S14_FDSW210004955-1a_HTVHVDSXY_L3_1_contigs_50w_500m.1
Saccharina latissima	Seq8238-S14_FDSW210004955-1a_HTVHVDSXY_L3_1_contigs_50w_500m.10
Saccharina latissima	Seq8765-S14_FDSW210004955-1a_HTVHVDSXY_L3_1_contigs_50w_500m.3
Saccharina latissima	Seq8765-S14_FDSW210004955-1a_HTVHVDSXY_L3_1_contigs_50w_500m.5
Saccharina latissima	Seq8766-S14_FDSW210004955-1a_HTVHVDSXY_L3_1_contigs_50w_500m.8
Saccharina latissima	Seq94375-S14_FDSW210004955-1a_HTVHVDSXY_L3_1_contigs_50w_500m.1
Sargassum angustifolium	Seq103776-2_6SDSE_S3_contigs_60w_500m.2
Sargassum angustifolium	Seq110824-2_6SDSE_S3_contigs_60w_500m.1
Sargassum angustifolium	Seq124671-2_6SDSE_S3_contigs_60w_500m.2
Sargassum angustifolium	Seq136209-2_6SDSE_S3_contigs_60w_500m.1
Sargassum angustifolium	Seq140572-2_6SDSE_S3_contigs_60w_500m.1
Sargassum angustifolium	Seq14540-2_6SDSE_S3_contigs_60w_500m.1
Sargassum angustifolium	Seq177147-2_6SDSE_S3_contigs_60w_500m.1
Sargassum angustifolium	Seq180150-2_6SDSE_S3_contigs_60w_500m.1
Sargassum angustifolium	Seq187480-2_6SDSE_S3_contigs_60w_500m.1
Sargassum angustifolium	Seq1957-2_6SDSE_S3_contigs_60w_500m.7
Sargassum angustifolium	Seq211682-2_6SDSE_S3_contigs_60w_500m.1
Sargassum angustifolium	Seq221959-2_6SDSE_S3_contigs_60w_500m.2
Sargassum angustifolium	Seq241652-2_6SDSE_S3_contigs_60w_500m.2
Sargassum angustifolium	Seq25181-2_6SDSE_S3_contigs_60w_500m.2
Sargassum angustifolium	Seq25181-2_6SDSE_S3_contigs_60w_500m.3
Sargassum angustifolium	Seq25182-2_6SDSE_S3_contigs_60w_500m.1
Sargassum angustifolium	Seq25182-2_6SDSE_S3_contigs_60w_500m.2

Sargassum angustifolium	Seq25184-2_6SDSE_S3_contigs_60w_500m.1
Sargassum angustifolium	Seq27550-2_6SDSE_S3_contigs_60w_500m.1
Sargassum angustifolium	Seq286-2_6SDSE_S3_contigs_60w_500m.1
Sargassum angustifolium	Seq312168-2_6SDSE_S3_contigs_60w_500m.1
Sargassum angustifolium	Seq37706-2_6SDSE_S3_contigs_60w_500m.4
Sargassum angustifolium	Seq37706-2_6SDSE_S3_contigs_60w_500m.5
Sargassum angustifolium	Seq37707-2_6SDSE_S3_contigs_60w_500m.1
Sargassum angustifolium	Seq45258-2_6SDSE_S3_contigs_60w_500m.1
Sargassum angustifolium	Seq53092-2_6SDSE_S3_contigs_60w_500m.1
Sargassum angustifolium	Seq53092-2_6SDSE_S3_contigs_60w_500m.2
Sargassum angustifolium	Seq6907-2_6SDSE_S3_contigs_60w_500m.3
Sargassum angustifolium	Seq86337-2_6SDSE_S3_contigs_60w_500m.1
Sargassum latifolium	Seq103931-20200302_1_Sargasssum1_sp_1_5SDS_S2_contigs_60w_500m.1
Sargassum latifolium	Seq109916-20200302_1_Sargasssum1_sp_1_5SDS_S2_contigs_60w_500m.1
Sargassum latifolium	Seq113254-20200302_1_Sargasssum1_sp_1_5SDS_S2_contigs_60w_500m.1
Sargassum latifolium	Seq124721-20200302_1_Sargasssum1_sp_1_5SDS_S2_contigs_60w_500m.1
Sargassum latifolium	Seq143745-20200302_1_Sargasssum1_sp_1_5SDS_S2_contigs_60w_500m.1
Sargassum latifolium	Seq15298-20200302_1_Sargasssum1_sp_1_5SDS_S2_contigs_60w_500m.1
Sargassum latifolium	Seq15670-20200302_1_Sargasssum1_sp_1_5SDS_S2_contigs_60w_500m.8
Sargassum latifolium	Seq18221-20200302_1_Sargasssum1_sp_1_5SDS_S2_contigs_60w_500m.10
Sargassum latifolium	Seq18221-20200302_1_Sargasssum1_sp_1_5SDS_S2_contigs_60w_500m.9
Sargassum latifolium	Seq21397-20200302_1_Sargasssum1_sp_1_5SDS_S2_contigs_60w_500m.2
Sargassum latifolium	Seq22792-20200302_1_Sargasssum1_sp_1_5SDS_S2_contigs_60w_500m.2
Sargassum latifolium	Seq25958-20200302_1_Sargasssum1_sp_1_5SDS_S2_contigs_60w_500m.3
Sargassum latifolium	Seq25958-20200302_1_Sargasssum1_sp_1_5SDS_S2_contigs_60w_500m.4
Sargassum latifolium	Seq27627-20200302_1_Sargasssum1_sp_1_5SDS_S2_contigs_60w_500m.4
Sargassum latifolium	Seq27627-20200302_1_Sargasssum1_sp_1_5SDS_S2_contigs_60w_500m.5
Sargassum latifolium	Seq33176-20200302_1_Sargasssum1_sp_1_5SDS_S2_contigs_60w_500m.6
Sargassum latifolium	Seq34769-20200302_1_Sargasssum1_sp_1_5SDS_S2_contigs_60w_500m.2
Sargassum latifolium	Seq53383-20200302_1_Sargasssum1_sp_1_5SDS_S2_contigs_60w_500m.1
Sargassum latifolium	Seq615-20200302_1_Sargasssum1_sp_1_5SDS_S2_contigs_60w_500m.3
Sargassum latifolium	Seq61967-20200302_1_Sargasssum1_sp_1_5SDS_S2_contigs_60w_500m.2
Sargassum latifolium	Seq73945-20200302_1_Sargasssum1_sp_1_5SDS_S2_contigs_60w_500m.1
Sargassum latifolium	Seq80212-20200302_1_Sargasssum1_sp_1_5SDS_S2_contigs_60w_500m.3
Sargassum latifolium	Seq8249-20200302_1_Sargasssum1_sp_1_5SDS_S2_contigs_60w_500m.1
Scytosiphon promiscuus	Seq17405-renamed-19106D-06-05_S321_L004_contigs_60w_500min.3
Scytosiphon promiscuus	Seq5027-renamed-19106D-06-05_S321_L004_contigs_60w_500min.24
Scytosiphon promiscuus	Seq5027-renamed-19106D-06-05_S321_L004_contigs_60w_500min.25
Scytosiphon promiscuus	Seq51078-renamed-19106D-06-05_S321_L004_contigs_60w_500min.1
Scytosiphon promiscuus	Seq5564-renamed-19106D-06-05_S321_L004_contigs_60w_500min.6
Scytosiphon promiscuus	Seq64514-renamed-19106D-06-05_S321_L004_contigs_60w_500min.1
Scytosiphon promiscuus	Seq6462-renamed-19106D-06-05_S321_L004_contigs_60w_500min.3
Scytosiphon promiscuus	Seq79161-renamed-19106D-06-05_S321_L004_contigs_60w_500min.1
Scytosiphon promiscuus	Seq82057-renamed-19106D-06-05_S321_L004_contigs_60w_500min.1
Scytosiphon promiscuus	Seq8989-renamed-19106D-06-05_S321_L004_contigs_60w_500min.1
Scytosiphon promiscuus	Seq9799-renamed-19106D-06-05_S321_L004_contigs_60w_500min.7
Scytothamnus fasciculatus	Seq2077-S18_FDSW210282409-1b_H722NDSX2_L1_1_contigs_60w_500m.112
Scytothamnus fasciculatus	Seq498-S18_FDSW210282409-1b_H722NDSX2_L1_1_contigs_60w_500m.16
Scytothamnus fasciculatus	Seq498-S18_FDSW210282409-1b_H722NDSX2_L1_1_contigs_60w_500m.37
Scytothamnus fasciculatus	Seq498-S18_FDSW210282409-1b_H722NDSX2_L1_1_contigs_60w_500m.39
Scytothamnus fasciculatus	Seq552-S18_FDSW210282409-1b_H722NDSX2_L1_1_contigs_60w_500m.191
Sphacelaria divaricata	Seq103710-S25_FSFP210375666-2r_HKFFJDSX2_L3_1_40w_500m.7
Sphacelaria divaricata	Seq37955-S25_FSFP210375666-2r_HKFFJDSX2_L3_1_40w_500m.2
Sphacelaria divaricata	Seq53361-S25_FSFP210375666-2r_HKFFJDSX2_L3_1_40w_500m.2
Sphacelaria divaricata	Seq94754-S25_FSFP210375666-2r_HKFFJDSX2_L3_1_40w_500m.4
Sphacelaria rigidula	Seq57264-S24_FSFP210375665-2r_HKC7MDSX2_L4_1_40w_500m.1
Sphacelaria rigidula	Seq9991-S24_FSFP210375665-2r_HKC7MDSX2_L4_1_40w_500m.3
Sphacelaria sp.	Seq117382-renamed-S13_DDSW200007832-2a_HKKN3DSXY_L4_1_contigs_40w_500min.1
Sphacelaria sp.	Seq18983-renamed-S13_DDSW200007832-2a_HKKN3DSXY_L4_1_contigs_40w_500min.2
Sphacelaria sp.	Seq20026-renamed-S13_DDSW200007832-2a_HKKN3DSXY_L4_1_contigs_40w_500min.1
Sphacelaria sp.	Seq66192-renamed-S13_DDSW200007832-2a_HKKN3DSXY_L4_1_contigs_40w_500min.6
Sphacelaria sp.	Seq71131-renamed-S13_DDSW200007832-2a_HKKN3DSXY_L4_1_contigs_40w_500min.2
Sphacelaria sp.	Seq9782-renamed-S13_DDSW200007832-2a_HKKN3DSXY_L4_1_contigs_40w_500min.1
Stictyosiphon soriferus	Seq35171-renamed-19106D-06-07_S0_L001_contigs_60w_500min.2
Stictyosiphon soriferus	Seq66685-renamed-19106D-06-07_S0_L001_contigs_60w_500min.1
Stictyosiphon tortilis	Seq10541-S19_FDSW210282410-1r_H725MDSX2_L4_1_contigs_40w_500m.1

Stictyosiphon tortilis	Seq17680-S19_FDSW210282410-1r_H725MDSX2_L4_1_contigs_40w_500m.2
Stictyosiphon tortilis	Seq83682-S19_FDSW210282410-1r_H725MDSX2_L4_1_contigs_40w_500m.1
