# Supplementary material for: Vanadium-Dependent Haloperoxidase Gene Evolution in Brown Algae: Evidence for Horizontal Gene Transfer
Source: Int J Mol Sci. 2025 Jan 16;26(2):716. doi: 10.3390/ijms26020716 (PMC11765636; doi:10.3390/ijms26020716)
Supplement: Supplementary file 1 [file ijms-26-00716-s001.zip › Supplemental Table S2. Accessions of V-HPOs identified from red algae species.pdf]

Supplemental Table S2 . Accessions of V-HPOs identified from red algae species.

Species	**Accessions**
Antithamnion sp.	Seq1449-S21_FSFP210375662-1r_HLF3FDSX2_L4_1_40w_500m.2
Antithamnion sp.	Seq23419-S21_FSFP210375662-1r_HLF3FDSX2_L4_1_40w_500m.1
Antithamnion sp.	Seq32791-S21_FSFP210375662-1r_HLF3FDSX2_L4_1_40w_500m.1
Antithamnion sp.	Seq45422-S21_FSFP210375662-1r_HLF3FDSX2_L4_1_40w_500m.2
Antithamnion sp.	Seq67456-S21_FSFP210375662-1r_HLF3FDSX2_L4_1_40w_500m.1
Antithamnion sp.	Seq93170-S21_FSFP210375662-1r_HLF3FDSX2_L4_1_40w_500m.1
Audouinella boryana	Seq101294-renamed-S21_DDSW200007840-1a_HJLY3DSXY_L2_1_contigs_60w_500min.1
Bostrychia radicans	Seq10449-S13_FSFP210375654-1r_HMJYFDSX2_L1_1_60w_500m.3
Bostrychia radicans	Seq14194-S13_FSFP210375654-1r_HMJYFDSX2_L1_1_60w_500m.59
Bostrychia radicans	Seq168847-S13_FSFP210375654-1r_HMJYFDSX2_L1_1_60w_500m.1
Bostrychia radicans	Seq23933-S13_FSFP210375654-1r_HMJYFDSX2_L1_1_60w_500m.1
Bostrychia radicans	Seq26963-S13_FSFP210375654-1r_HMJYFDSX2_L1_1_60w_500m.1
Bostrychia radicans	Seq31559-S13_FSFP210375654-1r_HMJYFDSX2_L1_1_60w_500m.7
Bostrychia radicans	Seq38075-S13_FSFP210375654-1r_HMJYFDSX2_L1_1_60w_500m.3
Bostrychia radicans	Seq41205-S13_FSFP210375654-1r_HMJYFDSX2_L1_1_60w_500m.3
Bostrychia radicans	Seq42276-S13_FSFP210375654-1r_HMJYFDSX2_L1_1_60w_500m.1
Bostrychia radicans	Seq6217-S13_FSFP210375654-1r_HMJYFDSX2_L1_1_60w_500m.32
Bostrychia radicans	Seq72796-S13_FSFP210375654-1r_HMJYFDSX2_L1_1_60w_500m.23
Bostrychia radicans	Seq91058-S13_FSFP210375654-1r_HMJYFDSX2_L1_1_60w_500m.1
Caloglossa vieillardii	Seq123609-renamed-19106D-06-13_S324_L004_contigs_60w_500min.1
Caloglossa vieillardii	Seq14304-renamed-19106D-06-13_S324_L004_contigs_60w_500min.13
Caloglossa vieillardii	Seq19978-renamed-19106D-06-13_S324_L004_contigs_60w_500min.2
Caloglossa vieillardii	Seq19982-renamed-19106D-06-13_S324_L004_contigs_60w_500min.2
Caloglossa vieillardii	Seq21118-renamed-19106D-06-13_S324_L004_contigs_60w_500min.2
Caloglossa vieillardii	Seq2364-renamed-19106D-06-13_S324_L004_contigs_60w_500min.133
Caloglossa vieillardii	Seq3460-renamed-19106D-06-13_S324_L004_contigs_60w_500min.42
Caloglossa vieillardii	Seq3743-renamed-19106D-06-13_S324_L004_contigs_60w_500min.28
Caloglossa vieillardii	Seq50635-renamed-19106D-06-13_S324_L004_contigs_60w_500min.1
Caloglossa vieillardii	Seq563-renamed-19106D-06-13_S324_L004_contigs_60w_500min.24
Caloglossa vieillardii	Seq59974-renamed-19106D-06-13_S324_L004_contigs_60w_500min.1
Caloglossa vieillardii	Seq6704-renamed-19106D-06-13_S324_L004_contigs_60w_500min.13
Caloglossa vieillardii	Seq8804-renamed-19106D-06-13_S324_L004_contigs_60w_500min.1
Catenella sp.	Seq1052-renamed-19106D-06-08_S312_L003_contigs_40w_500min.35
Catenella sp.	Seq138-renamed-19106D-06-08_S312_L003_contigs_40w_500min.35
Catenella sp.	Seq1390-renamed-19106D-06-08_S312_L003_contigs_40w_500min.4
Catenella sp.	Seq16377-renamed-19106D-06-08_S312_L003_contigs_40w_500min.1
Catenella sp.	Seq1705-renamed-19106D-06-08_S312_L003_contigs_40w_500min.5
Catenella sp.	Seq1896-renamed-19106D-06-08_S312_L003_contigs_40w_500min.1
Catenella sp.	Seq191-renamed-19106D-06-08_S312_L003_contigs_40w_500min.14
Catenella sp.	Seq192-renamed-19106D-06-08_S312_L003_contigs_40w_500min.6
Catenella sp.	Seq1940-renamed-19106D-06-08_S312_L003_contigs_40w_500min.15
Catenella sp.	Seq2087-renamed-19106D-06-08_S312_L003_contigs_40w_500min.9
Catenella sp.	Seq2633-renamed-19106D-06-08_S312_L003_contigs_40w_500min.39
Catenella sp.	Seq3315-renamed-19106D-06-08_S312_L003_contigs_40w_500min.2
Catenella sp.	Seq434-renamed-19106D-06-08_S312_L003_contigs_40w_500min.1
Catenella sp.	Seq4726-renamed-19106D-06-08_S312_L003_contigs_40w_500min.8
Catenella sp.	Seq480-renamed-19106D-06-08_S312_L003_contigs_40w_500min.14
Catenella sp.	Seq4909-renamed-19106D-06-08_S312_L003_contigs_40w_500min.8
Catenella sp.	Seq4947-renamed-19106D-06-08_S312_L003_contigs_40w_500min.8
Catenella sp.	Seq703-renamed-19106D-06-08_S312_L003_contigs_40w_500min.29
Catenella sp.	Seq8-renamed-19106D-06-08_S312_L003_contigs_40w_500min.7
Catenella sp.	Seq94-renamed-19106D-06-08_S312_L003_contigs_40w_500min.90
Chondria dasyphylla	Seq101282-20200302_4_Green2_4_3SDSE_S3_contigs_40w_500m.1
Chondria dasyphylla	Seq104359-20200302_4_Green2_4_3SDSE_S3_contigs_40w_500m.1
Chondria dasyphylla	Seq12950-20200302_4_Green2_4_3SDSE_S3_contigs_40w_500m.1
Chondria dasyphylla	Seq14384-20200302_4_Green2_4_3SDSE_S3_contigs_40w_500m.1
Chondria dasyphylla	Seq1505-20200302_4_Green2_4_3SDSE_S3_contigs_40w_500m.1
Chondria dasyphylla	Seq173623-20200302_4_Green2_4_3SDSE_S3_contigs_40w_500m.1
Chondria dasyphylla	Seq17671-20200302_4_Green2_4_3SDSE_S3_contigs_40w_500m.1
Chondria dasyphylla	Seq185113-20200302_4_Green2_4_3SDSE_S3_contigs_40w_500m.1
Chondria dasyphylla	Seq187352-20200302_4_Green2_4_3SDSE_S3_contigs_40w_500m.3
Chondria dasyphylla	Seq196554-20200302_4_Green2_4_3SDSE_S3_contigs_40w_500m.7

Chondria dasyphylla	Seq24893-20200302_4_Green2_4_3SDSE_S3_contigs_40w_500m.1
Chondria dasyphylla	Seq2586-20200302_4_Green2_4_3SDSE_S3_contigs_40w_500m.1
Chondria dasyphylla	Seq259920-20200302_4_Green2_4_3SDSE_S3_contigs_40w_500m.1
Chondria dasyphylla	Seq261665-20200302_4_Green2_4_3SDSE_S3_contigs_40w_500m.1
Chondria dasyphylla	Seq28785-20200302_4_Green2_4_3SDSE_S3_contigs_40w_500m.1
Chondria dasyphylla	Seq29383-20200302_4_Green2_4_3SDSE_S3_contigs_40w_500m.1
Chondria dasyphylla	Seq2992-20200302_4_Green2_4_3SDSE_S3_contigs_40w_500m.1
Chondria dasyphylla	Seq31296-20200302_4_Green2_4_3SDSE_S3_contigs_40w_500m.1
Chondria dasyphylla	Seq34793-20200302_4_Green2_4_3SDSE_S3_contigs_40w_500m.2
Chondria dasyphylla	Seq36708-20200302_4_Green2_4_3SDSE_S3_contigs_40w_500m.1
Chondria dasyphylla	Seq37807-20200302_4_Green2_4_3SDSE_S3_contigs_40w_500m.1
Chondria dasyphylla	Seq42347-20200302_4_Green2_4_3SDSE_S3_contigs_40w_500m.1
Chondria dasyphylla	Seq42624-20200302_4_Green2_4_3SDSE_S3_contigs_40w_500m.1
Chondria dasyphylla	Seq42626-20200302_4_Green2_4_3SDSE_S3_contigs_40w_500m.1
Chondria dasyphylla	Seq48051-20200302_4_Green2_4_3SDSE_S3_contigs_40w_500m.2
Chondria dasyphylla	Seq48051-20200302_4_Green2_4_3SDSE_S3_contigs_40w_500m.3
Chondria dasyphylla	Seq48839-20200302_4_Green2_4_3SDSE_S3_contigs_40w_500m.5
Chondria dasyphylla	Seq56229-20200302_4_Green2_4_3SDSE_S3_contigs_40w_500m.1
Chondria dasyphylla	Seq59917-20200302_4_Green2_4_3SDSE_S3_contigs_40w_500m.1
Chondria dasyphylla	Seq69585-20200302_4_Green2_4_3SDSE_S3_contigs_40w_500m.1
Chondria dasyphylla	Seq8062-20200302_4_Green2_4_3SDSE_S3_contigs_40w_500m.1
Chondria dasyphylla	Seq83070-20200302_4_Green2_4_3SDSE_S3_contigs_40w_500m.1
Chondria dasyphylla	Seq83424-20200302_4_Green2_4_3SDSE_S3_contigs_40w_500m.2
Chondria dasyphylla	Seq9202-20200302_4_Green2_4_3SDSE_S3_contigs_40w_500m.1
Chondria dasyphylla	Seq962-20200302_4_Green2_4_3SDSE_S3_contigs_40w_500m.1
Chondria dasyphylla	Seq98262-20200302_4_Green2_4_3SDSE_S3_contigs_40w_500m.1
Chondria dasyphylla	Seq98543-20200302_4_Green2_4_3SDSE_S3_contigs_40w_500m.1
Chondria dasyphylla	Seq99999-20200302_4_Green2_4_3SDSE_S3_contigs_40w_500m.2
Chondrus crispus	Seq191-renamed-Chondrus_crispus_GCF_000350225.1_ASM35022v2_genomic.250
Chondrus crispus	Seq523-renamed-Chondrus_crispus_GCF_000350225.1_ASM35022v2_genomic.69
Chondrus crispus	Seq682-renamed-Chondrus_crispus_GCF_000350225.1_ASM35022v2_genomic.59
Chondrus crispus	Seq682-renamed-Chondrus_crispus_GCF_000350225.1_ASM35022v2_genomic.6
Chondrus crispus	Seq782-renamed-Chondrus_crispus_GCF_000350225.1_ASM35022v2_genomic.63
Chondrus crispus	Seq824-renamed-Chondrus_crispus_GCF_000350225.1_ASM35022v2_genomic.27
Chondrus crispus	Seq824-renamed-Chondrus_crispus_GCF_000350225.1_ASM35022v2_genomic.40
Chondrus crispus	Seq865-renamed-Chondrus_crispus_GCF_000350225.1_ASM35022v2_genomic.23
Erythrotrichia carnea	Seq10761-S3_FDSW210282394-1b_H722NDSX2_L1_1_contigs_50w_500min.3
Erythrotrichia carnea	Seq10761-S3_FDSW210282394-1b_H722NDSX2_L1_1_contigs_50w_500min.7
Gracilaria chorda	Seq10066-S12_FSFP210375653-1r_HMK2LDSX2_L2_1_contigs_40w_500m.2
Gracilaria chorda	Seq10185-S12_FSFP210375653-1r_HMK2LDSX2_L2_1_contigs_40w_500m.1
Gracilaria chorda	Seq2306-S12_FSFP210375653-1r_HMK2LDSX2_L2_1_contigs_40w_500m.1
Gracilaria chorda	Seq2434-S12_FSFP210375653-1r_HMK2LDSX2_L2_1_contigs_40w_500m.10
Gracilaria chorda	Seq2434-S12_FSFP210375653-1r_HMK2LDSX2_L2_1_contigs_40w_500m.7
Gracilaria chorda	Seq5008-S12_FSFP210375653-1r_HMK2LDSX2_L2_1_contigs_40w_500m.22
Gracilaria chorda	Seq5271-S12_FSFP210375653-1r_HMK2LDSX2_L2_1_contigs_40w_500m.1
Gracilaria chorda	Seq7062-S12_FSFP210375653-1r_HMK2LDSX2_L2_1_contigs_40w_500m.4
Gracilaria chorda	Seq7747-S12_FSFP210375653-1r_HMK2LDSX2_L2_1_contigs_40w_500m.1
Gracilariopsis chorda	Seq103-renamed-Gracilariopsis_chorda_GCA_003194525.1_GraCho1.0_genomic.37
Gracilariopsis chorda	Seq1111-renamed-Gracilariopsis_chorda_GCA_003194525.1_GraCho1.0_genomic.2
Gracilariopsis chorda	Seq140-renamed-Gracilariopsis_chorda_GCA_003194525.1_GraCho1.0_genomic.58
Gracilariopsis chorda	Seq140-renamed-Gracilariopsis_chorda_GCA_003194525.1_GraCho1.0_genomic.63
Gracilariopsis chorda	Seq140-renamed-Gracilariopsis_chorda_GCA_003194525.1_GraCho1.0_genomic.76
Gracilariopsis chorda	Seq328-renamed-Gracilariopsis_chorda_GCA_003194525.1_GraCho1.0_genomic.19
Gracilariopsis chorda	Seq4-renamed-Gracilariopsis_chorda_GCA_003194525.1_GraCho1.0_genomic.507
Gracilariopsis chorda	Seq49-renamed-Gracilariopsis_chorda_GCA_003194525.1_GraCho1.0_genomic.93
Gracilariopsis chorda	Seq49-renamed-Gracilariopsis_chorda_GCA_003194525.1_GraCho1.0_genomic.96
Gracilariopsis chorda	Seq7-renamed-Gracilariopsis_chorda_GCA_003194525.1_GraCho1.0_genomic.233
Gracilariopsis chorda	Seq88-renamed-Gracilariopsis_chorda_GCA_003194525.1_GraCho1.0_genomic.123
Gracilariopsis chorda	Seq92-renamed-Gracilariopsis_chorda_GCA_003194525.1_GraCho1.0_genomic.109
Gracilariopsis chorda	Seq92-renamed-Gracilariopsis_chorda_GCA_003194525.1_GraCho1.0_genomic.178
Gracilariopsis chorda	Seq92-renamed-Gracilariopsis_chorda_GCA_003194525.1_GraCho1.0_genomic.192
Hildenbrandia prototypus	Seq133-S1_FDSW210282392-1r_HTNYJDSXY_L4_1_contigs_60w_500min.36
Hildenbrandia prototypus	Seq133-S1_FDSW210282392-1r_HTNYJDSXY_L4_1_contigs_60w_500min.38
Hildenbrandia prototypus	Seq133-S1_FDSW210282392-1r_HTNYJDSXY_L4_1_contigs_60w_500min.41
Hildenbrandia prototypus	Seq1342-S1_FDSW210282392-1r_HTNYJDSXY_L4_1_contigs_60w_500min.1
Hildenbrandia prototypus	Seq143-S1_FDSW210282392-1r_HTNYJDSXY_L4_1_contigs_60w_500min.5

Hildenbrandia prototypus	Seq2533-S1_FDSW210282392-1r_HTNYJDSXY_L4_1_contigs_60w_500min.12
Hildenbrandia prototypus	Seq317-S1_FDSW210282392-1r_HTNYJDSXY_L4_1_contigs_60w_500min.35
Hildenbrandia prototypus	Seq639-S1_FDSW210282392-1r_HTNYJDSXY_L4_1_contigs_60w_500min.3
Hildenbrandia prototypus	Seq845-S1_FDSW210282392-1r_HTNYJDSXY_L4_1_contigs_60w_500min.2
Hildenbrandia prototypus	Seq892-S1_FDSW210282392-1r_HTNYJDSXY_L4_1_contigs_60w_500min.21
Hymenocladiopsis crustigena	Seq1134-renamed-19106D-06-03_S0_L001_contigs_60w_500min.1
Hymenocladiopsis crustigena	Seq1326-renamed-19106D-06-03_S0_L001_contigs_60w_500min.13
Hymenocladiopsis crustigena	Seq1326-renamed-19106D-06-03_S0_L001_contigs_60w_500min.18
Hymenocladiopsis crustigena	Seq1326-renamed-19106D-06-03_S0_L001_contigs_60w_500min.20
Hymenocladiopsis crustigena	Seq1331-renamed-19106D-06-03_S0_L001_contigs_60w_500min.21
Hymenocladiopsis crustigena	Seq1635-renamed-19106D-06-03_S0_L001_contigs_60w_500min.24
Hymenocladiopsis crustigena	Seq1635-renamed-19106D-06-03_S0_L001_contigs_60w_500min.25
Hymenocladiopsis crustigena	Seq1714-renamed-19106D-06-03_S0_L001_contigs_60w_500min.20
Hymenocladiopsis crustigena	Seq1787-renamed-19106D-06-03_S0_L001_contigs_60w_500min.36
Hymenocladiopsis crustigena	Seq1904-renamed-19106D-06-03_S0_L001_contigs_60w_500min.4
Hymenocladiopsis crustigena	Seq1915-renamed-19106D-06-03_S0_L001_contigs_60w_500min.1
Hymenocladiopsis crustigena	Seq2094-renamed-19106D-06-03_S0_L001_contigs_60w_500min.2
Hymenocladiopsis crustigena	Seq2094-renamed-19106D-06-03_S0_L001_contigs_60w_500min.5
Hymenocladiopsis crustigena	Seq3512-renamed-19106D-06-03_S0_L001_contigs_60w_500min.3
Hymenocladiopsis crustigena	Seq6965-renamed-19106D-06-03_S0_L001_contigs_60w_500min.2
Hymenocladiopsis crustigena	Seq8454-renamed-19106D-06-03_S0_L001_contigs_60w_500min.1
Hymenocladiopsis crustigena	Seq948-renamed-19106D-06-03_S0_L001_contigs_60w_500min.4
Hymenocladiopsis crustigena	Seq957-renamed-19106D-06-03_S0_L001_contigs_60w_500min.1
Hymenocladiopsis crustigena	Seq971-renamed-19106D-06-03_S0_L001_contigs_60w_500min.1
Hypnea sp.	Seq10643-renamed-19106D-07-08_S0_L001_R1_001_contigs_60w_500min.4
Hypnea sp.	Seq1136-renamed-19106D-07-08_S0_L001_R1_001_contigs_60w_500min.1
Hypnea sp.	Seq1643-renamed-19106D-07-08_S0_L001_R1_001_contigs_60w_500min.107
Hypnea sp.	Seq2179-renamed-19106D-07-08_S0_L001_R1_001_contigs_60w_500min.30
Hypnea sp.	Seq3004-renamed-19106D-07-08_S0_L001_R1_001_contigs_60w_500min.49
Hypnea sp.	Seq3276-renamed-19106D-07-08_S0_L001_R1_001_contigs_60w_500min.72
Hypnea sp.	Seq3560-renamed-19106D-07-08_S0_L001_R1_001_contigs_60w_500min.82
Hypnea sp.	Seq4085-renamed-19106D-07-08_S0_L001_R1_001_contigs_60w_500min.34
Hypnea sp.	Seq5173-renamed-19106D-07-08_S0_L001_R1_001_contigs_60w_500min.3
Hypnea sp.	Seq5195-renamed-19106D-07-08_S0_L001_R1_001_contigs_60w_500min.1
Hypnea sp.	Seq6312-renamed-19106D-07-08_S0_L001_R1_001_contigs_60w_500min.4
Hypnea sp.	Seq6942-renamed-19106D-07-08_S0_L001_R1_001_contigs_60w_500min.55
Hypoglossum anomalum	Seq10937-renamed-S4_CCAP_1344_1_CTAB_Hypoglossum_anomalum_Red_100GB_Novogene_contigs_60w_500min.7
Hypoglossum anomalum	Seq14678-renamed-S4_CCAP_1344_1_CTAB_Hypoglossum_anomalum_Red_100GB_Novogene_contigs_60w_500min.41
Hypoglossum anomalum	Seq16308-renamed-S4_CCAP_1344_1_CTAB_Hypoglossum_anomalum_Red_100GB_Novogene_contigs_60w_500min.7
Hypoglossum anomalum	Seq18504-renamed-S4_CCAP_1344_1_CTAB_Hypoglossum_anomalum_Red_100GB_Novogene_contigs_60w_500min.1
Hypoglossum anomalum	Seq25987-renamed-S4_CCAP_1344_1_CTAB_Hypoglossum_anomalum_Red_100GB_Novogene_contigs_60w_500min.9
Hypoglossum anomalum	Seq26281-renamed-S4_CCAP_1344_1_CTAB_Hypoglossum_anomalum_Red_100GB_Novogene_contigs_60w_500min.4
Hypoglossum anomalum	Seq26556-renamed-S4_CCAP_1344_1_CTAB_Hypoglossum_anomalum_Red_100GB_Novogene_contigs_60w_500min.1
Hypoglossum anomalum	Seq28529-renamed-S4_CCAP_1344_1_CTAB_Hypoglossum_anomalum_Red_100GB_Novogene_contigs_60w_500min.10
Hypoglossum anomalum	Seq28976-renamed-S4_CCAP_1344_1_CTAB_Hypoglossum_anomalum_Red_100GB_Novogene_contigs_60w_500min.7
Hypoglossum anomalum	Seq31888-renamed-S4_CCAP_1344_1_CTAB_Hypoglossum_anomalum_Red_100GB_Novogene_contigs_60w_500min.8
Hypoglossum anomalum	Seq37325-renamed-S4_CCAP_1344_1_CTAB_Hypoglossum_anomalum_Red_100GB_Novogene_contigs_60w_500min.1
Hypoglossum anomalum	Seq45272-renamed-S4_CCAP_1344_1_CTAB_Hypoglossum_anomalum_Red_100GB_Novogene_contigs_60w_500min.4
Hypoglossum anomalum	Seq45838-renamed-S4_CCAP_1344_1_CTAB_Hypoglossum_anomalum_Red_100GB_Novogene_contigs_60w_500min.21
Hypoglossum anomalum	Seq48889-renamed-S4_CCAP_1344_1_CTAB_Hypoglossum_anomalum_Red_100GB_Novogene_contigs_60w_500min.1
Hypoglossum anomalum	Seq51433-renamed-S4_CCAP_1344_1_CTAB_Hypoglossum_anomalum_Red_100GB_Novogene_contigs_60w_500min.1

Hypoglossum anomalum	Seq518-renamed-S4_CCAP_1344_1_CTAB_Hypoglossum_anomalum_Red_100GB_Novogene_contigs_60w_500min.5
Hypoglossum anomalum	Seq65540-renamed-S4_CCAP_1344_1_CTAB_Hypoglossum_anomalum_Red_100GB_Novogene_contigs_60w_500min.2
Hypoglossum anomalum	Seq6756-renamed-S4_CCAP_1344_1_CTAB_Hypoglossum_anomalum_Red_100GB_Novogene_contigs_60w_500min.3
Hypoglossum anomalum	Seq7924-renamed-S4_CCAP_1344_1_CTAB_Hypoglossum_anomalum_Red_100GB_Novogene_contigs_60w_500min.26
Hypoglossum anomalum	Seq84164-renamed-S4_CCAP_1344_1_CTAB_Hypoglossum_anomalum_Red_100GB_Novogene_contigs_60w_500min.1
Hypoglossum anomalum	Seq91111-renamed-S4_CCAP_1344_1_CTAB_Hypoglossum_anomalum_Red_100GB_Novogene_contigs_60w_500min.4
Hypoglossum anomalum	Seq99687-renamed-S4_CCAP_1344_1_CTAB_Hypoglossum_anomalum_Red_100GB_Novogene_contigs_60w_500min.1
Jania rubens	Seq13026-seaweed_CCAP1399_1_Dneasy_Jania_rubens_Red_40GB_Genohub_contigs_60w_500m.1
Jania rubens	Seq3670-seaweed_CCAP1399_1_Dneasy_Jania_rubens_Red_40GB_Genohub_contigs_60w_500m.2
Jania rubens	Seq4587-seaweed_CCAP1399_1_Dneasy_Jania_rubens_Red_40GB_Genohub_contigs_60w_500m.2
Jania rubens	Seq5781-seaweed_CCAP1399_1_Dneasy_Jania_rubens_Red_40GB_Genohub_contigs_60w_500m.2
Jania rubens	Seq5789-seaweed_CCAP1399_1_Dneasy_Jania_rubens_Red_40GB_Genohub_contigs_60w_500m.1
Jania rubens	Seq6414-seaweed_CCAP1399_1_Dneasy_Jania_rubens_Red_40GB_Genohub_contigs_60w_500m.1
Jania rubens	Seq664-seaweed_CCAP1399_1_Dneasy_Jania_rubens_Red_40GB_Genohub_contigs_60w_500m.35
Kylinia rosulata	Seq3800-S1_FSFP210375642-1r_HMK2LDSX2_L3_1_contigs_60w_500m.3
Laingia sp.	Seq10398-renamed-3_CCMP466_Dneasy_Laingia_sp_Red_40GB_Genohub_contigs_40w_500min.1
Laingia sp.	Seq10398-renamed-3_CCMP466_Dneasy_Laingia_sp_Red_40GB_Genohub_contigs_40w_500min.5
Laingia sp.	Seq1049-renamed-3_CCMP466_Dneasy_Laingia_sp_Red_40GB_Genohub_contigs_40w_500min.1
Laingia sp.	Seq1080-renamed-3_CCMP466_Dneasy_Laingia_sp_Red_40GB_Genohub_contigs_40w_500min.2
Laingia sp.	Seq11458-renamed-3_CCMP466_Dneasy_Laingia_sp_Red_40GB_Genohub_contigs_40w_500min.3
Laingia sp.	Seq17243-renamed-3_CCMP466_Dneasy_Laingia_sp_Red_40GB_Genohub_contigs_40w_500min.1
Laingia sp.	Seq1966-renamed-3_CCMP466_Dneasy_Laingia_sp_Red_40GB_Genohub_contigs_40w_500min.4
Laingia sp.	Seq1966-renamed-3_CCMP466_Dneasy_Laingia_sp_Red_40GB_Genohub_contigs_40w_500min.5
Laingia sp.	Seq1967-renamed-3_CCMP466_Dneasy_Laingia_sp_Red_40GB_Genohub_contigs_40w_500min.9
Laingia sp.	Seq3942-renamed-3_CCMP466_Dneasy_Laingia_sp_Red_40GB_Genohub_contigs_40w_500min.3
Laingia sp.	Seq4297-renamed-3_CCMP466_Dneasy_Laingia_sp_Red_40GB_Genohub_contigs_40w_500min.3
Laingia sp.	Seq4412-renamed-3_CCMP466_Dneasy_Laingia_sp_Red_40GB_Genohub_contigs_40w_500min.9
Laingia sp.	Seq4728-renamed-3_CCMP466_Dneasy_Laingia_sp_Red_40GB_Genohub_contigs_40w_500min.4
Laingia sp.	Seq4786-renamed-3_CCMP466_Dneasy_Laingia_sp_Red_40GB_Genohub_contigs_40w_500min.3
Laingia sp.	Seq5275-renamed-3_CCMP466_Dneasy_Laingia_sp_Red_40GB_Genohub_contigs_40w_500min.1
Laingia sp.	Seq8144-renamed-3_CCMP466_Dneasy_Laingia_sp_Red_40GB_Genohub_contigs_40w_500min.11
Laingia sp.	Seq8144-renamed-3_CCMP466_Dneasy_Laingia_sp_Red_40GB_Genohub_contigs_40w_500min.19
Laingia sp.	Seq82-renamed-3_CCMP466_Dneasy_Laingia_sp_Red_40GB_Genohub_contigs_40w_500min.9
Laingia sp.	Seq8586-renamed-3_CCMP466_Dneasy_Laingia_sp_Red_40GB_Genohub_contigs_40w_500min.1
Laminariocolax aecidioides	Seq2145-S22_FSFP210375663-1r_HLF3FDSX2_L4_1_60w_500m.18
Laminariocolax aecidioides	Seq2145-S22_FSFP210375663-1r_HLF3FDSX2_L4_1_60w_500m.9
Laminariocolax aecidioides	Seq21675-S22_FSFP210375663-1r_HLF3FDSX2_L4_1_60w_500m.1
Laminariocolax aecidioides	Seq22644-S22_FSFP210375663-1r_HLF3FDSX2_L4_1_60w_500m.1
Laminariocolax aecidioides	Seq27988-S22_FSFP210375663-1r_HLF3FDSX2_L4_1_60w_500m.1
Laminariocolax aecidioides	Seq37844-S22_FSFP210375663-1r_HLF3FDSX2_L4_1_60w_500m.1
Laminariocolax aecidioides	Seq738-S22_FSFP210375663-1r_HLF3FDSX2_L4_1_60w_500m.5
Laminariocolax aecidioides	Seq7815-S22_FSFP210375663-1r_HLF3FDSX2_L4_1_60w_500m.5
Laminariocolax aecidioides	Seq9578-S22_FSFP210375663-1r_HLF3FDSX2_L4_1_60w_500m.2
Myriogramme manginii	Seq1076-renamed-19106D-08-08_S8_L003_contigs_60w_500m.11
Myriogramme manginii	Seq19138-renamed-19106D-08-08_S8_L003_contigs_60w_500m.70
Myriogramme manginii	Seq70138-renamed-19106D-08-08_S8_L003_contigs_60w_500m.1
Myriogramme manginii	Seq7456-renamed-19106D-08-08_S8_L003_contigs_60w_500m.11
Myriogramme manginii	Seq7456-renamed-19106D-08-08_S8_L003_contigs_60w_500m.15
Myriogramme manginii	Seq7456-renamed-19106D-08-08_S8_L003_contigs_60w_500m.5
Nemalionopsis parkeri	Seq1102-S6_FDSW210282397-1r_HTNYJDSXY_L4_1_contigs_60w_500min.1
Nemalionopsis parkeri	Seq2887-S6_FDSW210282397-1r_HTNYJDSXY_L4_1_contigs_60w_500min.1
Palmaria decipiens	Seq37942-renamed-19106D-06-02_S0_L001_contigs_60w_500min.1
Palmaria decipiens	Seq5953-renamed-19106D-06-02_S0_L001_contigs_60w_500min.1
Palmaria decipiens	Seq62634-renamed-19106D-06-02_S0_L001_contigs_60w_500min.3
Phyllophora antarctica	Seq170100-4_CCMP1276_Dneasy_Phyllophora_antarctica_Red_40GB_Genohub_contigs_40w_500m.3
Phyllophora antarctica	Seq19908-4_CCMP1276_Dneasy_Phyllophora_antarctica_Red_40GB_Genohub_contigs_40w_500m.1
Phyllophora antarctica	Seq282815-4_CCMP1276_Dneasy_Phyllophora_antarctica_Red_40GB_Genohub_contigs_40w_500m.1
Phyllophora antarctica	Seq56532-4_CCMP1276_Dneasy_Phyllophora_antarctica_Red_40GB_Genohub_contigs_40w_500m.1
Polyneura hilliae	Seq11135-renamed-S14_FDSW210282405-2r_H5L2YDSX2_L2_1_contigs_40w_500m.1
Polyneura hilliae	Seq11136-renamed-S14_FDSW210282405-2r_H5L2YDSX2_L2_1_contigs_40w_500m.1

Polyneura hilliae	Seq127029-renamed-S14_FDSW210282405-2r_H5L2YDSX2_L2_1_contigs_40w_500m.4
Polyneura hilliae	Seq128189-renamed-S14_FDSW210282405-2r_H5L2YDSX2_L2_1_contigs_40w_500m.2
Polyneura hilliae	Seq128190-renamed-S14_FDSW210282405-2r_H5L2YDSX2_L2_1_contigs_40w_500m.2
Polyneura hilliae	Seq132792-renamed-S14_FDSW210282405-2r_H5L2YDSX2_L2_1_contigs_40w_500m.1
Polyneura hilliae	Seq156486-renamed-S14_FDSW210282405-2r_H5L2YDSX2_L2_1_contigs_40w_500m.1
Polyneura hilliae	Seq37987-renamed-S14_FDSW210282405-2r_H5L2YDSX2_L2_1_contigs_40w_500m.4
Polyneura hilliae	Seq37987-renamed-S14_FDSW210282405-2r_H5L2YDSX2_L2_1_contigs_40w_500m.5
Polyneura hilliae	Seq71059-renamed-S14_FDSW210282405-2r_H5L2YDSX2_L2_1_contigs_40w_500m.1
Polyneura hilliae	Seq8603-renamed-S14_FDSW210282405-2r_H5L2YDSX2_L2_1_contigs_40w_500m.1
Polyneura hilliae	Seq89179-renamed-S14_FDSW210282405-2r_H5L2YDSX2_L2_1_contigs_40w_500m.1
Porphyra lucasii	Seq13657-renamed-19106D-07-01_S0_L001_R1_001_contigs_60w_500min.1
Porphyra lucasii	Seq148523-renamed-19106D-07-01_S0_L001_R1_001_contigs_60w_500min.1
Porphyra lucasii	Seq157799-renamed-19106D-07-01_S0_L001_R1_001_contigs_60w_500min.1
Porphyra lucasii	Seq3645-renamed-19106D-07-01_S0_L001_R1_001_contigs_60w_500min.28
Porphyra lucasii	Seq53773-renamed-19106D-07-01_S0_L001_R1_001_contigs_60w_500min.1
Porphyra lucasii	Seq6639-renamed-19106D-07-01_S0_L001_R1_001_contigs_60w_500min.3
Pugetia sp.	Seq1076-renamed-5_CCMP719_Dneasy_Pugetia_sp_Red_40GB_Genohub_contigs_60w_500min.2
Pugetia sp.	Seq1093-renamed-5_CCMP719_Dneasy_Pugetia_sp_Red_40GB_Genohub_contigs_60w_500min.7
Pugetia sp.	Seq1735-renamed-5_CCMP719_Dneasy_Pugetia_sp_Red_40GB_Genohub_contigs_60w_500min.12
Pugetia sp.	Seq1735-renamed-5_CCMP719_Dneasy_Pugetia_sp_Red_40GB_Genohub_contigs_60w_500min.15
Pugetia sp.	Seq2626-renamed-5_CCMP719_Dneasy_Pugetia_sp_Red_40GB_Genohub_contigs_60w_500min.2
Pugetia sp.	Seq273-renamed-5_CCMP719_Dneasy_Pugetia_sp_Red_40GB_Genohub_contigs_60w_500min.8
Pugetia sp.	Seq365-renamed-5_CCMP719_Dneasy_Pugetia_sp_Red_40GB_Genohub_contigs_60w_500min.14
Pugetia sp.	Seq3724-renamed-5_CCMP719_Dneasy_Pugetia_sp_Red_40GB_Genohub_contigs_60w_500min.2
Pugetia sp.	Seq3753-renamed-5_CCMP719_Dneasy_Pugetia_sp_Red_40GB_Genohub_contigs_60w_500min.1
Pugetia sp.	Seq4567-renamed-5_CCMP719_Dneasy_Pugetia_sp_Red_40GB_Genohub_contigs_60w_500min.3
Pugetia sp.	Seq4841-renamed-5_CCMP719_Dneasy_Pugetia_sp_Red_40GB_Genohub_contigs_60w_500min.13
Pugetia sp.	Seq5795-renamed-5_CCMP719_Dneasy_Pugetia_sp_Red_40GB_Genohub_contigs_60w_500min.6
Pugetia sp.	Seq7394-renamed-5_CCMP719_Dneasy_Pugetia_sp_Red_40GB_Genohub_contigs_60w_500min.2
Pugetia sp.	Seq850-renamed-5_CCMP719_Dneasy_Pugetia_sp_Red_40GB_Genohub_contigs_60w_500min.22
Pugetia sp.	Seq9052-renamed-5_CCMP719_Dneasy_Pugetia_sp_Red_40GB_Genohub_contigs_60w_500min.1
Pyropia yezoensis	Seq0-renamed-Pyropia_yezoensis_GCA_009829735.1_ASM982973v1_genomic.11209
Pyropia yezoensis	Seq0-renamed-Pyropia_yezoensis_GCA_009829735.1_ASM982973v1_genomic.18856
Pyropia yezoensis	Seq1-renamed-Pyropia_yezoensis_GCA_009829735.1_ASM982973v1_genomic.1241
Pyropia yezoensis	Seq1-renamed-Pyropia_yezoensis_GCA_009829735.1_ASM982973v1_genomic.26727
Pyropia yezoensis	Seq1-renamed-Pyropia_yezoensis_GCA_009829735.1_ASM982973v1_genomic.3923
Pyropia yezoensis	Seq2-renamed-Pyropia_yezoensis_GCA_009829735.1_ASM982973v1_genomic.18221
Rhodachlya madagascarensis	Seq125391-<br>S3_CCAP1389_1_DNeasy_Rhodachlya_madagascarensis_Red_40GB_Novogene_contigs_60w_500m.1
Rhodachlya madagascarensis	Seq19673-S3_CCAP1389_1_DNeasy_Rhodachlya_madagascarensis_Red_40GB_Novogene_contigs_60w_500m.1
Rhodachlya madagascarensis	Seq48804-S3_CCAP1389_1_DNeasy_Rhodachlya_madagascarensis_Red_40GB_Novogene_contigs_60w_500m.1
Rhodachlya madagascarensis	Seq55800-S3_CCAP1389_1_DNeasy_Rhodachlya_madagascarensis_Red_40GB_Novogene_contigs_60w_500m.1
Rhodachlya madagascarensis	Seq71125-S3_CCAP1389_1_DNeasy_Rhodachlya_madagascarensis_Red_40GB_Novogene_contigs_60w_500m.1
Rhodachlya madagascarensis	Seq7425-S3_CCAP1389_1_DNeasy_Rhodachlya_madagascarensis_Red_40GB_Novogene_contigs_60w_500m.1
Rhodachlya madagascarensis	Seq85769-S3_CCAP1389_1_DNeasy_Rhodachlya_madagascarensis_Red_40GB_Novogene_contigs_60w_500m.1
Rhodenigma contortum	Seq4372-renamed-19106D-07-11_S0_L001_R1_001_contigs_60w_500min.2
Rhodochorton purpureum	Seq63903-renamed-19106D-08-09_S0_L001_contigs_40w_500m.1
Rhodothamniella floridula	Seq33762-S20_FSFP210375661-1r_HLGVHDSX2_L1_1_40w_500m.1
Rhodothamniella floridula	Seq3910-S20_FSFP210375661-1r_HLGVHDSX2_L1_1_40w_500m.4
Rhodothamniella floridula	Seq67149-S20_FSFP210375661-1r_HLGVHDSX2_L1_1_40w_500m.1
Rhodothamniella floridula	Seq77802-S20_FSFP210375661-1r_HLGVHDSX2_L1_1_40w_500m.1
Vertebrata fucoides	Seq19423-renamed-S15_FDSW210282406-1r_HTTF7DSXY_L1_1_contigs_50w_500m.2
Vertebrata fucoides	Seq23869-renamed-S15_FDSW210282406-1r_HTTF7DSXY_L1_1_contigs_50w_500m.1
Vertebrata fucoides	Seq23916-renamed-S15_FDSW210282406-1r_HTTF7DSXY_L1_1_contigs_50w_500m.1
Vertebrata fucoides	Seq28339-renamed-S15_FDSW210282406-1r_HTTF7DSXY_L1_1_contigs_50w_500m.1
Vertebrata fucoides	Seq32191-renamed-S15_FDSW210282406-1r_HTTF7DSXY_L1_1_contigs_50w_500m.2
Vertebrata fucoides	Seq37672-renamed-S15_FDSW210282406-1r_HTTF7DSXY_L1_1_contigs_50w_500m.7
Vertebrata fucoides	Seq3796-renamed-S15_FDSW210282406-1r_HTTF7DSXY_L1_1_contigs_50w_500m.5
Vertebrata fucoides	Seq55418-renamed-S15_FDSW210282406-1r_HTTF7DSXY_L1_1_contigs_50w_500m.2
Vertebrata fucoides	Seq72724-renamed-S15_FDSW210282406-1r_HTTF7DSXY_L1_1_contigs_50w_500m.1
Vertebrata fucoides	Seq8377-renamed-S15_FDSW210282406-1r_HTTF7DSXY_L1_1_contigs_50w_500m.15
