# Supplementary material for: Vanadium-Dependent Haloperoxidase Gene Evolution in Brown Algae: Evidence for Horizontal Gene Transfer
Source: Int J Mol Sci. 2025 Jan 16;26(2):716. doi: 10.3390/ijms26020716 (PMC11765636; doi:10.3390/ijms26020716)
Supplement: Supplementary file 1 [file ijms-26-00716-s001.zip › Supplemental Table S3. The V-HPO clusters in brown and red algae.pdf]

Supplemental Table S3. The V-HPO clusters in brown and red algae.

	Algae Species	Chromosome/Scaffold	Copy Number
Brown Algae	*Agarum clathratum*	Seq11480	2
		Seq2043	2
		Seq32263	2
	Alethocladus corymbosus	Seq146720	2
	Botrytella uvaeformis	Seq30773	2
	Carpomitra costata	Seq27075	2
	Desmarestia aculeata	Seq17725	2
		Seq21836	2
		Seq30144	2
	Desmarestia anceps	Seq6537	3
		Seq37906	2
	Himantothallus grandifolius	Seq10010	2
	Laminaria digitata	Seq12916	2
		Seq21802	2
		Seq49288	2
	Laminariocolax aecidoides	Seq1546	2
		Seq7672	2
	Laminarionema elsbetiae	Seq24411	2
	Macrocystis pyrifera	Seq19629	3
		Seq10306	2
		Seq15064	2
		Seq24140	2
		Seq5274	2
		Seq5907	2
		Seq9206	2
	Polycladia myrica	Seq4333	2
	Saccharina japonica	LG03	58
		LG04	13
		LG13	2
		LG21	9
		LG23	1
		LG28	5
	Saccharina latissima	Seq17775	2

		Seq26351	2
		Seq28011	2
		Seq3357	2
		Seq50163	2
		Seq8765	2
	Sargassum angustifolium	Seq25181	2
		Seq25182	2
		Seq37706	2
		Seq53092	2
	Sargassum latifolium	Seq18221	2
		Seq25958	2
		Seq27627	2
	Scytosiphon promiscuus	Seq5027	2
	Scytothamnus fasciculatus	Seq498	3
Red Algae	*Chondria dasyphylla*	Seq48051	2
	Chondrus crispus	Seq682	2
		Seq824	2
	Erythrotrichia carnea	Seq10761	2
	Gracilaria chorda	Seq2434	2
	Gracilariopsis chorda	Seq140	3
		Seq92	3
		Seq49	2
	Hildenbrandia prototypus	Seq133	3
	Hymenocladiopsis crustigena	Seq1326	3
		Seq1635	2
		Seq2094	2
	Laingia sp.	Seq10398	2
		Seq1966	2
		Seq8144	2
	Laminariocolax aecidioides	Seq2145	2
	Myriogramme manginii	Seq7456	3
	Polyneura hilliae	Seq37987	2
	Pugetia sp.	Seq1735	2
	Pyropia yezoensis	Seq1	3
		Seq0	2
