# Supplementary material for: Vanadium-Dependent Haloperoxidase Gene Evolution in Brown Algae: Evidence for Horizontal Gene Transfer
Source: Int J Mol Sci. 2025 Jan 16;26(2):716. doi: 10.3390/ijms26020716 (PMC11765636; doi:10.3390/ijms26020716)
Supplement: Supplementary file 1 [file ijms-26-00716-s001.zip › Supplemental Table S4. The BLAST hit of Thermoanaerobaculum aquaticum proteins against Saccharina japonica genome..pdf]

Supplemental Table S4. The BLAST hit of *Thermoanaerobaculum aquaticum* proteins against *Saccharina japonica* genome.

Thermoanaerobaculum aquaticum protein accession	Hit on Saccharina japonica	pident	evalue	bitscore	qcovs
WP_053335200.1_F0F1_ATP_synthase_subunit_A_[Thermoanaerobaculum_aquaticum]	ptg001624l	32.787	2.91E-18	87.4	70
WP_053334697.1_lipoyl_protein_ligase_domain-containing_protein_[Thermoanaerobaculum_aquaticum]	ptg002738l	33.516	9.23E-15	76.3	82
WP_038049880.1_UDP-3-O-acyl-N-acetylglucosamine_deacetylase_[Thermoanaerobaculum_aquaticum]	ptg004510l	40.511	1.33E-54	194	87
WP_038047577.1_protein_disulfide_oxidoreductase_[Thermoanaerobaculum_aquaticum]	ptg004735l	26.19	3.36E-12	68.6	59
WP_038046336.1_F0F1_ATP_synthase_subunit_beta_[Thermoanaerobaculum_aquaticum]	ptg001921l	68.421	0	581	95
WP_038046334.1_ATP_synthase_F1_subunit_gamma_[Thermoanaerobaculum_aquaticum]	ptg000372l	39.322	5.42E-58	203	99
WP_038046332.1_F0F1_ATP_synthase_subunit_alpha_[Thermoanaerobaculum_aquaticum]	ptg001624l	61.616	0	639	97
WP_081799864.1_peptidylprolyl_isomerase_[Thermoanaerobaculum_aquaticum]	ptg005527l	22.143	0.000000151	56.6	65
WP_038050171.1_peptidylprolyl_isomerase_[Thermoanaerobaculum_aquaticum]	LG28	41.27	2.43E-08	59.7	19
WP_235208716.1_glycosyltransferase,_partial_[Thermoanaerobaculum_aquaticum]	ptg001871l	29.612	2.22E-10	63.2	96
WP_152543920.1_glycosyltransferase_[Thermoanaerobaculum_aquaticum]	ptg002316l	30.108	9.37E-29	122	95
WP_053335149.1_glycosyltransferase_[Thermoanaerobaculum_aquaticum]	ptg003112l	32.258	6E-11	67.8	42
WP_038049024.1_glycosyltransferase_[Thermoanaerobaculum_aquaticum]	ptg002534l	27.148	9.19E-11	67.4	75
WP_038046667.1_cytochrome_c_biogenesis_protein_CcsA_[Thermoanaerobaculum_aquaticum]	ptg001025l	31.469	0.00000398	50.8	63
WP_038046403.1_1-acyl-sn-glycerol-3-phosphate_acyltransferase_[Thermoanaerobaculum_aquaticum]	ptg005535l	27.151	5.06E-25	112	78
WP_200867129.1_M2_family_metallopeptidase_[Thermoanaerobaculum_aquaticum]	ptg003548l	23.704	0.00000512	53.5	45
WP_161685349.1_tetratricopeptide_repeat_protein_[Thermoanaerobaculum_aquaticum]	ptg004982l	40	6.01E-17	82.8	25
WP_053334798.1_helix-turn-helix_domain-containing_protein_[Thermoanaerobaculum_aquaticum]	ptg002176l	30.303	1.31E-08	54.7	84
WP_038049815.1_alkaline_phosphatase_family_protein_[Thermoanaerobaculum_aquaticum]	ptg000619l	31.077	5.89E-35	148	40
WP_038048159.1_biotin/lipoyl-containing_protein_[Thermoanaerobaculum_aquaticum]	ptg002223l	40.845	2.19E-08	55.8	43
WP_038048155.1_alkaline_phosphatase_family_protein_[Thermoanaerobaculum_aquaticum]	ptg000619l	32.282	3.37E-40	164	52
WP_038046133.1_zinc-binding_dehydrogenase_[Thermoanaerobaculum_aquaticum]	ptg004026l	33.533	1.75E-41	158	95
WP_200867140.1_orotidine_5'-phosphate_decarboxylase_/_HUMPS_family_protein,_partial_[Thermoanaerobaculum_aquaticum]	ptg001025l	71.053	0.000000271	48.9	57
WP_152543834.1_glutamate_synthase-related_protein_[Thermoanaerobaculum_aquaticum]	ptg005053l	35.22	4E-40	166	32
WP_081799994.1_transketolase_C-terminal_domain-containing_protein_[Thermoanaerobaculum_aquaticum]	ptg002867l	25.281	3.68E-09	62.8	79
WP_053335244.1_CBS_domain-containing_protein_[Thermoanaerobaculum_aquaticum]	ptg004623l	32.787	1.77E-14	82.4	25
WP_053335194.1_M28_family_peptidase_[Thermoanaerobaculum_aquaticum]	ptg001897l	45.714	2.91E-08	60.5	15
WP_053335156.1_phosphotransferase_[Thermoanaerobaculum_aquaticum]	ptg004480l	29.293	1.17E-16	84.3	86
WP_053335048.1_transketolase_C-terminal_domain-containing_protein_[Thermoanaerobaculum_aquaticum]	ptg002612l	30.382	1.57E-67	245	93

WP_053334787.1_CHASE2_domain-containing_protein_[Thermoanaerobaculum_aquaticum]	ptg003903l	31.734	5.11E-21	102	42
WP_053334781.1_arginine--tRNA_ligase_[Thermoanaerobaculum_aquaticum]	ptg005820l	32.479	2.35E-08	62	15
WP_038048487.1_DnaJ_C-terminal_domain-containing_protein_[Thermoanaerobaculum_aquaticum]	ptg004942l	31.755	5.53E-36	141	95
WP_038047759.1_thioredoxin_fold_domain-containing_protein_[Thermoanaerobaculum_aquaticum]	ptg002760l	33.628	0.000000209	53.1	64
WP_038047149.1_NapC/NirT_family_cytochrome_c_[Thermoanaerobaculum_aquaticum]	ptg002214l	22.928	5.44E-15	81.6	59
WP_038046684.1_thioredoxin_fold_domain-containing_protein_[Thermoanaerobaculum_aquaticum]	ptg003334l	35.577	2.76E-11	63.5	71
WP_161685566.1_glycosyltransferase_[Thermoanaerobaculum_aquaticum]	ptg003768l	27.459	1.65E-12	75.5	29
WP_053335138.1_glycosyltransferase_[Thermoanaerobaculum_aquaticum]	ptg004058l	33.613	0.000000013	62	22
WP_053335137.1_glycosyltransferase_[Thermoanaerobaculum_aquaticum]	ptg004085l	31.452	0.000000144	56.6	38
WP_053335192.1_glycoside_hydrolase_family_3_N-terminal_domain-containing_protein_[Thermoanaerobaculum_aquaticum]	ptg005474l	30.745	1.59E-29	123	92
WP_053334965.1_ATP-binding_cassette_domain-containing_protein_[Thermoanaerobaculum_aquaticum]	ptg005227l	33.588	1.01E-33	134	93
WP_038050585.1_ATP-binding_cassette_domain-containing_protein,_partial_[Thermoanaerobaculum_aquaticum]	ptg005526l	33.742	1.25E-19	89.7	80
WP_038050202.1_ATP-binding_cassette_domain-containing_protein_[Thermoanaerobaculum_aquaticum]	ptg001353l	49.541	6.78E-65	225	68
WP_038048389.1_MFS_transporter_[Thermoanaerobaculum_aquaticum]	ptg002429l	30.688	8.23E-16	83.6	43
WP_038047119.1_FHA_domain-containing_protein_[Thermoanaerobaculum_aquaticum]	ptg004476l	26.502	9.89E-12	70.1	81
WP_038046405.1_trypsin-like_peptidase_domain-containing_protein_[Thermoanaerobaculum_aquaticum]	ptg004479l	32.668	2E-53	196	86
WP_161685588.1_IPT/TIG_domain-containing_protein_[Thermoanaerobaculum_aquaticum]	LG01	29.795	6.88E-11	67	92
WP_152543996.1_HD_domain-containing_phosphohydrolase_[Thermoanaerobaculum_aquaticum]	ptg005301l	31.016	3.54E-19	95.5	36
WP_081800069.1_ATP-binding_protein_[Thermoanaerobaculum_aquaticum]	ptg002909l	30.35	2.64E-14	81.3	33
WP_053335162.1_HD_domain-containing_phosphohydrolase_[Thermoanaerobaculum_aquaticum]	ptg003829l	34.146	3.93E-21	101	33
WP_053335110.1_ATP-binding_protein_[Thermoanaerobaculum_aquaticum]	ptg005484l	29.135	3.82E-40	165	59
WP_053334880.1_ATP-binding_protein_[Thermoanaerobaculum_aquaticum]	ptg005484l	33.929	3.35E-37	155	42
WP_053334788.1_M20/M25/M40_family_metallo-hydrolase_[Thermoanaerobaculum_aquaticum]	ptg002658l	34.429	6.42E-69	246	94
WP_038050177.1_NADH-quinone_oxidoreductase_subunit_C_[Thermoanaerobaculum_aquaticum]	ptg004143l	24.74	7.01E-15	81.6	71
WP_038050093.1_HD_domain-containing_phosphohydrolase_[Thermoanaerobaculum_aquaticum]	ptg005035l	31.933	1.5E-49	181	94
WP_038048832.1_M20/M25/M40_family_metallo-hydrolase_[Thermoanaerobaculum_aquaticum]	ptg001897l	38.889	0.000000397	57	13
WP_038048611.1_aminotransferase_class_IV_[Thermoanaerobaculum_aquaticum]	ptg005680l	26.596	3.6E-19	91.7	91
WP_038048035.1_thiamine_pyrophosphate-dependent_enzyme_[Thermoanaerobaculum_aquaticum]	ptg002612l	35.235	3.41E-113	380	99
WP_038046376.1_NTP_transferase_domain-containing_protein_[Thermoanaerobaculum_aquaticum]	ptg004075l	34.796	2.17E-49	180	99
WP_152543982.1_ATP-binding_protein_[Thermoanaerobaculum_aquaticum]	ptg003336l	35.18	2.04E-48	191	40
WP_152543954.1_aminotransferase_class_I/II-fold_pyridoxal_phosphate-dependent_enzyme_[Thermoanaerobaculum_aquaticum]	ptg001224l	35.714	8.81E-68	236	94
WP_152543906.1_ATP-binding_protein_[Thermoanaerobaculum_aquaticum]	ptg004494l	32.766	5.42E-20	98.6	41

WP_053335264.1_aminotransferase_class_I/II-fold_pyridoxal_phosphate-dependent_enzyme_[Thermoanaerobaculum_aquaticum]	ptg005435l	26.73	2.17E-16	84.3	88
WP_038049817.1_adenylate/guanylate_cyclase_domain-containing_protein_[Thermoanaerobaculum_aquaticum]	ptg004476l	30.64	3.03E-24	111	53
WP_038049764.1_aminotransferase_class_I/II-fold_pyridoxal_phosphate-dependent_enzyme_[Thermoanaerobaculum_aquaticum]	ptg003299l	30.457	8.06E-33	141	50
WP_038048571.1_LptF/LptG_family_permease_[Thermoanaerobaculum_aquaticum]	ptg001925l	25.862	0.00000949	53.5	22
WP_038048232.1_VIT_domain-containing_protein_[Thermoanaerobaculum_aquaticum]	ptg004987l	21.581	5.29E-10	67.4	54
WP_038048121.1_aminotransferase_class_III-fold_pyridoxal_phosphate-dependent_enzyme_[Thermoanaerobaculum_aquaticum]	ptg005487l	29.508	1.79E-24	68.2	59
WP_038047232.1_SpoIIE_family_protein_phosphatase_[Thermoanaerobaculum_aquaticum]	ptg004476l	25.185	3.59E-08	61.6	32
WP_038046756.1_EF-Tu/IF-2/RF-3_family_GTPase,_partial_[Thermoanaerobaculum_aquaticum]	ptg002214l	68.273	5.32E-112	357	99
WP_038046201.1_aminotransferase_class_I/II-fold_pyridoxal_phosphate-dependent_enzyme_[Thermoanaerobaculum_aquaticum]	ptg005614l	36.364	1.64E-63	224	85
WP_161685276.1_DEAD/DEAH_box_helicase,_partial_[Thermoanaerobaculum_aquaticum]	ptg003903l	43.094	3.43E-27	117	44
WP_038046268.1_ABC_transporter_substrate-binding_protein_[Thermoanaerobaculum_aquaticum]	ptg003131l	27.103	4.1E-10	66.6	52
WP_161685670.1_response_regulator,_partial_[Thermoanaerobaculum_aquaticum]	ptg005150l	35.514	0.000000394	50.1	97
WP_161685523.1_ABC_transporter_substrate-binding_protein_[Thermoanaerobaculum_aquaticum]	ptg005227l	24.887	1.53E-21	102	85
WP_161685457.1_carbonic_anhydrase_[Thermoanaerobaculum_aquaticum]	ptg003910l	30.208	1.23E-23	101	94
WP_053335236.1_glucokinase_[Thermoanaerobaculum_aquaticum]	ptg001925l	26.91	1.12E-22	102	89
WP_053335207.1_protein_kinase_[Thermoanaerobaculum_aquaticum]	ptg004928l	36.054	4.81E-37	149	63
WP_053334945.1_LysM_peptidoglycan-binding_domain-containing_protein_[Thermoanaerobaculum_aquaticum]	ptg005398l	39.815	1.7E-17	90.5	20
WP_053334865.1_prolyl_oligopeptidase_family_serine_peptidase_[Thermoanaerobaculum_aquaticum]	ptg004136l	26.45	2.53E-25	117	46
WP_038048769.1_aldehyde_dehydrogenase_family_protein_[Thermoanaerobaculum_aquaticum]	ptg003910l	47.031	8.29E-110	361	88
WP_038046439.1_TraR/DksA_C4-type_zinc_finger_protein_[Thermoanaerobaculum_aquaticum]	ptg001658l	37.313	1.32E-09	57.8	57
WP_161685518.1_DNA_translocase_FtsK_4TM_domain-containing_protein_[Thermoanaerobaculum_aquaticum]	ptg004538l	48.017	6.32E-121	405	58
WP_081800132.1_c-type_cytochrome_domain-containing_protein_[Thermoanaerobaculum_aquaticum]	ptg004179l	30.631	0.000000198	52	84
WP_081799885.1_response_regulator_[Thermoanaerobaculum_aquaticum]	ptg005484l	29.496	2.7E-09	65.5	15
WP_081799775.1_response_regulator_[Thermoanaerobaculum_aquaticum]	ptg003734l	32.456	2.59E-54	205	66
WP_053334890.1_response_regulator_[Thermoanaerobaculum_aquaticum]	ptg004158l	27.551	1.64E-33	101	41
WP_053334769.1_thiamine_pyrophosphate-dependent_enzyme_[Thermoanaerobaculum_aquaticum]	ptg002867l	31.579	3.73E-15	81.6	45
WP_038050334.1_response_regulator_[Thermoanaerobaculum_aquaticum]	ptg001025l	24.538	1.85E-21	65.9	59
WP_038050266.1_CRISPR-associated_ring_nuclease_[Thermoanaerobaculum_aquaticum]	ptg005754l	23.602	1.65E-08	62	50
WP_038049904.1_Swt1_family_HEPN_domain-containing_protein_[Thermoanaerobaculum_aquaticum]	ptg003045l	45.447	0	976	99
WP_038048122.1_response_regulator_[Thermoanaerobaculum_aquaticum]	ptg005341l	31.452	9.14E-13	67	95
WP_038047755.1_response_regulator_[Thermoanaerobaculum_aquaticum]	ptg002316l	38.393	8.81E-20	95.1	31
WP_038047637.1_acyl-CoA_dehydrogenase_family_protein_[Thermoanaerobaculum_aquaticum]	LG02	49.038	1.36E-21	101	65
WP_038047517.1_MotA/TolQ/ExbB_proton_channel_family_protein_[Thermoanaerobaculum_aquaticum]	ptg005341l	34.337	2.23E-19	89.7	74

WP_038046497.1_response_regulator_[Thermoanaerobaculum_aquaticum]	ptg002942l	35.849	1.56E-12	66.2	87
WP_161685667.1_AAA_family_ATPase,_partial_[Thermoanaerobaculum_aquaticum]	ptg002345l	38.129	1.58E-15	79	58
WP_081799987.1_NrfD/PsrC_family_molybdoenzyme_membrane_anchor_subunit_[Thermoanaerobaculum_aquaticum]	ptg002453l	65.188	3.47E-172	541	92
WP_053335129.1_S41_family_peptidase_[Thermoanaerobaculum_aquaticum]	ptg003980l	29.898	1.25E-118	407	97
WP_053335103.1_FAD-dependent_oxidoreductase_[Thermoanaerobaculum_aquaticum]	LG02	30.588	0.00000748	54.7	15
WP_053334922.1_GH36-type_glycosyl_hydrolase_domain-containing_protein_[Thermoanaerobaculum_aquaticum]	ptg004790l	25.392	1.7E-56	215	78
WP_053334904.1_AAA_family_ATPase_[Thermoanaerobaculum_aquaticum]	ptg005527l	26.776	3.05E-19	93.6	89
WP_053334859.1_glycosyltransferase_family_2_protein_[Thermoanaerobaculum_aquaticum]	ptg003213l	32.374	0.00000428	52	46
WP_038049562.1_beta-ketoacyl_synthase_N-terminal-like_domain-containing_protein_[Thermoanaerobaculum_aquaticum]	ptg003980l	24.339	0.00000232	53.5	46
WP_038047422.1_ammonia-forming_cytochrome_c_nitrite_reductase_subunit_c552_[Thermoanaerobaculum_aquaticum]	ptg004519l	25.556	5.17E-08	60.1	43
WP_038046879.1_GDP-mannose_4,6-dehydratase_[Thermoanaerobaculum_aquaticum]	ptg001690l	39.936	1.72E-70	241	99
WP_200867152.1_PLP-dependent_transferase_[Thermoanaerobaculum_aquaticum]	ptg002030l	39.247	4.77E-46	109	73
WP_161685251.1_nitrilase-related_carbon-nitrogen_hydrolase_[Thermoanaerobaculum_aquaticum]	ptg003299l	28.767	4.67E-22	99.8	93
WP_053335286.1_nitrilase-related_carbon-nitrogen_hydrolase_[Thermoanaerobaculum_aquaticum]	ptg004519l	31.868	1.97E-13	73.6	66
WP_053334758.1_SAM-dependent_methyltransferase_[Thermoanaerobaculum_aquaticum]	ptg005689l	36.364	5.38E-16	53.5	91
WP_038050393.1_M1_family_aminopeptidase_[Thermoanaerobaculum_aquaticum]	ptg004724l	20.588	0.00000227	55.1	52
WP_038049175.1_nucleotidyl_transferase_AbiEii/AbiGii_toxin_family_protein_[Thermoanaerobaculum_aquaticum]	ptg002909l	25.882	0.00000134	51.6	79
WP_038047954.1_S1_RNA-binding_domain-containing_protein_[Thermoanaerobaculum_aquaticum]	ptg001025l	33.092	1.05E-45	174	85
WP_038047708.1_DUF6580_family_putative_transport_protein_[Thermoanaerobaculum_aquaticum]	ptg000568l	37.838	3.2E-14	73.2	79
WP_038046638.1_prephenate_dehydrogenase/arogenate_dehydrogenase_family_protein_[Thermoanaerobaculum_aquaticum]	ptg005341l	25.568	1.25E-19	94.7	95
WP_200867163.1_GTPase,_partial_[Thermoanaerobaculum_aquaticum]	LG12	54.167	2.47E-09	59.3	38
WP_152543838.1_transglycosylase_SLT_domain-containing_protein,_partial_[Thermoanaerobaculum_aquaticum]	ptg004256l	36.364	9.42E-12	72.8	22
WP_053335185.1_acyl-CoA_dehydrogenase_family_protein_[Thermoanaerobaculum_aquaticum]	ptg004179l	28.693	4.15E-36	144	87
WP_053335023.1_DUF3179_domain-containing_(seleno)protein_[Thermoanaerobaculum_aquaticum]	ptg003734l	30.566	1E-30	127	74
WP_038050518.1_TonB-dependent_receptor_domain-containing_protein_[Thermoanaerobaculum_aquaticum]	ptg001925l	22.928	2.71E-24	114	90
WP_038049916.1_TonB-dependent_receptor_domain-containing_protein_[Thermoanaerobaculum_aquaticum]	ptg001925l	24.573	0.00000224	55.8	30
WP_038048890.1_COX15/CtaA_family_protein_[Thermoanaerobaculum_aquaticum]	ptg004250l	47.143	2.75E-08	60.1	16
WP_038048217.1_TonB-dependent_receptor_domain-containing_protein_[Thermoanaerobaculum_aquaticum]	ptg001925l	22.569	6.88E-14	80.5	82
WP_161685299.1_SMP-30/gluconolactonase/LRE_family_protein_[Thermoanaerobaculum_aquaticum]	ptg005354l	28.058	5.61E-18	89	75
WP_053335139.1_FHA_domain-containing_protein_[Thermoanaerobaculum_aquaticum]	ptg001238l	48.485	0.000000264	53.9	31
WP_053334928.1_S8_family_serine_peptidase_[Thermoanaerobaculum_aquaticum]	ptg005110l	27.486	2.49E-24	115	63

WP_053334799.1_SMP-30/gluconolactonase/LRE_family_protein_[Thermoanaerobaculum_aquaticum]	ptg005354l	25.532	0.000000151	56.6	85
WP_038050541.1_S8_family_serine_peptidase_[Thermoanaerobaculum_aquaticum]	ptg002270l	26.205	7.28E-08	60.1	43
WP_038050488.1_NADH-quinone_oxidoreductase_subunit_A_[Thermoanaerobaculum_aquaticum]	ptg004143l	41.593	6.99E-24	98.6	93
WP_038050165.1_NADH-quinone_oxidoreductase_subunit_A_[Thermoanaerobaculum_aquaticum]	ptg005338l	36.975	4.14E-18	82.4	92
WP_038050095.1_amino_acid_permease_[Thermoanaerobaculum_aquaticum]	ptg002867l	30.303	3.56E-24	110	96
WP_038049830.1_PhoH_family_protein_[Thermoanaerobaculum_aquaticum]	ptg002760l	33.544	5.18E-10	66.2	32
WP_038049429.1_class_I_SAM-dependent_methyltransferase_[Thermoanaerobaculum_aquaticum]	ptg004863l	30.085	6.76E-26	109	91
WP_038049316.1_acyl-CoA_dehydrogenase_family_protein_[Thermoanaerobaculum_aquaticum]	ptg004179l	32.378	2.33E-33	135	89
WP_038049150.1_acyl-CoA_dehydrogenase_family_protein_[Thermoanaerobaculum_aquaticum]	ptg004179l	27.049	2.23E-25	112	93
WP_038048239.1_ATP-grasp_domain-containing_protein_[Thermoanaerobaculum_aquaticum]	ptg004466l	31.429	5.88E-17	85.5	74
WP_038047402.1_secretin_N-terminal_domain-containing_protein_[Thermoanaerobaculum_aquaticum]	ptg004827l	20.052	5.62E-11	70.5	49
WP_038046511.1_amino_acid_permease_[Thermoanaerobaculum_aquaticum]	ptg002867l	27.221	6.69E-13	75.5	69
WP_235208685.1_sigma_54-interacting_transcriptional_regulator_[Thermoanaerobaculum_aquaticum]	ptg002304l	25.938	1.63E-20	100	52
WP_200867137.1_NAD-dependent_epimerase/dehydratase_family_protein_[Thermoanaerobaculum_aquaticum]	ptg002634l	31.765	6.91E-09	58.9	80
WP_161685501.1_cyclic_nucleotide-binding_domain-containing_protein_[Thermoanaerobaculum_aquaticum]	ptg004457l	33.019	4.34E-09	60.5	42
WP_161685285.1_NAD(P)-dependent_oxidoreductase_[Thermoanaerobaculum_aquaticum]	ptg000619l	27.698	1.57E-17	87.4	83
WP_152543985.1_sigma_54-interacting_transcriptional_regulator_[Thermoanaerobaculum_aquaticum]	ptg001025l	44.015	1.08E-50	186	70
WP_152543972.1_CHRD_domain-containing_protein_[Thermoanaerobaculum_aquaticum]	ptg002848l	30.693	1.06E-10	69.3	28
WP_053335152.1_HAD_hydrolase-like_protein_[Thermoanaerobaculum_aquaticum]	ptg004085l	38.009	1.05E-33	131	94
WP_053335031.1_sigma_54-interacting_transcriptional_regulator_[Thermoanaerobaculum_aquaticum]	ptg005275l	54.545	1.95E-18	80.9	100
WP_053334895.1_PAS_domain-containing_protein_[Thermoanaerobaculum_aquaticum]	ptg005484l	34.197	4.86E-55	210	49
WP_053334730.1_sigma_54-interacting_transcriptional_regulator_[Thermoanaerobaculum_aquaticum]	ptg005150l	36.296	1.75E-41	156	86
WP_038049419.1_amidohydrolase_family_protein_[Thermoanaerobaculum_aquaticum]	ptg004359l	26.617	4.19E-14	79	80
WP_038049170.1_sulfatase-like_hydrolase/transferase_[Thermoanaerobaculum_aquaticum]	ptg003671l	27.126	3.75E-23	109	54
WP_038049122.1_DnaJ_domain-containing_protein_[Thermoanaerobaculum_aquaticum]	ptg002634l	40	3.58E-08	60.5	14
WP_038048732.1_PTS_sugar_transporter_subunit_IIA_[Thermoanaerobaculum_aquaticum]	ptg005709l	39.189	3.21E-09	60.1	32
WP_038048537.1_HRDC_domain-containing_protein_[Thermoanaerobaculum_aquaticum]	ptg002760l	29.682	6.17E-35	140	75
WP_038048362.1_lyase_family_protein_[Thermoanaerobaculum_aquaticum]	ptg005906l	40.22	1.69E-84	287	98

WP_038048044.1_NAD-dependent_epimerase/dehydratase_family_protein_[Thermoanaerobaculum_aquaticum]	ptg003768l	34.385	4.52E-47	173	99
WP_038047055.1_CDGSH_iron-sulfur_domain-containing_protein_[Thermoanaerobaculum_aquaticum]	ptg003058l	48.148	8.21E-19	82	100
WP_038049043.1_B12-binding_domain-containing_radical_SAM_protein_[Thermoanaerobaculum_aquaticum]	ptg003511l	25.339	1.72E-08	61.2	41
WP_038049893.1_elongation_factor_P_[Thermoanaerobaculum_aquaticum]	LG03	38.71	7.74E-30	118	100
WP_038049762.1_secondary_thiamine-phosphate_synthase_enzyme_YjbQ_[Thermoanaerobaculum_aquaticum]	ptg004959l	28.099	5.6E-10	59.7	84
WP_038046398.1_secondary_thiamine-phosphate_synthase_enzyme_YjbQ_[Thermoanaerobaculum_aquaticum]	ptg005825l	49.485	3.36E-18	82.8	70
WP_038047705.1_Nif3-like_dinuclear_metal_center_hexameric_protein_[Thermoanaerobaculum_aquaticum]	ptg002270l	36.546	3.68E-39	148	99
WP_038046237.1_membrane_protein_insertase_YidC_[Thermoanaerobaculum_aquaticum]	ptg000372l	40.249	1.65E-49	187	48
WP_053335100.1_lysophospholipid_acyltransferase_family_protein_[Thermoanaerobaculum_aquaticum]	ptg004147l	26.009	1.44E-09	62.8	73
WP_053334993.1_M23_family_metallopeptidase_[Thermoanaerobaculum_aquaticum]	ptg004510l	28.696	0.00000066	55.1	34
WP_053334795.1_M23_family_metallopeptidase_[Thermoanaerobaculum_aquaticum]	ptg004510l	45.69	8.88E-27	113	40
WP_038050076.1_NADH-quinone_oxidoreductase_subunit_N_[Thermoanaerobaculum_aquaticum]	ptg004143l	36.311	1.1E-51	192	71
WP_038049954.1_HU_family_DNA-binding_protein_[Thermoanaerobaculum_aquaticum]	ptg005527l	43.956	6.26E-14	68.9	96
WP_038049953.1_HU_family_DNA-binding_protein_[Thermoanaerobaculum_aquaticum]	ptg005527l	46.809	2.82E-18	81.3	99
WP_038049509.1_fumarylacetoacetate_hydrolase_family_protein_[Thermoanaerobaculum_aquaticum]	ptg003204l	45.5	9.49E-50	178	74
WP_038049026.1_enoyl-CoA_hydratase/isomerase_family_protein_[Thermoanaerobaculum_aquaticum]	ptg001245l	32.819	5.23E-19	89.7	98
WP_038048775.1_HU_family_DNA-binding_protein_[Thermoanaerobaculum_aquaticum]	ptg003072l	46.667	1.53E-13	67.8	99
WP_038048622.1_replication-associated_recombination_protein_A_[Thermoanaerobaculum_aquaticum]	ptg001690l	47.242	4.93E-95	317	93
WP_038048358.1_HU_family_DNA-binding_protein_[Thermoanaerobaculum_aquaticum]	ptg005527l	44.706	9.08E-18	79.7	96
WP_038047695.1_NAD(P)H-dependent_glycerol-3-phosphate_dehydrogenase_[Thermoanaerobaculum_aquaticum]	ptg001005l	38.066	1.77E-46	172	99
WP_038047590.1_3-hydroxyacyl-CoA_dehydrogenase/enoyl-CoA_hydratase_family_protein_[Thermoanaerobaculum_aquaticum]	ptg002760l	28.115	1.72E-22	108	38
WP_038047473.1_NADH-quinone_oxidoreductase_subunit_N_[Thermoanaerobaculum_aquaticum]	ptg004143l	32.849	3.12E-26	116	70
WP_038046218.1_enoyl-CoA_hydratase/isomerase_family_protein_[Thermoanaerobaculum_aquaticum]	ptg004214l	34.028	1.99E-10	64.3	55
WP_268746958.1_MotA/TolQ/ExbB_proton_channel_family_protein_[Thermoanaerobaculum_aquaticum]	ptg005341l	36.126	7.48E-19	87.8	87
WP_161685234.1_RsmB/NOP_family_class_I_SAM-dependent_RNA_methyltransferase_[Thermoanaerobaculum_aquaticum]	ptg003098l	27.857	8.01E-16	84	64
WP_152543995.1_DUF502_domain-containing_protein_[Thermoanaerobaculum_aquaticum]	ptg002909l	28.037	4.53E-14	74.3	86
WP_081799925.1_glycosyltransferase_family_39_protein_[Thermoanaerobaculum_aquaticum]	ptg000909l	28.689	2.84E-13	76.6	60
WP_081799829.1_SDR_family_oxidoreductase_[Thermoanaerobaculum_aquaticum]	ptg005905l	32.24	5.78E-11	66.2	69
WP_053335209.1_ceramide_glucosyltransferase_[Thermoanaerobaculum_aquaticum]	ptg003511l	29.6	0.000000421	56.2	31

WP_053335161.1_dolichyl-phosphate_beta-glucosyltransferase_[Thermoanaerobaculum_aquaticum]	ptg003431l	27.7	1.24E-16	82.8	81
WP_053335035.1_ABC_transporter_ATP-binding_protein_[Thermoanaerobaculum_aquaticum]	ptg003163l	35.616	3.94E-47	175	77
WP_053334750.1_M4_family_metallopeptidase_[Thermoanaerobaculum_aquaticum]	ptg003282l	48.78	3.97E-13	77.4	11
WP_038050443.1_indole-3-glycerol-phosphate_synthase_[Thermoanaerobaculum_aquaticum]	ptg004054l	36.293	1.22E-39	149	99
WP_038049791.1_ABC_transporter_permease_[Thermoanaerobaculum_aquaticum]	ptg003336l	44.872	4.6E-48	174	90
WP_038049790.1_replicative_DNA_helicase_[Thermoanaerobaculum_aquaticum]	ptg005535l	39.374	1.05E-80	276	97
WP_038049469.1_ABC_transporter_ATP-binding_protein_[Thermoanaerobaculum_aquaticum]	ptg002820l	42.857	1.1E-44	162	98
WP_038049324.1_ABC_transporter_ATP-binding_protein_[Thermoanaerobaculum_aquaticum]	ptg000372l	27.605	6.65E-42	166	91
WP_038048785.1_ABC_transporter_ATP-binding_protein_[Thermoanaerobaculum_aquaticum]	ptg004774l	39.606	2.46E-84	292	74
WP_038048574.1_bifunctional_nuclease_family_protein_[Thermoanaerobaculum_aquaticum]	ptg003247l	37.374	3.34E-17	81.3	61
WP_038048555.1_ABC_transporter_ATP-binding_protein_[Thermoanaerobaculum_aquaticum]	ptg001659l	58.571	6.37E-79	261	93
WP_038047746.1_co-chaperone_GroES_[Thermoanaerobaculum_aquaticum]	ptg004085l	48.352	1.32E-21	90.9	95
WP_038047146.1_ABC_transporter_ATP-binding_protein_[Thermoanaerobaculum_aquaticum]	ptg001659l	51.835	8.22E-63	215	94
WP_038047008.1_ABC_transporter_permease_[Thermoanaerobaculum_aquaticum]	ptg003336l	41.026	6.89E-37	142	88
WP_200867134.1_MBL_fold_metallo-hydrolase_[Thermoanaerobaculum_aquaticum]	ptg005425l	27.189	8.27E-14	73.6	88
WP_161685293.1_class_I_SAM-dependent_methyltransferase_[Thermoanaerobaculum_aquaticum]	ptg002534l	35.849	1.61E-09	61.6	39
WP_152543917.1_class_I_SAM-dependent_methyltransferase_[Thermoanaerobaculum_aquaticum]	ptg004186l	35.616	0.000000421	54.3	29
WP_081800052.1_transketolase_[Thermoanaerobaculum_aquaticum]	ptg004917l	46.429	0	596	55
WP_053335243.1_RsmD_family_RNA_methyltransferase_[Thermoanaerobaculum_aquaticum]	ptg004885l	30.814	0.000000283	53.5	83
WP_053335195.1_transcriptional_coactivator_p15/PC4_family_protein_[Thermoanaerobaculum_aquaticum]	LG22	47.727	0.00000161	47.4	52
WP_053335193.1_dTDP-4-dehydrorhamnose_3,5-epimerase_family_protein_[Thermoanaerobaculum_aquaticum]	ptg003827l	26.829	0.00000582	49.3	87
WP_053335159.1_energy_transducer_TonB_[Thermoanaerobaculum_aquaticum]	ptg005754l	37.975	0.000000282	54.7	33
WP_053335141.1_ABC_transporter_ATP-binding_protein_[Thermoanaerobaculum_aquaticum]	ptg003137l	41.833	5.03E-58	202	93
WP_038050420.1_ribosome_recycling_factor_[Thermoanaerobaculum_aquaticum]	ptg004895l	49.398	3.74E-46	165	90
WP_038050375.1_type_4a_pilus_biogenesis_protein_PilO_[Thermoanaerobaculum_aquaticum]	ptg004771l	28.689	1.33E-08	57	66
WP_038050019.1_NupC/NupG_family_nucleoside_CNT_transporter_[Thermoanaerobaculum_aquaticum]	ptg001925l	44.414	8.34E-75	257	88
WP_038049838.1_RimK_family_alpha-L-glutamate_ligase_[Thermoanaerobaculum_aquaticum]	ptg002658l	26.636	8.46E-12	69.3	72
WP_038049082.1_M28_family_metallopeptidase_[Thermoanaerobaculum_aquaticum]	ptg005732l	35.885	2.89E-22	105	37
WP_038048347.1_single-stranded_DNA-binding_protein_[Thermoanaerobaculum_aquaticum]	ptg005756l	34.906	1.07E-14	72.8	78
WP_235208735.1_type_IV_pilus_secretin_PilQ_[Thermoanaerobaculum_aquaticum]	ptg004863l	33.691	4.65E-56	204	96
WP_200867104.1_gamma_carbonic_anhydrase_family_protein_[Thermoanaerobaculum_aquaticum]	ptg004532l	42.045	4.37E-43	156	91
WP_081799992.1_GTP_cyclohydrolase_I_FolE_[Thermoanaerobaculum_aquaticum]	ptg002479l	48.315	1.78E-48	172	84
WP_081799856.1_amidohydrolase_[Thermoanaerobaculum_aquaticum]	ptg003746l	29.717	1.27E-10	68.6	37
WP_053334921.1_carbohydrate_ABC_transporter_permease_[Thermoanaerobaculum_aquaticum]	ptg003163l	34.595	3.08E-14	76.3	66

WP_053334901.1_peptidylprolyl_isomerase_[Thermoanaerobaculum_aquaticum]	ptg002976l	52.866	1.14E-51	181	82
WP_053334834.1_response_regulator_transcription_factor_[Thermoanaerobaculum_aquaticum]	ptg004579l	31.696	6.2E-25	106	90
WP_038050543.1_TatD_family_hydrolase_[Thermoanaerobaculum_aquaticum]	ptg005067l	39.3	4.31E-36	139	97
WP_038048241.1_GNAT_family_N-acetyltransferase_[Thermoanaerobaculum_aquaticum]	LG03	41.463	2.28E-11	64.3	51
WP_038048178.1_A24_family_peptidase_[Thermoanaerobaculum_aquaticum]	ptg002916l	37.037	1.72E-25	71.2	90
WP_038047950.1_response_regulator_transcription_factor_[Thermoanaerobaculum_aquaticum]	LG03	37.553	4.84E-43	158	99
WP_038047785.1_peptide_MFS_transporter_[Thermoanaerobaculum_aquaticum]	ptg003813l	32.545	8.79E-60	218	92
WP_038047448.1_phosphoribosylformylglycinamidine_cyclo-ligase_[Thermoanaerobaculum_aquaticum]	ptg005227l	44.51	4.01E-83	278	97
WP_038047212.1_DNA_repair_protein_RecN_[Thermoanaerobaculum_aquaticum]	ptg005922l	32.92	2.36E-57	211	99
WP_038047122.1_PP2C_family_protein-serine/threonine_phosphatase_[Thermoanaerobaculum_aquaticum]	ptg004476l	29.197	5.17E-11	65.1	64
WP_038046131.1_response_regulator_transcription_factor_[Thermoanaerobaculum_aquaticum]	ptg004579l	39.912	2.29E-49	176	96
WP_200867095.1_aminoacyl-tRNA_hydrolase_[Thermoanaerobaculum_aquaticum]	ptg005867l	41.341	1.8E-26	108	93
WP_161685484.1_acyl-CoA_dehydrogenase_[Thermoanaerobaculum_aquaticum]	ptg005087l	36.995	1.9E-107	360	99
WP_161685238.1_class_IV_adenylate_cyclase_[Thermoanaerobaculum_aquaticum]	ptg003361l	32.558	0.0000026	50.4	67
WP_152544043.1_metallophosphoesterase_[Thermoanaerobaculum_aquaticum]	ptg003471l	31.984	8.97E-19	92	64
WP_053335211.1_GntR_family_transcriptional_regulator_[Thermoanaerobaculum_aquaticum]	ptg004986l	40.26	2.87E-12	65.9	59
WP_053335172.1_RHS_repeat-associated_core_domain-containing_protein_[Thermoanaerobaculum_aquaticum]	ptg002381l	36.782	3.02E-22	102	49
WP_053334944.1_metallophosphoesterase_[Thermoanaerobaculum_aquaticum]	ptg003471l	28.926	1.72E-13	74.3	79
WP_053334675.1_methyl-accepting_chemotaxis_protein_[Thermoanaerobaculum_aquaticum]	ptg006036l	34.101	6.33E-14	79	38
WP_038050428.1_efflux_RND_transporter_periplasmic_adaptor_subunit_[Thermoanaerobaculum_aquaticum]	ptg000732l	26.923	4.05E-18	89.4	65
WP_038050031.1_efflux_RND_transporter_periplasmic_adaptor_subunit_[Thermoanaerobaculum_aquaticum]	ptg005906l	25.862	0.00000467	53.1	55
WP_038049279.1_UDP-glucuronic_acid_decarboxylase_family_protein_[Thermoanaerobaculum_aquaticum]	ptg005433l	53.094	9.5E-96	313	97
WP_038048550.1_efflux_RND_transporter_periplasmic_adaptor_subunit_[Thermoanaerobaculum_aquaticum]	ptg005906l	27.803	6.5E-09	62	54
WP_038048057.1_tryptophanase_[Thermoanaerobaculum_aquaticum]	ptg004361l	56.637	9.08E-168	527	99
WP_038047581.1_PP2C_family_protein-serine/threonine_phosphatase_[Thermoanaerobaculum_aquaticum]	ptg004476l	29.032	1.23E-13	77	58
WP_038047485.1_patatin-like_phospholipase_family_protein_[Thermoanaerobaculum_aquaticum]	ptg004384l	27.857	5.35E-24	105	92
WP_038047148.1_efflux_RND_transporter_periplasmic_adaptor_subunit_[Thermoanaerobaculum_aquaticum]	ptg000732l	25.98	7.78E-11	67.8	52
WP_038047107.1_efflux_RND_transporter_periplasmic_adaptor_subunit_[Thermoanaerobaculum_aquaticum]	ptg001766l	29.722	1.68E-27	119	88
WP_038046829.1_efflux_RND_transporter_periplasmic_adaptor_subunit_[Thermoanaerobaculum_aquaticum]	ptg000732l	24.742	0.00000017	57	50
WP_038046310.1_peroxiredoxin_[Thermoanaerobaculum_aquaticum]	ptg002047l	49.162	4.61E-51	179	91
WP_038046249.1_FAD:protein_FMN_transferase_[Thermoanaerobaculum_aquaticum]	ptg004017l	29.249	1.18E-20	96.3	76
WP_161685305.1_pseudouridine_synthase_[Thermoanaerobaculum_aquaticum]	ptg002909l	46.429	2.62E-25	105	71

WP_081799934.1_LacI_family_DNA-binding_transcriptional_regulator_[Thermoanaerobaculum_aquaticum]	ptg005822l	33.228	2.43E-37	146	90
WP_081799914.1_NYN_domain-containing_protein_[Thermoanaerobaculum_aquaticum]	ptg002557l	30.769	3.56E-19	91.3	77
WP_053334953.1_class_I_SAM-dependent_methyltransferase_[Thermoanaerobaculum_aquaticum]	ptg002534l	28.986	0.000000965	53.1	48
WP_053334821.1_apolipoprotein_N-acyltransferase_[Thermoanaerobaculum_aquaticum]	ptg004790l	30.258	1.02E-26	118	52
WP_038050527.1_MBL_fold_metallo-hydrolase_[Thermoanaerobaculum_aquaticum]	ptg002918l	27.953	2.53E-29	120	90
WP_038050431.1_hydroxyacid_dehydrogenase_[Thermoanaerobaculum_aquaticum]	ptg003603l	43.939	2.76E-62	216	88
WP_038050084.1_sigma-70_family_RNA_polymerase_sigma_factor_[Thermoanaerobaculum_aquaticum]	ptg004479l	29.268	1.73E-21	95.5	79
WP_038050029.1_macro_domain-containing_protein_[Thermoanaerobaculum_aquaticum]	ptg003471l	39.516	1.48E-15	77.4	63
WP_038048591.1_transglycosylase_domain-containing_protein_[Thermoanaerobaculum_aquaticum]	ptg005297l	32.846	1.09E-72	260	84
WP_038048396.1_NAD(P)(+)_transhydrogenase_(Re/Si-specific)_subunit_beta_[Thermoanaerobaculum_aquaticum]	ptg005929l	51.974	2.46E-105	347	97
WP_038048113.1_Mrp/NBP35_family_ATP-binding_protein_[Thermoanaerobaculum_aquaticum]	ptg002214l	47.907	1.02E-64	225	62
WP_038047628.1_alanine_dehydrogenase_[Thermoanaerobaculum_aquaticum]	ptg003980l	38.275	8.51E-49	180	99
WP_038047438.1_MBL_fold_metallo-hydrolase_[Thermoanaerobaculum_aquaticum]	ptg004509l	38.073	5.35E-41	153	85
WP_038047387.1_type_II_secretion_system_F_family_protein_[Thermoanaerobaculum_aquaticum]	ptg005301l	31.965	5.56E-46	173	85
WP_038047369.1_type_II_secretion_system_F_family_protein_[Thermoanaerobaculum_aquaticum]	ptg005301l	39.85	4.03E-88	295	99
WP_161685386.1_peroxiredoxin_[Thermoanaerobaculum_aquaticum]	ptg004538l	32.258	9.51E-13	68.9	70
WP_152543832.1_gluconeogenesis_factor_YvcK_family_protein_[Thermoanaerobaculum_aquaticum]	ptg004284l	30.032	8.8E-15	79	75
WP_053335276.1_NADH-quinone_oxidoreductase_subunit_I_[Thermoanaerobaculum_aquaticum]	ptg004143l	30.841	0.00000277	50.1	57
WP_053335107.1_diacylglycerol_kinase_family_protein_[Thermoanaerobaculum_aquaticum]	ptg004476l	24.333	0.000000123	57	95
WP_053335078.1_lytic_transglycosylase_domain-containing_protein_[Thermoanaerobaculum_aquaticum]	ptg003673l	36.957	0.00000071	53.1	38
WP_053334839.1_lytic_transglycosylase_domain-containing_protein_[Thermoanaerobaculum_aquaticum]	ptg005110l	37.113	1.88E-11	67	40
WP_053334826.1_gamma-glutamyl-gamma-aminobutyrate_hydrolase_family_protein_[Thermoanaerobaculum_aquaticum]	LG10	32.843	2.96E-20	92.4	84
WP_038050520.1_PstS_family_phosphate_ABC_transporter_substrate-binding_protein,_partial_[Thermoanaerobaculum_aquaticum]	ptg002567l	57.895	4.13E-99	322	92
WP_038050390.1_DUF1820_family_protein_[Thermoanaerobaculum_aquaticum]	ptg004790l	47	1.01E-25	103	86
WP_038050376.1_PilN_domain-containing_protein_[Thermoanaerobaculum_aquaticum]	ptg004863l	31.579	1.75E-12	68.9	74
WP_038050030.1_ABC_transporter_permease_[Thermoanaerobaculum_aquaticum]	ptg004986l	33.185	1.04E-32	134	98
WP_038049905.1_DUF1156_domain-containing_protein_[Thermoanaerobaculum_aquaticum]	ptg003045l	42.957	0	758	98
WP_038049339.1_ABC_transporter_permease_[Thermoanaerobaculum_aquaticum]	ptg004986l	31.602	9.47E-18	89.4	51
WP_038048802.1_diacylglycerol_kinase_family_protein_[Thermoanaerobaculum_aquaticum]	ptg004476l	24.229	0.000000385	55.1	76
WP_038048656.1_RtcB_family_protein_[Thermoanaerobaculum_aquaticum]	ptg003039l	30.562	5.69E-30	128	91
WP_038048638.1_efflux_RND_transporter_permease_subunit_[Thermoanaerobaculum_aquaticum]	ptg004670l	32.464	1.39E-174	572	99
WP_038048556.1_ABC_transporter_permease_[Thermoanaerobaculum_aquaticum]	ptg004986l	31.461	4.71E-37	147	99

WP_038048482.1_diacylglycerol_kinase_family_protein_[Thermoanaerobaculum_aquaticum]	ptg004476l	27.5	5.49E-14	75.9	80
WP_038048412.1_penicillin_acylase_family_protein_[Thermoanaerobaculum_aquaticum]	ptg004534l	28.136	1.34E-44	178	85
WP_038046687.1_inorganic_phosphate_transporter_[Thermoanaerobaculum_aquaticum]	ptg004169l	33.846	4.49E-12	70.9	38
WP_038046382.1_mechanosensitive_ion_channel_family_protein_[Thermoanaerobaculum_aquaticum]	ptg003982l	38.211	4.8E-28	125	36
WP_200867155.1_PaaI_family_thioesterase_[Thermoanaerobaculum_aquaticum]	ptg003942l	51.974	1.82E-48	170	99
WP_200867115.1_ferritin_[Thermoanaerobaculum_aquaticum]	ptg001693l	48.052	4.89E-40	146	93
WP_152544021.1_ankyrin_repeat_domain-containing_protein_[Thermoanaerobaculum_aquaticum]	ptg004364l	34.812	1.96E-60	222	85
WP_081799862.1_DMT_family_transporter_[Thermoanaerobaculum_aquaticum]	ptg002270l	25.279	5.12E-11	67.4	86
WP_053335101.1_LemA_family_protein_[Thermoanaerobaculum_aquaticum]	ptg005195l	42.529	5.39E-27	110	87
WP_053334816.1_3-deoxy-D-manno-octulosonic_acid_transferase_[Thermoanaerobaculum_aquaticum]	ptg001658l	36.193	2.93E-50	186	87
WP_053334808.1_PIG-L_deacetylase_family_protein_[Thermoanaerobaculum_aquaticum]	ptg005204l	37.5	7.14E-09	59.7	42
WP_053334806.1_sigma-54-dependent_Fis_family_transcriptional_regulator_[Thermoanaerobaculum_aquaticum]	ptg003137l	44.805	4.82E-57	208	62
WP_053334753.1_PIG-L_deacetylase_family_protein_[Thermoanaerobaculum_aquaticum]	ptg004000l	27.451	3.53E-08	57.4	79
WP_038050325.1_slipin_family_protein_[Thermoanaerobaculum_aquaticum]	ptg002123l	30.4	1.13E-11	68.2	48
WP_038050282.1_peptidylprolyl_isomerase_[Thermoanaerobaculum_aquaticum]	LG03	43.137	3.54E-33	126	99
WP_038049913.1_alpha/beta_fold_hydrolase_[Thermoanaerobaculum_aquaticum]	ptg005922l	26.705	0.00000476	52	55
WP_038049507.1_alpha/beta_fold_hydrolase_[Thermoanaerobaculum_aquaticum]	ptg005092l	28.632	2.54E-10	64.7	83
WP_038049351.1_PTS_sugar_transporter_subunit_IIA_[Thermoanaerobaculum_aquaticum]	ptg003536l	34.286	2.19E-16	79.3	81
WP_038048580.1_PTS_sugar_transporter_subunit_IIA_[Thermoanaerobaculum_aquaticum]	ptg005709l	32.812	2.31E-15	75.5	84
WP_038048542.1_acyclic_terpene_utilization_AtuA_family_protein_[Thermoanaerobaculum_aquaticum]	ptg004500l	56.733	1.26E-165	521	99
WP_038047982.1_glycine_cleavage_system_protein_H_[Thermoanaerobaculum_aquaticum]	ptg005318l	30.208	3.93E-08	57	43
WP_038046581.1_PTS_sugar_transporter_subunit_IIA_[Thermoanaerobaculum_aquaticum]	ptg004997l	31.515	7.73E-13	72	58
WP_038046407.1_dicarboxylate/amino_acid:cation_symporter_[Thermoanaerobaculum_aquaticum]	ptg001291l	33.172	1.75E-64	228	98
WP_038046271.1_LemA_family_protein_[Thermoanaerobaculum_aquaticum]	ptg005363l	55.172	1.04E-50	178	89
WP_038046188.1_deoxynucleoside_kinase_[Thermoanaerobaculum_aquaticum]	ptg004203l	27.835	2.51E-10	63.2	79
WP_200867128.1_S9_family_peptidase_[Thermoanaerobaculum_aquaticum]	ptg003703l	33.846	1.15E-20	97.4	54
WP_161685585.1_RNA_polymerase_sigma_factor_[Thermoanaerobaculum_aquaticum]	ptg002465l	36.464	2.84E-28	114	93
WP_161685520.1_PAS_domain-containing_sensor_histidine_kinase_[Thermoanaerobaculum_aquaticum]	ptg001909l	40.909	2.98E-09	63.5	19
WP_161685367.1_PAS_domain-containing_sensor_histidine_kinase_[Thermoanaerobaculum_aquaticum]	ptg001025l	25.672	1.8E-16	84.7	91
WP_161685325.1_nitrogen_regulation_protein_NR(II)_[Thermoanaerobaculum_aquaticum]	ptg003057l	30.29	5.44E-26	117	41
WP_152544005.1_RNA_polymerase_sigma_factor_[Thermoanaerobaculum_aquaticum]	ptg002465l	30.994	1.57E-12	69.3	83
WP_152543948.1_class_I_SAM-dependent_RNA_methyltransferase_[Thermoanaerobaculum_aquaticum]	ptg001291l	34.174	6.27E-44	166	92

WP_152543866.1_sensor_histidine_kinase_[Thermoanaerobaculum_aquaticum]	ptg003057l	31.056	3.68E-28	123	56
WP_081800079.1_folylpolyglutamate_synthase/dihydrofolate_synthase_family_protein_[Thermoanaerobaculum_aquaticum]	ptg003967l	30.671	1.74E-27	119	69
WP_081800034.1_dipeptidase_[Thermoanaerobaculum_aquaticum]	LG02	40.58	0.000000493	57	13
WP_081799787.1_site-specific_DNA-methyltransferase_[Thermoanaerobaculum_aquaticum]	ptg003009l	43.522	9.49E-127	431	90
WP_053335280.1_RNA_polymerase_sigma_factor_[Thermoanaerobaculum_aquaticum]	ptg004479l	26.136	3.52E-10	62	90
WP_053335237.1_tRNA_(guanosine(46)-N(7))-methyltransferase_TrmB_[Thermoanaerobaculum_aquaticum]	ptg004880l	30.939	3.47E-09	59.7	84
WP_053335097.1_HD-GYP_domain-containing_protein_[Thermoanaerobaculum_aquaticum]	ptg005035l	32.819	5.63E-31	132	41
WP_053335008.1_ScpA_family_protein_[Thermoanaerobaculum_aquaticum]	ptg005938l	35.789	1.42E-12	70.9	35
WP_053334932.1_murein_hydrolase_activator_EnvC_[Thermoanaerobaculum_aquaticum]	ptg005526l	35.976	2.42E-20	97.1	42
WP_053334838.1_acetyl-CoA_hydrolase/transferase_family_protein_[Thermoanaerobaculum_aquaticum]	LG22	70.588	1.32E-12	73.9	24
WP_053334817.1_RNA_polymerase_sigma_factor_[Thermoanaerobaculum_aquaticum]	ptg004479l	33.14	7.62E-22	96.3	80
WP_053334766.1_sigma-54-dependent_Fis_family_transcriptional_regulator_[Thermoanaerobaculum_aquaticum]	ptg003057l	43.082	5.73E-53	209	25
WP_053334710.1_UDP-N-acetylmuramate--L-alanine_ligase_[Thermoanaerobaculum_aquaticum]	ptg004510l	28.621	4.62E-27	84	87
WP_053334673.1_protein-glutamate_O-methyltransferase_CheR_[Thermoanaerobaculum_aquaticum]	ptg005265l	33.649	1.07E-27	120	45
WP_038050366.1_metallophosphoesterase_[Thermoanaerobaculum_aquaticum]	ptg005702l	40.244	1.18E-16	58.2	99
WP_038050320.1_bifunctional_diguanylate_cyclase/phosphodiesterase_[Thermoanaerobaculum_aquaticum]	ptg005265l	31.271	4.28E-64	236	76
WP_038050126.1_L-aspartate_oxidase,_partial_[Thermoanaerobaculum_aquaticum]	ptg004479l	42.931	4.5E-88	295	99
WP_038050104.1_ExeA_family_protein_[Thermoanaerobaculum_aquaticum]	ptg004623l	43.825	1.75E-57	201	93
WP_038050088.1_PAS_domain-containing_sensor_histidine_kinase_[Thermoanaerobaculum_aquaticum]	ptg003137l	29.437	4.18E-16	88.6	18
WP_038049878.1_PAS_domain-containing_sensor_histidine_kinase_[Thermoanaerobaculum_aquaticum]	ptg004026l	25.431	2.79E-16	87.8	33
WP_038049146.1_menaquinone_biosynthesis_family_protein_[Thermoanaerobaculum_aquaticum]	ptg003947l	32.979	2.4E-35	138	95
WP_038048846.1_HlyD_family_secretion_protein_[Thermoanaerobaculum_aquaticum]	ptg004663l	33.929	5.15E-11	67.4	35
WP_038048161.1_acetyl/propionyl/methylcrotonyl-CoA_carboxylase_subunit_alpha_[Thermoanaerobaculum_aquaticum]	ptg004240l	46.593	1.07E-118	387	91
WP_038047978.1_PAS_domain-containing_sensor_histidine_kinase_[Thermoanaerobaculum_aquaticum]	ptg002453l	29.796	1.55E-23	109	45
WP_038047694.1_sensor_histidine_kinase_KdpD_[Thermoanaerobaculum_aquaticum]	ptg005614l	26.244	6.32E-13	73.2	70
WP_038047616.1_HD_family_phosphohydrolase_[Thermoanaerobaculum_aquaticum]	ptg004524l	44.444	5.38E-56	212	40
WP_038047573.1_enoyl-CoA_hydratase/isomerase_family_protein_[Thermoanaerobaculum_aquaticum]	ptg002612l	46.124	8.81E-52	184	97
WP_038047224.1_PP2C_family_protein-serine/threonine_phosphatase_[Thermoanaerobaculum_aquaticum]	ptg004476l	26.482	2.63E-11	68.6	76
WP_038047105.1_efflux_RND_transporter_permease_subunit_[Thermoanaerobaculum_aquaticum]	ptg004670l	40.811	0	676	99
WP_038046709.1_HD-GYP_domain-containing_protein_[Thermoanaerobaculum_aquaticum]	ptg005301l	26.119	1.28E-09	63.9	35
WP_235208695.1_RHS_repeat_domain-containing_protein_[Thermoanaerobaculum_aquaticum]	ptg004854l	28.755	3.36E-14	78.6	56

WP_161685401.1_RHS_repeat_domain-containing_protein_[Thermoanaerobaculum_aquaticum]	ptg002381l	42.308	2.79E-23	103	47
WP_152544061.1_RHS_repeat_domain-containing_protein_[Thermoanaerobaculum_aquaticum]	ptg002381l	36.111	2.9E-09	62.4	41
WP_152543937.1_bifunctional_2-polyprenyl-6-hydroxyphenol_methylase/3-demethylubiquinol_3-O-methyltransferase_UbiG_[Thermoanaerobaculum_aquaticum]	ptg005770l	29.012	0.00000249	53.5	43
WP_081799877.1_RHS_repeat_domain-containing_protein_[Thermoanaerobaculum_aquaticum]	ptg004854l	28.571	9.19E-18	91.7	56
WP_053335171.1_RHS_repeat_domain-containing_protein_[Thermoanaerobaculum_aquaticum]	ptg002381l	28.205	6.17E-22	101	81
WP_053334873.1_ubiquinol-cytochrome_c_reductase_iron-sulfur_subunit_[Thermoanaerobaculum_aquaticum]	ptg004676l	36.047	0.00000288	49.3	53
WP_053334825.1_RHS_repeat_domain-containing_protein_[Thermoanaerobaculum_aquaticum]	ptg004854l	29.118	2.53E-21	100	76
WP_053334768.1_aldehyde_dehydrogenase_[Thermoanaerobaculum_aquaticum]	ptg004321l	32.883	1.5E-60	220	81
WP_053334699.1_16S_rRNA_(guanine(527)-N(7))-methyltransferase_RsmG_[Thermoanaerobaculum_aquaticum]	ptg003829l	31.707	6.54E-09	58.5	42
WP_038050563.1_RHS_repeat_domain-containing_protein_[Thermoanaerobaculum_aquaticum]	ptg002381l	27.935	1.88E-16	87	47
WP_038050515.1_methylmalonyl-CoA_mutase_[Thermoanaerobaculum_aquaticum]	LG30	60.345	3.84E-12	73.6	44
WP_038050430.1_DUF1015_domain-containing_protein_[Thermoanaerobaculum_aquaticum]	ptg001693l	45.673	3.05E-114	371	100
WP_038049839.1_response_regulator_transcription_factor_[Thermoanaerobaculum_aquaticum]	ptg002316l	43.59	2.3E-28	111	98
WP_038049425.1_MBL_fold_metallo-hydrolase_RNA_specificity_domain-containing_protein_[Thermoanaerobaculum_aquaticum]	ptg003382l	39.53	6.9E-96	320	99
WP_038047731.1_pyridoxal_phosphate-dependent_aminotransferase_[Thermoanaerobaculum_aquaticum]	ptg004905l	33.146	4.79E-42	161	90
WP_038047503.1_RHS_repeat_domain-containing_protein_[Thermoanaerobaculum_aquaticum]	ptg002381l	25.225	1.55E-08	58.5	88
WP_038047484.1_glycosyltransferase_family_4_protein_[Thermoanaerobaculum_aquaticum]	ptg004384l	29.323	0.00000515	52.8	31
WP_235208741.1_RHS_repeat_domain-containing_protein_[Thermoanaerobaculum_aquaticum]	ptg002381l	32.394	1.45E-12	73.6	34
WP_152543949.1_outer_membrane_protein_assembly_factor_[Thermoanaerobaculum_aquaticum]	ptg005656l	23.415	7.7E-17	90.1	58
WP_081800011.1_3-hydroxyacyl-CoA_dehydrogenase_family_protein_[Thermoanaerobaculum_aquaticum]	ptg002612l	33.684	7.08E-39	149	94
WP_081799967.1_glycosyltransferase_[Thermoanaerobaculum_aquaticum]	ptg004111l	31.474	5.5E-25	110	68
WP_081799854.1_helix-turn-helix_transcriptional_regulator_[Thermoanaerobaculum_aquaticum]	ptg001034l	38.806	0.000000127	52.8	50
WP_053335242.1_type_IV_pilus_twitching_motility_protein_PilT_[Thermoanaerobaculum_aquaticum]	ptg004468l	32.016	4.96E-36	143	69
WP_053335079.1_NADH-quinone_oxidoreductase_subunit_B_[Thermoanaerobaculum_aquaticum]	ptg004143l	50.685	1.07E-44	160	86
WP_053334764.1_S9_family_peptidase_[Thermoanaerobaculum_aquaticum]	ptg003382l	35.195	1.58E-100	343	80
WP_053334746.1_heme_lyase_CcmF/NrfE_family_subunit_[Thermoanaerobaculum_aquaticum]	ptg002942l	41.107	1.3E-38	160	30
WP_053334743.1_protein-disulfide_reductase_DsbD_[Thermoanaerobaculum_aquaticum]	ptg004779l	29.787	4.31E-23	108	59
WP_038050560.1_RHS_repeat_domain-containing_protein,_partial_[Thermoanaerobaculum_aquaticum]	ptg002381l	34.783	3.25E-10	65.9	30
WP_038050547.1_SIS_domain-containing_protein_[Thermoanaerobaculum_aquaticum]	ptg005611l	44.937	3.48E-66	228	99
WP_038050449.1_anthranilate_synthase_component_I_family_protein_[Thermoanaerobaculum_aquaticum]	ptg002760l	42.137	5.85E-104	345	99
WP_038049551.1_helix-turn-helix_transcriptional_regulator_[Thermoanaerobaculum_aquaticum]	ptg005046l	37.037	6.1E-11	61.2	69

WP_038047630.1_SDR_family_NAD(P)-dependent_oxidoreductase_[Thermoanaerobaculum_aquaticum]	ptg004732l	31.6	1.54E-29	119	95
WP_038047597.1_sensor_histidine_kinase_KdpD_[Thermoanaerobaculum_aquaticum]	ptg002909l	28.879	1.71E-21	98.2	71
WP_038047001.1_helix-turn-helix_transcriptional_regulator_[Thermoanaerobaculum_aquaticum]	ptg005107l	39.506	5.28E-09	55.8	70
WP_038046275.1_SDR_family_NAD(P)-dependent_oxidoreductase_[Thermoanaerobaculum_aquaticum]	ptg005905l	33.071	2.72E-17	84.7	98
WP_038046130.1_cell_wall_metabolism_sensor_histidine_kinase_WalK_[Thermoanaerobaculum_aquaticum]	ptg005010l	30.112	1.65E-30	129	58
WP_200867106.1_NuoM_family_protein_[Thermoanaerobaculum_aquaticum]	ptg004143l	47.404	4.09E-110	363	89
WP_161685451.1_1-acyl-sn-glycerol-3-phosphate_acyltransferase_[Thermoanaerobaculum_aquaticum]	ptg003708l	36.975	2.43E-18	88.6	43
WP_161685410.1_ABC_transporter_ATP-binding_protein_[Thermoanaerobaculum_aquaticum]	ptg004676l	40	8.81E-54	191	93
WP_161685376.1_16S_rRNA_(uracil(1498)-N(3))-methyltransferase_[Thermoanaerobaculum_aquaticum]	ptg002882l	36.986	1.65E-12	70.1	59
WP_152544069.1_ABC_transporter_ATP-binding_protein_[Thermoanaerobaculum_aquaticum]	ptg004529l	34.911	2.56E-24	104	68
WP_081800126.1_prolyl_oligopeptidase_family_protein_[Thermoanaerobaculum_aquaticum]	ptg003431l	53.001	0	757	96
WP_081799920.1_phosphoribosyltransferase,_partial_[Thermoanaerobaculum_aquaticum]	ptg000492l	26.23	7.3E-10	58.9	95
WP_053335092.1_diguanylate_cyclase_[Thermoanaerobaculum_aquaticum]	LG04	39.61	8.09E-27	115	42
WP_053335057.1_DegT/DnrJ/EryC1/StrS_aminotransferase_family_protein_[Thermoanaerobaculum_aquaticum]	ptg004525l	50.543	2.38E-103	338	97
WP_053335042.1_acetate/propionate_family_kinase_[Thermoanaerobaculum_aquaticum]	ptg004885l	45.623	7.79E-97	321	90
WP_053335012.1_HAD_family_hydrolase_[Thermoanaerobaculum_aquaticum]	ptg004584l	40.816	0.00000667	35.8	92
WP_053334952.1_class_I_adenylate-forming_enzyme_family_protein_[Thermoanaerobaculum_aquaticum]	ptg005087l	27.823	5.95E-29	125	91
WP_038049912.1_class_I_adenylate-forming_enzyme_family_protein_[Thermoanaerobaculum_aquaticum]	ptg005087l	32.576	1.03E-58	214	97
WP_038049560.1_beta-ketoacyl_synthase_[Thermoanaerobaculum_aquaticum]	ptg002723l	42.857	2.28E-49	183	51
WP_038049322.1_RNA_methyltransferase_[Thermoanaerobaculum_aquaticum]	ptg002223l	34.934	2.81E-22	99	87
WP_038049152.1_amino_acid_ABC_transporter_ATP-binding_protein_[Thermoanaerobaculum_aquaticum]	ptg005526l	40.741	5.88E-38	144	98
WP_038048857.1_PLP-dependent_cysteine_synthase_family_protein_[Thermoanaerobaculum_aquaticum]	ptg005554l	45.307	1.89E-63	221	93
WP_038048392.1_diguanylate_cyclase_[Thermoanaerobaculum_aquaticum]	ptg005110l	34.694	1.06E-14	79.7	36
WP_038047381.1_bifunctional_heptose_7-phosphate_kinase/heptose_1-phosphate_adenyltransferase_[Thermoanaerobaculum_aquaticum]	ptg002916l	34.835	5.3E-25	109	97
WP_038047215.1_low_specificity_L-threonine_aldolase_[Thermoanaerobaculum_aquaticum]	LG13	61.538	2.84E-10	65.9	25
WP_038046963.1_class_I_SAM-dependent_RNA_methyltransferase_[Thermoanaerobaculum_aquaticum]	ptg002278l	40.541	3.92E-08	59.7	18
WP_038046884.1_ABC_transporter_ATP-binding_protein_[Thermoanaerobaculum_aquaticum]	ptg003336l	42.009	1.5E-40	152	85
WP_038046366.1_ABC_transporter_ATP-binding_protein_[Thermoanaerobaculum_aquaticum]	ptg002760l	35.417	2.95E-53	129	96
WP_200867153.1_6-phosphofructokinase_[Thermoanaerobaculum_aquaticum]	ptg003223l	48.78	2.78E-96	317	97
WP_081800005.1_molybdenum_ABC_transporter_permease_[Thermoanaerobaculum_aquaticum]	ptg005433l	43.119	1.12E-10	65.5	40

WP_081799833.1_penicillin-binding_protein_1A_[Thermoanaerobaculum_aquaticum]	ptg004863l	32.015	1.54E-106	363	88
WP_053334954.1_acetate--CoA_ligase_family_protein_[Thermoanaerobaculum_aquaticum]	ptg002316l	28.112	2.61E-09	64.7	34
WP_053334898.1_1-acyl-sn-glycerol-3-phosphate_acyltransferase_[Thermoanaerobaculum_aquaticum]	ptg002123l	36.129	4.23E-19	89.7	62
WP_038049142.1_DNA_polymerase_III_subunit_alpha_[Thermoanaerobaculum_aquaticum]	ptg004169l	40.883	0	692	97
WP_038048673.1_HAD_family_hydrolase_[Thermoanaerobaculum_aquaticum]	ptg005448l	37.5	2.98E-17	82	93
WP_038048658.1_exodeoxyribonuclease_V_subunit_beta_[Thermoanaerobaculum_aquaticum]	ptg001995l	25.253	1.62E-17	93.2	40
WP_038048221.1_carbohydrate_ABC_transporter_permease_[Thermoanaerobaculum_aquaticum]	ptg005435l	32.317	1.43E-17	86.7	55
WP_038048211.1_mannose-1-phosphate_guanylyltransferase_[Thermoanaerobaculum_aquaticum]	ptg004242l	36.769	6.92E-61	215	99
WP_038048145.1_NAD(P)-dependent_oxidoreductase_[Thermoanaerobaculum_aquaticum]	ptg006001l	24.286	2.25E-19	92.8	98
WP_038047969.1_molybdopterin_oxidoreductase_family_protein_[Thermoanaerobaculum_aquaticum]	ptg004306l	34.306	1.49E-113	382	95
WP_038047512.1_RHS_repeat_domain-containing_protein_[Thermoanaerobaculum_aquaticum]	ptg004854l	28.351	1.29E-10	63.5	92
WP_038046636.1_lysophospholipid_acyltransferase_family_protein,_partial_[Thermoanaerobaculum_aquaticum]	ptg003171l	30.526	0.00000228	51.2	43
WP_038046380.1_ATP-dependent_helicase_[Thermoanaerobaculum_aquaticum]	ptg005430l	36.828	1.14E-114	384	99
WP_038046244.1_Fur_family_transcriptional_regulator_[Thermoanaerobaculum_aquaticum]	ptg005922l	37.931	1.91E-15	75.5	79
WP_038046128.1_class_I_adenylate-forming_enzyme_family_protein_[Thermoanaerobaculum_aquaticum]	ptg002844l	25.49	2.51E-25	114	71
WP_161685611.1_folate-binding_protein_YgfZ_[Thermoanaerobaculum_aquaticum]	ptg002992l	26.667	3.08E-11	67.4	97
WP_053335189.1_ClpP_family_protease_[Thermoanaerobaculum_aquaticum]	ptg001558l	44.633	3.64E-37	139	99
WP_053335115.1_TrkA_family_potassium_uptake_protein_[Thermoanaerobaculum_aquaticum]	ptg001878l	29.825	3.04E-30	125	67
WP_053334925.1_RluA_family_pseudouridine_synthase_[Thermoanaerobaculum_aquaticum]	ptg003213l	35.484	5.9E-21	95.5	59
WP_038049914.1_3-oxoacyl-ACP_synthase_III_family_protein_[Thermoanaerobaculum_aquaticum]	ptg003871l	29.129	1.69E-24	108	96
WP_038046425.1_menaquinone_biosynthetic_enzyme_MqnA/MqnD_family_protein_[Thermoanaerobaculum_aquaticum]	ptg002867l	33.484	1.53E-27	115	83
WP_038046297.1_HD_family_hydrolase_[Thermoanaerobaculum_aquaticum]	ptg002902l	40.217	1.55E-28	76.3	83
WP_038046180.1_Lon_protease_family_protein_[Thermoanaerobaculum_aquaticum]	ptg003304l	37.657	8.06E-155	504	99
WP_038049965.1_thioredoxin-disulfide_reductase_[Thermoanaerobaculum_aquaticum]	ptg005689l	55.591	2.03E-95	312	99
WP_038048399.1_Re/Si-specific_NAD(P)(+)_transhydrogenase_subunit_alpha_[Thermoanaerobaculum_aquaticum]	ptg004054l	45.345	1.6E-74	254	89
WP_161685559.1_Do_family_serine_endopeptidase_[Thermoanaerobaculum_aquaticum]	ptg004479l	37.188	1.82E-73	256	89
WP_152543991.1_outer_membrane_protein_assembly_factor_BamA_[Thermoanaerobaculum_aquaticum]	ptg005656l	25.904	2.42E-40	165	80
WP_081799965.1_LPS_export_ABC_transporter_ATP-binding_protein_[Thermoanaerobaculum_aquaticum]	ptg005709l	52.101	5.16E-76	253	96
WP_053335180.1_orotidine-5'-phosphate_decarboxylase_[Thermoanaerobaculum_aquaticum]	ptg001025l	50	4E-33	125	88
WP_053335111.1_Fe-S_cluster_assembly_protein_SufD_[Thermoanaerobaculum_aquaticum]	ptg003472l	34.551	5.57E-43	165	71
WP_038050512.1_UDP-N-acetylglucosamine_1-carboxyvinyltransferase_[Thermoanaerobaculum_aquaticum]	ptg005611l	49.758	1.2E-70	166	98

WP_038050295.1_dihydrolipoyl_dehydrogenase_[Thermoanaerobaculum_aquaticum]	ptg003780l	41.88	4.4E-95	318	99
WP_038050217.1_transcription_termination/antitermination_protein_NusG_[Thermoanaerobaculum_aquaticum]	ptg002214l	54.696	4.68E-50	176	99
WP_038049491.1_Fe-S_cluster_assembly_protein_SufB_[Thermoanaerobaculum_aquaticum]	ptg002634l	63.771	0	612	99
WP_038049438.1_GDP-mannose_4,6-dehydratase_[Thermoanaerobaculum_aquaticum]	ptg006001l	56.725	2.24E-122	391	97
WP_038049035.1_GTP_3',8-cyclase_MoaA_[Thermoanaerobaculum_aquaticum]	ptg005933l	40.625	1.03E-56	201	89
WP_038049033.1_cyclic_pyranopterin_monophosphate_synthase_MoaC_[Thermoanaerobaculum_aquaticum]	ptg005976l	47.059	4.22E-22	95.1	84
WP_038048617.1_glutamate-1-semialdehyde_2,1-aminomutase_[Thermoanaerobaculum_aquaticum]	ptg002871l	47.585	1.23E-104	344	96
WP_038048615.1_hydroxymethylbilane_synthase_[Thermoanaerobaculum_aquaticum]	ptg001995l	43.29	6.42E-40	152	76
WP_038048614.1_glutamyl-tRNA_reductase_[Thermoanaerobaculum_aquaticum]	ptg001290l	30.915	3.93E-26	115	73
WP_038048087.1_glutamine--fructose-6-phosphate_transaminase_(isomerizing)_[Thermoanaerobaculum_aquaticum]	ptg002878l	29.299	5.59E-66	238	100
WP_038047297.1_DNA/RNA_nuclease_SfsA_[Thermoanaerobaculum_aquaticum]	ptg003691l	52.308	0.000000709	53.5	27
WP_038046776.1_30S_ribosomal_protein_S3_[Thermoanaerobaculum_aquaticum]	ptg004509l	57.488	5.14E-61	209	96
WP_038046645.1_tRNA_dihydrouridine_synthase_DusB_[Thermoanaerobaculum_aquaticum]	ptg004321l	36.508	1.68E-50	183	94
WP_038046378.1_glutamine--fructose-6-phosphate_transaminase_(isomerizing)_[Thermoanaerobaculum_aquaticum]	ptg002878l	44.444	1.78E-147	475	100
WP_081799966.1_monofunctional_biosynthetic_peptidoglycan_transglycosylase_[Thermoanaerobaculum_aquaticum]	ptg005647l	51.282	2.8E-27	113	48
WP_053335066.1_polynucleotide_adenylyltransferase_PcnB_[Thermoanaerobaculum_aquaticum]	ptg003220l	37.712	1.36E-32	134	53
WP_038047945.1_phosphate_signaling_complex_protein_PhoU_[Thermoanaerobaculum_aquaticum]	ptg004075l	41.206	2.4E-34	133	88
WP_038050367.1_acetyl-CoA_carboxylase_biotin_carboxylase_subunit_[Thermoanaerobaculum_aquaticum]	ptg004240l	55.53	3.55E-142	452	99
WP_038049860.1_23S_rRNA_(adenine(2503)-C(2))-methyltransferase_RlmN_[Thermoanaerobaculum_aquaticum]	ptg002634l	40.761	1.97E-56	202	95
WP_038047744.1_chaperonin_GroEL_[Thermoanaerobaculum_aquaticum]	ptg002031l	61.29	0	616	97
WP_038047295.1_4-hydroxy-3-methylbut-2-enyl_diphosphate_reductase_[Thermoanaerobaculum_aquaticum]	ptg005010l	34.629	5.25E-40	152	95
WP_038046779.1_50S_ribosomal_protein_L16_[Thermoanaerobaculum_aquaticum]	ptg001624l	61.062	6.28E-44	156	82
WP_038046207.1_BPTD_3080_family_restriction_endonuclease_[Thermoanaerobaculum_aquaticum]	ptg003009l	33.093	8.97E-137	456	95
WP_200867123.1_cytochrome_c_oxidase_subunit_II_[Thermoanaerobaculum_aquaticum]	ptg002453l	41.987	1.14E-60	214	87
WP_081800168.1_carboxylating_nicotinate-nucleotide_diphosphorylase,_partial_[Thermoanaerobaculum_aquaticum]	ptg004584l	46.403	1.26E-59	207	99
WP_081800145.1_30S_ribosomal_protein_S2_[Thermoanaerobaculum_aquaticum]	ptg004895l	54.299	1.93E-77	258	81
WP_053335217.1_NADH-quinone_oxidoreductase_subunit_NuoF_[Thermoanaerobaculum_aquaticum]	ptg005451l	45.052	4.81E-95	317	88
WP_053335098.1_bifunctional_phosphopantothenoylcysteine_decarboxylase/phosphopantothenate--cysteine_ligase_CoaBC_[Thermoanaerobaculum_aquaticum]	ptg002199l	37.222	4.57E-58	208	89
WP_053335018.1_DHA2_family_efflux_MFS_transporter_permease_subunit_[Thermoanaerobaculum_aquaticum]	ptg002902l	29.146	4.31E-24	75.5	55
WP_053334985.1_efflux_RND_transporter_periplasmic_adaptor_subunit_[Thermoanaerobaculum_aquaticum]	ptg001766l	29.647	3.96E-45	174	83
WP_053334970.1_phosphoenolpyruvate--protein_phosphotransferase_[Thermoanaerobaculum_aquaticum]	ptg002723l	35.395	5.39E-101	340	98

WP_038050456.1_imidazole_glycerol_phosphate_synthase_subunit_HisH_[Thermoanaerobaculum_aquaticum]	ptg004372l	31.795	1.21E-21	95.5	97
WP_038050225.1_30S_ribosomal_protein_S7_[Thermoanaerobaculum_aquaticum]	ptg003722l	47.742	3.52E-46	164	99
WP_038050224.1_30S_ribosomal_protein_S12_[Thermoanaerobaculum_aquaticum]	ptg003722l	78.632	5.09E-56	191	88
WP_038049959.1_phosphoribosylamine--glycine_ligase_[Thermoanaerobaculum_aquaticum]	ptg002175l	44.104	4.98E-96	319	98
WP_038049786.1_L-glutamate_gamma-semialdehyde_dehydrogenase_[Thermoanaerobaculum_aquaticum]	ptg004321l	28.14	1.26E-32	137	77
WP_038049504.1_thioredoxin_[Thermoanaerobaculum_aquaticum]	ptg003762l	50	1.33E-30	117	97
WP_038048828.1_dTDP-glucose_4,6-dehydratase_[Thermoanaerobaculum_aquaticum]	ptg001690l	44.476	5.49E-90	298	96
WP_038047444.1_amidophosphoribosyltransferase_[Thermoanaerobaculum_aquaticum]	ptg000484l	37.815	3.27E-78	269	97
WP_038046991.1_50S_ribosomal_protein_L22_[Thermoanaerobaculum_aquaticum]	ptg004509l	46.364	1.1E-19	86.3	97
WP_038046966.1_DNA_internalization-related_competence_protein_ComEC/Rec2_[Thermoanaerobaculum_aquaticum]	ptg005976l	27.969	1.6E-15	85.5	33
WP_038046934.1_3-phosphoshikimate_1-carboxyvinyltransferase_[Thermoanaerobaculum_aquaticum]	ptg002123l	33.415	2.97E-55	201	94
WP_038046808.1_preprotein_translocase_subunit_SecY_[Thermoanaerobaculum_aquaticum]	ptg004509l	44.69	6.51E-111	363	97
WP_038046806.1_50S_ribosomal_protein_L15_[Thermoanaerobaculum_aquaticum]	ptg005891l	55.385	1.05E-32	125	87
WP_038046804.1_30S_ribosomal_protein_S5_[Thermoanaerobaculum_aquaticum]	ptg004509l	56.489	6.65E-39	144	74
WP_038046788.1_50S_ribosomal_protein_L14_[Thermoanaerobaculum_aquaticum]	ptg004509l	61.789	6.01E-44	155	100
WP_038046773.1_30S_ribosomal_protein_S19_[Thermoanaerobaculum_aquaticum]	ptg004509l	60	2.44E-32	121	96
WP_038046419.1_trigger_factor_[Thermoanaerobaculum_aquaticum]	ptg005527l	29.24	2.11E-12	73.2	41
WP_038046284.1_ATP-dependent_protease_subunit_HslV_[Thermoanaerobaculum_aquaticum]	ptg002928l	55.172	1.21E-46	166	98
WP_161685563.1_sulfotransferase_domain-containing_protein,_partial_[Thermoanaerobaculum_aquaticum]	ptg005259l	33.333	6.54E-10	62.4	49
WP_038046282.1_ATP-dependent_protease_ATPase_subunit_HslU_[Thermoanaerobaculum_aquaticum]	ptg005430l	50.885	1.11E-126	407	100
WP_200867098.1_type_I_methionyl_aminopeptidase_[Thermoanaerobaculum_aquaticum]	ptg002436l	47.154	3.64E-73	245	98
WP_053335112.1_SufS_family_cysteine_desulfurase_[Thermoanaerobaculum_aquaticum]	ptg005671l	46.684	1.17E-113	369	94
WP_053334986.1_heavy_metal_translocating_P-type_ATPase_[Thermoanaerobaculum_aquaticum]	ptg003880l	37.037	1.65E-75	271	76
WP_053334752.1_peptidase_T_[Thermoanaerobaculum_aquaticum]	ptg001690l	43.029	2.27E-110	360	98
WP_038050382.1_protein_translocase_subunit_SecD_[Thermoanaerobaculum_aquaticum]	ptg005746l	38.664	6.04E-79	273	92
WP_038050381.1_protein_translocase_subunit_SecF_[Thermoanaerobaculum_aquaticum]	ptg004256l	47.305	4.3E-43	164	43
WP_038049890.1_arginase_[Thermoanaerobaculum_aquaticum]	LG29	54.545	2.95E-12	70.9	74
WP_038049414.1_signal_recognition_particle-docking_protein_FtsY_[Thermoanaerobaculum_aquaticum]	ptg004885l	48.361	1.16E-44	165	82
WP_038049284.1_transcriptional_regulator_NrdR_[Thermoanaerobaculum_aquaticum]	ptg004151l	44.218	5.96E-35	131	96
WP_038047855.1_gamma-glutamyltransferase_[Thermoanaerobaculum_aquaticum]	ptg001595l	56.261	0	593	97
WP_038047668.1_tRNA_epoxyqueuosine(34)_reductase_QueG_[Thermoanaerobaculum_aquaticum]	ptg005839l	39.024	1.93E-51	186	90
WP_038046356.1_signal_peptidase_I_[Thermoanaerobaculum_aquaticum]	ptg002902l	32.203	1.31E-17	75.9	77
WP_235208674.1_aspartate_1-decarboxylase_[Thermoanaerobaculum_aquaticum]	ptg004018l	50.82	1.61E-29	115	95

WP_038046944.1_alanine_racemase_[Thermoanaerobaculum_aquaticum]	ptg004959l	29.282	9E-37	145	90
WP_235208684.1_UDP-N-acetylmuramoyl-L-alanine--D-glutamate_ligase_[Thermoanaerobaculum_aquaticum]	ptg003536l	27.654	5.03E-08	59.7	70
WP_235208679.1_ribosome_small_subunit-dependent_GTPase_A,_partial_[Thermoanaerobaculum_aquaticum]	ptg004147l	41.633	6.21E-53	189	84
WP_200867105.1_phosphoribosylglycinamide_formyltransferase_[Thermoanaerobaculum_aquaticum]	LG04	41.872	6.63E-37	139	95
WP_053335147.1_dephospho-CoA_kinase_[Thermoanaerobaculum_aquaticum]	ptg002909l	30.653	2.08E-10	63.5	89
WP_053334995.1_(d)CMP_kinase_[Thermoanaerobaculum_aquaticum]	ptg002942l	43.367	1.63E-40	151	81
WP_053334980.1_oxygen-independent_coproporphyrinogen_III_oxidase_[Thermoanaerobaculum_aquaticum]	ptg003548l	39.13	7.82E-108	355	98
WP_038050459.1_ATP_phosphoribosyltransferase_[Thermoanaerobaculum_aquaticum]	ptg005709l	29.717	1.11E-22	99	99
WP_038050379.1_50S_ribosomal_protein_L28_[Thermoanaerobaculum_aquaticum]	ptg003250l	38.462	7.63E-11	58.5	100
WP_038050222.1_50S_ribosomal_protein_L7/L12_[Thermoanaerobaculum_aquaticum]	ptg002214l	59.223	2.51E-18	83.2	80
WP_038050040.1_cysteine--tRNA_ligase_[Thermoanaerobaculum_aquaticum]	ptg002844l	40.385	7.41E-93	311	98
WP_038049989.1_magnesium/cobalt_transporter_CorA_[Thermoanaerobaculum_aquaticum]	ptg003762l	35.644	2.89E-13	74.7	29
WP_038049958.1_5-(carboxyamino)imidazole_ribonucleotide_mutase_[Thermoanaerobaculum_aquaticum]	ptg002871l	48.039	1.81E-15	76.3	62
WP_038049770.1_preQ(1)_synthase_[Thermoanaerobaculum_aquaticum]	ptg003420l	46.847	3.47E-28	111	82
WP_038049511.1_catalase/peroxidase_HPI_[Thermoanaerobaculum_aquaticum]	ptg003304l	61.326	0	905	98
WP_038049472.1_lysine--tRNA_ligase_[Thermoanaerobaculum_aquaticum]	ptg004479l	48.104	3.99E-135	434	98
WP_038049409.1_radical_SAM_family_heme_chaperone_HemW_[Thermoanaerobaculum_aquaticum]	ptg003090l	33.945	6.94E-46	172	87
WP_038049148.1_DNA_mismatch_repair_endonuclease_MutL_[Thermoanaerobaculum_aquaticum]	ptg004564l	39.306	1.09E-57	212	94
WP_038048746.1_methionine_adenosyltransferase_[Thermoanaerobaculum_aquaticum]	ptg005611l	53.865	1.38E-129	415	97
WP_038048740.1_30S_ribosomal_protein_S12_methylthiotransferase_RimO_[Thermoanaerobaculum_aquaticum]	ptg004147l	38.391	1.26E-82	282	92
WP_038048523.1_glutamine-hydrolyzing_carbamoyl-phosphate_synthase_small_subunit_[Thermoanaerobaculum_aquaticum]	ptg001693l	40.741	5.29E-70	241	99
WP_038048170.1_redox-regulated_ATPase_YchF_[Thermoanaerobaculum_aquaticum]	ptg002928l	38.63	7.05E-70	241	99
WP_038048139.1_threonine--tRNA_ligase_[Thermoanaerobaculum_aquaticum]	ptg004758l	43.711	3.61E-169	539	97
WP_038048137.1_50S_ribosomal_protein_L35_[Thermoanaerobaculum_aquaticum]	ptg001925l	50	2.09E-10	57.4	94
WP_038047772.1_class_II_fructose-bisphosphatase_[Thermoanaerobaculum_aquaticum]	ptg004284l	46.377	4.43E-56	199	84
WP_038047692.1_signal_recognition_particle_protein_[Thermoanaerobaculum_aquaticum]	ptg002916l	47.209	6.25E-95	317	97
WP_038047658.1_IMP_dehydrogenase_[Thermoanaerobaculum_aquaticum]	ptg002610l	57.143	4.4E-174	546	99
WP_038047379.1_adenylyltransferase/cytidyltransferase_family_protein_[Thermoanaerobaculum_aquaticum]	ptg002916l	43.333	6.83E-30	117	90
WP_038047093.1_30S_ribosomal_protein_S15_[Thermoanaerobaculum_aquaticum]	ptg004026l	46.429	5.95E-21	88.6	92
WP_038046953.1_3-dehydroquinate_synthase_[Thermoanaerobaculum_aquaticum]	ptg003363l	43.043	5.41E-43	163	61
WP_038046800.1_50S_ribosomal_protein_L6_[Thermoanaerobaculum_aquaticum]	ptg001624l	44.134	1.71E-37	140	99
WP_038046530.1_30S_ribosomal_protein_S21_[Thermoanaerobaculum_aquaticum]	ptg003583l	52.632	0.000007	45.1	54

WP_038046402.1_ATP-dependent_chaperone_ClpB_[Thermoanaerobaculum_aquaticum]	ptg002304l	52.602	0	778	99
WP_038046246.1_ferrous_iron_transport_protein_B_[Thermoanaerobaculum_aquaticum]	ptg003831l	26.667	1.75E-36	151	83
WP_235208677.1_type_I_glyceraldehyde-3-phosphate_dehydrogenase_[Thermoanaerobaculum_aquaticum]	ptg002223l	53.211	2.93E-103	337	91
WP_081799919.1_ATP-dependent_zinc_metalloprotease_FtsH_[Thermoanaerobaculum_aquaticum]	ptg003039l	54.709	1.53E-154	496	79
WP_081799852.1_polyribonucleotide_nucleotidyltransferase_[Thermoanaerobaculum_aquaticum]	ptg004026l	48.088	0	649	93
WP_081799839.1_selenide,_water_dikinase_SelD_[Thermoanaerobaculum_aquaticum]	ptg001871l	34.925	1.27E-44	167	95
WP_081799803.1_recombination_mediator_RecR_[Thermoanaerobaculum_aquaticum]	ptg003168l	45.833	4.32E-49	174	96
WP_053335024.1_heme_o_synthase_[Thermoanaerobaculum_aquaticum]	LG06	49.479	1.83E-41	157	60
WP_053335001.1_cytochrome_c_oxidase_subunit_I_[Thermoanaerobaculum_aquaticum]	ptg001690l	65.038	0	633	96
WP_053334738.1_cell_division_protein_FtsZ_[Thermoanaerobaculum_aquaticum]	ptg004510l	48.966	1.68E-54	197	74
WP_053334716.1_DNA_polymerase_III_subunit_gamma/tau_[Thermoanaerobaculum_aquaticum]	ptg002760l	47.464	9.88E-79	272	55
WP_053334713.1_bacillithiol_biosynthesis_deacetylase_BshB1_[Thermoanaerobaculum_aquaticum]	ptg005204l	30.942	6.04E-14	74.7	90
WP_038050499.1_aconitate_hydratase_[Thermoanaerobaculum_aquaticum]	ptg002123l	30.945	6.93E-23	109	50
WP_038050427.1_50S_ribosomal_protein_L13_[Thermoanaerobaculum_aquaticum]	ptg000819l	56.693	1.54E-42	152	88
WP_038050421.1_UMP_kinase_[Thermoanaerobaculum_aquaticum]	ptg002436l	53.39	5.4E-67	228	96
WP_038050357.1_3-oxoacyl-[acyl-carrier-protein]_reductase_[Thermoanaerobaculum_aquaticum]	ptg005905l	45.935	9.47E-61	209	99
WP_038050231.1_elongation_factor_G_[Thermoanaerobaculum_aquaticum]	ptg004438l	46.328	2.89E-178	568	99
WP_038050218.1_50S_ribosomal_protein_L11_[Thermoanaerobaculum_aquaticum]	ptg002214l	66.429	1.06E-56	193	99
WP_038049501.1_16S_rRNA_(adenine(1518)-N(6)/adenine(1519)-N(6))-dimethyltransferase_RsmA_[Thermoanaerobaculum_aquaticum]	ptg004306l	33.533	4.99E-18	87	61
WP_038049418.1_guanylate_kinase_[Thermoanaerobaculum_aquaticum]	ptg004759l	36.842	6.56E-32	125	88
WP_038049055.1_rod_shape-determining_protein_RodA_[Thermoanaerobaculum_aquaticum]	ptg004510l	31.466	7.33E-19	92	64
WP_038048884.1_transcription_antitermination_factor_NusB_[Thermoanaerobaculum_aquaticum]	ptg004564l	46.281	1.07E-24	101	86
WP_038048758.1_glycine_cleavage_system_protein_GcvH_[Thermoanaerobaculum_aquaticum]	ptg005318l	47.581	1.21E-31	120	95
WP_038048402.1_galactose-1-phosphate_uridylyltransferase_[Thermoanaerobaculum_aquaticum]	ptg003785l	31.549	5.18E-48	177	97
WP_038048126.1_phosphopyruvate_hydratase_[Thermoanaerobaculum_aquaticum]	ptg004466l	57.009	4.39E-141	449	99
WP_038048015.1_molecular_chaperone_DnaJ_[Thermoanaerobaculum_aquaticum]	ptg003680l	47.268	3.76E-71	245	98
WP_038047527.1_pyruvate,_phosphate_dikinase_[Thermoanaerobaculum_aquaticum]	ptg005844l	54.686	0	949	98
WP_038047233.1_dTMP_kinase_[Thermoanaerobaculum_aquaticum]	ptg003947l	36.364	5.37E-36	137	94
WP_038047209.1_histidine--tRNA_ligase_[Thermoanaerobaculum_aquaticum]	ptg003703l	40.594	2.58E-87	293	94
WP_038047206.1_deoxyribose-phosphate_aldolase_[Thermoanaerobaculum_aquaticum]	ptg005596l	34.146	1.58E-10	63.9	82
WP_038047080.1_30S_ribosome-binding_factor_RbfA_[Thermoanaerobaculum_aquaticum]	ptg001690l	35.398	9.61E-18	81.3	92
WP_038047077.1_translation_initiation_factor_IF-2_[Thermoanaerobaculum_aquaticum]	ptg001690l	45.44	2.6E-147	486	65

WP_038046820.1_50S_ribosomal_protein_L17_[Thermoanaerobaculum_aquaticum]	ptg004509l	51.724	8.46E-32	122	83
WP_038046802.1_50S_ribosomal_protein_L18_[Thermoanaerobaculum_aquaticum]	ptg005254l	52.525	2.03E-27	108	81
WP_038046781.1_50S_ribosomal_protein_L29_[Thermoanaerobaculum_aquaticum]	ptg004509l	46.667	4.54E-10	56.6	92
WP_038046655.1_50S_ribosomal_protein_L9_[Thermoanaerobaculum_aquaticum]	ptg005487l	36.301	3.79E-21	92	95
WP_038046342.1_N-acetyl-alpha-D-glucosaminyl_L-malate_synthase_BshA_[Thermoanaerobaculum_aquaticum]	ptg001909l	26.948	3.7E-19	93.2	80
WP_038046223.1_fructose-6-phosphate_aldolase_[Thermoanaerobaculum_aquaticum]	ptg002573l	27.615	1.22E-10	63.9	89
WP_038046174.1_CDP-diacylglycerol--glycerol-3-phosphate_3-phosphatidyltransferase_[Thermoanaerobaculum_aquaticum]	ptg005527l	34.078	7.5E-17	80.9	96
WP_152543888.1_peptide_chain_release_factor_2_[Thermoanaerobaculum_aquaticum]	ptg004479l	53.257	1.13E-78	266	71
WP_081799841.1_50S_ribosomal_protein_L36_[Thermoanaerobaculum_aquaticum]	ptg004564l	72.973	2.45E-11	58.5	100
WP_038046790.1_50S_ribosomal_protein_L24_[Thermoanaerobaculum_aquaticum]	ptg004509l	50.485	2.78E-27	107	95
WP_152543825.1_ribonuclease_PH_[Thermoanaerobaculum_aquaticum]	ptg003928l	51.471	9.88E-44	137	77
WP_053334905.1_phenylalanine--tRNA_ligase_subunit_beta_[Thermoanaerobaculum_aquaticum]	ptg001925l	34.961	3.22E-89	309	91
WP_038050220.1_50S_ribosomal_protein_L1_[Thermoanaerobaculum_aquaticum]	ptg002723l	53.604	6.78E-66	224	97
WP_038046226.1_DNA_topoisomerase_(ATP-hydrolyzing)_subunit_B_[Thermoanaerobaculum_aquaticum]	ptg000372l	60.634	0	564	99
WP_327138679.1_phosphate_ABC_transporter_ATP-binding_protein_PstB_[Thermoanaerobaculum_aquaticum]	ptg002567l	55.823	8.92E-90	293	98
WP_161685279.1_primosomal_protein_N',_partial_[Thermoanaerobaculum_aquaticum]	ptg004114l	40	4.36E-70	242	99
WP_161685270.1_ribonuclease_R_[Thermoanaerobaculum_aquaticum]	ptg004242l	34.644	1.48E-83	295	85
WP_161685241.1_tyrosine--tRNA_ligase_[Thermoanaerobaculum_aquaticum]	ptg000819l	51	1.06E-134	429	98
WP_081800070.1_tRNA_pseudouridine(38-40)_synthase_TruA_[Thermoanaerobaculum_aquaticum]	ptg001238l	34.8	9.47E-39	147	92
WP_081799810.1_transcription_termination_factor_Rho_[Thermoanaerobaculum_aquaticum]	ptg003762l	65.823	1.56E-176	552	85
WP_053335277.1_ribonuclease_III_[Thermoanaerobaculum_aquaticum]	ptg003583l	47.059	2.37E-09	60.8	28
WP_053335220.1_DNA-directed_RNA_polymerase_subunit_beta'_[Thermoanaerobaculum_aquaticum]	ptg002723l	51.608	0	1315	97
WP_053334903.1_transcription-repair_coupling_factor_[Thermoanaerobaculum_aquaticum]	ptg004785l	42.278	0	614	81
KAB2958905.1_MAG:_hypothetical_protein_F9K16_12870_[Thermoanaerobaculia_bacterium]	ptg002199l	39.535	1.61E-20	91.7	70
KAB2958895.1_MAG:_ACP_S-malonyltransferase,_partial_[Thermoanaerobaculia_bacterium]	ptg003980l	43.443	1.4E-22	95.9	83
KAB2958894.1_MAG:_NADH-quinone_oxidoreductase_subunit_I_[Thermoanaerobaculia_bacterium]	ptg004143l	32.727	7.49E-19	87.4	85
KAB2958892.1_MAG:_NADH-quinone_oxidoreductase_subunit_B_[Thermoanaerobaculia_bacterium]	ptg004143l	48.322	1.72E-49	174	89
KAB2958891.1_MAG:_NADH-quinone_oxidoreductase_subunit_D_[Thermoanaerobaculia_bacterium]	ptg004143l	39.798	4.71E-85	293	91
KAB2958890.1_MAG:_glycosyltransferase,_partial_[Thermoanaerobaculia_bacterium]	ptg004523l	35.814	1.99E-16	86.3	45
KAB2958843.1_MAG:_type_IV_pilus_secretin_PilQ,_partial_[Thermoanaerobaculia_bacterium]	ptg004863l	32.104	5.54E-55	206	65
KAB2958838.1_MAG:_insulinase_family_protein_[Thermoanaerobaculia_bacterium]	ptg005195l	29.808	1.07E-24	113	66
KAB2958784.1_MAG:_carbamoyl-phosphate_synthase_large_subunit_[Thermoanaerobaculia_bacterium]	ptg002303l	54.132	0	1162	99

KAB2958782.1_MAG:_amidase,_partial_[Thermoanaerobaculia_bacterium]	ptg003250l	29.124	2.63E-33	139	95
KAB2958781.1_MAG:_sulfatase_[Thermoanaerobaculia_bacterium]	ptg000693l	26.062	7.78E-14	79.7	42
KAB2958776.1_MAG:_4-hydroxybenzoate_octaprenyltransferase_[Thermoanaerobaculia_bacterium]	ptg001571l	44.898	4.94E-38	146	93
KAB2958773.1_MAG:_NADP-dependent_isocitrate_dehydrogenase_[Thermoanaerobaculia_bacterium]	ptg002573l	37.226	1.75E-10	67.4	31
KAB2958772.1_MAG:_S9_family_peptidase_[Thermoanaerobaculia_bacterium]	ptg002902l	46.078	5.81E-120	295	91
KAB2958771.1_MAG:_aspartate_ammonia-lyase,_partial_[Thermoanaerobaculia_bacterium]	ptg004500l	49.321	3.02E-56	196	92
KAB2958735.1_MAG:_alcohol_dehydrogenase_catalytic_domain-containing_protein_[Thermoanaerobaculia_bacterium]	ptg001784l	38.889	4.65E-10	61.2	50
KAB2958731.1_MAG:_sensor_histidine_kinase_[Thermoanaerobaculia_bacterium]	ptg003548l	32.461	1.36E-15	82	47
KAB2958730.1_MAG:_response_regulator_transcription_factor_[Thermoanaerobaculia_bacterium]	ptg003829l	31.579	2.04E-19	90.9	98
KAB2958726.1_MAG:_phosphoribosylformylglycinamidine_cyclo-ligase_[Thermoanaerobaculia_bacterium]	ptg005227l	42.012	1.27E-77	263	94
KAB2958725.1_MAG:_xanthine_dehydrogenase_family_protein,_partial_[Thermoanaerobaculia_bacterium]	ptg005556l	32.95	2.76E-28	122	59
KAB2958724.1_MAG:_NAD(+)/NADH_kinase_[Thermoanaerobaculia_bacterium]	ptg002867l	37.991	4.34E-30	122	81
KAB2958722.1_MAG:_TlyA_family_RNA_methyltransferase,_partial_[Thermoanaerobaculia_bacterium]	ptg004075l	42.623	1.73E-29	118	87
KAB2958680.1_MAG:_protoporphyrinogen_oxidase,_partial_[Thermoanaerobaculia_bacterium]	ptg004501l	31.176	6.11E-17	83.2	69
KAB2958679.1_MAG:_oxygen-independent_coproporphyrinogen_III_oxidase_[Thermoanaerobaculia_bacterium]	ptg003548l	38.116	6.42E-103	340	98
KAB2958678.1_MAG:_uroporphyrinogen_decarboxylase_[Thermoanaerobaculia_bacterium]	ptg005545l	40.708	4.78E-80	270	94
KAB2958677.1_MAG:_porphobilinogen_synthase_[Thermoanaerobaculia_bacterium]	ptg004997l	51.057	5.47E-85	283	99
KAB2958675.1_MAG:_nuclear_transport_factor_2_family_protein_[Thermoanaerobaculia_bacterium]	ptg003213l	34.579	0.0000052	49.7	54
KAB2958673.1_MAG:_fumarate_hydratase_[Thermoanaerobaculia_bacterium]	LG24	66.154	2.97E-18	93.2	68
KAB2958672.1_MAG:_TonB-dependent_receptor_[Thermoanaerobaculia_bacterium]	ptg001925l	26.667	1.19E-09	66.6	31
KAB2958671.1_MAG:_hypothetical_protein_F9K16_13080,_partial_[Thermoanaerobaculia_bacterium]	ptg000619l	32.698	1.85E-40	159	79
KAB2958670.1_MAG:_serine/threonine-protein_phosphatase_[Thermoanaerobaculia_bacterium]	ptg003137l	29.87	5.84E-21	97.1	74
KAB2958669.1_MAG:_molybdopterin-dependent_oxidoreductase,_partial_[Thermoanaerobaculia_bacterium]	ptg003787l	27.559	5.18E-36	147	86
KAB2958628.1_MAG:_ribulose-phosphate_3-epimerase_[Thermoanaerobaculia_bacterium]	ptg004771l	45.876	1.96E-37	141	87
KAB2958619.1_MAG:_S1_RNA-binding_domain-containing_protein,_partial_[Thermoanaerobaculia_bacterium]	ptg001025l	33.333	9.37E-27	114	95
KAB2958618.1_MAG:_M23_family_metallopeptidase_[Thermoanaerobaculia_bacterium]	ptg004510l	38.298	1.42E-10	67.8	20
KAB2958616.1_MAG:_cytochrome_C_oxidase_subunit_I_[Thermoanaerobaculia_bacterium]	ptg002453l	28.409	0.00000136	55.5	30
KAB2958596.1_MAG:_AAA_domain-containing_protein,_partial_[Thermoanaerobaculia_bacterium]	ptg005150l	46.332	1.28E-49	184	63
KAB2958595.1_MAG:_ATP-binding_protein_[Thermoanaerobaculia_bacterium]	ptg002742l	32.432	1.53E-09	58.9	72
KAB2958593.1_MAG:_MBL_fold_metallo-hydrolase_[Thermoanaerobaculia_bacterium]	ptg002871l	31.923	1.42E-25	109	94
KAB2958589.1_MAG:_OmpA_family_protein_[Thermoanaerobaculia_bacterium]	ptg004509l	38.462	7.57E-11	64.3	37
KAB2958587.1_MAG:_ATP-dependent_protease,_partial_[Thermoanaerobaculia_bacterium]	ptg004017l	45.848	4.84E-55	196	91
KAB2958584.1_MAG:_ABC_transporter_permease_[Thermoanaerobaculia_bacterium]	ptg003548l	46.639	1.9E-42	159	82

KAB2958583.1_MAG:_ABC_transporter_permease_[Thermoanaerobaculia_bacterium]	ptg005227l	35.789	1.84E-47	174	92
KAB2958558.1_MAG:_MBL_fold_metallo-hydrolase_[Thermoanaerobaculia_bacterium]	ptg001658l	44.841	3.91E-67	228	99
KAB2958556.1_MAG:_hypothetical_protein_F9K18_12025,_partial_[Thermoanaerobaculia_bacterium]	ptg002123l	37.681	2.09E-28	119	65
KAB2958553.1_MAG:_ABC_transporter_ATP-binding_protein_[Thermoanaerobaculia_bacterium]	ptg001604l	36.304	3.66E-58	205	95
KAB2958551.1_MAG:_ABC_transporter_ATP-binding_protein_[Thermoanaerobaculia_bacterium]	ptg004724l	35.974	3.46E-43	162	96
KAB2958534.1_MAG:_ATP-binding_cassette_domain-containing_protein,_partial_[Thermoanaerobaculia_bacterium]	LG14	50.877	0.000000331	50.1	55
KAB2958532.1_MAG:_potassium_transporter_Kup_[Thermoanaerobaculia_bacterium]	ptg005550l	47.516	6.53E-134	290	90
KAB2958530.1_MAG:_response_regulator,_partial_[Thermoanaerobaculia_bacterium]	ptg001909l	34.677	4.16E-14	71.6	86
KAB2958528.1_MAG:_AMP-binding_protein,_partial_[Thermoanaerobaculia_bacterium]	LG03	35.641	1.53E-71	248	91
KAB2958527.1_MAG:_aminotransferase_class_I/II-fold_pyridoxal_phosphate-dependent_enzyme_[Thermoanaerobaculia_bacterium]	ptg004905l	25.074	6.02E-18	90.5	73
KAB2958526.1_MAG:_lysine_2,3-aminomutase_[Thermoanaerobaculia_bacterium]	LG18	56.818	6.84E-25	112	62
KAB2958501.1_MAG:_S41_family_peptidase_[Thermoanaerobaculia_bacterium]	ptg002534l	40.391	1.93E-48	184	57
KAB2958500.1_MAG:_histone_deacetylase,_partial_[Thermoanaerobaculia_bacterium]	ptg005274l	28.161	2.27E-12	68.9	84
KAB2958496.1_MAG:_amidohydrolase_[Thermoanaerobaculia_bacterium]	ptg002610l	55.014	4.2E-117	356	86
KAB2958482.1_MAG:_glycosyltransferase_[Thermoanaerobaculia_bacterium]	ptg001909l	36.022	7.89E-17	84	64
KAB2958481.1_MAG:_ATP-dependent_DNA_helicase_RecG_[Thermoanaerobaculia_bacterium]	ptg001995l	50.633	9.26E-87	302	54
KAB2958478.1_MAG:_acetyl-CoA_C-acyltransferase,_partial_[Thermoanaerobaculia_bacterium]	ptg002612l	45.143	1.51E-76	261	91
KAB2958477.1_MAG:_gamma-glutamyl-gamma-aminobutyrate_hydrolase_family_protein_[Thermoanaerobaculia_bacterium]	ptg005309l	30.579	2.34E-13	53.9	81
KAB2958476.1_MAG:_enoyl-CoA_hydratase/isomerase_family_protein,_partial_[Thermoanaerobaculia_bacterium]	ptg002612l	32.143	1.14E-16	80.9	97
KAB2958475.1_MAG:_GGDEF_domain-containing_protein,_partial_[Thermoanaerobaculia_bacterium]	ptg003536l	38.298	1.58E-19	94.4	37
KAB2958474.1_MAG:_PhoH_family_protein_[Thermoanaerobaculia_bacterium]	ptg005772l	35.664	7.77E-22	102	32
KAB2958461.1_MAG:_fumarylacetoacetate_hydrolase_family_protein_[Thermoanaerobaculia_bacterium]	ptg003204l	38.202	2.34E-26	111	68
KAB2958458.1_MAG:_NUDIX_hydrolase_[Thermoanaerobaculia_bacterium]	ptg003498l	39.062	1.16E-08	57	72
KAB2958448.1_MAG:_fatty_acid_oxidation_complex_subunit_alpha_FadJ,_partial_[Thermoanaerobaculia_bacterium]	ptg005527l	38.328	8.32E-53	196	56
KAB2958447.1_MAG:_Stp1/IreP_family_PP2C-type_Ser/Thr_phosphatase_[Thermoanaerobaculia_bacterium]	ptg003137l	38.76	1.14E-40	153	90
KAB2958446.1_MAG:_DnaJ_domain-containing_protein_[Thermoanaerobaculia_bacterium]	ptg005113l	43.478	2.37E-10	63.2	31
KAB2958444.1_MAG:_hypothetical_protein_F9K18_12135_[Thermoanaerobaculia_bacterium]	LG11	29.911	0.00000173	55.8	25
KAB2958442.1_MAG:_pyridoxal_phosphate-dependent_aminotransferase,_partial_[Thermoanaerobaculia_bacterium]	ptg004905l	30.838	5.46E-36	144	82
KAB2958440.1_MAG:_tyrosine--tRNA_ligase_[Thermoanaerobaculia_bacterium]	ptg000819l	48.642	4.44E-108	353	98
KAB2958432.1_MAG:_hypothetical_protein_F9K16_13385_[Thermoanaerobaculia_bacterium]	ptg006050l	40	1.68E-09	65.9	9
KAB2958425.1_MAG:_alpha/beta_fold_hydrolase_[Thermoanaerobaculia_bacterium]	ptg005416l	29.348	1.36E-18	89	95
KAB2958424.1_MAG:_NAD(P)H-binding_protein,_partial_[Thermoanaerobaculia_bacterium]	ptg003220l	46.154	4.34E-08	50.8	85
KAB2958423.1_MAG:_GTP_3',8-cyclase_MoaA_[Thermoanaerobaculia_bacterium]	ptg005933l	43.175	6.3E-58	205	93

KAB2958422.1_MAG:_M28_family_peptidase,_partial_[Thermoanaerobaculia_bacterium]	ptg001897l	40	0.000000281	57.4	15
KAB2958404.1_MAG:_response_regulator_transcription_factor_[Thermoanaerobaculia_bacterium]	ptg002505l	41.304	1.27E-41	154	96
KAB2958396.1_MAG:_hypothetical_protein_F9K18_12180_[Thermoanaerobaculia_bacterium]	ptg002660l	26.69	0.000000111	58.5	60
KAB2958395.1_MAG:_rRNA_pseudouridine_synthase,_partial_[Thermoanaerobaculia_bacterium]	ptg004652l	41.102	1.54E-48	176	85
KAB2958394.1_MAG:_SMC-Scp_complex_subunit_ScpB_[Thermoanaerobaculia_bacterium]	ptg004651l	50.667	9.08E-17	63.5	52
KAB2958393.1_MAG:_hypothetical_protein_F9K16_13465_[Thermoanaerobaculia_bacterium]	ptg003247l	37.383	1.16E-16	83.2	40
KAB2958392.1_MAG:_tryptophan--tRNA_ligase_[Thermoanaerobaculia_bacterium]	ptg003327l	37.082	6.82E-56	199	96
KAB2958382.1_MAG:_hypothetical_protein_F9K16_13490_[Thermoanaerobaculia_bacterium]	ptg003592l	33.333	0.000000431	53.1	51
KAB2958381.1_MAG:_NADP-dependent_malic_enzyme_[Thermoanaerobaculia_bacterium]	ptg005092l	45.106	0	595	99
KAB2958372.1_MAG:_NAD(P)-dependent_oxidoreductase_[Thermoanaerobaculia_bacterium]	ptg005030l	32.184	1.3E-16	83.2	91
KAB2958357.1_MAG:_dienelactone_hydrolase_family_protein,_partial_[Thermoanaerobaculia_bacterium]	ptg004926l	32.143	7.98E-16	79.3	95
KAB2958356.1_MAG:_tetratricopeptide_repeat_protein,_partial_[Thermoanaerobaculia_bacterium]	ptg006047l	32.059	8.13E-30	127	68
KAB2958355.1_MAG:_agmatine_deiminase_family_protein,_partial_[Thermoanaerobaculia_bacterium]	ptg001693l	25.18	8.63E-14	77	66
KAB2958349.1_MAG:_EVE_domain-containing_protein_[Thermoanaerobaculia_bacterium]	ptg003831l	33.094	0.000000553	50.8	88
KAB2958341.1_MAG:_type_4a_pilus_biogenesis_protein_PilO_[Thermoanaerobaculia_bacterium]	ptg004771l	30.769	0.00000227	50.8	56
KAB2958339.1_MAG:_type_IV_pilus_assembly_protein_PilM_[Thermoanaerobaculia_bacterium]	ptg004863l	32.295	5.57E-44	167	88
KAB2958337.1_MAG:_Tol-Pal_system_subunit_TolQ_[Thermoanaerobaculia_bacterium]	ptg005341l	30.097	8.5E-09	58.5	97
KAB2958335.1_MAG:_electron_transfer_flavoprotein_subunit_beta/FixA_family_protein_[Thermoanaerobaculia_bacterium]	ptg005554l	36.15	5.58E-15	78.2	81
KAB2958334.1_MAG:_protein_TolR_[Thermoanaerobaculia_bacterium]	ptg005341l	39.695	2.1E-22	95.5	88
KAB2958330.1_MAG:_transcriptional_regulator,_partial_[Thermoanaerobaculia_bacterium]	ptg002106l	40.206	2.11E-15	74.7	76
KAB2958328.1_MAG:_type_II/IV_secretion_system_protein_[Thermoanaerobaculia_bacterium]	ptg004018l	43.186	7.4E-123	403	87
KAB2958320.1_MAG:_PDZ_domain-containing_protein,_partial_[Thermoanaerobaculia_bacterium]	ptg004564l	40.476	1.48E-11	62.8	76
KAB2958319.1_MAG:_sulfatase_[Thermoanaerobaculia_bacterium]	ptg002760l	24.082	3.24E-17	89	83
KAB2958318.1_MAG:_electron_transfer_flavoprotein_subunit_alpha/FixB_family_protein,_partial_[Thermoanaerobaculia_bacterium]	LG19	64.615	2.06E-18	89.7	21
KAB2958317.1_MAG:_TlpA_family_protein_disulfide_reductase_[Thermoanaerobaculia_bacterium]	ptg004529l	36.066	2.91E-19	87.8	68
KAB2958316.1_MAG:_vitamin_B12-dependent_ribonucleotide_reductase_[Thermoanaerobaculia_bacterium]	ptg002475l	23.817	1.44E-30	134	64
KAB2958311.1_MAG:_dihydrolipoyl_dehydrogenase_[Thermoanaerobaculia_bacterium]	ptg003780l	42.129	2.88E-100	333	95
KAB2958310.1_MAG:_thiamine_pyrophosphate-dependent_dehydrogenase_E1_component_subunit_alpha_[Thermoanaerobaculia_bacterium]	ptg004010l	26.948	1.2E-20	96.7	94
KAB2958309.1_MAG:_alpha-ketoacid_dehydrogenase_subunit_beta_[Thermoanaerobaculia_bacterium]	ptg002612l	28.659	1.79E-42	160	99
KAB2958308.1_MAG:_hypothetical_protein_F9K16_13630,_partial_[Thermoanaerobaculia_bacterium]	ptg003019l	47.222	0.0000055	43.5	95
KAB2958306.1_MAG:_ATP-binding_protein_[Thermoanaerobaculia_bacterium]	ptg003363l	39.583	6.67E-11	62.8	62
KAB2958304.1_MAG:_ABC_transporter_substrate-binding_protein,_partial_[Thermoanaerobaculia_bacterium]	ptg005227l	26.923	2.62E-10	65.9	64

KAB2958303.1_MAG:_FHA_domain-containing_protein,_partial_[Thermoanaerobaculia_bacterium]	ptg001871l	45.833	3.52E-11	63.2	52
KAB2958302.1_MAG:_excinuclease_ABC_subunit_A_[Thermoanaerobaculia_bacterium]	ptg004916l	39.586	6.87E-177	577	87
KAB2958298.1_MAG:_ribonuclease_Z,_partial_[Thermoanaerobaculia_bacterium]	ptg002871l	32.389	1.22E-16	85.5	68
KAB2958295.1_MAG:_preprotein_translocase_subunit_SecA,_partial_[Thermoanaerobaculia_bacterium]	ptg005005l	51.381	0	801	94
KAB2958294.1_MAG:_M23_family_metallopeptidase_[Thermoanaerobaculia_bacterium]	ptg004510l	44.167	1.51E-24	107	41
KAB2958292.1_MAG:_cytochrome_C_assembly_protein,_partial_[Thermoanaerobaculia_bacterium]	ptg002540l	28.358	1.06E-15	85.9	28
KAB2958291.1_MAG:_DNA_topoisomerase_III,_partial_[Thermoanaerobaculia_bacterium]	ptg002246l	44.737	1.69E-34	130	100
KAB2958277.1_MAG:_4-alpha-glucanotransferase_[Thermoanaerobaculia_bacterium]	LG03	26.496	3.78E-09	63.5	46
KAB2958274.1_MAG:_asparagine_synthase_(glutamine-hydrolyzing),_partial_[Thermoanaerobaculia_bacterium]	ptg004111l	32.877	5.83E-51	188	83
KAB2958273.1_MAG:_glycosyltransferase_family_4_protein_[Thermoanaerobaculia_bacterium]	ptg005234l	33.75	1.33E-18	91.7	64
KAB2958272.1_MAG:_TRAP_transporter_large_permease_subunit_[Thermoanaerobaculia_bacterium]	ptg005526l	30.256	3.49E-23	106	90
KAB2958270.1_MAG:_aldehyde_dehydrogenase_family_protein_[Thermoanaerobaculia_bacterium]	ptg001925l	26.98	3.11E-43	168	83
KAB2958269.1_MAG:_sulfatase_[Thermoanaerobaculia_bacterium]	ptg001401l	24.205	9.37E-16	84.3	77
KAB2958268.1_MAG:_glycosyltransferase_family_2_protein_[Thermoanaerobaculia_bacterium]	ptg003431l	29.661	5.62E-19	90.1	85
KAB2958266.1_MAG:_NADP-dependent_malic_enzyme_[Thermoanaerobaculia_bacterium]	ptg005092l	44.474	0	588	97
KAB2958265.1_MAG:_hypothetical_protein_F9K16_13765_[Thermoanaerobaculia_bacterium]	ptg005759l	26.627	0.000000152	54.7	80
KAB2958261.1_MAG:_hypothetical_protein_F9K16_13785_[Thermoanaerobaculia_bacterium]	ptg005913l	23.651	8.99E-09	62.4	45
KAB2958259.1_MAG:_ATP-dependent_chaperone_ClpB,_partial_[Thermoanaerobaculia_bacterium]	ptg002345l	53.72	0	776	98
KAB2958256.1_MAG:_prepilin-type_N-terminal_cleavage/methylation_domain-containing_protein_[Thermoanaerobaculia_bacterium]	ptg004798l	48.837	0.00000101	50.1	31
KAB2958255.1_MAG:_hypothetical_protein_F9K16_13795,_partial_[Thermoanaerobaculia_bacterium]	ptg005260l	27.211	6.86E-14	80.1	36
KAB2958252.1_MAG:_NAD(P)/FAD-dependent_oxidoreductase_[Thermoanaerobaculia_bacterium]	ptg002658l	33.333	4.65E-40	157	68
KAB2958250.1_MAG:_AbgT_family_transporter_[Thermoanaerobaculia_bacterium]	ptg003282l	48.013	3.16E-68	245	88
KAB2958249.1_MAG:_serine/threonine_protein_kinase,_partial_[Thermoanaerobaculia_bacterium]	ptg004928l	39.689	1.26E-47	177	68
KAB2958247.1_MAG:_Rne/Rng_family_ribonuclease,_partial_[Thermoanaerobaculia_bacterium]	ptg002760l	40.454	1.13E-97	334	73
KAB2958245.1_MAG:_serine/threonine_protein_kinase,_partial_[Thermoanaerobaculia_bacterium]	ptg004564l	39.85	7.17E-52	187	82
KAB2958244.1_MAG:_hypothetical_protein_F9K18_12445,_partial_[Thermoanaerobaculia_bacterium]	LG06	60	0.00000753	43.1	95
KAB2958237.1_MAG:_M20_family_metallopeptidase_[Thermoanaerobaculia_bacterium]	ptg002928l	30.651	1.06E-10	67.8	58
KAB2958235.1_MAG:_(deoxy)nucleoside_triphosphate_pyrophosphohydrolase,_partial_[Thermoanaerobaculia_bacterium]	ptg005005l	36.036	8.98E-13	66.6	92
KAB2958233.1_MAG:_hypothetical_protein_F9K16_13855_[Thermoanaerobaculia_bacterium]	LG07	36.842	8.26E-10	67	32
KAB2958232.1_MAG:_MBL_fold_metallo-hydrolase,_partial_[Thermoanaerobaculia_bacterium]	ptg002760l	35.294	2.35E-17	83.6	99
KAB2958231.1_MAG:_SDR_family_oxidoreductase_[Thermoanaerobaculia_bacterium]	ptg003472l	30.328	9.39E-13	73.6	64
KAB2958230.1_MAG:_sulfatase-like_hydrolase/transferase,_partial_[Thermoanaerobaculia_bacterium]	ptg002345l	33.333	0.00000808	50.1	37
KAB2958229.1_MAG:_S9_family_peptidase_[Thermoanaerobaculia_bacterium]	ptg004136l	33.864	1.69E-110	371	94

KAB2958228.1_MAG:_pyridoxal-phosphate_dependent_enzyme,_partial_[Thermoanaerobaculia_bacterium]	ptg004827l	35.811	0.00000123	53.1	56
KAB2958227.1_MAG:_sulfatase-like_hydrolase/transferase,_partial_[Thermoanaerobaculia_bacterium]	ptg004306l	33.913	0.00000156	56.2	14
KAB2958226.1_MAG:_pyridoxine_5'-phosphate_synthase_[Thermoanaerobaculia_bacterium]	ptg003483l	46.746	7.68E-36	120	98
KAB2958224.1_MAG:_sigma-54-dependent_Fis_family_transcriptional_regulator_[Thermoanaerobaculia_bacterium]	ptg005150l	44.072	3.29E-100	333	80
KAB2958222.1_MAG:_NAD-dependent_DNA_ligase_LigA,_partial_[Thermoanaerobaculia_bacterium]	ptg005087l	50.725	2.31E-13	67	77
KAB2958023.1_MAG:_tetratricopeptide_repeat_protein,_partial_[Thermoanaerobaculia_bacterium]	ptg005189l	32.456	0.000000734	56.6	20
KAB2958019.1_MAG:_AI-2E_family_transporter,_partial_[Thermoanaerobaculia_bacterium]	ptg005886l	31.176	6.87E-18	89	47
KAB2958018.1_MAG:_transketolase,_partial_[Thermoanaerobaculia_bacterium]	ptg002612l	31.804	1.07E-33	138	73
KAB2958014.1_MAG:_GTP_3',8-cyclase_MoaA_[Thermoanaerobaculia_bacterium]	ptg005933l	41.194	4.1E-49	180	98
KAB2958010.1_MAG:_signal_recognition_particle-docking_protein_FtsY_[Thermoanaerobaculia_bacterium]	ptg004885l	52.91	2.64E-46	171	61
KAB2958008.1_MAG:_HD_domain-containing_protein_[Thermoanaerobaculia_bacterium]	ptg002928l	44.262	1.12E-32	138	29
KAB2958007.1_MAG:_3-dehydroquinate_synthase,_partial_[Thermoanaerobaculia_bacterium]	ptg004409l	41.026	3.74E-10	60.1	82
KAB2958005.1_MAG:_hypothetical_protein_F9K18_12520,_partial_[Thermoanaerobaculia_bacterium]	ptg005309l	33.553	1.04E-08	57.4	79
KAB2958004.1_MAG:_hypothetical_protein_F9K16_13965,_partial_[Thermoanaerobaculia_bacterium]	ptg003585l	45.536	1.7E-24	102	67
KAB2958003.1_MAG:_GNAT_family_N-acetyltransferase_[Thermoanaerobaculia_bacterium]	ptg002465l	28.07	0.00000484	49.7	92
KAB2958002.1_MAG:_AAA_family_ATPase_[Thermoanaerobaculia_bacterium]	ptg005204l	40.472	1.01E-123	411	85
KAB2957998.1_MAG:_IS3_family_transposase_[Thermoanaerobaculia_bacterium]	ptg005964l	44.068	0.000000169	50.8	61
KAB2957995.1_MAG:_hypothetical_protein_F9K16_13970,_partial_[Thermoanaerobaculia_bacterium]	ptg004351l	37.895	3.15E-12	67.4	52
KAB2957993.1_MAG:_PLP-dependent_transferase_[Thermoanaerobaculia_bacterium]	ptg004895l	40.674	9.35E-84	285	82
KAB2957990.1_MAG:_hypothetical_protein_F9K16_13995_[Thermoanaerobaculia_bacterium]	ptg001224l	31.122	0.00000316	53.9	35
KAB2957822.1_MAG:_glycosyl_hydrolase,_partial_[Thermoanaerobaculia_bacterium]	ptg005375l	45.186	0	838	96
KAB2957819.1_MAG:_type_IV-A_pilus_assembly_ATPase_PilB_[Thermoanaerobaculia_bacterium]	ptg000568l	43.554	1.3E-144	465	99
KAB2957817.1_MAG:_cob(I)yrinic_acid_a,c-diamide_adenosyltransferase_[Thermoanaerobaculia_bacterium]	ptg003336l	36.946	7.42E-21	92.8	95
KAB2957814.1_MAG:_transketolase,_partial_[Thermoanaerobaculia_bacterium]	ptg004917l	55.455	3.81E-25	102	89
KAB2957813.1_MAG:_hypothetical_protein_F9K16_14035_[Thermoanaerobaculia_bacterium]	LG07	36.585	8.63E-21	103	70
KAB2957812.1_MAG:_3-octaprenyl-4-hydroxybenzoate_carboxy-lyase,_partial_[Thermoanaerobaculia_bacterium]	ptg003903l	40.171	2.44E-21	92.4	79
KAB2957811.1_MAG:_lysophospholipid_acyltransferase_family_protein,_partial_[Thermoanaerobaculia_bacterium]	ptg003471l	27.203	4.74E-17	85.1	85
KAB2957805.1_MAG:_ribonuclease_III,_partial_[Thermoanaerobaculia_bacterium]	ptg003583l	34.3	1.29E-26	110	91
KAB2957804.1_MAG:_hypothetical_protein_F9K18_12620,_partial_[Thermoanaerobaculia_bacterium]	ptg004564l	31.694	0.00000121	53.1	71
KAB2957803.1_MAG:_SDR_family_oxidoreductase_[Thermoanaerobaculia_bacterium]	ptg005905l	34.274	4.82E-42	157	92
KAB2957802.1_MAG:_hypothetical_protein_F9K16_14080,_partial_[Thermoanaerobaculia_bacterium]	ptg005430l	51.02	9.21E-09	53.1	70

KAB2957800.1_MAG:_thiolase_family_protein_[Thermoanaerobaculia_bacterium]	ptg004745l	41.902	4.1E-82	278	97
KAB2957797.1_MAG:_sigma-70_family_RNA_polymerase_sigma_factor_[Thermoanaerobaculia_bacterium]	ptg004479l	30.387	4.72E-11	65.1	85
KAB2957795.1_MAG:_S9_family_peptidase_[Thermoanaerobaculia_bacterium]	ptg001762l	32.803	2.68E-39	160	47
KAB2957791.1_MAG:_S9_family_peptidase_[Thermoanaerobaculia_bacterium]	ptg003703l	29.798	1.5E-16	85.1	54
KAB2957626.1_MAG:_hypothetical_protein_F9K16_14105_[Thermoanaerobaculia_bacterium]	ptg002214l	24.762	6.04E-09	63.2	63
KAB2957625.1_MAG:_4Fe-4S_binding_protein,_partial_[Thermoanaerobaculia_bacterium]	ptg005961l	34.568	0.000000876	55.5	19
KAB2957624.1_MAG:_hydroxymethylbilane_synthase,_partial_[Thermoanaerobaculia_bacterium]	ptg001995l	46.471	1.79E-29	118	82
KAB2957623.1_MAG:_response_regulator_transcription_factor_[Thermoanaerobaculia_bacterium]	ptg001814l	44.255	1.72E-51	182	97
KAB2957622.1_MAG:_HAMP_domain-containing_histidine_kinase,_partial_[Thermoanaerobaculia_bacterium]	ptg001814l	30.556	3.37E-14	77.4	75
KAB2957621.1_MAG:_chromosomal_replication_initiator_protein_DnaA_[Thermoanaerobaculia_bacterium]	ptg000372l	49.851	9.66E-105	344	78
KAB2957620.1_MAG:_DNA_polymerase_III_subunit_beta_[Thermoanaerobaculia_bacterium]	ptg000372l	34.043	1.06E-59	212	100
KAB2957619.1_MAG:_AAA_family_ATPase,_partial_[Thermoanaerobaculia_bacterium]	ptg000372l	32.168	4.05E-11	65.9	60
KAB2957617.1_MAG:_cell_division_ATP-binding_protein_FtsE_[Thermoanaerobaculia_bacterium]	ptg004885l	46.711	9.12E-44	139	93
KAB2957615.1_MAG:_OmpA_family_protein,_partial_[Thermoanaerobaculia_bacterium]	ptg003691l	38.095	9.77E-12	63.5	90
KAB2957612.1_MAG:_arginase_[Thermoanaerobaculia_bacterium]	LG29	59.322	6.2E-11	67	50
KAB2957611.1_MAG:_MogA/MoaB_family_molybdenum_cofactor_biosynthesis_protein_[Thermoanaerobaculia_bacterium]	ptg002844l	47.934	1.35E-28	114	71
KAB2957610.1_MAG:_DNA_polymerase_I,_partial_[Thermoanaerobaculia_bacterium]	ptg003762l	39.223	1.81E-56	201	84
KAB2957608.1_MAG:_efflux_RND_transporter_permease_subunit,_partial_[Thermoanaerobaculia_bacterium]	ptg005906l	44.141	1.21E-56	192	89
KAB2957607.1_MAG:_efflux_RND_transporter_periplasmic_adaptor_subunit_[Thermoanaerobaculia_bacterium]	ptg005906l	29.073	1.94E-25	112	82
KAB2957606.1_MAG:_chorismate_synthase,_partial_[Thermoanaerobaculia_bacterium]	ptg003960l	38.816	1.57E-13	72.4	70
KAB2957605.1_MAG:_diguanylate_cyclase,_partial_[Thermoanaerobaculia_bacterium]	ptg005110l	44.048	6.65E-34	141	30
KAB2957450.1_MAG:_4-hydroxybenzoate_octaprenyltransferase_[Thermoanaerobaculia_bacterium]	ptg003903l	46.35	5.48E-35	137	89
KAB2957442.1_MAG:_HD_domain-containing_protein_[Thermoanaerobaculia_bacterium]	ptg004053l	31.529	1.5E-47	177	98
KAB2957441.1_MAG:_TIGR01777_family_protein,_partial_[Thermoanaerobaculia_bacterium]	ptg005375l	46.067	7.15E-23	99.8	82
KAB2957440.1_MAG:_insulinase_family_protein_[Thermoanaerobaculia_bacterium]	ptg001828l	26.705	5.25E-24	110	71
KAB2957434.1_MAG:_methionine--tRNA_ligase_subunit_beta,_partial_[Thermoanaerobaculia_bacterium]	ptg002999l	41.509	3.45E-21	90.9	90
KAB2957433.1_MAG:_TatD_family_deoxyribonuclease_[Thermoanaerobaculia_bacterium]	ptg005067l	42.636	7.9E-49	176	97
KAB2957432.1_MAG:_Nif3-like_dinuclear_metal_center_hexameric_protein_[Thermoanaerobaculia_bacterium]	ptg002270l	40.65	1.48E-58	204	98
KAB2957429.1_MAG:_DnaJ_domain-containing_protein_[Thermoanaerobaculia_bacterium]	ptg005113l	44.118	2.16E-11	66.2	30
KAB2957428.1_MAG:_Stp1/IreP_family_PP2C-type_Ser/Thr_phosphatase_[Thermoanaerobaculia_bacterium]	ptg003137l	36.822	8.93E-39	147	91
KAB2957426.1_MAG:_cytochrome-c_peroxidase_[Thermoanaerobaculia_bacterium]	ptg004510l	28.896	1.67E-21	99.4	76
KAB2957425.1_MAG:_hypothetical_protein_F9K18_12750_[Thermoanaerobaculia_bacterium]	ptg006032l	29.412	1.47E-12	67	97
KAB2957423.1_MAG:_amidohydrolase_[Thermoanaerobaculia_bacterium]	ptg006022l	36	5.51E-69	239	91

KAB2957422.1_MAG:_sulfatase,_partial_[Thermoanaerobaculia_bacterium]	ptg002773l	24.022	2.75E-08	60.8	59
KAB2957420.1_MAG:_cytochrome_C_biogenesis_protein_[Thermoanaerobaculia_bacterium]	ptg002540l	44.828	1.18E-15	80.5	32
KAB2957417.1_MAG:_alpha-ketoacid_dehydrogenase_subunit_beta_[Thermoanaerobaculia_bacterium]	ptg004010l	35.185	5.04E-46	171	94
KAB2957414.1_MAG:_TonB-dependent_receptor_[Thermoanaerobaculia_bacterium]	ptg003282l	35.237	1.24E-72	262	76
KAB2957412.1_MAG:_DNA_mismatch_repair_protein_MutS,_partial_[Thermoanaerobaculia_bacterium]	ptg001460l	48.684	1.83E-125	406	94
KAB2957411.1_MAG:_hypothetical_protein_F9K18_12790,_partial_[Thermoanaerobaculia_bacterium]	ptg005746l	36.066	1.81E-24	106	83
KAB2957289.1_MAG:_magnesium_transporter_[Thermoanaerobaculia_bacterium]	ptg005611l	33.096	1.23E-25	113	69
KAB2957287.1_MAG:_TIGR00730_family_Rossman_fold_protein,_partial_[Thermoanaerobaculia_bacterium]	ptg002465l	34.426	4.15E-23	101	68
KAB2957286.1_MAG:_hypothetical_protein_F9K18_12810_[Thermoanaerobaculia_bacterium]	ptg004242l	37.952	4.8E-29	116	84
KAB2957284.1_MAG:_tRNA_(guanosine(46)-N7)-methyltransferase_TrmB_[Thermoanaerobaculia_bacterium]	ptg004470l	34.94	7.42E-16	79.7	72
KAB2957283.1_MAG:_UDP-N-acetylmuramate_dehydrogenase,_partial_[Thermoanaerobaculia_bacterium]	ptg004662l	30.636	1.08E-13	75.1	59
KAB2957282.1_MAG:_hypothetical_protein_F9K16_14300_[Thermoanaerobaculia_bacterium]	LG02	28.8	2.79E-08	60.1	60
KAB2957279.1_MAG:_glycosyltransferase_[Thermoanaerobaculia_bacterium]	ptg003213l	38.333	2.17E-08	60.5	27
KAB2957277.1_MAG:_deoxyhypusine_synthase_family_protein_[Thermoanaerobaculia_bacterium]	ptg005865l	58.025	4.65E-104	337	99
KAB2957276.1_MAG:_lipoyl(octanoyl)_transferase_LipB_[Thermoanaerobaculia_bacterium]	ptg004799l	43.75	3.07E-18	87.8	63
KAB2957271.1_MAG:_acetyl-CoA_carboxylase_carboxyltransferase_subunit_beta_[Thermoanaerobaculia_bacterium]	ptg000484l	51.418	8.7E-87	286	99
KAB2957270.1_MAG:_bifunctional_folylpolyglutamate_synthase/dihydrofolate_synthase,_partial_[Thermoanaerobaculia_bacterium]	ptg004203l	43.523	1.98E-25	106	91
KAB2957268.1_MAG:_hypothetical_protein_F9K16_14330_[Thermoanaerobaculia_bacterium]	ptg001170l	34.307	2.46E-34	136	86
KAB2957267.1_MAG:_23S_rRNA_(adenine(2503)-C(2))-methyltransferase_RlmN,_partial_[Thermoanaerobaculia_bacterium]	ptg003703l	41.912	3.89E-56	200	79
KAB2957265.1_MAG:_phosphotransferase,_partial_[Thermoanaerobaculia_bacterium]	ptg004480l	30.556	2.62E-17	85.9	89
KAB2957139.1_MAG:_cation-transporting_P-type_ATPase,_partial_[Thermoanaerobaculia_bacterium]	LG16	46.753	7.7E-09	62	19
KAB2957137.1_MAG:_PilT/PilU_family_type_4a_pilus_ATPase_[Thermoanaerobaculia_bacterium]	LG13	45	1.64E-80	271	95
KAB2957135.1_MAG:_type_IV_pilus_twitching_motility_protein_PilT_[Thermoanaerobaculia_bacterium]	ptg000568l	45.562	6.31E-70	241	95
KAB2957134.1_MAG:_ABC_transporter_ATP-binding_protein_[Thermoanaerobaculia_bacterium]	ptg004026l	38.086	1.82E-81	284	80
KAB2957130.1_MAG:_CDP-diacylglycerol--serine_O-phosphatidyltransferase_[Thermoanaerobaculia_bacterium]	ptg002304l	39.732	4.62E-37	142	88
KAB2957127.1_MAG:_hypothetical_protein_F9K16_14390_[Thermoanaerobaculia_bacterium]	ptg001718l	39.844	5.88E-10	66.2	24
KAB2957126.1_MAG:_response_regulator_[Thermoanaerobaculia_bacterium]	ptg002738l	35.329	9.32E-09	61.6	40
KAB2957124.1_MAG:_transcriptional_repressor_NrdR_[Thermoanaerobaculia_bacterium]	ptg004151l	43.421	1.4E-24	102	93
KAB2957123.1_MAG:_endopeptidase_La,_partial_[Thermoanaerobaculia_bacterium]	ptg005527l	45.817	0	679	96
KAB2957122.1_MAG:_amidase_[Thermoanaerobaculia_bacterium]	ptg003250l	29.412	1.19E-37	151	97
KAB2957119.1_MAG:_chromosome_segregation_protein_SMC,_partial_[Thermoanaerobaculia_bacterium]	ptg005087l	46.479	5.74E-46	172	56
KAB2957114.1_MAG:_sigma-70_family_RNA_polymerase_sigma_factor_[Thermoanaerobaculia_bacterium]	ptg005299l	35.849	7.18E-21	93.2	79

KAB2956998.1_MAG:_NAD-dependent_epimerase/dehydratase_family_protein_[Thermoanaerobaculia_bacterium]	ptg003768l	35.35	4.06E-48	176	99
KAB2956997.1_MAG:_hypothetical_protein_F9K18_12940_[Thermoanaerobaculia_bacterium]	ptg002052l	28.571	2.7E-13	72.4	60
KAB2956994.1_MAG:_efflux_RND_transporter_permease_subunit_[Thermoanaerobaculia_bacterium]	ptg004798l	39.267	1.08E-31	129	56
KAB2956993.1_MAG:_RHS_repeat_protein,_partial_[Thermoanaerobaculia_bacterium]	ptg005063l	24.653	6.65E-08	57.4	92
KAB2956992.1_MAG:_polyprenyl_synthetase_family_protein,_partial_[Thermoanaerobaculia_bacterium]	ptg002867l	40.952	6.1E-13	67	95
KAB2956991.1_MAG:_1-deoxy-D-xylulose-5-phosphate_synthase,_partial_[Thermoanaerobaculia_bacterium]	ptg003058l	42.926	2.24E-169	540	97
KAB2956990.1_MAG:_sensor_domain-containing_diguanylate_cyclase_[Thermoanaerobaculia_bacterium]	LG04	35.204	1.61E-18	91.7	50
KAB2956989.1_MAG:_L-lysine_6-transaminase_[Thermoanaerobaculia_bacterium]	ptg002126l	26.531	5.31E-30	127	96
KAB2956988.1_MAG:_hypothetical_protein_F9K18_12970_[Thermoanaerobaculia_bacterium]	ptg004186l	32.143	0.000000715	56.6	15
KAB2956986.1_MAG:_hypothetical_protein_F9K16_14475,_partial_[Thermoanaerobaculia_bacterium]	ptg002246l	34.078	1.91E-26	114	53
KAB2956983.1_MAG:_TraR/DksA_family_transcriptional_regulator,_partial_[Thermoanaerobaculia_bacterium]	ptg001644l	28.571	0.000000834	50.1	64
KAB2956862.1_MAG:_cyclic_nucleotide-binding_domain-containing_protein_[Thermoanaerobaculia_bacterium]	ptg004457l	30.579	0.000000368	53.1	62
KAB2956860.1_MAG:_arsenite_methyltransferase_[Thermoanaerobaculia_bacterium]	ptg001690l	40.513	1.2E-32	130	55
KAB2956859.1_MAG:_DNA_polymerase_III_subunit_alpha,_partial_[Thermoanaerobaculia_bacterium]	ptg006032l	48.744	2.22E-152	495	80
KAB2956855.1_MAG:_radical_SAM_protein_[Thermoanaerobaculia_bacterium]	ptg003511l	27.83	4.85E-17	88.6	43
KAB2956849.1_MAG:_MBOAT_family_protein_[Thermoanaerobaculia_bacterium]	ptg005137l	46.445	3.63E-49	185	40
KAB2956844.1_MAG:_tRNA_pseudouridine(38-40)_synthase_TruA_[Thermoanaerobaculia_bacterium]	ptg003585l	35.714	6.53E-18	87.8	52
KAB2956843.1_MAG:_cyclic_nucleotide-binding_domain-containing_protein_[Thermoanaerobaculia_bacterium]	ptg004457l	29.07	0.000000583	55.1	43
KAB2956840.1_MAG:_FKBP-type_peptidyl-prolyl_cis-trans_isomerase_[Thermoanaerobaculia_bacterium]	ptg003237l	64.151	3.84E-30	122	42
KAB2956837.1_MAG:_isocitrate/isopropylmalate_dehydrogenase_family_protein_[Thermoanaerobaculia_bacterium]	ptg001005l	35.754	1.67E-42	160	99
KAB2956734.1_MAG:_HAMP_domain-containing_protein,_partial_[Thermoanaerobaculia_bacterium]	ptg005903l	27.717	1.57E-15	73.9	56
KAB2956732.1_MAG:_glycine_dehydrogenase_(aminomethyl-transferring),_partial_[Thermoanaerobaculia_bacterium]	ptg005299l	54.476	2.71E-115	373	99
KAB2956731.1_MAG:_glycine_cleavage_system_aminomethyltransferase_GcvT_[Thermoanaerobaculia_bacterium]	ptg005299l	44.077	1.79E-82	277	98
KAB2956730.1_MAG:_glycine_cleavage_system_protein_GcvH_[Thermoanaerobaculia_bacterium]	ptg005318l	36	1.1E-24	101	98
KAB2956729.1_MAG:_polyphosphate_kinase_2_family_protein,_partial_[Thermoanaerobaculia_bacterium]	ptg004579l	47.899	9.34E-64	220	85
KAB2956728.1_MAG:_prohibitin_family_protein_[Thermoanaerobaculia_bacterium]	ptg005920l	28.07	5.54E-10	63.9	57
KAB2956725.1_MAG:_flavodoxin-dependent_(E)-4-hydroxy-3-methylbut-2-enyl-diphosphate_synthase,_partial_[Thermoanaerobaculia_bacterium]	ptg003703l	38.066	3.35E-45	171	79
KAB2956722.1_MAG:_translational_GTPase_TypA_[Thermoanaerobaculia_bacterium]	LG18	51.581	0	627	99
KAB2956721.1_MAG:_ribonuclease_D,_partial_[Thermoanaerobaculia_bacterium]	LG04	39.779	9.52E-23	100	77
KAB2956720.1_MAG:_NUDIX_hydrolase_[Thermoanaerobaculia_bacterium]	ptg003498l	32.955	5.39E-18	84.7	92
KAB2956591.1_MAG:_sugar_ABC_transporter_permease_[Thermoanaerobaculia_bacterium]	ptg005435l	29.412	1.51E-17	86.3	76
KAB2956590.1_MAG:_carbohydrate_ABC_transporter_permease_[Thermoanaerobaculia_bacterium]	ptg003163l	34.615	1.34E-20	94.4	81
KAB2956588.1_MAG:_dihydrofolate_reductase_[Thermoanaerobaculia_bacterium]	ptg001366l	46.012	3.79E-37	139	84

KAB2956587.1_MAG:_LemA_family_protein_[Thermoanaerobaculia_bacterium]	ptg000492l	38.889	1.24E-24	104	88
KAB2956586.1_MAG:_thymidylate_synthase_[Thermoanaerobaculia_bacterium]	ptg004689l	67.045	2.75E-115	367	100
KAB2956585.1_MAG:_ATP-binding_protein,_partial_[Thermoanaerobaculia_bacterium]	ptg004355l	42.609	9.74E-35	135	88
KAB2956584.1_MAG:_hypothetical_protein_F9K18_13115_[Thermoanaerobaculia_bacterium]	ptg005922l	33.884	3.26E-28	115	62
KAB2956578.1_MAG:_thioredoxin_[Thermoanaerobaculia_bacterium]	ptg004480l	51.22	3.33E-23	95.9	76
KAB2956576.1_MAG:_RNA_polymerase_sigma_factor_[Thermoanaerobaculia_bacterium]	ptg003901l	33.333	0.000000247	55.5	63
KAB2956575.1_MAG:_glutamine--fructose-6-phosphate_transaminase_(isomerizing)_[Thermoanaerobaculia_bacterium]	ptg002878l	45.82	6.4E-159	508	100
KAB2956574.1_MAG:_UDP-N-acetylglucosamine_diphosphorylase/glucosamine-1-phosphate_N-acetyltransferase,_partial_[Thermoanaerobaculia_bacterium]	ptg005526l	45	8.49E-70	239	96
KAB2956569.1_MAG:_threonine/serine_exporter_family_protein,_partial_[Thermoanaerobaculia_bacterium]	ptg003928l	37.047	3.77E-25	112	92
KAB2956567.1_MAG:_biotin/lipoyl-binding_protein_[Thermoanaerobaculia_bacterium]	ptg003830l	38.318	3.44E-12	67	64
KAB2956565.1_MAG:_NUDIX_hydrolase_[Thermoanaerobaculia_bacterium]	ptg003839l	32.479	2.19E-09	58.5	69
KAB2956564.1_MAG:_pyridoxamine_5'-phosphate_oxidase_[Thermoanaerobaculia_bacterium]	ptg004500l	47.222	3.77E-34	131	72
KAB2956563.1_MAG:_YqgE/AlgH_family_protein_[Thermoanaerobaculia_bacterium]	ptg005204l	36.145	9.89E-20	89.7	83
KAB2956560.1_MAG:_DNA-3-methyladenine_glycosylase_2_family_protein_[Thermoanaerobaculia_bacterium]	ptg005964l	36.923	7.21E-34	130	93
KAB2956464.1_MAG:_methionine--tRNA_ligase,_partial_[Thermoanaerobaculia_bacterium]	ptg002214l	26.619	8.91E-44	171	96
KAB2956463.1_MAG:_Hsp33_family_molecular_chaperone_HslO,_partial_[Thermoanaerobaculia_bacterium]	ptg003498l	24.157	0.000000185	53.9	93
KAB2956462.1_MAG:_NAD(P)(+)_transhydrogenase_(Re/Si-specific)_subunit_beta_[Thermoanaerobaculia_bacterium]	ptg005929l	53.986	1.4E-83	284	60
KAB2956461.1_MAG:_NAD(P)_transhydrogenase_subunit_alpha_[Thermoanaerobaculia_bacterium]	ptg005929l	61.538	2.29E-22	92.8	83
KAB2956460.1_MAG:_ATP-dependent_protease_subunit_HslV_[Thermoanaerobaculia_bacterium]	ptg004863l	52.809	6.57E-41	149	99
KAB2956459.1_MAG:_ATP-dependent_protease_ATPase_subunit_HslU_[Thermoanaerobaculia_bacterium]	ptg004476l	56.278	1.12E-110	362	97
KAB2956456.1_MAG:_aminoacyl-histidine_dipeptidase_[Thermoanaerobaculia_bacterium]	ptg003431l	44.77	1.86E-119	389	97
KAB2956453.1_MAG:_NAD(P)_transhydrogenase_subunit_alpha_[Thermoanaerobaculia_bacterium]	ptg005929l	62.821	6.43E-22	91.7	83
KAB2956452.1_MAG:_NAD(P)(+)_transhydrogenase_(Re/Si-specific)_subunit_beta_[Thermoanaerobaculia_bacterium]	ptg005929l	55.682	1.24E-84	290	78
KAB2956372.1_MAG:_Holliday_junction_branch_migration_protein_RuvA_[Thermoanaerobaculia_bacterium]	ptg005341l	39.344	4.06E-20	87.8	83
KAB2956371.1_MAG:_Holliday_junction_branch_migration_DNA_helicase_RuvB_[Thermoanaerobaculia_bacterium]	ptg005341l	58.147	6.59E-118	378	91
KAB2956366.1_MAG:_L-glutamate_gamma-semialdehyde_dehydrogenase_[Thermoanaerobaculia_bacterium]	ptg004321l	27.955	8.98E-28	122	79
KAB2956362.1_MAG:_SPFH_domain-containing_protein_[Thermoanaerobaculia_bacterium]	ptg004708l	48.872	1.76E-43	128	68
KAB2956280.1_MAG:_hypothetical_protein_F9K18_13235,_partial_[Thermoanaerobaculia_bacterium]	ptg003603l	44.444	6.06E-75	256	79
KAB2956279.1_MAG:_alanine--glyoxylate_aminotransferase_family_protein_[Thermoanaerobaculia_bacterium]	ptg005754l	31.746	1.21E-17	88.6	52
KAB2956276.1_MAG:_fumarylacetoacetate_hydrolase_family_protein_[Thermoanaerobaculia_bacterium]	ptg003204l	32.932	9.74E-27	112	89
KAB2956275.1_MAG:_ABC_transporter_ATP-binding_protein_[Thermoanaerobaculia_bacterium]	ptg002104l	40.838	2.26E-26	111	77

KAB2956274.1_MAG:_iron_ABC_transporter_permease,_partial_[Thermoanaerobaculia_bacterium]	ptg003901l	45.161	7.9E-12	64.3	97
KAB2956271.1_MAG:_dihydropteroate_synthase,_partial_[Thermoanaerobaculia_bacterium]	ptg003947l	44.33	1.03E-33	130	96
KAB2956269.1_MAG:_SDR_family_NAD(P)-dependent_oxidoreductase_[Thermoanaerobaculia_bacterium]	ptg002723l	32.627	3.15E-20	92.8	93
KAB2956267.1_MAG:_undecaprenyl-phosphate_glucose_phosphotransferase_[Thermoanaerobaculia_bacterium]	ptg000819l	36.747	4.62E-52	194	63
KAB2956187.1_MAG:_RnfABCDGE_type_electron_transport_complex_subunit_E_[Thermoanaerobaculia_bacterium]	ptg002192l	38.462	5.55E-31	122	74
KAB2956186.1_MAG:_electron_transport_complex_subunit_RsxA_[Thermoanaerobaculia_bacterium]	ptg004538l	42.714	3.58E-33	128	96
KAB2956184.1_MAG:_RHS_repeat-associated_core_domain-containing_protein,_partial_[Thermoanaerobaculia_bacterium]	ptg004854l	42.466	1.3E-10	58.5	97
KAB2956179.1_MAG:_NYN_domain-containing_protein_[Thermoanaerobaculia_bacterium]	ptg002557l	32.599	6.5E-21	95.1	94
KAB2956178.1_MAG:_phosphatidate_cytidylyltransferase_[Thermoanaerobaculia_bacterium]	ptg003471l	47.347	5.89E-47	173	73
KAB2956177.1_MAG:_1-acyl-sn-glycerol-3-phosphate_acyltransferase_[Thermoanaerobaculia_bacterium]	ptg003625l	50	7.26E-52	182	90
KAB2956173.1_MAG:_alanine--tRNA_ligase,_partial_[Thermoanaerobaculia_bacterium]	ptg002916l	46.598	7.72E-149	383	85
KAB2956172.1_MAG:_zinc-binding_dehydrogenase,_partial_[Thermoanaerobaculia_bacterium]	ptg004026l	38.824	0.000000106	53.9	51
KAB2956171.1_MAG:_DNA_recombination_protein_RmuC_[Thermoanaerobaculia_bacterium]	ptg004538l	27.005	2.52E-31	131	83
KAB2956092.1_MAG:_phospholipase_D_family_protein,_partial_[Thermoanaerobaculia_bacterium]	ptg004779l	29.101	4.94E-19	89.7	70
KAB2956091.1_MAG:_sigma-70_family_RNA_polymerase_sigma_factor_[Thermoanaerobaculia_bacterium]	ptg004340l	38.15	8.21E-16	78.6	85
KAB2956090.1_MAG:_prolyl_oligopeptidase_family_serine_peptidase,_partial_[Thermoanaerobaculia_bacterium]	ptg002871l	40.278	5.83E-27	112	95
KAB2956086.1_MAG:_NAD(P)H-hydrate_epimerase,_partial_[Thermoanaerobaculia_bacterium]	ptg001460l	34.014	0.00000226	50.1	84
KAB2956085.1_MAG:_anthranilate_phosphoribosyltransferase_[Thermoanaerobaculia_bacterium]	ptg004652l	47.15	7.93E-41	156	57
KAB2956084.1_MAG:_anthranilate_synthase_component_I_family_protein,_partial_[Thermoanaerobaculia_bacterium]	ptg004214l	60.645	2.95E-57	196	90
KAB2956083.1_MAG:_aminodeoxychorismate/anthranilate_synthase_component_II_[Thermoanaerobaculia_bacterium]	ptg003134l	50.521	1.53E-48	172	94
KAB2956076.1_MAG:_F0F1_ATP_synthase_subunit_A_[Thermoanaerobaculia_bacterium]	ptg004470l	31.364	4.73E-11	67	76
KAB2956075.1_MAG:_F0F1_ATP_synthase_subunit_B_[Thermoanaerobaculia_bacterium]	ptg004470l	37.5	4.38E-16	79.3	79
KAB2956072.1_MAG:_response_regulator_[Thermoanaerobaculia_bacterium]	ptg001170l	43.668	2.16E-36	139	100
KAB2956004.1_MAG:_response_regulator,_partial_[Thermoanaerobaculia_bacterium]	ptg005150l	36.364	1.98E-14	72.4	73
KAB2956003.1_MAG:_magnesium_transporter_[Thermoanaerobaculia_bacterium]	ptg004297l	41.081	6.49E-24	109	39
KAB2956001.1_MAG:_cysteine_desulfurase,_partial_[Thermoanaerobaculia_bacterium]	ptg005265l	48.667	1.97E-86	286	96
KAB2956000.1_MAG:_Fe-S_cluster_assembly_protein_SufD_[Thermoanaerobaculia_bacterium]	ptg005265l	37.752	4.25E-71	244	92
KAB2955998.1_MAG:_aldehyde_dehydrogenase_family_protein_[Thermoanaerobaculia_bacterium]	ptg001925l	27.913	7.42E-38	152	84
KAB2955997.1_MAG:_ATP-dependent_metallopeptidase_FtsH/Yme1/Tma_family_protein,_partial_[Thermoanaerobaculia_bacterium]	ptg003039l	57.026	6.92E-143	462	78
KAB2955994.1_MAG:_phosphate_ABC_transporter_substrate-binding_protein_[Thermoanaerobaculia_bacterium]	ptg002567l	62.069	1.21E-113	365	90
KAB2955993.1_MAG:_phosphate_signaling_complex_protein_PhoU_[Thermoanaerobaculia_bacterium]	ptg004075l	40.541	1.18E-46	168	97
KAB2955992.1_MAG:_bacillithiol_biosynthesis_deacetylase_BshB1_[Thermoanaerobaculia_bacterium]	ptg005204l	32.203	5.7E-13	71.6	95

KAB2955991.1_MAG:_N-acetyl-alpha-D-glucosaminyl_L-malate_synthase_BshA_[Thermoanaerobaculia_bacterium]	ptg004384l	26.667	8.95E-16	83.2	76
KAB2955989.1_MAG:_FAD-binding_oxidoreductase,_partial_[Thermoanaerobaculia_bacterium]	ptg001870l	37.963	2.47E-15	79.7	39
KAB2955988.1_MAG:_magnesium_transporter,_partial_[Thermoanaerobaculia_bacterium]	ptg005611l	33.176	8.11E-57	206	94
KAB2955917.1_MAG:_signal_peptide_peptidase_SppA,_partial_[Thermoanaerobaculia_bacterium]	ptg004077l	30.476	6.18E-34	140	81
KAB2955916.1_MAG:_A/G-specific_adenine_glycosylase,_partial_[Thermoanaerobaculia_bacterium]	ptg005844l	48.175	1.34E-34	130	86
KAB2955910.1_MAG:_hypothetical_protein_F9K16_15055,_partial_[Thermoanaerobaculia_bacterium]	ptg003019l	47.222	0.0000055	43.5	95
KAB2955909.1_MAG:_PAS_domain_S-box_protein,_partial_[Thermoanaerobaculia_bacterium]	ptg002738l	32.735	1.19E-24	112	44
KAB2955836.1_MAG:_aminoacyl-histidine_dipeptidase_[Thermoanaerobaculia_bacterium]	ptg003431l	43.238	2.16E-117	385	89
KAB2955829.1_MAG:_thioredoxin_domain-containing_protein,_partial_[Thermoanaerobaculia_bacterium]	ptg001290l	43.782	1.66E-91	305	91
KAB2955828.1_MAG:_response_regulator,_partial_[Thermoanaerobaculia_bacterium]	ptg005150l	35.2	6.37E-17	80.5	76
KAB2955827.1_MAG:_magnesium_transporter_[Thermoanaerobaculia_bacterium]	ptg005611l	29.613	8.9E-40	157	95
KAB2955825.1_MAG:_methionine_gamma-lyase_[Thermoanaerobaculia_bacterium]	ptg004895l	33.813	1.84E-58	210	97
KAB2955824.1_MAG:_hypothetical_protein_F9K18_13500,_partial_[Thermoanaerobaculia_bacterium]	ptg005772l	32.174	8.11E-10	65.5	25
KAB2955822.1_MAG:_peptidylprolyl_isomerase_[Thermoanaerobaculia_bacterium]	ptg002976l	29.878	7.04E-17	82	77
KAB2955820.1_MAG:_HAMP_domain-containing_protein,_partial_[Thermoanaerobaculia_bacterium]	ptg005886l	29.31	1.23E-18	93.6	46
KAB2955819.1_MAG:_GTP-binding_protein,_partial_[Thermoanaerobaculia_bacterium]	ptg004856l	46.602	5.28E-38	142	100
KAB2955752.1_MAG:_hypothetical_protein_F9K16_15125,_partial_[Thermoanaerobaculia_bacterium]	ptg005839l	33.803	0.00000116	54.7	19
KAB2955750.1_MAG:_bifunctional_folylpolyglutamate_synthase/dihydrofolate_synthase,_partial_[Thermoanaerobaculia_bacterium]	ptg004203l	46.512	2.23E-24	102	90
KAB2955749.1_MAG:_acetyl-CoA_carboxylase_carboxyltransferase_subunit_beta_[Thermoanaerobaculia_bacterium]	ptg000484l	51.59	9.75E-93	303	100
KAB2955745.1_MAG:_DNA-3-methyladenine_glycosylase_2_family_protein_[Thermoanaerobaculia_bacterium]	ptg005964l	38.308	6.38E-38	142	95
KAB2955744.1_MAG:_peroxiredoxin_[Thermoanaerobaculia_bacterium]	ptg000732l	38.129	7.99E-24	100	85
KAB2955666.1_MAG:_DNA_helicase_RecQ,_partial_[Thermoanaerobaculia_bacterium]	ptg005046l	43.408	2.9E-119	390	95
KAB2955660.1_MAG:_ribonuclease_HI_[Thermoanaerobaculia_bacterium]	ptg003903l	46.528	8.56E-32	122	88
KAB2955659.1_MAG:_protein_kinase,_partial_[Thermoanaerobaculia_bacterium]	ptg004277l	27.57	2.7E-10	65.9	59
KAB2955657.1_MAG:_asparaginase_[Thermoanaerobaculia_bacterium]	ptg003770l	40.994	1.89E-29	116	98
KAB2955595.1_MAG:_amidohydrolase_[Thermoanaerobaculia_bacterium]	ptg003947l	37.696	2.14E-62	223	82
KAB2955593.1_MAG:_peptidylprolyl_isomerase_[Thermoanaerobaculia_bacterium]	ptg003583l	50.442	9.5E-21	90.5	77
KAB2955592.1_MAG:_sigma-70_family_RNA_polymerase_sigma_factor_[Thermoanaerobaculia_bacterium]	ptg002465l	36.413	2.04E-30	120	95
KAB2955589.1_MAG:_sigma-54-dependent_Fis_family_transcriptional_regulator,_partial_[Thermoanaerobaculia_bacterium]	ptg003057l	45.732	2.48E-89	296	94
KAB2955588.1_MAG:_L-seryl-tRNA(Sec)_selenium_transferase,_partial_[Thermoanaerobaculia_bacterium]	ptg005933l	47.712	2.03E-33	134	44
KAB2955587.1_MAG:_hypothetical_protein_F9K16_15220_[Thermoanaerobaculia_bacterium]	ptg003045l	40.157	5.69E-20	94	43

KAB2955586.1_MAG:_hypothetical_protein_F9K16_15215,_partial_[Thermoanaerobaculia_bacterium]	ptg002429l	33.559	1.7E-23	105	85
KAB2955585.1_MAG:_transcription-repair_coupling_factor,_partial_[Thermoanaerobaculia_bacterium]	ptg004785l	47.352	1.47E-177	565	91
KAB2955522.1_MAG:_single-stranded_DNA-binding_protein_[Thermoanaerobaculia_bacterium]	ptg005756l	39.048	1.52E-16	78.6	73
KAB2955521.1_MAG:_D-tyrosyl-tRNA(Tyr)_deacylase_[Thermoanaerobaculia_bacterium]	ptg005416l	49.655	5.34E-30	117	100
KAB2955520.1_MAG:_site-2_protease_family_protein,_partial_[Thermoanaerobaculia_bacterium]	ptg001245l	31.325	5.43E-21	98.2	69
KAB2955519.1_MAG:_thiazole_synthase_[Thermoanaerobaculia_bacterium]	ptg002723l	52.536	1.08E-84	279	97
KAB2955514.1_MAG:_NAD(P)/FAD-dependent_oxidoreductase,_partial_[Thermoanaerobaculia_bacterium]	ptg002658l	28.448	3.16E-17	89.4	45
KAB2955513.1_MAG:_long-chain_fatty_acid--CoA_ligase,_partial_[Thermoanaerobaculia_bacterium]	ptg005087l	29.231	5.43E-10	61.2	95
KAB2955509.1_MAG:_mechanosensitive_ion_channel_family_protein_[Thermoanaerobaculia_bacterium]	ptg004724l	27.027	2.53E-12	73.2	41
KAB2955442.1_MAG:_L-glutamate_gamma-semialdehyde_dehydrogenase_[Thermoanaerobaculia_bacterium]	ptg004321l	29.586	1.41E-26	119	62
KAB2955438.1_MAG:_glutamine-hydrolyzing_carbamoyl-phosphate_synthase_small_subunit_[Thermoanaerobaculia_bacterium]	ptg001693l	49.219	6.33E-110	357	98
KAB2955435.1_MAG:_HAD_family_hydrolase_[Thermoanaerobaculia_bacterium]	ptg005448l	41.875	1.04E-13	72	87
KAB2955434.1_MAG:_KpsF/GutQ_family_sugar-phosphate_isomerase_[Thermoanaerobaculia_bacterium]	ptg005709l	52.143	7.63E-61	129	88
KAB2955433.1_MAG:_3-deoxy-8-phosphooctulonate_synthase,_partial_[Thermoanaerobaculia_bacterium]	ptg003247l	46.835	9.72E-08	51.2	87
KAB2955432.1_MAG:_cystathionine_gamma-synthase,_partial_[Thermoanaerobaculia_bacterium]	ptg005697l	34.862	1.72E-11	63.2	85
KAB2955431.1_MAG:_Holliday_junction_branch_migration_protein_RuvA_[Thermoanaerobaculia_bacterium]	ptg005341l	40.164	2.46E-16	75.1	87
KAB2955430.1_MAG:_Holliday_junction_branch_migration_DNA_helicase_RuvB_[Thermoanaerobaculia_bacterium]	ptg005341l	58.224	8.03E-115	369	89
KAB2955364.1_MAG:_DUF3520_domain-containing_protein,_partial_[Thermoanaerobaculia_bacterium]	ptg003230l	40.785	1.79E-66	241	51
KAB2955361.1_MAG:_S41_family_peptidase,_partial_[Thermoanaerobaculia_bacterium]	ptg002534l	37.874	2.01E-46	178	57
KAB2955355.1_MAG:_DNA_polymerase_III_subunit_alpha,_partial_[Thermoanaerobaculia_bacterium]	ptg006032l	47.994	6.35E-167	534	93
KAB2955295.1_MAG:_DUF1800_domain-containing_protein,_partial_[Thermoanaerobaculia_bacterium]	ptg004266l	28.028	6.82E-16	85.5	53
KAB2955293.1_MAG:_DUF5117_domain-containing_protein,_partial_[Thermoanaerobaculia_bacterium]	ptg005976l	47.044	7.09E-118	355	83
KAB2955292.1_MAG:_hypothetical_protein_F9K16_15355,_partial_[Thermoanaerobaculia_bacterium]	ptg004798l	30.435	5.94E-09	59.3	62
KAB2955290.1_MAG:_DUF255_domain-containing_protein,_partial_[Thermoanaerobaculia_bacterium]	ptg004321l	35.238	2.43E-09	60.5	41
KAB2955289.1_MAG:_pyridoxal_phosphate-dependent_aminotransferase_[Thermoanaerobaculia_bacterium]	ptg003299l	30.833	2.99E-36	144	90
KAB2955287.1_MAG:_RNA_methyltransferase,_partial_[Thermoanaerobaculia_bacterium]	ptg005487l	36.242	9.37E-25	104	74
KAB2955218.1_MAG:_ZIP_family_metal_transporter_[Thermoanaerobaculia_bacterium]	LG16	41.026	5.24E-15	78.2	44
KAB2955214.1_MAG:_serine/threonine_protein_kinase,_partial_[Thermoanaerobaculia_bacterium]	ptg006032l	33.455	3.09E-42	162	69
KAB2955212.1_MAG:_PBP1A_family_penicillin-binding_protein,_partial_[Thermoanaerobaculia_bacterium]	ptg005297l	34.606	4.56E-47	183	79
KAB2955155.1_MAG:_putative_lipid_II_flippase_FtsW_[Thermoanaerobaculia_bacterium]	ptg004510l	38.583	4.16E-33	134	69
KAB2955154.1_MAG:_UDP-N-acetylglucosamine--N-acetylmuramyl-(pentapeptide)_pyrophosphoryl-undecaprenol_N-	ptg004510l	52.5	0.00000032	47.8	75

acetylglucosamine_transferase,_partial_[Thermoanaerobaculia_bacterium]					
KAB2955150.1_MAG:_YHS_domain-containing_protein_[Thermoanaerobaculia_bacterium]	ptg004735l	49.123	7.58E-33	125	76
KAB2955146.1_MAG:_AAA_family_ATPase_[Thermoanaerobaculia_bacterium]	ptg004623l	47.036	1.47E-65	224	92
KAB2955145.1_MAG:_AAA_domain-containing_protein_[Thermoanaerobaculia_bacterium]	ptg005527l	27.954	5.79E-19	92.4	91
KAB2955079.1_MAG:_pyridoxal-phosphate_dependent_enzyme,_partial_[Thermoanaerobaculia_bacterium]	ptg005554l	50	1.55E-10	58.5	72
KAB2955073.1_MAG:_hypothetical_protein_F9K16_15445_[Thermoanaerobaculia_bacterium]	ptg005301l	32.075	1.15E-20	92	84
KAB2955071.1_MAG:_rhomboid_family_intramembrane_serine_protease_[Thermoanaerobaculia_bacterium]	ptg005092l	53.846	1.48E-11	66.6	36
KAB2955070.1_MAG:_MMPL_family_transporter,_partial_[Thermoanaerobaculia_bacterium]	ptg002976l	25	5.56E-11	66.6	59
KAB2955069.1_MAG:_cytochrome-c_peroxidase_[Thermoanaerobaculia_bacterium]	ptg005967l	29.358	5E-20	94.7	77
KAB2955068.1_MAG:_hypothetical_protein_F9K16_15455_[Thermoanaerobaculia_bacterium]	ptg006032l	30.882	3.12E-15	75.1	90
KAB2955066.1_MAG:_glutamate_racemase_[Thermoanaerobaculia_bacterium]	ptg004538l	30.392	2.87E-16	82.4	69
KAB2955065.1_MAG:_ribonuclease_PH,_partial_[Thermoanaerobaculia_bacterium]	ptg003928l	46.429	6.73E-22	98.2	72
KAB2954994.1_MAG:_hypothetical_protein_F9K16_15480,_partial_[Thermoanaerobaculia_bacterium]	ptg002878l	41.558	1.49E-08	53.9	75
KAB2954993.1_MAG:_recombinase_RecA_[Thermoanaerobaculia_bacterium]	ptg003603l	63.608	2.74E-128	408	91
KAB2954992.1_MAG:_thiamine_pyrophosphate-dependent_dehydrogenase_E1_component_subunit_alpha,_partial_[Thermoanaerobaculia_bacterium]	ptg002612l	35.443	4.76E-19	92	47
KAB2954990.1_MAG:_DNA_translocase_FtsK,_partial_[Thermoanaerobaculia_bacterium]	ptg004538l	44.559	4.79E-119	389	91
KAB2954991.1_MAG:_sulfatase,_partial_[Thermoanaerobaculia_bacterium]	ptg004524l	25.867	2.17E-15	84.3	51
KAB2954989.1_MAG:_phosphoglucosamine_mutase,_partial_[Thermoanaerobaculia_bacterium]	ptg004534l	38.288	9.28E-25	107	75
KAB2954919.1_MAG:_VWA_domain-containing_protein,_partial_[Thermoanaerobaculia_bacterium]	ptg005474l	25.641	0.000000337	55.5	59
KAB2954918.1_MAG:_OmpA_family_protein_[Thermoanaerobaculia_bacterium]	ptg004509l	44.156	1.33E-13	72.4	37
KAB2954917.1_MAG:_S1_RNA-binding_domain-containing_protein,_partial_[Thermoanaerobaculia_bacterium]	ptg001025l	35.692	2.03E-51	191	72
KAB2954916.1_MAG:_Glu-tRNA(Gln)_amidotransferase_subunit_GatE,_partial_[Thermoanaerobaculia_bacterium]	ptg003250l	26.429	4.78E-10	67	42
KAB2954915.1_MAG:_serine/threonine_protein_kinase,_partial_[Thermoanaerobaculia_bacterium]	ptg001897l	40.31	4.1E-48	176	86
KAB2954913.1_MAG:_dihydropyrimidinase,_partial_[Thermoanaerobaculia_bacterium]	ptg002179l	35.211	7.29E-27	118	46
KAB2954911.1_MAG:_hypothetical_protein_F9K16_15510_[Thermoanaerobaculia_bacterium]	ptg004885l	29.73	5.62E-15	82	52
KAB2954909.1_MAG:_sel1_repeat_family_protein,_partial_[Thermoanaerobaculia_bacterium]	ptg004798l	50.314	1.05E-26	110	78
KAB2954908.1_MAG:_hypothetical_protein_F9K18_13895,_partial_[Thermoanaerobaculia_bacterium]	ptg005772l	32.857	1.52E-70	253	92
KAB2954846.1_MAG:_amino_acid_racemase_[Thermoanaerobaculia_bacterium]	ptg005844l	38.537	6.62E-33	129	86
KAB2954845.1_MAG:_protein_kinase,_partial_[Thermoanaerobaculia_bacterium]	ptg004979l	39.91	3.58E-33	134	64
KAB2954842.1_MAG:_peptidase_M28,_partial_[Thermoanaerobaculia_bacterium]	ptg002658l	34.302	1.03E-43	166	79
KAB2954778.1_MAG:_argininosuccinate_synthase,_partial_[Thermoanaerobaculia_bacterium]	ptg001878l	32.107	2.73E-33	132	98
KAB2954775.1_MAG:_murein_biosynthesis_integral_membrane_protein_MurJ,_partial_[Thermoanaerobaculia_bacterium]	ptg002304l	31.419	7.05E-15	79.3	92
KAB2954774.1_MAG:_ABC_transporter_ATP-binding_protein_[Thermoanaerobaculia_bacterium]	ptg004026l	30.561	1.15E-38	156	81
KAB2954771.1_MAG:_lysine--tRNA_ligase_[Thermoanaerobaculia_bacterium]	ptg004479l	49.559	4.82E-130	421	87

KAB2954718.1_MAG:_N-acetylornithine_carbamoyltransferase_[Thermoanaerobaculia_bacterium]	ptg002760l	31.488	2.67E-22	101	87
KAB2954717.1_MAG:_aminotransferase_class_III-fold_pyridoxal_phosphate-dependent_enzyme,_partial_[Thermoanaerobaculia_bacterium]	ptg002126l	42.553	2.77E-19	89.4	64
KAB2954715.1_MAG:_recombination_protein_RecR_[Thermoanaerobaculia_bacterium]	ptg002760l	42.64	9.7E-47	167	100
KAB2954714.1_MAG:_aminotransferase_class_III-fold_pyridoxal_phosphate-dependent_enzyme,_partial_[Thermoanaerobaculia_bacterium]	ptg003982l	29.801	6.33E-18	84.7	78
KAB2954069.1_MAG:_polynucleotide_adenylyltransferase_PcnB,_partial_[Thermoanaerobaculia_bacterium]	ptg003220l	33.582	2E-27	119	57
KAB2954067.1_MAG:_methyltransferase_domain-containing_protein,_partial_[Thermoanaerobaculia_bacterium]	ptg002907l	33.898	3.46E-08	60.1	24
KAB2954065.1_MAG:_arginase_[Thermoanaerobaculia_bacterium]	LG29	60.377	2.04E-08	59.3	47
KAB2954064.1_MAG:_MogA/MoaB_family_molybdenum_cofactor_biosynthesis_protein_[Thermoanaerobaculia_bacterium]	ptg002844l	47.788	6.64E-12	66.2	66
KAB2954019.1_MAG:_nicotinate_phosphoribosyltransferase_[Thermoanaerobaculia_bacterium]	ptg002634l	57.021	1.84E-153	487	96
KAB2954017.1_MAG:_rhomboid_family_intramembrane_serine_protease_[Thermoanaerobaculia_bacterium]	ptg005092l	42.328	1.39E-29	120	74
KAB2953967.1_MAG:_cold-shock_protein_[Thermoanaerobaculia_bacterium]	ptg003498l	55.738	1.07E-19	84	90
KAB2953965.1_MAG:_1-acyl-sn-glycerol-3-phosphate_acyltransferase_[Thermoanaerobaculia_bacterium]	ptg003708l	34.416	2.85E-21	94	68
KAB2953601.1_MAG:_glutaminyl-peptide_cyclotransferase,_partial_[Thermoanaerobaculia_bacterium]	ptg005053l	37.9	2.29E-32	128	87
KAB2953600.1_MAG:_TIGR00266_family_protein_[Thermoanaerobaculia_bacterium]	ptg003839l	57.371	1.92E-77	261	74
KAB2953599.1_MAG:_DNA_helicase_RecQ,_partial_[Thermoanaerobaculia_bacterium]	ptg005046l	43.651	7.14E-116	380	98
KAB2953554.1_MAG:_DUF3520_domain-containing_protein,_partial_[Thermoanaerobaculia_bacterium]	ptg002031l	41.092	1.55E-74	257	79
KAB2953553.1_MAG:_cytochrome_c3_family_protein_[Thermoanaerobaculia_bacterium]	ptg002453l	44.724	1.27E-48	173	78
KAB2953552.1_MAG:_twin-arginine_translocation_signal_domain-containing_protein,_partial_[Thermoanaerobaculia_bacterium]	ptg001690l	32.781	1.61E-15	82	80
KAB2953507.1_MAG:_competence/damage-inducible_protein_A,_partial_[Thermoanaerobaculia_bacterium]	ptg002699l	46.452	8.19E-26	117	27
KAB2953246.1_MAG:_NYN_domain-containing_protein_[Thermoanaerobaculia_bacterium]	ptg002557l	31.349	7.06E-22	98.6	93
KAB2953245.1_MAG:_ion_transporter,_partial_[Thermoanaerobaculia_bacterium]	ptg002976l	32.558	0.000000143	56.2	60
KAB2953242.1_MAG:_sugar_transferase_[Thermoanaerobaculia_bacterium]	ptg003753l	41.818	8.04E-30	119	74
KAB2953189.1_MAG:_2-oxoglutarate_dehydrogenase,_partial_[Thermoanaerobaculia_bacterium]	ptg003019l	44	3.73E-13	67.4	71
KAB2953187.1_MAG:_lytic_transglycosylase_domain-containing_protein,_partial_[Thermoanaerobaculia_bacterium]	ptg004256l	33.784	2.94E-13	73.9	48
KAB2953186.1_MAG:_methylated-DNA--[protein]-cysteine_S-methyltransferase_[Thermoanaerobaculia_bacterium]	ptg005680l	41.509	8.28E-16	78.6	55
KAB2953128.1_MAG:_TRAP_transporter_large_permease_subunit,_partial_[Thermoanaerobaculia_bacterium]	ptg005526l	32.153	4.22E-30	130	65
KAB2952866.1_MAG:_molybdopterin-dependent_oxidoreductase,_partial_[Thermoanaerobaculia_bacterium]	ptg005556l	26.442	5.61E-23	106	89
KAB2952810.1_MAG:_radical_SAM_protein_[Thermoanaerobaculia_bacterium]	ptg003839l	30.052	9.25E-15	76.3	74
KAB2952809.1_MAG:_biotin/lipoyl-binding_protein,_partial_[Thermoanaerobaculia_bacterium]	ptg002477l	53.659	0.00000601	48.1	29

KAB2952808.1_MAG:_6-carboxytetrahydropterin_synthase_QueD_[Thermoanaerobaculia_bacterium]	ptg005533l	35.088	3.42E-13	68.2	80
KAB2952806.1_MAG:_peptidoglycan_DD-metalloendopeptidase_family_protein,_partial_[Thermoanaerobaculia_bacterium]	ptg005526l	29.839	1.54E-11	69.7	34
KAB2952803.1_MAG:_class_I_SAM-dependent_RNA_methyltransferase_[Thermoanaerobaculia_bacterium]	ptg001995l	31.69	7.66E-08	58.5	34
KAB2952760.1_MAG:_HAD-IC_family_P-type_ATPase,_partial_[Thermoanaerobaculia_bacterium]	ptg003880l	30.435	0.00000831	50.1	57
KAB2952758.1_MAG:_4-hydroxybenzoate_decarboxylase,_partial_[Thermoanaerobaculia_bacterium]	ptg001995l	39.785	3.94E-14	70.5	82
KAB2952757.1_MAG:_sigma-70_family_RNA_polymerase_sigma_factor_[Thermoanaerobaculia_bacterium]	ptg004479l	31.544	3.84E-09	59.3	69
KAB2952507.1_MAG:_phenylalanine--tRNA_ligase_subunit_alpha,_partial_[Thermoanaerobaculia_bacterium]	ptg001925l	53.405	1.87E-92	302	99
KAB2952506.1_MAG:_phenylalanine--tRNA_ligase_subunit_beta,_partial_[Thermoanaerobaculia_bacterium]	ptg002928l	41.772	2.39E-55	197	100
KAB2952464.1_MAG:_hypothetical_protein_F9K18_14315,_partial_[Thermoanaerobaculia_bacterium]	ptg001766l	26.855	3.68E-15	81.6	69
KAB2952461.1_MAG:_glycosyltransferase_[Thermoanaerobaculia_bacterium]	ptg002123l	39.13	4.01E-13	73.9	40
KAB2952420.1_MAG:_cyclic_nucleotide-binding_domain-containing_protein_[Thermoanaerobaculia_bacterium]	ptg002907l	31.481	0.00000229	53.1	33
KAB2952419.1_MAG:_tRNA_pseudouridine(38-40)_synthase_TruA,_partial_[Thermoanaerobaculia_bacterium]	ptg004564l	37.559	3.91E-11	65.5	99
KAB2952416.1_MAG:_tRNA_(adenosine(37)-N6)-dimethylallyltransferase_MiaA_[Thermoanaerobaculia_bacterium]	ptg003471l	38.591	2.74E-59	208	97
KAB2952257.1_MAG:_FAD-binding_protein,_partial_[Thermoanaerobaculia_bacterium]	ptg002199l	39.048	4.26E-15	73.2	91
KAB2952215.1_MAG:_stress_response_translation_initiation_inhibitor_YciH_[Thermoanaerobaculia_bacterium]	ptg001690l	36.957	3.47E-09	56.6	76
KAB2952182.1_MAG:_aldehyde_dehydrogenase_family_protein,_partial_[Thermoanaerobaculia_bacterium]	ptg001925l	32.208	1.42E-36	145	93
KAB2952066.1_MAG:_serine/threonine_protein_kinase,_partial_[Thermoanaerobaculia_bacterium]	ptg004928l	37.066	2.62E-36	144	65
KAB2952064.1_MAG:_membrane_protein_insertase_YidC,_partial_[Thermoanaerobaculia_bacterium]	ptg000372l	43.802	3.81E-52	195	46
KAB2951920.1_MAG:_DNA_translocase_FtsK_[Thermoanaerobaculia_bacterium]	ptg004538l	45.114	9.62E-117	383	88
KAB2951763.1_MAG:_molybdopterin-synthase_adenylyltransferase_MoeB_[Thermoanaerobaculia_bacterium]	ptg002848l	36.441	4.14E-38	149	62
KAB2951592.1_MAG:_dipeptidase_PepE,_partial_[Thermoanaerobaculia_bacterium]	ptg002313l	48.98	1.63E-55	193	87
KAB2951498.1_MAG:_AAA_domain-containing_protein_[Thermoanaerobaculia_bacterium]	ptg004240l	30.601	1.63E-21	100	91
KAB2951458.1_MAG:_xanthine_dehydrogenase_family_protein_molybdopterin-binding_subunit,_partial_[Thermoanaerobaculia_bacterium]	ptg005556l	25.524	2.16E-13	74.7	83
KAB2951457.1_MAG:_M28_family_peptidase,_partial_[Thermoanaerobaculia_bacterium]	ptg005732l	31.557	2.18E-21	102	48
KAB2951363.1_MAG:_exodeoxyribonuclease_VII_large_subunit,_partial_[Thermoanaerobaculia_bacterium]	ptg003058l	35.294	2.44E-33	134	87
KAB2951321.1_MAG:_peptidyl-prolyl_cis-trans_isomerase_[Thermoanaerobaculia_bacterium]	ptg002976l	57.862	2.53E-55	191	83
KAB2951244.1_MAG:_MBOAT_family_protein_[Thermoanaerobaculia_bacterium]	ptg005137l	51.534	1.56E-46	177	33
KAB2951121.1_MAG:_anthranilate_synthase_component_I_family_protein,_partial_[Thermoanaerobaculia_bacterium]	ptg004214l	56.067	9.03E-70	238	82
KAB2951120.1_MAG:_aminodeoxychorismate/anthranilate_synthase_component_II_[Thermoanaerobaculia_bacterium]	ptg003134l	52.332	5.11E-52	182	95
KAB2951119.1_MAG:_anthranilate_phosphoribosyltransferase,_partial_[Thermoanaerobaculia_bacterium]	ptg002909l	41.772	2.26E-12	64.7	80

KAB2951117.1_MAG:_SDR_family_oxidoreductase,_partial_[Thermoanaerobaculia_bacterium]	ptg004575l	37.681	2.28E-12	70.5	51
KAB2951083.1_MAG:_ATP-binding_cassette_domain-containing_protein,_partial_[Thermoanaerobaculia_bacterium]	ptg001995l	55.172	5E-34	129	76
KAB2951082.1_MAG:_arylesterase_[Thermoanaerobaculia_bacterium]	ptg001995l	38.462	5.55E-35	134	83
KAB2951081.1_MAG:_hypothetical_protein_F9K18_14590,_partial_[Thermoanaerobaculia_bacterium]	ptg002030l	35.443	9.72E-11	59.7	83
KAB2951068.1_MAG:_molybdopterin-synthase_adenylyltransferase_MoeB_[Thermoanaerobaculia_bacterium]	ptg002848l	33.523	8.54E-21	97.8	49
KAB2951039.1_MAG:_dihydroorotate_dehydrogenase_[Thermoanaerobaculia_bacterium]	ptg005474l	25.081	1.06E-11	69.3	91
KAB2950996.1_MAG:_molybdopterin-dependent_oxidoreductase,_partial_[Thermoanaerobaculia_bacterium]	ptg005556l	28.671	2.85E-42	167	88
KAB2950983.1_MAG:_winged_helix-turn-helix_domain-containing_protein_[Thermoanaerobaculia_bacterium]	ptg001814l	39.744	7.07E-11	60.1	89
KAB2950982.1_MAG:_HAMP_domain-containing_histidine_kinase,_partial_[Thermoanaerobaculia_bacterium]	ptg002760l	33.071	1.79E-11	68.6	44
KAB2950950.1_MAG:_protein-L-isoaspartate(D-aspartate)_O-methyltransferase_[Thermoanaerobaculia_bacterium]	ptg004026l	47.807	2.15E-50	180	93
KAB2950939.1_MAG:_RES_domain-containing_protein_[Thermoanaerobaculia_bacterium]	ptg002465l	35.165	3.19E-11	61.6	87
KAB2950901.1_MAG:_asparagine_synthetase_B,_partial_[Thermoanaerobaculia_bacterium]	ptg004111l	35.273	1.64E-45	173	60
KAB2950848.1_MAG:_riboflavin_synthase_[Thermoanaerobaculia_bacterium]	ptg003830l	40.299	6.88E-38	142	95
KAB2950847.1_MAG:_bifunctional_diaminohydroxyphosphoribosylaminopyrimidine_deaminase/5-amino-6-(5-phosphoribosylamino)uracil_reductase_RibD,_partial_[Thermoanaerobaculia_bacterium]	ptg002928l	38.281	1.16E-19	93.6	77
KAB2950797.1_MAG:_thioredoxin_[Thermoanaerobaculia_bacterium]	ptg001995l	45.545	1.85E-27	108	94
KAB2950755.1_MAG:_cell_division_protein_FtsA,_partial_[Thermoanaerobaculia_bacterium]	ptg004510l	34.979	1.36E-47	174	77
KAB2950719.1_MAG:_mechanosensitive_ion_channel_[Thermoanaerobaculia_bacterium]	ptg004798l	26.601	5.09E-16	85.1	43
KAB2950716.1_MAG:_imidazole_glycerol_phosphate_synthase_subunit_HisF_[Thermoanaerobaculia_bacterium]	ptg002534l	39.147	1.04E-43	161	98
KAB2950715.1_MAG:_bifunctional_phosphoribosyl-AMP_cyclohydrolase/phosphoribosyl-ATP_diphosphatase_HisIE_[Thermoanaerobaculia_bacterium]	ptg004372l	43.243	3.29E-08	57	33
KAB2950687.1_MAG:_CBS_domain-containing_protein,_partial_[Thermoanaerobaculia_bacterium]	ptg002429l	42.105	1.71E-10	68.2	13
KAB2950685.1_MAG:_polysaccharide_biosynthesis_tyrosine_autokinase,_partial_[Thermoanaerobaculia_bacterium]	ptg003058l	39.894	2.47E-33	140	33
KAB2950659.1_MAG:_metalloprotease,_partial_[Thermoanaerobaculia_bacterium]	ptg005333l	34.842	3.95E-29	118	85
KAB2950655.1_MAG:_histidinol_dehydrogenase_[Thermoanaerobaculia_bacterium]	ptg002031l	40.741	1.31E-44	166	98
KAB2950654.1_MAG:_aminotransferase_class_I/II-fold_pyridoxal_phosphate-dependent_enzyme,_partial_[Thermoanaerobaculia_bacterium]	ptg005435l	37.64	2.44E-28	117	64
KAB2950622.1_MAG:_exodeoxyribonuclease_VII_large_subunit,_partial_[Thermoanaerobaculia_bacterium]	ptg003058l	36.431	6.64E-37	145	71
KAB2950556.1_MAG:_hypothetical_protein_F9K18_14850,_partial_[Thermoanaerobaculia_bacterium]	ptg002573l	33.117	0.00000209	50.1	89
KAB2950528.1_MAG:_M1_family_metallopeptidase,_partial_[Thermoanaerobaculia_bacterium]	ptg002465l	32.136	1.9E-81	281	92
KAB2950526.1_MAG:_dihydroorotate_dehydrogenase_[Thermoanaerobaculia_bacterium]	ptg005474l	26.471	4.71E-12	70.5	91
KAB2950524.1_MAG:_histone_deacetylase,_partial_[Thermoanaerobaculia_bacterium]	ptg005274l	35.51	5.65E-32	135	43
KAB2950497.1_MAG:_tryptophanase,_partial_[Thermoanaerobaculia_bacterium]	ptg004361l	55.482	5.32E-157	496	99
KAB2950496.1_MAG:_16S_rRNA_(cytosine(1402)-N(4))-methyltransferase_RsmH,_partial_[Thermoanaerobaculia_bacterium]	ptg003204l	41.837	8.58E-34	132	81

KAB2950420.1_MAG:_ABC_transporter_ATP-binding_protein_[Thermoanaerobaculia_bacterium]	ptg004579l	41.86	5.52E-42	158	68
KAB2950419.1_MAG:_glycosyl_transferase,_partial_[Thermoanaerobaculia_bacterium]	ptg003592l	30.443	8.41E-51	192	91
KAB2950395.1_MAG:_DNA_polymerase_I,_partial_[Thermoanaerobaculia_bacterium]	ptg004532l	45	5.44E-120	390	99
KAB2950359.1_MAG:_SPFH_domain-containing_protein_[Thermoanaerobaculia_bacterium]	ptg004708l	54.622	5.07E-51	135	77
KAB2950356.1_MAG:_hypothetical_protein_F9K18_15010,_partial_[Thermoanaerobaculia_bacterium]	ptg004790l	32.558	0.00000174	48.1	78
KAB2950328.1_MAG:_metal-dependent_transcriptional_regulator,_partial_[Thermoanaerobaculia_bacterium]	ptg004010l	43.836	1.73E-08	57	37
KAB2950327.1_MAG:_N-acetyl-alpha-D-glucosaminyl_L-malate_synthase_BshA,_partial_[Thermoanaerobaculia_bacterium]	ptg004384l	27.6	1.08E-09	63.9	71
KAB2950326.1_MAG:_bacillithiol_biosynthesis_deacetylase_BshB1,_partial_[Thermoanaerobaculia_bacterium]	ptg005204l	27.368	1.78E-08	57	94
KAB2950294.1_MAG:_hydroxyacid_dehydrogenase,_partial_[Thermoanaerobaculia_bacterium]	ptg001693l	55.056	3.06E-50	177	87
KAB2950089.1_MAG:_lysophospholipid_acyltransferase_family_protein,_partial_[Thermoanaerobaculia_bacterium]	ptg003471l	32.967	4.37E-17	84	69
KAB2950088.1_MAG:_twin-arginine_translocase_subunit_TatC_[Thermoanaerobaculia_bacterium]	ptg002534l	32.105	2.15E-13	73.6	72
KAB2950066.1_MAG:_50S_ribosomal_protein_L19_[Thermoanaerobaculia_bacterium]	ptg005431l	58.879	3.15E-34	128	87
KAB2950065.1_MAG:_tRNA_(guanosine(37)-N1)-methyltransferase_TrmD_[Thermoanaerobaculia_bacterium]	ptg005431l	48.444	1.95E-43	160	87
KAB2950004.1_MAG:_recombinase_XerC,_partial_[Thermoanaerobaculia_bacterium]	ptg003090l	35.897	0.00000209	47	91
KAB2949928.1_MAG:_endopeptidase_La,_partial_[Thermoanaerobaculia_bacterium]	ptg005527l	54.988	2.38E-143	457	91
KAB2949908.1_MAG:_ATP-binding_protein,_partial_[Thermoanaerobaculia_bacterium]	ptg003942l	55.833	1.02E-137	387	99
KAB2949856.1_MAG:_serine/threonine_protein_kinase,_partial_[Thermoanaerobaculia_bacterium]	ptg004652l	34.686	1.05E-42	164	66
KAB2949854.1_MAG:_peptidylprolyl_isomerase_[Thermoanaerobaculia_bacterium]	ptg003583l	44.521	2.89E-28	114	73
KAB2949852.1_MAG:_sigma-70_family_RNA_polymerase_sigma_factor_[Thermoanaerobaculia_bacterium]	ptg002465l	35.676	3.89E-29	116	95
KAB2949788.1_MAG:_hypothetical_protein_F9K18_15165,_partial_[Thermoanaerobaculia_bacterium]	ptg004896l	23.645	0.000000963	53.5	78
KAB2949685.1_MAG:_S1_RNA-binding_domain-containing_protein,_partial_[Thermoanaerobaculia_bacterium]	ptg001025l	32.123	4.73E-54	198	80
KAB2949684.1_MAG:_argininosuccinate_synthase,_partial_[Thermoanaerobaculia_bacterium]	ptg001878l	34.091	1.06E-23	100	97
KAB2949621.1_MAG:_GGDEF_domain-containing_protein_[Thermoanaerobaculia_bacterium]	ptg004532l	35.821	5.46E-19	88.2	65
KAB2949620.1_MAG:_SDR_family_oxidoreductase_[Thermoanaerobaculia_bacterium]	ptg005905l	30.137	2.9E-09	61.2	56
KAB2949538.1_MAG:_glycosyltransferase,_partial_[Thermoanaerobaculia_bacterium]	ptg004409l	34.711	0.000000139	52.8	82
KAB2949536.1_MAG:_FAD-dependent_oxidoreductase_[Thermoanaerobaculia_bacterium]	ptg001658l	32.08	3.49E-54	197	96
KAB2949390.1_MAG:_serine/threonine_protein_kinase,_partial_[Thermoanaerobaculia_bacterium]	ptg003082l	41.786	8.43E-38	145	92
KAB2949286.1_MAG:_sigma-54-dependent_Fis_family_transcriptional_regulator,_partial_[Thermoanaerobaculia_bacterium]	ptg004075l	42.769	2.18E-73	250	93
KAB2949222.1_MAG:_acetyl-CoA_carboxylase_biotin_carboxylase_subunit,_partial_[Thermoanaerobaculia_bacterium]	ptg003168l	53.713	2.82E-125	406	82
KAB2949211.1_MAG:_glycosyltransferase_family_4_protein,_partial_[Thermoanaerobaculia_bacterium]	ptg002534l	33.333	4.5E-09	57.8	60
KAB2949210.1_MAG:_glycosyltransferase_family_4_protein,_partial_[Thermoanaerobaculia_bacterium]	ptg002534l	48.684	1.53E-11	70.5	18
KAB2949114.1_MAG:_hypothetical_protein_F9K18_15340,_partial_[Thermoanaerobaculia_bacterium]	ptg001644l	29.891	0.000000311	55.8	55

KAB2949099.1_MAG:_Glu/Leu/Phe/Val_dehydrogenase_[Thermoanaerobaculia_bacterium]	ptg004284l	47.669	1.81E-136	437	99
KAB2949047.1_MAG:_diguanylate_cyclase,_partial_[Thermoanaerobaculia_bacterium]	LG04	45.536	8.95E-14	78.2	23
KAB2949027.1_MAG:_ATP-dependent_RNA_helicase,_partial_[Thermoanaerobaculia_bacterium]	ptg004529l	49.235	3.72E-72	253	74
KAB2948971.1_MAG:_arsenite_methyltransferase_[Thermoanaerobaculia_bacterium]	ptg001690l	40.611	1.75E-34	135	67
KAB2948936.1_MAG:_phosphotransferase,_partial_[Thermoanaerobaculia_bacterium]	LG03	42.254	4.28E-29	114	99
KAB2948877.1_MAG:_hypothetical_protein_F9K18_15415_[Thermoanaerobaculia_bacterium]	ptg002844l	26.016	1.62E-08	60.1	36
KAB2948861.1_MAG:_AAA_family_ATPase,_partial_[Thermoanaerobaculia_bacterium]	ptg003304l	33.403	3.89E-55	203	93
KAB2948828.1_MAG:_UDP-N-acetylmuramate--L-alanine_ligase_[Thermoanaerobaculia_bacterium]	ptg004510l	39.437	4.29E-87	183	95
WP_038047475.1_redox-sensing_transcriptional_repressor_Rex_[Thermoanaerobaculum_aquaticum]	ptg005030l	40.5	2.43E-35	135	90
WP_038047373.1_type_IV-A_pilus_assembly_ATPase_PilB_[Thermoanaerobaculum_aquaticum]	ptg004468l	44.742	3.88E-134	435	98
WP_038046928.1_chromosome_segregation_protein_SMC_[Thermoanaerobaculum_aquaticum]	ptg004306l	44.048	1.68E-51	203	39
WP_053334855.1_RNA_2',3'-cyclic_phosphodiesterase_[Thermoanaerobaculum_aquaticum]	ptg003223l	28.177	7.18E-12	66.6	95
WP_038047702.1_competence/damage-inducible_protein_A_[Thermoanaerobaculum_aquaticum]	ptg005121l	27.682	1.4E-24	110	67
WP_053335228.1_CRISPR-associated_endonuclease_Cas1_[Thermoanaerobaculum_aquaticum]	ptg004111l	30.034	7.19E-27	115	82
WP_053334968.1_ParB/RepB/Spo0J_family_partition_protein_[Thermoanaerobaculum_aquaticum]	ptg001818l	37.245	9.25E-32	128	65
WP_038048249.1_SDR_family_oxidoreductase_[Thermoanaerobaculum_aquaticum]	ptg004500l	34.247	5.23E-25	106	93
WP_053334849.1_4-(cytidine_5'-diphospho)-2-C-methyl-D-erythritol_kinase_[Thermoanaerobaculum_aquaticum]	ptg004982l	30.282	4.4E-19	91.7	90
WP_081799893.1_exopolysaccharide_biosynthesis_polyprenyl_glycosylphosphotransferase_[Thermoanaerobaculum_aquaticum]	ptg000819l	41.29	5.58E-26	115	34
WP_038046899.1_acyl_carrier_protein_[Thermoanaerobaculum_aquaticum]	ptg004340l	60.274	6.57E-17	76.6	92
WP_053335258.1_NADPH-dependent_glutamate_synthase_[Thermoanaerobaculum_aquaticum]	ptg005689l	27.451	1.42E-10	68.2	67
WP_038048213.1_oxidative_damage_protection_protein_[Thermoanaerobaculum_aquaticum]	ptg004779l	30.189	0.00000909	44.7	70
WP_038047554.1_SDR_family_oxidoreductase_[Thermoanaerobaculum_aquaticum]	ptg005692l	35.361	1.94E-23	103	91
WP_038046764.1_50S_ribosomal_protein_L23_[Thermoanaerobaculum_aquaticum]	ptg004509l	42.857	2.45E-15	73.2	86
WP_038050377.1_type_IV_pilus_assembly_protein_PilM_[Thermoanaerobaculum_aquaticum]	ptg004863l	30.199	4.36E-37	145	98
WP_038046620.1_aspartate--tRNA_ligase_[Thermoanaerobaculum_aquaticum]	ptg003448l	46.735	1.26E-153	491	98
WP_038050324.1_lipoyl_synthase_[Thermoanaerobaculum_aquaticum]	ptg003871l	51.079	2.44E-81	271	94
WP_038049869.1_YebC/PmpR_family_DNA-binding_transcriptional_regulator_[Thermoanaerobaculum_aquaticum]	ptg003903l	47.5	2.08E-49	177	97
WP_053335084.1_NHLP_family_bacteriocin_export_ABC_transporter_peptidase/permease/ATPase_subunit_[Thermoanaerobaculum_aquaticum]	ptg004210l	32.169	7.54E-79	280	73
WP_053334935.1_ABC_transporter_ATP-binding_protein_[Thermoanaerobaculum_aquaticum]	ptg004620l	27.103	3.46E-13	74.7	60
WP_053334927.1_KamA_family_radical_SAM_protein_[Thermoanaerobaculum_aquaticum]	ptg003299l	34.169	2.2E-51	188	82
WP_053334674.1_diguanylate_cyclase_[Thermoanaerobaculum_aquaticum]	LG04	38.462	2.3E-24	111	60

WP_038049846.1_long-chain_fatty_acid--CoA_ligase_[Thermoanaerobaculum_aquaticum]	ptg002844l	26.613	1.53E-31	135	76
WP_038049558.1_TIGR00266_family_protein_[Thermoanaerobaculum_aquaticum]	ptg003839l	60.324	2.72E-87	290	75
WP_038048665.1_bifunctional_sulfate_adenylyltransferase/adenylylsulfate_kinase_[Thermoanaerobaculum_aquaticum]	ptg005689l	65.368	0	331	98
WP_038046853.1_2-oxoacid:acceptor_oxidoreductase_subunit_alpha_[Thermoanaerobaculum_aquaticum]	ptg002867l	32.743	6.25E-38	149	86
WP_038046437.1_23S_rRNA_(pseudouridine(1915)-N(3))-methyltransferase_RlmH_[Thermoanaerobaculum_aquaticum]	LG02	37.079	0.000000231	52.8	54
WP_053335167.1_glutamine--tRNA_ligase/YqeY_domain_fusion_protein_[Thermoanaerobaculum_aquaticum]	ptg005010l	57.812	0	610	89
WP_053335160.1_TonB_family_protein_[Thermoanaerobaculum_aquaticum]	ptg006032l	33.813	2.46E-39	163	30
WP_053335083.1_NHLP_bacteriocin_export_ABC_transporter_permease/ATPase_subunit_[Thermoanaerobaculum_aquaticum]	ptg005433l	29.032	9.83E-38	159	42
WP_038050478.1_pyridoxal_phosphate-dependent_aminotransferase_[Thermoanaerobaculum_aquaticum]	ptg004905l	29.923	1.78E-36	145	95
WP_038050288.1_fumarate_reductase/succinate_dehydrogenase_flavoprotein_subunit_[Thermoanaerobaculum_aquaticum]	ptg002316l	63.108	0	769	100
WP_038049030.1_MOSC_domain-containing_protein_[Thermoanaerobaculum_aquaticum]	ptg005756l	45.775	4.06E-27	115	44
WP_038046671.1_aldehyde_dehydrogenase_family_protein_[Thermoanaerobaculum_aquaticum]	ptg004037l	37.288	3.5E-90	304	98
WP_038046412.1_TIGR00282_family_metallophosphoesterase_[Thermoanaerobaculum_aquaticum]	ptg005484l	44.397	1.7E-59	207	88
WP_038046172.1_long-chain_fatty_acid--CoA_ligase_[Thermoanaerobaculum_aquaticum]	ptg005087l	31.077	1.12E-26	120	52
WP_038050021.1_3-deoxy-8-phosphooctulonate_synthase_[Thermoanaerobaculum_aquaticum]	ptg003247l	47.727	1.17E-73	248	94
WP_038049537.1_zinc_metalloprotease_HtpX_[Thermoanaerobaculum_aquaticum]	ptg005195l	34.51	4.97E-24	105	85
WP_038049129.1_helix-turn-helix_transcriptional_regulator_[Thermoanaerobaculum_aquaticum]	ptg004758l	52.083	0.00000684	47.4	37
WP_081800103.1_3-deoxy-manno-octulosonate_cytidylyltransferase_[Thermoanaerobaculum_aquaticum]	ptg002316l	38.798	8.92E-31	123	62
WP_053335128.1_thymidine_kinase_[Thermoanaerobaculum_aquaticum]	ptg004623l	26.816	1.54E-09	60.5	82
WP_053334943.1_signal_peptide_peptidase_SppA_[Thermoanaerobaculum_aquaticum]	ptg004077l	33.411	3.41E-51	194	73
WP_053334728.1_endopeptidase_La_[Thermoanaerobaculum_aquaticum]	ptg005527l	51.255	0	719	94
WP_038049892.1_enoyl-[acyl-carrier-protein]_reductase_FabL_[Thermoanaerobaculum_aquaticum]	ptg004732l	30.196	4.54E-30	121	98
WP_038049819.1_bifunctional_methylenetetrahydrofolate_dehydrogenase/methenyltetrahydrofolate_cyclohydrolase_FolD_[Thermoanaerobaculum_aquaticum]	ptg002453l	44.981	3.29E-53	190	86
WP_038049543.1_UDP-3-O-(3-hydroxymyristoyl)glucosamine_N-acyltransferase_[Thermoanaerobaculum_aquaticum]	ptg005656l	40.938	2.31E-61	215	90
WP_038049489.1_Fe-S_cluster_assembly_ATPase_SufC_[Thermoanaerobaculum_aquaticum]	ptg003472l	55.6	1.09E-86	284	96
WP_038049475.1_purine-nucleoside_phosphorylase_[Thermoanaerobaculum_aquaticum]	ptg002723l	32.308	7.89E-38	145	94
WP_038048544.1_glucose_1-dehydrogenase_[Thermoanaerobaculum_aquaticum]	ptg005905l	38.672	1.19E-28	117	99
WP_038048040.1_4a-hydroxytetrahydrobiopterin_dehydratase_[Thermoanaerobaculum_aquaticum]	ptg000492l	41.905	2.21E-21	90.9	92
WP_038047712.1_branched-chain_amino_acid_aminotransferase_[Thermoanaerobaculum_aquaticum]	ptg005886l	38.095	3.51E-13	74.7	41

WP_038047487.1_hypoxanthine_phosphoribosyltransferase_[Thermoanaerobaculum_aquaticum]	ptg003290l	40.881	2.74E-25	105	91
WP_038047071.1_malate_dehydrogenase_[Thermoanaerobaculum_aquaticum]	ptg003019l	48.052	3.46E-81	271	96
WP_038046509.1_S-methyl-5-thioribose-1-phosphate_isomerase_[Thermoanaerobaculum_aquaticum]	ptg004010l	42.45	4.74E-53	191	99
WP_038046387.1_YggS_family_pyridoxal_phosphate-dependent_enzyme_[Thermoanaerobaculum_aquaticum]	ptg005005l	44.348	1.98E-27	81.3	98
WP_081800083.1_aminofutalosine_synthase_MqnE_[Thermoanaerobaculum_aquaticum]	ptg001571l	52.342	2.28E-108	352	94
WP_081799837.1_L-2-hydroxyglutarate_oxidase_[Thermoanaerobaculum_aquaticum]	LG26	42.188	0.00000511	52.8	16
WP_053334833.1_asparagine_synthase_(glutamine-hydrolyzing)_[Thermoanaerobaculum_aquaticum]	ptg004111l	29.612	1.46E-63	231	99
WP_053334767.1_nicotinate_phosphoribosyltransferase_[Thermoanaerobaculum_aquaticum]	ptg005048l	40.749	6.09E-88	297	92
WP_053334744.1_30S_ribosomal_protein_S6_[Thermoanaerobaculum_aquaticum]	ptg005487l	29.67	0.00000116	50.1	65
WP_053334670.1_UDP-N-acetylmuramate_dehydrogenase_[Thermoanaerobaculum_aquaticum]	ptg005527l	29.477	1.61E-42	162	94
WP_038050445.1_anthranilate_phosphoribosyltransferase_[Thermoanaerobaculum_aquaticum]	LG07	50.244	2.47E-36	143	60
WP_038050313.1_3-hydroxybutyryl-CoA_dehydrogenase_[Thermoanaerobaculum_aquaticum]	ptg002612l	48.214	1.49E-73	248	99
WP_038049303.1_NAD-dependent_DNA_ligase_LigA_[Thermoanaerobaculum_aquaticum]	ptg005087l	44.262	1.34E-150	487	99
WP_038048826.1_glucose-1-phosphate_thymidylyltransferase_[Thermoanaerobaculum_aquaticum]	ptg004111l	39.496	7.11E-45	168	66
WP_038048569.1_ornithine--oxo-acid_transaminase_[Thermoanaerobaculum_aquaticum]	ptg002126l	36.883	9.44E-75	257	94
WP_038047570.1_ADP-forming_succinate--CoA_ligase_subunit_beta_[Thermoanaerobaculum_aquaticum]	ptg002316l	52.941	4.36E-125	401	99
WP_038046675.1_homoserine_dehydrogenase_[Thermoanaerobaculum_aquaticum]	ptg005433l	32.059	3.66E-36	142	96
WP_038046433.1_GTPase_ObgE_[Thermoanaerobaculum_aquaticum]	ptg002505l	47.576	9.44E-71	242	99
WP_038046222.1_pyridoxal_5'-phosphate_synthase_lyase_subunit_PdxS_[Thermoanaerobaculum_aquaticum]	LG29	77.778	3.44E-19	91.7	50
WP_038050210.1_ornithine_carbamoyltransferase_[Thermoanaerobaculum_aquaticum]	ptg003673l	51.493	1.52E-85	284	86
WP_038050015.1_DNA_mismatch_repair_protein_MutS_[Thermoanaerobaculum_aquaticum]	ptg001460l	39.659	1.47E-165	537	98
WP_038048579.1_RNase_adapter_RapZ_[Thermoanaerobaculum_aquaticum]	ptg005709l	36.94	2.06E-53	190	91
WP_038047569.1_succinate--CoA_ligase_subunit_alpha_[Thermoanaerobaculum_aquaticum]	ptg002316l	59.794	2.57E-98	320	100
WP_053335148.1_exodeoxyribonuclease_VII_large_subunit_[Thermoanaerobaculum_aquaticum]	ptg003058l	53.307	2.18E-82	281	56
WP_053334974.1_Stp1/IreP_family_PP2C-type_Ser/Thr_phosphatase_[Thermoanaerobaculum_aquaticum]	ptg001907l	35.271	6.86E-32	127	100
WP_038050027.1_bifunctional_phosphoribosylaminoimidazolecarboxamide_formyltransferase/IMP_cyclohydrolase_[Thermoanaerobaculum_aquaticum]	ptg000492l	42.775	5.66E-101	337	99
WP_038048736.1_asparagine--tRNA_ligase_[Thermoanaerobaculum_aquaticum]	ptg002477l	32.886	4.46E-75	259	96
WP_038048485.1_2-amino-4-hydroxy-6-hydroxymethyldihydropteridine_diphosphokinase_[Thermoanaerobaculum_aquaticum]	ptg003785l	35.185	2.49E-11	64.7	63
WP_038047735.1_AmmeMemoRadiSam_system_protein_B_[Thermoanaerobaculum_aquaticum]	LG16	28.571	3.11E-20	98.6	51
WP_038046648.1_isocitrate_dehydrogenase_(NADP(+))_[Thermoanaerobaculum_aquaticum]	ptg002573l	34.615	7.81E-16	84	37
WP_038046443.1_NAD+_synthase_[Thermoanaerobaculum_aquaticum]	ptg002304l	35.242	1.47E-21	97.8	78

WP_053334942.1_oligoendopeptidase_F_[Thermoanaerobaculum_aquaticum]	ptg005169l	33.675	4.07E-86	297	97
WP_081800110.1_bifunctional_methionine_sulfoxide_reductase_B/A_protein_[Thermoanaerobaculum_aquaticum]	ptg001224l	47.771	6.64E-41	157	44
WP_053335174.1_vitamin_B12-dependent_ribonucleotide_reductase_[Thermoanaerobaculum_aquaticum]	ptg002475l	22.111	1.08E-20	102	61
WP_038050358.1_ACP_S-malonyltransferase_[Thermoanaerobaculum_aquaticum]	ptg004340l	40.26	6.68E-59	207	99
WP_038049960.1_phosphate_acetyltransferase_[Thermoanaerobaculum_aquaticum]	ptg004584l	33.962	1.52E-30	125	93
WP_038049412.1_riboflavin_synthase_[Thermoanaerobaculum_aquaticum]	ptg003830l	46.734	1.42E-36	138	98
WP_038048804.1_2-amino-4-hydroxy-6-hydroxymethyldihydropteridine_diphosphokinase_[Thermoanaerobaculum_aquaticum]	ptg003785l	33.974	6.73E-15	74.7	92
WP_038048421.1_aspartate_carbamoyltransferase_catalytic_subunit_[Thermoanaerobaculum_aquaticum]	ptg005190l	52.601	5.28E-71	181	82
WP_038048354.1_murein_biosynthesis_integral_membrane_protein_MurJ_[Thermoanaerobaculum_aquaticum]	ptg002304l	33.993	8.02E-26	116	58
WP_038046421.1_ATP-dependent_Clp_endopeptidase_proteolytic_subunit_ClpP_[Thermoanaerobaculum_aquaticum]	ptg005527l	60.417	9.3E-70	233	94
WP_038046186.1_3-methyl-2-oxobutanoate_hydroxymethyltransferase_[Thermoanaerobaculum_aquaticum]	ptg002760l	45.312	7.23E-66	226	92
WP_053334989.1_acetyl-CoA_carboxylase_carboxyltransferase_subunit_alpha_[Thermoanaerobaculum_aquaticum]	ptg002304l	52.29	6.29E-90	296	83
WP_053334815.1_tetraacyldisaccharide_4'-kinase_[Thermoanaerobaculum_aquaticum]	ptg004746l	32.362	5.86E-30	124	90
WP_038050432.1_3-phosphoserine/phosphohydroxythreonine_transaminase_[Thermoanaerobaculum_aquaticum]	ptg005318l	44.875	8.02E-102	333	99
WP_038049768.1_sodium-translocating_pyrophosphatase_[Thermoanaerobaculum_aquaticum]	ptg004711l	39.735	4.45E-145	474	98
WP_038048830.1_dTDP-4-dehydrorhamnose_reductase_[Thermoanaerobaculum_aquaticum]	ptg003827l	37.809	3.39E-42	157	96
WP_038048616.1_porphobilinogen_synthase_[Thermoanaerobaculum_aquaticum]	ptg004997l	44.753	1.27E-70	241	96
WP_038048568.1_DNA_polymerase_III_subunit_alpha_[Thermoanaerobaculum_aquaticum]	ptg003999l	37.046	0	691	99
WP_038048055.1_peptidoglycan-associated_lipoprotein_Pal_[Thermoanaerobaculum_aquaticum]	ptg000834l	33.824	3.63E-13	70.9	70
WP_038047622.1_cob(I)yrinic_acid_a,c-diamide_adenosyltransferase_[Thermoanaerobaculum_aquaticum]	ptg003336l	35	1.1E-20	91.7	99
WP_038046892.1_glycerol-3-phosphate_1-O-acyltransferase_PlsY_[Thermoanaerobaculum_aquaticum]	LG04	40.838	6.24E-08	55.8	94
WP_038046464.1_DNA_polymerase_I_[Thermoanaerobaculum_aquaticum]	ptg003762l	43.883	2.05E-110	376	90
WP_038046338.1_F0F1_ATP_synthase_subunit_epsilon_[Thermoanaerobaculum_aquaticum]	ptg002658l	38.462	0.000000214	52	59
WP_081799911.1_PilT/PilU_family_type_4a_pilus_ATPase_[Thermoanaerobaculum_aquaticum]	ptg004468l	42.655	1.37E-75	262	73
WP_053334761.1_DNA-directed_RNA_polymerase_subunit_alpha_[Thermoanaerobaculum_aquaticum]	ptg004564l	41.304	5.97E-76	256	99
WP_038050530.1_type_I_glutamate--ammonia_ligase_[Thermoanaerobaculum_aquaticum]	ptg004075l	35.456	4.97E-66	233	99
WP_038049281.1_endopeptidase_La_[Thermoanaerobaculum_aquaticum]	ptg005527l	46.164	0	682	95
WP_038049172.1_menaquinone_biosynthesis_decarboxylase_[Thermoanaerobaculum_aquaticum]	ptg001995l	43.238	1.6E-118	386	99
WP_038048620.1_protoporphyrinogen_oxidase_[Thermoanaerobaculum_aquaticum]	ptg004501l	32.129	5.25E-43	119	91
WP_038048463.1_glycogen_synthase_GlgA_[Thermoanaerobaculum_aquaticum]	ptg003785l	38.9	8.83E-96	320	98

WP_038047783.1_adenylosuccinate_synthase_[Thermoanaerobaculum_aquaticum]	ptg005274l	43.675	2.22E-80	274	95
WP_038047299.1_peptide_chain_release_factor_1_[Thermoanaerobaculum_aquaticum]	ptg004982l	48.295	3.22E-93	308	97
WP_038046918.1_3-deoxy-7-phosphoheptulonate_synthase_[Thermoanaerobaculum_aquaticum]	ptg003247l	28.063	2.83E-12	72	68
WP_038046865.1_methylglyoxal_synthase_[Thermoanaerobaculum_aquaticum]	ptg005087l	57.576	4.22E-45	128	86
WP_053335126.1_CDP-diacylglycerol--serine_O-phosphatidyltransferase_[Thermoanaerobaculum_aquaticum]	ptg002304l	41.423	1.31E-46	169	92
WP_038049967.1_iron-sulfur_cluster_insertion_protein_ErpA_[Thermoanaerobaculum_aquaticum]	ptg005487l	51.402	1.4E-30	117	98
WP_038049872.1_ribose_5-phosphate_isomerase_B_[Thermoanaerobaculum_aquaticum]	ptg005354l	44.681	7.55E-33	125	97
WP_038047699.1_glycerol-3-phosphate_1-O-acyltransferase_PlsY_[Thermoanaerobaculum_aquaticum]	LG04	45.745	1.34E-11	66.2	48
WP_038047443.1_phosphoribosylformylglycinamidine_synthase_subunit_PurL_[Thermoanaerobaculum_aquaticum]	ptg002878l	31.744	2.63E-82	290	98
WP_038046600.1_undecaprenyldiphospho-muramoylpentapeptide_beta-N-acetylglucosaminyltransferase_[Thermoanaerobaculum_aquaticum]	ptg004510l	34.503	1.28E-35	141	94
WP_038046404.1_amidase_[Thermoanaerobaculum_aquaticum]	ptg003250l	32.106	7.69E-49	186	87
WP_038050311.1_proline--tRNA_ligase_[Thermoanaerobaculum_aquaticum]	ptg005656l	59.091	5.13E-51	165	57
WP_038049896.1_cysteine_synthase_A_[Thermoanaerobaculum_aquaticum]	ptg005554l	42.857	1.39E-58	206	96
WP_038048109.1_dihydropteroate_synthase_[Thermoanaerobaculum_aquaticum]	ptg003947l	49.027	1.06E-44	165	93
WP_038048050.1_phosphoribosylaminoimidazolesuccinocarboxamide_synthase_[Thermoanaerobaculum_aquaticum]	ptg004573l	45.918	1.93E-43	91.3	77
WP_038046594.1_UDP-N-acetylmuramoyl-L-alanyl-D-glutamate--2,6-diaminopimelate_ligase_[Thermoanaerobaculum_aquaticum]	ptg003536l	29.956	9.76E-35	142	92
WP_038046270.1_M48_family_metallopeptidase_[Thermoanaerobaculum_aquaticum]	ptg005195l	32.09	9.27E-26	113	67
WP_038047111.1_N(4)-(beta-N-acetylglucosaminyl)-L-asparaginase_[Thermoanaerobaculum_aquaticum]	ptg001690l	43.046	7.04E-58	206	84
WP_038046901.1_beta-ketoacyl-ACP_synthase_II_[Thermoanaerobaculum_aquaticum]	ptg003980l	48.985	3.56E-80	273	93
WP_038046247.1_metal-dependent_transcriptional_regulator_[Thermoanaerobaculum_aquaticum]	ptg003770l	26.455	1.97E-09	60.8	81
WP_053335158.1_polyphenol_oxidase_family_protein_[Thermoanaerobaculum_aquaticum]	ptg005689l	34.459	1.76E-12	70.1	60
WP_053334720.1_lytic_transglycosylase_domain-containing_protein_[Thermoanaerobaculum_aquaticum]	ptg004256l	31.544	3.29E-08	61.2	21
WP_038046903.1_electron_transfer_flavoprotein_subunit_beta/FixA_family_protein_[Thermoanaerobaculum_aquaticum]	ptg005554l	33.61	1.82E-27	114	91
WP_081800127.1_dCMP_deaminase_family_protein_[Thermoanaerobaculum_aquaticum]	ptg005856l	42.623	8.17E-09	56.2	43
WP_038050287.1_succinate_dehydrogenase_cytochrome_b_subunit_[Thermoanaerobaculum_aquaticum]	ptg003304l	39.381	1.89E-38	144	98
WP_053335178.1_hypothetical_protein_[Thermoanaerobaculum_aquaticum]	ptg005756l	32.143	6.44E-32	130	72
WP_038048038.1_NAD(P)/FAD-dependent_oxidoreductase_[Thermoanaerobaculum_aquaticum]	ptg005035l	27.61	4.35E-52	192	97
WP_038048089.1_cobalamin_B12-binding_domain-containing_protein_[Thermoanaerobaculum_aquaticum]	LG30	47.297	6.66E-09	56.2	54
WP_081799998.1_hemolysin_III_family_protein_[Thermoanaerobaculum_aquaticum]	ptg004790l	42.69	1.09E-14	76.6	68
WP_053335144.1_uracil-DNA_glycosylase_[Thermoanaerobaculum_aquaticum]	ptg004814l	48.214	2.01E-54	191	91
WP_038049008.1_inorganic_diphosphatase_[Thermoanaerobaculum_aquaticum]	ptg004711l	40.964	2.6E-28	114	88
WP_038047519.1_ExbD/TolR_family_protein_[Thermoanaerobaculum_aquaticum]	ptg005341l	35.115	1.41E-16	78.6	91

WP_053335275.1_NADH-quinone_oxidoreductase_subunit_C_[Thermoanaerobaculum_aquaticum]	ptg005338l	32.773	7.64E-22	70.1	70
WP_038048624.1_class_II_aldolase/adducin_family_protein_[Thermoanaerobaculum_aquaticum]	ptg001878l	33.333	8.96E-21	92.4	97
WP_053334689.1_helix-hairpin-helix_domain-containing_protein_[Thermoanaerobaculum_aquaticum]	ptg003787l	44.068	1.79E-08	54.3	52
WP_038049127.1_RNA_polymerase_sigma_factor_RpoD/SigA_[Thermoanaerobaculum_aquaticum]	ptg001806l	39.3	2.25E-37	144	89
WP_053334878.1_4Fe-4S_dicluster_domain-containing_protein_[Thermoanaerobaculum_aquaticum]	ptg004084l	37.086	1.95E-24	106	53
WP_053335002.1_cytochrome_c_oxidase_subunit_3_family_protein_[Thermoanaerobaculum_aquaticum]	ptg002453l	53.968	3.64E-38	145	74
WP_038049493.1_metal-sulfur_cluster_assembly_factor_[Thermoanaerobaculum_aquaticum]	ptg002634l	36.111	1.7E-14	70.9	99
WP_038048115.1_metal-sulfur_cluster_assembly_factor_[Thermoanaerobaculum_aquaticum]	ptg005265l	36.364	3.21E-13	67	98
WP_053334874.1_cytochrome_bc_complex_cytochrome_b_subunit_[Thermoanaerobaculum_aquaticum]	ptg001921l	43.204	2.42E-50	185	56
WP_038048777.1_HIT_domain-containing_protein_[Thermoanaerobaculum_aquaticum]	ptg004051l	30.882	7.91E-09	57.4	80
WP_038048397.1_NAD(P)_transhydrogenase_subunit_alpha_[Thermoanaerobaculum_aquaticum]	ptg005929l	62.025	2.73E-21	89.7	85
WP_038048893.1_7-carboxy-7-deazaguanine_synthase_QueE_[Thermoanaerobaculum_aquaticum]	ptg003839l	42.126	2.78E-52	184	100
WP_038047094.1_S46_family_peptidase_[Thermoanaerobaculum_aquaticum]	ptg001262l	48.159	0	605	99
WP_053335041.1_S41_family_peptidase_[Thermoanaerobaculum_aquaticum]	ptg002534l	40.468	8.59E-62	224	54
WP_038050289.1_succinate_dehydrogenase/fumarate_reductase_iron-sulfur_subunit_[Thermoanaerobaculum_aquaticum]	ptg003304l	65.517	7.66E-116	369	99
WP_038050454.1_1-(5-phosphoribosyl)-5-[(5-phosphoribosylamino)methylideneamino]_imidazole-4-carboxamide_isomerase_[Thermoanaerobaculum_aquaticum]	ptg002534l	33.054	2.37E-36	139	97
WP_038050244.1_GDP-L-fucose_synthase_[Thermoanaerobaculum_aquaticum]	ptg006001l	58.576	1.53E-111	358	99
WP_038049801.1_NifU_family_protein_[Thermoanaerobaculum_aquaticum]	ptg003708l	39.344	2.78E-11	60.5	85
WP_053334701.1_metal-dependent_transcriptional_regulator_[Thermoanaerobaculum_aquaticum]	ptg002573l	32.283	6.12E-08	55.1	71
WP_038049497.1_undecaprenyl-diphosphate_phosphatase_[Thermoanaerobaculum_aquaticum]	ptg003644l	29.104	7.13E-17	83.6	98
WP_081800080.1_diguanylate_cyclase_[Thermoanaerobaculum_aquaticum]	ptg005110l	39.691	1.81E-28	125	33
WP_038049968.1_DegT/DnrJ/EryC1/StrS_aminotransferase_family_protein_[Thermoanaerobaculum_aquaticum]	ptg001690l	30	2.29E-47	176	98
WP_053335206.1_sodium/proline_symporter_[Thermoanaerobaculum_aquaticum]	ptg001353l	41.436	1.43E-55	204	70
WP_053334711.1_DUF1573_domain-containing_protein_[Thermoanaerobaculum_aquaticum]	ptg003830l	22.109	0.00000226	53.5	82
WP_038049154.1_Hsp20/alpha_crystallin_family_protein_[Thermoanaerobaculum_aquaticum]	ptg002102l	35.338	1.54E-18	84.7	87
WP_038048796.1_DUF3341_domain-containing_protein_[Thermoanaerobaculum_aquaticum]	ptg001690l	38.624	4.64E-31	122	94
WP_038046209.1_nucleotidyltransferase_[Thermoanaerobaculum_aquaticum]	ptg000672l	28.333	2.11E-27	115	98
WP_053335090.1_enoyl-CoA_hydratase/isomerase_family_protein_[Thermoanaerobaculum_aquaticum]	ptg002612l	29.834	8.64E-18	86.3	71
WP_053335067.1_NUDIX_hydrolase_[Thermoanaerobaculum_aquaticum]	ptg002634l	46.667	8.85E-16	76.3	63
WP_053334857.1_alkaline_phosphatase_[Thermoanaerobaculum_aquaticum]	ptg005903l	28.571	1.49E-14	81.3	42
WP_081799847.1_hypothetical_protein_[Thermoanaerobaculum_aquaticum]	ptg002102l	27.35	3.13E-15	81.3	92

WP_038050419.1_bifunctional_2-C-methyl-D-erythritol_4-phosphate_cytidylyltransferase/2-C-methyl-D-erythritol_2,4-cyclodiphosphate_synthase_[Thermoanaerobaculum_aquaticum]	ptg004256l	47.586	2.93E-36	144	78
WP_038048022.1_bifunctional_ADP-dependent_NAD(P)H-hydrate_dehydratase/NAD(P)H-hydrate_epimerase_[Thermoanaerobaculum_aquaticum]	ptg005732l	49.091	2.11E-18	93.2	21
WP_038048563.1_aldehyde_dehydrogenase_family_protein_[Thermoanaerobaculum_aquaticum]	ptg004942l	28.293	1.91E-43	169	80
WP_053334862.1_complex_I_NDUFA9_subunit_family_protein_[Thermoanaerobaculum_aquaticum]	ptg003045l	27.311	2.04E-16	84	73
WP_038046364.1_ABC_transporter_permease_[Thermoanaerobaculum_aquaticum]	ptg002738l	26.797	1.89E-13	76.6	98
WP_053334937.1_site-2_protease_family_protein_[Thermoanaerobaculum_aquaticum]	ptg001245l	33.468	1.02E-26	114	74
WP_038048609.1_phosphomannomutase/phosphoglucomutase_[Thermoanaerobaculum_aquaticum]	ptg002505l	36.364	2.76E-70	246	98
WP_053335065.1_histone_deacetylase_[Thermoanaerobaculum_aquaticum]	ptg005274l	28.402	7.12E-10	63.5	57
WP_038046980.1_carbon_starvation_protein_A_[Thermoanaerobaculum_aquaticum]	ptg002928l	34.36	1.46E-58	215	98
WP_038047767.1_aminoacyl-histidine_dipeptidase_[Thermoanaerobaculum_aquaticum]	ptg003913l	42.857	2.83E-114	374	99
WP_038049773.1_CBS_domain-containing_protein_[Thermoanaerobaculum_aquaticum]	ptg004810l	26.271	5.11E-09	57	76
WP_053335046.1_hypothetical_protein_[Thermoanaerobaculum_aquaticum]	ptg003603l	38.095	5.45E-08	56.6	44
WP_053334832.1_hypothetical_protein_[Thermoanaerobaculum_aquaticum]	ptg002871l	26.329	4.75E-19	93.6	94
WP_038050038.1_hypothetical_protein_[Thermoanaerobaculum_aquaticum]	ptg002612l	46.067	1.79E-14	71.6	75
WP_038050392.1_tetracycline_resistance_MFS_efflux_pump_[Thermoanaerobaculum_aquaticum]	ptg003830l	40.678	8E-33	134	41
WP_038050619.1_hypothetical_protein,_partial_[Thermoanaerobaculum_aquaticum]	ptg003039l	33.533	1.7E-11	64.7	97
WP_038050415.1_hypothetical_protein,_partial_[Thermoanaerobaculum_aquaticum]	ptg003098l	37.647	5.36E-11	60.1	100
WP_038050291.1_hypothetical_protein_[Thermoanaerobaculum_aquaticum]	ptg005076l	24.022	3.18E-24	112	93
WP_038048798.1_hypothetical_protein_[Thermoanaerobaculum_aquaticum]	ptg001690l	41.294	1.14E-76	262	93
WP_038048767.1_hypothetical_protein_[Thermoanaerobaculum_aquaticum]	ptg004662l	38.933	7.22E-78	265	99
WP_038048364.1_hypothetical_protein_[Thermoanaerobaculum_aquaticum]	ptg005375l	35.029	5.09E-172	565	90
WP_038048360.1_hypothetical_protein_[Thermoanaerobaculum_aquaticum]	LG13	30.233	8.89E-11	67.8	34
WP_038048238.1_D-alanine--D-alanine_ligase_[Thermoanaerobaculum_aquaticum]	ptg004466l	26.25	1.61E-14	78.2	92
WP_038048227.1_glycosyl_hydrolase_[Thermoanaerobaculum_aquaticum]	ptg005375l	46.984	0	1005	99
WP_038048128.1_hypothetical_protein_[Thermoanaerobaculum_aquaticum]	ptg003213l	25.737	1.49E-18	93.2	94
WP_038048107.1_hypothetical_protein_[Thermoanaerobaculum_aquaticum]	ptg004534l	39.51	7.62E-33	135	63
WP_038047710.1_hypothetical_protein_[Thermoanaerobaculum_aquaticum]	LG28	39.837	4.75E-12	68.9	50
WP_038047583.1_hypothetical_protein_[Thermoanaerobaculum_aquaticum]	ptg004372l	34.591	1.37E-20	93.6	61
WP_038047235.1_hypothetical_protein_[Thermoanaerobaculum_aquaticum]	ptg005844l	32.143	4.06E-11	67.8	50
WP_038047174.1_hypothetical_protein_[Thermoanaerobaculum_aquaticum]	ptg005354l	25.746	3.15E-18	91.7	58
WP_038046855.1_2-oxoacid:ferredoxin_oxidoreductase_subunit_beta_[Thermoanaerobaculum_aquaticum]	ptg004077l	40	2.82E-40	152	63
KDA55085.1_hypothetical_protein_EG19_00240_[Thermoanaerobaculum_aquaticum]	ptg003098l	27.857	6.83E-16	84	66
KDA55084.1_hypothetical_protein_EG19_00235_[Thermoanaerobaculum_aquaticum]	ptg005527l	36.364	1.48E-14	70.9	94

KDA55083.1_hypothetical_protein_EG19_00230_[Thermoanaerobaculum_aquaticum]	ptg002634l	53.211	9.4E-45	124	99
KDA55078.1_translation_initiation_factor_IF-3_[Thermoanaerobaculum_aquaticum]	ptg001558l	43.571	1.95E-30	119	88
KDA55077.1_L-lysine_6-aminotransferase_[Thermoanaerobaculum_aquaticum]	ptg002126l	28.117	1.34E-32	135	89
KDA55073.1_hypothetical_protein_EG19_00180_[Thermoanaerobaculum_aquaticum]	ptg005150l	39.644	1.22E-96	322	99
KDA55072.1_hypothetical_protein_EG19_00175_[Thermoanaerobaculum_aquaticum]	ptg004026l	33.533	1.75E-41	158	95
KDA55071.1_recombinase_RecA_[Thermoanaerobaculum_aquaticum]	ptg002699l	65.031	4E-125	399	96
KDA55070.1_transcriptional_regulator_[Thermoanaerobaculum_aquaticum]	ptg004579l	39.912	2.29E-49	176	96
KDA55069.1_hypothetical_protein_EG19_00160_[Thermoanaerobaculum_aquaticum]	ptg005010l	30.112	1.65E-30	129	58
KDA55067.1_hypothetical_protein_EG19_00150_[Thermoanaerobaculum_aquaticum]	ptg004457l	28.448	3.21E-10	64.7	39
KDA55066.1_hypothetical_protein_EG19_00145_[Thermoanaerobaculum_aquaticum]	ptg002844l	25.49	2.51E-25	114	71
KDA55063.1_hypothetical_protein_EG19_00130_[Thermoanaerobaculum_aquaticum]	ptg006036l	34.101	6.33E-14	79	38
KDA55062.1_hypothetical_protein_EG19_00125_[Thermoanaerobaculum_aquaticum]	ptg003734l	32.456	2.19E-54	206	67
KDA55061.1_hypothetical_protein_EG19_00120_[Thermoanaerobaculum_aquaticum]	ptg005550l	38.095	1.36E-40	155	91
KDA55060.1_hypothetical_protein_EG19_00115_[Thermoanaerobaculum_aquaticum]	LG04	38.462	2.3E-24	111	60
KDA55059.1_hypothetical_protein_EG19_00110_[Thermoanaerobaculum_aquaticum]	ptg005265l	33.649	1.07E-27	120	45
KDA55058.1_hypothetical_protein_EG19_00105_[Thermoanaerobaculum_aquaticum]	ptg004564l	35.521	2.65E-35	149	31
KDA55056.1_hypothetical_protein_EG19_00090_[Thermoanaerobaculum_aquaticum]	ptg003327l	36.441	1.71E-12	65.9	94
KDA55055.1_hypothetical_protein_EG19_00085_[Thermoanaerobaculum_aquaticum]	ptg004979l	32.83	7.35E-35	136	95
KDA55053.1_alpha-ketoglutarate_decarboxylase_[Thermoanaerobaculum_aquaticum]	ptg004814l	48.014	0	739	88
KDA55052.1_hypothetical_protein_EG19_00065_[Thermoanaerobaculum_aquaticum]	ptg005484l	32.873	4.05E-43	171	53
KDA55051.1_hypothetical_protein_EG19_00060_[Thermoanaerobaculum_aquaticum]	ptg005527l	29.477	1.61E-42	162	94
KDA55040.1_hypothetical_protein_EG19_04385_[Thermoanaerobaculum_aquaticum]	ptg003299l	29.268	2.56E-21	97.4	95
KDA55039.1_hypothetical_protein_EG19_04380_[Thermoanaerobaculum_aquaticum]	ptg002304l	35.242	1.47E-21	97.8	78
KDA55036.1_hypothetical_protein_EG19_04365_[Thermoanaerobaculum_aquaticum]	ptg001658l	37.313	1.32E-09	57.8	57
KDA55035.1_hypothetical_protein_EG19_04360_[Thermoanaerobaculum_aquaticum]	LG02	37.079	0.000000231	52.8	54
KDA55034.1_hypothetical_protein_EG19_04355_[Thermoanaerobaculum_aquaticum]	ptg005150l	36.296	1.75E-41	156	86
KDA55033.1_hypothetical_protein_EG19_04350_[Thermoanaerobaculum_aquaticum]	ptg004055l	41.237	3.61E-12	65.1	77
KDA55032.1_hypothetical_protein_EG19_04345_[Thermoanaerobaculum_aquaticum]	ptg001008l	30.233	8.14E-26	108	97
KDA55031.1_hypothetical_protein_EG19_04340_[Thermoanaerobaculum_aquaticum]	ptg002505l	47.576	9.44E-71	242	99
KDA55030.1_50S_ribosomal_protein_L27_[Thermoanaerobaculum_aquaticum]	ptg003971l	59.722	7.56E-21	88.2	85
KDA55029.1_50S_ribosomal_protein_L21_[Thermoanaerobaculum_aquaticum]	ptg001290l	44.66	1.13E-14	71.6	98
KDA55027.1_hypothetical_protein_EG19_04320_[Thermoanaerobaculum_aquaticum]	ptg002867l	50.852	4.34E-105	342	99
KDA55026.1_hypothetical_protein_EG19_04315_[Thermoanaerobaculum_aquaticum]	ptg002867l	33.484	1.53E-27	115	83
KDA55025.1_transcription_termination_factor_Rho_[Thermoanaerobaculum_aquaticum]	ptg003762l	65.823	1.56E-176	552	85
KDA55024.1_hypothetical_protein_EG19_04305_[Thermoanaerobaculum_aquaticum]	ptg004532l	37.433	1.57E-30	120	93

KDA55023.1_DNA-binding_protein_[Thermoanaerobaculum_aquaticum]	ptg005527l	51.255	0	719	94
KDA55022.1_ATP-dependent_protease_[Thermoanaerobaculum_aquaticum]	ptg004240l	59.5	1.68E-157	495	97
KDA55021.1_hypothetical_protein_EG19_04290_[Thermoanaerobaculum_aquaticum]	ptg005527l	60.417	9.3E-70	233	94
KDA55020.1_hypothetical_protein_EG19_04285_[Thermoanaerobaculum_aquaticum]	ptg005527l	29.24	2.11E-12	73.2	41
KDA55018.1_hypothetical_protein_EG19_04265_[Thermoanaerobaculum_aquaticum]	ptg003625l	32.157	1.24E-19	91.7	97
KDA55017.1_metallophosphoesterase_[Thermoanaerobaculum_aquaticum]	ptg005484l	44.397	1.7E-59	207	88
KDA55015.1_cytochrome_bc_complex_cytochrome_b_subunit_[Thermoanaerobaculum_aquaticum]	ptg001921l	43.662	5.66E-46	172	59
KDA55012.1_hypothetical_protein_EG19_04235_[Thermoanaerobaculum_aquaticum]	ptg001291l	33.172	1.75E-64	228	98
KDA55011.1_hypothetical_protein_EG19_04225_[Thermoanaerobaculum_aquaticum]	ptg004479l	32.668	2E-53	196	86
KDA55010.1_amidase_[Thermoanaerobaculum_aquaticum]	ptg003250l	32.106	5.95E-49	186	89
KDA55009.1_hypothetical_protein_EG19_04215_[Thermoanaerobaculum_aquaticum]	ptg005535l	27.151	5.06E-25	112	78
KDA55006.1_hypothetical_protein_EG19_04200_[Thermoanaerobaculum_aquaticum]	ptg005053l	35.22	3.09E-40	166	33
KDA55004.1_protein_disaggregation_chaperone_[Thermoanaerobaculum_aquaticum]	ptg002304l	52.602	0	778	99
KDA55002.1_hypothetical_protein_EG19_04180_[Thermoanaerobaculum_aquaticum]	ptg004077l	29.644	2.88E-13	75.5	63
KDA55001.1_hypothetical_protein_EG19_04175_[Thermoanaerobaculum_aquaticum]	ptg005825l	49.485	3.36E-18	82.8	70
KDA55000.1_hypothetical_protein_EG19_04170_[Thermoanaerobaculum_aquaticum]	ptg004256l	31.544	3.29E-08	61.2	21
KDA54997.1_30S_ribosomal_protein_S21_[Thermoanaerobaculum_aquaticum]	ptg003583l	52.632	0.000007	45.1	54
KDA54996.1_hypothetical_protein_EG19_04150_[Thermoanaerobaculum_aquaticum]	ptg001460l	27.509	4.25E-11	70.9	34
KDA54995.1_hypothetical_protein_EG19_04145_[Thermoanaerobaculum_aquaticum]	ptg004856l	35.165	7.14E-64	231	61
KDA54994.1_hypothetical_protein_EG19_04140_[Thermoanaerobaculum_aquaticum]	ptg002303l	41.737	2.69E-99	334	83
KDA54991.1_hypothetical_protein_EG19_04120_[Thermoanaerobaculum_aquaticum]	ptg005005l	44.348	1.98E-27	81.3	98
KDA54988.1_hypothetical_protein_EG19_04105_[Thermoanaerobaculum_aquaticum]	ptg003982l	38.211	4.8E-28	125	36
KDA54987.1_hypothetical_protein_EG19_04100_[Thermoanaerobaculum_aquaticum]	ptg005430l	36.828	1.14E-114	384	99
KDA54986.1_hypothetical_protein_EG19_04095_[Thermoanaerobaculum_aquaticum]	ptg002878l	44.444	1.78E-147	475	100
KDA54985.1_hypothetical_protein_EG19_04090_[Thermoanaerobaculum_aquaticum]	ptg004075l	34.796	2.17E-49	180	99
KDA54984.1_hypothetical_protein_EG19_04085_[Thermoanaerobaculum_aquaticum]	ptg004284l	30.032	8.58E-15	79	76
KDA54981.1_recombinase_RecR_[Thermoanaerobaculum_aquaticum]	ptg003168l	45.833	4.32E-49	174	96
KDA54979.1_hypothetical_protein_EG19_04060_[Thermoanaerobaculum_aquaticum]	ptg002760l	47.464	9.88E-79	272	55
KDA54978.1_hypothetical_protein_EG19_04045_[Thermoanaerobaculum_aquaticum]	ptg001690l	50	6.27E-28	112	78
KDA54975.1_hypothetical_protein_EG19_04020_[Thermoanaerobaculum_aquaticum]	ptg002760l	35.417	2.95E-53	129	96
KDA54974.1_hypothetical_protein_EG19_04015_[Thermoanaerobaculum_aquaticum]	ptg002738l	26.797	1.89E-13	76.6	98
KDA54973.1_hypothetical_protein_EG19_04005_[Thermoanaerobaculum_aquaticum]	ptg003585l	35.349	4.46E-30	122	80
KDA54971.1_amino_acid_permease_[Thermoanaerobaculum_aquaticum]	ptg002867l	27.221	6.77E-13	75.5	67
KDA54970.1_sodium:calcium_symporter_[Thermoanaerobaculum_aquaticum]	ptg003960l	33.333	6.77E-37	150	88
KDA54968.1_hypothetical_protein_EG19_03975_[Thermoanaerobaculum_aquaticum]	ptg002902l	32.203	1.31E-17	75.9	77
KDA54967.1_GTP-binding_protein_LepA_[Thermoanaerobaculum_aquaticum]	ptg004662l	54.561	0	651	99

KDA54966.1_hypothetical_protein_EG19_03965_[Thermoanaerobaculum_aquaticum]	ptg005331l	40.439	1.17E-39	153	87
KDA54965.1_hypothetical_protein_EG19_03960_[Thermoanaerobaculum_aquaticum]	ptg004010l	44.51	1.62E-52	189	98
KDA54964.1_glyceraldehyde-3-phosphate_dehydrogenase_[Thermoanaerobaculum_aquaticum]	ptg002223l	53.211	1.97E-103	336	97
KDA54963.1_phosphoglycerate_kinase_[Thermoanaerobaculum_aquaticum]	ptg004779l	43.511	8.58E-92	305	99
KDA54962.1_hypothetical_protein_EG19_03945_[Thermoanaerobaculum_aquaticum]	ptg005107l	42.396	4.12E-44	162	85
KDA54959.1_hypothetical_protein_EG19_03915_[Thermoanaerobaculum_aquaticum]	ptg001909l	26.948	3.7E-19	93.2	80
KDA54958.1_hypothetical_protein_EG19_03910_[Thermoanaerobaculum_aquaticum]	ptg005204l	30.942	5.58E-14	74.7	90
KDA54957.1_hypothetical_protein_EG19_03905_[Thermoanaerobaculum_aquaticum]	ptg004277l	28.025	1.98E-08	54.7	86
KDA54954.1_hypothetical_protein_EG19_03890_[Thermoanaerobaculum_aquaticum]	ptg002658l	38.462	0.000000214	52	59
KDA54953.1_ATP_F0F1_synthase_subunit_beta_[Thermoanaerobaculum_aquaticum]	ptg001921l	68.421	0	581	95
KDA54952.1_hypothetical_protein_EG19_03880_[Thermoanaerobaculum_aquaticum]	ptg000372l	39.322	5.42E-58	203	99
KDA54951.1_ATP_F0F1_synthase_subunit_alpha_[Thermoanaerobaculum_aquaticum]	ptg001624l	61.616	0	639	97
KDA54945.1_hypothetical_protein_EG19_03845_[Thermoanaerobaculum_aquaticum]	ptg003830l	22.109	0.00000226	53.5	82
KDA54944.1_hypothetical_protein_EG19_03840_[Thermoanaerobaculum_aquaticum]	ptg004510l	28.621	4.62E-27	84	87
KDA54940.1_hypothetical_protein_EG19_03820_[Thermoanaerobaculum_aquaticum]	ptg002942l	35.849	1.56E-12	66.2	87
KDA54936.1_peroxiredoxin_[Thermoanaerobaculum_aquaticum]	ptg002047l	49.162	4.61E-51	179	91
KDA54935.1_hypothetical_protein_EG19_03795_[Thermoanaerobaculum_aquaticum]	LG10	55.682	6.14E-20	99	14
KDA54934.1_hypothetical_protein_EG19_03790_[Thermoanaerobaculum_aquaticum]	ptg003980l	27.65	1.1E-09	66.6	21
KDA54931.1_hypothetical_protein_EG19_03775_[Thermoanaerobaculum_aquaticum]	ptg003363l	43.369	1.45E-56	201	80
KDA54929.1_hypothetical_protein_EG19_03765_[Thermoanaerobaculum_aquaticum]	LG04	40.855	7.96E-154	499	85
KDA54928.1_tRNA_(uracil-5-)-methyltransferase_[Thermoanaerobaculum_aquaticum]	ptg004077l	41.429	0.000000556	56.2	15
KDA54927.1_phosphohydrolase_[Thermoanaerobaculum_aquaticum]	ptg002902l	40.217	1.55E-28	76.3	83
KDA54925.1_hypothetical_protein_EG19_03745_[Thermoanaerobaculum_aquaticum]	ptg003090l	43.919	3.19E-65	224	99
KDA54923.1_hypothetical_protein_EG19_03735_[Thermoanaerobaculum_aquaticum]	ptg003829l	35.948	5.9E-13	70.5	71
KDA54921.1_hypothetical_protein_EG19_03725_[Thermoanaerobaculum_aquaticum]	ptg002928l	55.172	1.21E-46	166	98
KDA54920.1_hypothetical_protein_EG19_03720_[Thermoanaerobaculum_aquaticum]	ptg005430l	50.885	1.11E-126	407	100
KDA54918.1_hypothetical_protein_EG19_03710_[Thermoanaerobaculum_aquaticum]	ptg005641l	28.194	2.02E-12	70.9	72
KDA54915.1_short-chain_dehydrogenase_[Thermoanaerobaculum_aquaticum]	ptg005905l	33.071	2.72E-17	84.7	98
KDA54912.1_hypothetical_protein_EG19_03680_[Thermoanaerobaculum_aquaticum]	ptg005363l	55.172	1.04E-50	178	89
KDA54911.1_hypothetical_protein_EG19_03675_[Thermoanaerobaculum_aquaticum]	ptg005195l	32.09	9.27E-26	113	67
KDA54910.1_hypothetical_protein_EG19_03670_[Thermoanaerobaculum_aquaticum]	ptg003131l	27.103	4.1E-10	66.6	52
KDA54909.1_seryl-tRNA_synthetase_[Thermoanaerobaculum_aquaticum]	ptg005746l	47.786	4.53E-119	385	99
KDA54905.1_hypothetical_protein_EG19_03645_[Thermoanaerobaculum_aquaticum]	ptg004085l	51.19	8.93E-44	169	35
KDA54902.1_tyrosine--tRNA_ligase_[Thermoanaerobaculum_aquaticum]	ptg000819l	51	1.06E-134	429	98
KDA54899.1_hypothetical_protein_EG19_03615_[Thermoanaerobaculum_aquaticum]	ptg005922l	36.22	1.42E-31	132	54

KDA54898.1_hypothetical_protein_EG19_03610_[Thermoanaerobaculum_aquaticum]	ptg004017l	29.249	1.18E-20	96.3	76
KDA54897.1_hypothetical_protein_EG19_03605_[Thermoanaerobaculum_aquaticum]	ptg002573l	32.283	6.12E-08	55.1	71
KDA54896.1_hypothetical_protein_EG19_03600_[Thermoanaerobaculum_aquaticum]	ptg003770l	26.455	1.97E-09	60.8	81
KDA54895.1_hypothetical_protein_EG19_03595_[Thermoanaerobaculum_aquaticum]	ptg003831l	27.172	2.17E-36	150	82
KDA54894.1_hypothetical_protein_EG19_03590_[Thermoanaerobaculum_aquaticum]	ptg005922l	37.931	1.91E-15	75.5	79
KDA54892.1_hypothetical_protein_EG19_03580_[Thermoanaerobaculum_aquaticum]	ptg001818l	42.922	4.08E-44	163	75
KDA54891.1_hypothetical_protein_EG19_03575_[Thermoanaerobaculum_aquaticum]	ptg000372l	49.167	3.78E-70	238	88
KDA54890.1_hypothetical_protein_EG19_03570_[Thermoanaerobaculum_aquaticum]	ptg003829l	31.707	6.54E-09	58.5	42
KDA54889.1_hypothetical_protein_EG19_03565_[Thermoanaerobaculum_aquaticum]	ptg000372l	40.249	1.65E-49	187	48
KDA54888.1_hypothetical_protein_EG19_03560_[Thermoanaerobaculum_aquaticum]	ptg000372l	54.286	2.13E-13	67	81
KDA54886.1_hypothetical_protein_EG19_03545_[Thermoanaerobaculum_aquaticum]	ptg000372l	51.592	1.28E-94	315	73
KDA54885.1_hypothetical_protein_EG19_03535_[Thermoanaerobaculum_aquaticum]	ptg000372l	29.301	5.74E-35	140	99
KDA54883.1_DNA_gyrase_subunit_B_[Thermoanaerobaculum_aquaticum]	ptg000372l	60.634	0	564	99
KDA54882.1_DNA_gyrase_subunit_A_[Thermoanaerobaculum_aquaticum]	ptg003798l	46.612	0	685	98
KDA54881.1_transaldolase_[Thermoanaerobaculum_aquaticum]	ptg002573l	27.615	1.22E-10	63.9	89
KDA54880.1_pyridoxal_biosynthesis_protein_[Thermoanaerobaculum_aquaticum]	LG29	77.778	3.44E-19	91.7	50
KDA54878.1_hypothetical_protein_EG19_03500_[Thermoanaerobaculum_aquaticum]	ptg004214l	34.028	1.99E-10	64.3	55
KDA54877.1_hypothetical_protein_EG19_03495_[Thermoanaerobaculum_aquaticum]	ptg003764l	25.328	0.00000357	51.6	88
KDA54876.1_hypothetical_protein_EG19_03490_[Thermoanaerobaculum_aquaticum]	ptg002738l	33.516	9.23E-15	76.3	82
KDA54873.1_hypothetical_protein_EG19_03475_[Thermoanaerobaculum_aquaticum]	ptg004538l	29.703	2.37E-14	76.6	68
KDA54872.1_ribonuclease_PH_[Thermoanaerobaculum_aquaticum]	ptg003928l	51.471	1.08E-43	137	79
KDA54870.1_hypothetical_protein_EG19_03455_[Thermoanaerobaculum_aquaticum]	ptg003009l	43.522	9.49E-127	431	90
KDA54869.1_tRNA_nucleotidyltransferase_(CCA-adding_enzyme)_[Thermoanaerobaculum_aquaticum]	ptg000672l	28.333	2.11E-27	115	98
KDA54868.1_hypothetical_protein_EG19_03445_[Thermoanaerobaculum_aquaticum]	ptg003009l	33.093	8.97E-137	456	95
KDA54866.1_7-keto-8-aminopelargonate_synthetase_[Thermoanaerobaculum_aquaticum]	ptg005614l	36.364	1.64E-63	224	85
KDA54864.1_hypothetical_protein_EG19_03425_[Thermoanaerobaculum_aquaticum]	ptg003762l	43.883	2.05E-110	376	90
KDA54861.1_hypothetical_protein_EG19_03405_[Thermoanaerobaculum_aquaticum]	ptg004856l	45.091	2.81E-58	216	42
KDA54860.1_hypothetical_protein_EG19_03400_[Thermoanaerobaculum_aquaticum]	ptg003361l	32.558	0.0000026	50.4	67
KDA54857.1_hypothetical_protein_EG19_03385_[Thermoanaerobaculum_aquaticum]	ptg003787l	44.068	1.79E-08	54.3	52
KDA54855.1_hypothetical_protein_EG19_03375_[Thermoanaerobaculum_aquaticum]	ptg001571l	35.897	2.61E-28	114	96
KDA54854.1_hypothetical_protein_EG19_03370_[Thermoanaerobaculum_aquaticum]	ptg003903l	36.877	5.21E-30	122	97
KDA54853.1_deoxyadenosine_kinase_[Thermoanaerobaculum_aquaticum]	ptg004203l	27.835	2.51E-10	63.2	79
KDA54852.1_3-methyl-2-oxobutanoate_hydroxymethyltransferase_[Thermoanaerobaculum_aquaticum]	ptg002760l	45.312	7.23E-66	226	92
KDA54851.1_pantoate--beta-alanine_ligase_[Thermoanaerobaculum_aquaticum]	ptg002465l	44.815	7.73E-67	229	95

KDA54850.1_aspartate_decarboxylase_[Thermoanaerobaculum_aquaticum]	ptg004018l	51.24	2.64E-29	114	95
KDA54848.1_hypothetical_protein_EG19_03340_[Thermoanaerobaculum_aquaticum]	ptg003304l	37.657	8.06E-155	504	99
KDA54845.1_hypothetical_protein_EG19_03325_[Thermoanaerobaculum_aquaticum]	ptg005527l	34.078	7.5E-17	80.9	96
KDA54843.1_hypothetical_protein_EG19_03315_[Thermoanaerobaculum_aquaticum]	ptg005087l	31.077	1.12E-26	120	52
KDA54841.1_hypothetical_protein_EG19_03305_[Thermoanaerobaculum_aquaticum]	ptg002612l	39.773	3.25E-14	71.2	71
KDA54839.1_hypothetical_protein_EG19_07580_[Thermoanaerobaculum_aquaticum]	ptg003448l	46.735	1.26E-153	491	98
KDA54838.1_hypothetical_protein_EG19_07575_[Thermoanaerobaculum_aquaticum]	ptg004242l	34.644	1.48E-83	295	85
KDA54832.1_hypothetical_protein_EG19_07540_[Thermoanaerobaculum_aquaticum]	ptg004510l	48.966	1.68E-54	197	74
KDA54829.1_hypothetical_protein_EG19_07525_[Thermoanaerobaculum_aquaticum]	ptg004510l	37.324	3.69E-78	172	96
KDA54828.1_hypothetical_protein_EG19_07520_[Thermoanaerobaculum_aquaticum]	ptg004510l	34.503	1.28E-35	141	94
KDA54826.1_hypothetical_protein_EG19_07510_[Thermoanaerobaculum_aquaticum]	ptg003536l	28.134	0.000000048	59.7	69
KDA54824.1_hypothetical_protein_EG19_07495_[Thermoanaerobaculum_aquaticum]	ptg003536l	29.956	9.76E-35	142	92
KDA54823.1_hypothetical_protein_EG19_07490_[Thermoanaerobaculum_aquaticum]	ptg004863l	25.373	3.98E-12	73.6	48
KDA54821.1_hypothetical_protein_EG19_07480_[Thermoanaerobaculum_aquaticum]	ptg003204l	38.361	1.34E-47	174	98
KDA54818.1_hypothetical_protein_EG19_07465_[Thermoanaerobaculum_aquaticum]	ptg004997l	31.515	7.73E-13	72	58
KDA54814.1_hypothetical_protein_EG19_09195_[Thermoanaerobaculum_aquaticum]	ptg005301l	26.119	1.28E-09	63.9	35
KDA54812.1_hypothetical_protein_EG19_09185_[Thermoanaerobaculum_aquaticum]	ptg005689l	36.364	5.38E-16	53.5	91
KDA54810.1_hypothetical_protein_EG19_09175_[Thermoanaerobaculum_aquaticum]	ptg005920l	26.667	6.35E-11	66.6	67
KDA54809.1_hypothetical_protein_EG19_09170_[Thermoanaerobaculum_aquaticum]	ptg004928l	39.921	1.72E-38	154	52
KDA54804.1_hypothetical_protein_EG19_09145_[Thermoanaerobaculum_aquaticum]	ptg002313l	30.732	1.23E-12	73.6	48
KDA54803.1_hydroxyglutarate_oxidase_[Thermoanaerobaculum_aquaticum]	LG26	42.188	0.00000511	52.8	16
KDA54799.1_hypothetical_protein_EG19_09120_[Thermoanaerobaculum_aquaticum]	ptg004000l	27.451	3.85E-08	57.4	78
KDA54797.1_peptidase_T_[Thermoanaerobaculum_aquaticum]	ptg001690l	43.029	2.38E-110	360	98
KDA54796.1_excinuclease_ABC_subunit_B_[Thermoanaerobaculum_aquaticum]	ptg004926l	58.094	0	767	98
KDA54794.1_hypothetical_protein_EG19_09090_[Thermoanaerobaculum_aquaticum]	ptg004863l	32.015	1.47E-106	363	88
KDA54793.1_inorganic_phosphate_transporter_[Thermoanaerobaculum_aquaticum]	ptg004169l	33.846	4.49E-12	70.9	38
KDA54791.1_hypothetical_protein_EG19_09075_[Thermoanaerobaculum_aquaticum]	ptg003334l	35.577	2.76E-11	63.5	71
KDA54790.1_hypothetical_protein_EG19_09070_[Thermoanaerobaculum_aquaticum]	ptg003282l	48.78	3.97E-13	77.4	11
KDA54788.1_hypothetical_protein_EG19_09060_[Thermoanaerobaculum_aquaticum]	ptg002871l	33.803	4.21E-15	74.7	93
KDA54787.1_hypothetical_protein_EG19_09055_[Thermoanaerobaculum_aquaticum]	ptg005433l	32.059	3.66E-36	142	96
KDA54786.1_aldehyde_dehydrogenase_[Thermoanaerobaculum_aquaticum]	ptg004037l	37.288	3.5E-90	304	98
KDA54784.1_hypothetical_protein_EG19_09040_[Thermoanaerobaculum_aquaticum]	ptg004203l	35.754	4.35E-25	105	97
KDA54782.1_hypothetical_protein_EG19_09030_[Thermoanaerobaculum_aquaticum]	ptg001025l	31.469	0.00000377	50.8	65
KDA54780.1_hypothetical_protein_EG19_09015_[Thermoanaerobaculum_aquaticum]	ptg002942l	41.107	1.3E-38	160	30
KDA54776.1_hypothetical_protein_EG19_08995_[Thermoanaerobaculum_aquaticum]	ptg005487l	36.301	3.79E-21	92	95

KDA54774.1_30S_ribosomal_protein_S18_[Thermoanaerobaculum_aquaticum]	ptg005487l	55.172	1.27E-15	72.8	76
KDA54773.1_hypothetical_protein_EG19_08980_[Thermoanaerobaculum_aquaticum]	ptg005487l	29.67	0.00000116	50.1	65
KDA54772.1_hypothetical_protein_EG19_08975_[Thermoanaerobaculum_aquaticum]	ptg005867l	41.341	1.8E-26	108	93
KDA54771.1_hypothetical_protein_EG19_08970_[Thermoanaerobaculum_aquaticum]	ptg004982l	39.412	1.42E-23	102	74
KDA54770.1_phosphoribosylpyrophosphate_synthetase_[Thermoanaerobaculum_aquaticum]	ptg004982l	55.663	2.66E-113	363	98
KDA54769.1_hypothetical_protein_EG19_08955_[Thermoanaerobaculum_aquaticum]	ptg004779l	29.787	4.31E-23	108	59
KDA54768.1_hypothetical_protein_EG19_08950_[Thermoanaerobaculum_aquaticum]	ptg003625l	38.735	8.73E-47	171	87
KDA54767.1_isocitrate_dehydrogenase_[Thermoanaerobaculum_aquaticum]	ptg002573l	34.615	7.81E-16	84	37
KDA54766.1_hypothetical_protein_EG19_08940_[Thermoanaerobaculum_aquaticum]	ptg000909l	51.2	4.71E-28	110	96
KDA54765.1_hypothetical_protein_EG19_08935_[Thermoanaerobaculum_aquaticum]	ptg002304l	25.938	1.77E-20	100	50
KDA54764.1_hypothetical_protein_EG19_08930_[Thermoanaerobaculum_aquaticum]	LG06	32.456	0.00000103	52.8	49
KDA54763.1_hypothetical_protein_EG19_08925_[Thermoanaerobaculum_aquaticum]	ptg004321l	36.508	1.68E-50	183	94
KDA54762.1_hypothetical_protein_EG19_08920_[Thermoanaerobaculum_aquaticum]	ptg005536l	26.241	0.000000026	60.1	37
KDA54760.1_hypothetical_protein_EG19_08900_[Thermoanaerobaculum_aquaticum]	ptg005341l	25.568	1.25E-19	94.7	95
KDA54759.1_hypothetical_protein_EG19_08895,_partial_[Thermoanaerobaculum_aquaticum]	ptg003171l	30.526	0.00000228	51.2	43
KDA54756.1_hypothetical_protein_EG19_10045_[Thermoanaerobaculum_aquaticum]	ptg001715l	30.657	6.78E-14	79.7	20
KDA54754.1_hypothetical_protein_EG19_10035_[Thermoanaerobaculum_aquaticum]	ptg005484l	33.846	1.99E-43	177	38
KDA54753.1_hypothetical_protein_EG19_10030_[Thermoanaerobaculum_aquaticum]	ptg002928l	34.36	1.46E-58	215	98
KDA54751.1_hypothetical_protein_EG19_10020_[Thermoanaerobaculum_aquaticum]	ptg001995l	30.067	1.12E-45	173	95
KDA54748.1_hypothetical_protein_EG19_10005_[Thermoanaerobaculum_aquaticum]	ptg002907l	26.761	2.87E-18	86.7	94
KDA54745.1_hypothetical_protein_EG19_09990_[Thermoanaerobaculum_aquaticum]	ptg004997l	39.781	5.99E-56	198	89
KDA54744.1_hypothetical_protein_EG19_09985_[Thermoanaerobaculum_aquaticum]	ptg005976l	27.969	1.6E-15	85.5	33
KDA54743.1_hypothetical_protein_EG19_09980_[Thermoanaerobaculum_aquaticum]	ptg002278l	40.541	3.92E-08	59.7	18
KDA54741.1_hypothetical_protein_EG19_09970_[Thermoanaerobaculum_aquaticum]	ptg002214l	30.047	3.74E-12	69.7	78
KDA54739.1_hypothetical_protein_EG19_09960_[Thermoanaerobaculum_aquaticum]	ptg001925l	44.383	0	769	96
KDA54738.1_hypothetical_protein_EG19_09955_[Thermoanaerobaculum_aquaticum]	ptg003363l	43.043	5.41E-43	163	61
KDA54737.1_hypothetical_protein_EG19_09950_[Thermoanaerobaculum_aquaticum]	ptg002102l	28.182	1.65E-14	79	88
KDA54736.1_hypothetical_protein_EG19_09945_[Thermoanaerobaculum_aquaticum]	ptg002867l	31.579	3.73E-15	81.6	45
KDA54734.1_hypothetical_protein_EG19_09935_[Thermoanaerobaculum_aquaticum]	ptg004959l	29.282	9E-37	145	90
KDA54733.1_hypothetical_protein_EG19_09930_[Thermoanaerobaculum_aquaticum]	ptg003536l	40.8	2.38E-18	83.2	93
KDA54732.1_hypothetical_protein_EG19_09925_[Thermoanaerobaculum_aquaticum]	ptg004321l	32.883	1.5E-60	220	81
KDA54731.1_hypothetical_protein_EG19_09920_[Thermoanaerobaculum_aquaticum]	ptg000619l	27.698	2.52E-17	87	80
KDA54730.1_hypothetical_protein_EG19_09915_[Thermoanaerobaculum_aquaticum]	ptg005048l	40.749	6.09E-88	297	92
KDA54728.1_hypothetical_protein_EG19_09905_[Thermoanaerobaculum_aquaticum]	ptg003057l	43.082	5.73E-53	209	25
KDA54727.1_hypothetical_protein_EG19_09900_[Thermoanaerobaculum_aquaticum]	ptg002123l	33.415	2.97E-55	201	94

KDA54725.1_hypothetical_protein_EG19_09890_[Thermoanaerobaculum_aquaticum]	ptg004306l	44.048	1.68E-51	203	39
KDA54720.1_3-deoxy-7-phosphoheptulonate_synthase_[Thermoanaerobaculum_aquaticum]	ptg003247l	28.063	2.83E-12	72	68
KDA54717.1_phosphopantetheine_adenylyltransferase_[Thermoanaerobaculum_aquaticum]	ptg004885l	49.664	1.41E-42	154	90
KDA54714.1_electron_transfer_flavoprotein_subunit_alpha_[Thermoanaerobaculum_aquaticum]	LG19	70.833	5.4E-24	106	63
KDA54713.1_hypothetical_protein_EG19_09830_[Thermoanaerobaculum_aquaticum]	ptg005554l	33.61	1.82E-27	114	91
KDA54712.1_3-oxoacyl-ACP_synthase_[Thermoanaerobaculum_aquaticum]	ptg003980l	48.985	3.56E-80	273	93
KDA54711.1_acyl_carrier_protein_[Thermoanaerobaculum_aquaticum]	ptg004340l	60.274	6.57E-17	76.6	92
KDA54708.1_hypothetical_protein_EG19_09805_[Thermoanaerobaculum_aquaticum]	LG04	40.838	6.24E-08	55.8	94
KDA54704.1_hypothetical_protein_EG19_09785_[Thermoanaerobaculum_aquaticum]	ptg003336l	41.026	7.53E-37	141	89
KDA54703.1_hypothetical_protein_EG19_09780_[Thermoanaerobaculum_aquaticum]	ptg003336l	42.723	2.6E-40	151	85
KDA54701.1_epimerase_[Thermoanaerobaculum_aquaticum]	ptg001690l	39.936	1.72E-70	241	99
KDA54697.1_hypothetical_protein_EG19_09750_[Thermoanaerobaculum_aquaticum]	ptg003382l	35.195	1.58E-100	343	80
KDA54696.1_UDP-glucose_6-dehydrogenase_[Thermoanaerobaculum_aquaticum]	ptg001925l	46.606	8.42E-120	388	98
KDA54695.1_methylglyoxal_synthase_[Thermoanaerobaculum_aquaticum]	ptg005087l	57.576	2.84E-45	129	89
KDA54692.1_2-oxoacid:ferredoxin_oxidoreductase_subunit_beta_[Thermoanaerobaculum_aquaticum]	ptg004077l	40	2.82E-40	152	63
KDA54691.1_2-oxoglutarate_ferredoxin_oxidoreductase_subunit_alpha_[Thermoanaerobaculum_aquaticum]	ptg002867l	32.743	6.25E-38	149	86
KDA54685.1_hypothetical_protein_EG19_09685_[Thermoanaerobaculum_aquaticum]	ptg005754l	33.065	0.0000001	53.1	86
KDA54684.1_tryptophanase_[Thermoanaerobaculum_aquaticum]	ptg004361l	45.934	1.93E-132	425	99
KDA54682.1_hypothetical_protein_EG19_09655_[Thermoanaerobaculum_aquaticum]	ptg005046l	37.975	2.28E-08	55.1	53
KDA54679.1_multidrug_transporter_AcrB_[Thermoanaerobaculum_aquaticum]	ptg005692l	28.118	7.24E-40	166	43
KDA54678.1_hypothetical_protein_EG19_09635_[Thermoanaerobaculum_aquaticum]	ptg000732l	24.742	0.00000017	57	50
KDA54675.1_50S_ribosomal_protein_L17_[Thermoanaerobaculum_aquaticum]	ptg004509l	51.724	8.46E-32	122	83
KDA54674.1_hypothetical_protein_EG19_09615_[Thermoanaerobaculum_aquaticum]	ptg004564l	41.304	5.97E-76	256	99
KDA54673.1_30S_ribosomal_protein_S4_[Thermoanaerobaculum_aquaticum]	ptg004509l	55.024	1.04E-62	213	100
KDA54672.1_30S_ribosomal_protein_S11_[Thermoanaerobaculum_aquaticum]	ptg001245l	61.594	8.61E-44	155	100
KDA54671.1_30S_ribosomal_protein_S13_[Thermoanaerobaculum_aquaticum]	ptg004509l	59.483	3.09E-39	142	91
KDA54670.1_translation_initiation_factor_IF-1_[Thermoanaerobaculum_aquaticum]	ptg004746l	75	1.56E-29	112	100
KDA54669.1_methionine_aminopeptidase_[Thermoanaerobaculum_aquaticum]	ptg002436l	47.154	3.93E-73	245	98
KDA54668.1_adenylate_kinase_[Thermoanaerobaculum_aquaticum]	LG12	49.057	1.43E-08	58.2	24
KDA54667.1_preprotein_translocase_subunit_SecY_[Thermoanaerobaculum_aquaticum]	ptg004509l	44.69	6.51E-111	363	97
KDA54666.1_50S_ribosomal_protein_L15_[Thermoanaerobaculum_aquaticum]	ptg005891l	55.385	1.05E-32	125	87
KDA54664.1_30S_ribosomal_protein_S5_[Thermoanaerobaculum_aquaticum]	ptg004509l	56.489	6.65E-39	144	74
KDA54663.1_50S_ribosomal_protein_L18_[Thermoanaerobaculum_aquaticum]	ptg005254l	52.525	2.03E-27	108	81
KDA54662.1_50S_ribosomal_protein_L6_[Thermoanaerobaculum_aquaticum]	ptg001624l	44.134	1.71E-37	140	99

KDA54661.1_30S_ribosomal_protein_S8_[Thermoanaerobaculum_aquaticum]	ptg004509l	54.545	2.39E-33	126	100
KDA54660.1_hypothetical_protein_EG19_09545_[Thermoanaerobaculum_aquaticum]	ptg005254l	68.182	5.21E-13	64.7	72
KDA54659.1_50S_ribosomal_protein_L5_[Thermoanaerobaculum_aquaticum]	ptg004509l	54.237	2.42E-63	214	98
KDA54658.1_50S_ribosomal_protein_L24_[Thermoanaerobaculum_aquaticum]	ptg004509l	50.485	2.78E-27	107	95
KDA54657.1_50S_ribosomal_protein_L14_[Thermoanaerobaculum_aquaticum]	ptg004509l	61.789	6.01E-44	155	100
KDA54656.1_30S_ribosomal_protein_S17_[Thermoanaerobaculum_aquaticum]	ptg004509l	44.595	7.73E-14	68.2	85
KDA54655.1_50S_ribosomal_protein_L29_[Thermoanaerobaculum_aquaticum]	ptg004509l	46.667	4.54E-10	56.6	92
KDA54654.1_50S_ribosomal_protein_L16_[Thermoanaerobaculum_aquaticum]	ptg001624l	61.062	6.28E-44	156	82
KDA54653.1_30S_ribosomal_protein_S3_[Thermoanaerobaculum_aquaticum]	ptg004509l	57.488	5.14E-61	209	96
KDA54652.1_hypothetical_protein_EG19_09505_[Thermoanaerobaculum_aquaticum]	ptg004509l	46.364	1.1E-19	86.3	97
KDA54651.1_30S_ribosomal_protein_S19_[Thermoanaerobaculum_aquaticum]	ptg004509l	60	2.44E-32	121	96
KDA54650.1_50S_ribosomal_protein_L2_[Thermoanaerobaculum_aquaticum]	ptg005891l	62.182	3.71E-95	310	100
KDA54649.1_50S_ribosomal_protein_L23_[Thermoanaerobaculum_aquaticum]	ptg004509l	42.857	2.45E-15	73.2	86
KDA54648.1_hypothetical_protein_EG19_09485_[Thermoanaerobaculum_aquaticum]	ptg004509l	39.894	1.94E-29	118	90
KDA54647.1_50S_ribosomal_protein_L3_[Thermoanaerobaculum_aquaticum]	ptg001624l	42.647	6.44E-33	128	99
KDA54646.1_30S_ribosomal_protein_S10_[Thermoanaerobaculum_aquaticum]	ptg005314l	78.218	4.6E-47	164	94
KDA54645.1_elongation_factor_Tu,_partial_[Thermoanaerobaculum_aquaticum]	ptg002214l	68.273	5.32E-112	357	99
KDA54642.1_hypothetical_protein_EG19_10810_[Thermoanaerobaculum_aquaticum]	ptg004055l	30.699	6.03E-45	170	90
KDA54641.1_hypothetical_protein_EG19_10805_[Thermoanaerobaculum_aquaticum]	ptg002534l	27.703	0.000000811	55.1	41
KDA54637.1_hypothetical_protein_EG19_10785_[Thermoanaerobaculum_aquaticum]	ptg005204l	37.5	7.14E-09	59.7	42
KDA54635.1_hypothetical_protein_EG19_10775_[Thermoanaerobaculum_aquaticum]	ptg005844l	32.143	4.06E-11	67.8	50
KDA54634.1_hypothetical_protein_EG19_10770_[Thermoanaerobaculum_aquaticum]	ptg003947l	36.364	5.37E-36	137	94
KDA54633.1_hypothetical_protein_EG19_10765_[Thermoanaerobaculum_aquaticum]	ptg004476l	25.185	3.59E-08	61.6	32
KDA54631.1_hypothetical_protein_EG19_10755_[Thermoanaerobaculum_aquaticum]	ptg003137l	44.805	4.82E-57	208	62
KDA54627.1_hypothetical_protein_EG19_10730_[Thermoanaerobaculum_aquaticum]	ptg004476l	26.482	2.63E-11	68.6	76
KDA54622.1_threonine_aldolase_[Thermoanaerobaculum_aquaticum]	LG13	61.538	2.84E-10	65.9	25
KDA54621.1_hypothetical_protein_EG19_10700_[Thermoanaerobaculum_aquaticum]	ptg005922l	32.92	2.36E-57	211	99
KDA54620.1_hypothetical_protein_EG19_10695_[Thermoanaerobaculum_aquaticum]	ptg005354l	36.552	4.71E-16	79.7	61
KDA54619.1_hypothetical_protein_EG19_10685_[Thermoanaerobaculum_aquaticum]	ptg004532l	42.045	4.37E-43	156	91
KDA54618.1_hypothetical_protein_EG19_10680_[Thermoanaerobaculum_aquaticum]	ptg003703l	40.594	2.58E-87	293	94
KDA54617.1_deoxyribose-phosphate_aldolase_[Thermoanaerobaculum_aquaticum]	ptg005596l	34.146	1.58E-10	63.9	82
KDA54605.1_hypothetical_protein_EG19_10615_[Thermoanaerobaculum_aquaticum]	ptg001814l	48.718	1.74E-36	136	89
KDA54604.1_hypothetical_protein_EG19_10610_[Thermoanaerobaculum_aquaticum]	ptg002909l	44.156	6.3E-25	105	69
KDA54602.1_hypothetical_protein_EG19_10595_[Thermoanaerobaculum_aquaticum]	ptg005354l	25.746	3.15E-18	91.7	58
KDA54598.1_hypothetical_protein_EG19_10575_[Thermoanaerobaculum_aquaticum]	ptg005354l	25.532	0.000000151	56.6	85

KDA54596.1_hypothetical_protein_EG19_10565_[Thermoanaerobaculum_aquaticum]	ptg005527l	22.143	0.00000013	56.6	70
KDA54594.1_hypothetical_protein_EG19_10555_[Thermoanaerobaculum_aquaticum]	ptg005354l	28.058	5.48E-18	89.4	75
KDA54592.1_hypothetical_protein_EG19_10535_[Thermoanaerobaculum_aquaticum]	ptg003057l	31.056	3.13E-28	123	58
KDA54591.1_hypothetical_protein_EG19_10530_[Thermoanaerobaculum_aquaticum]	ptg002270l	25.758	5.07E-11	67	87
KDA54588.1_hypothetical_protein_EG19_10505_[Thermoanaerobaculum_aquaticum]	ptg002214l	22.928	5.44E-15	81.6	59
KDA54587.1_hypothetical_protein_EG19_10500_[Thermoanaerobaculum_aquaticum]	ptg000732l	25.98	7.78E-11	67.8	52
KDA54586.1_hypothetical_protein_EG19_10495_[Thermoanaerobaculum_aquaticum]	ptg001659l	51.835	8.22E-63	215	94
KDA54585.1_hypothetical_protein_EG19_10490_[Thermoanaerobaculum_aquaticum]	ptg005920l	31.975	1.02E-25	114	70
KDA54584.1_hypothetical_protein_EG19_10485_[Thermoanaerobaculum_aquaticum]	ptg005920l	26.84	3.18E-14	79	51
KDA54581.1_hypothetical_protein_EG19_10470_[Thermoanaerobaculum_aquaticum]	ptg002760l	34.815	1.44E-39	152	78
KDA54579.1_hypothetical_protein_EG19_10460_[Thermoanaerobaculum_aquaticum]	ptg004982l	31.618	2.56E-22	100	91
KDA54577.1_peptide_chain_release_factor_1_[Thermoanaerobaculum_aquaticum]	ptg004982l	48.295	4.37E-93	308	94
KDA54576.1_50S_ribosomal_protein_L31_[Thermoanaerobaculum_aquaticum]	ptg004959l	53.846	6.47E-20	84.7	96
KDA54575.1_hypothetical_protein_EG19_10440_[Thermoanaerobaculum_aquaticum]	ptg003691l	52.308	0.000000709	53.5	27
KDA54574.1_hypothetical_protein_EG19_10435_[Thermoanaerobaculum_aquaticum]	ptg005010l	34.982	2.62E-40	152	94
KDA54573.1_hypothetical_protein_EG19_10430_[Thermoanaerobaculum_aquaticum]	ptg001005l	30.816	7.35E-18	89.7	82
KDA54572.1_hypothetical_protein_EG19_10425_[Thermoanaerobaculum_aquaticum]	ptg002658l	34.429	6.42E-69	246	94
KDA54569.1_hypothetical_protein_EG19_10410_[Thermoanaerobaculum_aquaticum]	ptg004476l	29.197	5.17E-11	65.1	64
KDA54568.1_hypothetical_protein_EG19_10405_[Thermoanaerobaculum_aquaticum]	ptg004476l	26.502	9.89E-12	70.1	81
KDA54567.1_protein-L-isoaspartate_O-methyltransferase_[Thermoanaerobaculum_aquaticum]	ptg004026l	49.302	3.55E-56	196	89
KDA54566.1_hypothetical_protein_EG19_10395_[Thermoanaerobaculum_aquaticum]	ptg003746l	29.717	1.68E-10	68.2	39
KDA54565.1_hypothetical_protein_EG19_10390_[Thermoanaerobaculum_aquaticum]	ptg001690l	43.046	7.04E-58	206	84
KDA54562.1_hypothetical_protein_EG19_10370_[Thermoanaerobaculum_aquaticum]	ptg001766l	29.722	1.68E-27	119	88
KDA54561.1_cation_transporter_[Thermoanaerobaculum_aquaticum]	ptg004670l	40.811	0	676	99
KDA54558.1_hypothetical_protein_EG19_10350_[Thermoanaerobaculum_aquaticum]	ptg003057l	39.062	6.15E-83	282	96
KDA54557.1_hypothetical_protein_EG19_10345_[Thermoanaerobaculum_aquaticum]	ptg003903l	31.734	5.11E-21	102	42
KDA54556.1_hypothetical_protein_EG19_10340_[Thermoanaerobaculum_aquaticum]	ptg001034l	38.806	0.000000127	52.8	50
KDA54552.1_hypothetical_protein_EG19_10320_[Thermoanaerobaculum_aquaticum]	ptg001262l	48.159	0	605	99
KDA54551.1_hypothetical_protein_EG19_10315_[Thermoanaerobaculum_aquaticum]	ptg004026l	48.088	0	649	93
KDA54550.1_30S_ribosomal_protein_S15_[Thermoanaerobaculum_aquaticum]	ptg004026l	46.429	5.95E-21	88.6	92
KDA54548.1_DNA_methyltransferase_[Thermoanaerobaculum_aquaticum]	ptg002453l	28.102	2.36E-24	107	79
KDA54545.1_hypothetical_protein_EG19_10285_[Thermoanaerobaculum_aquaticum]	ptg003078l	40.157	1.1E-45	169	81
KDA54544.1_hypothetical_protein_EG19_10280_[Thermoanaerobaculum_aquaticum]	ptg004026l	32.534	3.2E-24	107	90
KDA54543.1_hypothetical_protein_EG19_10275_[Thermoanaerobaculum_aquaticum]	ptg001690l	35.398	9.61E-18	81.3	92
KDA54541.1_hypothetical_protein_EG19_10265_[Thermoanaerobaculum_aquaticum]	ptg001690l	45.44	2.6E-147	486	65

KDA54540.1_hypothetical_protein_EG19_10260_[Thermoanaerobaculum_aquaticum]	ptg001571l	34.536	6.83E-55	202	80
KDA54538.1_malate_dehydrogenase_[Thermoanaerobaculum_aquaticum]	ptg003019l	48.052	3.46E-81	271	96
KDA54537.1_endonuclease_III_[Thermoanaerobaculum_aquaticum]	ptg005275l	40.994	1.58E-36	139	74
KDA54534.1_hypothetical_protein_EG19_10225_[Thermoanaerobaculum_aquaticum]	ptg002505l	27.419	0.00000291	52.8	52
KDA54533.1_hypothetical_protein_EG19_10220_[Thermoanaerobaculum_aquaticum]	ptg003058l	48.148	8.21E-19	82	100
KDA54532.1_hypothetical_protein_EG19_10215_[Thermoanaerobaculum_aquaticum]	ptg005820l	32.479	2.35E-08	62	15
KDA54527.1_phosphoribosylglycinamide_formyltransferase_[Thermoanaerobaculum_aquaticum]	LG04	41.872	6.63E-37	139	95
KDA54526.1_phosphoribosylaminoimidazole_synthetase_[Thermoanaerobaculum_aquaticum]	ptg005227l	44.51	4.12E-83	278	96
KDA54524.1_amidophosphoribosyltransferase_[Thermoanaerobaculum_aquaticum]	ptg000484l	37.815	3.27E-78	269	97
KDA54523.1_phosphoribosylformylglycinamidine_synthase_[Thermoanaerobaculum_aquaticum]	ptg002878l	31.744	2.63E-82	290	98
KDA54522.1_phosphoribosylformylglycinamidine_synthase_[Thermoanaerobaculum_aquaticum]	ptg005375l	35.319	4.85E-32	126	93
KDA54520.1_hypothetical_protein_EG19_11155_[Thermoanaerobaculum_aquaticum]	ptg004509l	38.073	5.35E-41	153	85
KDA54519.1_deoxyuridine_5'-triphosphate_nucleotidohydrolase_[Thermoanaerobaculum_aquaticum]	ptg002199l	41.176	2.79E-27	109	91
KDA54518.1_hypothetical_protein_EG19_11145_[Thermoanaerobaculum_aquaticum]	ptg001828l	24.814	1.93E-27	120	85
KDA54517.1_hypothetical_protein_EG19_11140_[Thermoanaerobaculum_aquaticum]	ptg004519l	25.556	5.17E-08	60.1	43
KDA54514.1_hypothetical_protein_EG19_11120_[Thermoanaerobaculum_aquaticum]	ptg004779l	30.742	3.38E-25	113	58
KDA54513.1_transcriptional_regulator_[Thermoanaerobaculum_aquaticum]	ptg004779l	38.393	1.97E-35	136	97
KDA54508.1_hypothetical_protein_EG19_11085_[Thermoanaerobaculum_aquaticum]	ptg004827l	20.052	5.62E-11	70.5	49
KDA54503.1_pilus_assembly_protein_PilB_[Thermoanaerobaculum_aquaticum]	ptg000568l	42.505	2.47E-120	394	95
KDA54502.1_hypothetical_protein_EG19_11055_[Thermoanaerobaculum_aquaticum]	ptg005301l	31.965	5.56E-46	173	85
KDA54499.1_hypothetical_protein_EG19_11040_[Thermoanaerobaculum_aquaticum]	ptg004479l	33.14	7.62E-22	96.3	80
KDA54498.1_hypothetical_protein_EG19_11035_[Thermoanaerobaculum_aquaticum]	ptg001658l	36.461	2.69E-50	186	88
KDA54497.1_hypothetical_protein_EG19_11030_[Thermoanaerobaculum_aquaticum]	ptg004746l	32.362	5.86E-30	124	90
KDA54496.1_hypothetical_protein_EG19_11025_[Thermoanaerobaculum_aquaticum]	ptg002916l	34.835	5.3E-25	109	97
KDA54495.1_ADP-heptose_synthase_[Thermoanaerobaculum_aquaticum]	ptg002916l	43.333	6.83E-30	117	90
KDA54494.1_hypothetical_protein_EG19_11015_[Thermoanaerobaculum_aquaticum]	ptg004974l	31.529	3.88E-26	113	86
KDA54492.1_type_II_secretion_system_protein_E_[Thermoanaerobaculum_aquaticum]	ptg004468l	44.742	5.84E-134	434	98
KDA54491.1_twitching_motility_protein_pilT_[Thermoanaerobaculum_aquaticum]	LG13	48.991	3.94E-97	320	93
KDA54490.1_pilus_assembly_protein_PilC_[Thermoanaerobaculum_aquaticum]	ptg005301l	39.85	4.03E-88	295	99
KDA54489.1_hypothetical_protein_EG19_10990_[Thermoanaerobaculum_aquaticum]	ptg003057l	30.29	4.84E-26	117	39
KDA54488.1_hypothetical_protein_EG19_10985_[Thermoanaerobaculum_aquaticum]	ptg002304l	41.703	3.98E-103	340	99
KDA54486.1_UTP--glucose-1-phosphate_uridylyltransferase_[Thermoanaerobaculum_aquaticum]	ptg002505l	44.487	5.29E-68	233	90
KDA54485.1_hypothetical_protein_EG19_10970_[Thermoanaerobaculum_aquaticum]	ptg004942l	37.143	3.94E-08	52.4	72
KDA54473.1_hypothetical_protein_EG19_12215_[Thermoanaerobaculum_aquaticum]	ptg003813l	32.545	8.79E-60	218	92

KDA54472.1_hypothetical_protein_EG19_12210_[Thermoanaerobaculum_aquaticum]	ptg005274l	43.675	2.22E-80	274	95
KDA54471.1_ferritin_[Thermoanaerobaculum_aquaticum]	ptg001693l	47.403	9.37E-40	145	94
KDA54470.1_hypothetical_protein_EG19_12200_[Thermoanaerobaculum_aquaticum]	ptg004136l	26.45	2.53E-25	117	46
KDA54466.1_fructose_1,6-bisphosphatase_[Thermoanaerobaculum_aquaticum]	ptg004284l	46.377	4.43E-56	199	84
KDA54464.1_hypothetical_protein_EG19_12170_[Thermoanaerobaculum_aquaticum]	ptg003913l	42.857	2.83E-114	374	99
KDA54463.1_hypothetical_protein_EG19_12165_[Thermoanaerobaculum_aquaticum]	ptg005905l	33.884	1.68E-27	114	91
KDA54461.1_hypothetical_protein_EG19_12155_[Thermoanaerobaculum_aquaticum]	ptg003334l	34.091	0.000000251	52.4	56
KDA54459.1_hypothetical_protein_EG19_12145_[Thermoanaerobaculum_aquaticum]	ptg004055l	41.631	3.31E-45	167	73
KDA54458.1_hypothetical_protein_EG19_12140_[Thermoanaerobaculum_aquaticum]	ptg002760l	33.628	0.000000209	53.1	64
KDA54457.1_hypothetical_protein_EG19_12135_[Thermoanaerobaculum_aquaticum]	ptg003045l	27.311	2.04E-16	84	73
KDA54455.1_hypothetical_protein_EG19_12125_[Thermoanaerobaculum_aquaticum]	ptg002316l	38.393	8.81E-20	95.1	31
KDA54454.1_hypothetical_protein_EG19_12120_[Thermoanaerobaculum_aquaticum]	ptg002021l	31.646	0.000000666	56.6	25
KDA54452.1_hypothetical_protein_EG19_12110_[Thermoanaerobaculum_aquaticum]	ptg000819l	41.29	4.64E-26	115	34
KDA54451.1_hypothetical_protein_EG19_12105_[Thermoanaerobaculum_aquaticum]	ptg003213l	32.374	0.00000428	52	46
KDA54450.1_hypothetical_protein_EG19_12100_[Thermoanaerobaculum_aquaticum]	ptg003112l	35.878	9.03E-18	89.4	33
KDA54449.1_molecular_chaperone_GroES_[Thermoanaerobaculum_aquaticum]	ptg004085l	48.352	1.32E-21	90.9	95
KDA54448.1_molecular_chaperone_GroEL_[Thermoanaerobaculum_aquaticum]	ptg002031l	61.29	0	616	97
KDA54447.1_translation_elongation_factor_P_[Thermoanaerobaculum_aquaticum]	ptg003382l	30.645	9.63E-23	98.2	98
KDA54441.1_hypothetical_protein_EG19_12050_[Thermoanaerobaculum_aquaticum]	LG16	28.571	3.11E-20	98.6	51
KDA54439.1_hypothetical_protein_EG19_12040_[Thermoanaerobaculum_aquaticum]	ptg005903l	28.571	1.49E-14	81.3	42
KDA54438.1_aspartate_aminotransferase_[Thermoanaerobaculum_aquaticum]	ptg004905l	33.146	4.79E-42	161	90
KDA54429.1_S-adenosyl-L-homocysteine_hydrolase_[Thermoanaerobaculum_aquaticum]	ptg002467l	42.259	2.48E-88	298	99
KDA54428.1_hypothetical_protein_EG19_11985_[Thermoanaerobaculum_aquaticum]	ptg005886l	38.095	3.51E-13	74.7	41
KDA54427.1_hypothetical_protein_EG19_11980_[Thermoanaerobaculum_aquaticum]	LG28	39.837	4.75E-12	68.9	50
KDA54426.1_hypothetical_protein_EG19_11975_[Thermoanaerobaculum_aquaticum]	ptg000568l	37.838	3.2E-14	73.2	79
KDA54425.1_hypothetical_protein_EG19_11970_[Thermoanaerobaculum_aquaticum]	ptg002270l	36.546	3.68E-39	148	99
KDA54424.1_hypothetical_protein_EG19_11965_[Thermoanaerobaculum_aquaticum]	ptg005121l	27.682	1.4E-24	110	67
KDA54423.1_hypothetical_protein_EG19_11960_[Thermoanaerobaculum_aquaticum]	ptg003223l	28.177	7.18E-12	66.6	95
KDA54422.1_hypothetical_protein_EG19_11955_[Thermoanaerobaculum_aquaticum]	LG04	45.745	1.34E-11	66.2	48
KDA54421.1_hypothetical_protein_EG19_11950_[Thermoanaerobaculum_aquaticum]	ptg001005l	38.066	1.77E-46	172	99
KDA54420.1_hypothetical_protein_EG19_11945_[Thermoanaerobaculum_aquaticum]	ptg006031l	32.743	1.36E-27	116	66
KDA54419.1_hypothetical_protein_EG19_11940_[Thermoanaerobaculum_aquaticum]	ptg005614l	26.244	6.32E-13	73.2	70
KDA54418.1_signal_recognition_particle_[Thermoanaerobaculum_aquaticum]	ptg002916l	47.209	6.25E-95	317	97
KDA54417.1_30S_ribosomal_protein_S16_[Thermoanaerobaculum_aquaticum]	ptg005431l	45	1.07E-16	75.9	100
KDA54414.1_hypothetical_protein_EG19_11915_[Thermoanaerobaculum_aquaticum]	ptg005017l	47.143	3.27E-60	208	81
KDA54413.1_50S_ribosomal_protein_L19_[Thermoanaerobaculum_aquaticum]	ptg005431l	54.955	3.79E-30	116	97

KDA54412.1_hypothetical_protein_EG19_11905_[Thermoanaerobaculum_aquaticum]	ptg002436l	43.889	5.82E-37	139	95
KDA54404.1_hypothetical_protein_EG19_11865_[Thermoanaerobaculum_aquaticum]	ptg005839l	39.024	1.93E-51	186	90
KDA54402.1_hypothetical_protein_EG19_11855_[Thermoanaerobaculum_aquaticum]	ptg004982l	40	6.13E-17	82.8	24
KDA54400.1_hypothetical_protein_EG19_11845_[Thermoanaerobaculum_aquaticum]	ptg004982l	30.282	4.4E-19	91.7	90
KDA54399.1_hypothetical_protein_EG19_11840_[Thermoanaerobaculum_aquaticum]	ptg003363l	41.667	0.00000022	51.2	50
KDA54392.1_hypothetical_protein_EG19_11800_[Thermoanaerobaculum_aquaticum]	ptg004277l	25.874	0.00000223	49.3	70
KDA54390.1_inosine-5-monophosphate_dehydrogenase_[Thermoanaerobaculum_aquaticum]	ptg002610l	57.143	4.4E-174	546	99
KDA54387.1_hypothetical_protein_EG19_11775_[Thermoanaerobaculum_aquaticum]	ptg001290l	41.47	1.63E-79	282	49
KDA54386.1_hypothetical_protein_EG19_11770_[Thermoanaerobaculum_aquaticum]	ptg005886l	36.236	1.51E-53	193	91
KDA54385.1_hypothetical_protein_EG19_11765_[Thermoanaerobaculum_aquaticum]	ptg004468l	51.744	1.74E-104	346	69
KDA54384.1_twitching_motility_protein_PilT_[Thermoanaerobaculum_aquaticum]	LG13	47.429	2.39E-77	264	90
KDA54382.1_alanyl-tRNA_synthetase_[Thermoanaerobaculum_aquaticum]	ptg002916l	50.204	0	459	96
KDA54381.1_hypothetical_protein_EG19_11745_[Thermoanaerobaculum_aquaticum]	ptg005110l	37.113	1.88E-11	67	40
KDA54380.1_tryptophanyl-tRNA_synthetase_[Thermoanaerobaculum_aquaticum]	ptg003327l	50.311	9.19E-103	334	99
KDA54377.1_acyl-CoA_dehydrogenase_[Thermoanaerobaculum_aquaticum]	LG02	49.038	1.36E-21	101	65
KDA54375.1_hypothetical_protein_EG19_11715_[Thermoanaerobaculum_aquaticum]	ptg004732l	31.6	1.54E-29	119	95
KDA54374.1_hypothetical_protein_EG19_11710_[Thermoanaerobaculum_aquaticum]	ptg003980l	38.275	8.51E-49	180	99
KDA54373.1_4-hydroxybutyrate_CoA-transferase_[Thermoanaerobaculum_aquaticum]	LG22	70.588	1.25E-12	73.9	24
KDA54371.1_gamma-glutamyltransferase_[Thermoanaerobaculum_aquaticum]	ptg001595l	56.261	0	593	97
KDA54370.1_hypothetical_protein_EG19_11690_[Thermoanaerobaculum_aquaticum]	ptg005484l	29.496	2.7E-09	65.5	15
KDA54369.1_phosphate_starvation_protein_PhoH_[Thermoanaerobaculum_aquaticum]	ptg005772l	46.689	5.63E-78	262	95
KDA54368.1_hypothetical_protein_EG19_11675_[Thermoanaerobaculum_aquaticum]	ptg004524l	44.444	5.38E-56	212	40
KDA54367.1_hypothetical_protein_EG19_11670_[Thermoanaerobaculum_aquaticum]	ptg004790l	34	3.38E-12	66.2	70
KDA54366.1_hypothetical_protein_EG19_11665_[Thermoanaerobaculum_aquaticum]	ptg002760l	39	1.15E-26	117	47
KDA54365.1_hypothetical_protein_EG19_11660_[Thermoanaerobaculum_aquaticum]	ptg002902l	38.854	2.63E-22	100	51
KDA54360.1_hypothetical_protein_EG19_11635_[Thermoanaerobaculum_aquaticum]	ptg002907l	38.136	3.49E-12	65.5	85
KDA54357.1_hypothetical_protein_EG19_11620_[Thermoanaerobaculum_aquaticum]	ptg002909l	28.879	1.71E-21	98.2	71
KDA54356.1_hypothetical_protein_EG19_11615_[Thermoanaerobaculum_aquaticum]	ptg004579l	32.287	2.07E-25	107	95
KDA54355.1_alcohol_dehydrogenase_[Thermoanaerobaculum_aquaticum]	ptg000693l	35.638	7.49E-13	73.6	55
KDA54353.1_acetyl-CoA_acetyltransferase_[Thermoanaerobaculum_aquaticum]	ptg005527l	44.73	1.71E-88	295	99
KDA54352.1_hypothetical_protein_EG19_11595_[Thermoanaerobaculum_aquaticum]	ptg002760l	28.115	1.72E-22	108	38
KDA54350.1_hypothetical_protein_EG19_11580_[Thermoanaerobaculum_aquaticum]	ptg004917l	26.667	1.28E-09	63.9	61
KDA54349.1_hypothetical_protein_EG19_11575_[Thermoanaerobaculum_aquaticum]	ptg004111l	29.612	1.46E-63	231	99
KDA54346.1_hypothetical_protein_EG19_11560_[Thermoanaerobaculum_aquaticum]	ptg004372l	34.591	1.37E-20	93.6	61
KDA54345.1_hypothetical_protein_EG19_11555_[Thermoanaerobaculum_aquaticum]	ptg004476l	29.032	1.23E-13	77	58

KDA54343.1_hypothetical_protein_EG19_11545_[Thermoanaerobaculum_aquaticum]	ptg004735l	26.19	3.36E-12	68.6	59
KDA54341.1_enoyl-CoA_hydratase_[Thermoanaerobaculum_aquaticum]	ptg002612l	46.124	8.81E-52	184	97
KDA54340.1_hypothetical_protein_EG19_11530_[Thermoanaerobaculum_aquaticum]	ptg002871l	26.329	4.75E-19	93.6	94
KDA54339.1_hypothetical_protein_EG19_11525_[Thermoanaerobaculum_aquaticum]	ptg006032l	34.842	5.98E-26	119	27
KDA54337.1_succinyl-CoA_synthetase_subunit_beta_[Thermoanaerobaculum_aquaticum]	ptg002316l	52.941	4.36E-125	401	99
KDA54336.1_hypothetical_protein_EG19_11510_[Thermoanaerobaculum_aquaticum]	ptg002316l	59.794	2.57E-98	320	100
KDA54335.1_nucleoside_diphosphate_kinase_[Thermoanaerobaculum_aquaticum]	ptg002916l	51.799	5.61E-43	154	100
KDA54332.1_hypothetical_protein_EG19_11490_[Thermoanaerobaculum_aquaticum]	ptg003673l	38.125	6.52E-18	93.6	17
KDA54331.1_short-chain_dehydrogenase_[Thermoanaerobaculum_aquaticum]	ptg005692l	35.361	1.94E-23	103	91
KDA54318.1_glycosyl_transferase_[Thermoanaerobaculum_aquaticum]	ptg004788l	41.629	5.76E-42	155	93
KDA54317.1_hypothetical_protein_EG19_11415_[Thermoanaerobaculum_aquaticum]	LG10	32.843	2.96E-20	92.4	84
KDA54315.1_hypothetical_protein_EG19_11395_[Thermoanaerobaculum_aquaticum]	ptg001925l	50.649	1.72E-19	86.3	61
KDA54312.1_pyruvate_phosphate_dikinase_[Thermoanaerobaculum_aquaticum]	ptg005844l	54.686	0	949	98
KDA54310.1_hypothetical_protein_EG19_11360_[Thermoanaerobaculum_aquaticum]	ptg001604l	27.976	9.16E-15	80.9	54
KDA54308.1_hypothetical_protein_EG19_11350_[Thermoanaerobaculum_aquaticum]	ptg005341l	35.115	1.41E-16	78.6	91
KDA54307.1_hypothetical_protein_EG19_11345_[Thermoanaerobaculum_aquaticum]	ptg005341l	34.337	2.23E-19	89.7	74
KDA54305.1_hypothetical_protein_EG19_11335_[Thermoanaerobaculum_aquaticum]	ptg004854l	28.351	1.29E-10	63.5	92
KDA54303.1_hypothetical_protein_EG19_11320_[Thermoanaerobaculum_aquaticum]	ptg002381l	25.225	1.55E-08	58.5	88
KDA54301.1_hypothetical_protein_EG19_11310_[Thermoanaerobaculum_aquaticum]	ptg004854l	29.118	6.72E-21	100	70
KDA54299.1_hypothetical_protein_EG19_11300_[Thermoanaerobaculum_aquaticum]	ptg004854l	28.571	6.45E-18	91.7	60
KDA54297.1_hypothetical_protein_EG19_11290_[Thermoanaerobaculum_aquaticum]	ptg004854l	28.755	2.7E-14	78.6	57
KDA54296.1_hypothetical_protein_EG19_11285_[Thermoanaerobaculum_aquaticum]	ptg004256l	34.783	2.51E-10	64.7	34
KDA54294.1_hypothetical_protein_EG19_11275_[Thermoanaerobaculum_aquaticum]	ptg003290l	40.881	2.74E-25	105	91
KDA54293.1_hypothetical_protein_EG19_11270_[Thermoanaerobaculum_aquaticum]	ptg004297l	29.323	1.79E-08	54.7	97
KDA54291.1_hypothetical_protein_EG19_11260_[Thermoanaerobaculum_aquaticum]	ptg004384l	27.857	5.35E-24	105	92
KDA54290.1_hypothetical_protein_EG19_11255_[Thermoanaerobaculum_aquaticum]	ptg004790l	30.258	1.02E-26	118	52
KDA54289.1_peptide_chain_release_factor_2_[Thermoanaerobaculum_aquaticum]	ptg004479l	52.852	1.92E-79	266	84
KDA54288.1_hypothetical_protein_EG19_11245_[Thermoanaerobaculum_aquaticum]	ptg004384l	29.323	0.00000515	52.8	31
KDA54287.1_hypothetical_protein_EG19_11240_[Thermoanaerobaculum_aquaticum]	ptg005213l	41.176	7.35E-19	87	61
KDA54285.1_hypothetical_protein_EG19_11230_[Thermoanaerobaculum_aquaticum]	ptg003082l	33.333	3.7E-42	165	57
KDA54283.1_hypothetical_protein_EG19_11220_[Thermoanaerobaculum_aquaticum]	ptg005030l	40.5	2.43E-35	135	90
KDA54282.1_hypothetical_protein_EG19_11215_[Thermoanaerobaculum_aquaticum]	ptg004143l	32.849	3.12E-26	116	70
KDA54281.1_NADH_dehydrogenase_[Thermoanaerobaculum_aquaticum]	ptg004143l	47.404	4.09E-110	363	89
KDA54279.1_hypothetical_protein_EG19_12425_[Thermoanaerobaculum_aquaticum]	ptg005484l	33.929	3.35E-37	155	42
KDA54278.1_hypothetical_protein_EG19_12420_[Thermoanaerobaculum_aquaticum]	ptg005318l	30.208	3.93E-08	57	43

KDA54276.1_hypothetical_protein_EG19_12400_[Thermoanaerobaculum_aquaticum]	ptg004084l	37.086	1.95E-24	106	53
KDA54273.1_Fis_family_transcriptional_regulator_[Thermoanaerobaculum_aquaticum]	ptg003137l	43.177	7.16E-113	369	97
KDA54272.1_hypothetical_protein_EG19_12380_[Thermoanaerobaculum_aquaticum]	ptg002453l	29.796	1.55E-23	109	45
KDA54266.1_nitrate_reductase_[Thermoanaerobaculum_aquaticum]	ptg004306l	34.306	1.49E-113	382	95
KDA54262.1_hypothetical_protein_EG19_12330_[Thermoanaerobaculum_aquaticum]	ptg001921l	43.204	2.42E-50	185	56
KDA54261.1_hypothetical_protein_EG19_12325_[Thermoanaerobaculum_aquaticum]	ptg004676l	36.047	0.00000288	49.3	53
KDA54260.1_hypothetical_protein_EG19_12320_[Thermoanaerobaculum_aquaticum]	ptg004564l	38.511	3.47E-81	277	98
KDA54259.1_hypothetical_protein_EG19_12315_[Thermoanaerobaculum_aquaticum]	ptg001025l	25.672	1.75E-16	84.7	90
KDA54256.1_hypothetical_protein_EG19_12300_[Thermoanaerobaculum_aquaticum]	ptg005274l	34.602	1.04E-43	172	45
KDA54255.1_ferrochelatase_[Thermoanaerobaculum_aquaticum]	ptg001995l	47.748	4.24E-105	342	91
KDA54253.1_hypothetical_protein_EG19_12285_[Thermoanaerobaculum_aquaticum]	ptg003199l	28.507	6.46E-14	73.9	89
KDA54252.1_hypothetical_protein_EG19_12280_[Thermoanaerobaculum_aquaticum]	ptg001025l	33.092	1.05E-45	174	85
KDA54251.1_hypothetical_protein_EG19_12275_[Thermoanaerobaculum_aquaticum]	ptg004494l	32.766	5.69E-20	98.6	41
KDA54250.1_hypothetical_protein_EG19_12270_[Thermoanaerobaculum_aquaticum]	LG03	37.553	4.84E-43	158	99
KDA54249.1_hypothetical_protein_EG19_12265_[Thermoanaerobaculum_aquaticum]	ptg002567l	55.823	8.92E-90	293	98
KDA54248.1_hypothetical_protein_EG19_12260_[Thermoanaerobaculum_aquaticum]	ptg004075l	41.206	2.4E-34	133	88
KDA54247.1_hypothetical_protein_EG19_12255_[Thermoanaerobaculum_aquaticum]	ptg002567l	54.656	2.71E-85	280	98
KDA54246.1_hypothetical_protein_EG19_12250_[Thermoanaerobaculum_aquaticum]	ptg002567l	63.74	1.16E-83	286	52
KDA54245.1_hypothetical_protein_EG19_12245,_partial_[Thermoanaerobaculum_aquaticum]	ptg002567l	35.577	6.48E-52	124	68
KDA54244.1_hypothetical_protein_EG19_00475_[Thermoanaerobaculum_aquaticum]	ptg005484l	34.197	4.86E-55	210	49
KDA54243.1_tryptophanase_[Thermoanaerobaculum_aquaticum]	ptg004361l	56.637	9.08E-168	527	99
KDA54242.1_hypothetical_protein_EG19_00460_[Thermoanaerobaculum_aquaticum]	ptg000834l	33.824	3.63E-13	70.9	70
KDA54240.1_hypothetical_protein_EG19_00450_[Thermoanaerobaculum_aquaticum]	ptg002505l	44.872	0.000000386	49.7	81
KDA54239.1_phosphoribosylaminoimidazole-succinocarboxamide_synthase_[Thermoanaerobaculum_aquaticum]	ptg004573l	45.918	1.93E-43	91.3	77
KDA54238.1_elongation_factor_G_[Thermoanaerobaculum_aquaticum]	ptg002658l	35.043	1.62E-132	436	97
KDA54235.1_UDP-glucose_4-epimerase_[Thermoanaerobaculum_aquaticum]	ptg003768l	34.385	4.52E-47	173	99
KDA54233.1_hypothetical_protein_EG19_00415_[Thermoanaerobaculum_aquaticum]	ptg002557l	30.769	3.56E-19	91.3	77
KDA54232.1_pterin-4-alpha-carbinolamine_dehydratase_[Thermoanaerobaculum_aquaticum]	ptg000492l	41.905	2.21E-21	90.9	92
KDA54231.1_hypothetical_protein_EG19_00405_[Thermoanaerobaculum_aquaticum]	ptg002907l	32.847	2.51E-81	286	94
KDA54229.1_hypothetical_protein_EG19_00395_[Thermoanaerobaculum_aquaticum]	ptg005035l	27.61	4.35E-52	192	97
KDA54228.1_methylmalonyl-CoA_mutase_[Thermoanaerobaculum_aquaticum]	LG30	47.297	6.14E-09	56.6	53
KDA54226.1_hypothetical_protein_EG19_00380_[Thermoanaerobaculum_aquaticum]	ptg002878l	29.299	5.59E-66	238	100
KDA54225.1_dehydrogenase_[Thermoanaerobaculum_aquaticum]	ptg002612l	35.235	5.41E-113	379	98
KDA54224.1_hypothetical_protein_EG19_00370_[Thermoanaerobaculum_aquaticum]	ptg005333l	25.088	1.11E-10	65.9	97

KDA54223.1_hypothetical_protein_EG19_00365_[Thermoanaerobaculum_aquaticum]	ptg004538l	32.258	9.51E-13	68.9	70
KDA54221.1_hypothetical_protein_EG19_00350_[Thermoanaerobaculum_aquaticum]	ptg002909l	30.833	2.19E-17	86.7	70
KDA54220.1_hypothetical_protein_EG19_00345_[Thermoanaerobaculum_aquaticum]	ptg004158l	27.551	1.64E-33	101	41
KDA54219.1_peptidase_M16_[Thermoanaerobaculum_aquaticum]	ptg002214l	26.452	4.44E-79	285	97
KDA54217.1_hypothetical_protein_EG19_00330_[Thermoanaerobaculum_aquaticum]	ptg002573l	25.707	1.76E-24	110	90
KDA54216.1_hypothetical_protein_EG19_00325_[Thermoanaerobaculum_aquaticum]	ptg004468l	42.529	1.93E-75	262	72
KDA54214.1_hypothetical_protein_EG19_00315_[Thermoanaerobaculum_aquaticum]	ptg005732l	49.091	2.11E-18	93.2	21
KDA54211.1_hypothetical_protein_EG19_00300_[Thermoanaerobaculum_aquaticum]	ptg003134l	35.484	2.89E-14	79.7	40
KDA54210.1_hypothetical_protein_EG19_00295_[Thermoanaerobaculum_aquaticum]	ptg001995l	40.393	5.71E-118	393	93
KDA54207.1_hypothetical_protein_EG19_00280_[Thermoanaerobaculum_aquaticum]	ptg002882l	34.932	1.33E-12	70.9	57
KDA54205.1_molecular_chaperone_DnaJ_[Thermoanaerobaculum_aquaticum]	ptg003680l	47.268	3.76E-71	245	98
KDA54204.1_molecular_chaperone_DnaK_[Thermoanaerobaculum_aquaticum]	ptg005922l	54.561	0	618	94
KDA54203.1_hypothetical_protein_EG19_00260_[Thermoanaerobaculum_aquaticum]	ptg005922l	37.333	3.42E-23	100	68
KDA54198.1_hypothetical_protein_EG19_01125_[Thermoanaerobaculum_aquaticum]	ptg002381l	42.308	4.83E-23	103	44
KDA54193.1_hypothetical_protein_EG19_01095_[Thermoanaerobaculum_aquaticum]	ptg004500l	34.247	5.23E-25	106	93
KDA54191.1_hypothetical_protein_EG19_01085_[Thermoanaerobaculum_aquaticum]	ptg005110l	27.486	2.49E-24	115	63
KDA54189.1_pyruvate_dehydrogenase_[Thermoanaerobaculum_aquaticum]	ptg005844l	53.089	5.2E-157	514	57
KDA54188.1_hypothetical_protein_EG19_01070_[Thermoanaerobaculum_aquaticum]	LG03	41.463	2.28E-11	64.3	51
KDA54187.1_hypothetical_protein_EG19_01065_[Thermoanaerobaculum_aquaticum]	ptg004466l	31.429	5.88E-17	85.5	74
KDA54186.1_D-alanine--D-alanine_ligase_[Thermoanaerobaculum_aquaticum]	ptg004466l	26.25	1.97E-14	78.2	93
KDA54185.1_lysine_2,3-aminomutase_[Thermoanaerobaculum_aquaticum]	ptg003299l	34.169	2.2E-51	188	82
KDA54182.1_hypothetical_protein_EG19_01040_[Thermoanaerobaculum_aquaticum]	ptg001925l	23.017	4.44E-16	87.8	63
KDA54181.1_hypothetical_protein_EG19_01035_[Thermoanaerobaculum_aquaticum]	ptg003213l	35.484	5.9E-21	95.5	59
KDA54178.1_hypothetical_protein_EG19_01015_[Thermoanaerobaculum_aquaticum]	ptg004987l	21.581	5.29E-10	67.4	54
KDA54174.1_hypothetical_protein_EG19_00995_[Thermoanaerobaculum_aquaticum]	ptg005375l	46.984	0	1005	99
KDA54173.1_hypothetical_protein_EG19_00990_[Thermoanaerobaculum_aquaticum]	ptg005435l	45.17	9.67E-95	312	99
KDA54172.1_hypothetical_protein_EG19_00985_[Thermoanaerobaculum_aquaticum]	ptg004790l	25.392	1.7E-56	215	78
KDA54171.1_beta-glucosidase_[Thermoanaerobaculum_aquaticum]	LG20	45.161	2.47E-17	89	34
KDA54169.1_sugar_ABC_transporter_permease_[Thermoanaerobaculum_aquaticum]	ptg003163l	34.595	3.08E-14	76.3	66
KDA54168.1_sugar_ABC_transporter_permease_[Thermoanaerobaculum_aquaticum]	ptg005435l	32.317	1.43E-17	86.7	55
KDA54165.1_hypothetical_protein_EG19_00950_[Thermoanaerobaculum_aquaticum]	ptg001925l	22.569	6.88E-14	80.5	82
KDA54164.1_hypothetical_protein_EG19_00945_[Thermoanaerobaculum_aquaticum]	ptg005822l	33.228	2.43E-37	146	90
KDA54161.1_hypothetical_protein_EG19_00930_[Thermoanaerobaculum_aquaticum]	ptg004779l	30.189	0.00000909	44.7	70
KDA54160.1_hypothetical_protein_EG19_00925_[Thermoanaerobaculum_aquaticum]	ptg003304l	25.907	9.41E-08	56.2	71
KDA54159.1_hypothetical_protein_EG19_00920_[Thermoanaerobaculum_aquaticum]	ptg004242l	36.769	6.92E-61	215	99

KDA54155.1_hypothetical_protein_EG19_00900_[Thermoanaerobaculum_aquaticum]	ptg004055l	38.996	1.82E-47	177	84
KDA54151.1_glycosyl_transferase_family_2_[Thermoanaerobaculum_aquaticum]	ptg003871l	35.945	1.07E-27	114	84
KDA54143.1_hypothetical_protein_EG19_00840_[Thermoanaerobaculum_aquaticum]	ptg002658l	30.796	1.46E-24	114	65
KDA54142.1_hypothetical_protein_EG19_00835_[Thermoanaerobaculum_aquaticum]	ptg002534l	34.194	4.11E-18	90.1	41
KDA54141.1_hypothetical_protein_EG19_00830_[Thermoanaerobaculum_aquaticum]	ptg002534l	27.843	9.02E-12	70.5	68
KDA54138.1_hypothetical_protein_EG19_00810_[Thermoanaerobaculum_aquaticum]	ptg002316l	30.108	1.16E-28	122	92
KDA54137.1_hypothetical_protein_EG19_00805_[Thermoanaerobaculum_aquaticum]	ptg002916l	37.143	1.77E-24	67.8	90
KDA54135.1_hypothetical_protein_EG19_00795_[Thermoanaerobaculum_aquaticum]	ptg005150l	40.052	5.21E-80	272	90
KDA54134.1_hypothetical_protein_EG19_00790_[Thermoanaerobaculum_aquaticum]	ptg005933l	41.573	2.37E-81	277	96
KDA54131.1_hypothetical_protein_EG19_00775_[Thermoanaerobaculum_aquaticum]	ptg002928l	38.63	7.05E-70	241	99
KDA54128.1_methylmalonyl-CoA_carboxyltransferase_[Thermoanaerobaculum_aquaticum]	ptg003168l	62.754	0	582	97
KDA54127.1_hypothetical_protein_EG19_00755_[Thermoanaerobaculum_aquaticum]	ptg004240l	46.593	1.07E-118	387	91
KDA54126.1_hypothetical_protein_EG19_00750_[Thermoanaerobaculum_aquaticum]	ptg002223l	40.845	2.19E-08	55.8	43
KDA54125.1_hypothetical_protein_EG19_00745_[Thermoanaerobaculum_aquaticum]	ptg001925l	24.742	0.000000043	61.6	29
KDA54124.1_hypothetical_protein_EG19_00740_[Thermoanaerobaculum_aquaticum]	ptg000619l	32.282	3.37E-40	164	52
KDA54119.1_hypothetical_protein_EG19_00710_[Thermoanaerobaculum_aquaticum]	ptg004186l	35.616	0.000000563	54.3	28
KDA54117.1_hypothetical_protein_EG19_00700_[Thermoanaerobaculum_aquaticum]	ptg006001l	24.286	2.25E-19	92.8	98
KDA54116.1_hypothetical_protein_EG19_00695_[Thermoanaerobaculum_aquaticum]	ptg002928l	37.719	4.87E-19	90.1	43
KDA54113.1_threonyl-tRNA_synthetase_[Thermoanaerobaculum_aquaticum]	ptg004758l	43.711	3.61E-169	539	97
KDA54112.1_50S_ribosomal_protein_L35_[Thermoanaerobaculum_aquaticum]	ptg001925l	50	2.09E-10	57.4	94
KDA54111.1_50S_ribosomal_protein_L20_[Thermoanaerobaculum_aquaticum]	ptg001925l	53.333	5.5E-24	99	98
KDA54110.1_hypothetical_protein_EG19_00650_[Thermoanaerobaculum_aquaticum]	ptg001925l	52.615	9.74E-109	352	94
KDA54108.1_hypothetical_protein_EG19_00635_[Thermoanaerobaculum_aquaticum]	ptg005527l	26.776	3.05E-19	93.6	89
KDA54107.1_hypothetical_protein_EG19_00625_[Thermoanaerobaculum_aquaticum]	ptg004785l	42.278	0	614	81
KDA54106.1_cysteine_desulfurase_[Thermoanaerobaculum_aquaticum]	ptg005265l	47.383	3.14E-101	332	94
KDA54104.1_hypothetical_protein_EG19_00610_[Thermoanaerobaculum_aquaticum]	ptg003213l	25.737	1.49E-18	93.2	94
KDA54103.1_enolase_[Thermoanaerobaculum_aquaticum]	ptg004466l	57.009	4.39E-141	449	99
KDA54101.1_hypothetical_protein_EG19_00595_[Thermoanaerobaculum_aquaticum]	ptg002986l	51.397	1.56E-49	150	41
KDA54100.1_hypothetical_protein_EG19_00590_[Thermoanaerobaculum_aquaticum]	ptg002976l	52.866	1.14E-51	181	82
KDA54098.1_hypothetical_protein_EG19_00580_[Thermoanaerobaculum_aquaticum]	ptg003363l	40.678	3.94E-15	74.3	84
KDA54097.1_anti-sigma_factor_antagonist_[Thermoanaerobaculum_aquaticum]	ptg004689l	35.417	2.3E-10	59.7	81
KDA54096.1_hypothetical_protein_EG19_00570_[Thermoanaerobaculum_aquaticum]	ptg005341l	31.452	9.14E-13	67	95
KDA54095.1_hypothetical_protein_EG19_00565_[Thermoanaerobaculum_aquaticum]	ptg005487l	29.508	1.79E-24	68.2	59
KDA54093.1_cell_division_protein_FtsH_[Thermoanaerobaculum_aquaticum]	ptg003039l	54.709	1.53E-154	496	79
KDA54092.1_hypothetical_protein_EG19_00530_[Thermoanaerobaculum_aquaticum]	ptg002123l	36.129	4.23E-19	89.7	62
KDA54091.1_hypothetical_protein_EG19_00525_[Thermoanaerobaculum_aquaticum]	ptg005265l	36.364	3.21E-13	67	98

KDA54090.1_mrp_[Thermoanaerobaculum_aquaticum]	ptg002214l	47.907	1.02E-64	225	62
KDA54087.1_dihydropteroate_synthase_[Thermoanaerobaculum_aquaticum]	ptg003947l	49.027	1.06E-44	165	93
KDA54086.1_hypothetical_protein_EG19_00490_[Thermoanaerobaculum_aquaticum]	ptg004534l	39.51	7.62E-33	135	63
KDA54085.1_hypothetical_protein_EG19_00480_[Thermoanaerobaculum_aquaticum]	ptg005341l	38.938	2.85E-68	237	87
KDA54084.1_hypothetical_protein_EG19_01240_[Thermoanaerobaculum_aquaticum]	ptg005375l	35.029	5.09E-172	565	90
KDA54083.1_hypothetical_protein_EG19_01235_[Thermoanaerobaculum_aquaticum]	ptg005906l	40.22	1.69E-84	287	98
KDA54082.1_hypothetical_protein_EG19_01230_[Thermoanaerobaculum_aquaticum]	ptg003708l	44.444	0.00000941	49.3	37
KDA54081.1_hypothetical_protein_EG19_01225_[Thermoanaerobaculum_aquaticum]	ptg003827l	34.615	2.92E-09	58.5	64
KDA54080.1_hypothetical_protein_EG19_01220_[Thermoanaerobaculum_aquaticum]	LG13	30.233	8.89E-11	67.8	34
KDA54079.1_histidyl-tRNA_synthetase_[Thermoanaerobaculum_aquaticum]	ptg005527l	44.706	9.08E-18	79.7	96
KDA54078.1_hypothetical_protein_EG19_01210_[Thermoanaerobaculum_aquaticum]	ptg001245l	33.468	6.69E-27	114	77
KDA54077.1_D-tyrosyl-tRNA(Tyr)_deacylase_[Thermoanaerobaculum_aquaticum]	ptg004026l	54.795	2.03E-36	135	97
KDA54076.1_hypothetical_protein_EG19_01200_[Thermoanaerobaculum_aquaticum]	ptg002304l	33.993	8.02E-26	116	58
KDA54073.1_single-stranded_DNA-binding_protein_[Thermoanaerobaculum_aquaticum]	ptg005756l	34.906	1.07E-14	72.8	78
KDA54072.1_hypothetical_protein_EG19_01180_[Thermoanaerobaculum_aquaticum]	ptg005903l	28.854	4.02E-11	69.7	49
KDA54070.1_hypothetical_protein_EG19_01170_[Thermoanaerobaculum_aquaticum]	ptg002658l	35.952	3.31E-59	213	76
KDA54068.1_hypothetical_protein_EG19_01155_[Thermoanaerobaculum_aquaticum]	ptg002534l	36.111	1.87E-21	96.7	87
KDA54066.1_hypothetical_protein_EG19_01145_[Thermoanaerobaculum_aquaticum]	ptg005526l	35.976	2.42E-20	97.1	42
KDA54064.1_hypothetical_protein_EG19_01440_[Thermoanaerobaculum_aquaticum]	ptg002534l	28.986	0.000000965	53.1	48
KDA54062.1_hypothetical_protein_EG19_01430_[Thermoanaerobaculum_aquaticum]	ptg005087l	27.823	5.95E-29	125	91
KDA54059.1_dihydroorotase_[Thermoanaerobaculum_aquaticum]	LG04	44.268	8.56E-72	228	97
KDA54058.1_aspartate_carbamoyltransferase_[Thermoanaerobaculum_aquaticum]	ptg005190l	52.601	7.31E-71	181	84
KDA54052.1_hypothetical_protein_EG19_01375_[Thermoanaerobaculum_aquaticum]	ptg004534l	28.136	1.34E-44	178	85
KDA54048.1_hypothetical_protein_EG19_01355_[Thermoanaerobaculum_aquaticum]	ptg005563l	33.333	4.86E-16	87.4	22
KDA54047.1_galactose-1-phosphate_uridylyltransferase_[Thermoanaerobaculum_aquaticum]	ptg003785l	31.549	5.18E-48	177	97
KDA54045.1_hypothetical_protein_EG19_01330_[Thermoanaerobaculum_aquaticum]	ptg003785l	38.9	8.83E-96	320	98
KDA54043.1_hypothetical_protein_EG19_01320_[Thermoanaerobaculum_aquaticum]	ptg005398l	39.815	1.7E-17	90.5	20
KDA54042.1_NAD(P)_transhydrogenase_subunit_alpha_[Thermoanaerobaculum_aquaticum]	ptg004054l	45.345	1.6E-74	254	89
KDA54041.1_hypothetical_protein_EG19_01310_[Thermoanaerobaculum_aquaticum]	ptg005929l	62.025	2.73E-21	89.7	85
KDA54040.1_NAD_synthetase_[Thermoanaerobaculum_aquaticum]	ptg005929l	51.974	2.46E-105	347	97
KDA54039.1_hypothetical_protein_EG19_01300_[Thermoanaerobaculum_aquaticum]	ptg005110l	34.694	1.06E-14	79.7	36
KDA54038.1_hypothetical_protein_EG19_01295_[Thermoanaerobaculum_aquaticum]	ptg002429l	30.688	8.23E-16	83.6	43
KDA54036.1_hypothetical_protein_EG19_01285_[Thermoanaerobaculum_aquaticum]	ptg003471l	28.926	1.72E-13	74.3	79
KDA54035.1_hypothetical_protein_EG19_01280_[Thermoanaerobaculum_aquaticum]	ptg004077l	33.411	3.41E-51	194	73
KDA54034.1_hypothetical_protein_EG19_01275_[Thermoanaerobaculum_aquaticum]	ptg005169l	33.675	4.07E-86	297	97

KDA54033.1_hypothetical_protein_EG19_01270_[Thermoanaerobaculum_aquaticum]	ptg004256l	41.399	5.25E-65	226	99
KDA54030.1_hypothetical_protein_EG19_01255_[Thermoanaerobaculum_aquaticum]	ptg002928l	32.222	8.4E-17	81.3	88
KDA54029.1_hypothetical_protein_EG19_01250_[Thermoanaerobaculum_aquaticum]	ptg003973l	46.281	5.04E-28	112	78
KDA54022.1_hypothetical_protein_EG19_01500_[Thermoanaerobaculum_aquaticum]	ptg005770l	29.012	0.00000252	53.5	44
KDA54020.1_hypothetical_protein_EG19_01490_[Thermoanaerobaculum_aquaticum]	ptg004942l	31.755	5.53E-36	141	95
KDA54019.1_hypothetical_protein_EG19_01485_[Thermoanaerobaculum_aquaticum]	ptg002986l	31.833	2.79E-37	129	46
KDA54017.1_hypothetical_protein_EG19_01475_[Thermoanaerobaculum_aquaticum]	ptg003785l	35.185	2.49E-11	64.7	63
KDA54016.1_hypothetical_protein_EG19_01470_[Thermoanaerobaculum_aquaticum]	ptg002316l	28.112	2.61E-09	64.7	34
KDA54015.1_hypothetical_protein_EG19_01465_[Thermoanaerobaculum_aquaticum]	ptg004476l	27.5	5.49E-14	75.9	80
KDA54014.1_ATP-dependent_DNA_helicase_RuvB,_partial_[Thermoanaerobaculum_aquaticum]	ptg005341l	58.657	2.6E-99	323	95
KDA54012.1_adenylyltransferase_[Thermoanaerobaculum_aquaticum]	ptg005689l	65.368	0	331	98
KDA54010.1_hypothetical_protein_EG19_02085_[Thermoanaerobaculum_aquaticum]	ptg001995l	25.253	1.62E-17	93.2	40
KDA54009.1_tRNA-splicing_ligase_[Thermoanaerobaculum_aquaticum]	ptg003039l	30.562	5.69E-30	128	91
KDA54008.1_hypothetical_protein_EG19_02075_[Thermoanaerobaculum_aquaticum]	ptg003708l	36.975	3.05E-18	88.2	42
KDA54005.1_hypothetical_protein_EG19_02055_[Thermoanaerobaculum_aquaticum]	ptg002867l	35.065	5.98E-36	140	78
KDA54003.1_hypothetical_protein_EG19_02045_[Thermoanaerobaculum_aquaticum]	ptg005656l	23.415	7.3E-17	90.1	58
KDA54001.1_3,4-dihydroxy-2-butanone_4-phosphate_synthase_[Thermoanaerobaculum_aquaticum]	ptg003075l	53.351	4.29E-122	393	94
KDA53999.1_hypothetical_protein_EG19_02025_[Thermoanaerobaculum_aquaticum]	ptg003220l	34.646	2.47E-12	66.6	84
KDA53998.1_haloacid_dehalogenase_[Thermoanaerobaculum_aquaticum]	ptg003880l	37.037	1.65E-75	271	76
KDA53997.1_cation_transporter_[Thermoanaerobaculum_aquaticum]	ptg004670l	32.464	1.39E-174	572	99
KDA53996.1_hypothetical_protein_EG19_02010_[Thermoanaerobaculum_aquaticum]	ptg001766l	29.647	3.96E-45	174	83
KDA53993.1_hypothetical_protein_EG19_01995_[Thermoanaerobaculum_aquaticum]	ptg005680l	47.573	8.03E-09	57	63
KDA53991.1_hypothetical_protein_EG19_01985_[Thermoanaerobaculum_aquaticum]	ptg005709l	41.758	1.45E-21	95.5	89
KDA53989.1_hypothetical_protein_EG19_01975_[Thermoanaerobaculum_aquaticum]	ptg004075l	36.975	1.3E-13	70.5	79
KDA53986.1_hypothetical_protein_EG19_01955_[Thermoanaerobaculum_aquaticum]	ptg001878l	33.333	8.96E-21	92.4	97
KDA53985.1_ATPase_AAA_[Thermoanaerobaculum_aquaticum]	ptg001690l	47.242	4.93E-95	317	93
KDA53983.1_hypothetical_protein_EG19_01940_[Thermoanaerobaculum_aquaticum]	ptg003304l	29.457	2.18E-12	67.4	79
KDA53982.1_hypothetical_protein_EG19_01935_[Thermoanaerobaculum_aquaticum]	ptg004501l	32.129	5.1E-43	119	93
KDA53981.1_hypothetical_protein_EG19_01930_[Thermoanaerobaculum_aquaticum]	ptg003548l	39.703	5.68E-108	355	99
KDA53980.1_hypothetical_protein_EG19_01925_[Thermoanaerobaculum_aquaticum]	ptg005545l	39.466	2.2E-70	241	99
KDA53979.1_glutamate-1-semialdehyde_aminotransferase_[Thermoanaerobaculum_aquaticum]	ptg002871l	47.585	1.23E-104	344	96
KDA53978.1_delta-aminolevulinic_acid_dehydratase_[Thermoanaerobaculum_aquaticum]	ptg004997l	44.753	1.27E-70	241	96
KDA53976.1_hypothetical_protein_EG19_01905_[Thermoanaerobaculum_aquaticum]	ptg001995l	43.29	6.42E-40	152	76
KDA53975.1_hypothetical_protein_EG19_01900_[Thermoanaerobaculum_aquaticum]	ptg001290l	30.915	3.93E-26	115	73

KDA53974.1_hypothetical_protein_EG19_01895_[Thermoanaerobaculum_aquaticum]	ptg001291l	34.174	6.27E-44	166	92
KDA53973.1_hypothetical_protein_EG19_01890_[Thermoanaerobaculum_aquaticum]	ptg003431l	30.935	1.92E-12	70.5	52
KDA53972.1_hypothetical_protein_EG19_01885_[Thermoanaerobaculum_aquaticum]	ptg003980l	39.331	4.23E-43	158	99
KDA53971.1_hypothetical_protein_EG19_01880_[Thermoanaerobaculum_aquaticum]	ptg005680l	26.596	3.6E-19	91.7	91
KDA53970.1_hypothetical_protein_EG19_01875_[Thermoanaerobaculum_aquaticum]	ptg002505l	36.364	3.48E-70	245	97
KDA53965.1_hypothetical_protein_EG19_01845_[Thermoanaerobaculum_aquaticum]	ptg005113l	46.479	2.31E-13	71.6	33
KDA53964.1_hypothetical_protein_EG19_01840_[Thermoanaerobaculum_aquaticum]	ptg001907l	35.714	1.48E-31	126	97
KDA53961.1_hypothetical_protein_EG19_01825_[Thermoanaerobaculum_aquaticum]	ptg005297l	32.846	1.09E-72	260	84
KDA53958.1_hypothetical_protein_EG19_01810_[Thermoanaerobaculum_aquaticum]	ptg005647l	51.282	1.61E-27	114	50
KDA53957.1_acetyl-CoA_carboxylase_subunit_alpha_[Thermoanaerobaculum_aquaticum]	ptg002304l	52.29	6.29E-90	296	83
KDA53955.1_hypothetical_protein_EG19_01795_[Thermoanaerobaculum_aquaticum]	ptg005709l	52.101	5.16E-76	253	96
KDA53954.1_hypothetical_protein_EG19_01790_[Thermoanaerobaculum_aquaticum]	ptg003787l	32.891	5.91E-55	203	73
KDA53952.1_hypothetical_protein_EG19_01780_[Thermoanaerobaculum_aquaticum]	ptg005709l	32.812	2.31E-15	75.5	84
KDA53950.1_hypothetical_protein_EG19_01770_[Thermoanaerobaculum_aquaticum]	ptg005709l	36.94	2.06E-53	190	91
KDA53948.1_phosphocarrier_protein_HPr_[Thermoanaerobaculum_aquaticum]	ptg005709l	42.222	3.41E-15	72.4	96
KDA53947.1_hypothetical_protein_EG19_01755_[Thermoanaerobaculum_aquaticum]	ptg002723l	35.395	5.39E-101	340	98
KDA53944.1_hypothetical_protein_EG19_01740_[Thermoanaerobaculum_aquaticum]	ptg005772l	41.324	5.89E-103	340	94
KDA53943.1_hypothetical_protein_EG19_01735_[Thermoanaerobaculum_aquaticum]	ptg003247l	37.374	3.34E-17	81.3	61
KDA53942.1_chromosome_partitioning_protein_[Thermoanaerobaculum_aquaticum]	ptg003829l	40.323	1.71E-57	200	99
KDA53941.1_hypothetical_protein_EG19_01725_[Thermoanaerobaculum_aquaticum]	ptg001818l	37.245	9.25E-32	128	65
KDA53940.1_hypothetical_protein_EG19_01720_[Thermoanaerobaculum_aquaticum]	ptg001925l	25.862	0.00000949	53.5	22
KDA53939.1_ornithine--oxo-acid_aminotransferase_[Thermoanaerobaculum_aquaticum]	ptg002126l	36.883	9.44E-75	257	94
KDA53938.1_hypothetical_protein_EG19_01710_[Thermoanaerobaculum_aquaticum]	ptg003999l	37.046	0	691	99
KDA53935.1_aldehyde_dehydrogenase_[Thermoanaerobaculum_aquaticum]	ptg004942l	28.293	1.91E-43	169	80
KDA53930.1_hypothetical_protein_EG19_01670_[Thermoanaerobaculum_aquaticum]	ptg003861l	29.108	8.34E-14	73.6	93
KDA53929.1_hypothetical_protein_EG19_01665_[Thermoanaerobaculum_aquaticum]	ptg004986l	31.461	4.71E-37	147	99
KDA53928.1_macrolide_ABC_transporter_ATP-binding_protein_[Thermoanaerobaculum_aquaticum]	ptg001659l	58.571	6.37E-79	261	93
KDA53925.1_hypothetical_protein_EG19_01645_[Thermoanaerobaculum_aquaticum]	ptg005906l	27.803	6.5E-09	62	54
KDA53923.1_hypothetical_protein_EG19_01635_[Thermoanaerobaculum_aquaticum]	ptg004500l	28.947	4.26E-12	73.2	34
KDA53922.1_hypothetical_protein_EG19_01630_[Thermoanaerobaculum_aquaticum]	ptg005227l	33.588	1.01E-33	134	93
KDA53921.1_hypothetical_protein_EG19_01625_[Thermoanaerobaculum_aquaticum]	ptg005227l	32.277	1.02E-19	95.1	92
KDA53920.1_hypothetical_protein_EG19_01620_[Thermoanaerobaculum_aquaticum]	ptg005448l	37.5	2.98E-17	82	93
KDA53919.1_hypothetical_protein_EG19_01615_[Thermoanaerobaculum_aquaticum]	ptg005905l	38.672	1.19E-28	117	99
KDA53918.1_hypothetical_protein_EG19_01610_[Thermoanaerobaculum_aquaticum]	ptg004500l	56.733	1.26E-165	521	99
KDA53916.1_hypothetical_protein_EG19_01600_[Thermoanaerobaculum_aquaticum]	ptg005121l	31.955	1.18E-30	126	76

KDA53911.1_carbamoyl_phosphate_synthase_large_subunit_[Thermoanaerobaculum_aquaticum]	ptg002303l	51.185	0	1040	97
KDA53910.1_hypothetical_protein_EG19_01565_[Thermoanaerobaculum_aquaticum]	ptg001693l	40.741	5.29E-70	241	99
KDA53908.1_hypothetical_protein_EG19_01555_[Thermoanaerobaculum_aquaticum]	ptg002175l	20.654	9.63E-16	85.9	70
KDA53907.1_acyl-CoA_dehydrogenase_[Thermoanaerobaculum_aquaticum]	ptg004179l	27.5	1.75E-30	128	93
KDA53902.1_hypothetical_protein_EG19_02700_[Thermoanaerobaculum_aquaticum]	ptg003839l	42.126	2.78E-52	184	100
KDA53901.1_hypothetical_protein_EG19_02695_[Thermoanaerobaculum_aquaticum]	ptg004250l	47.143	2.75E-08	60.1	16
KDA53900.1_hypothetical_protein_EG19_02690_[Thermoanaerobaculum_aquaticum]	LG06	49.479	1.83E-41	157	60
KDA53899.1_hypothetical_protein_EG19_02685_[Thermoanaerobaculum_aquaticum]	ptg003734l	30.566	1E-30	127	74
KDA53896.1_hypothetical_protein_EG19_02670_[Thermoanaerobaculum_aquaticum]	ptg004564l	46.281	1.07E-24	101	86
KDA53895.1_6,7-dimethyl-8-ribityllumazine_synthase_[Thermoanaerobaculum_aquaticum]	ptg004564l	41.727	1.7E-28	113	90
KDA53891.1_hemolysin_III_[Thermoanaerobaculum_aquaticum]	ptg004790l	42.69	1.09E-14	76.6	68
KDA53885.1_hypothetical_protein_EG19_02615_[Thermoanaerobaculum_aquaticum]	ptg002902l	29.146	4.31E-24	75.5	55
KDA53880.1_cystathionine_beta-synthase_[Thermoanaerobaculum_aquaticum]	ptg005554l	45.307	1.89E-63	221	93
KDA53876.1_pyruvate_ferredoxin_oxidoreductase_[Thermoanaerobaculum_aquaticum]	ptg002867l	25.281	3.68E-09	62.8	79
KDA53875.1_hypothetical_protein_EG19_02560_[Thermoanaerobaculum_aquaticum]	LG02	46.512	5.49E-08	60.1	15
KDA53873.1_hypothetical_protein_EG19_02550_[Thermoanaerobaculum_aquaticum]	ptg004125l	33.077	0.000000386	53.5	60
KDA53872.1_hypothetical_protein_EG19_02545_[Thermoanaerobaculum_aquaticum]	ptg004663l	33.929	5.15E-11	67.4	35
KDA53871.1_hypothetical_protein_EG19_02540_[Thermoanaerobaculum_aquaticum]	ptg004026l	31.111	3.02E-32	130	84
KDA53869.1_hypothetical_protein_EG19_02530_[Thermoanaerobaculum_aquaticum]	ptg004579l	41.096	2.26E-46	171	69
KDA53866.1_hypothetical_protein_EG19_02515_[Thermoanaerobaculum_aquaticum]	ptg001897l	38.889	0.000000397	57	13
KDA53865.1_hypothetical_protein_EG19_02510_[Thermoanaerobaculum_aquaticum]	ptg003827l	37.809	3.39E-42	157	96
KDA53864.1_hypothetical_protein_EG19_02505_[Thermoanaerobaculum_aquaticum]	ptg001690l	44.886	1.33E-90	300	97
KDA53863.1_glucose-1-phosphate_thymidylyltransferase_[Thermoanaerobaculum_aquaticum]	ptg004111l	39.496	7.11E-45	168	66
KDA53862.1_hypothetical_protein_EG19_02495_[Thermoanaerobaculum_aquaticum]	ptg002479l	48.315	1.78E-48	172	84
KDA53859.1_malic_enzyme_[Thermoanaerobaculum_aquaticum]	ptg005092l	44.504	0	609	98
KDA53858.1_hypothetical_protein_EG19_02475_[Thermoanaerobaculum_aquaticum]	ptg004510l	34.701	9.13E-44	165	72
KDA53856.1_hypothetical_protein_EG19_02465_[Thermoanaerobaculum_aquaticum]	ptg004584l	40.816	0.00000667	35.8	92
KDA53853.1_hypothetical_protein_EG19_02450_[Thermoanaerobaculum_aquaticum]	ptg004670l	28.727	2.23E-29	121	91
KDA53851.1_hypothetical_protein_EG19_02440_[Thermoanaerobaculum_aquaticum]	ptg002505l	27.362	1.21E-24	109	81
KDA53850.1_hypothetical_protein_EG19_02435_[Thermoanaerobaculum_aquaticum]	ptg004652l	46.218	2.5E-56	199	80
KDA53849.1_hypothetical_protein_EG19_02430_[Thermoanaerobaculum_aquaticum]	ptg004457l	34.586	3.04E-20	90.9	73
KDA53848.1_hypothetical_protein_EG19_02425_[Thermoanaerobaculum_aquaticum]	ptg005938l	35.789	1.42E-12	70.9	35
KDA53844.1_hypothetical_protein_EG19_02405_[Thermoanaerobaculum_aquaticum]	ptg003785l	33.974	6.73E-15	74.7	92
KDA53843.1_hypothetical_protein_EG19_02400_[Thermoanaerobaculum_aquaticum]	ptg005905l	32.5	9.16E-23	100	95
KDA53841.1_hypothetical_protein_EG19_02390_[Thermoanaerobaculum_aquaticum]	ptg004476l	24.229	0.000000385	55.1	76

KDA53840.1_hypothetical_protein_EG19_02385_[Thermoanaerobaculum_aquaticum]	ptg005204l	35.135	1.26E-11	67	49
KDA53838.1_hypothetical_protein_EG19_02375_[Thermoanaerobaculum_aquaticum]	ptg001690l	36.792	8.9E-10	58.2	91
KDA53837.1_hypothetical_protein_EG19_02370_[Thermoanaerobaculum_aquaticum]	ptg002453l	53.968	3.64E-38	145	74
KDA53836.1_cytochrome_C_oxidase_subunit_I_[Thermoanaerobaculum_aquaticum]	ptg001690l	65.038	0	633	96
KDA53835.1_hypothetical_protein_EG19_02360_[Thermoanaerobaculum_aquaticum]	ptg002453l	41.987	8.71E-61	214	90
KDA53834.1_hypothetical_protein_EG19_02355_[Thermoanaerobaculum_aquaticum]	ptg002453l	33.032	5.26E-37	142	81
KDA53832.1_hypothetical_protein_EG19_02345_[Thermoanaerobaculum_aquaticum]	ptg001690l	41.294	1.14E-76	262	93
KDA53831.1_hypothetical_protein_EG19_02340_[Thermoanaerobaculum_aquaticum]	ptg001690l	40.553	1.77E-36	139	75
KDA53830.1_hypothetical_protein_EG19_02335_[Thermoanaerobaculum_aquaticum]	ptg001690l	38.624	3.9E-31	122	96
KDA53829.1_hydrogenase_[Thermoanaerobaculum_aquaticum]	ptg002453l	65.188	3E-172	541	92
KDA53828.1_hypothetical_protein_EG19_02325_[Thermoanaerobaculum_aquaticum]	ptg001690l	55.2	9.02E-87	310	90
KDA53827.1_cytochrome_C_[Thermoanaerobaculum_aquaticum]	ptg002453l	50.242	2.82E-61	209	89
KDA53825.1_hypothetical_protein_EG19_02310_[Thermoanaerobaculum_aquaticum]	ptg004670l	30.882	3.41E-66	179	83
KDA53824.1_hypothetical_protein_EG19_02305_[Thermoanaerobaculum_aquaticum]	ptg005107l	51.316	2.96E-16	82.8	26
KDA53823.1_hypothetical_protein_EG19_02300_[Thermoanaerobaculum_aquaticum]	ptg004774l	39.606	2.46E-84	292	74
KDA53822.1_hypothetical_protein_EG19_02295_[Thermoanaerobaculum_aquaticum]	ptg002436l	37.647	3.85E-44	113	83
KDA53818.1_hypothetical_protein_EG19_02275_[Thermoanaerobaculum_aquaticum]	ptg004051l	30.882	7.91E-09	57.4	80
KDA53817.1_integration_host_factor_subunit_beta_[Thermoanaerobaculum_aquaticum]	ptg003072l	46.667	1.53E-13	67.8	99
KDA53816.1_hypothetical_protein_EG19_02265_[Thermoanaerobaculum_aquaticum]	ptg002742l	43.596	3.22E-103	347	75
KDA53815.1_hypothetical_protein_EG19_02260_[Thermoanaerobaculum_aquaticum]	ptg002942l	43.367	1.63E-40	151	81
KDA53813.1_hypothetical_protein_EG19_02250_[Thermoanaerobaculum_aquaticum]	ptg004214l	28.435	7.39E-25	108	97
KDA53812.1_hypothetical_protein_EG19_02245_[Thermoanaerobaculum_aquaticum]	ptg003910l	47.031	8.29E-110	361	88
KDA53811.1_hypothetical_protein_EG19_02240_[Thermoanaerobaculum_aquaticum]	ptg004662l	38.933	7.22E-78	265	99
KDA53810.1_2-amino-3-ketobutyrate_CoA_ligase_[Thermoanaerobaculum_aquaticum]	ptg001224l	35.714	5.78E-68	236	96
KDA53808.1_hypothetical_protein_EG19_02225_[Thermoanaerobaculum_aquaticum]	ptg005533l	31.579	7E-14	71.2	97
KDA53807.1_glycine_dehydrogenase_[Thermoanaerobaculum_aquaticum]	ptg005318l	43.35	2.53E-87	296	81
KDA53806.1_hypothetical_protein_EG19_02215_[Thermoanaerobaculum_aquaticum]	ptg005299l	36.119	1.07E-51	191	82
KDA53805.1_glycine_cleavage_system_protein_H_[Thermoanaerobaculum_aquaticum]	ptg005318l	47.581	1.21E-31	120	95
KDA53804.1_glycine_cleavage_system_protein_T_[Thermoanaerobaculum_aquaticum]	ptg005299l	37.741	1.06E-78	266	98
KDA53803.1_hypothetical_protein_EG19_02200_[Thermoanaerobaculum_aquaticum]	ptg004510l	28.696	0.00000066	55.1	34
KDA53798.1_S-adenosylmethionine_synthetase_[Thermoanaerobaculum_aquaticum]	ptg005611l	53.865	1.38E-129	415	97
KDA53796.1_hypothetical_protein_EG19_02165_[Thermoanaerobaculum_aquaticum]	ptg002304l	35.374	3.3E-38	147	92
KDA53795.1_hypothetical_protein_EG19_02160_[Thermoanaerobaculum_aquaticum]	ptg004147l	38.391	1.26E-82	282	92
KDA53793.1_asparagine--tRNA_ligase_[Thermoanaerobaculum_aquaticum]	ptg002477l	32.886	4.46E-75	259	96
KDA53792.1_hypothetical_protein_EG19_02135_[Thermoanaerobaculum_aquaticum]	ptg003910l	30.208	1.23E-23	101	94

KDA53791.1_cold-shock_protein_[Thermoanaerobaculum_aquaticum]	ptg003498l	71.875	3.82E-23	93.6	94
KDA53790.1_hypothetical_protein_EG19_02125_[Thermoanaerobaculum_aquaticum]	ptg005709l	39.189	3.21E-09	60.1	32
KDA53789.1_hypothetical_protein_EG19_02120_[Thermoanaerobaculum_aquaticum]	ptg004143l	28.228	1.16E-14	82	50
KDA53786.1_hypothetical_protein_EG19_02925_[Thermoanaerobaculum_aquaticum]	ptg005527l	27.714	5.24E-21	102	54
KDA53780.1_hypothetical_protein_EG19_02895_[Thermoanaerobaculum_aquaticum]	ptg004510l	31.466	7.33E-19	92	64
KDA53779.1_hypothetical_protein_EG19_02890_[Thermoanaerobaculum_aquaticum]	ptg005709l	38.337	1.16E-97	326	99
KDA53778.1_hypothetical_protein_EG19_02885_[Thermoanaerobaculum_aquaticum]	ptg004136l	30.621	4.42E-87	303	92
KDA53775.1_hypothetical_protein_EG19_02865_[Thermoanaerobaculum_aquaticum]	ptg003511l	25.339	1.72E-08	61.2	41
KDA53774.1_peptidase_M28_[Thermoanaerobaculum_aquaticum]	ptg005732l	35.885	2.87E-22	105	37
KDA53773.1_hypothetical_protein_EG19_02855_[Thermoanaerobaculum_aquaticum]	ptg002270l	36.364	1.71E-37	148	74
KDA53771.1_hypothetical_protein_EG19_02845_[Thermoanaerobaculum_aquaticum]	ptg005933l	40.625	1.03E-56	201	89
KDA53770.1_molybdenum_cofactor_biosynthesis_protein_MoaC_[Thermoanaerobaculum_aquaticum]	ptg005976l	47.059	4.22E-22	95.1	84
KDA53769.1_hypothetical_protein_EG19_02835_[Thermoanaerobaculum_aquaticum]	ptg005756l	45.775	4.06E-27	115	44
KDA53767.1_hypothetical_protein_EG19_02825_[Thermoanaerobaculum_aquaticum]	ptg005433l	43.119	9.39E-11	65.5	41
KDA53766.1_hypothetical_protein_EG19_02820_[Thermoanaerobaculum_aquaticum]	ptg003163l	35.616	3.94E-47	175	77
KDA53765.1_hypothetical_protein_EG19_02815_[Thermoanaerobaculum_aquaticum]	ptg001245l	32.819	5.23E-19	89.7	98
KDA53764.1_hypothetical_protein_EG19_02810_[Thermoanaerobaculum_aquaticum]	ptg002534l	31.839	7.15E-19	93.2	51
KDA53762.1_hypothetical_protein_EG19_02800_[Thermoanaerobaculum_aquaticum]	ptg002534l	29.153	4E-20	96.3	75
KDA53761.1_hypothetical_protein_EG19_02795_[Thermoanaerobaculum_aquaticum]	ptg002534l	27.148	9.19E-11	67.4	75
KDA53760.1_hypothetical_protein_EG19_02790_[Thermoanaerobaculum_aquaticum]	ptg003365l	40.439	1.1E-52	195	67
KDA53756.1_hypothetical_protein_EG19_02765_[Thermoanaerobaculum_aquaticum]	ptg005275l	54.545	1.95E-18	80.9	100
KDA53752.1_inorganic_pyrophosphatase_[Thermoanaerobaculum_aquaticum]	ptg004711l	40.964	2.6E-28	114	88
KDA53751.1_hypothetical_protein_EG19_02735_[Thermoanaerobaculum_aquaticum]	ptg005074l	31.959	0.000000021	54.3	69
KDA53744.1_hypothetical_protein_EG19_03255_[Thermoanaerobaculum_aquaticum]	ptg003864l	30.943	1.32E-69	245	97
KDA53743.1_hypothetical_protein_EG19_03250_[Thermoanaerobaculum_aquaticum]	ptg002848l	30.693	1.15E-10	69.3	28
KDA53740.1_peptidase_S9_[Thermoanaerobaculum_aquaticum]	ptg003703l	33.846	1.15E-20	97.4	54
KDA53739.1_hypothetical_protein_EG19_03230_[Thermoanaerobaculum_aquaticum]	ptg002909l	25.882	0.00000134	51.6	79
KDA53737.1_hypothetical_protein_EG19_03210_[Thermoanaerobaculum_aquaticum]	ptg001995l	43.238	1.6E-118	386	99
KDA53736.1_hypothetical_protein_EG19_03205_[Thermoanaerobaculum_aquaticum]	ptg003671l	27.126	3.75E-23	109	54
KDA53733.1_glycosyltransferase_[Thermoanaerobaculum_aquaticum]	ptg003592l	43.226	1.23E-68	235	95
KDA53728.1_hypothetical_protein_EG19_03165_[Thermoanaerobaculum_aquaticum]	ptg002928l	45.238	5.99E-12	73.2	13
KDA53726.1_hypothetical_protein_EG19_03155_[Thermoanaerobaculum_aquaticum]	ptg002102l	35.338	1.54E-18	84.7	87
KDA53725.1_hypothetical_protein_EG19_03150_[Thermoanaerobaculum_aquaticum]	ptg005526l	40.741	5.88E-38	144	98
KDA53724.1_hypothetical_protein_EG19_03145_[Thermoanaerobaculum_aquaticum]	ptg005526l	33.939	1.23E-11	71.2	34
KDA53723.1_acyl-CoA_dehydrogenase_[Thermoanaerobaculum_aquaticum]	ptg004179l	27.049	2.23E-25	112	93

KDA53722.1_hypothetical_protein_EG19_03135_[Thermoanaerobaculum_aquaticum]	ptg004564l	39.306	1.09E-57	212	94
KDA53721.1_ABC_transporter_substrate-binding_protein_[Thermoanaerobaculum_aquaticum]	ptg003947l	32.979	2.4E-35	138	95
KDA53720.1_pyruvate_dehydrogenase_[Thermoanaerobaculum_aquaticum]	ptg002612l	30.382	1.57E-67	245	93
KDA53716.1_hypothetical_protein_EG19_03105_[Thermoanaerobaculum_aquaticum]	ptg004169l	40.883	0	692	97
KDA53714.1_hypothetical_protein_EG19_03095_[Thermoanaerobaculum_aquaticum]	ptg003603l	38.095	5.45E-08	56.6	44
KDA53708.1_molecular_chaperone_DnaK_[Thermoanaerobaculum_aquaticum]	ptg005922l	61.526	0	649	94
KDA53706.1_hypothetical_protein_EG19_03055_[Thermoanaerobaculum_aquaticum]	ptg004758l	52.083	0.00000684	47.4	37
KDA53705.1_hypothetical_protein_EG19_03050_[Thermoanaerobaculum_aquaticum]	ptg001806l	39.3	2.25E-37	144	89
KDA53704.1_hypothetical_protein_EG19_03045_[Thermoanaerobaculum_aquaticum]	ptg002612l	32.567	9.04E-37	142	91
KDA53703.1_acetate_kinase_[Thermoanaerobaculum_aquaticum]	ptg004885l	45.623	7.79E-97	321	90
KDA53701.1_hypothetical_protein_EG19_03030_[Thermoanaerobaculum_aquaticum]	ptg002634l	40	3.58E-08	60.5	14
KDA53698.1_hypothetical_protein_EG19_03010_[Thermoanaerobaculum_aquaticum]	ptg005527l	36.181	2.15E-84	292	98
KDA53696.1_hypothetical_protein_EG19_03000_[Thermoanaerobaculum_aquaticum]	ptg002534l	32.719	1.3E-27	114	86
KDA53694.1_hypothetical_protein_EG19_02990_[Thermoanaerobaculum_aquaticum]	ptg002534l	40.468	8.59E-62	224	54
KDA53681.1_hypothetical_protein_EG19_05635_[Thermoanaerobaculum_aquaticum]	ptg002304l	41.423	1.31E-46	169	92
KDA53677.1_hypothetical_protein_EG19_05615_[Thermoanaerobaculum_aquaticum]	ptg003980l	24.339	0.00000232	53.5	46
KDA53676.1_hypothetical_protein_EG19_05610_[Thermoanaerobaculum_aquaticum]	ptg002723l	42.857	2.57E-49	183	50
KDA53675.1_hypothetical_protein_EG19_05605_[Thermoanaerobaculum_aquaticum]	ptg003839l	60.324	2.72E-87	290	75
KDA53674.1_lipoprotein_[Thermoanaerobaculum_aquaticum]	ptg001995l	25.592	0.0000014	54.7	47
KDA53671.1_voltage-gated_potassium_channel_[Thermoanaerobaculum_aquaticum]	ptg004266l	28.358	5.92E-17	83.6	98
KDA53670.1_ATPase_P_[Thermoanaerobaculum_aquaticum]	ptg003880l	33.582	5.8E-48	187	75
KDA53669.1_TrmB_family_transcriptional_regulator_[Thermoanaerobaculum_aquaticum]	ptg005046l	37.037	6.1E-11	61.2	69
KDA53668.1_RNA_3'-terminal-phosphate_cyclase_[Thermoanaerobaculum_aquaticum]	LG03	46.605	5.01E-76	258	95
KDA53667.1_exodeoxyribonuclease_III_[Thermoanaerobaculum_aquaticum]	ptg003839l	30.078	2.71E-26	111	97
KDA53666.1_hypothetical_protein_EG19_05560_[Thermoanaerobaculum_aquaticum]	ptg004917l	46.429	0	596	55
KDA53664.1_hypothetical_protein_EG19_05550_[Thermoanaerobaculum_aquaticum]	ptg005656l	40.938	2.31E-61	215	90
KDA53663.1_hypothetical_protein_EG19_05545_[Thermoanaerobaculum_aquaticum]	ptg004895l	47.482	1.14E-36	136	96
KDA53662.1_UDP-N-acetylglucosamine_acyltransferase_[Thermoanaerobaculum_aquaticum]	ptg004895l	39.113	2.87E-59	206	95
KDA53660.1_hypothetical_protein_EG19_05530_[Thermoanaerobaculum_aquaticum]	ptg005301l	31.016	4.43E-19	95.1	36
KDA53659.1_protease_HtpX_[Thermoanaerobaculum_aquaticum]	ptg005195l	34.51	4.97E-24	105	85
KDA53658.1_hypothetical_protein_EG19_05515_[Thermoanaerobaculum_aquaticum]	ptg005150l	37.958	7.21E-77	265	82
KDA53657.1_hypothetical_protein_EG19_05510_[Thermoanaerobaculum_aquaticum]	ptg003548l	36.19	2.33E-21	98.6	65
KDA53656.1_hypothetical_protein_EG19_05505_[Thermoanaerobaculum_aquaticum]	ptg003163l	34.42	5.32E-25	109	86
KDA53655.1_hypothetical_protein_EG19_05500_[Thermoanaerobaculum_aquaticum]	ptg005227l	24.887	2.18E-21	102	78
KDA53654.1_hypothetical_protein_EG19_05495_[Thermoanaerobaculum_aquaticum]	ptg001909l	40.909	3.13E-09	63.5	19

KDA53649.1_thymidine_kinase_[Thermoanaerobaculum_aquaticum]	ptg004623l	26.816	1.68E-09	60.5	81
KDA53648.1_endonuclease_III_[Thermoanaerobaculum_aquaticum]	ptg005275l	44.39	7.46E-45	162	96
KDA53647.1_hypothetical_protein_EG19_05460_[Thermoanaerobaculum_aquaticum]	ptg002909l	28.302	4.67E-14	74.7	81
KDA53642.1_hydroperoxidase_[Thermoanaerobaculum_aquaticum]	ptg003304l	61.326	0	905	98
KDA53641.1_2-hydroxyhepta-2,4-diene-1,7-dioate_isomerase_[Thermoanaerobaculum_aquaticum]	ptg003204l	45.5	9.49E-50	178	74
KDA53640.1_hypothetical_protein_EG19_05415_[Thermoanaerobaculum_aquaticum]	ptg001878l	29.825	3.04E-30	125	67
KDA53639.1_hypothetical_protein_EG19_05410_[Thermoanaerobaculum_aquaticum]	ptg005092l	28.632	2.54E-10	64.7	83
KDA53638.1_thioredoxin_[Thermoanaerobaculum_aquaticum]	ptg003762l	50	1.33E-30	117	97
KDA53637.1_hypothetical_protein_EG19_05400_[Thermoanaerobaculum_aquaticum]	ptg004306l	33.533	4.99E-18	87	61
KDA53635.1_hypothetical_protein_EG19_05390_[Thermoanaerobaculum_aquaticum]	ptg003644l	29.104	7.13E-17	83.6	98
KDA53634.1_hypothetical_protein_EG19_05385_[Thermoanaerobaculum_aquaticum]	ptg004538l	48.017	5.86E-121	405	58
KDA53632.1_hypothetical_protein_EG19_05375_[Thermoanaerobaculum_aquaticum]	ptg002634l	36.111	1.7E-14	70.9	99
KDA53630.1_cysteine_desulfurase_[Thermoanaerobaculum_aquaticum]	ptg005671l	46.684	1.17E-113	369	94
KDA53629.1_hypothetical_protein_EG19_05360_[Thermoanaerobaculum_aquaticum]	ptg003472l	34.551	5.57E-43	165	71
KDA53628.1_Fe-S_cluster_assembly_protein_SufB_[Thermoanaerobaculum_aquaticum]	ptg002634l	63.771	0	612	99
KDA53627.1_hypothetical_protein_EG19_05350_[Thermoanaerobaculum_aquaticum]	ptg003472l	55.6	1.09E-86	284	96
KDA53625.1_hypothetical_protein_EG19_05340_[Thermoanaerobaculum_aquaticum]	ptg003703l	25.646	3.33E-28	124	91
KDA53624.1_methylcrotonoyl-CoA_carboxylase_[Thermoanaerobaculum_aquaticum]	ptg002612l	51.02	4.19E-175	552	97
KDA53622.1_hypothetical_protein_EG19_05325_[Thermoanaerobaculum_aquaticum]	ptg005484l	29.135	3.82E-40	165	59
KDA53621.1_hypothetical_protein_EG19_05320_[Thermoanaerobaculum_aquaticum]	ptg004987l	35.377	3.21E-35	135	99
KDA53620.1_hypothetical_protein_EG19_05315_[Thermoanaerobaculum_aquaticum]	ptg002723l	32.308	7.89E-38	145	94
KDA53619.1_hypothetical_protein_EG19_05310_[Thermoanaerobaculum_aquaticum]	ptg004479l	48.104	3.99E-135	434	98
KDA53617.1_hypothetical_protein_EG19_05300_[Thermoanaerobaculum_aquaticum]	ptg002820l	42.857	1.1E-44	162	98
KDA53616.1_Clp_protease_ClpX_[Thermoanaerobaculum_aquaticum]	ptg003199l	45.542	0	640	99
KDA53615.1_hypothetical_protein_EG19_05290_[Thermoanaerobaculum_aquaticum]	ptg002436l	25.856	2.8E-40	165	65
KDA53614.1_peptidase_S41_[Thermoanaerobaculum_aquaticum]	ptg003980l	26.718	2.61E-35	150	82
KDA53605.1_hypothetical_protein_EG19_05235_[Thermoanaerobaculum_aquaticum]	ptg003827l	27.793	3.22E-26	117	65
KDA53604.1_hypothetical_protein_EG19_05230_[Thermoanaerobaculum_aquaticum]	ptg004476l	24.333	0.000000123	57	95
KDA53598.1_GDP-D-mannose_dehydratase_[Thermoanaerobaculum_aquaticum]	ptg006001l	56.725	2.24E-122	391	97
KDA53596.1_pyridine_nucleotide-disulfide_oxidoreductase_[Thermoanaerobaculum_aquaticum]	LG02	30.588	0.00000748	54.7	15
KDA53591.1_dimethylmenaquinone_methyltransferase_[Thermoanaerobaculum_aquaticum]	ptg004863l	30.085	6.76E-26	109	91
KDA53589.1_hypothetical_protein_EG19_05145_[Thermoanaerobaculum_aquaticum]	ptg003382l	39.53	6.9E-96	320	99
KDA53587.1_superoxide_dismutase_[Thermoanaerobaculum_aquaticum]	ptg001604l	59.296	1.27E-74	247	98
KDA53585.1_hypothetical_protein_EG19_05125_[Thermoanaerobaculum_aquaticum]	ptg004359l	26.617	2.69E-14	79.3	82
KDA53584.1_LemA_family_protein_[Thermoanaerobaculum_aquaticum]	ptg005195l	42.529	5.39E-27	110	87

KDA53583.1_hypothetical_protein_EG19_05115_[Thermoanaerobaculum_aquaticum]	ptg004147l	26.009	1.44E-09	62.8	73
KDA53582.1_hypothetical_protein_EG19_05110_[Thermoanaerobaculum_aquaticum]	ptg004759l	36.842	6.56E-32	125	88
KDA53580.1_hypothetical_protein_EG19_05100_[Thermoanaerobaculum_aquaticum]	ptg002199l	37.222	4.57E-58	208	89
KDA53579.1_hypothetical_protein_EG19_05095_[Thermoanaerobaculum_aquaticum]	ptg003644l	54.494	3.44E-58	202	76
KDA53578.1_hypothetical_protein_EG19_05090_[Thermoanaerobaculum_aquaticum]	ptg005035l	32.819	5.63E-31	132	41
KDA53575.1_hypothetical_protein_EG19_05075_[Thermoanaerobaculum_aquaticum]	ptg004885l	48.361	1.16E-44	165	82
KDA53574.1_hypothetical_protein_EG19_05070_[Thermoanaerobaculum_aquaticum]	ptg002928l	37.088	2.13E-50	185	95
KDA53573.1_hypothetical_protein_EG19_05065_[Thermoanaerobaculum_aquaticum]	ptg003830l	46.734	1.42E-36	138	98
KDA53571.1_hypothetical_protein_EG19_05055_[Thermoanaerobaculum_aquaticum]	ptg003090l	33.945	6.94E-46	172	87
KDA53567.1_hypothetical_protein_EG19_05035_[Thermoanaerobaculum_aquaticum]	ptg005265l	45.07	4.78E-47	171	75
KDA53566.1_hypothetical_protein_EG19_05030_[Thermoanaerobaculum_aquaticum]	LG04	39.61	8.09E-27	115	42
KDA53561.1_hypothetical_protein_EG19_04995_[Thermoanaerobaculum_aquaticum]	ptg003831l	28.082	0.000000237	56.2	47
KDA53560.1_hypothetical_protein_EG19_04990_[Thermoanaerobaculum_aquaticum]	ptg002612l	29.834	8.64E-18	86.3	71
KDA53559.1_NAD-dependent_protein_deacylase_[Thermoanaerobaculum_aquaticum]	ptg004476l	38.095	5.17E-33	130	73
KDA53558.1_hypothetical_protein_EG19_04980_[Thermoanaerobaculum_aquaticum]	ptg002199l	34.545	1.86E-19	90.1	98
KDA53557.1_hypothetical_protein_EG19_04975_[Thermoanaerobaculum_aquaticum]	ptg002867l	37.565	5.19E-173	558	99
KDA53555.1_hypothetical_protein_EG19_04960_[Thermoanaerobaculum_aquaticum]	ptg004075l	32.867	1.81E-28	121	72
KDA53554.1_hypothetical_protein_EG19_04955_[Thermoanaerobaculum_aquaticum]	ptg005030l	37.234	2.12E-10	65.1	31
KDA53550.1_hypothetical_protein_EG19_04935_[Thermoanaerobaculum_aquaticum]	ptg004457l	33.019	2.53E-09	60.8	43
KDA53549.1_hypothetical_protein_EG19_04930_[Thermoanaerobaculum_aquaticum]	ptg001025l	44.015	7.4E-51	186	74
KDA53541.1_ABC_transporter_[Thermoanaerobaculum_aquaticum]	ptg004210l	32.169	7.54E-79	280	73
KDA53540.1_hypothetical_protein_EG19_04885_[Thermoanaerobaculum_aquaticum]	ptg005433l	29.032	9.83E-38	159	42
KDA53534.1_cold-shock_protein_[Thermoanaerobaculum_aquaticum]	ptg003498l	70.492	1.3E-23	95.1	92
KDA53533.1_hypothetical_protein_EG19_04840_[Thermoanaerobaculum_aquaticum]	ptg004143l	34.615	7.62E-21	92.8	82
KDA53532.1_hypothetical_protein_EG19_04835_[Thermoanaerobaculum_aquaticum]	ptg003419l	39.426	1.63E-92	314	68
KDA53531.1_hypothetical_protein_EG19_04830_[Thermoanaerobaculum_aquaticum]	ptg004143l	50.685	1.07E-44	160	86
KDA53530.1_hypothetical_protein_EG19_04825_[Thermoanaerobaculum_aquaticum]	ptg003673l	36.957	0.00000071	53.1	38
KDA53529.1_hypothetical_protein_EG19_04820_[Thermoanaerobaculum_aquaticum]	ptg003734l	40.067	1.94E-56	200	96
KDA53527.1_hypothetical_protein_EG19_04810_[Thermoanaerobaculum_aquaticum]	ptg002214l	43.015	2.42E-113	375	94
KDA53526.1_hypothetical_protein_EG19_04805_[Thermoanaerobaculum_aquaticum]	ptg003536l	34.286	2.19E-16	79.3	81
KDA53524.1_hypothetical_protein_EG19_04795_[Thermoanaerobaculum_aquaticum]	ptg004523l	43.411	5.94E-27	110	64
KDA53523.1_hypothetical_protein_EG19_04790_[Thermoanaerobaculum_aquaticum]	ptg004266l	31	0.00000304	49.3	60
KDA53520.1_hypothetical_protein_EG19_04745_[Thermoanaerobaculum_aquaticum]	ptg004986l	26.728	2.27E-17	88.2	49
KDA53519.1_hypothetical_protein_EG19_04740_[Thermoanaerobaculum_aquaticum]	ptg004986l	31.602	9.47E-18	89.4	51
KDA53518.1_macrolide_ABC_transporter_ATP-binding_protein_[Thermoanaerobaculum_aquaticum]	ptg005920l	46.512	2.46E-57	199	96

KDA53515.1_hypothetical_protein_EG19_04720_[Thermoanaerobaculum_aquaticum]	ptg001607l	37.959	4.96E-33	138	81
KDA53511.1_preprotein_translocase_subunit_SecA_[Thermoanaerobaculum_aquaticum]	ptg005005l	52.488	0	815	90
KDA53510.1_hypothetical_protein_EG19_04695_[Thermoanaerobaculum_aquaticum]	ptg003336l	35.18	1.58E-48	191	41
KDA53508.1_hypothetical_protein_EG19_04685_[Thermoanaerobaculum_aquaticum]	ptg000372l	27.605	6.65E-42	166	91
KDA53507.1_hypothetical_protein_EG19_04680_[Thermoanaerobaculum_aquaticum]	ptg002223l	34.934	2.81E-22	99	87
KDA53503.1_acyl-CoA_dehydrogenase_[Thermoanaerobaculum_aquaticum]	ptg004179l	32.378	2.33E-33	135	89
KDA53500.1_ADP-ribose_pyrophosphatase_[Thermoanaerobaculum_aquaticum]	ptg002634l	46.667	8.85E-16	76.3	63
KDA53498.1_hypothetical_protein_EG19_04630_[Thermoanaerobaculum_aquaticum]	ptg005150l	40.95	1.07E-101	337	96
KDA53497.1_hypothetical_protein_EG19_04625_[Thermoanaerobaculum_aquaticum]	ptg005087l	44.262	1.34E-150	487	99
KDA53496.1_hypothetical_protein_EG19_04620_[Thermoanaerobaculum_aquaticum]	ptg003220l	37.712	1.36E-32	134	53
KDA53495.1_hypothetical_protein_EG19_04615_[Thermoanaerobaculum_aquaticum]	ptg004026l	26.216	8.11E-22	100	98
KDA53489.1_hypothetical_protein_EG19_04580_[Thermoanaerobaculum_aquaticum]	ptg002381l	61.224	6.46E-10	63.5	18
KDA53488.1_hypothetical_protein_EG19_04575_[Thermoanaerobaculum_aquaticum]	ptg005274l	28.402	7.12E-10	63.5	57
KDA53486.1_hypothetical_protein_EG19_04565_[Thermoanaerobaculum_aquaticum]	ptg004151l	44.218	5.96E-35	131	96
KDA53484.1_peptidase_[Thermoanaerobaculum_aquaticum]	ptg005527l	46.164	0	682	95
KDA53482.1_NAD-dependent_dehydratase_[Thermoanaerobaculum_aquaticum]	ptg005433l	53.094	9.5E-96	313	97
KDA53481.1_hypothetical_protein_EG19_04540_[Thermoanaerobaculum_aquaticum]	ptg005087l	36.995	1.9E-107	360	99
KDA53473.1_hypothetical_protein_EG19_04500_[Thermoanaerobaculum_aquaticum]	ptg004403l	31.915	1.17E-54	206	80
KDA53472.1_hypothetical_protein_EG19_04495_[Thermoanaerobaculum_aquaticum]	ptg001353l	46.966	6.45E-74	255	86
KDA53470.1_thiol_peroxidase_[Thermoanaerobaculum_aquaticum]	ptg004538l	47.321	3E-28	112	72
KDA53464.1_hypothetical_protein_EG19_04455_[Thermoanaerobaculum_aquaticum]	ptg002214l	23.009	1.37E-15	84	90
KDA53463.1_hypothetical_protein_EG19_04450_[Thermoanaerobaculum_aquaticum]	ptg005195l	32.895	2.55E-18	93.6	48
KDA53462.1_hypothetical_protein_EG19_04445_[Thermoanaerobaculum_aquaticum]	ptg003548l	23.704	0.00000512	53.5	45
KDA53461.1_Pleiotropic_regulatory_protein_[Thermoanaerobaculum_aquaticum]	ptg004525l	50.543	2.06E-103	338	99
KDA53460.1_hypothetical_protein_EG19_04435_[Thermoanaerobaculum_aquaticum]	ptg005092l	41.228	8.56E-32	126	94
KDA53449.1_ABC_transporter_[Thermoanaerobaculum_aquaticum]	ptg003137l	42.972	5.19E-58	202	98
KDA53448.1_hypothetical_protein_EG19_05860_[Thermoanaerobaculum_aquaticum]	ptg003336l	44.872	4.6E-48	174	90
KDA53447.1_hypothetical_protein_EG19_05855_[Thermoanaerobaculum_aquaticum]	ptg005535l	39.374	1.05E-80	276	97
KDA53446.1_hypothetical_protein_EG19_05850_[Thermoanaerobaculum_aquaticum]	ptg004651l	34.872	2.08E-58	208	99
KDA53445.1_hypothetical_protein_EG19_05845_[Thermoanaerobaculum_aquaticum]	ptg001238l	48.485	0.000000264	53.9	31
KDA53444.1_hypothetical_protein_EG19_05840_[Thermoanaerobaculum_aquaticum]	ptg003764l	42.48	3.38E-85	285	96
KDA53442.1_hypothetical_protein_EG19_05830_[Thermoanaerobaculum_aquaticum]	ptg004058l	33.613	0.000000013	62	22
KDA53441.1_hypothetical_protein_EG19_05825_[Thermoanaerobaculum_aquaticum]	ptg004085l	31.452	0.000000144	56.6	38
KDA53440.1_1-pyrroline-5-carboxylate_dehydrogenase_[Thermoanaerobaculum_aquaticum]	ptg004321l	28.14	1.26E-32	137	77
KDA53437.1_hypothetical_protein_EG19_05805_[Thermoanaerobaculum_aquaticum]	ptg002465l	30.994	1.21E-12	69.3	87

KDA53436.1_nitrogen_fixation_protein_NifU_[Thermoanaerobaculum_aquaticum]	ptg003708l	39.344	2.78E-11	60.5	85
KDA53432.1_hypothetical_protein_EG19_05780_[Thermoanaerobaculum_aquaticum]	LG12	43.137	5.79E-14	76.6	63
KDA53429.1_glycosyl_transferase_[Thermoanaerobaculum_aquaticum]	ptg004680l	54.202	1.57E-62	214	98
KDA53427.1_hypothetical_protein_EG19_05755_[Thermoanaerobaculum_aquaticum]	ptg004810l	26.271	5.11E-09	57	76
KDA53426.1_hypothetical_protein_EG19_05750_[Thermoanaerobaculum_aquaticum]	ptg002767l	25.676	1.54E-08	62.8	35
KDA53424.1_hypothetical_protein_EG19_05740_[Thermoanaerobaculum_aquaticum]	ptg004814l	48.214	1.87E-54	191	90
KDA53423.1_hypothetical_protein_EG19_05735_[Thermoanaerobaculum_aquaticum]	ptg003420l	46.847	3.47E-28	111	82
KDA53420.1_pyrophosphatase_[Thermoanaerobaculum_aquaticum]	ptg004711l	39.735	4.45E-145	474	98
KDA53419.1_hypothetical_protein_EG19_05715_[Thermoanaerobaculum_aquaticum]	ptg001690l	29.032	5.74E-11	62.8	83
KDA53416.1_hypothetical_protein_EG19_05700_[Thermoanaerobaculum_aquaticum]	ptg003299l	30.457	8.06E-33	141	50
KDA53413.1_hypothetical_protein_EG19_05685_[Thermoanaerobaculum_aquaticum]	ptg004266l	50	1.2E-29	116	76
KDA53412.1_hypothetical_protein_EG19_05680_[Thermoanaerobaculum_aquaticum]	ptg004959l	28.099	5.6E-10	59.7	84
KDA53409.1_hypothetical_protein_EG19_05665_[Thermoanaerobaculum_aquaticum]	ptg003980l	29.898	1.25E-118	407	97
KDA53406.1_hypothetical_protein_EG19_06555_[Thermoanaerobaculum_aquaticum]	ptg001925l	24.573	0.00000224	55.8	30
KDA53405.1_3-oxoacyl-ACP_synthase_[Thermoanaerobaculum_aquaticum]	ptg005905l	48.571	6.58E-55	193	81
KDA53404.1_3-oxoacyl-ACP_synthase_[Thermoanaerobaculum_aquaticum]	ptg003871l	29.129	1.69E-24	108	96
KDA53403.1_hypothetical_protein_EG19_06540_[Thermoanaerobaculum_aquaticum]	ptg005922l	26.705	0.00000476	52	55
KDA53402.1_hypothetical_protein_EG19_06535_[Thermoanaerobaculum_aquaticum]	ptg005087l	32.576	1.03E-58	214	97
KDA53396.1_RNA_helicase_[Thermoanaerobaculum_aquaticum]	ptg003045l	58.11	0	1227	100
KDA53394.1_hypothetical_protein_EG19_06490_[Thermoanaerobaculum_aquaticum]	ptg003045l	42.957	0	758	98
KDA53393.1_hypothetical_protein_EG19_06485_[Thermoanaerobaculum_aquaticum]	ptg003045l	45.447	0	976	99
KDA53389.1_hypothetical_protein_EG19_06460_[Thermoanaerobaculum_aquaticum]	ptg005865l	35.87	1.82E-09	59.7	49
KDA53388.1_radical_SAM_protein_[Thermoanaerobaculum_aquaticum]	ptg001571l	52.342	2.28E-108	352	94
KDA53385.1_glutamyl-tRNA_synthetase_[Thermoanaerobaculum_aquaticum]	ptg004724l	35.361	1.88E-89	302	99
KDA53384.1_glutamate--tRNA_ligase_[Thermoanaerobaculum_aquaticum]	ptg005010l	57.812	0	610	91
KDA53383.1_hypothetical_protein_EG19_06430_[Thermoanaerobaculum_aquaticum]	ptg002634l	29.478	1.49E-13	74.7	89
KDA53380.1_hypothetical_protein_EG19_06415_[Thermoanaerobaculum_aquaticum]	ptg004895l	42.703	3.97E-88	294	95
KDA53379.1_cysteine_synthase_[Thermoanaerobaculum_aquaticum]	ptg005554l	42.857	1.39E-58	206	96
KDA53376.1_elongation_factor_P_[Thermoanaerobaculum_aquaticum]	LG03	38.71	7.74E-30	118	100
KDA53375.1_enoyl-ACP_reductase_[Thermoanaerobaculum_aquaticum]	ptg004732l	30.196	4.84E-30	121	98
KDA53372.1_arginase_[Thermoanaerobaculum_aquaticum]	LG29	54.545	2.95E-12	70.9	74
KDA53371.1_hypothetical_protein_EG19_06370_[Thermoanaerobaculum_aquaticum]	ptg005110l	39.691	1.79E-28	125	33
KDA53370.1_hypothetical_protein_EG19_06365_[Thermoanaerobaculum_aquaticum]	ptg004862l	31.452	2.29E-09	58.5	77
KDA53363.1_excinuclease_ABC_subunit_A_[Thermoanaerobaculum_aquaticum]	ptg004916l	57.757	0	980	99
KDA53362.1_hypothetical_protein_EG19_06325_[Thermoanaerobaculum_aquaticum]	ptg003498l	34.783	8.47E-16	77.8	75
KDA53361.1_hypothetical_protein_EG19_06320_[Thermoanaerobaculum_aquaticum]	ptg004680l	49.655	3.2E-31	120	97

KDA53360.1_hypothetical_protein_EG19_06315_[Thermoanaerobaculum_aquaticum]	ptg003829l	34.146	3.93E-21	101	33
KDA53359.1_hypothetical_protein_EG19_06310_[Thermoanaerobaculum_aquaticum]	ptg004510l	40.511	1.33E-54	194	87
KDA53357.1_hypothetical_protein_EG19_06300_[Thermoanaerobaculum_aquaticum]	ptg004026l	25.431	2.79E-16	87.8	33
KDA53356.1_Fis_family_transcriptional_regulator_[Thermoanaerobaculum_aquaticum]	ptg004075l	38.478	1.66E-84	287	97
KDA53352.1_hypothetical_protein_EG19_06275_[Thermoanaerobaculum_aquaticum]	ptg003431l	27.7	1.24E-16	82.8	81
KDA53351.1_serine_hydroxymethyltransferase_[Thermoanaerobaculum_aquaticum]	ptg000693l	56.897	5.18E-139	442	98
KDA53350.1_ribose_5-phosphate_isomerase_[Thermoanaerobaculum_aquaticum]	ptg005354l	44.681	7.55E-33	125	97
KDA53349.1_hypothetical_protein_EG19_06260_[Thermoanaerobaculum_aquaticum]	ptg005746l	36.83	3.28E-58	210	94
KDA53348.1_hypothetical_protein_EG19_06255_[Thermoanaerobaculum_aquaticum]	ptg003967l	30.671	1.74E-27	119	69
KDA53347.1_acetyl-CoA_carboxyl_transferase_[Thermoanaerobaculum_aquaticum]	ptg000484l	53.696	6.18E-79	263	93
KDA53345.1_hypothetical_protein_EG19_06240_[Thermoanaerobaculum_aquaticum]	ptg006032l	33.813	2.46E-39	163	30
KDA53344.1_transcriptional_regulator_[Thermoanaerobaculum_aquaticum]	ptg003903l	47.5	2.08E-49	177	97
KDA53341.1_hypothetical_protein_EG19_06210_[Thermoanaerobaculum_aquaticum]	ptg005754l	37.975	0.000000282	54.7	33
KDA53337.1_cell_division_protein_FtsE_[Thermoanaerobaculum_aquaticum]	ptg005920l	38.636	9.14E-33	128	97
KDA53336.1_hypothetical_protein_EG19_06185_[Thermoanaerobaculum_aquaticum]	ptg005689l	34.459	1.76E-12	70.1	60
KDA53335.1_hypothetical_protein_EG19_06180_[Thermoanaerobaculum_aquaticum]	ptg002634l	41.389	4.67E-56	200	96
KDA53333.1_hypothetical_protein_EG19_06170_[Thermoanaerobaculum_aquaticum]	ptg004480l	29.293	1.17E-16	84.3	86
KDA53331.1_hypothetical_protein_EG19_06160_[Thermoanaerobaculum_aquaticum]	ptg004256l	31.544	6.39E-09	57.8	71
KDA53327.1_ribulose-phosphate_3-epimerase_[Thermoanaerobaculum_aquaticum]	ptg004771l	47.867	8.68E-57	197	95
KDA53324.1_hypothetical_protein_EG19_06125_[Thermoanaerobaculum_aquaticum]	ptg002436l	33.017	6.31E-44	168	88
KDA53323.1_1-deoxy-D-xylulose_5-phosphate_reductoisomerase_[Thermoanaerobaculum_aquaticum]	ptg003090l	49.708	1.59E-83	281	88
KDA53322.1_hypothetical_protein_EG19_06115_[Thermoanaerobaculum_aquaticum]	ptg004895l	45.6	9.27E-22	98.2	44
KDA53321.1_UDP_pyrophosphate_synthase_[Thermoanaerobaculum_aquaticum]	ptg005274l	50.424	1.37E-76	256	89
KDA53320.1_hypothetical_protein_EG19_06105_[Thermoanaerobaculum_aquaticum]	ptg001238l	34.8	9.47E-39	147	92
KDA53319.1_hypothetical_protein_EG19_06100_[Thermoanaerobaculum_aquaticum]	ptg002844l	26.613	1.53E-31	135	76
KDA53318.1_hypothetical_protein_EG19_06095_[Thermoanaerobaculum_aquaticum]	ptg004085l	38.009	1.05E-33	131	94
KDA53310.1_hypothetical_protein_EG19_06055_[Thermoanaerobaculum_aquaticum]	ptg002760l	36.434	8.84E-34	136	59
KDA53309.1_hypothetical_protein_EG19_06050_[Thermoanaerobaculum_aquaticum]	ptg002909l	30.35	2.64E-14	81.3	33
KDA53308.1_hypothetical_protein_EG19_06045_[Thermoanaerobaculum_aquaticum]	ptg002316l	43.59	2.46E-28	111	100
KDA53307.1_hypothetical_protein_EG19_06040_[Thermoanaerobaculum_aquaticum]	ptg002658l	26.636	8.46E-12	69.3	72
KDA53306.1_hypothetical_protein_EG19_06035_[Thermoanaerobaculum_aquaticum]	ptg002871l	31.5	5.86E-34	137	98
KDA53303.1_ribonuclease_[Thermoanaerobaculum_aquaticum]	ptg005484l	53.659	5.71E-96	323	63
KDA53302.1_hypothetical_protein_EG19_06010_[Thermoanaerobaculum_aquaticum]	ptg003112l	32.258	6E-11	67.8	42
KDA53299.1_hypothetical_protein_EG19_05995_[Thermoanaerobaculum_aquaticum]	ptg003058l	53.307	2.18E-82	281	56

KDA53297.1_hypothetical_protein_EG19_05985_[Thermoanaerobaculum_aquaticum]	ptg002867l	46.256	2.93E-54	193	77
KDA53296.1_hypothetical_protein_EG19_05980_[Thermoanaerobaculum_aquaticum]	ptg004075l	44.488	2.23E-36	139	100
KDA53295.1_Zn-dependent_hydrolase_[Thermoanaerobaculum_aquaticum]	ptg005425l	27.189	8.27E-14	73.6	88
KDA53294.1_hypothetical_protein_EG19_05970_[Thermoanaerobaculum_aquaticum]	ptg002760l	33.544	5.18E-10	66.2	32
KDA53291.1_hypothetical_protein_EG19_05955_[Thermoanaerobaculum_aquaticum]	ptg004355l	48.171	7.55E-134	431	97
KDA53288.1_hypothetical_protein_EG19_05935_[Thermoanaerobaculum_aquaticum]	ptg004457l	35.135	1.79E-29	118	95
KDA53287.1_hypothetical_protein_EG19_05930_[Thermoanaerobaculum_aquaticum]	ptg002453l	44.981	3.29E-53	190	86
KDA53286.1_hypothetical_protein_EG19_05925_[Thermoanaerobaculum_aquaticum]	ptg002909l	30.653	2.08E-10	63.5	89
KDA53284.1_hypothetical_protein_EG19_05915_[Thermoanaerobaculum_aquaticum]	ptg004476l	30.64	3.03E-24	111	53
KDA53283.1_hypothetical_protein_EG19_05910_[Thermoanaerobaculum_aquaticum]	ptg000619l	31.077	5.89E-35	148	40
KDA53282.1_hypothetical_protein_EG19_05905_[Thermoanaerobaculum_aquaticum]	ptg001925l	25.806	1.34E-10	69.7	34
KDA53281.1_hypothetical_protein_EG19_05895_[Thermoanaerobaculum_aquaticum]	ptg001925l	25.424	0.00000167	56.2	31
KDA53275.1_acyl-CoA_dehydrogenase_[Thermoanaerobaculum_aquaticum]	ptg004179l	28.693	4.15E-36	144	87
KDA53274.1_hypothetical_protein_EG19_06775_[Thermoanaerobaculum_aquaticum]	ptg005129l	24.551	6.05E-15	80.9	82
KDA53273.1_hypothetical_protein_EG19_06770_[Thermoanaerobaculum_aquaticum]	ptg004364l	34.812	1.09E-60	221	98
KDA53269.1_hypothetical_protein_EG19_06750_[Thermoanaerobaculum_aquaticum]	ptg001690l	30	2.29E-47	176	98
KDA53268.1_hypothetical_protein_EG19_06745_[Thermoanaerobaculum_aquaticum]	ptg001690l	30.702	6.52E-08	57	44
KDA53267.1_hypothetical_protein_EG19_06735_[Thermoanaerobaculum_aquaticum]	ptg003762l	35.644	2.89E-13	74.7	29
KDA53266.1_hypothetical_protein_EG19_06730_[Thermoanaerobaculum_aquaticum]	ptg004467l	35.465	2.39E-24	102	98
KDA53265.1_hypothetical_protein_EG19_06725_[Thermoanaerobaculum_aquaticum]	ptg005487l	51.402	1.4E-30	117	98
KDA53264.1_thioredoxin_reductase_[Thermoanaerobaculum_aquaticum]	ptg005689l	55.591	2.03E-95	312	99
KDA53260.1_hypothetical_protein_EG19_06670_[Thermoanaerobaculum_aquaticum]	ptg005756l	32.143	6.44E-32	130	72
KDA53259.1_hypothetical_protein_EG19_06665_[Thermoanaerobaculum_aquaticum]	ptg004584l	33.962	1.52E-30	125	93
KDA53258.1_phosphoribosylamine--glycine_ligase_[Thermoanaerobaculum_aquaticum]	ptg002175l	44.104	4.98E-96	319	98
KDA53257.1_phosphoribosylaminoimidazole_carboxylase_[Thermoanaerobaculum_aquaticum]	ptg002871l	48.039	1.81E-15	76.3	62
KDA53254.1_uridine_kinase_[Thermoanaerobaculum_aquaticum]	ptg001291l	49.038	1.06E-43	159	98
KDA53250.1_hypothetical_protein_EG19_06620_[Thermoanaerobaculum_aquaticum]	ptg005527l	43.956	6.26E-14	68.9	96
KDA53249.1_DNA-binding_protein_[Thermoanaerobaculum_aquaticum]	ptg005527l	46.809	2.82E-18	81.3	99
KDA53248.1_vitamin_B12-dependent_ribonucleotide_reductase_[Thermoanaerobaculum_aquaticum]	ptg002475l	22.111	1.08E-20	102	61
KDA53246.1_hypothetical_protein_EG19_06595_[Thermoanaerobaculum_aquaticum]	LG03	24.242	0.000000917	51.6	70
KDA53245.1_hypothetical_protein_EG19_06590_[Thermoanaerobaculum_aquaticum]	ptg005550l	38.06	3.22E-19	86.7	85
KDA53244.1_hypothetical_protein_EG19_06580_[Thermoanaerobaculum_aquaticum]	ptg002381l	36.782	3.02E-22	102	49
KDA53242.1_hypothetical_protein_EG19_06570_[Thermoanaerobaculum_aquaticum]	ptg002381l	28.205	6.17E-22	101	81
KDA53236.1_methionyl-tRNA_synthetase_[Thermoanaerobaculum_aquaticum]	ptg002214l	25.953	4.39E-61	225	99
KDA53233.1_cysteinyl-tRNA_synthetase_[Thermoanaerobaculum_aquaticum]	ptg002844l	40.385	7.41E-93	311	98

KDA53231.1_hypothetical_protein_EG19_07020_[Thermoanaerobaculum_aquaticum]	LG22	47.727	0.00000107	47.8	56
KDA53230.1_hypothetical_protein_EG19_07015_[Thermoanaerobaculum_aquaticum]	ptg001897l	45.714	2.91E-08	60.5	15
KDA53228.1_hypothetical_protein_EG19_07000_[Thermoanaerobaculum_aquaticum]	ptg002612l	46.067	1.79E-14	71.6	75
KDA53226.1_hypothetical_protein_EG19_06990_[Thermoanaerobaculum_aquaticum]	ptg004680l	33.99	3.57E-18	89	62
KDA53221.1_hypothetical_protein_EG19_06965_[Thermoanaerobaculum_aquaticum]	ptg005906l	25.862	0.00000467	53.1	55
KDA53220.1_multidrug_ABC_transporter_substrate-binding_protein_[Thermoanaerobaculum_aquaticum]	ptg004986l	33.185	1.04E-32	134	98
KDA53219.1_hypothetical_protein_EG19_06955_[Thermoanaerobaculum_aquaticum]	ptg003471l	39.516	1.48E-15	77.4	63
KDA53217.1_hypothetical_protein_EG19_06945_[Thermoanaerobaculum_aquaticum]	ptg000492l	42.775	5.66E-101	337	99
KDA53215.1_hypothetical_protein_EG19_06935_[Thermoanaerobaculum_aquaticum]	ptg002505l	37.421	1.56E-52	189	99
KDA53213.1_hypothetical_protein_EG19_06925_[Thermoanaerobaculum_aquaticum]	ptg002316l	38.798	6.3E-31	124	64
KDA53212.1_CTP_synthetase_[Thermoanaerobaculum_aquaticum]	ptg004256l	55.699	0	610	96
KDA53211.1_2-dehydro-3-deoxyphosphooctonate_aldolase_[Thermoanaerobaculum_aquaticum]	ptg003247l	47.727	1.17E-73	248	94
KDA53210.1_hypothetical_protein_EG19_06910_[Thermoanaerobaculum_aquaticum]	ptg003827l	26.829	0.00000254	49.7	99
KDA53208.1_nucleoside_transporter_NupC_[Thermoanaerobaculum_aquaticum]	ptg001925l	44.414	8.34E-75	257	88
KDA53207.1_membrane_protein_[Thermoanaerobaculum_aquaticum]	ptg003199l	44.611	3.96E-83	227	98
KDA53206.1_7-cyano-7-deazaguanine_synthase_[Thermoanaerobaculum_aquaticum]	ptg004480l	32.444	3.66E-21	95.1	96
KDA53205.1_hypothetical_protein_EG19_06880_[Thermoanaerobaculum_aquaticum]	ptg005474l	30.745	1.59E-29	123	92
KDA53204.1_hypothetical_protein_EG19_06875_[Thermoanaerobaculum_aquaticum]	ptg001460l	39.659	1.47E-165	537	98
KDA53202.1_hypothetical_protein_EG19_06865_[Thermoanaerobaculum_aquaticum]	ptg004510l	41.322	2.35E-20	95.9	37
KDA53199.1_hypothetical_protein_EG19_06850_[Thermoanaerobaculum_aquaticum]	ptg001558l	44.633	3.64E-37	139	99
KDA53195.1_hypothetical_protein_EG19_06830_[Thermoanaerobaculum_aquaticum]	ptg002304l	39.848	1.76E-75	262	80
KDA53189.1_hypothetical_protein_EG19_06800,_partial_[Thermoanaerobaculum_aquaticum]	ptg003382l	34.503	1.86E-21	95.1	82
KDA53187.1_hypothetical_protein_EG19_07375,_partial_[Thermoanaerobaculum_aquaticum]	ptg004479l	42.931	4.5E-88	295	99
KDA53181.1_hypothetical_protein_EG19_07335_[Thermoanaerobaculum_aquaticum]	ptg004708l	58.261	1.52E-53	144	64
KDA53180.1_peptidase_S9_[Thermoanaerobaculum_aquaticum]	ptg003703l	27.864	4.35E-53	206	67
KDA53177.1_hypothetical_protein_EG19_07315_[Thermoanaerobaculum_aquaticum]	ptg004986l	40.26	2.87E-12	65.9	59
KDA53176.1_hypothetical_protein_EG19_07310_[Thermoanaerobaculum_aquaticum]	ptg004470l	34.783	3.46E-17	85.5	76
KDA53174.1_hypothetical_protein_EG19_07300_[Thermoanaerobaculum_aquaticum]	ptg001224l	47.771	6.64E-41	157	44
KDA53173.1_ATPase_[Thermoanaerobaculum_aquaticum]	ptg004623l	43.825	1.75E-57	201	93
KDA53170.1_hypothetical_protein_EG19_07280_[Thermoanaerobaculum_aquaticum]	ptg003511l	29.6	0.000000421	56.2	31
KDA53165.1_hypothetical_protein_EG19_07255_[Thermoanaerobaculum_aquaticum]	ptg006032l	34.657	2.57E-39	156	55
KDA53163.1_amino_acid_permease_[Thermoanaerobaculum_aquaticum]	ptg002867l	30.303	3.56E-24	110	96
KDA53162.1_hypothetical_protein_EG19_07240_[Thermoanaerobaculum_aquaticum]	ptg004928l	37.5	3.41E-38	148	81
KDA53161.1_symporter_[Thermoanaerobaculum_aquaticum]	ptg001353l	41.436	1.43E-55	204	70
KDA53159.1_hypothetical_protein_EG19_07215_[Thermoanaerobaculum_aquaticum]	LG04	37.811	2.92E-24	108	53
KDA53158.1_hypothetical_protein_EG19_07210_[Thermoanaerobaculum_aquaticum]	ptg004479l	37.188	8.57E-74	257	91

KDA53157.1_metal-dependent_phosphohydrolase_[Thermoanaerobaculum_aquaticum]	ptg005035l	31.933	1.5E-49	181	94
KDA53156.1_transcription_termination_factor_Rho_[Thermoanaerobaculum_aquaticum]	ptg004523l	47.645	2.49E-103	337	97
KDA53155.1_hypothetical_protein_EG19_07195_[Thermoanaerobaculum_aquaticum]	ptg006032l	29.68	1.76E-12	75.1	33
KDA53153.1_acetyl-CoA_acetyltransferase_[Thermoanaerobaculum_aquaticum]	ptg004745l	48.346	5.07E-98	323	99
KDA53150.1_hypothetical_protein_EG19_07165_[Thermoanaerobaculum_aquaticum]	ptg003137l	29.437	4.18E-16	88.6	18
KDA53147.1_hypothetical_protein_EG19_07150_[Thermoanaerobaculum_aquaticum]	ptg000819l	36.667	1.19E-53	196	72
KDA53145.1_hypothetical_protein_EG19_07140_[Thermoanaerobaculum_aquaticum]	ptg004479l	29.268	1.73E-21	95.5	79
KDA53143.1_hypothetical_protein_EG19_07130_[Thermoanaerobaculum_aquaticum]	ptg001624l	32.787	2.91E-18	87.4	70
KDA53140.1_hypothetical_protein_EG19_07450,_partial_[Thermoanaerobaculum_aquaticum]	ptg003918l	30.802	9.66E-17	84.7	73
KDA53139.1_hydrogenase_[Thermoanaerobaculum_aquaticum]	ptg004143l	24.74	7.01E-15	81.6	71
KDA53138.1_hydrogenase_[Thermoanaerobaculum_aquaticum]	ptg004143l	42.857	3E-19	90.5	46
KDA53137.1_hypothetical_protein_EG19_07435_[Thermoanaerobaculum_aquaticum]	ptg005005l	37.903	2.03E-15	75.1	91
KDA53136.1_hypothetical_protein_EG19_07430_[Thermoanaerobaculum_aquaticum]	LG28	41.27	2.43E-08	59.7	19
KDA53134.1_hypothetical_protein_EG19_07420_[Thermoanaerobaculum_aquaticum]	ptg002030l	38.267	1.34E-52	187	97
KDA53133.1_hypothetical_protein_EG19_07415_[Thermoanaerobaculum_aquaticum]	ptg005338l	36.975	4.14E-18	82.4	92
KDA53132.1_hypothetical_protein_EG19_07410_[Thermoanaerobaculum_aquaticum]	ptg004740l	37.5	6.87E-08	54.7	50
KDA53131.1_NADH_dehydrogenase_[Thermoanaerobaculum_aquaticum]	ptg005451l	45.052	6.86E-95	316	87
KDA53130.1_hypothetical_protein_EG19_07400_[Thermoanaerobaculum_aquaticum]	ptg005338l	41.935	4.22E-26	98.2	33
KDA53129.1_hypothetical_protein_EG19_07395_[Thermoanaerobaculum_aquaticum]	ptg004143l	39.941	5.77E-54	194	97
KDA53125.1_ornithine_carbamoyltransferase_[Thermoanaerobaculum_aquaticum]	ptg003673l	51.493	1.52E-85	284	86
KDA53122.1_hypothetical_protein_EG19_07640_[Thermoanaerobaculum_aquaticum]	ptg001353l	49.541	6.78E-65	225	68
KDA53121.1_hypothetical_protein_EG19_07635_[Thermoanaerobaculum_aquaticum]	ptg005110l	31.174	3.13E-15	78.6	99
KDA53118.1_hypothetical_protein_EG19_07620_[Thermoanaerobaculum_aquaticum]	ptg002304l	40.796	1.75E-128	433	83
KDA53117.1_hypothetical_protein_EG19_07615_[Thermoanaerobaculum_aquaticum]	ptg004827l	33.75	1.03E-09	46.2	69
KDA53115.1_hypothetical_protein_EG19_07605_[Thermoanaerobaculum_aquaticum]	ptg006031l	43.39	1.98E-68	235	91
KDA53114.1_hypothetical_protein_EG19_07600_[Thermoanaerobaculum_aquaticum]	ptg005150l	40.98	1.83E-99	330	96
KDA53113.1_hypothetical_protein_EG19_07595_[Thermoanaerobaculum_aquaticum]	ptg001025l	27.374	1.37E-21	104	55
KDA53112.1_elongation_factor_G_[Thermoanaerobaculum_aquaticum]	ptg004438l	46.328	2.89E-178	568	99
KDA53111.1_30S_ribosomal_protein_S7_[Thermoanaerobaculum_aquaticum]	ptg003722l	47.742	3.52E-46	164	99
KDA53110.1_30S_ribosomal_protein_S12_[Thermoanaerobaculum_aquaticum]	ptg003722l	78.632	5.09E-56	191	88
KDA53109.1_50S_ribosomal_protein_L7/L12_[Thermoanaerobaculum_aquaticum]	ptg002214l	59.223	2.51E-18	83.2	80
KDA53108.1_hypothetical_protein_EG19_07715_[Thermoanaerobaculum_aquaticum]	ptg002278l	34.459	7.65E-19	86.3	85
KDA53107.1_50S_ribosomal_protein_L1_[Thermoanaerobaculum_aquaticum]	ptg002723l	53.604	6.78E-66	224	97
KDA53106.1_50S_ribosomal_protein_L11_[Thermoanaerobaculum_aquaticum]	ptg002214l	66.429	1.06E-56	193	99
KDA53105.1_hypothetical_protein_EG19_07700_[Thermoanaerobaculum_aquaticum]	ptg002214l	54.696	4.68E-50	176	99

KDA53102.1_elongation_factor_Tu,_partial_[Thermoanaerobaculum_aquaticum]	ptg002214l	68.273	5.32E-112	357	99
KDA53100.1_GDP-L-fucose_synthase_[Thermoanaerobaculum_aquaticum]	ptg006001l	58.576	1.53E-111	358	99
KDA53098.1_hypothetical_protein_EG19_07795_[Thermoanaerobaculum_aquaticum]	ptg003768l	27.459	1.89E-12	75.5	28
KDA53097.1_hypothetical_protein_EG19_07790_[Thermoanaerobaculum_aquaticum]	ptg001690l	29.565	4.33E-11	67.8	34
KDA53095.1_hypothetical_protein_EG19_07775_[Thermoanaerobaculum_aquaticum]	ptg004085l	26.432	1.01E-16	84.7	70
KDA53093.1_hypothetical_protein_EG19_07765_[Thermoanaerobaculum_aquaticum]	ptg004055l	44	6.88E-53	196	43
KDA53092.1_hypothetical_protein_EG19_07760,_partial_[Thermoanaerobaculum_aquaticum]	ptg005259l	33.333	1.06E-09	62	45
KDA53086.1_type_I_citrate_synthase_[Thermoanaerobaculum_aquaticum]	ptg005518l	29.126	4.61E-30	127	89
KDA53084.1_hypothetical_protein_EG19_08185_[Thermoanaerobaculum_aquaticum]	ptg001025l	24.538	2.23E-21	65.9	58
KDA53083.1_hypothetical_protein_EG19_08180_[Thermoanaerobaculum_aquaticum]	ptg004179l	30.631	0.00000019	52	85
KDA53082.1_hypothetical_protein_EG19_08175_[Thermoanaerobaculum_aquaticum]	ptg004623l	32.787	1.77E-14	82.4	25
KDA53081.1_hypothetical_protein_EG19_08170_[Thermoanaerobaculum_aquaticum]	ptg004885l	30.814	0.000000283	53.5	83
KDA53080.1_hypothetical_protein_EG19_08165_[Thermoanaerobaculum_aquaticum]	ptg004468l	32.016	4.96E-36	143	69
KDA53078.1_hypothetical_protein_EG19_08155_[Thermoanaerobaculum_aquaticum]	ptg003999l	49.038	1.04E-81	273	98
KDA53076.1_hypothetical_protein_EG19_08145_[Thermoanaerobaculum_aquaticum]	ptg004759l	37.594	2.06E-14	81.3	17
KDA53073.1_hypothetical_protein_EG19_08130_[Thermoanaerobaculum_aquaticum]	ptg002123l	30.4	1.13E-11	68.2	48
KDA53071.1_lipoyl_synthase_[Thermoanaerobaculum_aquaticum]	ptg003871l	51.079	2.44E-81	271	94
KDA53070.1_deoxyhypusine_synthase_[Thermoanaerobaculum_aquaticum]	ptg005865l	57.812	6.99E-110	354	98
KDA53069.1_hypothetical_protein_EG19_08110_[Thermoanaerobaculum_aquaticum]	ptg005265l	31.271	4.28E-64	236	76
KDA53068.1_4-hydroxy-3-methylbut-2-en-1-yl_diphosphate_synthase_[Thermoanaerobaculum_aquaticum]	ptg003703l	36.585	1.3E-53	196	98
KDA53067.1_deaminase_[Thermoanaerobaculum_aquaticum]	ptg005856l	42.623	8.17E-09	56.2	43
KDA53066.1_6-phosphofructokinase_[Thermoanaerobaculum_aquaticum]	ptg003223l	48.78	2.78E-96	317	97
KDA53065.1_hypothetical_protein_EG19_08090_[Thermoanaerobaculum_aquaticum]	ptg004880l	30.939	3.47E-09	59.7	84
KDA53063.1_prolyl_endopeptidase_[Thermoanaerobaculum_aquaticum]	ptg003431l	53.001	0	757	96
KDA53062.1_3-hydroxybutyryl-CoA_dehydrogenase_[Thermoanaerobaculum_aquaticum]	ptg002612l	48.214	1.49E-73	248	99
KDA53060.1_prolyl-tRNA_synthetase_[Thermoanaerobaculum_aquaticum]	ptg005656l	59.091	5.13E-51	165	57
KDA53057.1_hypothetical_protein_EG19_08050_[Thermoanaerobaculum_aquaticum]	ptg001925l	26.91	1.12E-22	102	89
KDA53054.1_hypothetical_protein_EG19_08035_[Thermoanaerobaculum_aquaticum]	ptg005301l	29.289	3.21E-27	115	82
KDA53052.1_aminotransferase_class-V_family_protein_[Thermoanaerobaculum_aquaticum]	ptg002030l	39.247	4.77E-46	109	73
KDA53043.1_hypothetical_protein_EG19_07980_[Thermoanaerobaculum_aquaticum]	ptg002313l	32.624	1.9E-11	64.3	89
KDA53042.1_hypothetical_protein_EG19_07975_[Thermoanaerobaculum_aquaticum]	ptg005110l	40.333	4.48E-58	211	78
KDA53041.1_dihydrolipoamide_dehydrogenase_[Thermoanaerobaculum_aquaticum]	ptg003780l	41.88	4.4E-95	318	99
KDA53040.1_hypothetical_protein_EG19_07965_[Thermoanaerobaculum_aquaticum]	ptg003365l	40.523	1.78E-32	124	96
KDA53039.1_hypothetical_protein_EG19_07960_[Thermoanaerobaculum_aquaticum]	ptg005076l	24.022	3.18E-24	112	93
KDA53038.1_succinate_dehydrogenase_[Thermoanaerobaculum_aquaticum]	ptg003304l	65.517	7.66E-116	369	99
KDA53037.1_succinate_dehydrogenase_[Thermoanaerobaculum_aquaticum]	ptg002316l	63.108	0	769	100

KDA53036.1_hypothetical_protein_EG19_07945_[Thermoanaerobaculum_aquaticum]	ptg003304l	39.381	1.89E-38	144	98
KDA53035.1_hypothetical_protein_EG19_07940_[Thermoanaerobaculum_aquaticum]	ptg001693l	39.375	6.83E-30	118	90
KDA53034.1_hypothetical_protein_EG19_07935_[Thermoanaerobaculum_aquaticum]	ptg003471l	31.984	5.47E-19	92.8	65
KDA53033.1_peptidase_S9_[Thermoanaerobaculum_aquaticum]	ptg004136l	32.842	2.79E-109	367	95
KDA53031.1_peptidylprolyl_isomerase_[Thermoanaerobaculum_aquaticum]	LG03	43.137	3.54E-33	126	99
KDA53021.1_hypothetical_protein_EG19_07865_[Thermoanaerobaculum_aquaticum]	ptg005754l	23.602	1.65E-08	62	50
KDA53018.1_hypothetical_protein_EG19_07850_[Thermoanaerobaculum_aquaticum]	ptg004111l	30.034	7.19E-27	115	82
KDA53016.1_hypothetical_protein_EG19_08210_[Thermoanaerobaculum_aquaticum]	ptg005905l	45.935	9.47E-61	209	99
KDA53015.1_hypothetical_protein_EG19_08440_[Thermoanaerobaculum_aquaticum]	ptg004724l	20.588	0.00000227	55.1	52
KDA53014.1_superoxide_dismutase_[Thermoanaerobaculum_aquaticum]	ptg001604l	53.439	7.3E-56	195	79
KDA53013.1_hypothetical_protein_EG19_08430_[Thermoanaerobaculum_aquaticum]	ptg003830l	40.678	8E-33	134	41
KDA53011.1_dihydropyrimidine_dehydrogenase_[Thermoanaerobaculum_aquaticum]	ptg005689l	27.451	1.42E-10	68.2	67
KDA53010.1_hypothetical_protein_EG19_08415_[Thermoanaerobaculum_aquaticum]	ptg004790l	47	1.01E-25	103	86
KDA53009.1_hypothetical_protein_EG19_08410_[Thermoanaerobaculum_aquaticum]	ptg003625l	27.072	0.000000028	60.8	32
KDA53008.1_hypothetical_protein_EG19_08405_[Thermoanaerobaculum_aquaticum]	ptg003839l	52.756	1.72E-71	243	78
KDA53004.1_hypothetical_protein_EG19_08385_[Thermoanaerobaculum_aquaticum]	ptg005754l	37.805	4.31E-09	60.5	32
KDA53003.1_hypothetical_protein_EG19_08380_[Thermoanaerobaculum_aquaticum]	ptg005341l	36.126	6.06E-19	88.2	86
KDA53002.1_hypothetical_protein_EG19_08375_[Thermoanaerobaculum_aquaticum]	ptg005341l	32.558	2.37E-09	57.8	88
KDA52999.1_queuine_tRNA-ribosyltransferase_[Thermoanaerobaculum_aquaticum]	ptg002867l	44.957	2.22E-90	300	93
KDA52998.1_hypothetical_protein_EG19_08355_[Thermoanaerobaculum_aquaticum]	ptg005746l	39.394	2.44E-10	58.9	67
KDA52997.1_hypothetical_protein_EG19_08350_[Thermoanaerobaculum_aquaticum]	ptg005746l	38.664	6.04E-79	273	92
KDA52996.1_hypothetical_protein_EG19_08345_[Thermoanaerobaculum_aquaticum]	ptg004256l	47.305	4.3E-43	164	43
KDA52995.1_hypothetical_protein_EG19_08340_[Thermoanaerobaculum_aquaticum]	ptg002030l	32.918	1.22E-114	385	95
KDA52994.1_50S_ribosomal_protein_L28_[Thermoanaerobaculum_aquaticum]	ptg003250l	38.462	7.63E-11	58.5	100
KDA52993.1_hypothetical_protein_EG19_08330_[Thermoanaerobaculum_aquaticum]	ptg004863l	30.199	4.36E-37	145	98
KDA52992.1_hypothetical_protein_EG19_08325_[Thermoanaerobaculum_aquaticum]	ptg004863l	31.579	1.75E-12	68.9	74
KDA52991.1_hypothetical_protein_EG19_08320_[Thermoanaerobaculum_aquaticum]	ptg004771l	28.689	1.33E-08	57	66
KDA52988.1_hypothetical_protein_EG19_08300_[Thermoanaerobaculum_aquaticum]	LG01	29.795	2.17E-10	66.2	80
KDA52985.1_hypothetical_protein_EG19_08285_[Thermoanaerobaculum_aquaticum]	ptg004240l	50	5.27E-12	66.6	41
KDA52984.1_acetyl-CoA_carboxylase_[Thermoanaerobaculum_aquaticum]	ptg004240l	55.53	3.55E-142	452	99
KDA52983.1_hypothetical_protein_EG19_08275_[Thermoanaerobaculum_aquaticum]	ptg004564l	31.847	9.05E-20	89	85
KDA52982.1_hypothetical_protein_EG19_08270_[Thermoanaerobaculum_aquaticum]	ptg005702l	40.244	1.18E-16	58.2	99
KDA52981.1_hypothetical_protein_EG19_08265_[Thermoanaerobaculum_aquaticum]	ptg003583l	35.047	1.22E-18	95.9	26
KDA52980.1_glyoxylate_reductase_[Thermoanaerobaculum_aquaticum]	ptg005692l	45.603	2.24E-42	160	93
KDA52979.1_hypothetical_protein_EG19_08255_[Thermoanaerobaculum_aquaticum]	ptg005005l	30.199	1.61E-38	152	82

KDA52978.1_glutamate_dehydrogenase_[Thermoanaerobaculum_aquaticum]	ptg002928l	43.396	4.58E-97	322	99
KDA52977.1_hypothetical_protein_EG19_08245_[Thermoanaerobaculum_aquaticum]	ptg002465l	36.813	1.35E-27	114	77
KDA52972.1_3-oxoacyl-ACP_synthase_[Thermoanaerobaculum_aquaticum]	ptg003871l	44.817	1.2E-82	276	97
KDA52971.1_hypothetical_protein_EG19_08205_[Thermoanaerobaculum_aquaticum]	ptg002199l	40.441	2.53E-53	189	99
KDA52969.1_hypothetical_protein_EG19_08665_[Thermoanaerobaculum_aquaticum]	ptg003361l	29.012	3.33E-23	107	64
KDA52967.1_hypothetical_protein_EG19_08655_[Thermoanaerobaculum_aquaticum]	ptg005709l	29.717	1.11E-22	99	99
KDA52966.1_hypothetical_protein_EG19_08645_[Thermoanaerobaculum_aquaticum]	ptg005435l	26.73	2.17E-16	84.3	88
KDA52965.1_imidazoleglycerol-phosphate_dehydratase_[Thermoanaerobaculum_aquaticum]	ptg002031l	50.256	4.76E-44	159	98
KDA52964.1_hypothetical_protein_EG19_08635_[Thermoanaerobaculum_aquaticum]	ptg004372l	31.795	1.21E-21	95.5	97
KDA52963.1_hypothetical_protein_EG19_08630_[Thermoanaerobaculum_aquaticum]	ptg002534l	33.054	2.37E-36	139	97
KDA52962.1_imidazole_glycerol_phosphate_synthase_[Thermoanaerobaculum_aquaticum]	ptg002534l	49.804	9.77E-52	184	97
KDA52961.1_hypothetical_protein_EG19_08620_[Thermoanaerobaculum_aquaticum]	ptg004372l	32.335	4.48E-15	76.6	82
KDA52960.1_hypothetical_protein_EG19_08615_[Thermoanaerobaculum_aquaticum]	ptg002760l	42.137	5.85E-104	345	99
KDA52959.1_hypothetical_protein_EG19_08610_[Thermoanaerobaculum_aquaticum]	ptg004525l	54.404	2.73E-61	208	98
KDA52958.1_hypothetical_protein_EG19_08605_[Thermoanaerobaculum_aquaticum]	LG07	50.244	2.47E-36	143	60
KDA52957.1_hypothetical_protein_EG19_08600_[Thermoanaerobaculum_aquaticum]	ptg004054l	36.293	1.22E-39	149	99
KDA52956.1_hypothetical_protein_EG19_08595_[Thermoanaerobaculum_aquaticum]	ptg003967l	39.801	7.88E-13	70.1	91
KDA52955.1_hypothetical_protein_EG19_08590_[Thermoanaerobaculum_aquaticum]	ptg004214l	56.555	3.19E-134	427	98
KDA52954.1_hypothetical_protein_EG19_08585_[Thermoanaerobaculum_aquaticum]	ptg004981l	36.364	1.3E-31	127	82
KDA52948.1_thioesterase_[Thermoanaerobaculum_aquaticum]	ptg003942l	51.974	1.82E-48	170	99
KDA52946.1_MFS_transporter_[Thermoanaerobaculum_aquaticum]	ptg005318l	44.875	8.02E-102	333	99
KDA52945.1_hydroxyacid_dehydrogenase_[Thermoanaerobaculum_aquaticum]	ptg003603l	43.939	2.76E-62	216	88
KDA52944.1_hypothetical_protein_EG19_08535_[Thermoanaerobaculum_aquaticum]	ptg001693l	45.673	3.05E-114	371	100
KDA52942.1_hypothetical_protein_EG19_08525_[Thermoanaerobaculum_aquaticum]	ptg003098l	33.333	1.14E-12	74.7	33
KDA52941.1_hypothetical_protein_EG19_08520_[Thermoanaerobaculum_aquaticum]	ptg000732l	26.923	4.05E-18	89.4	65
KDA52940.1_hypothetical_protein_EG19_08515_[Thermoanaerobaculum_aquaticum]	ptg004798l	28.447	4.33E-84	303	98
KDA52939.1_50S_ribosomal_protein_L13_[Thermoanaerobaculum_aquaticum]	ptg000819l	56.693	1.54E-42	152	88
KDA52938.1_30S_ribosomal_protein_S9_[Thermoanaerobaculum_aquaticum]	ptg000819l	61.6	2.7E-37	137	98
KDA52937.1_hypothetical_protein_EG19_08495_[Thermoanaerobaculum_aquaticum]	ptg005656l	41.391	1.95E-31	127	100
KDA52936.1_uridylate_kinase_[Thermoanaerobaculum_aquaticum]	ptg002436l	53.39	5.4E-67	228	96
KDA52935.1_ribosome_recycling_factor_[Thermoanaerobaculum_aquaticum]	ptg004895l	49.398	3.74E-46	165	90
KDA52934.1_hypothetical_protein_EG19_08480_[Thermoanaerobaculum_aquaticum]	ptg004256l	47.586	2.93E-36	144	78
KDA52933.1_hypothetical_protein_EG19_08475_[Thermoanaerobaculum_aquaticum]	ptg005487l	30.165	5.93E-11	65.5	98
KDA52931.1_hypothetical_protein_EG19_08825_[Thermoanaerobaculum_aquaticum]	ptg004143l	32.934	1.89E-09	59.7	90
KDA52930.1_hypothetical_protein_EG19_08820_[Thermoanaerobaculum_aquaticum]	ptg004143l	30.841	0.00000277	50.1	57

KDA52929.1_hypothetical_protein_EG19_08815_[Thermoanaerobaculum_aquaticum]	ptg005338l	47.925	2.54E-67	233	78
KDA52928.1_NADH_dehydrogenase_[Thermoanaerobaculum_aquaticum]	ptg004143l	50.518	3.02E-122	392	97
KDA52927.1_hypothetical_protein_EG19_08805_[Thermoanaerobaculum_aquaticum]	ptg005338l	32.773	7.64E-22	70.1	70
KDA52926.1_NADH_dehydrogenase_[Thermoanaerobaculum_aquaticum]	ptg004143l	64.706	7.1E-57	194	84
KDA52925.1_hypothetical_protein_EG19_08795_[Thermoanaerobaculum_aquaticum]	ptg004143l	41.593	6.99E-24	98.6	93
KDA52916.1_aspartate_aminotransferase_[Thermoanaerobaculum_aquaticum]	ptg004905l	29.923	1.78E-36	145	95
KDA52903.1_hypothetical_protein_EG19_08685_[Thermoanaerobaculum_aquaticum]	ptg002728l	42.391	1.85E-12	66.2	74
KDA52902.1_aconitate_hydratase_[Thermoanaerobaculum_aquaticum]	ptg002123l	30.945	6.93E-23	109	50
KDA52900.1_phosphate-binding_protein,_partial_[Thermoanaerobaculum_aquaticum]	ptg002567l	57.895	4.13E-99	322	92
KDA52898.1_hypothetical_protein_EG19_08880_[Thermoanaerobaculum_aquaticum]	ptg001925l	22.928	2.71E-24	114	90
KDA52897.1_hypothetical_protein_EG19_08875_[Thermoanaerobaculum_aquaticum]	ptg003112l	29.121	2.97E-12	72.4	45
KDA52896.1_hypothetical_protein_EG19_08870_[Thermoanaerobaculum_aquaticum]	ptg002246l	25.724	8.9E-54	201	93
KDA52895.1_methylmalonyl-CoA_mutase_[Thermoanaerobaculum_aquaticum]	LG30	60.345	3.84E-12	73.6	44
KDA52892.1_hypothetical_protein_EG19_08850_[Thermoanaerobaculum_aquaticum]	ptg003583l	47.059	2.37E-09	60.8	28
KDA52891.1_UDP-N-acetylglucosamine_1-carboxyvinyltransferase_[Thermoanaerobaculum_aquaticum]	ptg005611l	49.758	1.2E-70	166	98
KDA52889.1_stationary_phase_survival_protein_SurE_[Thermoanaerobaculum_aquaticum]	ptg005398l	42.276	6.41E-48	173	92
KDA52888.1_hypothetical_protein_EG19_09310_[Thermoanaerobaculum_aquaticum]	ptg004256l	49.744	1.82E-38	144	87
KDA52887.1_hypothetical_protein_EG19_09305_[Thermoanaerobaculum_aquaticum]	ptg005611l	44.937	3.48E-66	228	99
KDA52886.1_GMP_synthase_[Thermoanaerobaculum_aquaticum]	ptg002345l	55.556	5.37E-173	544	99
KDA52883.1_hypothetical_protein_EG19_09285_[Thermoanaerobaculum_aquaticum]	ptg005067l	39.3	4.31E-36	139	97
KDA52882.1_hypothetical_protein_EG19_09280_[Thermoanaerobaculum_aquaticum]	ptg002270l	26.205	7.28E-08	60.1	43
KDA52881.1_hypothetical_protein_EG19_09275_[Thermoanaerobaculum_aquaticum]	ptg004297l	43.411	3.25E-25	108	49
KDA52879.1_hypothetical_protein_EG19_09265_[Thermoanaerobaculum_aquaticum]	ptg003592l	37.383	9.36E-17	80.5	61
KDA52876.1_hypothetical_protein_EG19_09250_[Thermoanaerobaculum_aquaticum]	ptg004509l	41.905	1.32E-14	75.5	50
KDA52873.1_glutamine_synthetase_[Thermoanaerobaculum_aquaticum]	ptg004075l	35.456	4.97E-66	233	99
KDA52871.1_hypothetical_protein_EG19_09225_[Thermoanaerobaculum_aquaticum]	ptg002992l	26.667	3.08E-11	67.4	97
KDA52870.1_hypothetical_protein_EG19_09220_[Thermoanaerobaculum_aquaticum]	ptg002918l	27.953	2.53E-29	120	90
KDA52868.1_hypothetical_protein_EG19_09210_[Thermoanaerobaculum_aquaticum]	ptg004479l	26.136	3.52E-10	62	90
KDA52864.1_hypothetical_protein_EG19_09405_[Thermoanaerobaculum_aquaticum]	ptg002381l	36.17	1.61E-08	60.1	40
KDA52860.1_hypothetical_protein_EG19_09385_[Thermoanaerobaculum_aquaticum]	ptg002381l	26.23	5.59E-16	84.7	53
KDA52858.1_hypothetical_protein_EG19_09370_[Thermoanaerobaculum_aquaticum]	ptg002381l	27.935	1.88E-16	87	47
KDA52850.1_hypothetical_protein_EG19_10095_[Thermoanaerobaculum_aquaticum]	ptg002021l	30.097	4.36E-09	61.2	67
KDA52848.1_hypothetical_protein_EG19_10085_[Thermoanaerobaculum_aquaticum]	ptg004519l	31.868	1.97E-13	73.6	66
KDA52845.1_hypothetical_protein_EG19_10105,_partial_[Thermoanaerobaculum_aquaticum]	ptg005526l	33.742	1.25E-19	89.7	80
KDA52840.1_hypothetical_protein_EG19_10145_[Thermoanaerobaculum_aquaticum]	ptg004584l	47.388	7.38E-59	205	99

KDA52839.1_hypothetical_protein_EG19_10165_[Thermoanaerobaculum_aquaticum]	ptg004529l	34.911	2.56E-24	104	68
KDA52835.1_hypothetical_protein_EG19_10885_[Thermoanaerobaculum_aquaticum]	ptg005150l	36.082	0.000000687	49.3	95
WP_053334868.1_phosphate_ABC_transporter_permease_PstA_[Thermoanaerobaculum_aquaticum]	ptg002567l	63.74	1.16E-83	286	52
WP_053334803.1_L-threonylcarbamoyladenylate_synthase_[Thermoanaerobaculum_aquaticum]	ptg005354l	36.552	4.71E-16	79.7	61
WP_053334785.1_tRNA_pseudouridine(55)_synthase_TruB_[Thermoanaerobaculum_aquaticum]	ptg003078l	40.157	1.1E-45	169	81
WP_053334715.1_tRNA_(adenosine(37)-N6)-threonylcarbamoyltransferase_complex_transferase_subunit_TsaD_[Thermoanaerobaculum_aquaticum]	ptg005331l	40.439	1.17E-39	153	87
WP_053334692.1_selenocysteine-specific_translation_elongation_factor_[Thermoanaerobaculum_aquaticum]	ptg004856l	45.091	2.81E-58	216	42
WP_038050194.1_isoleucine--tRNA_ligase_[Thermoanaerobaculum_aquaticum]	ptg002304l	40.796	1.75E-128	433	83
WP_038049899.1_glutamate--tRNA_ligase_[Thermoanaerobaculum_aquaticum]	ptg004724l	35.361	1.88E-89	302	99
WP_038049836.1_ribonuclease_Y_[Thermoanaerobaculum_aquaticum]	ptg005484l	53.659	5.71E-96	323	63
WP_038049387.1_leucine--tRNA_ligase_[Thermoanaerobaculum_aquaticum]	ptg002867l	37.565	5.19E-173	558	99
WP_038049086.1_Holliday_junction_branch_migration_protein_RuvA,_partial_[Thermoanaerobaculum_aquaticum]	ptg005341l	31.765	0.000000379	38.5	94
WP_038049054.1_Rne/Rng_family_ribonuclease_[Thermoanaerobaculum_aquaticum]	ptg005709l	38.337	1.16E-97	326	99
WP_038048174.1_L-seryl-tRNA(Sec)_selenium_transferase_[Thermoanaerobaculum_aquaticum]	ptg005933l	41.573	2.37E-81	277	96
WP_038048135.1_phenylalanine--tRNA_ligase_subunit_alpha_[Thermoanaerobaculum_aquaticum]	ptg001925l	52.615	9.74E-109	352	94
WP_038048052.1_30S_ribosomal_protein_S20_[Thermoanaerobaculum_aquaticum]	ptg002505l	44.872	0.000000386	49.7	81
WP_038047942.1_phosphate_ABC_transporter_ATP-binding_protein_PstB_[Thermoanaerobaculum_aquaticum]	ptg002567l	54.656	2.71E-85	280	98
WP_038047649.1_alanine--tRNA_ligase_[Thermoanaerobaculum_aquaticum]	ptg002916l	50.204	0	459	96
WP_038047645.1_tryptophan--tRNA_ligase_[Thermoanaerobaculum_aquaticum]	ptg003327l	50.311	9.19E-103	334	99
WP_038047074.1_transcription_termination_factor_NusA_[Thermoanaerobaculum_aquaticum]	ptg001571l	34.536	6.83E-55	202	80
WP_038046814.1_30S_ribosomal_protein_S13_[Thermoanaerobaculum_aquaticum]	ptg004509l	59.483	3.09E-39	142	91
WP_038046770.1_50S_ribosomal_protein_L2_[Thermoanaerobaculum_aquaticum]	ptg005891l	62.182	3.71E-95	310	100
WP_038046512.1_tRNA_pseudouridine(38-40)_synthase_TruA_[Thermoanaerobaculum_aquaticum]	ptg003585l	35.349	4.46E-30	122	80
WP_038046431.1_50S_ribosomal_protein_L21_[Thermoanaerobaculum_aquaticum]	ptg001290l	44.66	1.13E-14	71.6	98
WP_038046292.1_tyrosine_recombinase_XerC_[Thermoanaerobaculum_aquaticum]	ptg003090l	43.919	3.19E-65	224	99
WP_038046266.1_serine--tRNA_ligase_[Thermoanaerobaculum_aquaticum]	ptg005746l	47.786	4.53E-119	385	99
WP_038046232.1_DNA_polymerase_III_subunit_beta_[Thermoanaerobaculum_aquaticum]	ptg000372l	29.301	5.74E-35	140	99
WP_038046132.1_recombinase_RecA_[Thermoanaerobaculum_aquaticum]	ptg002699l	65.325	2.23E-124	397	94
WP_053334687.1_UbiX_family_flavin_prenyltransferase_[Thermoanaerobaculum_aquaticum]	ptg001571l	35.897	2.61E-28	114	96
WP_038048527.1_carbamoyl-phosphate_synthase_large_subunit_[Thermoanaerobaculum_aquaticum]	ptg002303l	51.185	0	1040	97
WP_038048243.1_pyruvate_dehydrogenase_(acetyl-transferring),_homodimeric_type_[Thermoanaerobaculum_aquaticum]	ptg005844l	53.089	5.2E-157	514	57
WP_038047442.1_phosphoribosylformylglycinamidine_synthase_subunit_PurQ_[Thermoanaerobaculum_aquaticum]	ptg005375l	35.319	4.85E-32	126	93

WP_038049520.1_endonuclease_III_[Thermoanaerobaculum_aquaticum]	ptg005275l	44.39	7.46E-45	162	96
WP_038049871.1_dGTP_triphosphohydrolase_[Thermoanaerobaculum_aquaticum]	ptg005746l	36.83	3.28E-58	210	94
WP_038049832.1_polyprenyl_synthetase_family_protein_[Thermoanaerobaculum_aquaticum]	ptg002867l	46.256	2.93E-54	193	77
WP_327138697.1_NADH-quinone_oxidoreductase_subunit_B_family_protein_[Thermoanaerobaculum_aquaticum]	ptg004143l	64.706	6.25E-57	194	86
WP_327138687.1_SelT/SelW/SelH_family_(seleno)protein_[Thermoanaerobaculum_aquaticum]	ptg001460l	45.833	2.82E-15	71.6	100
WP_327138680.1_HAMP_domain-containing_sensor_histidine_kinase_[Thermoanaerobaculum_aquaticum]	ptg002909l	30.833	1.24E-17	85.9	87
WP_053335267.1_type_I_3-dehydroquinate_dehydratase_[Thermoanaerobaculum_aquaticum]	ptg003361l	29.012	3.33E-23	107	64
WP_053334861.1_phosphoenolpyruvate_carboxykinase_(ATP)_[Thermoanaerobaculum_aquaticum]	ptg002021l	31.646	0.000000666	56.6	25
WP_053334776.1_GreA/GreB_family_elongation_factor_[Thermoanaerobaculum_aquaticum]	ptg001715l	30.657	6.78E-14	79.7	20
WP_053335073.1_SUMF1/EgtB/PvdO_family_nonheme_iron_enzyme_[Thermoanaerobaculum_aquaticum]	ptg001607l	37.959	4.96E-33	138	81
WP_038050322.1_deoxyhypusine_synthase_family_protein_[Thermoanaerobaculum_aquaticum]	ptg005865l	57.812	6.99E-110	354	98
WP_038050298.1_DUF6036_family_nucleotidyltransferase_[Thermoanaerobaculum_aquaticum]	ptg002313l	32.624	1.9E-11	64.3	89
WP_053335216.1_2Fe-2S_iron-sulfur_cluster-binding_protein_[Thermoanaerobaculum_aquaticum]	ptg005338l	41.935	4.22E-26	98.2	33
WP_038049484.1_cation:proton_antiporter_[Thermoanaerobaculum_aquaticum]	ptg003703l	25.646	3.33E-28	124	91
WP_268746956.1_GTP-binding_protein,_partial_[Thermoanaerobaculum_aquaticum]	ptg002214l	96	2.05E-10	56.2	60
WP_038050234.1_GTP-binding_protein,_partial_[Thermoanaerobaculum_aquaticum]	ptg002723l	61.176	1.28E-28	110	100
WP_038047057.1_TGS_domain-containing_protein_[Thermoanaerobaculum_aquaticum]	ptg002505l	27.419	0.00000291	52.8	52
WP_081800137.1_peptidylprolyl_isomerase_[Thermoanaerobaculum_aquaticum]	ptg003583l	35.047	1.22E-18	95.9	26
WP_038049181.1_AAA_family_ATPase_[Thermoanaerobaculum_aquaticum]	ptg003864l	30.943	1.32E-69	245	97
WP_038046393.1_Smr/MutS_family_protein_[Thermoanaerobaculum_aquaticum]	ptg001460l	27.509	4.25E-11	70.9	34
WP_053334823.1_FHA_domain-containing_protein_[Thermoanaerobaculum_aquaticum]	ptg004256l	34.783	2.51E-10	64.7	34
WP_038047589.1_aminotransferase_class_V-fold_PLP-dependent_enzyme_[Thermoanaerobaculum_aquaticum]	ptg004917l	26.667	1.28E-09	63.9	61
WP_053334843.1_multiheme_c-type_cytochrome_[Thermoanaerobaculum_aquaticum]	ptg004277l	25.874	0.00000223	49.3	70
WP_053334791.1_peptide_chain_release_factor_N(5)-glutamine_methyltransferase_[Thermoanaerobaculum_aquaticum]	ptg004982l	31.618	2.56E-22	100	91
WP_038046184.1_pantoate--beta-alanine_ligase_[Thermoanaerobaculum_aquaticum]	ptg002465l	44.815	7.73E-67	229	95
WP_053335003.1_cytochrome_C_oxidase_subunit_IV_family_protein_[Thermoanaerobaculum_aquaticum]	ptg001690l	37	1.09E-08	54.3	98
WP_152543905.1_ABC_transporter_permease_subunit,_partial_[Thermoanaerobaculum_aquaticum]	ptg002567l	35.577	6.48E-52	124	68
WP_038049064.1_SurA_N-terminal_domain-containing_protein_[Thermoanaerobaculum_aquaticum]	ptg005527l	27.714	5.24E-21	102	54
WP_268746959.1_proton-conducting_transporter_membrane_subunit,_partial_[Thermoanaerobaculum_aquaticum]	ptg005338l	52.607	1E-51	186	70
WP_268746954.1_proton-conducting_transporter_membrane_subunit,_partial_[Thermoanaerobaculum_aquaticum]	ptg005338l	52.752	3.78E-54	192	76
WP_200867146.1_proton-conducting_transporter_membrane_subunit,_partial_[Thermoanaerobaculum_aquaticum]	ptg003918l	30.802	9.66E-17	84.7	73

WP_053334991.1_proton-conducting_transporter_membrane_subunit_[Thermoanaerobaculum_aquaticum]	ptg004143l	28.228	1.16E-14	82	50
WP_038050159.1_proton-conducting_transporter_membrane_subunit,_partial_[Thermoanaerobaculum_aquaticum]	ptg005338l	66.129	4.1E-20	85.5	83
WP_161685437.1_SDR_family_oxidoreductase_[Thermoanaerobaculum_aquaticum]	ptg003980l	39.111	2.06E-38	145	88
WP_053335007.1_SDR_family_oxidoreductase_[Thermoanaerobaculum_aquaticum]	ptg005905l	32.5	9.16E-23	100	95
WP_053334864.1_SDR_family_oxidoreductase_[Thermoanaerobaculum_aquaticum]	ptg005905l	33.884	1.68E-27	114	91
WP_038050540.1_SDR_family_oxidoreductase_[Thermoanaerobaculum_aquaticum]	ptg004297l	43.411	3.25E-25	108	49
WP_038049907.1_helicase-related_protein_[Thermoanaerobaculum_aquaticum]	ptg003045l	58.11	0	1227	100
WP_038048405.1_sugar_phosphate_nucleotidyltransferase_[Thermoanaerobaculum_aquaticum]	ptg005563l	33.333	4.86E-16	87.4	22
WP_161685620.1_DEAD/DEAH_box_helicase_family_protein,_partial_[Thermoanaerobaculum_aquaticum]	ptg001718l	34.028	1.76E-15	56.2	97
WP_038049813.1_carboxypeptidase_regulatory-like_domain-containing_protein_[Thermoanaerobaculum_aquaticum]	ptg001925l	25.806	1.34E-10	69.7	34
WP_038049478.1_MBL_fold_metallo-hydrolase_[Thermoanaerobaculum_aquaticum]	ptg004987l	35.377	3.21E-35	135	99
WP_161685448.1_YHS_domain-containing_protein_[Thermoanaerobaculum_aquaticum]	ptg003220l	34.4	9.68E-12	64.7	86
WP_152543830.1_multiheme_c-type_cytochrome_[Thermoanaerobaculum_aquaticum]	ptg004277l	28.025	2.11E-08	55.5	76
WP_038049041.1_gephyrin-like_molybdotransferase_Glp_[Thermoanaerobaculum_aquaticum]	ptg002270l	36.364	1.71E-37	148	74
WP_038048136.1_50S_ribosomal_protein_L20_[Thermoanaerobaculum_aquaticum]	ptg001925l	53.333	5.5E-24	99	98
WP_053334882.1_nucleotide_exchange_factor_GrpE_[Thermoanaerobaculum_aquaticum]	ptg005922l	37.333	3.42E-23	100	68
WP_038047361.1_FmdB_family_zinc_ribbon_protein_[Thermoanaerobaculum_aquaticum]	ptg004942l	37.143	3.94E-08	52.4	72
WP_038050293.1_GAF_domain-containing_protein_[Thermoanaerobaculum_aquaticum]	ptg003365l	40.523	1.78E-32	124	96
WP_053335077.1_site-specific_tyrosine_recombinase_XerD_[Thermoanaerobaculum_aquaticum]	ptg003734l	40.067	1.94E-56	200	96
WP_038050388.1_serine_O-acetyltransferase_EpsC_[Thermoanaerobaculum_aquaticum]	ptg003839l	52.756	1.72E-71	243	78
WP_038049548.1_exodeoxyribonuclease_III_[Thermoanaerobaculum_aquaticum]	ptg003839l	30.078	2.71E-26	111	97
WP_038049021.1_undecaprenyl-phosphate_glucose_phosphotransferase_[Thermoanaerobaculum_aquaticum]	ptg003365l	40.439	1.1E-52	195	67
WP_038048787.1_YicC/YloC_family_endoribonuclease_[Thermoanaerobaculum_aquaticum]	ptg005107l	51.316	2.96E-16	82.8	26
WP_038047566.1_nucleoside-diphosphate_kinase_[Thermoanaerobaculum_aquaticum]	ptg002916l	51.799	6.56E-43	154	99
WP_038049861.1_cell_division_ATP-binding_protein_FtsE_[Thermoanaerobaculum_aquaticum]	ptg005920l	38.636	9.14E-33	128	97
WP_038049383.1_tRNA_uracil_4-sulfurtransferase_ThiI_[Thermoanaerobaculum_aquaticum]	ptg004075l	32.867	1.81E-28	121	72
WP_235208693.1_PhoH_family_protein_[Thermoanaerobaculum_aquaticum]	ptg005772l	46.689	7.74E-78	262	96
WP_200867117.1_ATP-dependent_helicase_[Thermoanaerobaculum_aquaticum]	ptg002907l	32.847	2.51E-81	286	94
WP_081800144.1_RNA_methyltransferase_[Thermoanaerobaculum_aquaticum]	ptg005487l	30.328	3.32E-11	66.6	96
WP_053334706.1_OmpA_family_protein_[Thermoanaerobaculum_aquaticum]	ptg003829l	35.948	5.9E-13	70.5	71
WP_038050533.1_OmpA_family_protein_[Thermoanaerobaculum_aquaticum]	ptg004509l	41.905	1.32E-14	75.5	50
WP_038049882.1_NUDIX_hydrolase_[Thermoanaerobaculum_aquaticum]	ptg003498l	34.783	8.47E-16	77.8	75
WP_038049867.1_MotA/TolQ/ExbB_proton_channel_family_protein_[Thermoanaerobaculum_aquaticum]	ptg002166l	34.737	1.98E-28	115	84

WP_038049483.1_acyl-CoA_carboxylase_subunit_beta_[Thermoanaerobaculum_aquaticum]	ptg002612l	51.02	4.19E-175	552	97
WP_038048849.1_TetR/AcrR_family_transcriptional_regulator_[Thermoanaerobaculum_aquaticum]	ptg004125l	33.077	0.000000386	53.5	60
WP_038048164.1_acyl-CoA_carboxylase_subunit_beta_[Thermoanaerobaculum_aquaticum]	ptg003168l	62.697	0	583	97
WP_038046215.1_3',5'-cyclic-nucleotide_phosphodiesterase_[Thermoanaerobaculum_aquaticum]	ptg003764l	25.328	0.00000357	51.6	88
WP_038046113.1_ferredoxin--NADP_reductase_[Thermoanaerobaculum_aquaticum]	ptg004979l	32.83	7.35E-35	136	95
WP_161685617.1_RHS_repeat-associated_core_domain-containing_protein_[Thermoanaerobaculum_aquaticum]	ptg002381l	45.283	0.000000197	56.2	17
WP_152544026.1_polyprenyl_synthetase_family_protein_[Thermoanaerobaculum_aquaticum]	ptg005486l	38.507	8.58E-53	190	98
WP_053335251.1_bifunctional_(p)ppGpp_synthetase/guanosine-3',5'-bis(diphosphate)_3'-pyrophosphohydrolase_[Thermoanaerobaculum_aquaticum]	ptg002030l	32.918	1.17E-114	385	95
WP_053335212.1_S9_family_peptidase_[Thermoanaerobaculum_aquaticum]	ptg003703l	27.864	4.35E-53	206	67
WP_053335201.1_M23_family_metallopeptidase_[Thermoanaerobaculum_aquaticum]	ptg000819l	36.667	1.19E-53	196	72
WP_053335190.1_M23_family_metallopeptidase_[Thermoanaerobaculum_aquaticum]	ptg004510l	41.322	2.35E-20	95.9	37
WP_053335127.1_NAD-dependent_deacylase_[Thermoanaerobaculum_aquaticum]	ptg004476l	38.095	5.17E-33	130	73
WP_053335089.1_FHA_domain-containing_protein_[Thermoanaerobaculum_aquaticum]	ptg005030l	37.234	2.12E-10	65.1	31
WP_053334936.1_NAD(P)/FAD-dependent_oxidoreductase_[Thermoanaerobaculum_aquaticum]	ptg002658l	35.952	3.31E-59	213	76
WP_053334888.1_pentapeptide_repeat-containing_protein_[Thermoanaerobaculum_aquaticum]	ptg003134l	35.484	2.89E-14	79.7	40
WP_038050558.1_RHS_repeat-associated_core_domain-containing_protein,_partial_[Thermoanaerobaculum_aquaticum]	ptg002381l	44.643	0.000000263	55.5	20
WP_038050284.1_S9_family_peptidase_[Thermoanaerobaculum_aquaticum]	ptg004136l	32.842	2.79E-109	367	95
WP_038049898.1_NAD(P)-dependent_oxidoreductase_[Thermoanaerobaculum_aquaticum]	ptg002634l	29.478	1.49E-13	74.7	89
WP_038049467.1_ATP-dependent_Clp_protease_ATP-binding_subunit_[Thermoanaerobaculum_aquaticum]	ptg003199l	45.542	0	640	99
WP_038049357.1_cold-shock_protein_[Thermoanaerobaculum_aquaticum]	ptg003498l	70.492	1.3E-23	95.1	92
WP_038049354.1_NAD-dependent_malic_enzyme_[Thermoanaerobaculum_aquaticum]	ptg002214l	43.015	2.42E-113	375	94
WP_038049347.1_thioredoxin_family_protein_[Thermoanaerobaculum_aquaticum]	ptg004523l	43.411	5.94E-27	110	64
WP_038049051.1_S9_family_peptidase_[Thermoanaerobaculum_aquaticum]	ptg004136l	30.621	4.42E-87	303	92
WP_038048765.1_6-carboxytetrahydropterin_synthase_[Thermoanaerobaculum_aquaticum]	ptg005533l	31.579	7E-14	71.2	97
WP_038048734.1_cold-shock_protein_[Thermoanaerobaculum_aquaticum]	ptg003498l	71.875	3.82E-23	93.6	94
WP_038048675.1_ABC_transporter_ATP-binding_protein_[Thermoanaerobaculum_aquaticum]	ptg005227l	32.277	5.94E-20	95.1	97
WP_038048572.1_ParA_family_protein_[Thermoanaerobaculum_aquaticum]	ptg003829l	40.323	1.71E-57	200	99
WP_038048546.1_TolC_family_protein_[Thermoanaerobaculum_aquaticum]	ptg004500l	28.947	4.26E-12	73.2	34
WP_038048380.1_tRNA_(cytidine(34)-2'-O)-methyltransferase_[Thermoanaerobaculum_aquaticum]	ptg003973l	46.281	5.04E-28	112	78
WP_038047596.1_NAD(P)-dependent_alcohol_dehydrogenase_[Thermoanaerobaculum_aquaticum]	ptg000693l	35.638	7.49E-13	73.6	55

WP_038047532.1_MerR_family_transcriptional_regulator_[Thermoanaerobaculum_aquaticum]	ptg001925l	50.649	1.72E-19	86.3	61
WP_038046478.1_ParA_family_protein_[Thermoanaerobaculum_aquaticum]	ptg000372l	49.167	3.56E-70	238	89
WP_161685505.1_AEC_family_transporter_[Thermoanaerobaculum_aquaticum]	ptg003831l	28.082	0.000000237	56.2	47
WP_161685439.1_glycosyltransferase_family_2_protein_[Thermoanaerobaculum_aquaticum]	ptg003431l	30.935	1.75E-12	70.5	52
WP_161685322.1_UTP--glucose-1-phosphate_uridylyltransferase_[Thermoanaerobaculum_aquaticum]	ptg002505l	44.487	4.68E-68	233	90
WP_081799985.1_TonB-dependent_receptor_[Thermoanaerobaculum_aquaticum]	ptg004670l	30.882	3.41E-66	179	83
WP_053334984.1_acyl-CoA_thioesterase_[Thermoanaerobaculum_aquaticum]	ptg004075l	36.975	9.43E-14	70.5	87
WP_053334757.1_prohibitin_family_protein_[Thermoanaerobaculum_aquaticum]	ptg005920l	26.667	6.35E-11	66.6	67
WP_038050036.1_glycosyltransferase_family_2_protein_[Thermoanaerobaculum_aquaticum]	ptg004680l	33.99	3.57E-18	89	62
WP_038049873.1_serine_hydroxymethyltransferase_[Thermoanaerobaculum_aquaticum]	ptg000693l	56.897	7.8E-139	442	96
WP_038049776.1_glycosyltransferase_family_2_protein_[Thermoanaerobaculum_aquaticum]	ptg004680l	54.202	1.57E-62	214	98
WP_038049553.1_ion_transporter_[Thermoanaerobaculum_aquaticum]	ptg004266l	28.358	5.92E-17	83.6	98
WP_038048273.1_ATP-binding_protein_[Thermoanaerobaculum_aquaticum]	ptg003363l	40.678	3.94E-15	74.3	84
WP_038048197.1_glycosyltransferase_family_2_protein_[Thermoanaerobaculum_aquaticum]	ptg003871l	35.945	1.07E-27	114	84
WP_038047087.1_site-specific_DNA-methyltransferase_[Thermoanaerobaculum_aquaticum]	ptg002453l	28.102	2.36E-24	107	79
WP_038046978.1_Crp/Fnr_family_transcriptional_regulator_[Thermoanaerobaculum_aquaticum]	ptg002907l	26.761	2.87E-18	86.7	94
WP_038046650.1_ribose-phosphate_pyrophosphokinase_[Thermoanaerobaculum_aquaticum]	ptg004982l	55.663	2.66E-113	363	98
WP_038046591.1_penicillin-binding_protein_2_[Thermoanaerobaculum_aquaticum]	ptg004863l	25.373	3.98E-12	73.6	48
WP_161685569.1_glycosyltransferase_family_1_protein_[Thermoanaerobaculum_aquaticum]	ptg002534l	29.213	3.84E-15	82.8	35
WP_161685515.1_glycosyltransferase_family_1_protein_[Thermoanaerobaculum_aquaticum]	ptg002534l	30.962	7.96E-18	89.7	58
WP_161685460.1_RluA_family_pseudouridine_synthase_[Thermoanaerobaculum_aquaticum]	ptg005204l	35.135	1.27E-11	67	50
WP_081800003.1_glycosyltransferase_family_1_protein_[Thermoanaerobaculum_aquaticum]	ptg002534l	31.839	7.01E-19	93.6	50
WP_053335032.1_glycosyltransferase_family_1_protein_[Thermoanaerobaculum_aquaticum]	ptg002534l	29.153	4E-20	96.3	75
WP_053334911.1_glycosyltransferase_family_1_protein_[Thermoanaerobaculum_aquaticum]	ptg002534l	34.194	2.2E-18	90.9	43
WP_053334910.1_glycosyltransferase_family_1_protein_[Thermoanaerobaculum_aquaticum]	ptg002534l	27.843	9.02E-12	70.5	68
WP_053334854.1_RluA_family_pseudouridine_synthase_[Thermoanaerobaculum_aquaticum]	ptg006031l	33.043	1.75E-27	116	64
WP_053334775.1_PAS_domain-containing_sensor_histidine_kinase_[Thermoanaerobaculum_aquaticum]	ptg005484l	33.846	1.99E-43	177	38
WP_038050188.1_RluA_family_pseudouridine_synthase_[Thermoanaerobaculum_aquaticum]	ptg006031l	43.39	1.98E-68	235	91
WP_038046868.1_UDP-glucose/GDP-mannose_dehydrogenase_family_protein_[Thermoanaerobaculum_aquaticum]	ptg001925l	46.606	8.42E-120	388	98
WP_038046107.1_PAS_domain-containing_sensor_histidine_kinase_[Thermoanaerobaculum_aquaticum]	ptg005484l	32.873	4.05E-43	171	53
WP_152543891.1_serine/threonine-protein_kinase_[Thermoanaerobaculum_aquaticum]	ptg006032l	35.714	2.34E-30	133	29
WP_152543822.1_serine/threonine-protein_kinase_[Thermoanaerobaculum_aquaticum]	ptg003082l	37.132	3.88E-40	165	31
WP_081800115.1_ABC_transporter_ATP-binding_protein_[Thermoanaerobaculum_aquaticum]	ptg004055l	44	7.14E-53	196	43

WP_081799838.1_serine/threonine-protein_kinase_[Thermoanaerobaculum_aquaticum]	ptg005260l	39.224	3.71E-39	155	46
WP_053335218.1_sigma-54_dependent_transcriptional_regulator_[Thermoanaerobaculum_aquaticum]	ptg005150l	40.98	1.83E-99	330	96
WP_053334956.1_serine/threonine-protein_kinase_[Thermoanaerobaculum_aquaticum]	ptg002986l	31.833	2.79E-37	129	46
WP_053334915.1_ABC_transporter_ATP-binding_protein_[Thermoanaerobaculum_aquaticum]	ptg004055l	38.996	2.13E-47	177	83
WP_053334902.1_serine/threonine-protein_kinase_[Thermoanaerobaculum_aquaticum]	ptg002986l	51.397	1.56E-49	150	41
WP_053334820.1_serine/threonine-protein_kinase_[Thermoanaerobaculum_aquaticum]	ptg003082l	33.333	3.87E-45	174	58
WP_053334809.1_ABC_transporter_ATP-binding_protein_[Thermoanaerobaculum_aquaticum]	ptg004055l	30.699	6.03E-45	170	90
WP_053334797.1_sigma-54_dependent_transcriptional_regulator_[Thermoanaerobaculum_aquaticum]	ptg004564l	44.753	4.07E-63	220	96
WP_038050517.1_bifunctional_UDP-sugar_hydrolase/5'-nucleotidase_[Thermoanaerobaculum_aquaticum]	ptg002246l	25.724	8.9E-54	201	93
WP_038050447.1_aminodeoxychorismate/anthranilate_synthase_component_II_[Thermoanaerobaculum_aquaticum]	ptg004525l	54.404	2.73E-61	208	98
WP_038050097.1_serine/threonine-protein_kinase_[Thermoanaerobaculum_aquaticum]	ptg006032l	34.657	2.57E-39	156	55
WP_038049259.1_ABC-F_family_ATP-binding_cassette_domain-containing_protein_[Thermoanaerobaculum_aquaticum]	ptg004403l	31.915	1.17E-54	206	80
WP_200867099.1_efflux_RND_transporter_permease_subunit_[Thermoanaerobaculum_aquaticum]	ptg005692l	28.118	6.58E-40	166	42
WP_152543916.1_cysteine_desulfurase_family_protein_[Thermoanaerobaculum_aquaticum]	ptg005265l	47.383	3.14E-101	332	94
WP_081799888.1_type_IV_pilus_twitching_motility_protein_PilT_[Thermoanaerobaculum_aquaticum]	ptg004468l	51.744	1.74E-104	346	69
WP_081799887.1_type_IV_pilus_twitching_motility_protein_PilT_[Thermoanaerobaculum_aquaticum]	LG13	47.429	2.39E-77	264	90
WP_053335261.1_efflux_RND_transporter_permease_subunit_[Thermoanaerobaculum_aquaticum]	ptg004798l	28.447	4.33E-84	303	98
WP_053334863.1_ABC_transporter_ATP-binding_protein_[Thermoanaerobaculum_aquaticum]	ptg004055l	41.631	3.31E-45	167	73
WP_053334748.1_ABC_transporter_ATP-binding_protein_[Thermoanaerobaculum_aquaticum]	ptg004203l	35.754	3.63E-25	105	97
WP_038050385.1_biopolymer_transporter_ExbD_[Thermoanaerobaculum_aquaticum]	ptg005341l	32.558	2.37E-09	57.8	88
WP_038050108.1_ABC_transporter_ATP-binding_protein_[Thermoanaerobaculum_aquaticum]	ptg004470l	34.783	3.46E-17	85.5	76
WP_038049837.1_class_I_SAM-dependent_rRNA_methyltransferase_[Thermoanaerobaculum_aquaticum]	ptg002871l	31.5	5.86E-34	137	98
WP_038049789.1_cysteine_desulfurase_family_protein_[Thermoanaerobaculum_aquaticum]	ptg004651l	34.872	2.08E-58	208	99
WP_038048843.1_ABC_transporter_ATP-binding_protein_[Thermoanaerobaculum_aquaticum]	ptg004026l	31.111	3.02E-32	130	84
WP_038048838.1_ABC_transporter_ATP-binding_protein_[Thermoanaerobaculum_aquaticum]	ptg004579l	41.096	2.26E-46	171	69
WP_038048633.1_methylated-DNA--[protein]-cysteine_S-methyltransferase_[Thermoanaerobaculum_aquaticum]	ptg005680l	47.573	8.03E-09	57	63
WP_038047371.1_type_IV_pilus_twitching_motility_protein_PilT_[Thermoanaerobaculum_aquaticum]	LG13	48.991	3.94E-97	320	93
WP_038046840.1_biopolymer_transporter_ExbD_[Thermoanaerobaculum_aquaticum]	ptg005754l	33.065	0.0000001	53.1	86
WP_038046352.1_phosphoglycerate_kinase_[Thermoanaerobaculum_aquaticum]	ptg004779l	43.511	8.58E-92	305	99
WP_038046147.1_HU_family_DNA-binding_protein_[Thermoanaerobaculum_aquaticum]	ptg005527l	36.364	1.48E-14	70.9	94
WP_200867088.1_sigma-54_dependent_transcriptional_regulator_[Thermoanaerobaculum_aquaticum]	ptg005150l	39.514	1.37E-96	322	99
WP_161685591.1_RsmB/NOP_family_class_I_SAM-dependent_RNA_methyltransferase_[Thermoanaerobaculum_aquaticum]	ptg003098l	33.333	8.2E-13	75.1	34

WP_152544071.1_ATP-binding_protein,_partial_[Thermoanaerobaculum_aquaticum]	ptg003039l	38.519	1.02E-20	90.1	98
WP_152544009.1_TlyA_family_RNA_methyltransferase_[Thermoanaerobaculum_aquaticum]	ptg004075l	43.969	5.82E-36	139	99
WP_152543979.1_sigma-54_dependent_transcriptional_regulator_[Thermoanaerobaculum_aquaticum]	ptg005150l	40.95	1.07E-101	337	96
WP_081800033.1_NADH-quinone_oxidoreductase_subunit_I_[Thermoanaerobaculum_aquaticum]	ptg004143l	34.615	7.62E-21	92.8	82
WP_081799984.1_DMT_family_transporter_[Thermoanaerobaculum_aquaticum]	ptg004214l	28.435	7.39E-25	108	97
WP_081799930.1_sigma-54_dependent_transcriptional_regulator_[Thermoanaerobaculum_aquaticum]	ptg005150l	40.052	5.21E-80	272	90
WP_053334784.1_bifunctional_oligoribonuclease/PAP_phosphatase_NrnA_[Thermoanaerobaculum_aquaticum]	ptg004026l	32.534	3.79E-24	107	87
WP_038050581.1_DMT_family_transporter_[Thermoanaerobaculum_aquaticum]	ptg002021l	30.097	4.36E-09	61.2	67
WP_038050363.1_Glu/Leu/Phe/Val_dehydrogenase_[Thermoanaerobaculum_aquaticum]	ptg002928l	43.396	4.58E-97	322	99
WP_038050305.1_cation_diffusion_facilitator_family_transporter_[Thermoanaerobaculum_aquaticum]	ptg005301l	29.289	3.21E-27	115	82
WP_038050007.1_sigma-54_dependent_transcriptional_regulator_[Thermoanaerobaculum_aquaticum]	ptg002304l	39.848	1.76E-75	262	80
WP_038049877.1_sigma-54_dependent_transcriptional_regulator_[Thermoanaerobaculum_aquaticum]	ptg004075l	38.478	1.66E-84	287	97
WP_038049535.1_sigma-54_dependent_transcriptional_regulator_[Thermoanaerobaculum_aquaticum]	ptg005150l	37.958	7.21E-77	265	82
WP_038048034.1_universal_stress_protein_[Thermoanaerobaculum_aquaticum]	ptg005333l	25.088	1.11E-10	65.9	97
WP_038047980.1_sigma-54_dependent_transcriptional_regulator_[Thermoanaerobaculum_aquaticum]	ptg003137l	43.177	7.16E-113	369	97
WP_038047962.1_sigma-54_dependent_transcriptional_regulator_[Thermoanaerobaculum_aquaticum]	ptg004564l	38.511	3.47E-81	277	98
WP_038047365.1_sigma-54_dependent_transcriptional_regulator_[Thermoanaerobaculum_aquaticum]	ptg002304l	41.703	3.98E-103	340	99
WP_038047100.1_sigma-54_dependent_transcriptional_regulator_[Thermoanaerobaculum_aquaticum]	ptg003057l	39.062	6.15E-83	282	96
WP_038046904.1_electron_transfer_flavoprotein_subunit_alpha/FixB_family_protein_[Thermoanaerobaculum_aquaticum]	LG19	70.833	5.4E-24	106	63
WP_161685526.1_ABC_transporter_permease_[Thermoanaerobaculum_aquaticum]	ptg003163l	34.42	7.2E-25	108	85
WP_161685364.1_RNA_polymerase_sigma_factor_[Thermoanaerobaculum_aquaticum]	ptg003441l	28.947	1.47E-13	71.6	95
WP_053335282.1_TlpA_disulfide_reductase_family_protein_[Thermoanaerobaculum_aquaticum]	ptg003592l	37.383	9.36E-17	80.5	61
WP_053335249.1_shikimate_kinase_[Thermoanaerobaculum_aquaticum]	ptg004564l	31.847	9.05E-20	89	85
WP_053335154.1_phosphatidate_cytidylyltransferase_[Thermoanaerobaculum_aquaticum]	ptg004895l	45.6	9.27E-22	98.2	44
WP_053335120.1_ABC_transporter_permease_[Thermoanaerobaculum_aquaticum]	ptg003548l	36.19	2.33E-21	98.6	65
WP_053335075.1_TlpA_disulfide_reductase_family_protein_[Thermoanaerobaculum_aquaticum]	ptg004266l	31	0.00000304	49.3	60
WP_038050200.1_ABC_transporter_permease_[Thermoanaerobaculum_aquaticum]	ptg005110l	31.174	3.13E-15	78.6	99
WP_038049337.1_ABC_transporter_ATP-binding_protein_[Thermoanaerobaculum_aquaticum]	ptg005920l	46.512	2.46E-57	199	96
WP_038048577.1_HPr_family_phosphocarrier_protein_[Thermoanaerobaculum_aquaticum]	ptg005709l	42.222	3.41E-15	72.4	96
WP_038048123.1_STAS_domain-containing_protein_[Thermoanaerobaculum_aquaticum]	ptg004689l	35.417	2.3E-10	59.7	81

WP_038047068.1_endonuclease_III_[Thermoanaerobaculum_aquaticum]	ptg005275l	40.994	1.58E-36	139	74
WP_200867127.1_glycosyltransferase_family_2_protein_[Thermoanaerobaculum_aquaticum]	ptg003592l	43.226	1.23E-68	235	95
WP_200867125.1_D-alanine--D-alanine_ligase_family_protein_[Thermoanaerobaculum_aquaticum]	ptg004510l	31.361	8.44E-44	166	90
WP_200867124.1_pseudouridine_synthase_[Thermoanaerobaculum_aquaticum]	ptg004652l	46.218	2.5E-56	199	80
WP_200867116.1_pitrilysin_family_protein_[Thermoanaerobaculum_aquaticum]	ptg002214l	26.452	3.9E-79	285	98
WP_152543864.1_anthranilate_synthase_component_I_family_protein_[Thermoanaerobaculum_aquaticum]	ptg002760l	34.815	2.97E-39	152	74
WP_053335000.1_SCO_family_protein_[Thermoanaerobaculum_aquaticum]	ptg002453l	33.032	5.26E-37	142	81
WP_038050496.1_NADH-quinone_oxidoreductase_subunit_J_[Thermoanaerobaculum_aquaticum]	ptg004143l	32.934	1.89E-09	59.7	90
WP_038048519.1_acyl-CoA_dehydrogenase_family_protein_[Thermoanaerobaculum_aquaticum]	ptg004179l	27.5	1.75E-30	128	93
WP_038046129.1_cyclic_nucleotide-binding_domain-containing_protein_[Thermoanaerobaculum_aquaticum]	ptg004457l	28.448	3.21E-10	64.7	39
WP_152544003.1_ATP-dependent_DNA_helicase_[Thermoanaerobaculum_aquaticum]	ptg002767l	25.676	1.54E-08	62.8	35
WP_152543903.1_glycosyltransferase_family_4_protein_[Thermoanaerobaculum_aquaticum]	ptg003112l	35.878	7.88E-18	89.4	34
WP_152543886.1_pitrilysin_family_protein_[Thermoanaerobaculum_aquaticum]	ptg001828l	25.189	7.74E-28	120	94
WP_053334966.1_TetR/AcrR_family_transcriptional_regulator_[Thermoanaerobaculum_aquaticum]	ptg003861l	29.108	8.34E-14	73.6	93
WP_038048345.1_mechanosensitive_ion_channel_family_protein_[Thermoanaerobaculum_aquaticum]	ptg005903l	28.854	4.02E-11	69.7	49
WP_081800136.1_hemolysin_family_protein_[Thermoanaerobaculum_aquaticum]	ptg005005l	30.199	1.68E-38	152	82
WP_053335260.1_superoxide_dismutase_[Thermoanaerobaculum_aquaticum]	ptg001604l	53.439	6.23E-56	195	84
WP_053335163.1_isoprenylcysteine_carboxylmethyltransferase_family_protein_[Thermoanaerobaculum_aquaticum]	ptg004862l	31.452	2.29E-09	58.5	77
WP_053334841.1_thioredoxin_domain-containing_protein_[Thermoanaerobaculum_aquaticum]	ptg001290l	41.47	1.63E-79	282	49
WP_053334835.1_hemolysin_family_protein_[Thermoanaerobaculum_aquaticum]	ptg002760l	39	1.15E-26	117	47
WP_053334773.1_Xaa-Pro_peptidase_family_protein_[Thermoanaerobaculum_aquaticum]	ptg001995l	30.067	1.12E-45	173	95
WP_053334683.1_divalent-cation_tolerance_protein_CutA_[Thermoanaerobaculum_aquaticum]	ptg002612l	39.773	3.25E-14	71.2	71
WP_038050365.1_D-glycerate_dehydrogenase_[Thermoanaerobaculum_aquaticum]	ptg005692l	45.603	2.24E-42	160	93
WP_038050359.1_beta-ketoacyl-ACP_synthase_III_[Thermoanaerobaculum_aquaticum]	ptg003871l	44.817	1.2E-82	276	97
WP_038050091.1_thiolase_family_protein_[Thermoanaerobaculum_aquaticum]	ptg004745l	48.346	5.07E-98	323	99
WP_038049780.1_TerC_family_protein_[Thermoanaerobaculum_aquaticum]	LG12	43.137	5.79E-14	76.6	63
WP_038049556.1_Xaa-Pro_peptidase_family_protein_[Thermoanaerobaculum_aquaticum]	ptg001995l	25.592	0.0000014	54.7	47
WP_038049421.1_superoxide_dismutase_[Thermoanaerobaculum_aquaticum]	ptg001604l	59.296	1.27E-74	247	98
WP_038048423.1_dihydroorotase_[Thermoanaerobaculum_aquaticum]	LG04	44.268	8.56E-72	228	97
WP_038047741.1_elongation_factor_P_[Thermoanaerobaculum_aquaticum]	ptg003382l	30.645	9.63E-23	98.2	98
WP_038047591.1_thiolase_family_protein_[Thermoanaerobaculum_aquaticum]	ptg005527l	44.73	1.71E-88	295	99

WP_038047482.1_CoA_pyrophosphatase_[Thermoanaerobaculum_aquaticum]	ptg005213l	41.176	7.35E-19	87	61
WP_038046678.1_Lrp/AsnC_family_transcriptional_regulator_[Thermoanaerobaculum_aquaticum]	ptg002871l	33.803	4.21E-15	74.7	93
WP_081800045.1_uracil-DNA_glycosylase_[Thermoanaerobaculum_aquaticum]	ptg003644l	54.494	3.44E-58	202	76
WP_081799988.1_cytochrome_c_[Thermoanaerobaculum_aquaticum]	ptg001690l	40.553	1.77E-36	139	75
WP_081799986.1_cytochrome_c3_family_protein_[Thermoanaerobaculum_aquaticum]	ptg002453l	46.862	1.58E-62	213	95
WP_081799938.1_TonB-dependent_receptor_[Thermoanaerobaculum_aquaticum]	ptg001925l	23.017	3.09E-16	88.2	62
WP_081799896.1_thioredoxin_family_protein_[Thermoanaerobaculum_aquaticum]	ptg003334l	34.091	0.00000019	53.5	49
WP_081799806.1_glycosyltransferase_family_4_protein_[Thermoanaerobaculum_aquaticum]	ptg004077l	29.644	3.03E-13	75.5	61
WP_053335130.1_arsenate_reductase_ArsC_[Thermoanaerobaculum_aquaticum]	ptg004266l	50	1.2E-29	116	76
WP_053335108.1_glycerol-3-phosphate_dehydrogenase/oxidase_[Thermoanaerobaculum_aquaticum]	ptg003827l	27.793	3.22E-26	117	65
WP_053335093.1_inositol_monophosphatase_family_protein_[Thermoanaerobaculum_aquaticum]	ptg005265l	45.07	4.78E-47	171	75
WP_053335058.1_pitrilysin_family_protein_[Thermoanaerobaculum_aquaticum]	ptg005195l	32.895	2.55E-18	93.6	48
WP_053334742.1_glycosyltransferase_family_2_protein_[Thermoanaerobaculum_aquaticum]	ptg003625l	38.735	8.73E-47	171	87
WP_038050297.1_dihydrolipoamide_acetyltransferase_family_protein_[Thermoanaerobaculum_aquaticum]	ptg005110l	40.333	4.48E-58	211	78
WP_038049767.1_thioesterase_family_protein_[Thermoanaerobaculum_aquaticum]	ptg001690l	29.032	5.74E-11	62.8	83
WP_038049245.1_pitrilysin_family_protein_[Thermoanaerobaculum_aquaticum]	ptg002214l	23.009	1.37E-15	84	90
WP_038048821.1_NADP-dependent_malic_enzyme_[Thermoanaerobaculum_aquaticum]	ptg005092l	44.504	0	609	98
WP_038048631.1_nucleoside_triphosphate_pyrophosphatase_[Thermoanaerobaculum_aquaticum]	ptg005709l	41.758	1.45E-21	95.5	89
WP_038046942.1_thioesterase_family_protein_[Thermoanaerobaculum_aquaticum]	ptg003536l	40.8	2.38E-18	83.2	93
WP_038046414.1_glycosyltransferase_family_2_protein_[Thermoanaerobaculum_aquaticum]	ptg003625l	32.157	1.24E-19	91.7	97
WP_053335279.1_glycosyltransferase_family_4_protein_[Thermoanaerobaculum_aquaticum]	ptg003112l	29.121	2.87E-12	72.4	45
WP_053335223.1_glycosyltransferase_family_2_protein_[Thermoanaerobaculum_aquaticum]	ptg001690l	30.328	1.6E-11	69.3	35
WP_053335222.1_glycosyltransferase_family_2_protein_[Thermoanaerobaculum_aquaticum]	ptg004085l	26.432	1.11E-16	84.7	69
WP_053335205.1_sensor_domain-containing_diguanylate_cyclase_[Thermoanaerobaculum_aquaticum]	LG04	37.811	2.92E-24	108	53
WP_053335010.1_cation_diffusion_facilitator_family_transporter_[Thermoanaerobaculum_aquaticum]	ptg004670l	28.727	2.23E-29	121	91
WP_053334981.1_Fur_family_transcriptional_regulator_[Thermoanaerobaculum_aquaticum]	ptg003304l	29.457	2.18E-12	67.4	79
WP_053334960.1_M1_family_metallopeptidase_[Thermoanaerobaculum_aquaticum]	ptg002175l	20.654	9.63E-16	85.9	70
WP_053334906.1_glycosyltransferase_family_2_protein_[Thermoanaerobaculum_aquaticum]	ptg002928l	37.719	4.87E-19	90.1	43
WP_053334827.1_glycosyltransferase_family_2_protein_[Thermoanaerobaculum_aquaticum]	ptg004788l	41.629	5.76E-42	155	93
WP_053334794.1_ABC_transporter_permease_[Thermoanaerobaculum_aquaticum]	ptg005920l	31.975	1.02E-25	114	70
WP_053334793.1_ABC_transporter_permease_[Thermoanaerobaculum_aquaticum]	ptg005920l	26.84	3.18E-14	79	51
WP_038050331.1_MoxR_family_ATPase_[Thermoanaerobaculum_aquaticum]	ptg003999l	49.038	1.04E-81	273	98
WP_038050174.1_(deoxy)nucleoside_triphosphate_pyrophosphohydrolase_[Thermoanaerobaculum_aquaticum]	ptg005005l	37.903	2.03E-15	75.1	91

WP_038049897.1_PLP-dependent_aspartate_aminotransferase_family_protein_[Thermoanaerobaculum_aquaticum]	ptg004895l	42.703	3.97E-88	294	95
WP_038049812.1_TonB-dependent_receptor_[Thermoanaerobaculum_aquaticum]	ptg001925l	25.424	0.00000167	56.2	31
WP_038049463.1_S41_family_peptidase_[Thermoanaerobaculum_aquaticum]	ptg003980l	26.718	2.61E-35	150	82
WP_038049342.1_ABC_transporter_permease_[Thermoanaerobaculum_aquaticum]	ptg004986l	26.728	2.27E-17	88.2	49
WP_038049243.1_rhomboid_family_intramembrane_serine_protease_[Thermoanaerobaculum_aquaticum]	ptg005092l	41.228	8.56E-32	126	94
WP_038048599.1_J_domain-containing_protein_[Thermoanaerobaculum_aquaticum]	ptg005113l	46.479	2.31E-13	71.6	33
WP_038048382.1_YceI_family_protein_[Thermoanaerobaculum_aquaticum]	ptg002928l	32.222	8.4E-17	81.3	88
WP_038048225.1_ABC_transporter_ATP-binding_protein_[Thermoanaerobaculum_aquaticum]	ptg005435l	45.17	9.67E-95	312	99
WP_038048157.1_TonB-dependent_receptor_[Thermoanaerobaculum_aquaticum]	ptg001925l	24.742	0.000000043	61.6	29
WP_038047389.1_GspE/PulE_family_protein_[Thermoanaerobaculum_aquaticum]	ptg000568l	42.505	2.47E-120	394	95
WP_038046643.1_class_I_SAM-dependent_methyltransferase_[Thermoanaerobaculum_aquaticum]	ptg005536l	26.241	0.000000026	60.1	37
WP_038046260.1_sugar_transferase_[Thermoanaerobaculum_aquaticum]	ptg004085l	51.19	8.93E-44	169	35
WP_038046190.1_UbiA-like_polyprenyltransferase_[Thermoanaerobaculum_aquaticum]	ptg003903l	36.877	5.21E-30	122	97
WP_200867162.1_RHS_repeat-associated_core_domain-containing_protein,_partial_[Thermoanaerobaculum_aquaticum]	ptg002381l	57.5	0.000000781	50.4	30
WP_038049290.1_RHS_repeat-associated_core_domain-containing_protein_[Thermoanaerobaculum_aquaticum]	ptg002381l	61.224	6.46E-10	63.5	18
WP_038046427.1_cyclic_dehypoxanthinyl_futalosine_synthase_[Thermoanaerobaculum_aquaticum]	ptg002867l	50.852	4.34E-105	342	99
WP_038047715.1_adenosylhomocysteinase_[Thermoanaerobaculum_aquaticum]	ptg002467l	42.259	2.48E-88	298	99
BCW94516.1_MAG:_undecaprenyl-phosphate_glucose_phosphotransferase_[Thermoanaerobaculum_sp.]	ptg003365l	40.752	1.14E-50	189	67
BCW94515.1_MAG:_glycosyl_transferase_family_1_[Thermoanaerobaculum_sp.]	ptg002534l	27.148	9.19E-11	67.4	75
BCW94514.1_MAG:_glycosyl_transferase_family_1_[Thermoanaerobaculum_sp.]	ptg002534l	29.153	1.11E-19	94.7	75
BCW94512.1_MAG:_glycosyl_transferase_family_1_[Thermoanaerobaculum_sp.]	ptg002534l	33.184	1.05E-20	99	50
BCW94511.1_MAG:_enoyl-CoA_hydratase_[Thermoanaerobaculum_sp.]	ptg001245l	32.819	3.52E-19	90.5	98
BCW94510.1_MAG:_spermidine/putrescine_ABC_transporter_ATP-binding_protein_[Thermoanaerobaculum_sp.]	ptg003163l	35.616	9.7E-48	177	77
BCW94509.1_MAG:_hypothetical_protein_KatS3mg007_2403_[Thermoanaerobaculum_sp.]	ptg005433l	43.119	7.46E-12	68.2	45
BCW94507.1_MAG:_hypothetical_protein_KatS3mg007_2401_[Thermoanaerobaculum_sp.]	ptg005756l	45.775	4.13E-27	115	44
BCW94506.1_MAG:_cyclic_pyranopterin_monophosphate_synthase_accessory_protein_[Thermoanaerobaculum_sp.]	ptg003770l	48.734	3.27E-28	112	97
BCW94505.1_MAG:_GTP_3',8-cyclase_[Thermoanaerobaculum_sp.]	ptg005933l	40.972	1.15E-61	216	89
BCW94504.1_MAG:_molybdopterin_molybdenumtransferase_MoeA_[Thermoanaerobaculum_sp.]	ptg002270l	34.4	1.19E-38	151	94
BCW94503.1_MAG:_hypothetical_protein_KatS3mg007_2397_[Thermoanaerobaculum_sp.]	ptg005732l	35.407	6.69E-22	104	37
BCW94502.1_MAG:_B12-binding_domain-containing_radical_SAM_protein_[Thermoanaerobaculum_sp.]	ptg003511l	25.792	5.67E-09	62.8	41
BCW94498.1_MAG:_alanyl_dipeptidyl_peptidase_[Thermoanaerobaculum_sp.]	ptg004136l	30.917	6.16E-89	308	92
BCW94497.1_MAG:_ribonuclease_G_[Thermoanaerobaculum_sp.]	ptg005709l	39.248	1.5E-96	323	97
BCW94496.1_MAG:_rod_shape-determining_protein_RodA_[Thermoanaerobaculum_sp.]	ptg004510l	31.466	3.44E-13	74.7	64

BCW94490.1_MAG:_peptidylprolyl_isomerase_[Thermoanaerobaculum_sp.]	ptg005527l	27.143	3.45E-20	99.8	54
BCW94489.1_MAG:_Holliday_junction_ATP-dependent_DNA_helicase_RuvA_[Thermoanaerobaculum_sp.]	ptg005341l	34.711	6.33E-11	49.3	95
BCW94488.1_MAG:_Holliday_junction_ATP-dependent_DNA_helicase_RuvB_[Thermoanaerobaculum_sp.]	ptg005341l	57.516	2.37E-106	345	89
BCW94487.1_MAG:_diacylglycerol_kinase_[Thermoanaerobaculum_sp.]	ptg004476l	27.5	4.16E-14	76.3	80
BCW94486.1_MAG:_CoA-binding_protein_[Thermoanaerobaculum_sp.]	ptg002316l	27.711	4.33E-08	60.8	34
BCW94485.1_MAG:_hypothetical_protein_KatS3mg007_2379_[Thermoanaerobaculum_sp.]	ptg003785l	35.669	3.77E-18	84.3	89
BCW94483.1_MAG:_hypothetical_protein_KatS3mg007_2377_[Thermoanaerobaculum_sp.]	ptg002986l	31.833	2.93E-37	129	46
BCW94482.1_MAG:_chaperone_protein_DnaJ_[Thermoanaerobaculum_sp.]	ptg004942l	32.033	1.34E-36	143	95
BCW94473.1_MAG:_hypothetical_protein_KatS3mg007_2367_[Thermoanaerobaculum_sp.]	ptg003947l	27.885	5.29E-45	170	99
BCW94470.1_MAG:_hypothetical_protein_KatS3mg007_2364_[Thermoanaerobaculum_sp.]	ptg005204l	37.5	7.07E-09	59.7	42
BCW94468.1_MAG:_hypothetical_protein_KatS3mg007_2362_[Thermoanaerobaculum_sp.]	ptg005844l	31.548	8.73E-11	66.6	50
BCW94467.1_MAG:_thymidylate_kinase_[Thermoanaerobaculum_sp.]	ptg003947l	36.364	5.64E-36	137	94
BCW94466.1_MAG:_hypothetical_protein_KatS3mg007_2360_[Thermoanaerobaculum_sp.]	ptg004476l	25.556	1.31E-08	63.2	32
BCW94464.1_MAG:_hypothetical_protein_KatS3mg007_2358_[Thermoanaerobaculum_sp.]	ptg003137l	44.805	4.82E-57	208	62
BCW94460.1_MAG:_hypothetical_protein_KatS3mg007_2354_[Thermoanaerobaculum_sp.]	ptg004476l	27.341	1.35E-12	74.7	52
BCW94455.1_MAG:_threonine_aldolase_[Thermoanaerobaculum_sp.]	LG13	61.538	2.89E-10	65.9	25
BCW94454.1_MAG:_DNA_repair_protein_RecN_[Thermoanaerobaculum_sp.]	ptg005922l	33.449	1.19E-58	215	100
BCW94453.1_MAG:_hypothetical_protein_KatS3mg007_2347_[Thermoanaerobaculum_sp.]	ptg005354l	32.24	1.32E-12	69.3	82
BCW94452.1_MAG:_gamma_carbonic_anhydrase_family_protein_[Thermoanaerobaculum_sp.]	ptg004532l	39.884	1.4E-36	137	89
BCW94451.1_MAG:_histidine--tRNA_ligase_[Thermoanaerobaculum_sp.]	ptg003703l	42.336	2.93E-86	290	96
BCW94450.1_MAG:_2-deoxyribose-5-phosphate_aldolase_[Thermoanaerobaculum_sp.]	ptg005596l	31.884	2.9E-16	80.9	83
BCW94445.1_MAG:_hypothetical_protein_KatS3mg007_2339_[Thermoanaerobaculum_sp.]	ptg001814l	49.296	1.65E-35	133	89
BCW94432.1_MAG:_IS256_family_transposase_[Thermoanaerobaculum_sp.]	ptg004058l	25.714	5.36E-08	58.9	68
BCW94428.1_MAG:_hypothetical_protein_KatS3mg007_2322_[Thermoanaerobaculum_sp.]	ptg001814l	44.737	2.25E-38	142	100
BCW94427.1_MAG:_hypothetical_protein_KatS3mg007_2321_[Thermoanaerobaculum_sp.]	ptg002909l	46.429	4.44E-25	105	67
BCW94426.1_MAG:_drug/metabolite_exporter_YedA_[Thermoanaerobaculum_sp.]	ptg002270l	25.279	5.45E-11	67.4	86
BCW94425.1_MAG:_peptidase_M23_[Thermoanaerobaculum_sp.]	ptg004510l	45.69	1.55E-26	112	40
BCW94422.1_MAG:_cytochrome_c_family_protein_[Thermoanaerobaculum_sp.]	ptg002214l	23.989	4.2E-16	85.1	61
BCW94421.1_MAG:_RND_transporter_[Thermoanaerobaculum_sp.]	ptg000732l	25.98	6.64E-11	68.2	52
BCW94420.1_MAG:_macrolide_ABC_transporter_ATP-binding_protein_[Thermoanaerobaculum_sp.]	ptg001659l	50.917	8.68E-62	212	94
BCW94419.1_MAG:_ABC_transporter_permease_[Thermoanaerobaculum_sp.]	ptg005920l	31.975	1.11E-25	113	70
BCW94418.1_MAG:_ABC_transporter_permease_[Thermoanaerobaculum_sp.]	ptg005920l	26.522	1.9E-14	79.7	51
BCW94415.1_MAG:_hypothetical_protein_KatS3mg007_2309_[Thermoanaerobaculum_sp.]	ptg002760l	34.815	2.97E-39	152	74
BCW94413.1_MAG:_hypothetical_protein_KatS3mg007_2307_[Thermoanaerobaculum_sp.]	ptg004982l	31.618	1.46E-22	100	91
BCW94411.1_MAG:_peptide_chain_release_factor_1_[Thermoanaerobaculum_sp.]	ptg004982l	48.295	2.47E-93	309	94

BCW94410.1_MAG:_50S_ribosomal_protein_L31_[Thermoanaerobaculum_sp.]	ptg004959l	53.846	6.47E-20	84.7	96
BCW94409.1_MAG:_sugar_fermentation_stimulation_protein_[Thermoanaerobaculum_sp.]	ptg003691l	52.308	0.000000894	53.5	25
BCW94408.1_MAG:_hypothetical_protein_KatS3mg007_2302_[Thermoanaerobaculum_sp.]	ptg005010l	34.982	2.62E-40	152	94
BCW94407.1_MAG:_chorismate_synthase_[Thermoanaerobaculum_sp.]	ptg001005l	30.816	5.75E-18	90.1	82
BCW94406.1_MAG:_hypothetical_protein_KatS3mg007_2300_[Thermoanaerobaculum_sp.]	ptg002658l	34.429	3.93E-69	247	94
BCW94401.1_MAG:_hypothetical_protein_KatS3mg007_2295_[Thermoanaerobaculum_sp.]	ptg004476l	25	1.87E-24	112	99
BCW94400.1_MAG:_protein-L-isoaspartate_O-methyltransferase_[Thermoanaerobaculum_sp.]	ptg004026l	49.302	3.09E-56	196	90
BCW94399.1_MAG:_amidohydrolase_[Thermoanaerobaculum_sp.]	ptg004680l	30.579	3.76E-10	67	22
BCW94398.1_MAG:_asparaginase_[Thermoanaerobaculum_sp.]	ptg001690l	43.878	6.65E-58	206	82
BCW94394.1_MAG:_RND_transporter_[Thermoanaerobaculum_sp.]	ptg001766l	29.722	1.68E-27	119	88
BCW94393.1_MAG:_cation_transporter_[Thermoanaerobaculum_sp.]	ptg004670l	40.528	0	660	99
BCW94390.1_MAG:_sigma-54-dependent_Fis_family_transcriptional_regulator_[Thermoanaerobaculum_sp.]	ptg003057l	39.286	2.09E-84	286	96
BCW94389.1_MAG:_hypothetical_protein_KatS3mg007_2283_[Thermoanaerobaculum_sp.]	ptg003903l	30.996	1.71E-19	97.4	42
BCW94388.1_MAG:_hypothetical_protein_KatS3mg007_2282_[Thermoanaerobaculum_sp.]	ptg001034l	38.806	0.000000127	52.8	50
BCW94384.1_MAG:_hypothetical_protein_KatS3mg007_2278_[Thermoanaerobaculum_sp.]	ptg001262l	47.865	0	600	99
BCW94383.1_MAG:_polyribonucleotide_nucleotidyltransferase_[Thermoanaerobaculum_sp.]	ptg004026l	48.235	0	652	93
BCW94382.1_MAG:_30S_ribosomal_protein_S15_[Thermoanaerobaculum_sp.]	ptg004026l	46.429	5.95E-21	88.6	92
BCW94378.1_MAG:_tRNA_pseudouridine_synthase_B_[Thermoanaerobaculum_sp.]	ptg003078l	40.551	7.99E-46	169	81
BCW94377.1_MAG:_phosphoesterase_[Thermoanaerobaculum_sp.]	ptg004026l	32.534	3.05E-24	107	87
BCW94376.1_MAG:_ribosome-binding_factor_A_[Thermoanaerobaculum_sp.]	ptg001690l	35.398	9.61E-18	81.3	92
BCW94374.1_MAG:_hypothetical_protein_KatS3mg007_2268_[Thermoanaerobaculum_sp.]	ptg001690l	45.44	1.81E-147	487	65
BCW94373.1_MAG:_transcription_termination/antitermination_protein_NusA_[Thermoanaerobaculum_sp.]	ptg001571l	34.536	8.18E-55	201	80
BCW94371.1_MAG:_malate_dehydrogenase_[Thermoanaerobaculum_sp.]	ptg003019l	50	5.41E-78	262	89
BCW94370.1_MAG:_endonuclease_III_[Thermoanaerobaculum_sp.]	ptg005275l	40.994	2.5E-36	138	74
BCW94368.1_MAG:_GTP-binding_protein_[Thermoanaerobaculum_sp.]	ptg002505l	27.128	0.00000441	52.4	53
BCW94367.1_MAG:_hypothetical_protein_KatS3mg007_2261_[Thermoanaerobaculum_sp.]	ptg003058l	48.148	5.72E-19	82.4	100
BCW94366.1_MAG:_arginine--tRNA_ligase_[Thermoanaerobaculum_sp.]	ptg005820l	32.479	2.22E-08	62	15
BCW94352.1_MAG:_UDP-N-acetylenolpyruvoylglucosamine_reductase_[Thermoanaerobaculum_sp.]	ptg005527l	29.211	2.04E-41	159	99
BCW94351.1_MAG:_hypothetical_protein_KatS3mg007_2245_[Thermoanaerobaculum_sp.]	ptg003336l	32.386	8.14E-46	179	53
BCW94350.1_MAG:_hypothetical_protein_KatS3mg007_2244_[Thermoanaerobaculum_sp.]	ptg004814l	48.014	0	737	92
BCW94349.1_MAG:_hypothetical_protein_KatS3mg007_2243_[Thermoanaerobaculum_sp.]	ptg004814l	26.768	1.07E-11	67.4	87
BCW94348.1_MAG:_octanoyltransferase_LipM_[Thermoanaerobaculum_sp.]	ptg000619l	29.588	2.87E-24	105	89
BCW94347.1_MAG:_ferredoxin--NADP(+)_reductase_[Thermoanaerobaculum_sp.]	ptg004979l	34.167	6.59E-35	136	87
BCW94346.1_MAG:_holo-[acyl-carrier-protein]_synthase_[Thermoanaerobaculum_sp.]	ptg003327l	36.441	3.37E-12	65.1	94

BCW94343.1_MAG:_hypothetical_protein_KatS3mg007_2237_[Thermoanaerobaculum_sp.]	ptg003082l	36.644	1.1E-41	169	33
BCW94342.1_MAG:_protein-glutamate_O-methyltransferase_[Thermoanaerobaculum_sp.]	ptg005265l	33.175	2.56E-27	119	45
BCW94341.1_MAG:_hypothetical_protein_KatS3mg007_2235_[Thermoanaerobaculum_sp.]	LG04	39.053	1.66E-24	111	60
BCW94340.1_MAG:_chemotaxis_response_regulator_protein-glutamate_methylesterase_[Thermoanaerobaculum_sp.]	ptg005550l	37.46	1.26E-40	155	91
BCW94339.1_MAG:_signal_transduction_histidine_kinase_[Thermoanaerobaculum_sp.]	ptg003734l	33.628	6.55E-60	222	67
BCW94338.1_MAG:_hypothetical_protein_KatS3mg007_2232_[Thermoanaerobaculum_sp.]	ptg006036l	34.101	4.26E-14	79.7	38
BCW94335.1_MAG:_hypothetical_protein_KatS3mg007_2229_[Thermoanaerobaculum_sp.]	ptg002844l	25.77	9.46E-26	115	71
BCW94334.1_MAG:_hypothetical_protein_KatS3mg007_2228_[Thermoanaerobaculum_sp.]	ptg004457l	28.448	3.42E-10	64.7	39
BCW94333.1_MAG:_5-methylthioadenosine/S-adenosylhomocysteine_deaminase_[Thermoanaerobaculum_sp.]	ptg002479l	21.186	0.00000614	52.8	73
BCW94332.1_MAG:_hypothetical_protein_KatS3mg007_2226_[Thermoanaerobaculum_sp.]	ptg005010l	30.112	2.23E-30	129	59
BCW94331.1_MAG:_DNA-binding_response_regulator_[Thermoanaerobaculum_sp.]	ptg004579l	39.912	2.29E-49	176	96
BCW94330.1_MAG:_protein_RecA_[Thermoanaerobaculum_sp.]	ptg002699l	65.325	2.23E-124	397	94
BCW94329.1_MAG:_oxidoreductase_[Thermoanaerobaculum_sp.]	ptg004026l	33.533	2.95E-40	154	96
BCW94328.1_MAG:_sigma-54-dependent_Fis_family_transcriptional_regulator_[Thermoanaerobaculum_sp.]	ptg005150l	39.735	5.81E-98	326	99
BCW94324.1_MAG:_L-lysine_6-transaminase_[Thermoanaerobaculum_sp.]	ptg002126l	27.941	9.41E-33	135	89
BCW94323.1_MAG:_translation_initiation_factor_IF-3_[Thermoanaerobaculum_sp.]	ptg001558l	44.138	2.38E-33	127	87
BCW94318.1_MAG:_nicotinamidase_[Thermoanaerobaculum_sp.]	ptg002634l	53.211	6.37E-45	124	99
BCW94317.1_MAG:_SPBc2_prophage-derived_DNA-binding_protein_HU_2_[Thermoanaerobaculum_sp.]	ptg005527l	35.227	2.6E-14	70.1	94
BCW94316.1_MAG:_ribosomal_RNA_small_subunit_methyltransferase_B_[Thermoanaerobaculum_sp.]	ptg003098l	27.857	1.21E-15	83.6	63
BCW94315.1_MAG:_methionyl-tRNA_formyltransferase_[Thermoanaerobaculum_sp.]	ptg003098l	33.54	8.99E-57	201	99
BCW94312.1_MAG:_hypothetical_protein_KatS3mg007_2206_[Thermoanaerobaculum_sp.]	ptg002381l	35.758	1.94E-18	96.7	15
BCW94310.1_MAG:_hypothetical_protein_KatS3mg007_2204_[Thermoanaerobaculum_sp.]	ptg002381l	32.584	5.97E-20	95.9	42
BCW94308.1_MAG:_hypothetical_protein_KatS3mg007_2202_[Thermoanaerobaculum_sp.]	ptg002381l	46.875	1.46E-10	64.7	25
BCW94306.1_MAG:_hypothetical_protein_KatS3mg007_2200_[Thermoanaerobaculum_sp.]	ptg004724l	20.321	0.00000655	53.5	52
BCW94305.1_MAG:_superoxide_dismutase_[Thermoanaerobaculum_sp.]	ptg001604l	53.439	7.3E-56	195	79
BCW94304.1_MAG:_tetracycline_resistance_MFS_efflux_pump_[Thermoanaerobaculum_sp.]	ptg003830l	32.973	1.46E-35	142	90
BCW94302.1_MAG:_glutamate_synthase_(NADPH),_homotetrameric_[Thermoanaerobaculum_sp.]	ptg005689l	27.451	1.24E-10	68.2	67
BCW94301.1_MAG:_hypothetical_protein_KatS3mg007_2195_[Thermoanaerobaculum_sp.]	ptg004790l	47	1.01E-25	103	86
BCW94300.1_MAG:_uridine_kinase_[Thermoanaerobaculum_sp.]	ptg003625l	27.072	2.72E-08	60.8	32
BCW94299.1_MAG:_serine_acetyltransferase_[Thermoanaerobaculum_sp.]	ptg003839l	52.756	7.56E-72	244	78
BCW94295.1_MAG:_hypothetical_protein_KatS3mg007_2189_[Thermoanaerobaculum_sp.]	ptg005754l	37.805	4.52E-09	60.1	33
BCW94294.1_MAG:_protein_TolQ_[Thermoanaerobaculum_sp.]	ptg005341l	36.126	9.19E-19	88.2	82
BCW94293.1_MAG:_biopolymer_transporter_ExbD_[Thermoanaerobaculum_sp.]	ptg005341l	31.783	1.25E-08	55.8	88
BCW94290.1_MAG:_queuine_tRNA-ribosyltransferase_[Thermoanaerobaculum_sp.]	ptg002867l	45.245	1.11E-91	304	93

BCW94289.1_MAG:_hypothetical_protein_KatS3mg007_2183_[Thermoanaerobaculum_sp.]	ptg005746l	39.394	2.44E-10	58.9	67
BCW94288.1_MAG:_protein_translocase_subunit_SecD_[Thermoanaerobaculum_sp.]	ptg005746l	38.664	6.97E-79	273	92
BCW94287.1_MAG:_hypothetical_protein_KatS3mg007_2181_[Thermoanaerobaculum_sp.]	ptg004256l	47.305	9.73E-43	163	43
BCW94286.1_MAG:_GTP_pyrophosphokinase_[Thermoanaerobaculum_sp.]	ptg002030l	33.011	5.45E-118	395	95
BCW94285.1_MAG:_50S_ribosomal_protein_L28_[Thermoanaerobaculum_sp.]	ptg003250l	38.462	7.63E-11	58.5	100
BCW94284.1_MAG:_pilus_assembly_protein_PilM_[Thermoanaerobaculum_sp.]	ptg004863l	30.484	1.69E-37	146	98
BCW94283.1_MAG:_hypothetical_protein_KatS3mg007_2177_[Thermoanaerobaculum_sp.]	ptg004863l	31.579	1.75E-12	68.9	74
BCW94282.1_MAG:_hypothetical_protein_KatS3mg007_2176_[Thermoanaerobaculum_sp.]	ptg004771l	28.689	1.96E-08	57	62
BCW94280.1_MAG:_hypothetical_protein_KatS3mg007_2174_[Thermoanaerobaculum_sp.]	ptg004771l	34.242	2.87E-51	185	50
BCW94276.1_MAG:_hypothetical_protein_KatS3mg007_2170_[Thermoanaerobaculum_sp.]	LG01	29.795	1.88E-10	67	73
BCW94273.1_MAG:_acetyl-CoA_carboxylase,_biotin_carboxyl_carrier_protein_[Thermoanaerobaculum_sp.]	ptg004240l	48.571	7.88E-12	66.2	41
BCW94272.1_MAG:_acetyl-CoA_carboxylase_biotin_carboxylase_subunit_[Thermoanaerobaculum_sp.]	ptg004240l	55.53	4.18E-142	452	99
BCW94271.1_MAG:_shikimate_kinase_[Thermoanaerobaculum_sp.]	ptg004564l	31.847	8.22E-20	89.4	85
BCW94270.1_MAG:_metallophosphoesterase_[Thermoanaerobaculum_sp.]	ptg005702l	40.244	2.4E-16	58.2	99
BCW94269.1_MAG:_hypothetical_protein_KatS3mg007_2163_[Thermoanaerobaculum_sp.]	ptg003583l	44.853	1.1E-17	92.8	16
BCW94268.1_MAG:_D-glycerate_dehydrogenase_[Thermoanaerobaculum_sp.]	ptg005692l	44.951	4.24E-41	156	93
BCW94267.1_MAG:_membrane_protein_[Thermoanaerobaculum_sp.]	ptg005005l	29.915	5.56E-38	150	82
BCW94266.1_MAG:_glutamate_dehydrogenase_[Thermoanaerobaculum_sp.]	ptg002928l	43.75	6.12E-96	318	99
BCW94265.1_MAG:_RNA_polymerase_sigma24_factor_[Thermoanaerobaculum_sp.]	ptg002465l	36.813	1.35E-27	114	77
BCW94259.1_MAG:_3-oxoacyl-[acyl-carrier-protein]_synthase_3_[Thermoanaerobaculum_sp.]	ptg003871l	44.512	1.12E-81	273	97
BCW94258.1_MAG:_malonyl_CoA-acyl_carrier_protein_transacylase_[Thermoanaerobaculum_sp.]	ptg004340l	42.123	1.69E-60	211	94
BCW94257.1_MAG:_beta-ketoacyl-ACP_reductase_[Thermoanaerobaculum_sp.]	ptg005905l	45.935	9.47E-61	209	99
BCW94255.1_MAG:_aspartate--tRNA(Asp/Asn)_ligase_[Thermoanaerobaculum_sp.]	ptg003448l	46.907	4.57E-156	499	98
BCW94254.1_MAG:_ribonuclease_R_[Thermoanaerobaculum_sp.]	ptg004242l	34.513	2.21E-85	301	86
BCW94247.1_MAG:_cell_division_protein_FtsZ_[Thermoanaerobaculum_sp.]	ptg004510l	48.966	1.68E-54	197	74
BCW94244.1_MAG:_hypothetical_protein_KatS3mg007_2138_[Thermoanaerobaculum_sp.]	ptg004510l	36.972	5.79E-47	172	89
BCW94243.1_MAG:_hypothetical_protein_KatS3mg007_2137_[Thermoanaerobaculum_sp.]	ptg004510l	52.74	6.74E-27	109	88
BCW94242.1_MAG:_UDP-N-acetylglucosamine--N-acetylmuramyl-(pentapeptide)_pyrophosphoryl-undecaprenol_N-acetylglucosamine_transferase_[Thermoanaerobaculum_sp.]	ptg004510l	34.795	5.62E-36	142	94
BCW94240.1_MAG:_UDP-N-acetylmuramoylalanine--D-glutamate_ligase_[Thermoanaerobaculum_sp.]	ptg003536l	30.028	6.52E-09	62.4	69
BCW94237.1_MAG:_hypothetical_protein_KatS3mg007_2131_[Thermoanaerobaculum_sp.]	ptg003536l	29.956	2.55E-32	141	43
BCW94235.1_MAG:_ribosomal_RNA_small_subunit_methyltransferase_H_[Thermoanaerobaculum_sp.]	ptg003204l	38.361	7.89E-48	175	98
BCW94232.1_MAG:_PTS_transporter_subunit_IIA_[Thermoanaerobaculum_sp.]	ptg004997l	30.909	1.07E-11	68.6	58
BCW94227.1_MAG:_CDP-diacylglycerol--serine_O-phosphatidyltransferase_[Thermoanaerobaculum_sp.]	ptg002304l	41.423	1.22E-46	169	92

BCW94218.1_MAG:_hypothetical_protein_KatS3mg007_2112_[Thermoanaerobaculum_sp.]	ptg003980l	24.706	0.000000607	55.5	59
BCW94217.1_MAG:_beta-ketoacyl_synthase_[Thermoanaerobaculum_sp.]	ptg002723l	42.857	3.56E-49	182	51
BCW94216.1_MAG:_hypothetical_protein_KatS3mg007_2110_[Thermoanaerobaculum_sp.]	ptg003839l	60.324	5.24E-87	288	75
BCW94215.1_MAG:_peptidase_M24_[Thermoanaerobaculum_sp.]	ptg001995l	25.592	0.00000141	54.7	47
BCW94212.1_MAG:_ion_transporter_[Thermoanaerobaculum_sp.]	ptg002738l	38.028	6.18E-10	62.8	28
BCW94211.1_MAG:_cadmium_transporter_[Thermoanaerobaculum_sp.]	ptg003880l	33.582	9.97E-49	189	75
BCW94210.1_MAG:_hypothetical_protein_KatS3mg007_2104_[Thermoanaerobaculum_sp.]	ptg005046l	35.106	2.85E-10	59.3	74
BCW94209.1_MAG:_RNA_3'-terminal_phosphate_cyclase_[Thermoanaerobaculum_sp.]	ptg005891l	45.058	1.42E-75	256	98
BCW94208.1_MAG:_exodeoxyribonuclease_III_[Thermoanaerobaculum_sp.]	ptg003839l	29.688	7.47E-30	121	97
BCW94207.1_MAG:_hypothetical_protein_KatS3mg007_2101_[Thermoanaerobaculum_sp.]	ptg004917l	46.429	0	609	55
BCW94205.1_MAG:_UDP-3-O-acylglucosamine_N-acyltransferase_3_[Thermoanaerobaculum_sp.]	ptg003363l	40.645	3.97E-63	221	90
BCW94204.1_MAG:_3-hydroxyacyl-[acyl-carrier-protein]_dehydratase_FabZ_[Thermoanaerobaculum_sp.]	ptg004895l	48.201	8.95E-37	136	96
BCW94203.1_MAG:_acyl-[acyl-carrier-protein]--UDP-N-acetylglucosamine_O-acyltransferase_[Thermoanaerobaculum_sp.]	ptg004895l	39.113	9.4E-59	204	95
BCW94201.1_MAG:_hypothetical_protein_KatS3mg007_2095_[Thermoanaerobaculum_sp.]	ptg005301l	31.016	3.57E-19	95.5	36
BCW94200.1_MAG:_protease_HtpX_[Thermoanaerobaculum_sp.]	ptg005195l	34.51	4.87E-24	105	85
BCW94198.1_MAG:_sigma-54-dependent_Fis_family_transcriptional_regulator_[Thermoanaerobaculum_sp.]	ptg005150l	37.958	5.29E-78	268	82
BCW94197.1_MAG:_hypothetical_protein_KatS3mg007_2091_[Thermoanaerobaculum_sp.]	ptg003548l	36.19	2.33E-21	98.6	65
BCW94196.1_MAG:_peptide_ABC_transporter_permease_[Thermoanaerobaculum_sp.]	ptg003163l	34.783	6.76E-26	112	86
BCW94195.1_MAG:_ABC_transporter_substrate-binding_protein_[Thermoanaerobaculum_sp.]	ptg005227l	24.779	3.03E-22	105	80
BCW94194.1_MAG:_sensor_histidine_kinase_[Thermoanaerobaculum_sp.]	ptg001909l	40.909	3.24E-09	63.5	19
BCW94190.1_MAG:_thymidine_kinase_[Thermoanaerobaculum_sp.]	ptg004623l	26.816	1.54E-09	60.5	82
BCW94189.1_MAG:_endonuclease_III_[Thermoanaerobaculum_sp.]	ptg005275l	44.39	3.32E-45	163	96
BCW94188.1_MAG:_membrane_protein_[Thermoanaerobaculum_sp.]	ptg002909l	27.103	1.21E-13	73.2	86
BCW94183.1_MAG:_catalase-peroxidase_[Thermoanaerobaculum_sp.]	ptg003304l	61.188	0	901	98
BCW94182.1_MAG:_2-hydroxyhepta-2,4-diene-1,7-dioate_isomerase_[Thermoanaerobaculum_sp.]	ptg003204l	45.5	1.24E-42	158	74
BCW94181.1_MAG:_potassium_channel_protein_[Thermoanaerobaculum_sp.]	ptg001878l	29.825	3.02E-30	125	67
BCW94180.1_MAG:_alpha/beta_hydrolase_fold_protein_[Thermoanaerobaculum_sp.]	ptg004764l	29.126	4.38E-10	63.9	73
BCW94179.1_MAG:_thioredoxin_[Thermoanaerobaculum_sp.]	ptg003762l	50	1.33E-30	117	97
BCW94178.1_MAG:_ribosomal_RNA_small_subunit_methyltransferase_A_[Thermoanaerobaculum_sp.]	ptg004306l	33.533	4.95E-18	87	61
BCW94176.1_MAG:_undecaprenyl-diphosphatase_[Thermoanaerobaculum_sp.]	ptg003644l	29.963	6.21E-18	87	98
BCW94175.1_MAG:_hypothetical_protein_KatS3mg007_2069_[Thermoanaerobaculum_sp.]	ptg004538l	48.017	6.69E-121	405	58
BCW94173.1_MAG:_hypothetical_protein_KatS3mg007_2067_[Thermoanaerobaculum_sp.]	ptg002634l	36.111	1.7E-14	70.9	99
BCW94171.1_MAG:_cysteine_desulfurase_[Thermoanaerobaculum_sp.]	ptg005671l	46.684	1.17E-113	369	94
BCW94170.1_MAG:_Fe-S_cluster_assembly_protein_SufD_[Thermoanaerobaculum_sp.]	ptg003472l	34.551	4.84E-43	165	71

BCW94169.1_MAG:_Fe-S_cluster_assembly_protein_SufB_[Thermoanaerobaculum_sp.]	ptg002634l	63.559	0	611	99
BCW94168.1_MAG:_ABC_transporter_ATP-binding_protein_[Thermoanaerobaculum_sp.]	ptg003472l	55.2	3.37E-81	268	96
BCW94164.1_MAG:_sodium/hydrogen_exchanger_[Thermoanaerobaculum_sp.]	ptg003703l	25.845	1.31E-25	115	91
BCW94163.1_MAG:_propionyl-CoA_carboxylase_subunit_beta_[Thermoanaerobaculum_sp.]	ptg002612l	51.391	1.55E-176	556	97
BCW94161.1_MAG:_hypothetical_protein_KatS3mg007_2055_[Thermoanaerobaculum_sp.]	ptg005484l	29.026	5.51E-40	164	59
BCW94160.1_MAG:_hydrolase_[Thermoanaerobaculum_sp.]	ptg004987l	35.377	3.21E-35	135	99
BCW94159.1_MAG:_purine_nucleoside_phosphorylase_[Thermoanaerobaculum_sp.]	ptg002723l	32.308	7.89E-38	145	94
BCW94158.1_MAG:_lysine--tRNA_ligase_[Thermoanaerobaculum_sp.]	ptg004479l	48.303	7.61E-136	436	98
BCW94156.1_MAG:_lipoprotein-releasing_system_ATP-binding_protein_LolD_[Thermoanaerobaculum_sp.]	ptg002820l	42.857	2.4E-44	161	98
BCW94155.1_MAG:_ATP-dependent_Clp_protease_ATP-binding_subunit_ClpC_[Thermoanaerobaculum_sp.]	ptg003199l	45.663	0	640	99
BCW94154.1_MAG:_outer_membrane_protein_assembly_factor_BamA_[Thermoanaerobaculum_sp.]	ptg005656l	25.904	3.18E-40	164	80
BCW94153.1_MAG:_tricorn_protease_[Thermoanaerobaculum_sp.]	ptg003980l	26.718	3.31E-36	154	82
BCW94146.1_MAG:_hypothetical_protein_KatS3mg007_2040_[Thermoanaerobaculum_sp.]	ptg000819l	33.929	0.000000145	59.7	13
BCW94138.1_MAG:_FAD-dependent_glycerol-3-phosphate_dehydrogenase_[Thermoanaerobaculum_sp.]	ptg003827l	27.52	4.86E-26	117	65
BCW94137.1_MAG:_diacylglycerol_kinase_[Thermoanaerobaculum_sp.]	ptg004476l	25	7.98E-09	60.5	96
BCW94133.1_MAG:_hypothetical_protein_KatS3mg007_2027_[Thermoanaerobaculum_sp.]	ptg002534l	26.962	1.52E-14	79.7	69
BCW94129.1_MAG:_GDP-mannose_4,6-dehydratase_[Thermoanaerobaculum_sp.]	ptg006001l	56.725	5.03E-123	393	97
BCW94128.1_MAG:_demethylmenaquinone_methyltransferase_[Thermoanaerobaculum_sp.]	ptg004863l	30.508	1.57E-25	108	91
BCW94126.1_MAG:_MBL_fold_hydrolase_[Thermoanaerobaculum_sp.]	ptg003382l	39.53	9.75E-96	320	99
BCW94125.1_MAG:_superoxide_dismutase_[Mn]_[Thermoanaerobaculum_sp.]	ptg001604l	59.296	1.27E-74	247	98
BCW94123.1_MAG:_5-methylthioadenosine/S-adenosylhomocysteine_deaminase_[Thermoanaerobaculum_sp.]	ptg004359l	26.119	1.61E-13	77	80
BCW94122.1_MAG:_hypothetical_protein_KatS3mg007_2016_[Thermoanaerobaculum_sp.]	ptg005195l	42.529	5.39E-27	110	87
BCW94121.1_MAG:_hypothetical_protein_KatS3mg007_2015_[Thermoanaerobaculum_sp.]	ptg004147l	26.457	1.12E-09	63.2	73
BCW94120.1_MAG:_guanylate_kinase_[Thermoanaerobaculum_sp.]	ptg004759l	36.842	6.56E-32	125	88
BCW94118.1_MAG:_peptidase_ClpP_[Thermoanaerobaculum_sp.]	ptg002199l	37.19	1.54E-58	209	90
BCW94117.1_MAG:_hypothetical_protein_KatS3mg007_2011_[Thermoanaerobaculum_sp.]	ptg003644l	53.933	9.66E-58	200	76
BCW94116.1_MAG:_hypothetical_protein_KatS3mg007_2010_[Thermoanaerobaculum_sp.]	ptg005035l	32.819	6.17E-31	132	41
BCW94113.1_MAG:_signal_recognition_particle-docking_protein_FtsY_[Thermoanaerobaculum_sp.]	ptg004885l	48.77	6.3E-45	166	82
BCW94112.1_MAG:_riboflavin_biosynthesis_protein_RibD_[Thermoanaerobaculum_sp.]	ptg002928l	37.088	6.29E-43	163	95
BCW94111.1_MAG:_riboflavin_synthase_subunit_alpha_[Thermoanaerobaculum_sp.]	ptg003830l	46.231	9.43E-36	135	98
BCW94109.1_MAG:_coproporphyrinogen_III_oxidase_[Thermoanaerobaculum_sp.]	ptg003971l	36.778	2.19E-46	173	87
BCW94105.1_MAG:_inositol_monophosphatase_[Thermoanaerobaculum_sp.]	ptg005265l	45.54	1.26E-47	173	75
BCW94104.1_MAG:_hypothetical_protein_KatS3mg007_1998_[Thermoanaerobaculum_sp.]	LG04	39.073	1.69E-25	112	38
BCW94098.1_MAG:_transporter_[Thermoanaerobaculum_sp.]	ptg003831l	28.082	0.000000271	55.8	47
BCW94097.1_MAG:_enoyl-CoA_hydratase_[Thermoanaerobaculum_sp.]	ptg001245l	34.946	2.16E-16	82	72
BCW94096.1_MAG:_NAD-dependent_protein_deacylase_[Thermoanaerobaculum_sp.]	ptg004476l	38.624	8.59E-34	132	74

BCW94095.1_MAG:_UPF0758_protein_[Thermoanaerobaculum_sp.]	ptg002199l	35	1.6E-24	104	98
BCW94094.1_MAG:_leucine--tRNA_ligase_[Thermoanaerobaculum_sp.]	ptg002867l	37.565	8.04E-178	572	99
BCW94092.1_MAG:_putative_tRNA_sulfurtransferase_[Thermoanaerobaculum_sp.]	ptg004075l	33.217	3.56E-35	141	72
BCW94091.1_MAG:_hypothetical_protein_KatS3mg007_1985_[Thermoanaerobaculum_sp.]	ptg005030l	37.234	2.12E-10	65.1	31
BCW94087.1_MAG:_hypothetical_protein_KatS3mg007_1981_[Thermoanaerobaculum_sp.]	ptg004457l	32.381	0.000000014	58.9	42
BCW94086.1_MAG:_hypothetical_protein_KatS3mg007_1980_[Thermoanaerobaculum_sp.]	ptg001025l	48.416	1.89E-50	185	66
BCW94078.1_MAG:_NHLP_family_bacteriocin_export_ABC_transporter_peptidase/permease/ATPase_[Thermoanaerobaculum_sp.]	ptg004210l	31.308	6.22E-78	277	73
BCW94077.1_MAG:_hypothetical_protein_KatS3mg007_1971_[Thermoanaerobaculum_sp.]	ptg005433l	29.032	8.23E-38	159	42
BCW94075.1_MAG:_transposase_for_insertion_sequence_element_ISRM5_[Thermoanaerobaculum_sp.]	ptg004058l	25.357	4.19E-08	59.3	73
BCW94071.1_MAG:_peptidase_C69_[Thermoanaerobaculum_sp.]	LG02	39.13	0.00000123	55.5	13
BCW94065.1_MAG:_hypothetical_protein_KatS3mg007_1959_[Thermoanaerobaculum_sp.]	ptg003813l	33.641	1.8E-60	219	92
BCW94064.1_MAG:_adenylosuccinate_synthetase_[Thermoanaerobaculum_sp.]	ptg005274l	44.048	1.34E-79	272	95
BCW94063.1_MAG:_ferritin_[Thermoanaerobaculum_sp.]	ptg001693l	49.351	2.23E-41	150	93
BCW94062.1_MAG:_peptidase_S9_[Thermoanaerobaculum_sp.]	ptg004136l	26.366	1.49E-24	115	46
BCW94058.1_MAG:_fructose-1,6-bisphosphatase_[Thermoanaerobaculum_sp.]	ptg004284l	46.377	4.43E-56	199	84
BCW94056.1_MAG:_aminoacyl-histidine_dipeptidase_[Thermoanaerobaculum_sp.]	ptg003913l	42.739	2.06E-114	374	99
BCW94055.1_MAG:_2,4-dienoyl-CoA_reductase_[Thermoanaerobaculum_sp.]	ptg005905l	31.739	7.92E-23	101	87
BCW94050.1_MAG:_hypothetical_protein_KatS3mg007_1944_[Thermoanaerobaculum_sp.]	ptg004732l	34.091	3.88E-08	54.3	62
BCW94048.1_MAG:_ABC_transporter_ATP-binding_protein_[Thermoanaerobaculum_sp.]	ptg004055l	41.631	4.9E-45	167	73
BCW94047.1_MAG:_hypothetical_protein_KatS3mg007_1941_[Thermoanaerobaculum_sp.]	ptg003334l	34.375	0.00000019	53.5	56
BCW94046.1_MAG:_NAD-dependent_nucleoside_diphosphate-sugar_epimerase/dehydratase_[Thermoanaerobaculum_sp.]	ptg003045l	26.891	1.63E-16	84.3	71
BCW94044.1_MAG:_hypothetical_protein_KatS3mg007_1938_[Thermoanaerobaculum_sp.]	ptg002316l	38.393	1.25E-19	94.7	31
BCW94043.1_MAG:_hypothetical_protein_KatS3mg007_1937_[Thermoanaerobaculum_sp.]	ptg002021l	32.278	0.000000277	58.2	25
BCW94039.1_MAG:_hypothetical_protein_KatS3mg007_1933_[Thermoanaerobaculum_sp.]	ptg000819l	41.935	5.34E-26	115	34
BCW94038.1_MAG:_hypothetical_protein_KatS3mg007_1932_[Thermoanaerobaculum_sp.]	ptg003213l	32.374	0.00000602	51.6	46
BCW94037.1_MAG:_glycosyl_transferase_[Thermoanaerobaculum_sp.]	ptg003112l	35.878	4.95E-19	93.2	34
BCW94036.1_MAG:_10_kDa_chaperonin_[Thermoanaerobaculum_sp.]	ptg004085l	48.352	1.32E-21	90.9	95
BCW94035.1_MAG:_60_kDa_chaperonin_[Thermoanaerobaculum_sp.]	ptg002031l	61.29	0	616	97
BCW94034.1_MAG:_elongation_factor_P_[Thermoanaerobaculum_sp.]	ptg003382l	30.645	8.66E-23	98.6	98
BCW94029.1_MAG:_MEMO1_family_protein_[Thermoanaerobaculum_sp.]	LG16	28.571	4.45E-20	98.2	51
BCW94027.1_MAG:_alkaline_phosphatase_[Thermoanaerobaculum_sp.]	ptg005903l	26.936	3.17E-14	80.5	42
BCW94026.1_MAG:_hypothetical_protein_KatS3mg007_1920_[Thermoanaerobaculum_sp.]	ptg004905l	35.932	5.02E-43	164	72
BCW94015.1_MAG:_adenosylhomocysteinase_[Thermoanaerobaculum_sp.]	ptg002467l	42.259	2.41E-89	300	99
BCW94014.1_MAG:_branched-chain-amino-acid_aminotransferase_[Thermoanaerobaculum_sp.]	ptg005886l	37.415	6.84E-13	73.9	41
BCW94013.1_MAG:_ATPase_[Thermoanaerobaculum_sp.]	LG28	40.517	2.12E-12	70.1	47

BCW94012.1_MAG:_hypothetical_protein_KatS3mg007_1906_[Thermoanaerobaculum_sp.]	ptg000568l	37.838	1.63E-14	73.9	79
BCW94011.1_MAG:_GTP_cyclohydrolase_1_type_2_[Thermoanaerobaculum_sp.]	ptg002270l	36.508	3.41E-39	148	96
BCW94010.1_MAG:_competence/damage-inducible_protein_A_[Thermoanaerobaculum_sp.]	ptg005121l	27.703	5.15E-25	111	69
BCW94009.1_MAG:_RNA_2',3'-cyclic_phosphodiesterase_[Thermoanaerobaculum_sp.]	ptg003223l	28.177	7.18E-12	66.6	95
BCW94008.1_MAG:_glycerol-3-phosphate_acyltransferase_[Thermoanaerobaculum_sp.]	LG04	45.745	1.34E-11	66.2	48
BCW94007.1_MAG:_glycerol-3-phosphate_dehydrogenase_[NAD(P)+]_[Thermoanaerobaculum_sp.]	ptg001005l	38.066	1.11E-46	172	99
BCW94006.1_MAG:_pseudouridine_synthase_[Thermoanaerobaculum_sp.]	ptg006031l	32.301	4.94E-27	115	64
BCW94005.1_MAG:_hypothetical_protein_KatS3mg007_1899_[Thermoanaerobaculum_sp.]	ptg005614l	26.244	2.2E-13	74.3	70
BCW94004.1_MAG:_signal_recognition_particle_protein_[Thermoanaerobaculum_sp.]	ptg002916l	46.977	1.96E-94	315	97
BCW94003.1_MAG:_30S_ribosomal_protein_S16_[Thermoanaerobaculum_sp.]	ptg005431l	45	1.07E-16	75.9	100
BCW94000.1_MAG:_tRNA_(guanine-N(1)-)-methyltransferase_[Thermoanaerobaculum_sp.]	ptg005017l	46.047	2.94E-60	209	79
BCW93999.1_MAG:_50S_ribosomal_protein_L19_[Thermoanaerobaculum_sp.]	ptg005431l	54.955	3.79E-30	116	97
BCW93998.1_MAG:_ribonuclease_HII_[Thermoanaerobaculum_sp.]	ptg002436l	42.938	1E-34	132	96
BCW93992.1_MAG:_hypothetical_protein_KatS3mg007_1886_[Thermoanaerobaculum_sp.]	ptg004862l	29.73	0.00000812	52	30
BCW93990.1_MAG:_epoxyqueuosine_reductase_[Thermoanaerobaculum_sp.]	ptg005839l	39.024	2.62E-51	185	90
BCW93988.1_MAG:_hypothetical_protein_KatS3mg007_1882_[Thermoanaerobaculum_sp.]	ptg004982l	40	7.1E-17	82.4	24
BCW93986.1_MAG:_4-diphosphocytidyl-2-C-methyl-D-erythritol_kinase_[Thermoanaerobaculum_sp.]	ptg004982l	30.282	7.7E-19	90.9	90
BCW93985.1_MAG:_hypothetical_protein_KatS3mg007_1879_[Thermoanaerobaculum_sp.]	ptg003363l	41.667	0.000000216	51.2	50
BCW93975.1_MAG:_inosine-5'-monophosphate_dehydrogenase_[Thermoanaerobaculum_sp.]	ptg002610l	57.35	4.19E-174	546	99
BCW93974.1_MAG:_hypothetical_protein_KatS3mg007_1868_[Thermoanaerobaculum_sp.]	ptg003364l	38.202	0.000000213	54.3	42
BCW93972.1_MAG:_thioredoxin_domain-containing_protein_[Thermoanaerobaculum_sp.]	ptg001290l	36.842	1.02E-80	286	59
BCW93971.1_MAG:_hypothetical_protein_KatS3mg007_1865_[Thermoanaerobaculum_sp.]	ptg005886l	35.34	4.26E-54	195	99
BCW93970.1_MAG:_hypothetical_protein_KatS3mg007_1864_[Thermoanaerobaculum_sp.]	ptg004468l	51.744	8.83E-105	347	69
BCW93968.1_MAG:_cadmium_transporter_ATPase_[Thermoanaerobaculum_sp.]	ptg003880l	31.919	1.13E-51	197	75
BCW93963.1_MAG:_RNA_polymerase_subunit_sigma-24_[Thermoanaerobaculum_sp.]	ptg002465l	28.395	4.55E-08	55.5	84
BCW93961.1_MAG:_cation_diffusion_facilitator_transporter_[Thermoanaerobaculum_sp.]	ptg004670l	26.636	2.62E-09	62.4	64
BCW93960.1_MAG:_nitrogen_regulatory_protein_P-II_1_[Thermoanaerobaculum_sp.]	ptg003336l	38.938	1.65E-17	80.5	90
BCW93959.1_MAG:_cation_transporter_[Thermoanaerobaculum_sp.]	ptg004670l	41.908	0	654	99
BCW93958.1_MAG:_hypothetical_protein_KatS3mg007_1852_[Thermoanaerobaculum_sp.]	ptg001766l	28.261	7.71E-28	120	75
BCW93955.1_MAG:_twitching_motility_protein_PilT_[Thermoanaerobaculum_sp.]	LG13	47.429	2.39E-77	264	90
BCW93953.1_MAG:_alanine--tRNA_ligase_[Thermoanaerobaculum_sp.]	ptg002916l	50.204	0	459	96
BCW93952.1_MAG:_hypothetical_protein_KatS3mg007_1846_[Thermoanaerobaculum_sp.]	ptg005110l	38.542	1.21E-11	67.4	39
BCW93951.1_MAG:_tryptophan--tRNA_ligase_[Thermoanaerobaculum_sp.]	ptg003327l	50	5.92E-102	332	99
BCW93949.1_MAG:_acyl-CoA_dehydrogenase_[Thermoanaerobaculum_sp.]	ptg004179l	27.734	9.93E-22	101	63

BCW93947.1_MAG:_short-chain_dehydrogenase_[Thermoanaerobaculum_sp.]	ptg004732l	31.225	1.25E-27	114	97
BCW93946.1_MAG:_alanine_dehydrogenase_[Thermoanaerobaculum_sp.]	ptg003980l	38.275	1.98E-49	182	99
BCW93945.1_MAG:_4-hydroxybutyrate_CoA-transferase_[Thermoanaerobaculum_sp.]	LG22	68.627	5.11E-12	72	24
BCW93940.1_MAG:_IS256_family_transposase_[Thermoanaerobaculum_sp.]	ptg004058l	25.714	5.36E-08	58.9	68
BCW93939.1_MAG:_hypothetical_protein_KatS3mg007_1833_[Thermoanaerobaculum_sp.]	ptg002381l	27.193	8.52E-15	67.8	46
BCW93937.1_MAG:_hypothetical_protein_KatS3mg007_1831_[Thermoanaerobaculum_sp.]	ptg002381l	36.441	2.96E-12	71.2	39
BCW93935.1_MAG:_hypothetical_protein_KatS3mg007_1829_[Thermoanaerobaculum_sp.]	ptg002381l	40	1.44E-16	85.9	29
BCW93933.1_MAG:_gamma-glutamyltransferase_[Thermoanaerobaculum_sp.]	ptg001595l	56.08	0	587	97
BCW93932.1_MAG:_hypothetical_protein_KatS3mg007_1826_[Thermoanaerobaculum_sp.]	ptg005484l	28.777	1.28E-08	63.2	15
BCW93931.1_MAG:_hypothetical_protein_KatS3mg007_1825_[Thermoanaerobaculum_sp.]	ptg003839l	37.405	9.37E-15	73.2	87
BCW93930.1_MAG:_phosphate_starvation_protein_PhoH_[Thermoanaerobaculum_sp.]	ptg005772l	46.689	5.63E-78	262	95
BCW93929.1_MAG:_HD_family_phosphohydrolase_[Thermoanaerobaculum_sp.]	ptg004524l	44.444	5.47E-56	212	40
BCW93926.1_MAG:_hypothetical_protein_KatS3mg007_1820_[Thermoanaerobaculum_sp.]	ptg002760l	39	8.33E-27	117	47
BCW93925.1_MAG:_GTPase_Era_[Thermoanaerobaculum_sp.]	ptg002902l	38.854	1.31E-22	101	51
BCW93920.1_MAG:_UPF0102_protein_[Thermoanaerobaculum_sp.]	ptg002907l	36.752	1.71E-12	66.6	85
BCW93919.1_MAG:_alcohol_dehydrogenase_[Thermoanaerobaculum_sp.]	ptg000693l	35.602	1.17E-12	72.8	56
BCW93917.1_MAG:_acetyl-CoA_acetyltransferase_[Thermoanaerobaculum_sp.]	ptg005527l	44.73	1.71E-88	295	99
BCW93916.1_MAG:_putative_3-hydroxyacyl-CoA_dehydrogenase_[Thermoanaerobaculum_sp.]	ptg002760l	28.115	9.96E-23	108	38
BCW93914.1_MAG:_cysteine_desulfurase_[Thermoanaerobaculum_sp.]	ptg004917l	26.25	5.04E-09	62	61
BCW93913.1_MAG:_amidotransferase_1,_exosortase_A_system-associated_[Thermoanaerobaculum_sp.]	ptg004111l	29.567	3.24E-63	230	99
BCW93909.1_MAG:_glucose-1-phosphate_thymidylyltransferase_[Thermoanaerobaculum_sp.]	ptg004372l	34.591	1.41E-20	93.6	61
BCW93908.1_MAG:_hypothetical_protein_KatS3mg007_1802_[Thermoanaerobaculum_sp.]	ptg004476l	29.032	1.23E-13	77	58
BCW93906.1_MAG:_glutaredoxin_[Thermoanaerobaculum_sp.]	ptg004735l	27.049	3.91E-12	68.6	57
BCW93904.1_MAG:_enoyl-CoA_hydratase_[Thermoanaerobaculum_sp.]	ptg002612l	45.736	5.39E-51	182	97
BCW93903.1_MAG:_homoaconitate_hydratase_[Thermoanaerobaculum_sp.]	ptg002871l	26.768	9.32E-18	89.7	94
BCW93902.1_MAG:_hypothetical_protein_KatS3mg007_1796_[Thermoanaerobaculum_sp.]	ptg006032l	34.842	7.22E-26	119	27
BCW93900.1_MAG:_succinate--CoA_ligase_[ADP-forming]_subunit_beta_[Thermoanaerobaculum_sp.]	ptg002316l	52.685	6.49E-124	397	99
BCW93899.1_MAG:_succinate--CoA_ligase_[ADP-forming]_subunit_alpha_[Thermoanaerobaculum_sp.]	ptg002316l	59.794	3.85E-98	319	100
BCW93898.1_MAG:_nucleoside_diphosphate_kinase_[Thermoanaerobaculum_sp.]	ptg002916l	51.799	1.89E-42	152	99
BCW93895.1_MAG:_hypothetical_protein_KatS3mg007_1789_[Thermoanaerobaculum_sp.]	ptg003673l	38.125	5.15E-18	94	17
BCW93894.1_MAG:_short-chain_dehydrogenase/reductase_[Thermoanaerobaculum_sp.]	ptg005692l	35.361	2.14E-21	97.1	91
BCW93879.1_MAG:_glycosyl_transferase_[Thermoanaerobaculum_sp.]	ptg004788l	41.176	7.99E-42	155	93
BCW93878.1_MAG:_gamma-glutamyl-gamma-aminobutyrate_hydrolase_[Thermoanaerobaculum_sp.]	LG10	32.843	3E-21	95.5	84
BCW93876.1_MAG:_GTPase_Der_[Thermoanaerobaculum_sp.]	ptg003703l	41.014	2.07E-81	277	99
BCW93874.1_MAG:_transcriptional_regulator_[Thermoanaerobaculum_sp.]	ptg001925l	50.649	1.72E-19	86.3	61
BCW93871.1_MAG:_pyruvate,_phosphate_dikinase_[Thermoanaerobaculum_sp.]	ptg005844l	54.576	0	950	98

BCW93869.1_MAG:_protein_TolB_[Thermoanaerobaculum_sp.]	ptg001604l	27.976	3.98E-15	82	54
BCW93867.1_MAG:_protein_TolR_[Thermoanaerobaculum_sp.]	ptg005341l	35.115	1.41E-16	78.6	91
BCW93866.1_MAG:_Tol-Pal_system_subunit_TolQ_[Thermoanaerobaculum_sp.]	ptg005341l	34.94	6.39E-20	91.3	74
BCW93864.1_MAG:_hypothetical_protein_KatS3mg007_1758_[Thermoanaerobaculum_sp.]	ptg004854l	28.177	6.41E-09	59.3	77
BCW93861.1_MAG:_hypothetical_protein_KatS3mg007_1755_[Thermoanaerobaculum_sp.]	ptg004854l	27.273	3.24E-13	77	57
BCW93858.1_MAG:_hypothetical_protein_KatS3mg007_1752_[Thermoanaerobaculum_sp.]	ptg004854l	31.416	6.02E-18	89.4	62
BCW93856.1_MAG:_hypothetical_protein_KatS3mg007_1750_[Thermoanaerobaculum_sp.]	ptg004854l	30.677	1.01E-18	92.4	63
BCW93854.1_MAG:_hypothetical_protein_KatS3mg007_1748_[Thermoanaerobaculum_sp.]	ptg004854l	25.287	2.02E-14	80.9	58
BCW93852.1_MAG:_hypothetical_protein_KatS3mg007_1746_[Thermoanaerobaculum_sp.]	ptg004854l	25.183	5.76E-24	115	34
BCW93850.1_MAG:_hypothetical_protein_KatS3mg007_1744_[Thermoanaerobaculum_sp.]	ptg004256l	34.783	2.8E-10	64.7	33
BCW93848.1_MAG:_hypoxanthine_phosphoribosyltransferase_[Thermoanaerobaculum_sp.]	ptg003290l	40.881	7.43E-25	104	82
BCW93847.1_MAG:_reactive_intermediate/imine_deaminase_[Thermoanaerobaculum_sp.]	ptg004297l	29.323	1.56E-08	55.1	94
BCW93845.1_MAG:_hypothetical_protein_KatS3mg007_1739_[Thermoanaerobaculum_sp.]	ptg004384l	27.5	2.55E-23	103	92
BCW93844.1_MAG:_hypothetical_protein_KatS3mg007_1738_[Thermoanaerobaculum_sp.]	ptg004790l	30.258	1.25E-26	118	52
BCW93843.1_MAG:_hypothetical_protein_KatS3mg007_1737_[Thermoanaerobaculum_sp.]	ptg004479l	52.852	1.92E-79	266	84
BCW93842.1_MAG:_hypothetical_protein_KatS3mg007_1736_[Thermoanaerobaculum_sp.]	ptg004384l	29.323	0.00000515	52.8	31
BCW93841.1_MAG:_coenzyme_A_pyrophosphatase_[Thermoanaerobaculum_sp.]	ptg005213l	41.176	7.35E-19	87	61
BCW93839.1_MAG:_hypothetical_protein_KatS3mg007_1733_[Thermoanaerobaculum_sp.]	ptg003082l	33.333	3.39E-45	174	58
BCW93837.1_MAG:_redox-sensing_transcriptional_repressor_Rex_[Thermoanaerobaculum_sp.]	ptg005030l	40.5	2.43E-35	135	90
BCW93836.1_MAG:_NADH-quinone_oxidoreductase_subunit_N_[Thermoanaerobaculum_sp.]	ptg004143l	33.14	4.66E-26	116	70
BCW93835.1_MAG:_NADH:ubiquinone_oxidoreductase_subunit_M_[Thermoanaerobaculum_sp.]	ptg004143l	47.63	5.71E-111	365	89
BCW93834.1_MAG:_NADH-quinone_oxidoreductase_subunit_L_[Thermoanaerobaculum_sp.]	ptg005338l	42.655	1.91E-95	326	84
BCW93831.1_MAG:_NADH-quinone_oxidoreductase_subunit_H_[Thermoanaerobaculum_sp.]	ptg004143l	39.941	1.58E-54	196	97
BCW93830.1_MAG:_NADH_dehydrogenase_[Thermoanaerobaculum_sp.]	ptg005338l	42.742	1.42E-26	99	33
BCW93829.1_MAG:_NADH-quinone_oxidoreductase_subunit_F_2_[Thermoanaerobaculum_sp.]	ptg005451l	45.052	4.2E-95	317	88
BCW93828.1_MAG:_NADH-quinone_oxidoreductase_subunit_E_[Thermoanaerobaculum_sp.]	ptg004740l	36.585	0.000000238	53.1	47
BCW93827.1_MAG:_NADH-quinone_oxidoreductase_subunit_A_[Thermoanaerobaculum_sp.]	ptg005338l	37.815	1.32E-18	84	92
BCW93825.1_MAG:_nucleoside_triphosphate_pyrophosphohydrolase_[Thermoanaerobaculum_sp.]	ptg002030l	38.545	3.73E-46	168	97
BCW93823.1_MAG:_peptidylprolyl_isomerase_[Thermoanaerobaculum_sp.]	LG28	41.27	1.58E-08	60.1	19
BCW93822.1_MAG:_hypothetical_protein_KatS3mg007_1716_[Thermoanaerobaculum_sp.]	ptg005005l	33.708	0.0000021	47.4	95
BCW93821.1_MAG:_hydrogenase_[Thermoanaerobaculum_sp.]	ptg004143l	42.857	3.14E-19	90.5	46
BCW93820.1_MAG:_hydrogenase_[Thermoanaerobaculum_sp.]	ptg004143l	24.74	4.77E-15	82.4	71

BCW93819.1_MAG:_hydrogenase_[Thermoanaerobaculum_sp.]	ptg003918l	30.282	3.56E-20	98.2	72
BCW93816.1_MAG:_hydrogenase_[Thermoanaerobaculum_sp.]	ptg004143l	28.614	4.91E-16	86.7	50
BCW93815.1_MAG:_MerR_family_transcriptional_regulator_[Thermoanaerobaculum_sp.]	ptg005709l	39.189	3.21E-09	60.1	32
BCW93814.1_MAG:_cold-shock_protein_[Thermoanaerobaculum_sp.]	ptg003498l	71.875	3.82E-23	93.6	94
BCW93813.1_MAG:_carbonic_anhydrase_[Thermoanaerobaculum_sp.]	ptg003910l	30.208	1.31E-23	101	94
BCW93812.1_MAG:_asparagine--tRNA_ligase_[Thermoanaerobaculum_sp.]	ptg002477l	32.662	1.48E-74	257	96
BCW93811.1_MAG:_hypothetical_protein_KatS3mg007_1705_[Thermoanaerobaculum_sp.]	ptg003431l	38.144	5.96E-10	66.6	16
BCW93809.1_MAG:_ribosomal_protein_S12_methylthiotransferase_RimO_[Thermoanaerobaculum_sp.]	ptg004147l	38.391	1.72E-83	285	92
BCW93808.1_MAG:_riboflavin_biosynthesis_protein_[Thermoanaerobaculum_sp.]	ptg002304l	35.374	3.81E-32	130	92
BCW93806.1_MAG:_S-adenosylmethionine_synthase_[Thermoanaerobaculum_sp.]	ptg005611l	53.731	1.23E-129	415	97
BCW93801.1_MAG:_hypothetical_protein_KatS3mg007_1695_[Thermoanaerobaculum_sp.]	ptg004510l	30.841	0.000000923	54.7	32
BCW93800.1_MAG:_aminomethyltransferase_[Thermoanaerobaculum_sp.]	ptg005299l	37.741	4.38E-78	265	98
BCW93799.1_MAG:_glycine_cleavage_system_H_protein_[Thermoanaerobaculum_sp.]	ptg005318l	47.581	1.21E-31	120	95
BCW93798.1_MAG:_putative_glycine_dehydrogenase_(decarboxylating)_subunit_1_[Thermoanaerobaculum_sp.]	ptg005299l	36.915	6.13E-52	192	80
BCW93797.1_MAG:_putative_glycine_dehydrogenase_(decarboxylating)_subunit_2_[Thermoanaerobaculum_sp.]	ptg005318l	43.103	1.29E-86	295	81
BCW93796.1_MAG:_6-carboxytetrahydropterin_synthase_QueD_[Thermoanaerobaculum_sp.]	ptg005533l	31.579	7E-14	71.2	97
BCW93794.1_MAG:_2-amino-3-ketobutyrate_CoA_ligase_[Thermoanaerobaculum_sp.]	ptg001224l	35.969	3E-68	238	92
BCW93793.1_MAG:_hypothetical_protein_KatS3mg007_1687_[Thermoanaerobaculum_sp.]	ptg004662l	38.933	7.22E-78	265	99
BCW93792.1_MAG:_dehydrogenase_[Thermoanaerobaculum_sp.]	ptg003910l	47.031	8.29E-110	361	88
BCW93791.1_MAG:_hypothetical_protein_KatS3mg007_1685_[Thermoanaerobaculum_sp.]	ptg004214l	27.9	7.11E-25	108	98
BCW93789.1_MAG:_cytidylate_kinase_[Thermoanaerobaculum_sp.]	ptg002942l	42.929	6.64E-41	152	82
BCW93788.1_MAG:_30S_ribosomal_protein_S1_[Thermoanaerobaculum_sp.]	ptg002742l	43.046	2.99E-103	347	76
BCW93787.1_MAG:_integration_host_factor_subunit_beta_[Thermoanaerobaculum_sp.]	ptg003072l	46.667	1.53E-13	67.8	99
BCW93786.1_MAG:_hydrolase_[Thermoanaerobaculum_sp.]	ptg004051l	31.618	2.87E-09	58.5	80
BCW93783.1_MAG:_lipid-A-disaccharide_synthase_[Thermoanaerobaculum_sp.]	ptg002436l	39.264	4.02E-44	112	82
BCW93782.1_MAG:_ABC_transporter_ATP-binding_protein_[Thermoanaerobaculum_sp.]	ptg004774l	39.606	2.35E-84	292	74
BCW93781.1_MAG:_hypothetical_protein_KatS3mg007_1675_[Thermoanaerobaculum_sp.]	ptg005107l	51.316	3.94E-16	82.4	26
BCW93780.1_MAG:_hypothetical_protein_KatS3mg007_1674_[Thermoanaerobaculum_sp.]	ptg004670l	28.525	1.37E-46	114	72
BCW93779.1_MAG:_cytochrome_c_[Thermoanaerobaculum_sp.]	ptg002453l	50.242	2.82E-61	209	89
BCW93778.1_MAG:_molybdopterin_oxidoreductase_[Thermoanaerobaculum_sp.]	ptg001690l	55.6	3.52E-87	311	90
BCW93777.1_MAG:_molybdopterin_oxidoreductase_membrane_subunit_[Thermoanaerobaculum_sp.]	ptg002453l	64.967	8.88E-174	546	92
BCW93776.1_MAG:_hypothetical_protein_KatS3mg007_1670_[Thermoanaerobaculum_sp.]	ptg001690l	38.624	4.64E-31	122	94
BCW93775.1_MAG:_quinol:cytochrome_C_oxidoreductase_[Thermoanaerobaculum_sp.]	ptg001690l	40.553	1.77E-36	139	75
BCW93774.1_MAG:_membrane_protein_[Thermoanaerobaculum_sp.]	ptg001690l	41.045	1.2E-75	259	93
BCW93772.1_MAG:_photosynthetic_protein_synthase_I_[Thermoanaerobaculum_sp.]	ptg002453l	33.032	6.69E-37	142	81

BCW93771.1_MAG:_cytochrome_c_oxidase_subunit_2_[Thermoanaerobaculum_sp.]	ptg002453l	41.987	3.53E-61	215	90
BCW93770.1_MAG:_cytochrome_c_oxidase_subunit_I_[Thermoanaerobaculum_sp.]	ptg001690l	65.038	0	632	96
BCW93769.1_MAG:_cytochrome_c_oxidase_subunit_III_[Thermoanaerobaculum_sp.]	ptg002453l	53.968	3.09E-38	145	77
BCW93768.1_MAG:_hypothetical_protein_KatS3mg007_1662_[Thermoanaerobaculum_sp.]	ptg001690l	36.792	8.9E-10	58.2	91
BCW93766.1_MAG:_RNA_pseudouridine_synthase_[Thermoanaerobaculum_sp.]	ptg004470l	35.542	1.36E-15	79	69
BCW93763.1_MAG:_pteridine_reductase_[Thermoanaerobaculum_sp.]	ptg005905l	32.5	7.77E-21	94.7	95
BCW93762.1_MAG:_hypothetical_protein_KatS3mg007_1656_[Thermoanaerobaculum_sp.]	ptg003785l	34.615	2.67E-15	75.9	92
BCW93758.1_MAG:_segregation_and_condensation_protein_A_[Thermoanaerobaculum_sp.]	ptg005938l	33.333	8.56E-13	71.2	39
BCW93757.1_MAG:_segregation_and_condensation_protein_B_[Thermoanaerobaculum_sp.]	ptg004457l	41.667	5.74E-20	90.1	53
BCW93755.1_MAG:_histidinol-phosphate_aminotransferase_[Thermoanaerobaculum_sp.]	ptg002505l	27.687	5.7E-25	110	81
BCW93751.1_MAG:_cation_transporter_[Thermoanaerobaculum_sp.]	ptg004670l	29.455	3.78E-27	115	91
BCW93748.1_MAG:_phosphoglycolate_phosphatase,_bacterial_[Thermoanaerobaculum_sp.]	ptg004584l	40.816	0.00000377	36.2	92
BCW93746.1_MAG:_D-alanine--D-alanine_ligase_[Thermoanaerobaculum_sp.]	ptg004510l	34.007	7.16E-46	171	78
BCW93745.1_MAG:_bifunctional_malic_enzyme_oxidoreductase/phosphotransacetylase_[Thermoanaerobaculum_sp.]	ptg005092l	44.504	0	608	98
BCW93742.1_MAG:_GTP_cyclohydrolase_1_[Thermoanaerobaculum_sp.]	ptg002479l	48.315	1.78E-48	172	84
BCW93741.1_MAG:_glucose-1-phosphate_thymidylyltransferase_[Thermoanaerobaculum_sp.]	ptg004111l	39.496	6.52E-45	168	66
BCW93740.1_MAG:_dTDP-glucose_4,6-dehydratase_[Thermoanaerobaculum_sp.]	ptg001690l	44.476	7.34E-90	297	96
BCW93739.1_MAG:_NAD(P)-dependent_oxidoreductase_[Thermoanaerobaculum_sp.]	ptg003827l	38.163	1.15E-42	159	96
BCW93738.1_MAG:_peptidase_M28_[Thermoanaerobaculum_sp.]	ptg001897l	38.889	0.000000422	57	13
BCW93735.1_MAG:_ABC_transporter_ATP-binding_protein_[Thermoanaerobaculum_sp.]	ptg004579l	42.009	1.31E-47	174	69
BCW93733.1_MAG:_ABC_transporter_ATP-binding_protein_[Thermoanaerobaculum_sp.]	ptg004026l	32.103	1.15E-34	137	84
BCW93732.1_MAG:_hemolysin_secretion_protein_D_[Thermoanaerobaculum_sp.]	ptg004663l	33.036	1.76E-10	65.9	35
BCW93731.1_MAG:_hypothetical_protein_KatS3mg007_1625_[Thermoanaerobaculum_sp.]	ptg004125l	34.234	0.0000098	49.3	51
BCW93729.1_MAG:_hypothetical_protein_KatS3mg007_1623_[Thermoanaerobaculum_sp.]	LG02	46.512	5.13E-08	60.1	15
BCW93728.1_MAG:_pyruvate_ferredoxin_oxidoreductase_[Thermoanaerobaculum_sp.]	ptg002867l	25.281	3.58E-09	62.8	79
BCW93723.1_MAG:_hypothetical_protein_KatS3mg007_1617_[Thermoanaerobaculum_sp.]	ptg005554l	45.806	7.94E-64	222	93
BCW93722.1_MAG:_L-methionine_gamma-lyase_[Thermoanaerobaculum_sp.]	ptg004895l	47.581	9.53E-104	340	95
BCW93717.1_MAG:_multidrug_resistance_protein_A,_efflux_pump_[Thermoanaerobaculum_sp.]	ptg002902l	36.986	0.00000879	51.6	19
BCW93716.1_MAG:_MFS_transporter_[Thermoanaerobaculum_sp.]	ptg002902l	29.146	4.67E-24	75.5	55
BCW93710.1_MAG:_hemolysin_D_[Thermoanaerobaculum_sp.]	ptg004790l	42.69	7.96E-15	77	69
BCW93706.1_MAG:_6,7-dimethyl-8-ribityllumazine_synthase_[Thermoanaerobaculum_sp.]	ptg004564l	41.727	5.27E-29	114	90
BCW93705.1_MAG:_N_utilization_substance_protein_B_[Thermoanaerobaculum_sp.]	ptg004564l	46.281	1.07E-24	101	86
BCW93704.1_MAG:_hypothetical_protein_KatS3mg007_1598_[Thermoanaerobaculum_sp.]	LG03	36.364	0.00000374	51.6	35
BCW93702.1_MAG:_hypothetical_protein_KatS3mg007_1596_[Thermoanaerobaculum_sp.]	ptg003734l	35.61	5.09E-31	127	63
BCW93701.1_MAG:_protoheme_IX_farnesyltransferase_[Thermoanaerobaculum_sp.]	LG06	50	1.55E-38	149	60

BCW93700.1_MAG:_hypothetical_protein_KatS3mg007_1594_[Thermoanaerobaculum_sp.]	ptg004250l	45.714	0.000000097	58.5	16
BCW93699.1_MAG:_7-carboxy-7-deazaguanine_synthase_[Thermoanaerobaculum_sp.]	ptg003839l	41.732	1.87E-54	191	100
BCW93695.1_MAG:_peptidase_M23_[Thermoanaerobaculum_sp.]	ptg005526l	35.976	1.83E-20	97.4	42
BCW93694.1_MAG:_hypothetical_protein_KatS3mg007_1588_[Thermoanaerobaculum_sp.]	ptg002316l	22.36	5.94E-08	58.9	79
BCW93693.1_MAG:_hypothetical_protein_KatS3mg007_1587_[Thermoanaerobaculum_sp.]	ptg002534l	35.945	6.68E-20	95.9	56
BCW93691.1_MAG:_lipid_A_ABC_transporter_permease/ATP-binding_protein_[Thermoanaerobaculum_sp.]	ptg004620l	32.724	1.86E-68	246	81
BCW93690.1_MAG:_hydroxylase_[Thermoanaerobaculum_sp.]	ptg004564l	34.14	2.72E-59	213	84
BCW93688.1_MAG:_hypothetical_protein_KatS3mg007_1582_[Thermoanaerobaculum_sp.]	ptg005903l	29.183	4.67E-12	72.8	50
BCW93687.1_MAG:_hypothetical_protein_KatS3mg007_1581_[Thermoanaerobaculum_sp.]	ptg005756l	34.906	1.07E-14	72.8	78
BCW93684.1_MAG:_putative_lipid_II_flippase_MurJ_[Thermoanaerobaculum_sp.]	ptg002304l	33.663	2.99E-25	114	58
BCW93683.1_MAG:_D-aminoacyl-tRNA_deacylase_[Thermoanaerobaculum_sp.]	ptg004026l	53.425	1.9E-35	132	97
BCW93682.1_MAG:_hypothetical_protein_KatS3mg007_1576_[Thermoanaerobaculum_sp.]	ptg001245l	33.468	1.02E-26	114	74
BCW93681.1_MAG:_DNA-binding_protein_HU_[Thermoanaerobaculum_sp.]	ptg005527l	44.706	9.08E-18	79.7	96
BCW93680.1_MAG:_hypothetical_protein_KatS3mg007_1574_[Thermoanaerobaculum_sp.]	LG13	30.233	6.58E-11	68.2	34
BCW93679.1_MAG:_ribosomal-protein-alanine_acetyltransferase_[Thermoanaerobaculum_sp.]	ptg003827l	34.615	2.81E-09	58.5	64
BCW93678.1_MAG:_hypothetical_protein_KatS3mg007_1572_[Thermoanaerobaculum_sp.]	ptg003708l	45.714	8.18E-08	55.5	34
BCW93677.1_MAG:_fumarate_hydratase_class_II_[Thermoanaerobaculum_sp.]	ptg005906l	41.928	3.06E-86	292	90
BCW93676.1_MAG:_hypothetical_protein_KatS3mg007_1570_[Thermoanaerobaculum_sp.]	ptg005375l	34.834	4.4E-171	562	90
BCW93675.1_MAG:_type_I_citrate_synthase_[Thermoanaerobaculum_sp.]	ptg005518l	28.883	2.36E-29	125	89
BCW93673.1_MAG:_hypothetical_protein_KatS3mg007_1567_[Thermoanaerobaculum_sp.]	ptg005484l	25.253	3.89E-19	97.4	45
BCW93672.1_MAG:_hypothetical_protein_KatS3mg007_1566_[Thermoanaerobaculum_sp.]	ptg004179l	30.508	0.000000058	53.1	90
BCW93671.1_MAG:_poly(A)_polymerase_[Thermoanaerobaculum_sp.]	ptg004623l	32.377	4.32E-14	80.9	25
BCW93670.1_MAG:_methyltransferase_small_[Thermoanaerobaculum_sp.]	ptg004885l	30.814	0.000000283	53.5	83
BCW93669.1_MAG:_twitching_motility_protein_PilT_[Thermoanaerobaculum_sp.]	ptg004468l	32.016	6.89E-36	143	67
BCW93667.1_MAG:_ATPase_[Thermoanaerobaculum_sp.]	ptg003999l	49.359	1.5E-89	295	98
BCW93665.1_MAG:_hypothetical_protein_KatS3mg007_1559_[Thermoanaerobaculum_sp.]	ptg004759l	37.594	2.21E-14	81.3	17
BCW93662.1_MAG:_membrane_protein_[Thermoanaerobaculum_sp.]	ptg002123l	30.4	9.89E-12	68.6	48
BCW93660.1_MAG:_lipoyl_synthase_[Thermoanaerobaculum_sp.]	ptg003871l	50.896	1.6E-81	271	94
BCW93659.1_MAG:_deoxyhypusine_synthase_[Thermoanaerobaculum_sp.]	ptg005865l	57.812	5.13E-110	355	98
BCW93658.1_MAG:_hypothetical_protein_KatS3mg007_1552_[Thermoanaerobaculum_sp.]	ptg005876l	33.716	1.63E-64	238	58
BCW93657.1_MAG:_4-hydroxy-3-methylbut-2-en-1-yl_diphosphate_synthase_(flavodoxin)_[Thermoanaerobaculum_sp.]	ptg003703l	36.829	7E-54	197	98
BCW93656.1_MAG:_cytidine_deaminase_[Thermoanaerobaculum_sp.]	ptg005856l	42.623	8.17E-09	56.2	43
BCW93655.1_MAG:_6-phosphofructokinase_[Thermoanaerobaculum_sp.]	ptg003223l	48.78	2.78E-96	317	97
BCW93654.1_MAG:_hypothetical_protein_KatS3mg007_1548_[Thermoanaerobaculum_sp.]	ptg004470l	29.714	0.00000102	52.4	84
BCW93652.1_MAG:_prolyl_endopeptidase_[Thermoanaerobaculum_sp.]	ptg003431l	53.148	0	759	96
BCW93651.1_MAG:_3-hydroxybutyryl-CoA_dehydrogenase_[Thermoanaerobaculum_sp.]	ptg002612l	48.571	1.43E-73	248	99

BCW93649.1_MAG:_proline--tRNA_ligase_[Thermoanaerobaculum_sp.]	ptg005656l	59.091	5.13E-51	165	57
BCW93646.1_MAG:_glucokinase_[Thermoanaerobaculum_sp.]	ptg001925l	25.914	2.1E-22	102	89
BCW93643.1_MAG:_cation-efflux_pump_FieF_[Thermoanaerobaculum_sp.]	ptg005301l	29.289	5.73E-27	114	82
BCW93641.1_MAG:_O-acetylhomoserine_aminocarboxypropyltransferase_[Thermoanaerobaculum_sp.]	ptg004895l	31.264	8.42E-50	186	94
BCW93639.1_MAG:_hypothetical_protein_KatS3mg007_1533_[Thermoanaerobaculum_sp.]	LG02	36.19	5.58E-20	100	70
BCW93631.1_MAG:_hypothetical_protein_KatS3mg007_1525_[Thermoanaerobaculum_sp.]	ptg002313l	32.624	1.61E-11	64.7	89
BCW93630.1_MAG:_hypothetical_protein_KatS3mg007_1524_[Thermoanaerobaculum_sp.]	ptg005110l	40.333	5.07E-58	211	78
BCW93629.1_MAG:_dihydrolipoyl_dehydrogenase_[Thermoanaerobaculum_sp.]	ptg003780l	41.667	7.6E-95	317	99
BCW93628.1_MAG:_hypothetical_protein_KatS3mg007_1522_[Thermoanaerobaculum_sp.]	ptg003365l	40.523	2.84E-32	124	96
BCW93627.1_MAG:_hypothetical_protein_KatS3mg007_1521_[Thermoanaerobaculum_sp.]	ptg005076l	23.883	3.99E-24	112	93
BCW93626.1_MAG:_succinate_dehydrogenase_[Thermoanaerobaculum_sp.]	ptg003304l	66.284	3.28E-117	372	99
BCW93625.1_MAG:_succinate_dehydrogenase_flavoprotein_subunit_[Thermoanaerobaculum_sp.]	ptg002316l	63.108	0	769	100
BCW93624.1_MAG:_succinate_dehydrogenase_[Thermoanaerobaculum_sp.]	ptg003304l	39.381	3.4E-38	144	98
BCW93623.1_MAG:_putative_thiol_peroxidase_[Thermoanaerobaculum_sp.]	ptg001693l	39.375	6.38E-30	118	90
BCW93621.1_MAG:_hypothetical_protein_KatS3mg007_1515_[Thermoanaerobaculum_sp.]	ptg003471l	40.659	2.82E-10	64.3	35
BCW93620.1_MAG:_peptidase_S9_[Thermoanaerobaculum_sp.]	ptg004136l	32.695	4.05E-108	364	95
BCW93618.1_MAG:_peptidyl-prolyl_cis-trans_isomerase_[Thermoanaerobaculum_sp.]	LG03	43.137	3.54E-33	126	99
BCW93609.1_MAG:_hypothetical_protein_KatS3mg007_1503_[Thermoanaerobaculum_sp.]	ptg005754l	24.224	4.23E-10	67.4	50
BCW93606.1_MAG:_CRISPR-associated_endonuclease_Cas1_[Thermoanaerobaculum_sp.]	ptg004111l	30.034	3.83E-27	116	82
BCW93602.1_MAG:_hypothetical_protein_KatS3mg007_1496_[Thermoanaerobaculum_sp.]	ptg004510l	41.322	2.4E-20	95.9	37
BCW93600.1_MAG:_DNA_mismatch_repair_protein_MutS_[Thermoanaerobaculum_sp.]	ptg001460l	39.955	1.09E-163	533	96
BCW93599.1_MAG:_beta-hexosaminidase_[Thermoanaerobaculum_sp.]	ptg005474l	29.851	5.79E-29	121	94
BCW93598.1_MAG:_7-cyano-7-deazaguanine_synthase_[Thermoanaerobaculum_sp.]	ptg004480l	34.197	4.47E-21	94.7	83
BCW93597.1_MAG:_hypothetical_protein_KatS3mg007_1491_[Thermoanaerobaculum_sp.]	ptg003199l	43.988	3.16E-63	228	98
BCW93595.1_MAG:_nucleoside_transporter_[Thermoanaerobaculum_sp.]	ptg001925l	44.414	8.92E-75	257	88
BCW93593.1_MAG:_spore_coat_polysaccharide_biosynthesis_protein_SpsL_[Thermoanaerobaculum_sp.]	ptg003827l	26.829	0.00000545	49.7	87
BCW93592.1_MAG:_2-dehydro-3-deoxyphosphooctonate_aldolase_[Thermoanaerobaculum_sp.]	ptg003247l	48.485	2.79E-75	253	94
BCW93591.1_MAG:_CTP_synthase_[Thermoanaerobaculum_sp.]	ptg004256l	55.882	0	613	96
BCW93590.1_MAG:_3-deoxy-manno-octulosonate_cytidylyltransferase_[Thermoanaerobaculum_sp.]	ptg002316l	37.073	4.38E-32	127	75
BCW93587.1_MAG:_octaprenyl-diphosphate_synthase_[Thermoanaerobaculum_sp.]	ptg005486l	38.507	7.72E-53	191	98
BCW93585.1_MAG:_bifunctional_purine_biosynthesis_protein_PurH_[Thermoanaerobaculum_sp.]	ptg000492l	43.16	1.63E-114	376	99
BCW93583.1_MAG:_macrodomain_protein_[Thermoanaerobaculum_sp.]	ptg003471l	39.316	5.27E-14	72.8	60
BCW93582.1_MAG:_multidrug_ABC_transporter_substrate-binding_protein_[Thermoanaerobaculum_sp.]	ptg004986l	32.962	1E-32	134	98
BCW93581.1_MAG:_RND_transporter_[Thermoanaerobaculum_sp.]	ptg005906l	25.862	0.00000421	53.1	55

BCW93576.1_MAG:_glycosyl_transferase_[Thermoanaerobaculum_sp.]	ptg004680l	33.99	3.57E-18	89	62
BCW93573.1_MAG:_hypothetical_protein_KatS3mg007_1467_[Thermoanaerobaculum_sp.]	ptg002612l	44.944	1.14E-10	60.8	75
BCW93571.1_MAG:_peptidase_M28_[Thermoanaerobaculum_sp.]	ptg001897l	44.286	5.96E-08	59.3	15
BCW93570.1_MAG:_hypothetical_protein_KatS3mg007_1464_[Thermoanaerobaculum_sp.]	LG22	47.727	0.00000161	47.4	52
BCW93568.1_MAG:_cysteine--tRNA_ligase_[Thermoanaerobaculum_sp.]	ptg002844l	40.385	1.76E-92	310	98
BCW93566.1_MAG:_dienelactone_hydrolase_[Thermoanaerobaculum_sp.]	LG27	36.296	1.19E-13	74.3	48
BCW93565.1_MAG:_methionine--tRNA_ligase_[Thermoanaerobaculum_sp.]	ptg002214l	25.773	4.88E-60	221	99
BCW93560.1_MAG:_hypothetical_protein_KatS3mg007_1454_[Thermoanaerobaculum_sp.]	ptg002381l	30.374	2.19E-15	79.7	56
BCW93558.1_MAG:_hypothetical_protein_KatS3mg007_1452_[Thermoanaerobaculum_sp.]	ptg002381l	30.841	9.33E-16	80.1	58
BCW93556.1_MAG:_hypothetical_protein_KatS3mg007_1450_[Thermoanaerobaculum_sp.]	ptg002381l	34.862	1.57E-08	59.7	49
BCW93554.1_MAG:_hypothetical_protein_KatS3mg007_1448_[Thermoanaerobaculum_sp.]	ptg002381l	37.037	6.92E-09	61.2	31
BCW93552.1_MAG:_acyl-CoA_dehydrogenase_[Thermoanaerobaculum_sp.]	ptg004179l	27.792	4.62E-29	124	94
BCW93551.1_MAG:_peptidase_[Thermoanaerobaculum_sp.]	ptg002175l	23.133	2.46E-17	90.9	55
BCW93549.1_MAG:_carbamoyl-phosphate_synthase_small_chain_[Thermoanaerobaculum_sp.]	ptg001693l	41.005	1.12E-66	232	99
BCW93548.1_MAG:_carbamoyl-phosphate_synthase_(glutamine-hydrolyzing)_[Thermoanaerobaculum_sp.]	ptg002303l	51.668	0	1050	97
BCW93543.1_MAG:_ribonuclease_D_[Thermoanaerobaculum_sp.]	ptg002760l	29.682	6.39E-35	140	76
BCW93541.1_MAG:_hypothetical_protein_KatS3mg007_1435_[Thermoanaerobaculum_sp.]	ptg004500l	56.733	3.18E-166	522	99
BCW93540.1_MAG:_3-oxoacyl-ACP_reductase_[Thermoanaerobaculum_sp.]	ptg005905l	38.672	7.51E-29	118	99
BCW93539.1_MAG:_hydrolase_[Thermoanaerobaculum_sp.]	ptg005709l	41.04	1.64E-17	83.2	93
BCW93538.1_MAG:_ABC_transporter_ATP-binding_protein_[Thermoanaerobaculum_sp.]	ptg005227l	32.749	2.66E-29	122	97
BCW93537.1_MAG:_ABC_transporter_ATP-binding_protein_[Thermoanaerobaculum_sp.]	ptg005227l	33.824	4.68E-41	156	77
BCW93536.1_MAG:_hypothetical_protein_KatS3mg007_1430_[Thermoanaerobaculum_sp.]	ptg004500l	28.947	4.26E-12	73.2	34
BCW93534.1_MAG:_RND_transporter_[Thermoanaerobaculum_sp.]	ptg005906l	27.803	4.08E-09	62.8	54
BCW93531.1_MAG:_macrolide_ABC_transporter_ATP-binding_protein_[Thermoanaerobaculum_sp.]	ptg001659l	58.095	3.33E-78	259	93
BCW93530.1_MAG:_ABC_transporter_permease_[Thermoanaerobaculum_sp.]	ptg004986l	31.461	2.02E-37	148	99
BCW93529.1_MAG:_multidrug_transporter_[Thermoanaerobaculum_sp.]	ptg005692l	45.63	0	660	84
BCW93528.1_MAG:_RND_transporter_[Thermoanaerobaculum_sp.]	ptg005692l	37.267	7.5E-33	134	85
BCW93526.1_MAG:_hypothetical_protein_KatS3mg007_1420_[Thermoanaerobaculum_sp.]	ptg003861l	27.803	2.04E-12	69.3	98
BCW93521.1_MAG:_aldehyde_dehydrogenase_[Thermoanaerobaculum_sp.]	ptg004942l	28.537	8.3E-44	170	80
BCW93518.1_MAG:_DNA-directed_DNA_polymerase_[Thermoanaerobaculum_sp.]	ptg003999l	36.962	0	689	99
BCW93517.1_MAG:_ornithine_aminotransferase_[Thermoanaerobaculum_sp.]	ptg002126l	36.883	9.81E-75	257	94
BCW93516.1_MAG:_hypothetical_protein_KatS3mg007_1410_[Thermoanaerobaculum_sp.]	ptg001925l	25.862	0.00000876	53.5	22
BCW93515.1_MAG:_chromosome_partitioning_protein_ParB_[Thermoanaerobaculum_sp.]	ptg001818l	42	1.24E-31	127	50
BCW93514.1_MAG:_sporulation_initiation_inhibitor_Soj_[Thermoanaerobaculum_sp.]	ptg003829l	40.323	1.71E-57	200	99
BCW93513.1_MAG:_hypothetical_protein_KatS3mg007_1407_[Thermoanaerobaculum_sp.]	ptg003247l	37.374	3.34E-17	81.3	61
BCW93512.1_MAG:_tRNA-2-methylthio-N(6)-dimethylallyladenosine_synthase_[Thermoanaerobaculum_sp.]	ptg005772l	41.553	2.09E-103	342	94

BCW93509.1_MAG:_phosphoenolpyruvate-protein_phosphotransferase_[Thermoanaerobaculum_sp.]	ptg002723l	35.805	7.72E-98	330	96
BCW93508.1_MAG:_phosphocarrier_protein_HPr_[Thermoanaerobaculum_sp.]	ptg005709l	42.222	3.41E-15	72.4	96
BCW93506.1_MAG:_nucleotide-binding_protein_[Thermoanaerobaculum_sp.]	ptg005709l	36.94	2.45E-53	190	91
BCW93504.1_MAG:_PTS_sugar_transporter_subunit_IIA_[Thermoanaerobaculum_sp.]	ptg005709l	32.812	2.31E-15	75.5	84
BCW93502.1_MAG:_RNA_polymerase_sigma-54_factor_[Thermoanaerobaculum_sp.]	ptg003787l	32.891	5.8E-55	203	73
BCW93501.1_MAG:_ABC_transporter_ATP-binding_protein_[Thermoanaerobaculum_sp.]	ptg004306l	53.182	1.11E-72	243	98
BCW93499.1_MAG:_acetyl-coenzyme_A_carboxylase_carboxyl_transferase_subunit_alpha_[Thermoanaerobaculum_sp.]	ptg002304l	52.29	5.63E-90	297	82
BCW93498.1_MAG:_hypothetical_protein_KatS3mg007_1392_[Thermoanaerobaculum_sp.]	ptg005647l	51.282	2.82E-27	113	48
BCW93496.1_MAG:_hypothetical_protein_KatS3mg007_1390_[Thermoanaerobaculum_sp.]	ptg004111l	31.474	6.76E-25	110	68
BCW93495.1_MAG:_hypothetical_protein_KatS3mg007_1389_[Thermoanaerobaculum_sp.]	ptg005297l	33.81	3.75E-72	258	78
BCW93492.1_MAG:_protein_phosphatase_[Thermoanaerobaculum_sp.]	ptg001907l	35.714	1.21E-31	126	97
BCW93491.1_MAG:_hypothetical_protein_KatS3mg007_1385_[Thermoanaerobaculum_sp.]	ptg005113l	46.479	3.9E-13	71.2	33
BCW93486.1_MAG:_hypothetical_protein_KatS3mg007_1380_[Thermoanaerobaculum_sp.]	ptg005035l	34.694	1.11E-29	128	39
BCW93485.1_MAG:_putative_phosphomannomutase_[Thermoanaerobaculum_sp.]	ptg002505l	36.364	5.36E-75	259	98
BCW93484.1_MAG:_D-amino-acid_transaminase_[Thermoanaerobaculum_sp.]	ptg005680l	26.596	4.55E-19	91.3	91
BCW93483.1_MAG:_beta-ketoacyl-ACP_reductase_[Thermoanaerobaculum_sp.]	ptg003980l	38.667	1.46E-37	144	85
BCW93482.1_MAG:_glycosyl_transferase_family_2_[Thermoanaerobaculum_sp.]	ptg003431l	30.935	1.55E-12	70.9	52
BCW93481.1_MAG:_RNA_methyltransferase_[Thermoanaerobaculum_sp.]	ptg001291l	35.211	1.01E-43	166	91
BCW93480.1_MAG:_glutamyl-tRNA_reductase_[Thermoanaerobaculum_sp.]	ptg001290l	31.013	1.68E-26	116	73
BCW93479.1_MAG:_porphobilinogen_deaminase_[Thermoanaerobaculum_sp.]	ptg001995l	43.103	3.18E-42	159	76
BCW93477.1_MAG:_delta-aminolevulinic_acid_dehydratase_[Thermoanaerobaculum_sp.]	ptg004997l	44.753	4.59E-68	234	96
BCW93476.1_MAG:_glutamate-1-semialdehyde_2,1-aminomutase_[Thermoanaerobaculum_sp.]	ptg005487l	55.556	2.95E-104	213	91
BCW93475.1_MAG:_uroporphyrinogen_decarboxylase_[Thermoanaerobaculum_sp.]	ptg005545l	38.872	5.18E-69	237	99
BCW93474.1_MAG:_oxygen-independent_coproporphyrinogen_III_oxidase_[Thermoanaerobaculum_sp.]	ptg003548l	40.043	1.54E-108	357	96
BCW93473.1_MAG:_protoporphyrinogen_oxidase_[Thermoanaerobaculum_sp.]	ptg002760l	33.045	1.25E-43	168	98
BCW93472.1_MAG:_transcriptional_repressor_[Thermoanaerobaculum_sp.]	ptg003304l	29.457	2.11E-12	67.4	81
BCW93470.1_MAG:_ATPase_AAA_[Thermoanaerobaculum_sp.]	ptg001690l	47.42	1.86E-93	312	91
BCW93469.1_MAG:_aldolase_[Thermoanaerobaculum_sp.]	ptg001878l	33.333	3.1E-20	90.9	97
BCW93466.1_MAG:_acyl-CoA_thioesterase_[Thermoanaerobaculum_sp.]	ptg004075l	36.975	9.52E-14	70.5	87
BCW93464.1_MAG:_Maf-like_protein_[Thermoanaerobaculum_sp.]	ptg005709l	41.209	2.66E-21	94.7	89
BCW93462.1_MAG:_hypothetical_protein_KatS3mg007_1356_[Thermoanaerobaculum_sp.]	ptg005680l	46.602	3.6E-09	58.2	63
BCW93459.1_MAG:_cation_efflux_system_protein_[Thermoanaerobaculum_sp.]	ptg001766l	29.412	1.02E-46	178	84
BCW93458.1_MAG:_cation_transporter_[Thermoanaerobaculum_sp.]	ptg004670l	32.826	4.28E-174	571	99
BCW93457.1_MAG:_copper-translocating_P-type_ATPase_[Thermoanaerobaculum_sp.]	ptg003880l	37.099	2.27E-75	271	76
BCW93455.1_MAG:_hypothetical_protein_KatS3mg007_1349_[Thermoanaerobaculum_sp.]	ptg003220l	32.979	8.39E-09	56.6	64

BCW93446.1_MAG:_riboflavin_biosynthesis_protein_RibBA_[Thermoanaerobaculum_sp.]	ptg003075l	53.351	3.61E-122	393	94
BCW93444.1_MAG:_outer_membrane_protein_assembly_factor_BamA_[Thermoanaerobaculum_sp.]	ptg005656l	23.415	3.59E-16	87	81
BCW93441.1_MAG:_NAD_kinase_[Thermoanaerobaculum_sp.]	ptg002867l	34.199	6.09E-35	137	78
BCW93440.1_MAG:_hypothetical_protein_KatS3mg007_1334_[Thermoanaerobaculum_sp.]	ptg003753l	46.154	7.35E-08	49.7	98
BCW93437.1_MAG:_1-acyl-sn-glycerol-3-phosphate_acyltransferase_[Thermoanaerobaculum_sp.]	ptg003708l	36.975	1.44E-18	89.4	42
BCW93436.1_MAG:_RNA-splicing_ligase_RtcB_[Thermoanaerobaculum_sp.]	ptg003039l	30.562	1.59E-29	126	91
BCW93435.1_MAG:_hypothetical_protein_KatS3mg007_1329_[Thermoanaerobaculum_sp.]	ptg001995l	25.253	1.72E-17	92.8	40
BCW93433.1_MAG:_sulfate_adenylyltransferase_[Thermoanaerobaculum_sp.]	ptg005689l	64.935	7.99E-141	327	97
BCW93432.1_MAG:_hypothetical_protein_KatS3mg007_1326_[Thermoanaerobaculum_sp.]	ptg004575l	28.906	4.94E-33	144	22
BCW93430.1_MAG:_hypothetical_protein_KatS3mg007_1324_[Thermoanaerobaculum_sp.]	LG20	50	1.5E-11	61.6	61
BCW93428.1_MAG:_IS256_family_transposase_[Thermoanaerobaculum_sp.]	ptg004058l	25.357	7.74E-08	58.5	68
BCW93427.1_MAG:_hypothetical_protein_KatS3mg007_1321_[Thermoanaerobaculum_sp.]	ptg004775l	31.967	2.48E-11	69.7	31
BCW93425.1_MAG:_hypothetical_protein_KatS3mg007_1319_[Thermoanaerobaculum_sp.]	ptg004055l	41.121	7.35E-55	193	84
BCW93422.1_MAG:_UDP-N-acetylglucosamine_2-epimerase_[Thermoanaerobaculum_sp.]	ptg003768l	42.049	6.2E-72	247	96
BCW93421.1_MAG:_hypothetical_protein_KatS3mg007_1315_[Thermoanaerobaculum_sp.]	ptg004470l	29.972	4.02E-23	105	89
BCW93417.1_MAG:_ATPase_AAA_[Thermoanaerobaculum_sp.]	ptg003864l	30.943	1.63E-69	245	97
BCW93416.1_MAG:_hypothetical_protein_KatS3mg007_1310_[Thermoanaerobaculum_sp.]	ptg002848l	30.693	9.59E-11	69.7	28
BCW93413.1_MAG:_hypothetical_protein_KatS3mg007_1307_[Thermoanaerobaculum_sp.]	ptg003703l	33.846	1.15E-20	97.4	54
BCW93412.1_MAG:_hypothetical_protein_KatS3mg007_1306_[Thermoanaerobaculum_sp.]	ptg002909l	25.882	0.00000134	51.6	79
BCW93409.1_MAG:_menaquinone_biosynthesis_decarboxylase_[Thermoanaerobaculum_sp.]	ptg001995l	43.238	1.6E-118	386	99
BCW93408.1_MAG:_hypothetical_protein_KatS3mg007_1302_[Thermoanaerobaculum_sp.]	ptg003671l	27.126	1.86E-23	110	54
BCW93405.1_MAG:_glycosyl_transferase_[Thermoanaerobaculum_sp.]	ptg003592l	43.226	4.62E-69	237	95
BCW93400.1_MAG:_HD_family_phosphohydrolase_[Thermoanaerobaculum_sp.]	ptg002928l	45.238	5.94E-12	73.2	13
BCW93398.1_MAG:_molecular_chaperone_[Thermoanaerobaculum_sp.]	ptg002102l	35.338	1.54E-18	84.7	87
BCW93397.1_MAG:_polar_amino_acid_ABC_transporter_ATP-binding_protein_[Thermoanaerobaculum_sp.]	ptg005526l	41.564	5.49E-44	161	98
BCW93396.1_MAG:_amino_acid_ABC_transporter_permease_[Thermoanaerobaculum_sp.]	ptg002567l	30.286	4.45E-10	66.6	35
BCW93395.1_MAG:_acyl-CoA_dehydrogenase_[Thermoanaerobaculum_sp.]	ptg006047l	30.278	6.75E-26	114	89
BCW93394.1_MAG:_DNA_mismatch_repair_protein_MutL_[Thermoanaerobaculum_sp.]	ptg004564l	39.306	4.29E-57	211	95
BCW93393.1_MAG:_1,4-dihydroxy-6-naphtoate_synthase_[Thermoanaerobaculum_sp.]	ptg003947l	33.688	3.07E-35	137	95
BCW93392.1_MAG:_pyruvate_dehydrogenase_E1_subunit_beta_[Thermoanaerobaculum_sp.]	ptg002612l	30.215	2.5E-68	248	93
BCW93388.1_MAG:_error-prone_DNA_polymerase_[Thermoanaerobaculum_sp.]	ptg006032l	38.518	0	693	97
BCW93386.1_MAG:_hypothetical_protein_KatS3mg007_1280_[Thermoanaerobaculum_sp.]	ptg002699l	30.526	0.00000121	51.6	49
BCW93380.1_MAG:_chaperone_protein_DnaK_[Thermoanaerobaculum_sp.]	ptg005922l	61.526	0	649	94
BCW93378.1_MAG:_MerR_family_transcriptional_regulator_[Thermoanaerobaculum_sp.]	ptg004758l	52.083	0.00000684	47.4	37
BCW93377.1_MAG:_hypothetical_protein_KatS3mg007_1271_[Thermoanaerobaculum_sp.]	ptg001806l	39.3	2.25E-37	144	89
BCW93376.1_MAG:_3-hydroxybutyryl-CoA_dehydrogenase_[Thermoanaerobaculum_sp.]	ptg002612l	33.684	5.96E-38	146	94

BCW93375.1_MAG:_acetate_kinase_[Thermoanaerobaculum_sp.]	ptg004885l	45.358	9.35E-97	321	90
BCW93373.1_MAG:_hypothetical_protein_KatS3mg007_1267_[Thermoanaerobaculum_sp.]	ptg002634l	41.429	9.71E-09	62	14
BCW93370.1_MAG:_UvrABC_system_protein_C_[Thermoanaerobaculum_sp.]	ptg005527l	36.013	8.47E-84	290	97
BCW93368.1_MAG:_Sec-independent_protein_translocase_protein_TatC_[Thermoanaerobaculum_sp.]	ptg002534l	32.719	1.3E-27	114	86
BCW93366.1_MAG:_hypothetical_protein_KatS3mg007_1260_[Thermoanaerobaculum_sp.]	ptg002534l	40.468	9.35E-62	224	54
BCW93355.1_MAG:_tricorn_protease_[Thermoanaerobaculum_sp.]	ptg003980l	29.945	2.3E-117	403	97
BCW93352.1_MAG:_hypothetical_protein_KatS3mg007_1246_[Thermoanaerobaculum_sp.]	ptg004959l	28.099	5.6E-10	59.7	84
BCW93351.1_MAG:_arsenate_reductase_[Thermoanaerobaculum_sp.]	ptg004266l	50	1.2E-29	116	76
BCW93348.1_MAG:_hypothetical_protein_KatS3mg007_1242_[Thermoanaerobaculum_sp.]	ptg003299l	30.457	8.95E-33	140	50
BCW93345.1_MAG:_thioesterase_[Thermoanaerobaculum_sp.]	ptg001690l	30.357	2.9E-10	60.5	77
BCW93344.1_MAG:_K(+)-insensitive_pyrophosphate-energized_proton_pump_[Thermoanaerobaculum_sp.]	ptg004711l	40.642	1.09E-151	493	98
BCW93341.1_MAG:_NADPH-dependent_7-cyano-7-deazaguanine_reductase_[Thermoanaerobaculum_sp.]	ptg003420l	46.847	3.47E-28	111	82
BCW93340.1_MAG:_uracil-DNA_glycosylase_[Thermoanaerobaculum_sp.]	ptg004814l	48.214	6.93E-54	190	90
BCW93338.1_MAG:_ATP-dependent_helicase_[Thermoanaerobaculum_sp.]	ptg002767l	25.338	0.000000144	59.7	35
BCW93337.1_MAG:_inosine-5-monophosphate_dehydrogenase_[Thermoanaerobaculum_sp.]	ptg004810l	26.271	5.11E-09	57	76
BCW93335.1_MAG:_glycosyl_transferase_[Thermoanaerobaculum_sp.]	ptg004680l	54.202	1.57E-62	214	98
BCW93333.1_MAG:_membrane_protein_[Thermoanaerobaculum_sp.]	LG12	42.342	1.05E-14	78.6	66
BCW93328.1_MAG:_NifU_family_protein_[Thermoanaerobaculum_sp.]	ptg003708l	37.705	2E-10	58.2	79
BCW93327.1_MAG:_hypothetical_protein_KatS3mg007_1221_[Thermoanaerobaculum_sp.]	ptg002465l	30.994	9.77E-13	69.3	89
BCW93324.1_MAG:_1-pyrroline-5-carboxylate_dehydrogenase_[Thermoanaerobaculum_sp.]	ptg004321l	28.14	5.46E-32	135	77
BCW93323.1_MAG:_hypothetical_protein_KatS3mg007_1217_[Thermoanaerobaculum_sp.]	ptg004085l	31.452	0.000000196	56.2	40
BCW93322.1_MAG:_hypothetical_protein_KatS3mg007_1216_[Thermoanaerobaculum_sp.]	ptg004085l	29.808	3.28E-10	67	38
BCW93320.1_MAG:_tRNA-specific_2-thiouridylase_MnmA_[Thermoanaerobaculum_sp.]	ptg003764l	42.216	1.94E-85	285	99
BCW93319.1_MAG:_hypothetical_protein_KatS3mg007_1213_[Thermoanaerobaculum_sp.]	ptg001238l	48.485	0.000000282	53.9	31
BCW93318.1_MAG:_cysteine_desulfurase_[Thermoanaerobaculum_sp.]	ptg004651l	35.641	1.57E-59	212	94
BCW93317.1_MAG:_replicative_DNA_helicase_[Thermoanaerobaculum_sp.]	ptg003872l	39.15	1.02E-80	276	97
BCW93316.1_MAG:_ABC_transporter_permease_[Thermoanaerobaculum_sp.]	ptg003336l	44.872	4.38E-48	174	90
BCW93315.1_MAG:_ABC_transporter_ATP-binding_protein_[Thermoanaerobaculum_sp.]	ptg003137l	41.833	5.03E-58	202	93
BCW93311.1_MAG:_HD_family_phosphohydrolase_[Thermoanaerobaculum_sp.]	ptg003673l	25.824	9.28E-10	64.3	48
BCW93309.1_MAG:_SAM-dependent_methyltransferase_[Thermoanaerobaculum_sp.]	ptg005689l	28.279	4.77E-15	52.4	91
BCW93307.1_MAG:_hypothetical_protein_KatS3mg007_1201_[Thermoanaerobaculum_sp.]	ptg005920l	26.667	6.07E-11	66.6	67
BCW93306.1_MAG:_hypothetical_protein_KatS3mg007_1200_[Thermoanaerobaculum_sp.]	ptg005260l	39.224	2.49E-39	156	46
BCW93295.1_MAG:_primosomal_protein_N'_[Thermoanaerobaculum_sp.]	ptg002760l	41.186	5.9E-115	389	72
BCW93293.1_MAG:_peptidase_T_[Thermoanaerobaculum_sp.]	ptg001690l	43.029	2.27E-110	360	98
BCW93292.1_MAG:_UvrABC_system_protein_B_[Thermoanaerobaculum_sp.]	ptg004926l	58.548	0	771	98

BCW93290.1_MAG:_penicillin-binding_protein_[Thermoanaerobaculum_sp.]	ptg004863l	32.107	2.04E-106	363	88
BCW93289.1_MAG:_inorganic_phosphate_transporter_[Thermoanaerobaculum_sp.]	ptg004169l	34.109	5.19E-12	70.9	37
BCW93287.1_MAG:_hypothetical_protein_KatS3mg007_1181_[Thermoanaerobaculum_sp.]	ptg003334l	37.931	3.94E-11	63.2	59
BCW93286.1_MAG:_hypothetical_protein_KatS3mg007_1180_[Thermoanaerobaculum_sp.]	ptg003282l	54.878	2.33E-16	88.2	11
BCW93284.1_MAG:_transcriptional_regulator_[Thermoanaerobaculum_sp.]	ptg002871l	33.803	6.72E-15	75.1	84
BCW93283.1_MAG:_homoserine_dehydrogenase_[Thermoanaerobaculum_sp.]	ptg004284l	34.118	5.63E-37	145	97
BCW93282.1_MAG:_aldehyde_dehydrogenase_[Thermoanaerobaculum_sp.]	ptg004037l	37.076	4.44E-89	301	98
BCW93280.1_MAG:_heme_ABC_exporter_ATP-binding_protein_CcmA_[Thermoanaerobaculum_sp.]	ptg004203l	35.754	6.05E-25	105	97
BCW93278.1_MAG:_hypothetical_protein_KatS3mg007_1172_[Thermoanaerobaculum_sp.]	ptg001025l	31.469	0.00000398	50.8	63
BCW93275.1_MAG:_cytochrome_c_assembly_protein_[Thermoanaerobaculum_sp.]	ptg002942l	41.107	1.46E-37	156	30
BCW93271.1_MAG:_50S_ribosomal_protein_L9_[Thermoanaerobaculum_sp.]	ptg005487l	36.301	2.6E-21	92.4	95
BCW93269.1_MAG:_30S_ribosomal_protein_S18_[Thermoanaerobaculum_sp.]	ptg005487l	55.172	1.27E-15	72.8	76
BCW93268.1_MAG:_hypothetical_protein_KatS3mg007_1162_[Thermoanaerobaculum_sp.]	ptg005487l	29.67	0.00000146	49.7	65
BCW93267.1_MAG:_peptidyl-tRNA_hydrolase_[Thermoanaerobaculum_sp.]	ptg005867l	40.782	3.18E-26	108	93
BCW93266.1_MAG:_50S_ribosomal_protein_L25_[Thermoanaerobaculum_sp.]	ptg004982l	39.412	1.23E-23	102	74
BCW93265.1_MAG:_ribose-phosphate_pyrophosphokinase_[Thermoanaerobaculum_sp.]	ptg004982l	55.663	2.66E-113	363	98
BCW93264.1_MAG:_thiol:disulfide_interchange_protein_DsbD_[Thermoanaerobaculum_sp.]	ptg004779l	29.787	2.85E-24	112	59
BCW93263.1_MAG:_alpha-L-glycero-D-manno-heptose_beta-1,4-glucosyltransferase_[Thermoanaerobaculum_sp.]	ptg003625l	38.735	6.24E-47	172	87
BCW93262.1_MAG:_isocitrate_dehydrogenase_[NADP]_[Thermoanaerobaculum_sp.]	ptg002573l	34.615	7.6E-16	84	37
BCW93261.1_MAG:_cytidine_deaminase_[Thermoanaerobaculum_sp.]	ptg000909l	51.2	6.04E-28	110	92
BCW93260.1_MAG:_hypothetical_protein_KatS3mg007_1154_[Thermoanaerobaculum_sp.]	ptg002304l	26.562	2.45E-22	106	50
BCW93259.1_MAG:_short_chain_dehydrogenase_[Thermoanaerobaculum_sp.]	ptg005905l	33.333	1.43E-11	68.2	69
BCW93258.1_MAG:_tRNA-dihydrouridine_synthase_[Thermoanaerobaculum_sp.]	ptg004321l	36.624	2.44E-50	183	94
BCW93257.1_MAG:_hypothetical_protein_KatS3mg007_1151_[Thermoanaerobaculum_sp.]	ptg005536l	27.143	3.74E-09	62.8	37
BCW93255.1_MAG:_selenide,_water_dikinase_[Thermoanaerobaculum_sp.]	ptg001871l	34.483	8.35E-35	137	92
BCW93253.1_MAG:_T-protein_[Thermoanaerobaculum_sp.]	ptg005341l	25.568	4.47E-18	89.7	95
BCW93252.1_MAG:_hypothetical_protein_KatS3mg007_1146_[Thermoanaerobaculum_sp.]	ptg002505l	23.301	1.56E-13	74.7	96
BCW93251.1_MAG:_DUF159_family_protein_[Thermoanaerobaculum_sp.]	ptg003566l	30.29	8.04E-21	94	98
BCW93250.1_MAG:_alpha/beta_hydrolase_[Thermoanaerobaculum_sp.]	ptg004564l	30.131	1.17E-18	89	86
BCW93249.1_MAG:_hypothetical_protein_KatS3mg007_1143_[Thermoanaerobaculum_sp.]	ptg004186l	37.143	0.000000899	53.5	28
BCW93247.1_MAG:_benzoate--CoA_ligase_[Thermoanaerobaculum_sp.]	ptg005087l	28.6	5.27E-25	113	92
BCW93244.1_MAG:_dihydroorotase_[Thermoanaerobaculum_sp.]	LG04	44.268	8.56E-72	228	97
BCW93243.1_MAG:_aspartate_carbamoyltransferase_[Thermoanaerobaculum_sp.]	ptg005190l	52.601	5.28E-71	181	82
BCW93237.1_MAG:_penicillin_amidase_[Thermoanaerobaculum_sp.]	ptg004534l	27.978	4.02E-43	173	85
BCW93233.1_MAG:_phosphoglucomutase_[Thermoanaerobaculum_sp.]	ptg005563l	33.333	4.82E-16	87.4	22

BCW93231.1_MAG:_galactose-1-phosphate_uridylyltransferase_[Thermoanaerobaculum_sp.]	ptg003785l	31.549	5.43E-48	177	97
BCW93229.1_MAG:_hypothetical_protein_KatS3mg007_1123_[Thermoanaerobaculum_sp.]	ptg003785l	39.189	6.17E-58	206	82
BCW93226.1_MAG:_hypothetical_protein_KatS3mg007_1120_[Thermoanaerobaculum_sp.]	ptg005398l	39.815	1.7E-17	90.5	20
BCW93225.1_MAG:_NAD(P)_transhydrogenase_subunit_alpha_[Thermoanaerobaculum_sp.]	ptg004054l	45.045	2.39E-74	254	89
BCW93224.1_MAG:_hypothetical_protein_KatS3mg007_1118_[Thermoanaerobaculum_sp.]	ptg005929l	62.025	2.73E-21	89.7	85
BCW93223.1_MAG:_NAD(P)_transhydrogenase_subunit_beta_[Thermoanaerobaculum_sp.]	ptg005929l	52.632	2.94E-104	344	97
BCW93222.1_MAG:_hypothetical_protein_KatS3mg007_1116_[Thermoanaerobaculum_sp.]	ptg005110l	34.694	9.27E-15	80.1	36
BCW93221.1_MAG:_MFS_transporter_[Thermoanaerobaculum_sp.]	ptg002429l	30.688	1.67E-15	82.8	43
BCW93219.1_MAG:_hypothetical_protein_KatS3mg007_1113_[Thermoanaerobaculum_sp.]	ptg003471l	30.213	5.26E-14	75.9	79
BCW93218.1_MAG:_endopeptidase_IV_[Thermoanaerobaculum_sp.]	ptg004077l	33.178	1.54E-51	194	73
BCW93217.1_MAG:_oligoendopeptidase_F_[Thermoanaerobaculum_sp.]	ptg005169l	33.333	2.28E-85	295	97
BCW93216.1_MAG:_S-adenosylmethionine:tRNA_ribosyltransferase-isomerase_[Thermoanaerobaculum_sp.]	ptg004256l	40.171	1.28E-69	239	99
BCW93213.1_MAG:_hypothetical_protein_KatS3mg007_1107_[Thermoanaerobaculum_sp.]	ptg001925l	26.178	5.61E-15	84.3	34
BCW93212.1_MAG:_polyisoprenoid-binding_protein_[Thermoanaerobaculum_sp.]	ptg002928l	32.222	8.48E-17	81.3	88
BCW93211.1_MAG:_putative_tRNA_(cytidine(34)-2'-O)-methyltransferase_[Thermoanaerobaculum_sp.]	ptg003973l	46.281	5.04E-28	112	78
BCW93210.1_MAG:_histidine_kinase_[Thermoanaerobaculum_sp.]	ptg005484l	33.679	1.34E-43	175	49
BCW93209.1_MAG:_tryptophanase_[Thermoanaerobaculum_sp.]	ptg004361l	57.08	1.58E-168	529	99
BCW93208.1_MAG:_hypothetical_protein_KatS3mg007_1102_[Thermoanaerobaculum_sp.]	ptg000834l	33.824	3.63E-13	70.9	70
BCW93206.1_MAG:_30S_ribosomal_protein_S20_[Thermoanaerobaculum_sp.]	ptg002505l	44.872	0.000000386	49.7	81
BCW93205.1_MAG:_phosphoribosylaminoimidazole-succinocarboxamide_synthase_[Thermoanaerobaculum_sp.]	ptg004573l	45.918	2.09E-43	91.3	77
BCW93204.1_MAG:_elongation_factor_G_[Thermoanaerobaculum_sp.]	ptg002658l	35.043	3.55E-132	434	97
BCW93201.1_MAG:_UDP-glucose_4-epimerase_[Thermoanaerobaculum_sp.]	ptg003768l	34.385	1.3E-47	174	99
BCW93199.1_MAG:_hypothetical_protein_KatS3mg007_1093_[Thermoanaerobaculum_sp.]	ptg002557l	30.769	3.56E-19	91.3	77
BCW93198.1_MAG:_putative_pterin-4-alpha-carbinolamine_dehydratase_[Thermoanaerobaculum_sp.]	ptg000492l	42.593	4.75E-22	92.8	95
BCW93197.1_MAG:_DNA_helicase_[Thermoanaerobaculum_sp.]	ptg002907l	32.847	2.53E-81	286	94
BCW93195.1_MAG:_oxidoreductase_[Thermoanaerobaculum_sp.]	ptg005035l	27.378	2.64E-54	199	97
BCW93194.1_MAG:_methylmalonyl-CoA_mutase_[Thermoanaerobaculum_sp.]	LG30	46.667	1.44E-08	56.6	45
BCW93192.1_MAG:_glutamine--fructose-6-phosphate_aminotransferase_[Thermoanaerobaculum_sp.]	ptg002878l	29.299	5.59E-66	238	100
BCW93191.1_MAG:_tungsten_formylmethanofuran_dehydrogenase_subunit_E_[Thermoanaerobaculum_sp.]	ptg002612l	35.328	3.74E-114	382	99
BCW93190.1_MAG:_universal_stress_protein_UspA_[Thermoanaerobaculum_sp.]	ptg005333l	25.623	3.97E-13	73.2	97
BCW93189.1_MAG:_peroxiredoxin_[Thermoanaerobaculum_sp.]	ptg004538l	32.258	3.78E-12	67	70
BCW93187.1_MAG:_hypothetical_protein_KatS3mg007_1081_[Thermoanaerobaculum_sp.]	ptg005865l	38.614	1.86E-10	66.2	28
BCW93186.1_MAG:_hypothetical_protein_KatS3mg007_1080_[Thermoanaerobaculum_sp.]	ptg005484l	29	1.45E-33	144	46
BCW93185.1_MAG:_peptidase_M16_[Thermoanaerobaculum_sp.]	ptg002214l	26.46	1.91E-77	280	97

BCW93183.1_MAG:_putative_2,3-bisphosphoglycerate-independent_phosphoglycerate_mutase_[Thermoanaerobaculum_sp.]	ptg002573l	26.221	2.6E-21	100	90
BCW93182.1_MAG:_hypothetical_protein_KatS3mg007_1076_[Thermoanaerobaculum_sp.]	ptg004468l	42.655	1.33E-75	262	73
BCW93180.1_MAG:_bifunctional_NAD(P)H-hydrate_repair_enzyme_Nnr_[Thermoanaerobaculum_sp.]	ptg005732l	50	1.27E-18	94	21
BCW93177.1_MAG:_hypothetical_protein_KatS3mg007_1071_[Thermoanaerobaculum_sp.]	ptg003134l	35.484	2.45E-14	80.1	40
BCW93176.1_MAG:_ATP-dependent_DNA_helicase_RecG_[Thermoanaerobaculum_sp.]	ptg001995l	40.393	2.37E-118	394	93
BCW93173.1_MAG:_ribosomal_RNA_small_subunit_methyltransferase_E_[Thermoanaerobaculum_sp.]	ptg002882l	30.625	2.18E-13	72.8	61
BCW93171.1_MAG:_chaperone_protein_DnaJ_[Thermoanaerobaculum_sp.]	ptg003680l	47.268	3.76E-71	245	98
BCW93170.1_MAG:_chaperone_protein_DnaK_[Thermoanaerobaculum_sp.]	ptg005922l	54.389	0	618	94
BCW93169.1_MAG:_hypothetical_protein_KatS3mg007_1063_[Thermoanaerobaculum_sp.]	ptg005922l	38.095	4.03E-23	100	66
BCW93166.1_MAG:_cold-shock_protein_[Thermoanaerobaculum_sp.]	ptg003498l	70.492	1.3E-23	95.1	92
BCW93165.1_MAG:_NADH-quinone_oxidoreductase_subunit_I_[Thermoanaerobaculum_sp.]	ptg004143l	34.615	7.4E-21	92.8	82
BCW93164.1_MAG:_hypothetical_protein_KatS3mg007_1058_[Thermoanaerobaculum_sp.]	ptg003419l	39.426	1.07E-91	312	68
BCW93163.1_MAG:_NADH-quinone_oxidoreductase_subunit_B_[Thermoanaerobaculum_sp.]	ptg004143l	50.685	1.07E-44	160	86
BCW93162.1_MAG:_hypothetical_protein_KatS3mg007_1056_[Thermoanaerobaculum_sp.]	ptg003673l	36.957	0.00000071	53.1	38
BCW93161.1_MAG:_tyrosine_recombinase_XerD_[Thermoanaerobaculum_sp.]	ptg003734l	40.26	2.81E-57	202	100
BCW93160.1_MAG:_methyltransferase_[Thermoanaerobaculum_sp.]	ptg004790l	50	0.00000442	39.7	50
BCW93159.1_MAG:_NAD-dependent_malic_enzyme_[Thermoanaerobaculum_sp.]	ptg002214l	42.647	1.57E-112	373	94
BCW93158.1_MAG:_PTS_fructose_transporter_subunit_IIA_[Thermoanaerobaculum_sp.]	ptg003536l	34.286	2.32E-16	79.3	81
BCW93155.1_MAG:_thioredoxin_family_protein_[Thermoanaerobaculum_sp.]	ptg004523l	43.411	4.81E-27	111	64
BCW93154.1_MAG:_hypothetical_protein_KatS3mg007_1048_[Thermoanaerobaculum_sp.]	ptg004104l	29.915	0.000000038	55.1	75
BCW93152.1_MAG:_bifunctional_homocysteine_S-methyltransferase/methylenetetrahydrofolate_reductase_[Thermoanaerobaculum_sp.]	ptg001897l	29.084	3.8E-09	63.9	35
BCW93151.1_MAG:_methionine_synthase_[Thermoanaerobaculum_sp.]	ptg002021l	32.208	2.23E-166	551	99
BCW93145.1_MAG:_ABC_transporter_permease_[Thermoanaerobaculum_sp.]	ptg004986l	28.182	2.96E-20	97.1	49
BCW93144.1_MAG:_ABC_transporter_permease_[Thermoanaerobaculum_sp.]	ptg004986l	27.826	7.29E-16	83.6	77
BCW93143.1_MAG:_ABC_transporter_ATP-binding_protein_[Thermoanaerobaculum_sp.]	ptg005920l	47.907	6.6E-63	215	96
BCW93142.1_MAG:_RND_transporter_[Thermoanaerobaculum_sp.]	ptg000732l	27.374	0.000000301	56.6	47
BCW93141.1_MAG:_hypothetical_protein_KatS3mg007_1035_[Thermoanaerobaculum_sp.]	ptg005309l	35.178	1.93E-12	74.7	40
BCW93138.1_MAG:_hypothetical_protein_KatS3mg007_1032_[Thermoanaerobaculum_sp.]	ptg001607l	37.551	1.4E-32	137	87
BCW93134.1_MAG:_protein_translocase_subunit_SecA_[Thermoanaerobaculum_sp.]	ptg005005l	52.164	0	815	91
BCW93133.1_MAG:_hypothetical_protein_KatS3mg007_1027_[Thermoanaerobaculum_sp.]	ptg005195l	28.4	8.58E-14	78.6	48
BCW93132.1_MAG:_hypothetical_protein_KatS3mg007_1026_[Thermoanaerobaculum_sp.]	ptg003861l	28.571	2.06E-11	63.9	99
BCW93128.1_MAG:_hypothetical_protein_KatS3mg007_1022_[Thermoanaerobaculum_sp.]	ptg006032l	35.178	5.09E-29	129	27
BCW93127.1_MAG:_hypothetical_protein_KatS3mg007_1021_[Thermoanaerobaculum_sp.]	ptg005150l	42.46	1.45E-54	205	40

BCW93125.1_MAG:_ABC_transporter_ATP-binding_protein_[Thermoanaerobaculum_sp.]	ptg000372l	27.605	5.33E-42	166	91
BCW93124.1_MAG:_tRNA_(cytidine/uridine-2'-O-)-methyltransferase_TrmJ_[Thermoanaerobaculum_sp.]	ptg002223l	34.934	2.81E-22	99	87
BCW93120.1_MAG:_acyl-CoA_dehydrogenase_[Thermoanaerobaculum_sp.]	ptg004179l	32.665	4.04E-37	147	89
BCW93117.1_MAG:_A/G-specific_adenine_glycosylase_[Thermoanaerobaculum_sp.]	ptg005844l	28.065	1.69E-22	102	85
BCW93116.1_MAG:_hypothetical_protein_KatS3mg007_1010_[Thermoanaerobaculum_sp.]	ptg002634l	46.667	9.47E-16	76.3	63
BCW93114.1_MAG:_acetoacetate_metabolism_regulatory_protein_AtoC_[Thermoanaerobaculum_sp.]	ptg005150l	40.95	2.6E-101	336	96
BCW93113.1_MAG:_DNA_ligase_[Thermoanaerobaculum_sp.]	ptg005087l	44.411	4.29E-151	488	99
BCW93112.1_MAG:_poly(A)_polymerase_I_[Thermoanaerobaculum_sp.]	ptg003220l	38.627	1.7E-33	137	53
BCW93111.1_MAG:_hypothetical_protein_KatS3mg007_1005_[Thermoanaerobaculum_sp.]	ptg004026l	28.426	6.32E-10	60.8	97
BCW93109.1_MAG:_histone_deacetylase_[Thermoanaerobaculum_sp.]	ptg005274l	28.994	5.05E-10	64.7	51
BCW93107.1_MAG:_transcriptional_repressor_NrdR_[Thermoanaerobaculum_sp.]	ptg004151l	44.218	5.96E-35	131	96
BCW93105.1_MAG:_Lon_protease_[Thermoanaerobaculum_sp.]	ptg005527l	46.164	0	682	95
BCW93103.1_MAG:_epimerase_[Thermoanaerobaculum_sp.]	ptg005433l	52.769	2.54E-95	312	97
BCW93102.1_MAG:_acyl-CoA_dehydrogenase_[Thermoanaerobaculum_sp.]	ptg005087l	37.134	5.18E-108	361	98
BCW93094.1_MAG:_ABC_transporter_ATP-binding_protein_[Thermoanaerobaculum_sp.]	ptg004403l	32.302	2.76E-53	201	79
BCW93093.1_MAG:_thymidine_phosphorylase_[Thermoanaerobaculum_sp.]	ptg001353l	47.074	1.8E-81	277	86
BCW93091.1_MAG:_peroxiredoxin_[Thermoanaerobaculum_sp.]	ptg004538l	47.321	3.58E-28	112	71
BCW93089.1_MAG:_alcohol_dehydrogenase_[Thermoanaerobaculum_sp.]	ptg001604l	28.014	0.00000285	53.1	82
BCW93085.1_MAG:_hypothetical_protein_KatS3mg007_0979_[Thermoanaerobaculum_sp.]	ptg002214l	23.009	3.39E-16	85.9	90
BCW93084.1_MAG:_peptidase_M16_[Thermoanaerobaculum_sp.]	ptg005195l	33.04	2.61E-18	93.6	48
BCW93083.1_MAG:_angiotensin-converting_enzyme_[Thermoanaerobaculum_sp.]	ptg003548l	23.704	0.00000111	55.8	45
BCW93082.1_MAG:_glutamine--scyllo-inositol_aminotransferase_[Thermoanaerobaculum_sp.]	ptg004525l	50.815	4.11E-104	340	97
BCW93081.1_MAG:_rhomboid_family_intramembrane_serine_protease_[Thermoanaerobaculum_sp.]	ptg005092l	41.228	1.22E-31	125	94
BCW93072.1_MAG:_RNA_polymerase_sigma_factor_[Thermoanaerobaculum_sp.]	ptg002465l	32.857	0.00000108	52	70
BCW93070.1_MAG:_hypothetical_protein_KatS3mg007_0964_[Thermoanaerobaculum_sp.]	ptg003090l	28.731	1.14E-30	124	94
BCW93069.1_MAG:_hypothetical_protein_KatS3mg007_0963_[Thermoanaerobaculum_sp.]	ptg002992l	27.645	4.7E-09	61.2	89
BCW93067.1_MAG:_glutamine_synthetase_[Thermoanaerobaculum_sp.]	ptg004075l	35.456	5.68E-66	233	99
BCW93064.1_MAG:_hypothetical_protein_KatS3mg007_0958_[Thermoanaerobaculum_sp.]	ptg004509l	41.905	1.32E-14	75.5	50
BCW93061.1_MAG:_hypothetical_protein_KatS3mg007_0955_[Thermoanaerobaculum_sp.]	ptg003592l	37.383	9.36E-17	80.5	61
BCW93059.1_MAG:_short-chain_dehydrogenase_[Thermoanaerobaculum_sp.]	ptg002573l	36.29	1.44E-25	108	97
BCW93057.1_MAG:_hypothetical_protein_KatS3mg007_0951_[Thermoanaerobaculum_sp.]	ptg005067l	40.856	5.26E-38	144	97
BCW93054.1_MAG:_GMP_synthase_[glutamine-hydrolyzing]_[Thermoanaerobaculum_sp.]	ptg002345l	56.14	3.92E-161	510	99
BCW93053.1_MAG:_arabinose-5-phosphate_isomerase_[Thermoanaerobaculum_sp.]	ptg005611l	44.937	5.29E-68	234	99
BCW93052.1_MAG:_protein-L-isoaspartate_O-methyltransferase_[Thermoanaerobaculum_sp.]	ptg004256l	49.49	2.83E-42	155	88
BCW93051.1_MAG:_5'-nucleotidase_SurE_[Thermoanaerobaculum_sp.]	ptg005398l	42.276	3.91E-48	174	92
BCW93049.1_MAG:_hypothetical_protein_KatS3mg007_0943_[Thermoanaerobaculum_sp.]	ptg005309l	37.857	4.41E-15	83.6	19

BCW93047.1_MAG:_hypothetical_protein_KatS3mg007_0941_[Thermoanaerobaculum_sp.]	ptg005484l	32.067	1.66E-41	171	41
BCW93046.1_MAG:_carbon_starvation_protein_A_[Thermoanaerobaculum_sp.]	ptg002928l	35.484	5.08E-57	210	83
BCW93044.1_MAG:_Xaa-Pro_aminopeptidase_[Thermoanaerobaculum_sp.]	ptg001995l	30.067	4.67E-46	174	95
BCW93041.1_MAG:_Crp/Fnr_family_transcriptional_regulator_[Thermoanaerobaculum_sp.]	ptg002907l	26.761	4.28E-18	86.3	94
BCW93038.1_MAG:_tRNA_dimethylallyltransferase_[Thermoanaerobaculum_sp.]	ptg004997l	39.781	1.09E-55	197	89
BCW93037.1_MAG:_DNA_internalization-related_competence_protein_ComEC/Rec2_[Thermoanaerobaculum_sp.]	ptg005976l	28.163	1.2E-15	85.9	31
BCW93036.1_MAG:_putative_RNA_methyltransferase_[Thermoanaerobaculum_sp.]	ptg002278l	40.541	0.000000065	58.9	18
BCW93034.1_MAG:_type_III_pantothenate_kinase_[Thermoanaerobaculum_sp.]	ptg002214l	30.047	3.54E-12	69.7	78
BCW93032.1_MAG:_valine--tRNA_ligase_[Thermoanaerobaculum_sp.]	ptg001925l	44.605	0	775	96
BCW93031.1_MAG:_3-dehydroquinate_synthase_[Thermoanaerobaculum_sp.]	ptg003363l	43.043	6.18E-43	163	61
BCW93030.1_MAG:_2-oxoglutarate_ferredoxin_oxidoreductase_subunit_alpha_[Thermoanaerobaculum_sp.]	ptg002102l	27.35	9.98E-16	82.8	92
BCW93029.1_MAG:_hypothetical_protein_KatS3mg007_0923_[Thermoanaerobaculum_sp.]	ptg002867l	31.579	7.48E-15	80.9	45
BCW93027.1_MAG:_alanine_racemase_[Thermoanaerobaculum_sp.]	ptg004959l	29.282	7.01E-40	155	90
BCW93026.1_MAG:_4-hydroxybenzoyl-CoA_thioesterase_[Thermoanaerobaculum_sp.]	ptg003536l	40.8	1.73E-18	83.6	93
BCW93025.1_MAG:_NAD-dependent_succinate-semialdehyde_dehydrogenase_[Thermoanaerobaculum_sp.]	ptg004321l	33.17	1.34E-52	195	85
BCW93024.1_MAG:_hypothetical_protein_KatS3mg007_0918_[Thermoanaerobaculum_sp.]	ptg000619l	27.698	1.25E-17	87.8	80
BCW93023.1_MAG:_nicotinate_phosphoribosyltransferase_[Thermoanaerobaculum_sp.]	ptg005048l	39.868	4.56E-87	295	92
BCW93021.1_MAG:_hypothetical_protein_KatS3mg007_0915_[Thermoanaerobaculum_sp.]	ptg003057l	43.082	8.42E-53	208	25
BCW93020.1_MAG:_3-phosphoshikimate_1-carboxyvinyltransferase_[Thermoanaerobaculum_sp.]	ptg002123l	33.82	2.55E-55	201	94
BCW93018.1_MAG:_chromosome_partition_protein_Smc_[Thermoanaerobaculum_sp.]	ptg004306l	44.048	1.94E-51	203	39
BCW93013.1_MAG:_3-deoxy-7-phosphoheptulonate_synthase_[Thermoanaerobaculum_sp.]	ptg003247l	28.063	2.83E-12	72	68
BCW93010.1_MAG:_phosphopantetheine_adenylyltransferase_[Thermoanaerobaculum_sp.]	ptg004885l	49.664	3.12E-42	153	90
BCW93007.1_MAG:_electron_transfer_flavoprotein_subunit_alpha_[Thermoanaerobaculum_sp.]	LG19	70.833	5.1E-24	106	63
BCW93006.1_MAG:_electron_transfer_flavoprotein_subunit_beta_[Thermoanaerobaculum_sp.]	ptg005554l	32.78	1.71E-31	126	91
BCW93005.1_MAG:_3-oxoacyl-[acyl-carrier-protein]_synthase_2_[Thermoanaerobaculum_sp.]	ptg003980l	48.985	3.56E-80	273	93
BCW93004.1_MAG:_acyl_carrier_protein_[Thermoanaerobaculum_sp.]	ptg004340l	60.274	6.57E-17	76.6	92
BCW93003.1_MAG:_hypothetical_protein_KatS3mg007_0897_[Thermoanaerobaculum_sp.]	ptg005484l	32.468	1.84E-45	182	42
BCW93002.1_MAG:_glycerol-3-phosphate_acyltransferase_[Thermoanaerobaculum_sp.]	LG04	40.314	5.57E-08	55.8	94
BCW92998.1_MAG:_ABC_transporter_permease_[Thermoanaerobaculum_sp.]	ptg003336l	41.026	8.19E-37	142	87
BCW92997.1_MAG:_ABC_transporter_ATP-binding_protein_[Thermoanaerobaculum_sp.]	ptg003336l	42.009	6.29E-40	150	85
BCW92995.1_MAG:_epimerase_[Thermoanaerobaculum_sp.]	ptg001690l	39.617	1.34E-69	238	99
BCW92991.1_MAG:_peptidase_S9_[Thermoanaerobaculum_sp.]	ptg003382l	35.025	2.09E-103	352	80
BCW92990.1_MAG:_hypothetical_protein_KatS3mg007_0884_[Thermoanaerobaculum_sp.]	ptg001925l	49.582	3.34E-110	365	63
BCW92987.1_MAG:_hypothetical_protein_KatS3mg007_0881_[Thermoanaerobaculum_sp.]	ptg004077l	39.888	7.72E-38	145	65

BCW92986.1_MAG:_2-oxoglutarate_ferredoxin_oxidoreductase_subunit_alpha_[Thermoanaerobaculum_sp.]	ptg002867l	32.743	6.25E-38	149	86
BCW92980.1_MAG:_biopolymer_transporter_ExbD_[Thermoanaerobaculum_sp.]	ptg005754l	31.852	2.52E-08	54.7	94
BCW92979.1_MAG:_hypothetical_protein_KatS3mg007_0873_[Thermoanaerobaculum_sp.]	ptg004136l	56.604	1.07E-34	130	74
BCW92978.1_MAG:_hypothetical_protein_KatS3mg007_0872_[Thermoanaerobaculum_sp.]	ptg004361l	44.025	5.79E-87	288	99
BCW92975.1_MAG:_hypothetical_protein_KatS3mg007_0869_[Thermoanaerobaculum_sp.]	ptg005107l	38.554	3.33E-08	54.7	58
BCW92973.1_MAG:_multidrug_transporter_AcrB_[Thermoanaerobaculum_sp.]	ptg005692l	27.907	2.1E-39	164	42
BCW92972.1_MAG:_acriflavin_resistance_protein_[Thermoanaerobaculum_sp.]	ptg000732l	24.479	0.000000125	57.4	50
BCW92969.1_MAG:_50S_ribosomal_protein_L17_[Thermoanaerobaculum_sp.]	ptg004509l	51.724	8.46E-32	122	83
BCW92968.1_MAG:_DNA-directed_RNA_polymerase_subunit_alpha_[Thermoanaerobaculum_sp.]	ptg005964l	41.925	2.21E-73	249	99
BCW92967.1_MAG:_30S_ribosomal_protein_S4_[Thermoanaerobaculum_sp.]	ptg004509l	55.024	1.04E-62	213	100
BCW92966.1_MAG:_30S_ribosomal_protein_S11_[Thermoanaerobaculum_sp.]	ptg001245l	66.942	1.83E-43	154	92
BCW92965.1_MAG:_30S_ribosomal_protein_S13_[Thermoanaerobaculum_sp.]	ptg004509l	59.483	4.69E-39	142	91
BCW92964.1_MAG:_50S_ribosomal_protein_L36_[Thermoanaerobaculum_sp.]	ptg004564l	72.973	2.45E-11	58.5	100
BCW92963.1_MAG:_translation_initiation_factor_IF-1_[Thermoanaerobaculum_sp.]	ptg004746l	75	1.56E-29	112	100
BCW92962.1_MAG:_type_I_methionyl_aminopeptidase_[Thermoanaerobaculum_sp.]	ptg002436l	47.561	8.07E-74	247	98
BCW92961.1_MAG:_adenylate_kinase_[Thermoanaerobaculum_sp.]	LG12	49.057	1.14E-08	58.5	24
BCW92960.1_MAG:_protein_translocase_subunit_SecY_[Thermoanaerobaculum_sp.]	ptg004509l	44.69	5.53E-111	363	97
BCW92959.1_MAG:_50S_ribosomal_protein_L15_[Thermoanaerobaculum_sp.]	ptg005891l	54.615	2.49E-32	124	87
BCW92957.1_MAG:_30S_ribosomal_protein_S5_[Thermoanaerobaculum_sp.]	ptg004509l	56.489	6.65E-39	144	74
BCW92956.1_MAG:_50S_ribosomal_protein_L18_[Thermoanaerobaculum_sp.]	ptg005254l	51.515	5.47E-27	107	81
BCW92955.1_MAG:_50S_ribosomal_protein_L6_[Thermoanaerobaculum_sp.]	ptg001624l	44.693	7.67E-38	141	99
BCW92954.1_MAG:_30S_ribosomal_protein_S8_[Thermoanaerobaculum_sp.]	ptg004509l	54.545	2.39E-33	126	100
BCW92953.1_MAG:_30S_ribosomal_protein_S14_type_Z_[Thermoanaerobaculum_sp.]	ptg005254l	68.182	5.21E-13	64.7	72
BCW92952.1_MAG:_50S_ribosomal_protein_L5_[Thermoanaerobaculum_sp.]	ptg004509l	54.237	2.42E-63	214	98
BCW92951.1_MAG:_50S_ribosomal_protein_L24_[Thermoanaerobaculum_sp.]	ptg004509l	50.485	3.34E-27	107	95
BCW92950.1_MAG:_50S_ribosomal_protein_L14_[Thermoanaerobaculum_sp.]	ptg004509l	61.789	6.01E-44	155	100
BCW92949.1_MAG:_30S_ribosomal_protein_S17_[Thermoanaerobaculum_sp.]	ptg004509l	44.595	7.73E-14	68.2	85
BCW92948.1_MAG:_50S_ribosomal_protein_L29_[Thermoanaerobaculum_sp.]	ptg004509l	46.667	4.54E-10	56.6	92
BCW92947.1_MAG:_50S_ribosomal_protein_L16_[Thermoanaerobaculum_sp.]	ptg001624l	61.062	6.28E-44	156	82
BCW92946.1_MAG:_30S_ribosomal_protein_S3_[Thermoanaerobaculum_sp.]	ptg004509l	57.488	5.14E-61	209	96
BCW92945.1_MAG:_50S_ribosomal_protein_L22_[Thermoanaerobaculum_sp.]	ptg004509l	46.364	1.64E-19	85.9	94
BCW92944.1_MAG:_30S_ribosomal_protein_S19_[Thermoanaerobaculum_sp.]	ptg004509l	60	6.69E-32	120	96
BCW92943.1_MAG:_50S_ribosomal_protein_L2_[Thermoanaerobaculum_sp.]	ptg005891l	62.182	3.71E-95	310	100
BCW92942.1_MAG:_50S_ribosomal_protein_L23_[Thermoanaerobaculum_sp.]	ptg004509l	42.857	2.45E-15	73.2	86

BCW92941.1_MAG:_50S_ribosomal_protein_L4_[Thermoanaerobaculum_sp.]	ptg004509l	39.894	1.37E-29	118	90
BCW92940.1_MAG:_50S_ribosomal_protein_L3_[Thermoanaerobaculum_sp.]	ptg001624l	42.647	6.44E-33	128	99
BCW92939.1_MAG:_30S_ribosomal_protein_S10_[Thermoanaerobaculum_sp.]	ptg005314l	78.218	4.6E-47	164	94
BCW92938.1_MAG:_elongation_factor_Tu_[Thermoanaerobaculum_sp.]	ptg002214l	66.91	7.16E-170	530	99
BCW92937.1_MAG:_elongation_factor_G_2_[Thermoanaerobaculum_sp.]	ptg004438l	46.328	1.43E-178	569	99
BCW92936.1_MAG:_30S_ribosomal_protein_S7_[Thermoanaerobaculum_sp.]	ptg003722l	47.742	3.52E-46	164	99
BCW92935.1_MAG:_30S_ribosomal_protein_S12_[Thermoanaerobaculum_sp.]	ptg003722l	78.632	5.09E-56	191	88
BCW92934.1_MAG:_hypothetical_protein_KatS3mg007_0828_[Thermoanaerobaculum_sp.]	ptg002723l	48.086	2.4E-172	548	95
BCW92933.1_MAG:_hypothetical_protein_KatS3mg007_0827_[Thermoanaerobaculum_sp.]	ptg002278l	59.195	0	832	96
BCW92932.1_MAG:_DNA-directed_RNA_polymerase_subunit_beta_[Thermoanaerobaculum_sp.]	ptg002278l	51.339	0	1288	95
BCW92931.1_MAG:_50S_ribosomal_protein_L7/L12_[Thermoanaerobaculum_sp.]	ptg002214l	59.223	2.51E-18	83.2	80
BCW92930.1_MAG:_50S_ribosomal_protein_L10_[Thermoanaerobaculum_sp.]	ptg002278l	34.459	1.07E-18	85.9	85
BCW92929.1_MAG:_50S_ribosomal_protein_L1_[Thermoanaerobaculum_sp.]	ptg002723l	53.604	2.13E-66	225	97
BCW92928.1_MAG:_50S_ribosomal_protein_L11_[Thermoanaerobaculum_sp.]	ptg002214l	66.429	1.06E-56	193	99
BCW92927.1_MAG:_transcription_termination/antitermination_protein_NusG_[Thermoanaerobaculum_sp.]	ptg002214l	54.696	4.68E-50	176	99
BCW92924.1_MAG:_elongation_factor_Tu_[Thermoanaerobaculum_sp.]	ptg002214l	66.91	7.16E-170	530	99
BCW92923.1_MAG:_23S_rRNA_(guanosine-2'-O-)-methyltransferase_RlmB_[Thermoanaerobaculum_sp.]	ptg005487l	30.165	1.07E-10	64.7	98
BCW92922.1_MAG:_bifunctional_enzyme_IspD/IspF_[Thermoanaerobaculum_sp.]	ptg004256l	48.276	1.08E-36	145	76
BCW92921.1_MAG:_ribosome-recycling_factor_[Thermoanaerobaculum_sp.]	ptg004895l	49.701	9.93E-47	166	91
BCW92920.1_MAG:_uridylate_kinase_[Thermoanaerobaculum_sp.]	ptg002436l	53.39	9.46E-67	227	96
BCW92919.1_MAG:_elongation_factor_Ts_[Thermoanaerobaculum_sp.]	ptg005656l	41.722	5.29E-32	128	100
BCW92918.1_MAG:_30S_ribosomal_protein_S2_[Thermoanaerobaculum_sp.]	ptg004895l	54.867	4.92E-80	266	82
BCW92917.1_MAG:_30S_ribosomal_protein_S9_[Thermoanaerobaculum_sp.]	ptg000819l	61.6	2.7E-37	137	98
BCW92916.1_MAG:_50S_ribosomal_protein_L13_[Thermoanaerobaculum_sp.]	ptg000819l	56.693	1.54E-42	152	88
BCW92915.1_MAG:_cation_transporter_[Thermoanaerobaculum_sp.]	ptg004798l	28.654	8.37E-86	309	98
BCW92914.1_MAG:_RND_transporter_[Thermoanaerobaculum_sp.]	ptg000732l	26.923	3.46E-18	89.7	65
BCW92913.1_MAG:_hypothetical_protein_KatS3mg007_0807_[Thermoanaerobaculum_sp.]	ptg003098l	33.333	1.52E-12	73.9	34
BCW92911.1_MAG:_hypothetical_protein_KatS3mg007_0805_[Thermoanaerobaculum_sp.]	ptg001693l	45.433	1.49E-113	369	100
BCW92910.1_MAG:_hydroxypyruvate_reductase_[Thermoanaerobaculum_sp.]	ptg003603l	43.939	2.76E-62	216	88
BCW92909.1_MAG:_phosphoserine_aminotransferase_[Thermoanaerobaculum_sp.]	ptg005318l	45.152	2.91E-102	334	99
BCW92907.1_MAG:_thioesterase_[Thermoanaerobaculum_sp.]	ptg003942l	51.316	3.1E-48	169	99
BCW92902.1_MAG:_hypothetical_protein_KatS3mg007_0796_[Thermoanaerobaculum_sp.]	ptg006032l	22.42	0.000000298	58.2	38
BCW92901.1_MAG:_tryptophan_synthase_alpha_chain_[Thermoanaerobaculum_sp.]	ptg004981l	36.364	1.43E-31	126	82
BCW92900.1_MAG:_tryptophan_synthase_beta_chain_[Thermoanaerobaculum_sp.]	ptg004214l	56.298	7.82E-134	426	98
BCW92899.1_MAG:_N-(5'-phosphoribosyl)anthranilate_isomerase_[Thermoanaerobaculum_sp.]	ptg003967l	38.806	1.47E-20	92.8	91

BCW92898.1_MAG:_indole-3-glycerol_phosphate_synthase_[Thermoanaerobaculum_sp.]	ptg004054l	38.961	2.91E-40	151	89
BCW92897.1_MAG:_anthranilate_phosphoribosyltransferase_[Thermoanaerobaculum_sp.]	ptg004652l	42.188	1.17E-31	129	75
BCW92896.1_MAG:_glutamine_amidotransferase_[Thermoanaerobaculum_sp.]	ptg004525l	54.922	2.32E-61	209	98
BCW92895.1_MAG:_anthranilate_synthase_component_I_[Thermoanaerobaculum_sp.]	ptg002760l	42.339	1.55E-95	320	99
BCW92894.1_MAG:_histidine_biosynthesis_bifunctional_protein_HisIE_[Thermoanaerobaculum_sp.]	ptg004372l	32.335	5.31E-15	76.6	80
BCW92893.1_MAG:_imidazole_glycerol_phosphate_synthase_subunit_HisF_[Thermoanaerobaculum_sp.]	ptg002534l	49.412	4.31E-50	179	97
BCW92892.1_MAG:_1-(5-phosphoribosyl)-5-[(5-phosphoribosylamino)_methylideneamino]_imidazole-4-carboxamide_isomerase_[Thermoanaerobaculum_sp.]	ptg002534l	33.913	1.01E-35	137	93
BCW92891.1_MAG:_imidazole_glycerol_phosphate_synthase_subunit_HisH_[Thermoanaerobaculum_sp.]	ptg004372l	31.795	1.44E-21	95.1	97
BCW92890.1_MAG:_imidazoleglycerol-phosphate_dehydratase_[Thermoanaerobaculum_sp.]	ptg002031l	50.256	4.76E-44	159	98
BCW92889.1_MAG:_histidinol-phosphate_aminotransferase_[Thermoanaerobaculum_sp.]	ptg005435l	26.646	5.14E-16	83.2	88
BCW92888.1_MAG:_histidinol_dehydrogenase_[Thermoanaerobaculum_sp.]	ptg002031l	43.772	1.51E-47	179	66
BCW92887.1_MAG:_ATP_phosphoribosyltransferase_2_[Thermoanaerobaculum_sp.]	ptg005709l	29.717	8.82E-23	99.4	99
BCW92885.1_MAG:_shikimate_dehydrogenase_(NADP(+))_[Thermoanaerobaculum_sp.]	ptg003361l	29.012	6.65E-23	107	64
BCW92883.1_MAG:_hypothetical_protein_KatS3mg007_0777_[Thermoanaerobaculum_sp.]	ptg005299l	41.136	2.12E-154	500	97
BCW92882.1_MAG:_hypothetical_protein_KatS3mg007_0776_[Thermoanaerobaculum_sp.]	ptg005299l	41.408	4.26E-71	243	99
BCW92881.1_MAG:_cell_division_protein_Fic_[Thermoanaerobaculum_sp.]	ptg004254l	37.748	1.44E-54	198	74
BCW92880.1_MAG:_site-specific_DNA-methyltransferase_[Thermoanaerobaculum_sp.]	ptg005299l	41.077	1.91E-126	430	89
BCW92868.1_MAG:_hypothetical_protein_KatS3mg007_0762_[Thermoanaerobaculum_sp.]	ptg003586l	30.435	1.03E-10	64.3	63
BCW92867.1_MAG:_hypothetical_protein_KatS3mg007_0761_[Thermoanaerobaculum_sp.]	ptg004457l	27.586	7.62E-09	60.5	38
BCW92866.1_MAG:_hypothetical_protein_KatS3mg007_0760_[Thermoanaerobaculum_sp.]	ptg002021l	30.583	3.12E-09	62	67
BCW92864.1_MAG:_carbon-nitrogen_hydrolase_[Thermoanaerobaculum_sp.]	ptg004519l	30.556	1.72E-12	70.5	66
BCW92862.1_MAG:_hypothetical_protein_KatS3mg007_0756_[Thermoanaerobaculum_sp.]	ptg003586l	30.435	1.82E-10	63.5	63
BCW92861.1_MAG:_hypothetical_protein_KatS3mg007_0755_[Thermoanaerobaculum_sp.]	ptg004457l	27.434	4.7E-09	61.2	38
BCW92859.1_MAG:_phosphoribosylglycinamide_formyltransferase_[Thermoanaerobaculum_sp.]	ptg005227l	40.394	1.39E-36	139	94
BCW92858.1_MAG:_phosphoribosylformylglycinamidine_cyclo-ligase_[Thermoanaerobaculum_sp.]	ptg005227l	44.51	1.56E-82	276	97
BCW92856.1_MAG:_amidophosphoribosyltransferase_[Thermoanaerobaculum_sp.]	ptg000484l	37.815	2.69E-80	275	97
BCW92855.1_MAG:_phosphoribosylformylglycinamidine_synthase_subunit_PurL_[Thermoanaerobaculum_sp.]	ptg002878l	32.036	4.45E-85	298	96
BCW92854.1_MAG:_phosphoribosylformylglycinamidine_synthase_subunit_PurQ_[Thermoanaerobaculum_sp.]	ptg005375l	35.319	8.15E-32	126	93
BCW92852.1_MAG:_MBL_fold_metallo-hydrolase_[Thermoanaerobaculum_sp.]	ptg004509l	38.073	3.28E-41	154	85
BCW92851.1_MAG:_deoxyuridine_5'-triphosphate_nucleotidohydrolase_[Thermoanaerobaculum_sp.]	ptg002199l	40.441	4.14E-27	108	91
BCW92850.1_MAG:_peptidase_M16_[Thermoanaerobaculum_sp.]	ptg001828l	25.189	7.67E-28	120	94
BCW92849.1_MAG:_hypothetical_protein_KatS3mg007_0743_[Thermoanaerobaculum_sp.]	ptg004519l	25.926	3.48E-08	60.8	42
BCW92845.1_MAG:_two-component_sensor_histidine_kinase_[Thermoanaerobaculum_sp.]	ptg004779l	27.32	6.05E-26	115	80
BCW92844.1_MAG:_DNA-binding_response_regulator_[Thermoanaerobaculum_sp.]	ptg004779l	38.393	4.77E-35	136	85

BCW92838.1_MAG:_hypothetical_protein_KatS3mg007_0732_[Thermoanaerobaculum_sp.]	ptg000834l	22.917	2.4E-11	70.9	47
BCW92832.1_MAG:_type_II_secretion_system_ATPase_PulE_[Thermoanaerobaculum_sp.]	ptg000568l	42.505	2.47E-120	394	95
BCW92831.1_MAG:_type_II_secretion_system_protein_[Thermoanaerobaculum_sp.]	ptg005301l	31.085	2.99E-47	177	85
BCW92828.1_MAG:_DNA-directed_RNA_polymerase_sigma-70_factor_[Thermoanaerobaculum_sp.]	ptg004479l	33.14	7.62E-22	96.3	80
BCW92827.1_MAG:_3-deoxy-D-manno-octulosonic_acid_transferase_[Thermoanaerobaculum_sp.]	ptg001658l	36.461	5.09E-55	200	87
BCW92826.1_MAG:_tetraacyldisaccharide_4'-kinase_[Thermoanaerobaculum_sp.]	ptg004746l	34.058	2.11E-28	119	81
BCW92825.1_MAG:_ADP-heptose_synthase_[Thermoanaerobaculum_sp.]	ptg002916l	34.535	1.81E-24	108	97
BCW92824.1_MAG:_hypothetical_protein_KatS3mg007_0718_[Thermoanaerobaculum_sp.]	ptg002916l	42.667	1.87E-29	116	90
BCW92823.1_MAG:_lipopolysaccharide_heptosyltransferase_I_[Thermoanaerobaculum_sp.]	ptg004974l	30.484	1.13E-24	108	97
BCW92821.1_MAG:_type_IV-A_pilus_assembly_ATPase_PilB_[Thermoanaerobaculum_sp.]	ptg004468l	44.563	1.11E-133	434	98
BCW92820.1_MAG:_twitching_motility_protein_PilT_[Thermoanaerobaculum_sp.]	LG13	48.991	3.94E-97	320	93
BCW92819.1_MAG:_type_II_secretion_system_protein_F_[Thermoanaerobaculum_sp.]	ptg005301l	39.85	4.03E-88	295	99
BCW92818.1_MAG:_PAS_domain-containing_sensor_histidine_kinase_[Thermoanaerobaculum_sp.]	ptg003137l	30.279	5.22E-26	117	42
BCW92817.1_MAG:_acetoacetate_metabolism_regulatory_protein_AtoC_[Thermoanaerobaculum_sp.]	ptg002304l	41.485	2.08E-102	338	99
BCW92815.1_MAG:_UTP--glucose-1-phosphate_uridylyltransferase_[Thermoanaerobaculum_sp.]	ptg002505l	44.867	2.42E-71	242	90
BCW92814.1_MAG:_FmdB_family_transcriptional_regulator_[Thermoanaerobaculum_sp.]	ptg004942l	37.143	3.94E-08	52.4	72
BCW92810.1_MAG:_hypothetical_protein_KatS3mg007_0704_[Thermoanaerobaculum_sp.]	LG10	53.425	3.46E-14	75.1	30
BCW92804.1_MAG:_GTPase_HflX_[Thermoanaerobaculum_sp.]	ptg004355l	53.75	5.92E-48	149	77
BCW92802.1_MAG:_nicotinate-nucleotide_diphosphorylase_(carboxylating)_[Thermoanaerobaculum_sp.]	ptg004584l	46.403	7.5E-59	206	96
BCW92801.1_MAG:_L-aspartate_oxidase_[Thermoanaerobaculum_sp.]	ptg004479l	41.042	4.99E-93	314	91
BCW92794.1_MAG:_membrane_protein_[Thermoanaerobaculum_sp.]	ptg004708l	58.261	1.72E-53	144	64
BCW92793.1_MAG:_hypothetical_protein_KatS3mg007_0687_[Thermoanaerobaculum_sp.]	ptg003703l	28.007	3.57E-52	203	64
BCW92792.1_MAG:_hypothetical_protein_KatS3mg007_0686_[Thermoanaerobaculum_sp.]	ptg004986l	40.26	2.87E-12	65.9	59
BCW92791.1_MAG:_ABC_transporter_ATP-binding_protein_[Thermoanaerobaculum_sp.]	ptg004470l	34.783	3.6E-17	85.5	76
BCW92789.1_MAG:_peptide_methionine_sulfoxide_reductase_MsrA_[Thermoanaerobaculum_sp.]	ptg001224l	47.771	6.7E-41	157	44
BCW92788.1_MAG:_hypothetical_protein_KatS3mg007_0682_[Thermoanaerobaculum_sp.]	ptg004623l	43.825	1E-57	202	93
BCW92785.1_MAG:_ceramide_glucosyltransferase_[Thermoanaerobaculum_sp.]	ptg003511l	29.6	0.000000231	57	31
BCW92780.1_MAG:_hypothetical_protein_KatS3mg007_0674_[Thermoanaerobaculum_sp.]	ptg006032l	34.657	3.37E-39	156	55
BCW92778.1_MAG:_amino_acid_permease_[Thermoanaerobaculum_sp.]	ptg002867l	29.87	2.49E-23	107	96
BCW92777.1_MAG:_serine/threonine_protein_kinase_[Thermoanaerobaculum_sp.]	ptg004928l	36.395	1.47E-37	150	63
BCW92776.1_MAG:_sodium:proline_symporter_[Thermoanaerobaculum_sp.]	ptg001353l	41.436	5.67E-55	202	70
BCW92774.1_MAG:_hypothetical_protein_KatS3mg007_0668_[Thermoanaerobaculum_sp.]	LG04	37.811	3.09E-24	108	53
BCW92773.1_MAG:_peptidase_[Thermoanaerobaculum_sp.]	ptg004479l	37.188	8.49E-74	257	89
BCW92772.1_MAG:_two-component_system_response_regulator_[Thermoanaerobaculum_sp.]	ptg005035l	31.933	1.5E-49	181	94

BCW92771.1_MAG:_transcription_termination_factor_Rho_[Thermoanaerobaculum_sp.]	ptg004523l	48.199	1.09E-103	340	90
BCW92770.1_MAG:_hypothetical_protein_KatS3mg007_0664_[Thermoanaerobaculum_sp.]	ptg006032l	29.68	1.58E-12	75.1	33
BCW92768.1_MAG:_acetyl-CoA_acetyltransferase_[Thermoanaerobaculum_sp.]	ptg004745l	48.346	5.07E-98	323	99
BCW92765.1_MAG:_hypothetical_protein_KatS3mg007_0659_[Thermoanaerobaculum_sp.]	ptg003137l	27.519	2.65E-16	89	20
BCW92762.1_MAG:_hypothetical_protein_KatS3mg007_0656_[Thermoanaerobaculum_sp.]	ptg000819l	34.392	6.05E-28	121	42
BCW92760.1_MAG:_RNA_polymerase_sigma_factor_RpoE_[Thermoanaerobaculum_sp.]	ptg004479l	29.268	1.73E-21	95.5	79
BCW92758.1_MAG:_hypothetical_protein_KatS3mg007_0652_[Thermoanaerobaculum_sp.]	ptg001624l	32.787	2.91E-18	87.4	70
BCW92755.1_MAG:_NADH-quinone_oxidoreductase_subunit_N_[Thermoanaerobaculum_sp.]	ptg004143l	36.311	3.01E-51	191	71
BCW92754.1_MAG:_NADH:ubiquinone_oxidoreductase_subunit_M_[Thermoanaerobaculum_sp.]	ptg004143l	47.327	1.71E-122	400	94
BCW92750.1_MAG:_NADH:ubiquinone_oxidoreductase_subunit_J_[Thermoanaerobaculum_sp.]	ptg004143l	32.934	1.89E-09	59.7	90
BCW92749.1_MAG:_hypothetical_protein_KatS3mg007_0643_[Thermoanaerobaculum_sp.]	ptg004143l	30.841	0.0000022	50.4	57
BCW92748.1_MAG:_NADH-quinone_oxidoreductase_subunit_H_1_[Thermoanaerobaculum_sp.]	ptg005338l	48.339	6.81E-73	249	79
BCW92747.1_MAG:_NADH-quinone_oxidoreductase_subunit_D_1_[Thermoanaerobaculum_sp.]	ptg004143l	50.521	8.85E-122	390	96
BCW92746.1_MAG:_hypothetical_protein_KatS3mg007_0640_[Thermoanaerobaculum_sp.]	ptg005338l	33.654	2.66E-21	68.2	62
BCW92745.1_MAG:_hypothetical_protein_KatS3mg007_0639_[Thermoanaerobaculum_sp.]	ptg004143l	64.706	7.1E-57	194	84
BCW92744.1_MAG:_NADH-quinone_oxidoreductase_subunit_A_[Thermoanaerobaculum_sp.]	ptg004143l	41.593	6.99E-24	98.6	93
BCW92740.1_MAG:_aminotransferase_[Thermoanaerobaculum_sp.]	ptg004905l	29.923	1.32E-36	145	95
BCW92726.1_MAG:_putative_fluoride_ion_transporter_CrcB_[Thermoanaerobaculum_sp.]	ptg002728l	38.318	1.6E-12	66.2	85
BCW92725.1_MAG:_aconitate_hydratase_[Thermoanaerobaculum_sp.]	ptg002123l	30.82	8.09E-23	108	50
BCW92723.1_MAG:_hypothetical_protein_KatS3mg007_0617_[Thermoanaerobaculum_sp.]	ptg001025l	27.978	1.83E-20	102	38
BCW92722.1_MAG:_acetoacetate_metabolism_regulatory_protein_AtoC_[Thermoanaerobaculum_sp.]	ptg005150l	40.98	2.35E-99	330	96
BCW92721.1_MAG:_pseudouridine_synthase_[Thermoanaerobaculum_sp.]	ptg006031l	43.878	7.59E-70	239	91
BCW92720.1_MAG:_prolipoprotein_diacylglyceryl_transferase_[Thermoanaerobaculum_sp.]	ptg005689l	35.294	1.79E-08	58.5	72
BCW92719.1_MAG:_lipoprotein_signal_peptidase_[Thermoanaerobaculum_sp.]	ptg004827l	33.75	9.96E-10	46.6	69
BCW92718.1_MAG:_isoleucine--tRNA_ligase_[Thermoanaerobaculum_sp.]	ptg002304l	40.299	2.85E-127	429	83
BCW92715.1_MAG:_gliding_motility-associated_ABC_transporter_permease_subunit_GldF_[Thermoanaerobaculum_sp.]	ptg005110l	30.769	4.08E-15	78.2	99
BCW92714.1_MAG:_multidrug_ABC_transporter_ATP-binding_protein_[Thermoanaerobaculum_sp.]	ptg001353l	49.541	6.78E-65	225	68
BCW92711.1_MAG:_ornithine_carbamoyltransferase_[Thermoanaerobaculum_sp.]	ptg003673l	51.866	6.77E-86	285	86
BCW92709.1_MAG:_adenylosuccinate_lyase_[Thermoanaerobaculum_sp.]	ptg002345l	33.259	1.28E-54	201	94
BCW92708.1_MAG:_hypothetical_protein_KatS3mg007_0602_[Thermoanaerobaculum_sp.]	ptg005398l	45.968	9.63E-32	125	54
BCW92706.1_MAG:_hypothetical_protein_KatS3mg007_0600_[Thermoanaerobaculum_sp.]	ptg001925l	25.424	0.0000013	55.8	46
BCW92704.1_MAG:_hypothetical_protein_KatS3mg007_0598_[Thermoanaerobaculum_sp.]	ptg001925l	26.814	3.18E-11	71.6	34
BCW92703.1_MAG:_hypothetical_protein_KatS3mg007_0597_[Thermoanaerobaculum_sp.]	ptg000619l	30.556	4.05E-35	149	40

BCW92702.1_MAG:_adenylate_cyclase_[Thermoanaerobaculum_sp.]	ptg004476l	30.976	3.05E-25	114	53
BCW92700.1_MAG:_dephospho-CoA_kinase_[Thermoanaerobaculum_sp.]	ptg004510l	30.337	3.23E-14	74.7	81
BCW92699.1_MAG:_bifunctional_protein_FolD_2_[Thermoanaerobaculum_sp.]	ptg002453l	45.353	9.07E-54	192	86
BCW92698.1_MAG:_demethylmenaquinone_methyltransferase_[Thermoanaerobaculum_sp.]	ptg001571l	33.921	4.92E-30	121	94
BCW92694.1_MAG:_ATP-dependent_protease_[Thermoanaerobaculum_sp.]	ptg004355l	48.171	3.04E-135	436	97
BCW92690.1_MAG:_hypothetical_protein_KatS3mg007_0584_[Thermoanaerobaculum_sp.]	ptg002760l	33.544	5.3E-11	65.9	64
BCW92689.1_MAG:_metal-dependent_hydrolase_[Thermoanaerobaculum_sp.]	ptg005425l	26.728	8.95E-13	70.5	88
BCW92688.1_MAG:_TlyA_family_rRNA_(cytidine-2'-O)-methyltransferase_[Thermoanaerobaculum_sp.]	ptg004075l	45.161	7.47E-39	147	96
BCW92687.1_MAG:_farnesyl-diphosphate_synthase_[Thermoanaerobaculum_sp.]	ptg002867l	44.622	1.27E-57	202	85
BCW92685.1_MAG:_exodeoxyribonuclease_7_large_subunit_[Thermoanaerobaculum_sp.]	ptg003058l	40	8.25E-84	285	95
BCW92682.1_MAG:_hypothetical_protein_KatS3mg007_0576_[Thermoanaerobaculum_sp.]	ptg003112l	32.258	6E-11	67.8	42
BCW92681.1_MAG:_ribonuclease_Y_[Thermoanaerobaculum_sp.]	ptg005484l	53.659	5.71E-96	323	63
BCW92678.1_MAG:_ribosomal_RNA_large_subunit_methyltransferase_I_[Thermoanaerobaculum_sp.]	ptg002871l	31	9.47E-34	137	98
BCW92677.1_MAG:_putative_alpha-L-glutamate_ligase_[Thermoanaerobaculum_sp.]	ptg002658l	26.636	5.81E-12	70.1	72
BCW92676.1_MAG:_hypothetical_protein_KatS3mg007_0570_[Thermoanaerobaculum_sp.]	ptg002316l	44.444	1.34E-28	112	98
BCW92675.1_MAG:_hypothetical_protein_KatS3mg007_0569_[Thermoanaerobaculum_sp.]	ptg002909l	30.739	1.76E-15	85.1	33
BCW92674.1_MAG:_hypothetical_protein_KatS3mg007_0568_[Thermoanaerobaculum_sp.]	ptg002760l	36.434	5.73E-34	137	59
BCW92666.1_MAG:_hypothetical_protein_KatS3mg007_0560_[Thermoanaerobaculum_sp.]	ptg004085l	38.009	3.44E-26	110	94
BCW92665.1_MAG:_AMP-binding_protein_[Thermoanaerobaculum_sp.]	ptg002844l	27.016	4.72E-32	136	76
BCW92664.1_MAG:_tRNA_pseudouridine_synthase_A_[Thermoanaerobaculum_sp.]	ptg001238l	34.8	1.88E-38	146	95
BCW92663.1_MAG:_isoprenyl_transferase_[Thermoanaerobaculum_sp.]	ptg005274l	50.424	1.37E-76	256	89
BCW92662.1_MAG:_phosphatidate_cytidylyltransferase_[Thermoanaerobaculum_sp.]	ptg004895l	45.6	9.1E-22	98.2	44
BCW92661.1_MAG:_1-deoxy-D-xylulose_5-phosphate_reductoisomerase_[Thermoanaerobaculum_sp.]	ptg003090l	49.412	9.71E-85	285	88
BCW92660.1_MAG:_putative_zinc_metalloprotease_[Thermoanaerobaculum_sp.]	ptg002436l	33.017	8.37E-44	168	88
BCW92657.1_MAG:_ribulose-phosphate_3-epimerase_[Thermoanaerobaculum_sp.]	ptg004771l	47.867	8.68E-57	197	95
BCW92653.1_MAG:_hypothetical_protein_KatS3mg007_0547_[Thermoanaerobaculum_sp.]	ptg004256l	31.544	1.16E-08	57.8	64
BCW92651.1_MAG:_aminoglycoside_phosphotransferase_[Thermoanaerobaculum_sp.]	ptg004480l	30.137	1.59E-17	86.7	88
BCW92649.1_MAG:_dual-specificity_RNA_methyltransferase_RlmN_[Thermoanaerobaculum_sp.]	ptg002634l	40.872	6.61E-58	206	95
BCW92648.1_MAG:_hypothetical_protein_KatS3mg007_0542_[Thermoanaerobaculum_sp.]	ptg005689l	34.459	1.76E-12	70.1	60
BCW92647.1_MAG:_cell_division_ATP-binding_protein_FtsE_[Thermoanaerobaculum_sp.]	ptg004158l	38.914	3.2E-31	124	97
BCW92643.1_MAG:_protein_TonB_[Thermoanaerobaculum_sp.]	ptg005754l	41.791	0.000000199	55.1	28
BCW92640.1_MAG:_flagellar_motor_protein_MotA_[Thermoanaerobaculum_sp.]	ptg002166l	34.737	1.98E-28	115	84
BCW92639.1_MAG:_crossover_junction_endodeoxyribonuclease_RuvC_[Thermoanaerobaculum_sp.]	ptg005341l	33.083	4.93E-14	72	83
BCW92638.1_MAG:_putative_transcriptional_regulatory_protein_[Thermoanaerobaculum_sp.]	ptg003903l	47.083	4.21E-49	176	97
BCW92637.1_MAG:_hypothetical_protein_KatS3mg007_0531_[Thermoanaerobaculum_sp.]	ptg006032l	34.375	8.03E-41	167	31

BCW92635.1_MAG:_acetyl-coenzyme_A_carboxylase_carboxyl_transferase_subunit_beta_[Thermoanaerobaculum_sp.]	ptg000484l	53.64	5.09E-81	269	94
BCW92634.1_MAG:_hypothetical_protein_KatS3mg007_0528_[Thermoanaerobaculum_sp.]	ptg002477l	38.462	2.59E-13	67.8	85
BCW92633.1_MAG:_hypothetical_protein_KatS3mg007_0527_[Thermoanaerobaculum_sp.]	ptg003967l	26.02	3.15E-10	64.3	62
BCW92632.1_MAG:_deoxyguanosinetriphosphate_triphosphohydrolase-like_protein_[Thermoanaerobaculum_sp.]	ptg005746l	36.83	6.89E-58	209	94
BCW92631.1_MAG:_ribose_5-phosphate_isomerase_B_[Thermoanaerobaculum_sp.]	ptg005354l	44.681	1.15E-32	124	97
BCW92630.1_MAG:_serine_hydroxymethyltransferase_[Thermoanaerobaculum_sp.]	ptg000693l	57.143	2E-139	444	96
BCW92629.1_MAG:_glycosyl_transferase_[Thermoanaerobaculum_sp.]	ptg003431l	27.7	4.79E-15	78.2	81
BCW92625.1_MAG:_sigma-54-dependent_Fis_family_transcriptional_regulator_[Thermoanaerobaculum_sp.]	ptg004075l	38.478	6.28E-84	285	97
BCW92624.1_MAG:_PAS_domain-containing_sensor_histidine_kinase_[Thermoanaerobaculum_sp.]	ptg004026l	25.652	3.28E-16	87.4	32
BCW92622.1_MAG:_UDP-3-O-acyl-N-acetylglucosamine_deacetylase_[Thermoanaerobaculum_sp.]	ptg004510l	40.511	1.33E-54	194	87
BCW92621.1_MAG:_hypothetical_protein_KatS3mg007_0515_[Thermoanaerobaculum_sp.]	ptg003829l	34.146	3.97E-21	101	33
BCW92620.1_MAG:_SsrA-binding_protein_[Thermoanaerobaculum_sp.]	ptg004680l	48.966	1.5E-35	133	97
BCW92619.1_MAG:_hypothetical_protein_KatS3mg007_0513_[Thermoanaerobaculum_sp.]	ptg003498l	34.783	2.34E-16	79.3	75
BCW92618.1_MAG:_UvrABC_system_protein_A_[Thermoanaerobaculum_sp.]	ptg004916l	57.757	0	979	99
BCW92612.1_MAG:_hypothetical_protein_KatS3mg007_0506_[Thermoanaerobaculum_sp.]	ptg003483l	35.294	7.5E-12	66.2	66
BCW92609.1_MAG:_hypothetical_protein_KatS3mg007_0503_[Thermoanaerobaculum_sp.]	ptg004862l	29.839	2.08E-09	58.5	77
BCW92608.1_MAG:_hypothetical_protein_KatS3mg007_0502_[Thermoanaerobaculum_sp.]	ptg005110l	41.243	1.55E-28	125	30
BCW92607.1_MAG:_arginase_[Thermoanaerobaculum_sp.]	LG29	54.545	3.78E-12	70.5	74
BCW92605.1_MAG:_enoyl-[acyl-carrier-protein]_reductase_[Thermoanaerobaculum_sp.]	ptg004732l	30.196	4.54E-30	121	98
BCW92604.1_MAG:_elongation_factor_P_[Thermoanaerobaculum_sp.]	LG03	38.71	7.74E-30	118	100
BCW92600.1_MAG:_cysteine_synthase_[Thermoanaerobaculum_sp.]	ptg005554l	42.857	2.18E-58	206	96
BCW92599.1_MAG:_cystathionine_gamma-synthase_[Thermoanaerobaculum_sp.]	ptg004895l	42.973	1.38E-88	295	95
BCW92596.1_MAG:_dehydrogenase_[Thermoanaerobaculum_sp.]	ptg002634l	28.938	6.32E-14	75.9	92
BCW92595.1_MAG:_hypothetical_protein_KatS3mg007_0489_[Thermoanaerobaculum_sp.]	ptg005563l	32.277	1.62E-26	123	36
BCW92594.1_MAG:_glutamine--tRNA_ligase_[Thermoanaerobaculum_sp.]	ptg005010l	57.812	0	612	89
BCW92593.1_MAG:_glutamate--tRNA_ligase_[Thermoanaerobaculum_sp.]	ptg004724l	35.551	4.96E-90	304	99
BCW92590.1_MAG:_aminodeoxyfutalosine_synthase_[Thermoanaerobaculum_sp.]	ptg001571l	52.617	2.15E-109	355	94
BCW92589.1_MAG:_hypothetical_protein_KatS3mg007_0483_[Thermoanaerobaculum_sp.]	ptg005363l	29.586	3.26E-10	63.5	58
BCW92584.1_MAG:_molecular_chaperone_DnaK_[Thermoanaerobaculum_sp.]	ptg002047l	28.571	1.44E-19	98.6	49
BCW92581.1_MAG:_hypothetical_protein_KatS3mg007_0475_[Thermoanaerobaculum_sp.]	ptg003045l	37.013	8.57E-12	73.9	13
BCW92575.1_MAG:_long-chain-fatty-acid--CoA_ligase_[Thermoanaerobaculum_sp.]	ptg005087l	31.689	2.03E-53	198	97
BCW92573.1_MAG:_3-oxoacyl-[acyl-carrier-protein]_synthase_3_[Thermoanaerobaculum_sp.]	ptg003871l	29.129	2.43E-24	107	96
BCW92572.1_MAG:_beta-ketoacyl-ACP_reductase_[Thermoanaerobaculum_sp.]	ptg005905l	48.571	1.17E-48	175	81
BCW92571.1_MAG:_membrane_protein_[Thermoanaerobaculum_sp.]	ptg001925l	24.573	0.00000192	56.2	30

BCW92568.1_MAG:_hypothetical_protein_KatS3mg007_0462_[Thermoanaerobaculum_sp.]	ptg004457l	37.559	8.39E-10	67.4	18
BCW92560.1_MAG:_hypothetical_protein_KatS3mg007_0454_[Thermoanaerobaculum_sp.]	ptg003336l	32.976	8.57E-37	154	42
BCW92550.1_MAG:_nitrate_reductase_catalytic_subunit_[Thermoanaerobaculum_sp.]	ptg004306l	34.358	8.66E-114	383	95
BCW92546.1_MAG:_cytochrome_bc_complex_cytochrome_b_subunit_[Thermoanaerobaculum_sp.]	ptg001921l	43.204	3.16E-50	184	56
BCW92545.1_MAG:_cytochrome_b6_[Thermoanaerobaculum_sp.]	LG26	47.059	0.00000444	48.5	23
BCW92544.1_MAG:_acetoacetate_metabolism_regulatory_protein_AtoC_[Thermoanaerobaculum_sp.]	ptg003057l	40.222	7.58E-80	273	98
BCW92543.1_MAG:_hypothetical_protein_KatS3mg007_0437_[Thermoanaerobaculum_sp.]	ptg001025l	24.848	9.1E-18	88.6	90
BCW92540.1_MAG:_hypothetical_protein_KatS3mg007_0434_[Thermoanaerobaculum_sp.]	ptg005274l	34.602	5.53E-44	173	46
BCW92539.1_MAG:_ferrochelatase_2_[Thermoanaerobaculum_sp.]	ptg001995l	47.748	4.24E-105	342	91
BCW92537.1_MAG:_RNA_polymerase_sigma24_factor_[Thermoanaerobaculum_sp.]	ptg003441l	28.947	1.47E-13	71.6	95
BCW92536.1_MAG:_30S_ribosomal_protein_S1_[Thermoanaerobaculum_sp.]	ptg001025l	33.333	2.75E-46	176	85
BCW92535.1_MAG:_histidine_kinase_[Thermoanaerobaculum_sp.]	ptg004494l	32.766	1.51E-20	100	41
BCW92534.1_MAG:_DNA-binding_response_regulator_[Thermoanaerobaculum_sp.]	LG03	37.553	4.84E-43	158	99
BCW92533.1_MAG:_phosphate_import_ATP-binding_protein_PstB_[Thermoanaerobaculum_sp.]	ptg002567l	55.823	1.06E-89	293	96
BCW92532.1_MAG:_phosphate_transport_system_regulatory_protein_PhoU_[Thermoanaerobaculum_sp.]	ptg004075l	41.206	2.75E-34	132	88
BCW92531.1_MAG:_phosphate_import_ATP-binding_protein_PstB_[Thermoanaerobaculum_sp.]	ptg002567l	55.061	9.45E-86	281	98
BCW92530.1_MAG:_phosphate_transport_system_permease_protein_PstA_[Thermoanaerobaculum_sp.]	ptg002567l	63.359	2.93E-83	285	52
BCW92529.1_MAG:_hypothetical_protein_KatS3mg007_0423_[Thermoanaerobaculum_sp.]	ptg002567l	35.238	2.02E-52	124	63
BCW92528.1_MAG:_phosphate-binding_protein_PstS_[Thermoanaerobaculum_sp.]	ptg002567l	57.19	1.91E-111	358	95
BCW92526.1_MAG:_hypothetical_protein_KatS3mg007_0420_[Thermoanaerobaculum_sp.]	ptg001925l	23.06	8.49E-26	119	90
BCW92525.1_MAG:_glycosyl_transferase_family_1_[Thermoanaerobaculum_sp.]	ptg001909l	34.416	4.94E-13	75.1	37
BCW92524.1_MAG:_5'-nucleotidase_[Thermoanaerobaculum_sp.]	ptg002246l	25.812	1.1E-52	197	93
BCW92523.1_MAG:_methylmalonyl-CoA_mutase_[Thermoanaerobaculum_sp.]	LG30	50	1.21E-12	75.1	44
BCW92520.1_MAG:_ribonuclease_3_[Thermoanaerobaculum_sp.]	ptg003583l	47.059	2.37E-09	60.8	28
BCW92519.1_MAG:_UDP-N-acetylglucosamine_1-carboxyvinyltransferase_[Thermoanaerobaculum_sp.]	ptg005611l	49.758	1.27E-84	166	98
BCW92518.1_MAG:_hypothetical_protein_KatS3mg007_0412_[Thermoanaerobaculum_sp.]	ptg004532l	45.783	1.62E-28	127	22
BCW92517.1_MAG:_histidine_triad_nucleotide-binding_protein_[Thermoanaerobaculum_sp.]	ptg003220l	46.018	2.08E-29	114	100
BCW92516.1_MAG:_hypothetical_protein_KatS3mg007_0410_[Thermoanaerobaculum_sp.]	ptg004256l	35.714	1.92E-09	65.1	21
BCW92514.1_MAG:_carbon-nitrogen_hydrolase_[Thermoanaerobaculum_sp.]	ptg003299l	28.767	9.23E-20	93.2	93
BCW92513.1_MAG:_NH(3)-dependent_NAD(+)_synthetase_[Thermoanaerobaculum_sp.]	ptg002304l	35.242	1.03E-21	98.2	78
BCW92510.1_MAG:_hypothetical_protein_KatS3mg007_0404_[Thermoanaerobaculum_sp.]	ptg001658l	37.313	1.33E-09	57.8	57
BCW92509.1_MAG:_ribosomal_RNA_large_subunit_methyltransferase_H_[Thermoanaerobaculum_sp.]	LG02	37.079	0.000000238	52.8	54
BCW92508.1_MAG:_hypothetical_protein_KatS3mg007_0402_[Thermoanaerobaculum_sp.]	ptg005150l	37.778	5.6E-45	166	86
BCW92507.1_MAG:_ribosomal_silencing_factor_RsfS_[Thermoanaerobaculum_sp.]	ptg004055l	41.237	4.05E-12	65.1	77
BCW92506.1_MAG:_nicotinate-nicotinamide_nucleotide_adenylyltransferase_[Thermoanaerobaculum_sp.]	ptg001008l	30.233	8.14E-26	108	97

BCW92505.1_MAG:_GTPase_Obg_[Thermoanaerobaculum_sp.]	ptg002505l	47.879	2.36E-71	244	99
BCW92504.1_MAG:_50S_ribosomal_protein_L27_[Thermoanaerobaculum_sp.]	ptg003971l	59.722	7.56E-21	88.2	85
BCW92503.1_MAG:_50S_ribosomal_protein_L21_[Thermoanaerobaculum_sp.]	ptg001290l	45.631	5.69E-17	78.2	98
BCW92501.1_MAG:_cyclic_dehypoxanthine_futalosine_synthase_[Thermoanaerobaculum_sp.]	ptg002867l	50.852	1.47E-104	341	94
BCW92500.1_MAG:_chorismate_dehydratase_[Thermoanaerobaculum_sp.]	ptg005484l	36.199	7.45E-31	124	83
BCW92499.1_MAG:_transcription_termination_factor_Rho_[Thermoanaerobaculum_sp.]	ptg003762l	65.823	1.63E-176	552	85
BCW92498.1_MAG:_putative_GTP-binding_protein_EngB_[Thermoanaerobaculum_sp.]	ptg004532l	37.433	1.99E-30	120	93
BCW92497.1_MAG:_Lon_protease_[Thermoanaerobaculum_sp.]	ptg005527l	51.123	0	717	94
BCW92496.1_MAG:_ATP-dependent_Clp_protease_ATP-binding_subunit_ClpX_[Thermoanaerobaculum_sp.]	ptg004240l	59.5	1.68E-157	495	97
BCW92495.1_MAG:_ATP-dependent_Clp_protease_proteolytic_subunit_[Thermoanaerobaculum_sp.]	ptg005527l	60.938	3.61E-70	234	94
BCW92494.1_MAG:_trigger_factor_[Thermoanaerobaculum_sp.]	ptg005527l	29.24	9.11E-13	74.3	41
BCW92492.1_MAG:_glycosyl_transferase_family_2_[Thermoanaerobaculum_sp.]	ptg003625l	32.157	6.91E-20	92.4	97
BCW92491.1_MAG:_metallophosphoesterase_[Thermoanaerobaculum_sp.]	ptg005484l	44.444	1.99E-59	206	89
BCW92489.1_MAG:_cytochrome_bc_complex_cytochrome_b_subunit_[Thermoanaerobaculum_sp.]	ptg001921l	44.131	1.02E-46	174	60
BCW92487.1_MAG:_(Fe-S)-binding_protein_[Thermoanaerobaculum_sp.]	ptg005961l	27.027	0.000000021	60.1	50
BCW92486.1_MAG:_proton/sodium-glutamate_symport_protein_[Thermoanaerobaculum_sp.]	ptg001291l	33.172	1.51E-63	225	98
BCW92485.1_MAG:_putative_ribosome_biogenesis_GTPase_RsgA_[Thermoanaerobaculum_sp.]	ptg004147l	41.538	8.89E-58	204	83
BCW92484.1_MAG:_peptidase_[Thermoanaerobaculum_sp.]	ptg004479l	32.668	2E-53	196	86
BCW92483.1_MAG:_amidase_[Thermoanaerobaculum_sp.]	ptg003250l	31.902	6.87E-49	186	87
BCW92482.1_MAG:_glycerol-3-phosphate_1-O-acyltransferase_[Thermoanaerobaculum_sp.]	ptg005535l	27.151	5.06E-25	112	78
BCW92479.1_MAG:_chaperone_protein_ClpB_[Thermoanaerobaculum_sp.]	ptg002304l	52.429	0	777	99
BCW92477.1_MAG:_glycosyl_transferase_[Thermoanaerobaculum_sp.]	ptg004077l	29.644	3.03E-13	75.5	61
BCW92473.1_MAG:_tetrathionate_reductase_subunit_A_[Thermoanaerobaculum_sp.]	ptg003787l	33.948	2.73E-31	137	28
BCW92472.1_MAG:_dimethylsulfoxide_reductase,_chain_B_[Thermoanaerobaculum_sp.]	ptg002175l	39.103	4.9E-31	122	84
BCW92468.1_MAG:_two-component_sensor_histidine_kinase_[Thermoanaerobaculum_sp.]	ptg003137l	31.203	4.19E-26	115	54
BCW92467.1_MAG:_acetoacetate_metabolism_regulatory_protein_AtoC_[Thermoanaerobaculum_sp.]	ptg005150l	38.546	1.67E-89	301	98
BCW92466.1_MAG:_hypothetical_protein_KatS3mg007_0360_[Thermoanaerobaculum_sp.]	ptg005825l	49.485	3.26E-18	82.8	70
BCW92465.1_MAG:_hypothetical_protein_KatS3mg007_0359_[Thermoanaerobaculum_sp.]	ptg004256l	32.45	5.96E-08	60.5	21
BCW92462.1_MAG:_30S_ribosomal_protein_S21_[Thermoanaerobaculum_sp.]	ptg001715l	52.632	0.00000218	46.2	54
BCW92461.1_MAG:_endonuclease_MutS2_[Thermoanaerobaculum_sp.]	ptg001460l	29.13	7.36E-11	70.1	29
BCW92460.1_MAG:_DNA_primase_[Thermoanaerobaculum_sp.]	ptg004856l	33.257	3.05E-64	232	71
BCW92459.1_MAG:_RNA_polymerase_sigma_factor_RpoD_[Thermoanaerobaculum_sp.]	ptg002303l	41.949	8.49E-100	336	82
BCW92456.1_MAG:_YggS_family_pyridoxal_phosphate_enzyme_[Thermoanaerobaculum_sp.]	ptg005005l	44.348	1.78E-23	81.3	97
BCW92453.1_MAG:_hypothetical_protein_KatS3mg007_0347_[Thermoanaerobaculum_sp.]	ptg002453l	41.667	1.57E-24	114	32
BCW92452.1_MAG:_DNA_helicase_[Thermoanaerobaculum_sp.]	ptg005430l	37.017	1.12E-115	387	99

BCW92451.1_MAG:_glutamine--fructose-6-phosphate_aminotransferase_[isomerizing]_[Thermoanaerobaculum_sp.]	ptg002878l	44.286	1.15E-140	455	100
BCW92450.1_MAG:_hypothetical_protein_KatS3mg007_0344_[Thermoanaerobaculum_sp.]	ptg004075l	34.796	2.2E-49	180	99
BCW92449.1_MAG:_hypothetical_protein_KatS3mg007_0343_[Thermoanaerobaculum_sp.]	ptg004284l	30.032	8.58E-15	79	76
BCW92446.1_MAG:_recombination_protein_RecR_[Thermoanaerobaculum_sp.]	ptg003168l	45.312	1.06E-45	164	96
BCW92444.1_MAG:_hypothetical_protein_KatS3mg007_0338_[Thermoanaerobaculum_sp.]	ptg002760l	33.333	0.000000102	57.4	30
BCW92443.1_MAG:_tRNA-specific_adenosine_deaminase_[Thermoanaerobaculum_sp.]	ptg001690l	50	6.27E-28	112	78
BCW92434.1_MAG:_ABC_transporter_ATP-binding_protein_[Thermoanaerobaculum_sp.]	ptg002760l	35.938	2.65E-53	130	96
BCW92433.1_MAG:_ABC_transporter_permease_[Thermoanaerobaculum_sp.]	ptg002738l	26.797	1.89E-13	76.6	98
BCW92432.1_MAG:_tRNA_pseudouridine_synthase_A_[Thermoanaerobaculum_sp.]	ptg003585l	35.349	4.46E-30	122	80
BCW92430.1_MAG:_amino_acid_transporter_[Thermoanaerobaculum_sp.]	ptg002867l	27.273	2.62E-15	83.2	75
BCW92429.1_MAG:_sodium:neurotransmitter_symporter_family_protein_[Thermoanaerobaculum_sp.]	ptg003960l	32.587	5.67E-36	147	88
BCW92426.1_MAG:_signal_peptidase_I_[Thermoanaerobaculum_sp.]	ptg002902l	32.203	1.1E-17	76.3	77
BCW92425.1_MAG:_elongation_factor_4_[Thermoanaerobaculum_sp.]	ptg004662l	54.561	0	651	99
BCW92424.1_MAG:_tRNA_N6-adenosine_threonylcarbamoyltransferase_[Thermoanaerobaculum_sp.]	ptg005331l	40.125	5.81E-39	151	87
BCW92423.1_MAG:_methylthioribose-1-phosphate_isomerase_[Thermoanaerobaculum_sp.]	ptg004010l	43.466	7.17E-52	188	99
BCW92422.1_MAG:_glyceraldehyde-3-phosphate_dehydrogenase_[Thermoanaerobaculum_sp.]	ptg002223l	52.905	8.98E-103	334	97
BCW92421.1_MAG:_phosphoglycerate_kinase_[Thermoanaerobaculum_sp.]	ptg004779l	43.257	2.82E-90	301	99
BCW92420.1_MAG:_triosephosphate_isomerase_[Thermoanaerobaculum_sp.]	ptg005107l	43.318	2.52E-45	165	85
BCW92416.1_MAG:_N-acetyl-alpha-D-glucosaminyl_L-malate_synthase_BshA_[Thermoanaerobaculum_sp.]	ptg001909l	26.623	1.98E-18	91.3	80
BCW92415.1_MAG:_bacillithiol_biosynthesis_deacetylase_BshB1_[Thermoanaerobaculum_sp.]	ptg005204l	31.982	8.52E-10	62.4	90
BCW92414.1_MAG:_hypothetical_protein_KatS3mg007_0308_[Thermoanaerobaculum_sp.]	ptg004277l	28.662	1.16E-08	55.5	86
BCW92409.1_MAG:_ATP_synthase_epsilon_chain_[Thermoanaerobaculum_sp.]	ptg002658l	38.462	0.000000214	52	59
BCW92408.1_MAG:_ATP_synthase_subunit_beta_[Thermoanaerobaculum_sp.]	ptg001921l	68.421	0	581	95
BCW92407.1_MAG:_ATP_synthase_gamma_chain_[Thermoanaerobaculum_sp.]	ptg000372l	39.661	2.79E-59	207	99
BCW92406.1_MAG:_ATP_synthase_subunit_alpha_[Thermoanaerobaculum_sp.]	ptg001624l	62.02	0	613	97
BCW92400.1_MAG:_hypothetical_protein_KatS3mg007_0294_[Thermoanaerobaculum_sp.]	ptg003830l	22.109	0.00000246	53.5	82
BCW92399.1_MAG:_UDP-N-acetylmuramate:L-alanyl-gamma-D-glutamyl-meso-diaminopimelate_ligase_[Thermoanaerobaculum_sp.]	ptg004510l	28.621	6.05E-27	82.4	92
BCW92395.1_MAG:_hypothetical_protein_KatS3mg007_0289_[Thermoanaerobaculum_sp.]	ptg002942l	35.849	2.61E-12	65.5	87
BCW92392.1_MAG:_hypothetical_protein_KatS3mg007_0286_[Thermoanaerobaculum_sp.]	ptg005076l	24.011	0.00000573	55.1	47
BCW92391.1_MAG:_peroxidase_[Thermoanaerobaculum_sp.]	ptg002047l	49.162	2.6E-51	180	91
BCW92390.1_MAG:_divalent_metal_cation_transporter_MntH_[Thermoanaerobaculum_sp.]	LG10	54.545	1.29E-19	98.2	14
BCW92389.1_MAG:_hypothetical_protein_KatS3mg007_0283_[Thermoanaerobaculum_sp.]	ptg003980l	26.906	3.65E-10	68.2	21
BCW92386.1_MAG:_DNA_processing_protein_DprA_[Thermoanaerobaculum_sp.]	ptg003363l	43.369	9.68E-50	181	80
BCW92384.1_MAG:_DNA_topoisomerase_1_[Thermoanaerobaculum_sp.]	LG04	40.916	1.62E-154	501	85

BCW92383.1_MAG:_methylenetetrahydrofolate--tRNA-(uracil-5-)-methyltransferase_TrmFO_[Thermoanaerobaculum_sp.]	ptg004077l	41.429	0.000000565	56.2	15
BCW92382.1_MAG:_phosphohydrolase_[Thermoanaerobaculum_sp.]	ptg002902l	40.217	2.39E-28	75.9	83
BCW92380.1_MAG:_tyrosine_recombinase_XerC_[Thermoanaerobaculum_sp.]	ptg003090l	43.581	3.73E-64	222	93
BCW92378.1_MAG:_hypothetical_protein_KatS3mg007_0272_[Thermoanaerobaculum_sp.]	ptg003829l	38.211	7.3E-14	73.2	59
BCW92376.1_MAG:_ATP-dependent_protease_subunit_HslV_[Thermoanaerobaculum_sp.]	ptg002928l	55.429	8.81E-48	169	98
BCW92375.1_MAG:_ATP-dependent_protease_ATPase_subunit_HslU_[Thermoanaerobaculum_sp.]	ptg005430l	50.664	2.94E-126	406	100
BCW92373.1_MAG:_diaminopimelate_epimerase_[Thermoanaerobaculum_sp.]	ptg003364l	28.947	3.89E-15	79	74
BCW92371.1_MAG:_hypothetical_protein_KatS3mg007_0265_[Thermoanaerobaculum_sp.]	ptg005121l	30.131	8.08E-21	100	79
BCW92369.1_MAG:_short-chain_dehydrogenase_[Thermoanaerobaculum_sp.]	ptg005905l	32.677	9.11E-20	92	98
BCW92366.1_MAG:_LemA_family_protein_[Thermoanaerobaculum_sp.]	ptg005363l	55.172	6.26E-51	179	89
BCW92365.1_MAG:_hypothetical_protein_KatS3mg007_0259_[Thermoanaerobaculum_sp.]	ptg005195l	31.716	1.45E-25	113	67
BCW92364.1_MAG:_ABC_transporter_substrate-binding_protein_[Thermoanaerobaculum_sp.]	ptg003131l	27.103	3.34E-10	67	54
BCW92363.1_MAG:_serine--tRNA_ligase_[Thermoanaerobaculum_sp.]	ptg005746l	48.14	5.36E-113	368	99
BCW92359.1_MAG:_UDP-phosphate_galactose_phosphotransferase_[Thermoanaerobaculum_sp.]	ptg004085l	51.19	8.93E-44	169	35
BCW92356.1_MAG:_tyrosine--tRNA_ligase_[Thermoanaerobaculum_sp.]	ptg000819l	51	1.12E-134	429	98
BCW92353.1_MAG:_hypothetical_protein_KatS3mg007_0247_[Thermoanaerobaculum_sp.]	ptg005922l	35.547	4.29E-32	134	55
BCW92352.1_MAG:_FAD:protein_FMN_transferase_[Thermoanaerobaculum_sp.]	ptg004017l	28.571	1.92E-19	94	70
BCW92351.1_MAG:_hypothetical_protein_KatS3mg007_0245_[Thermoanaerobaculum_sp.]	ptg002573l	32.283	5.73E-08	55.1	71
BCW92350.1_MAG:_hypothetical_protein_KatS3mg007_0244_[Thermoanaerobaculum_sp.]	ptg003770l	26.455	2.17E-09	60.8	81
BCW92348.1_MAG:_ferrous_iron_transport_protein_B_[Thermoanaerobaculum_sp.]	ptg003831l	26.847	1.77E-37	154	83
BCW92347.1_MAG:_transcriptional_repressor_[Thermoanaerobaculum_sp.]	ptg005922l	37.931	1.91E-15	75.5	79
BCW92345.1_MAG:_chromosome_partitioning_protein_ParB_[Thermoanaerobaculum_sp.]	ptg001818l	43.379	2.97E-44	163	75
BCW92344.1_MAG:_chromosome_partitioning_protein_ParA_[Thermoanaerobaculum_sp.]	ptg000372l	48.75	7.71E-70	237	88
BCW92343.1_MAG:_hypothetical_protein_KatS3mg007_0237_[Thermoanaerobaculum_sp.]	ptg001818l	31.818	6.88E-09	58.5	55
BCW92342.1_MAG:_membrane_protein_insertase_YidC_[Thermoanaerobaculum_sp.]	ptg000372l	39.834	1.19E-48	184	48
BCW92341.1_MAG:_putative_membrane_protein_insertion_efficiency_factor_[Thermoanaerobaculum_sp.]	ptg000372l	54.286	2.13E-13	67	81
BCW92338.1_MAG:_chromosomal_replication_initiator_protein_DnaA_[Thermoanaerobaculum_sp.]	ptg000372l	51.592	1.21E-94	315	73
BCW92337.1_MAG:_DNA_polymerase_III_subunit_beta_[Thermoanaerobaculum_sp.]	ptg000372l	29.032	8.77E-35	139	99
BCW92336.1_MAG:_DNA_replication_and_repair_protein_RecF_[Thermoanaerobaculum_sp.]	ptg000372l	24.915	6.32E-17	86.7	73
BCW92335.1_MAG:_DNA_gyrase_subunit_B_[Thermoanaerobaculum_sp.]	ptg000372l	60.634	0	563	99
BCW92334.1_MAG:_DNA_gyrase_subunit_A_[Thermoanaerobaculum_sp.]	ptg003798l	46.495	0	684	98
BCW92333.1_MAG:_transaldolase_[Thermoanaerobaculum_sp.]	ptg002573l	27.615	1.22E-10	63.9	89
BCW92332.1_MAG:_pyridoxal_5'-phosphate_synthase_subunit_PdxS_[Thermoanaerobaculum_sp.]	LG29	77.778	3.44E-19	91.7	50

BCW92330.1_MAG:_hypothetical_protein_KatS3mg007_0224_[Thermoanaerobaculum_sp.]	ptg004214l	34.028	1.99E-10	64.3	55
BCW92328.1_MAG:_hypothetical_protein_KatS3mg007_0222_[Thermoanaerobaculum_sp.]	ptg002738l	34.066	7.23E-16	79.3	82
BCW92325.1_MAG:_glutamate_racemase_[Thermoanaerobaculum_sp.]	ptg004538l	30.198	3.69E-15	79.3	68
BCW92324.1_MAG:_ribonuclease_PH_[Thermoanaerobaculum_sp.]	ptg003928l	50.388	1.89E-40	127	73
BCW92320.1_MAG:_DNA_methyltransferase_[Thermoanaerobaculum_sp.]	ptg005254l	44.102	2.58E-138	453	86
BCW92319.1_MAG:_transcriptional_regulator_[Thermoanaerobaculum_sp.]	ptg005254l	39.459	2.92E-35	134	92
BCW92318.1_MAG:_hypothetical_protein_KatS3mg007_0212_[Thermoanaerobaculum_sp.]	ptg005254l	38.72	1.93E-64	238	99
BCW92315.1_MAG:_type_I_restriction_endonuclease_subunit_R_[Thermoanaerobaculum_sp.]	ptg005254l	35.886	6.71E-170	562	82
BCW92312.1_MAG:_2-amino-3-ketobutyrate_coenzyme_A_ligase_[Thermoanaerobaculum_sp.]	ptg005614l	36.364	7.57E-63	222	85
BCW92311.1_MAG:_DNA_polymerase_I,_thermostable_[Thermoanaerobaculum_sp.]	ptg003762l	50.254	4.85E-110	375	79
BCW92308.1_MAG:_selenocysteine-specific_translation_elongation_factor_[Thermoanaerobaculum_sp.]	ptg004856l	45.714	3.68E-61	225	58
BCW92307.1_MAG:_hypothetical_protein_KatS3mg007_0201_[Thermoanaerobaculum_sp.]	ptg003361l	32.45	0.000000334	53.5	71
BCW92304.1_MAG:_hypothetical_protein_KatS3mg007_0198_[Thermoanaerobaculum_sp.]	ptg003787l	44.068	1.79E-08	54.3	52
BCW92302.1_MAG:_flavin_prenyltransferase_UbiX_[Thermoanaerobaculum_sp.]	ptg001571l	36.41	1.92E-28	115	96
BCW92301.1_MAG:_4-hydroxybenzoate_octaprenyltransferase_[Thermoanaerobaculum_sp.]	ptg003903l	41.475	7.49E-29	119	70
BCW92300.1_MAG:_deoxyadenosine_kinase_[Thermoanaerobaculum_sp.]	ptg004203l	27.835	2.51E-10	63.2	79
BCW92299.1_MAG:_3-methyl-2-oxobutanoate_hydroxymethyltransferase_[Thermoanaerobaculum_sp.]	ptg002760l	45.312	7.23E-66	226	92
BCW92298.1_MAG:_pantothenate_synthetase_[Thermoanaerobaculum_sp.]	ptg002465l	44.815	7.73E-67	229	95
BCW92297.1_MAG:_aspartate_1-decarboxylase_[Thermoanaerobaculum_sp.]	ptg004018l	50.82	1.61E-29	115	95
BCW92295.1_MAG:_ATP-dependent_protease_[Thermoanaerobaculum_sp.]	ptg003304l	38.173	1.15E-154	503	99
BCW92292.1_MAG:_CDP-diacylglycerol--glycerol-3-phosphate_3-phosphatidyltransferase_[Thermoanaerobaculum_sp.]	ptg005527l	34.078	7.5E-17	80.9	96
BCW92290.1_MAG:_AMP-binding_protein_[Thermoanaerobaculum_sp.]	ptg005087l	30.769	1.58E-26	119	52
BCW92288.1_MAG:_divalent-cation_tolerance_protein_CutA_[Thermoanaerobaculum_sp.]	ptg002612l	39.773	3.25E-14	71.2	71
BCW92284.1_MAG:_acyl-CoA_dehydrogenase_[Thermoanaerobaculum_sp.]	ptg004179l	28.693	9.49E-36	143	87
BCW92283.1_MAG:_aminotransferase_[Thermoanaerobaculum_sp.]	ptg005129l	24.85	3.37E-15	81.6	82
BCW92281.1_MAG:_IS256_family_transposase_[Thermoanaerobaculum_sp.]	ptg004058l	25.714	5.36E-08	58.9	68
BCW92280.1_MAG:_LPS_biosynthesis_O-acetyl_transferase_[Thermoanaerobaculum_sp.]	ptg005770l	29.268	2.21E-08	55.8	80
BCW92279.1_MAG:_aminotransferase_[Thermoanaerobaculum_sp.]	ptg001690l	30.376	1.57E-41	159	98
BCW92278.1_MAG:_hypothetical_protein_KatS3mg007_0172_[Thermoanaerobaculum_sp.]	ptg001690l	31.579	0.000000017	58.9	44
BCW92277.1_MAG:_transposase_for_insertion_sequence_element_ISRM5_[Thermoanaerobaculum_sp.]	ptg004058l	25.714	0.000000101	58.2	68
BCW92276.1_MAG:_magnesium_transport_protein_CorA_[Thermoanaerobaculum_sp.]	ptg003762l	35.354	5.63E-13	73.9	29
BCW92275.1_MAG:_peptide_deformylase_[Thermoanaerobaculum_sp.]	ptg004467l	35.26	1.86E-25	106	87

BCW92274.1_MAG:_iron-sulfur_cluster_insertion_protein_ErpA_[Thermoanaerobaculum_sp.]	ptg005487l	51.402	1.4E-30	117	98
BCW92273.1_MAG:_thioredoxin-disulfide_reductase_[Thermoanaerobaculum_sp.]	ptg005689l	55.272	4.86E-94	308	99
BCW92271.1_MAG:_orotidine_5'-phosphate_decarboxylase_[Thermoanaerobaculum_sp.]	ptg001025l	53.757	1.1E-48	175	71
BCW92270.1_MAG:_dihydroorotate_dehydrogenase_B_(NAD(+)),_catalytic_subunit_[Thermoanaerobaculum_sp.]	ptg006047l	27.445	3.85E-14	76.6	97
BCW92269.1_MAG:_dihydroorotate_dehydrogenase_B_(NAD(+)),_electron_transfer_subunit_[Thermoanaerobaculum_sp.]	ptg004538l	28.481	0.000000202	55.5	54
BCW92267.1_MAG:_hypothetical_protein_KatS3mg007_0161_[Thermoanaerobaculum_sp.]	ptg005756l	30.952	2.02E-30	126	72
BCW92266.1_MAG:_phosphate_acetyltransferase_[Thermoanaerobaculum_sp.]	ptg004584l	33.121	1.37E-30	125	93
BCW92265.1_MAG:_phosphoribosylamine--glycine_ligase_[Thermoanaerobaculum_sp.]	ptg002175l	43.868	2.01E-95	317	98
BCW92264.1_MAG:_N5-carboxyaminoimidazole_ribonucleotide_mutase_[Thermoanaerobaculum_sp.]	ptg002871l	48.039	2.18E-15	76.3	62
BCW92260.1_MAG:_hypothetical_protein_KatS3mg007_0154_[Thermoanaerobaculum_sp.]	ptg001291l	54.286	1.83E-16	77	91
BCW92259.1_MAG:_hypothetical_protein_KatS3mg007_0153_[Thermoanaerobaculum_sp.]	LG31	60.759	1.07E-28	110	84
BCW92255.1_MAG:_DNA-binding_protein_HU-beta_[Thermoanaerobaculum_sp.]	ptg005527l	43.956	6.26E-14	68.9	96
BCW92254.1_MAG:_transcriptional_regulator_[Thermoanaerobaculum_sp.]	ptg005527l	46.809	2.82E-18	81.3	99
BCW92252.1_MAG:_ribonucleoside-diphosphate_reductase_subunit_alpha_[Thermoanaerobaculum_sp.]	ptg002475l	22.111	1.06E-20	102	61
BCW92250.1_MAG:_hypothetical_protein_KatS3mg007_0144_[Thermoanaerobaculum_sp.]	LG03	24.242	0.000000628	52.4	70
BCW92249.1_MAG:_tRNA_(N6-threonylcarbamoyladenosine(37)-N6)-methyltransferase_TrmO_[Thermoanaerobaculum_sp.]	ptg005550l	38.406	5.22E-19	85.9	85
BCW92248.1_MAG:_L-threonine_3-dehydrogenase_[Thermoanaerobaculum_sp.]	ptg005563l	27.686	1.52E-08	60.1	71
BCW92247.1_MAG:_hypothetical_protein_KatS3mg007_0141_[Thermoanaerobaculum_sp.]	ptg002381l	38.994	5.91E-22	102	50
BCW92245.1_MAG:_hypothetical_protein_KatS3mg007_0139_[Thermoanaerobaculum_sp.]	ptg002381l	43.75	3.48E-23	105	35
BCW92243.1_MAG:_hypothetical_protein_KatS3mg007_0137_[Thermoanaerobaculum_sp.]	ptg002381l	26.331	7.08E-18	89	95
BCW92239.1_MAG:_hypothetical_protein_KatS3mg007_0133_[Thermoanaerobaculum_sp.]	ptg002381l	42.188	1.09E-19	94.7	35
BCW92235.1_MAG:_hypothetical_protein_KatS3mg007_0129_[Thermoanaerobaculum_sp.]	ptg003671l	26.738	0.00000165	55.5	27
BCW92233.1_MAG:_short-chain_dehydrogenase_[Thermoanaerobaculum_sp.]	ptg004500l	34.247	1.13E-25	108	93
BCW92228.1_MAG:_peptidase_S8_[Thermoanaerobaculum_sp.]	ptg005110l	27.762	1.56E-25	119	63
BCW92226.1_MAG:_pyruvate_dehydrogenase_E1_component_[Thermoanaerobaculum_sp.]	ptg005844l	53.089	7.34E-157	513	57
BCW92225.1_MAG:_hypothetical_protein_KatS3mg007_0119_[Thermoanaerobaculum_sp.]	LG03	41.463	1.94E-11	64.7	51
BCW92224.1_MAG:_hypothetical_protein_KatS3mg007_0118_[Thermoanaerobaculum_sp.]	ptg004466l	28.862	3.13E-13	74.3	74
BCW92223.1_MAG:_hypothetical_protein_KatS3mg007_0117_[Thermoanaerobaculum_sp.]	ptg004466l	25.796	1.04E-14	79	92
BCW92222.1_MAG:_hypothetical_protein_KatS3mg007_0116_[Thermoanaerobaculum_sp.]	ptg003382l	34.891	4.23E-48	179	83
BCW92219.1_MAG:_membrane_protein_[Thermoanaerobaculum_sp.]	ptg001925l	23.017	2.34E-16	88.6	62
BCW92218.1_MAG:_hypothetical_protein_KatS3mg007_0112_[Thermoanaerobaculum_sp.]	ptg003213l	36.129	4.64E-21	95.9	59
BCW92215.1_MAG:_hypothetical_protein_KatS3mg007_0109_[Thermoanaerobaculum_sp.]	ptg004987l	21.581	5.29E-10	67.4	54
BCW92211.1_MAG:_hypothetical_protein_KatS3mg007_0105_[Thermoanaerobaculum_sp.]	ptg005375l	47.166	0	1009	99

BCW92210.1_MAG:_glycerol-3-phosphate_ABC_transporter_ATP-binding_protein_[Thermoanaerobaculum_sp.]	ptg005435l	45.17	8.86E-95	312	99
BCW92209.1_MAG:_glycosyl_transferase_[Thermoanaerobaculum_sp.]	ptg004790l	25.705	5.15E-56	213	78
BCW92208.1_MAG:_beta-glucosidase_[Thermoanaerobaculum_sp.]	LG20	44.086	1.39E-16	86.7	34
BCW92205.1_MAG:_sugar_ABC_transporter_permease_[Thermoanaerobaculum_sp.]	ptg003163l	34.595	2.12E-14	76.6	66
BCW92204.1_MAG:_sugar_ABC_transporter_permease_[Thermoanaerobaculum_sp.]	ptg005435l	32.317	2.57E-17	85.9	55
BCW92201.1_MAG:_membrane_protein_[Thermoanaerobaculum_sp.]	ptg001925l	22.685	5.11E-15	84.3	82
BCW92200.1_MAG:_LacI_family_transcriptional_regulator_[Thermoanaerobaculum_sp.]	ptg005822l	32.278	6.31E-35	139	90
BCW92197.1_MAG:_hypothetical_protein_KatS3mg007_0091_[Thermoanaerobaculum_sp.]	ptg004779l	30.189	0.00000141	46.6	85
BCW92196.1_MAG:_hydrolase_[Thermoanaerobaculum_sp.]	ptg003304l	25.907	9.41E-08	56.2	71
BCW92195.1_MAG:_mannose-1-phosphate_guanylyltransferase_[Thermoanaerobaculum_sp.]	ptg004242l	36.49	2.32E-57	204	99
BCW92191.1_MAG:_ABC_transporter_[Thermoanaerobaculum_sp.]	ptg004055l	38.996	6.4E-48	179	83
BCW92187.1_MAG:_glycosyl_transferase_family_2_[Thermoanaerobaculum_sp.]	ptg003431l	31.25	3.74E-27	113	88
BCW92186.1_MAG:_hypothetical_protein_KatS3mg007_0080_[Thermoanaerobaculum_sp.]	ptg002270l	27.737	0.00000203	51.6	61
BCW92178.1_MAG:_spermidine_synthase_[Thermoanaerobaculum_sp.]	ptg002658l	30.796	9.22E-25	115	66
BCW92177.1_MAG:_glycosyl_transferase_family_1_[Thermoanaerobaculum_sp.]	ptg002534l	34.194	2.57E-18	90.5	43
BCW92176.1_MAG:_glycosyl_transferase_[Thermoanaerobaculum_sp.]	ptg002534l	27.843	5.55E-12	70.9	68
BCW92172.1_MAG:_glycosyl_transferase_family_1_[Thermoanaerobaculum_sp.]	ptg002316l	30.108	1.16E-28	122	92
BCW92171.1_MAG:_type_4_prepilin-like_proteins_leader_peptide-processing_enzyme_[Thermoanaerobaculum_sp.]	ptg002916l	37.037	1.72E-25	71.2	90
BCW92169.1_MAG:_sigma-54-dependent_Fis_family_transcriptional_regulator_[Thermoanaerobaculum_sp.]	ptg005150l	40.052	1.41E-79	271	90
BCW92168.1_MAG:_L-seryl-tRNA(Sec)_selenium_transferase_[Thermoanaerobaculum_sp.]	ptg005933l	44.798	1.52E-72	248	96
BCW92165.1_MAG:_ribosome-binding_ATPase_YchF_[Thermoanaerobaculum_sp.]	ptg002928l	38.63	7.05E-70	241	99
BCW92162.1_MAG:_methylmalonyl-CoA_carboxyltransferase_[Thermoanaerobaculum_sp.]	ptg003168l	62.472	0	582	97
BCW92161.1_MAG:_hypothetical_protein_KatS3mg007_0055_[Thermoanaerobaculum_sp.]	ptg004240l	47.033	2.62E-120	392	91
BCW92160.1_MAG:_hypothetical_protein_KatS3mg007_0054_[Thermoanaerobaculum_sp.]	ptg002223l	39.437	8.26E-08	54.3	43
BCW92159.1_MAG:_membrane_protein_[Thermoanaerobaculum_sp.]	ptg001925l	25.753	2.49E-09	65.9	29
BCW92158.1_MAG:_hypothetical_protein_KatS3mg007_0052_[Thermoanaerobaculum_sp.]	ptg000619l	32.203	1.86E-46	183	53
BCW92156.1_MAG:_hypothetical_protein_KatS3mg007_0050_[Thermoanaerobaculum_sp.]	ptg003471l	27.778	1.52E-12	74.3	60
BCW92154.1_MAG:_type_11_methyltransferase_[Thermoanaerobaculum_sp.]	ptg004186l	32.867	0.000000465	54.3	50
BCW92152.1_MAG:_CDP-abequose_synthase_[Thermoanaerobaculum_sp.]	ptg006001l	25.434	1.16E-20	96.3	97
BCW92151.1_MAG:_hypothetical_protein_KatS3mg007_0045_[Thermoanaerobaculum_sp.]	ptg002928l	38.596	3.66E-20	93.6	43
BCW92148.1_MAG:_threonine--tRNA_ligase_[Thermoanaerobaculum_sp.]	ptg004758l	43.553	1.85E-168	537	97
BCW92147.1_MAG:_50S_ribosomal_protein_L35_[Thermoanaerobaculum_sp.]	ptg001925l	50	2.09E-10	57.4	94
BCW92146.1_MAG:_50S_ribosomal_protein_L20_[Thermoanaerobaculum_sp.]	ptg001925l	53.333	5.5E-24	99	98
BCW92145.1_MAG:_phenylalanine--tRNA_ligase_alpha_subunit_[Thermoanaerobaculum_sp.]	ptg001925l	52.615	9.74E-109	352	94

BCW92144.1_MAG:_phenylalanine--tRNA_ligase_beta_subunit_[Thermoanaerobaculum_sp.]	ptg001925l	34.331	2.45E-83	291	91
BCW92142.1_MAG:_ATP-dependent_Clp_protease_ATP-binding_subunit_ClpX_[Thermoanaerobaculum_sp.]	ptg005527l	26.648	2.06E-20	97.4	89
BCW92140.1_MAG:_transcription-repair-coupling_factor_[Thermoanaerobaculum_sp.]	ptg004785l	41.814	0	614	81
BCW92139.1_MAG:_cysteine_desulfurase_IscS_[Thermoanaerobaculum_sp.]	ptg005265l	47.107	8.71E-100	328	94
BCW92137.1_MAG:_phosphoglucomutase_[Thermoanaerobaculum_sp.]	ptg003213l	25.737	1.49E-18	93.2	94
BCW92136.1_MAG:_enolase_[Thermoanaerobaculum_sp.]	ptg004466l	57.009	4.39E-141	449	99
BCW92134.1_MAG:_hypothetical_protein_KatS3mg007_0028_[Thermoanaerobaculum_sp.]	ptg002986l	51.397	1.04E-49	151	40
BCW92133.1_MAG:_peptidyl-prolyl_cis-trans_isomerase_[Thermoanaerobaculum_sp.]	ptg002976l	52.866	2.12E-51	180	82
BCW92131.1_MAG:_hypothetical_protein_KatS3mg007_0025_[Thermoanaerobaculum_sp.]	ptg003363l	40.678	4.74E-15	73.9	85
BCW92130.1_MAG:_anti-sigma_factor_antagonist_[Thermoanaerobaculum_sp.]	ptg004689l	35.417	2.3E-10	59.7	81
BCW92129.1_MAG:_hypothetical_protein_KatS3mg007_0023_[Thermoanaerobaculum_sp.]	ptg005341l	38.372	2.01E-11	65.1	48
BCW92128.1_MAG:_glutamate-1-semialdehyde_2,1-aminomutase_[Thermoanaerobaculum_sp.]	ptg005487l	30.147	2.23E-24	67.8	59
BCW92126.1_MAG:_hypothetical_protein_KatS3mg007_0020_[Thermoanaerobaculum_sp.]	ptg005484l	33.632	4.01E-26	115	48
BCW92125.1_MAG:_hypoxanthine_phosphoribosyltransferase_[Thermoanaerobaculum_sp.]	ptg000492l	27.273	2.52E-17	82	96
BCW92124.1_MAG:_ATP-dependent_zinc_metalloprotease_FtsH_[Thermoanaerobaculum_sp.]	ptg003039l	54.709	3.18E-154	495	79
BCW92123.1_MAG:_1-acyl-sn-glycerol-3-phosphate_acyltransferase_[Thermoanaerobaculum_sp.]	ptg002123l	36.129	4.23E-19	89.7	62
BCW92122.1_MAG:_hypothetical_protein_KatS3mg007_0016_[Thermoanaerobaculum_sp.]	ptg005265l	36.364	3.21E-13	67	98
BCW92121.1_MAG:_iron-sulfur_cluster_carrier_protein_[Thermoanaerobaculum_sp.]	ptg002214l	48.372	3.32E-65	227	62
BCW92118.1_MAG:_dihydropteroate_synthase_[Thermoanaerobaculum_sp.]	ptg003947l	49.805	8.09E-39	148	93
BCW92115.1_MAG:_phosphoglucosamine_mutase_[Thermoanaerobaculum_sp.]	ptg004534l	39.51	5.13E-35	137	82
BCW92114.1_MAG:_hypothetical_protein_KatS3mg007_0008_[Thermoanaerobaculum_sp.]	ptg005341l	38.938	4.56E-69	239	87
BCW92111.1_MAG:_hypothetical_protein_KatS3mg007_0005_[Thermoanaerobaculum_sp.]	ptg005074l	32.99	7.83E-09	55.5	69
BCW92110.1_MAG:_inorganic_pyrophosphatase_[Thermoanaerobaculum_sp.]	ptg004711l	40.964	4.25E-28	113	88
WP_081800112.1_SPFH_domain-containing_protein_[Thermoanaerobaculum_aquaticum]	ptg004708l	58.261	1.52E-53	144	64
WP_053335029.1_DUF971_domain-containing_protein_[Thermoanaerobaculum_aquaticum]	ptg005074l	31.959	0.000000021	54.3	69
WP_038046239.1_ParB/RepB/Spo0J_family_partition_protein_[Thermoanaerobaculum_aquaticum]	ptg001818l	42.922	4.08E-44	163	75
WP_235208737.1_energy_transducer_TonB_[Thermoanaerobaculum_aquaticum]	ptg005754l	37.805	4.52E-09	60.1	33
WP_053334870.1_ferrochelatase_[Thermoanaerobaculum_aquaticum]	ptg001995l	47.748	4.24E-105	342	91
WP_053335252.1_preprotein_translocase_subunit_YajC_[Thermoanaerobaculum_aquaticum]	ptg005746l	39.394	2.44E-10	58.9	67
WP_053334818.1_heavy_metal_sensor_histidine_kinase_[Thermoanaerobaculum_aquaticum]	ptg004779l	30.742	3.38E-25	113	58
WP_038050467.1_fluoride_efflux_transporter_CrcB_[Thermoanaerobaculum_aquaticum]	ptg002728l	42.391	1.85E-12	66.2	74
WP_038050192.1_signal_peptidase_II_[Thermoanaerobaculum_aquaticum]	ptg004827l	33.75	1.03E-09	46.2	69
WP_038049849.1_RIP_metalloprotease_RseP_[Thermoanaerobaculum_aquaticum]	ptg002436l	33.017	6.31E-44	168	88
WP_038049111.1_twin-arginine_translocase_subunit_TatC_[Thermoanaerobaculum_aquaticum]	ptg002534l	32.719	1.3E-27	114	86
WP_038046359.1_sodium-dependent_transporter_[Thermoanaerobaculum_aquaticum]	ptg003960l	33.333	6.77E-37	150	88

WP_081800106.1_NADH-quinone_oxidoreductase_subunit_M_[Thermoanaerobaculum_aquaticum]	ptg004143l	47.6	3.86E-123	402	94
WP_038050494.1_NADH-quinone_oxidoreductase_subunit_NuoH_[Thermoanaerobaculum_aquaticum]	ptg005338l	47.925	2.54E-67	233	78
WP_038050492.1_NADH-quinone_oxidoreductase_subunit_D_[Thermoanaerobaculum_aquaticum]	ptg004143l	50.521	6.31E-122	391	96
WP_038050175.1_NADH-quinone_oxidoreductase_subunit_NuoB_[Thermoanaerobaculum_aquaticum]	ptg004143l	42.857	3E-19	90.5	46
WP_038050164.1_NADH-quinone_oxidoreductase_subunit_NuoH_[Thermoanaerobaculum_aquaticum]	ptg004143l	39.941	5.77E-54	194	97
WP_235208678.1_tRNA_adenosine(34)_deaminase_TadA_[Thermoanaerobaculum_aquaticum]	ptg001690l	50	6.27E-28	112	78
WP_038050356.1_bifunctional_DNA-formamidopyrimidine_glycosylase/DNA-(apurinic_or_apyrimidinic_site)_lyase_[Thermoanaerobaculum_aquaticum]	ptg002199l	40.441	2.53E-53	189	99
WP_038046423.1_ATP-dependent_Clp_protease_ATP-binding_subunit_ClpX_[Thermoanaerobaculum_aquaticum]	ptg004240l	59.5	1.68E-157	495	97
WP_038046715.1_cytidine_deaminase_[Thermoanaerobaculum_aquaticum]	ptg000909l	51.2	6.04E-28	110	92
WP_038049552.1_cation-translocating_P-type_ATPase_[Thermoanaerobaculum_aquaticum]	ptg003880l	33.582	5.8E-48	187	75
WP_200867141.1_peptide_deformylase_[Thermoanaerobaculum_aquaticum]	ptg004467l	35	1.6E-25	106	91
WP_053335283.1_protein-L-isoaspartate(D-aspartate)_O-methyltransferase_[Thermoanaerobaculum_aquaticum]	ptg004256l	49.744	1.82E-38	144	87
WP_053334938.1_ribosomal_protein_S18-alanine_N-acetyltransferase_[Thermoanaerobaculum_aquaticum]	ptg003827l	34.615	2.92E-09	58.5	64
WP_038047116.1_protein-L-isoaspartate(D-aspartate)_O-methyltransferase_[Thermoanaerobaculum_aquaticum]	ptg004026l	49.302	3.55E-56	196	89
WP_038049868.1_crossover_junction_endodeoxyribonuclease_RuvC_[Thermoanaerobaculum_aquaticum]	ptg005341l	33.083	1.25E-10	62.4	83
WP_200867112.1_ribonuclease_HII_[Thermoanaerobaculum_aquaticum]	ptg002436l	42.938	5.16E-35	133	96
WP_081800108.1_transcription_termination_factor_Rho_[Thermoanaerobaculum_aquaticum]	ptg004523l	47.645	5.38E-103	338	90
WP_038049881.1_SsrA-binding_protein_SmpB_[Thermoanaerobaculum_aquaticum]	ptg004680l	49.655	3.2E-31	120	97
WP_038047613.1_GTPase_Era_[Thermoanaerobaculum_aquaticum]	ptg002902l	38.854	2.63E-22	100	51
WP_038046758.1_30S_ribosomal_protein_S10_[Thermoanaerobaculum_aquaticum]	ptg005314l	78.218	4.6E-47	164	94
WP_053334916.1_HAD-IA_family_hydrolase_[Thermoanaerobaculum_aquaticum]	ptg003304l	25.907	9.41E-08	56.2	71
WP_038046689.1_excinuclease_ABC_subunit_UvrB_[Thermoanaerobaculum_aquaticum]	ptg004926l	58.094	0	767	98
WP_152544008.1_ubiquinone/menaquinone_biosynthesis_methyltransferase_[Thermoanaerobaculum_aquaticum]	ptg004457l	35.242	2.28E-30	121	92
WP_038049848.1_1-deoxy-D-xylulose-5-phosphate_reductoisomerase_[Thermoanaerobaculum_aquaticum]	ptg003090l	49.708	1.59E-83	281	88
WP_038048811.1_SMC-Scp_complex_subunit_ScpB_[Thermoanaerobaculum_aquaticum]	ptg004457l	34.586	3.04E-20	90.9	73
WP_200867132.1_bifunctional_diaminohydroxyphosphoribosylaminopyrimidine_deaminase/5-amino-6-(5-phosphoribosylamino)uracil_reductase_RibD_[Thermoanaerobaculum_aquaticum]	ptg002928l	37.017	2.7E-49	181	98
WP_081799999.1_6,7-dimethyl-8-ribityllumazine_synthase_[Thermoanaerobaculum_aquaticum]	ptg004564l	41.727	2E-28	113	90
WP_200867148.1_lyase_family_protein,_partial_[Thermoanaerobaculum_aquaticum]	ptg002345l	32.143	1.02E-09	58.2	90
WP_053334729.1_ribosome_biogenesis_GTP-binding_protein_YihA/YsxC_[Thermoanaerobaculum_aquaticum]	ptg004532l	37.433	1.57E-30	120	93

WP_038050383.1_tRNA_guanosine(34)_transglycosylase_Tgt_[Thermoanaerobaculum_aquaticum]	ptg002867l	44.957	2.22E-90	300	93
WP_038049951.1_tRNA_(N6-threonylcarbamoyladenosine(37)-N6)-methyltransferase_TrmO_[Thermoanaerobaculum_aquaticum]	ptg005550l	38.06	3.73E-19	86.7	83
WP_038049788.1_tRNA_2-thiouridine(34)_synthase_MnmA_[Thermoanaerobaculum_aquaticum]	ptg003764l	42.744	4.65E-85	284	99
WP_038048756.1_glycine_cleavage_system_aminomethyltransferase_GcvT_[Thermoanaerobaculum_aquaticum]	ptg005299l	37.741	1.06E-78	266	98
WP_038048742.1_bifunctional_riboflavin_kinase/FAD_synthetase_[Thermoanaerobaculum_aquaticum]	ptg002304l	35.374	3.3E-38	147	92
WP_038048642.1_bifunctional_3,4-dihydroxy-2-butanone-4-phosphate_synthase/GTP_cyclohydrolase_II_[Thermoanaerobaculum_aquaticum]	ptg003075l	53.351	4.29E-122	393	94
WP_038048439.1_tRNA_preQ1(34)_S-adenosylmethionine_ribosyltransferase-isomerase_QueA_[Thermoanaerobaculum_aquaticum]	ptg004256l	41.399	5.25E-65	226	99
WP_038048048.1_elongation_factor_G_[Thermoanaerobaculum_aquaticum]	ptg002658l	35.043	1.62E-132	436	97
WP_038047534.1_ribosome_biogenesis_GTPase_Der_[Thermoanaerobaculum_aquaticum]	ptg003703l	41.475	1.46E-82	280	99
WP_038046969.1_tRNA_(adenosine(37)-N6)-dimethylallyltransferase_MiaA_[Thermoanaerobaculum_aquaticum]	ptg004997l	39.781	5.99E-56	198	89
WP_038046302.1_type_I_DNA_topoisomerase_[Thermoanaerobaculum_aquaticum]	LG04	40.855	7.96E-154	499	85
WP_053335015.1_NAD(P)-binding_protein_[Thermoanaerobaculum_aquaticum]	LG02	46.512	5.49E-08	60.1	15
WP_235208738.1_tryptophan_synthase_subunit_beta_[Thermoanaerobaculum_aquaticum]	ptg004214l	56.555	3.19E-134	427	98
WP_235208676.1_triose-phosphate_isomerase_[Thermoanaerobaculum_aquaticum]	ptg005107l	42.396	4.12E-44	162	85
WP_235208712.1_histidinol-phosphate_transaminase_[Thermoanaerobaculum_aquaticum]	ptg002505l	27.362	6.56E-25	109	87
WP_235208690.1_putative_molybdenum_carrier_protein_[Thermoanaerobaculum_aquaticum]	ptg001814l	48.718	2.95E-36	136	83
WP_053334702.1_ABC_transporter_C-terminal_domain-containing_protein_[Thermoanaerobaculum_aquaticum]	ptg005922l	36.22	1.42E-31	132	54
WP_053335080.1_NADH_dehydrogenase_(quinone)_subunit_D_[Thermoanaerobaculum_aquaticum]	ptg003419l	39.426	1.63E-92	314	68
WP_038048356.1_D-aminoacyl-tRNA_deacylase_[Thermoanaerobaculum_aquaticum]	ptg004026l	54.795	2.03E-36	135	97
WP_038048317.1_GH1_family_beta-glucosidase_[Thermoanaerobaculum_aquaticum]	LG20	45.161	2.47E-17	89	34
WP_200867157.1_bifunctional_phosphoribosyl-AMP_cyclohydrolase/phosphoribosyl-ATP_diphosphatase_HisIE_[Thermoanaerobaculum_aquaticum]	ptg004372l	32.335	5.16E-15	76.6	80
WP_038049847.1_isoprenyl_transferase_[Thermoanaerobaculum_aquaticum]	ptg005274l	50.424	1.37E-76	256	89
WP_053334969.1_tRNA_(N6-isopentenyl_adenosine(37)-C2)-methylthiotransferase_MiaB_[Thermoanaerobaculum_aquaticum]	ptg005772l	41.324	5.89E-103	340	94
WP_038046603.1_UDP-N-acetylmuramate--L-alanine_ligase_[Thermoanaerobaculum_aquaticum]	ptg004510l	37.324	3.69E-78	172	96
WP_038049852.1_ribulose-phosphate_3-epimerase_[Thermoanaerobaculum_aquaticum]	ptg004771l	47.867	8.68E-57	197	95
WP_053334696.1_glutamate_racemase_[Thermoanaerobaculum_aquaticum]	ptg004538l	29.703	2.37E-14	76.6	68
WP_038050440.1_tryptophan_synthase_subunit_alpha_[Thermoanaerobaculum_aquaticum]	ptg004981l	36.364	1.3E-31	127	82
WP_038050423.1_translation_elongation_factor_Ts_[Thermoanaerobaculum_aquaticum]	ptg005656l	41.391	1.95E-31	127	100
WP_200867135.1_acetyl-CoA_carboxylase,_carboxyltransferase_subunit_beta_[Thermoanaerobaculum_aquaticum]	ptg000484l	53.696	5.44E-79	263	92
WP_161685407.1_divergent_polysaccharide_deacetylase_family_protein_[Thermoanaerobaculum_aquaticum]	ptg002534l	36.111	1.63E-20	96.7	64
WP_161685546.1_PAS_domain-containing_protein_[Thermoanaerobaculum_aquaticum]	ptg005363l	33.594	7.22E-10	61.2	60

WP_053335257.1_nucleoside_kinase_[Thermoanaerobaculum_aquaticum]	ptg003625l	27.072	2.75E-08	60.8	32
WP_053334759.1_50S_ribosomal_protein_L4_[Thermoanaerobaculum_aquaticum]	ptg004509l	39.894	1.94E-29	118	90
WP_053334705.1_diaminopimelate_epimerase_[Thermoanaerobaculum_aquaticum]	ptg005641l	28.194	2.02E-12	70.9	72
WP_152543922.1_fused_MFS/spermidine_synthase_[Thermoanaerobaculum_aquaticum]	ptg002658l	30.796	1.29E-24	115	66
WP_053335050.1_HD_family_phosphohydrolase_[Thermoanaerobaculum_aquaticum]	ptg002928l	45.238	5.99E-12	73.2	13
WP_053335049.1_ABC_transporter_substrate-binding_protein/permease_[Thermoanaerobaculum_aquaticum]	ptg005526l	33.939	1.23E-11	71.2	34
WP_038050329.1_DUF3488_and_transglutaminase-like_domain-containing_protein_[Thermoanaerobaculum_aquaticum]	ptg004759l	37.594	2.06E-14	81.3	17
WP_038046234.1_chromosomal_replication_initiator_protein_DnaA_[Thermoanaerobaculum_aquaticum]	ptg000372l	51.592	1.28E-94	315	73
WP_161685464.1_excinuclease_ABC_subunit_UvrC_[Thermoanaerobaculum_aquaticum]	ptg005527l	36.181	2.71E-84	292	97
WP_053334836.1_rRNA_maturation_RNase_YbeY_[Thermoanaerobaculum_aquaticum]	ptg004790l	34	3.38E-12	66.2	70
WP_053334719.1_DNA_primase_[Thermoanaerobaculum_aquaticum]	ptg004856l	35.165	7.14E-64	231	61
WP_038050451.1_imidazole_glycerol_phosphate_synthase_subunit_HisF_[Thermoanaerobaculum_aquaticum]	ptg002534l	49.804	9.77E-52	184	97
WP_038050221.1_50S_ribosomal_protein_L10_[Thermoanaerobaculum_aquaticum]	ptg002278l	34.459	7.65E-19	86.3	85
WP_038047690.1_30S_ribosomal_protein_S16_[Thermoanaerobaculum_aquaticum]	ptg005431l	45	1.07E-16	75.9	100
WP_038046911.1_pantetheine-phosphate_adenylyltransferase_[Thermoanaerobaculum_aquaticum]	ptg004885l	49.664	1.41E-42	154	90
WP_038046838.1_tyrosine_phenol-lyase_[Thermoanaerobaculum_aquaticum]	ptg004361l	45.934	1.93E-132	425	99
WP_038046653.1_30S_ribosomal_protein_S18_[Thermoanaerobaculum_aquaticum]	ptg005487l	55.172	1.27E-15	72.8	76
WP_038046586.1_16S_rRNA_(cytosine(1402)-N(4))-methyltransferase_RsmH_[Thermoanaerobaculum_aquaticum]	ptg003204l	38.361	1.34E-47	174	98
WP_038046236.1_membrane_protein_insertion_efficiency_factor_YidD_[Thermoanaerobaculum_aquaticum]	ptg000372l	54.286	2.13E-13	67	81
WP_038046141.1_translation_initiation_factor_IF-3_[Thermoanaerobaculum_aquaticum]	ptg001558l	45.333	5.56E-36	135	84
WP_200867107.1_Rid_family_detoxifying_hydrolase_[Thermoanaerobaculum_aquaticum]	ptg004297l	29.323	1.56E-08	55.1	94
WP_038047684.1_50S_ribosomal_protein_L19_[Thermoanaerobaculum_aquaticum]	ptg005431l	54.955	3.79E-30	116	97
WP_038046812.1_translation_initiation_factor_IF-1_[Thermoanaerobaculum_aquaticum]	ptg004746l	75	1.56E-29	112	100
WP_038046432.1_50S_ribosomal_protein_L27_[Thermoanaerobaculum_aquaticum]	ptg003971l	59.722	7.56E-21	88.2	85
WP_038048783.1_lipid-A-disaccharide_synthase_[Thermoanaerobaculum_aquaticum]	ptg002436l	36.264	1.52E-44	115	85
WP_038046354.1_translation_elongation_factor_4_[Thermoanaerobaculum_aquaticum]	ptg004662l	54.561	0	651	99
WP_038046139.1_L-lysine_6-transaminase_[Thermoanaerobaculum_aquaticum]	ptg002126l	28.117	1.34E-32	135	89
WP_200867161.1_5'/3'-nucleotidase_SurE_[Thermoanaerobaculum_aquaticum]	ptg005398l	42.276	6.2E-48	173	92
WP_081800155.1_diguanylate_cyclase,_partial_[Thermoanaerobaculum_aquaticum]	ptg004532l	44.578	3.4E-27	123	22
WP_053335263.1_imidazoleglycerol-phosphate_dehydratase_HisB_[Thermoanaerobaculum_aquaticum]	ptg002031l	50.256	4.76E-44	159	98
WP_038050368.1_acetyl-CoA_carboxylase_biotin_carboxyl_carrier_protein_[Thermoanaerobaculum_aquaticum]	ptg004240l	50	5.27E-12	66.6	41
WP_038050053.1_7-cyano-7-deazaguanine_synthase_QueC_[Thermoanaerobaculum_aquaticum]	ptg004480l	32.444	3.66E-21	95.1	96

WP_038049915.1_3-oxoacyl-ACP_reductase_FabG_[Thermoanaerobaculum_aquaticum]	ptg005905l	48.571	6.58E-55	193	81
WP_038048017.1_ATP-dependent_DNA_helicase_RecG_[Thermoanaerobaculum_aquaticum]	ptg001995l	40.393	5.71E-118	393	93
WP_038047557.1_diguanylate_cyclase_[Thermoanaerobaculum_aquaticum]	ptg003673l	38.125	6.52E-18	93.6	17
WP_038046958.1_type_III_pantothenate_kinase_[Thermoanaerobaculum_aquaticum]	ptg002214l	30.047	3.74E-12	69.7	78
WP_038046761.1_50S_ribosomal_protein_L3_[Thermoanaerobaculum_aquaticum]	ptg001624l	42.647	6.44E-33	128	99
WP_038050167.1_nucleoside_triphosphate_pyrophosphohydrolase_[Thermoanaerobaculum_aquaticum]	ptg002030l	38.267	1.34E-52	187	97
WP_038046435.1_nicotinate-nucleotide_adenylyltransferase_[Thermoanaerobaculum_aquaticum]	ptg001008l	30.233	8.14E-26	108	97
WP_081799798.1_DNA-processing_protein_DprA_[Thermoanaerobaculum_aquaticum]	ptg003363l	43.369	1.45E-56	201	80
WP_200867130.1_thioredoxin-dependent_thiol_peroxidase_[Thermoanaerobaculum_aquaticum]	ptg004538l	47.321	3.58E-28	112	71
WP_200867122.1_aminomethyl-transferring_glycine_dehydrogenase_subunit_GcvPA_[Thermoanaerobaculum_aquaticum]	ptg005299l	36.216	9.49E-52	191	82
WP_200867111.1_YraN_family_protein_[Thermoanaerobaculum_aquaticum]	ptg002907l	38.136	3.49E-12	65.5	85
WP_200867092.1_RNA_polymerase_sigma_factor_RpoD_[Thermoanaerobaculum_aquaticum]	ptg002303l	41.737	3.43E-99	334	82
WP_038049550.1_RNA_3'-terminal_phosphate_cyclase_[Thermoanaerobaculum_aquaticum]	LG03	46.605	5.01E-76	258	95
WP_200867156.1_phosphoribosylanthranilate_isomerase_[Thermoanaerobaculum_aquaticum]	ptg003967l	39.801	8.21E-13	70.1	92
WP_200867145.1_NAD(P)H-dependent_oxidoreductase_subunit_E_[Thermoanaerobaculum_aquaticum]	ptg004740l	37.5	6.87E-08	54.7	50
WP_200867142.1_hypothetical_protein_[Thermoanaerobaculum_aquaticum]	ptg003199l	44.611	3.96E-83	227	98
WP_038049826.1_YifB_family_Mg_chelatase-like_AAA_ATPase_[Thermoanaerobaculum_aquaticum]	ptg004355l	48.171	7.55E-134	431	97
WP_038049301.1_zinc-binding_dehydrogenase_[Thermoanaerobaculum_aquaticum]	ptg004026l	26.216	8.11E-22	100	98
WP_053334848.1_SpoVG_family_protein_[Thermoanaerobaculum_aquaticum]	ptg003363l	41.667	0.00000022	51.2	50
WP_152544022.1_pyridoxal_phosphate-dependent_aminotransferase_[Thermoanaerobaculum_aquaticum]	ptg005129l	24.551	6.12E-15	80.9	81
WP_152543907.1_histone_deacetylase_[Thermoanaerobaculum_aquaticum]	ptg005274l	34.602	9.16E-44	172	46
WP_152543847.1_alanyl-tRNA_editing_protein_[Thermoanaerobaculum_aquaticum]	ptg002313l	30.732	1.15E-12	73.9	49
WP_038047377.1_lipopolysaccharide_heptosyltransferase_I_[Thermoanaerobaculum_aquaticum]	ptg004974l	31.529	3.88E-26	113	86
WP_053334883.1_molecular_chaperone_DnaK_[Thermoanaerobaculum_aquaticum]	ptg005922l	54.561	0	618	94
WP_038049133.1_molecular_chaperone_DnaK_[Thermoanaerobaculum_aquaticum]	ptg005922l	61.526	0	649	94
WP_053334996.1_30S_ribosomal_protein_S1_[Thermoanaerobaculum_aquaticum]	ptg002742l	43.596	3.22E-103	347	75
WP_038049541.1_3-hydroxyacyl-ACP_dehydratase_FabZ_[Thermoanaerobaculum_aquaticum]	ptg004895l	47.482	1.14E-36	136	96
WP_038047652.1_PD40_domain-containing_protein_[Thermoanaerobaculum_aquaticum]	ptg005886l	36.236	1.51E-53	193	91
WP_038047522.1_PD40_domain-containing_protein_[Thermoanaerobaculum_aquaticum]	ptg001604l	27.976	9.16E-15	80.9	54
WP_038046309.1_PD40_domain-containing_protein_[Thermoanaerobaculum_aquaticum]	ptg003980l	27.65	1.1E-09	66.6	21
WP_053335155.1_lytic_transglycosylase_domain-containing_protein_[Thermoanaerobaculum_aquaticum]	ptg004256l	31.544	1.16E-08	57.8	64

WP_038049952.1_chalcone_isomerase_family_protein_[Thermoanaerobaculum_aquaticum]	LG03	24.242	0.000000917	51.6	70
WP_038049840.1_hybrid_sensor_histidine_kinase/response_regulator_[Thermoanaerobaculum_aquaticum]	ptg002760l	36.434	8.84E-34	136	59
WP_038048762.1_aminomethyl-transferring_glycine_dehydrogenase_subunit_GcvPB_[Thermoanaerobaculum_aquaticum]	ptg005318l	43.35	2.53E-87	296	81
WP_038046300.1_methylenetetrahydrofolate--tRNA-(uracil(54)-C(5))-methyltransferase_(FADH(2)-oxidizing)_TrmFO_[Thermoanaerobaculum_aquaticum]	ptg004077l	41.429	0.000000556	56.2	15
WP_038050335.1_citrate_(Si)-synthase_[Thermoanaerobaculum_aquaticum]	ptg005518l	29.126	4.61E-30	127	89
WP_038049389.1_DNA_repair_protein_RadC_[Thermoanaerobaculum_aquaticum]	ptg002199l	34.545	1.86E-19	90.1	98
WP_038046120.1_chemotaxis-specific_protein-glutamate_methyltransferase_CheB_[Thermoanaerobaculum_aquaticum]	ptg005550l	38.095	1.36E-40	155	91
WP_038046115.1_holo-ACP_synthase_[Thermoanaerobaculum_aquaticum]	ptg003327l	36.441	1.71E-12	65.9	94
WP_081799800.1_Nramp_family_divalent_metal_transporter_[Thermoanaerobaculum_aquaticum]	LG10	55.682	6.14E-20	99	14
WP_161685556.1_TonB_family_protein_[Thermoanaerobaculum_aquaticum]	ptg006032l	29.68	1.75E-12	75.1	33
WP_161685442.1_uroporphyrinogen_decarboxylase_[Thermoanaerobaculum_aquaticum]	ptg005545l	39.466	2.2E-70	241	99
WP_161685434.1_RNA_polymerase_factor_sigma-54_[Thermoanaerobaculum_aquaticum]	ptg003787l	32.891	6.06E-55	203	73
WP_053335176.1_uridine_kinase_[Thermoanaerobaculum_aquaticum]	ptg001291l	49.038	1.69E-43	159	93
WP_038050286.1_thiol_peroxidase_[Thermoanaerobaculum_aquaticum]	ptg001693l	39.375	6.83E-30	118	90
WP_038047433.1_dUTP_diphosphatase_[Thermoanaerobaculum_aquaticum]	ptg002199l	41.176	2.79E-27	109	91
WP_038046553.1_cytochrome_b_[Thermoanaerobaculum_aquaticum]	ptg001921l	43.662	3.75E-46	172	60
WP_038049883.1_excinuclease_ABC_subunit_UvrA_[Thermoanaerobaculum_aquaticum]	ptg004916l	57.757	0	980	99
WP_038048106.1_prephenate_dehydratase_[Thermoanaerobaculum_aquaticum]	ptg005341l	38.938	2.85E-68	237	87
WP_038050318.1_flavodoxin-dependent_(E)-4-hydroxy-3-methylbut-2-enyl-diphosphate_synthase_[Thermoanaerobaculum_aquaticum]	ptg003703l	36.585	1.3E-53	196	98
WP_038050042.1_methionine--tRNA_ligase_[Thermoanaerobaculum_aquaticum]	ptg002214l	25.953	4.39E-61	225	99
WP_038049539.1_acyl-ACP--UDP-N-acetylglucosamine_O-acyltransferase_[Thermoanaerobaculum_aquaticum]	ptg004895l	39.113	2.87E-59	206	95
WP_038048479.1_Holliday_junction_branch_migration_DNA_helicase_RuvB,_partial_[Thermoanaerobaculum_aquaticum]	ptg005341l	58.657	2.6E-99	323	95
WP_038047686.1_tRNA_(guanosine(37)-N1)-methyltransferase_TrmD_[Thermoanaerobaculum_aquaticum]	ptg005017l	47.143	3.27E-60	208	81
WP_038047130.1_chorismate_synthase_[Thermoanaerobaculum_aquaticum]	ptg001005l	30.816	7.35E-18	89.7	82
WP_038046816.1_30S_ribosomal_protein_S11_[Thermoanaerobaculum_aquaticum]	ptg001245l	61.594	8.61E-44	155	100
WP_038046793.1_50S_ribosomal_protein_L5_[Thermoanaerobaculum_aquaticum]	ptg004509l	54.237	2.42E-63	214	98
WP_038046785.1_30S_ribosomal_protein_S17_[Thermoanaerobaculum_aquaticum]	ptg004509l	44.595	7.73E-14	68.2	85
WP_053335072.1_preprotein_translocase_subunit_SecA_[Thermoanaerobaculum_aquaticum]	ptg005005l	52.164	0	816	91
WP_038050426.1_30S_ribosomal_protein_S9_[Thermoanaerobaculum_aquaticum]	ptg000819l	61.6	2.7E-37	137	98
WP_038048024.1_2,3-bisphosphoglycerate-independent_phosphoglycerate_mutase_[Thermoanaerobaculum_aquaticum]	ptg002573l	25.707	1.76E-24	110	90
WP_038046818.1_30S_ribosomal_protein_S4_[Thermoanaerobaculum_aquaticum]	ptg004509l	55.024	1.04E-62	213	100
WP_038046225.1_DNA_gyrase_subunit_A_[Thermoanaerobaculum_aquaticum]	ptg003798l	46.612	0	685	98

WP_038050546.1_glutamine-hydrolyzing_GMP_synthase_[Thermoanaerobaculum_aquaticum]	ptg002345l	55.556	5.37E-173	544	99
WP_038047133.1_50S_ribosomal_protein_L31_[Thermoanaerobaculum_aquaticum]	ptg004959l	53.846	6.47E-20	84.7	96
WP_038046798.1_30S_ribosomal_protein_S8_[Thermoanaerobaculum_aquaticum]	ptg004509l	54.545	2.39E-33	126	100
WP_053334999.1_TAT-variant-translocated_molybdopterin_oxidoreductase_[Thermoanaerobaculum_aquaticum]	ptg001690l	55.2	9.02E-87	310	90
WP_038050022.1_CTP_synthase_[Thermoanaerobaculum_aquaticum]	ptg004256l	55.699	0	610	96
WP_038049962.1_dihydroorotate_dehydrogenase,_partial_[Thermoanaerobaculum_aquaticum]	ptg006047l	28.146	5.64E-13	72.8	99
WP_038046795.1_type_Z_30S_ribosomal_protein_S14_[Thermoanaerobaculum_aquaticum]	ptg005254l	68.182	5.21E-13	64.7	72
WP_038049257.1_thymidine_phosphorylase_[Thermoanaerobaculum_aquaticum]	ptg001353l	46.966	6.45E-74	255	86
WP_038046956.1_valine--tRNA_ligase_[Thermoanaerobaculum_aquaticum]	ptg001925l	44.383	0	769	96
WP_038046109.1_multifunctional_oxoglutarate_decarboxylase/oxoglutarate_dehydrogenase_thiamine_pyrophosphate-binding_subunit/dihydrolipoyllysine-residue_succinyltransferase_subunit_[Thermoanaerobaculum_aquaticum]	ptg004814l	48.014	0	739	88
WP_038046145.1_bifunctional_nicotinamidase/pyrazinamidase_[Thermoanaerobaculum_aquaticum]	ptg002634l	53.211	9.4E-45	124	99
WP_038047464.1_heavy_metal_response_regulator_transcription_factor_[Thermoanaerobaculum_aquaticum]	ptg004779l	38.393	1.97E-35	136	97
WP_152543950.1_NAD(+)/NADH_kinase_[Thermoanaerobaculum_aquaticum]	ptg002867l	35.065	5.98E-36	140	78
WP_152543852.1_adenylate_kinase_[Thermoanaerobaculum_aquaticum]	LG12	49.057	0.000000015	58.2	24
WP_038046652.1_50S_ribosomal_protein_L25/general_stress_protein_Ctc_[Thermoanaerobaculum_aquaticum]	ptg004982l	39.412	1.42E-23	102	74
WP_038046562.1_ribosome_silencing_factor_[Thermoanaerobaculum_aquaticum]	ptg004055l	41.237	3.61E-12	65.1	77
KAB2970045.1_MAG:_cadmium-translocating_P-type_ATPase_[Thermoanaerobaculia_bacterium]	ptg003880l	31.191	3.97E-34	144	73
KAB2970043.1_MAG:_Glu-tRNA(Gln)_amidotransferase_subunit_GatD_[Thermoanaerobaculia_bacterium]	ptg003090l	29.08	1.27E-19	96.7	69
KAB2970031.1_MAG:_amidohydrolase_family_protein_[Thermoanaerobaculia_bacterium]	ptg002699l	25.058	3.33E-18	92.8	80
KAB2970021.1_MAG:_phosphate_acetyltransferase_[Thermoanaerobaculia_bacterium]	ptg004584l	31.164	3.51E-23	103	86
KAB2970020.1_MAG:_acetate_kinase_[Thermoanaerobaculia_bacterium]	ptg004885l	48.649	3.71E-98	325	89
KAB2970017.1_MAG:_thioredoxin_domain-containing_protein_[Thermoanaerobaculia_bacterium]	ptg001291l	32.099	3.75E-16	53.5	78
KAB2970015.1_MAG:_P-II_family_nitrogen_regulator_[Thermoanaerobaculia_bacterium]	ptg003336l	39.623	6E-18	81.3	95
KAB2970014.1_MAG:_efflux_RND_transporter_permease_subunit_[Thermoanaerobaculia_bacterium]	ptg004670l	39.579	0	667	97
KAB2970013.1_MAG:_efflux_RND_transporter_periplasmic_adaptor_subunit_[Thermoanaerobaculia_bacterium]	ptg001766l	26.842	1.73E-24	110	88
KAB2970009.1_MAG:_DUF1738_domain-containing_protein_[Thermoanaerobaculia_bacterium]	ptg004058l	40.656	2.69E-60	211	92
KAB2970008.1_MAG:_single-stranded_DNA-binding_protein_[Thermoanaerobaculia_bacterium]	ptg005756l	37.5	8.19E-14	69.7	90
KAB2970004.1_MAG:_TraM_recognition_domain-containing_protein_[Thermoanaerobaculia_bacterium]	ptg004058l	27.321	4.89E-20	99.8	51
KAB2970003.1_MAG:_fluoride_efflux_transporter_CrcB_[Thermoanaerobaculia_bacterium]	ptg004525l	37.5	1.26E-08	55.1	63
KAB2970002.1_MAG:_P-type_conjugative_transfer_ATPase_TrbB_[Thermoanaerobaculia_bacterium]	ptg003168l	34.036	2.22E-29	122	92
KAB2969994.1_MAG:_P-type_conjugative_transfer_protein_TrbG_[Thermoanaerobaculia_bacterium]	ptg005137l	27.068	1.08E-08	60.1	41

KAB2969993.1_MAG:_TrbI/VirB10_family_protein_[Thermoanaerobaculia_bacterium]	ptg005137l	42.675	2.78E-32	119	45
KAB2969992.1_MAG:_ParA_family_protein_[Thermoanaerobaculia_bacterium]	ptg002223l	25.792	1.12E-08	58.9	85
KAB2969991.1_MAG:_ParB/RepB/Spo0J_family_partition_protein_[Thermoanaerobaculia_bacterium]	ptg001818l	40	1.15E-16	84.7	41
KAB2969987.1_MAG:_heavy_metal-responsive_transcriptional_regulator_[Thermoanaerobaculia_bacterium]	ptg001766l	37.405	2.13E-19	86.7	89
KAB2969986.1_MAG:_DNA_repair_protein_RadC_[Thermoanaerobaculia_bacterium]	ptg002199l	36.923	1.1E-17	83.2	71
KAB2969984.1_MAG:_ABC_transporter_ATP-binding_protein_[Thermoanaerobaculia_bacterium]	ptg001659l	45.274	5.78E-52	184	85
KAB2969931.1_MAG:_DNA-directed_RNA_polymerase_subunit_beta'_[Thermoanaerobaculia_bacterium]	ptg002723l	49.714	0	1332	98
KAB2969930.1_MAG:_50S_ribosomal_protein_L18_[Thermoanaerobaculia_bacterium]	ptg003734l	46.078	5.29E-23	95.9	85
KAB2969929.1_MAG:_adenylate_kinase_[Thermoanaerobaculia_bacterium]	LG12	50.943	0.000000238	53.5	29
KAB2969928.1_MAG:_elongation_factor_Tu,_partial_[Thermoanaerobaculia_bacterium]	ptg002214l	64.103	3.75E-11	58.2	98
KAB2969926.1_MAG:_transcription_termination/antitermination_protein_NusG_[Thermoanaerobaculia_bacterium]	ptg002214l	48.851	6.13E-55	190	95
KAB2969925.1_MAG:_50S_ribosomal_protein_L11_[Thermoanaerobaculia_bacterium]	ptg003726l	62.857	1.49E-39	144	100
KAB2969924.1_MAG:_50S_ribosomal_protein_L1_[Thermoanaerobaculia_bacterium]	ptg004158l	53.636	2.55E-61	211	94
KAB2969923.1_MAG:_50S_ribosomal_protein_L10_[Thermoanaerobaculia_bacterium]	ptg002278l	40.373	2.72E-26	107	92
KAB2969922.1_MAG:_50S_ribosomal_protein_L7/L12_[Thermoanaerobaculia_bacterium]	ptg002278l	53.175	1.54E-25	103	100
KAB2969921.1_MAG:_DNA-directed_RNA_polymerase_subunit_beta_[Thermoanaerobaculia_bacterium]	ptg002278l	49.853	0	1238	97
KAB2969920.1_MAG:_30S_ribosomal_protein_S12_[Thermoanaerobaculia_bacterium]	ptg003232l	72.951	1.49E-55	189	98
KAB2969919.1_MAG:_30S_ribosomal_protein_S7_[Thermoanaerobaculia_bacterium]	ptg003722l	47.097	8.01E-47	166	99
KAB2969918.1_MAG:_elongation_factor_G_[Thermoanaerobaculia_bacterium]	ptg004438l	46.198	0	590	98
KAB2969917.1_MAG:_elongation_factor_Tu_[Thermoanaerobaculia_bacterium]	ptg002214l	68.613	5.78E-174	542	99
KAB2969916.1_MAG:_30S_ribosomal_protein_S10_[Thermoanaerobaculia_bacterium]	ptg005314l	72.277	9.46E-44	154	96
KAB2969915.1_MAG:_50S_ribosomal_protein_L3_[Thermoanaerobaculia_bacterium]	ptg005314l	44.878	2.48E-49	175	98
KAB2969914.1_MAG:_50S_ribosomal_protein_L4_[Thermoanaerobaculia_bacterium]	ptg004509l	32.642	1.85E-27	112	92
KAB2969913.1_MAG:_50S_ribosomal_protein_L23_[Thermoanaerobaculia_bacterium]	ptg004509l	42.857	5.44E-15	72	95
KAB2969912.1_MAG:_50S_ribosomal_protein_L2_[Thermoanaerobaculia_bacterium]	ptg004509l	58.736	2.28E-90	296	98
KAB2969911.1_MAG:_30S_ribosomal_protein_S19_[Thermoanaerobaculia_bacterium]	ptg004509l	62.651	2.1E-29	113	85
KAB2969910.1_MAG:_50S_ribosomal_protein_L22_[Thermoanaerobaculia_bacterium]	ptg003734l	46.903	1.24E-22	94.7	92
KAB2969909.1_MAG:_30S_ribosomal_protein_S3_[Thermoanaerobaculia_bacterium]	ptg005314l	59.33	2.05E-76	253	96
KAB2969908.1_MAG:_50S_ribosomal_protein_L16_[Thermoanaerobaculia_bacterium]	ptg001624l	62.879	4E-43	154	95
KAB2969906.1_MAG:_30S_ribosomal_protein_S17_[Thermoanaerobaculia_bacterium]	ptg002047l	43.421	5.28E-17	77.4	82
KAB2969905.1_MAG:_50S_ribosomal_protein_L14_[Thermoanaerobaculia_bacterium]	ptg004509l	63.934	2.59E-43	154	100
KAB2969904.1_MAG:_50S_ribosomal_protein_L24_[Thermoanaerobaculia_bacterium]	ptg004509l	49.02	9.08E-26	103	95
KAB2969903.1_MAG:_50S_ribosomal_protein_L5_[Thermoanaerobaculia_bacterium]	ptg003734l	51.872	2.46E-63	216	85
KAB2969902.1_MAG:_type_Z_30S_ribosomal_protein_S14_[Thermoanaerobaculia_bacterium]	ptg005254l	68.085	6.25E-14	67.4	77

KAB2969901.1_MAG:_30S_ribosomal_protein_S8_[Thermoanaerobaculia_bacterium]	ptg005254l	46.875	1.89E-35	132	97
KAB2969900.1_MAG:_50S_ribosomal_protein_L6_[Thermoanaerobaculia_bacterium]	ptg004509l	42.614	2.96E-39	145	98
KAB2969899.1_MAG:_30S_ribosomal_protein_S5_[Thermoanaerobaculia_bacterium]	ptg004509l	54.93	6.53E-45	160	85
KAB2969898.1_MAG:_50S_ribosomal_protein_L30_[Thermoanaerobaculia_bacterium]	ptg004509l	58.182	6.59E-15	70.1	89
KAB2969897.1_MAG:_50S_ribosomal_protein_L15_[Thermoanaerobaculia_bacterium]	ptg005891l	46.259	3.05E-34	129	99
KAB2969896.1_MAG:_preprotein_translocase_subunit_SecY_[Thermoanaerobaculia_bacterium]	ptg004509l	44.685	2.83E-107	353	98
KAB2969895.1_MAG:_type_I_methionyl_aminopeptidase_[Thermoanaerobaculia_bacterium]	ptg002436l	45.528	2.04E-70	237	99
KAB2969894.1_MAG:_translation_initiation_factor_IF-1_[Thermoanaerobaculia_bacterium]	ptg004746l	70	1.16E-21	89.7	97
KAB2969893.1_MAG:_50S_ribosomal_protein_L36_[Thermoanaerobaculia_bacterium]	ptg004564l	70.27	7.32E-11	57	100
KAB2969892.1_MAG:_30S_ribosomal_protein_S13_[Thermoanaerobaculia_bacterium]	ptg004509l	59.649	3.1E-40	145	90
KAB2969891.1_MAG:_30S_ribosomal_protein_S11_[Thermoanaerobaculia_bacterium]	ptg001245l	69.231	1.3E-42	152	83
KAB2969890.1_MAG:_30S_ribosomal_protein_S4_[Thermoanaerobaculia_bacterium]	ptg004509l	51.905	2.99E-62	212	100
KAB2969889.1_MAG:_DNA-directed_RNA_polymerase_subunit_alpha_[Thermoanaerobaculia_bacterium]	ptg005964l	47.02	1.45E-80	270	90
KAB2969888.1_MAG:_50S_ribosomal_protein_L17_[Thermoanaerobaculia_bacterium]	ptg004509l	46.552	1.06E-25	107	56
KAB2969886.1_MAG:_class_I_SAM-dependent_methyltransferase_[Thermoanaerobaculia_bacterium]	ptg001062l	37.121	5.97E-20	91.7	59
KAB2969885.1_MAG:_elongation_factor_P_[Thermoanaerobaculia_bacterium]	LG03	39.779	9.26E-38	141	98
KAB2969884.1_MAG:_elongation_factor_G_[Thermoanaerobaculia_bacterium]	ptg002658l	35.502	6.23E-119	396	98
KAB2969883.1_MAG:_serine/threonine_protein_kinase,_partial_[Thermoanaerobaculia_bacterium]	ptg006032l	42.308	6.97E-54	194	82
KAB2969838.1_MAG:_sulfoxide_reductase_heme-binding_subunit_YedZ_[Thermoanaerobaculia_bacterium]	ptg002407l	41.765	8.38E-27	110	81
KAB2969837.1_MAG:_L,D-transpeptidase_family_protein_[Thermoanaerobaculia_bacterium]	ptg004862l	35.616	0.00000828	49.3	37
KAB2969829.1_MAG:_PTS_sugar_transporter_subunit_IIA_[Thermoanaerobaculia_bacterium]	ptg003603l	48.438	2.39E-13	72	30
KAB2969828.1_MAG:_protein-methionine-sulfoxide_reductase_catalytic_subunit_MsrP_[Thermoanaerobaculia_bacterium]	ptg002407l	54.15	1.83E-89	292	98
KAB2969820.1_MAG:_YceI_family_protein_[Thermoanaerobaculia_bacterium]	ptg002928l	26.923	4.09E-16	79	96
KAB2969819.1_MAG:_sigma-54-dependent_Fis_family_transcriptional_regulator_[Thermoanaerobaculia_bacterium]	ptg005150l	44.776	3.23E-79	275	60
KAB2969818.1_MAG:_sulfatase_[Thermoanaerobaculia_bacterium]	ptg005545l	24.048	4.42E-17	89	73
KAB2969766.1_MAG:_tyrosine-type_recombinase/integrase_[Thermoanaerobaculia_bacterium]	ptg005274l	36.025	1.17E-12	69.3	80
KAB2969765.1_MAG:_serine/threonine_protein_kinase_[Thermoanaerobaculia_bacterium]	ptg003585l	34.363	5.73E-31	127	70
KAB2969764.1_MAG:_SAM-dependent_DNA_methyltransferase_[Thermoanaerobaculia_bacterium]	ptg005254l	43.856	1.69E-142	463	88
KAB2969763.1_MAG:_hypothetical_protein_F9K18_00780_[Thermoanaerobaculia_bacterium]	ptg005254l	26.536	2.01E-29	125	80
KAB2969757.1_MAG:_type_I_restriction_endonuclease_subunit_R_[Thermoanaerobaculia_bacterium]	ptg005254l	39.057	3.02E-173	572	77
KAB2969755.1_MAG:_heavy_metal_translocating_P-type_ATPase_[Thermoanaerobaculia_bacterium]	ptg003880l	35	4.63E-62	233	60
KAB2969702.1_MAG:_carbonate_dehydratase_[Thermoanaerobaculia_bacterium]	LG10	50	9.61E-11	64.3	23

KAB2969670.1_MAG:_integron_integrase,_partial_[Thermoanaerobaculia_bacterium]	ptg004494l	46.259	2.95E-81	272	92
KAB2969635.1_MAG:_ATP-binding_cassette_domain-containing_protein_[Thermoanaerobaculia_bacterium]	ptg004680l	42.347	1.47E-44	161	93
KAB2969634.1_MAG:_cation-translocating_P-type_ATPase_[Thermoanaerobaculia_bacterium]	ptg003880l	28.485	8.8E-30	130	68
KAB2969630.1_MAG:_hypothetical_protein_F9K18_01070_[Thermoanaerobaculia_bacterium]	ptg002199l	36.923	6.67E-12	67.8	57
KAB2969626.1_MAG:_sigma-70_family_RNA_polymerase_sigma_factor_[Thermoanaerobaculia_bacterium]	ptg002465l	30.857	5.25E-11	63.9	91
KAB2969622.1_MAG:_P-II_family_nitrogen_regulator_[Thermoanaerobaculia_bacterium]	ptg003336l	35.043	1.03E-13	70.5	84
KAB2969621.1_MAG:_efflux_RND_transporter_permease_subunit_[Thermoanaerobaculia_bacterium]	ptg004670l	46.61	0	800	100
KAB2969620.1_MAG:_efflux_RND_transporter_periplasmic_adaptor_subunit_[Thermoanaerobaculia_bacterium]	ptg001766l	27.76	2.41E-23	106	76
KAB2969557.1_MAG:_dNTP_triphosphohydrolase_[Thermoanaerobaculia_bacterium]	ptg005213l	26.379	1.05E-30	129	82
KAB2969505.1_MAG:_nucleotide_pyrophosphatase_[Thermoanaerobaculia_bacterium]	ptg000619l	37.5	1.09E-08	63.2	10
KAB2969503.1_MAG:_glycosyltransferase_family_4_protein_[Thermoanaerobaculia_bacterium]	ptg002534l	30.583	6.17E-16	85.1	42
KAB2969501.1_MAG:_glycosyltransferase_family_4_protein_[Thermoanaerobaculia_bacterium]	ptg002634l	31.953	4.25E-13	75.1	40
KAB2969498.1_MAG:_glycosyltransferase_family_4_protein_[Thermoanaerobaculia_bacterium]	ptg003753l	26.457	1.89E-11	70.1	54
KAB2969497.1_MAG:_glycosyltransferase_family_2_protein_[Thermoanaerobaculia_bacterium]	ptg003625l	28.655	0.000000647	53.9	66
KAB2969493.1_MAG:_sulfotransferase_family_protein_[Thermoanaerobaculia_bacterium]	ptg000619l	39.894	3.4E-39	146	89
KAB2969458.1_MAG:_transcription_antitermination_factor_NusB_[Thermoanaerobaculia_bacterium]	ptg004564l	37.405	4.16E-18	83.6	86
KAB2969457.1_MAG:_SDR_family_oxidoreductase_[Thermoanaerobaculia_bacterium]	ptg005692l	35.606	2.98E-12	70.5	49
KAB2969456.1_MAG:_6,7-dimethyl-8-ribityllumazine_synthase_[Thermoanaerobaculia_bacterium]	ptg004564l	42.742	3.73E-18	84	78
KAB2969455.1_MAG:_MerR_family_transcriptional_regulator_[Thermoanaerobaculia_bacterium]	ptg001925l	38.158	4.36E-10	60.5	49
KAB2969454.1_MAG:_ribosome_biogenesis_GTPase_Der_[Thermoanaerobaculia_bacterium]	ptg003703l	38.051	7.81E-68	240	88
KAB2969451.1_MAG:_SDR_family_oxidoreductase_[Thermoanaerobaculia_bacterium]	ptg003980l	42.534	1.39E-46	169	89
KAB2969449.1_MAG:_glycosyltransferase_family_2_protein_[Thermoanaerobaculia_bacterium]	ptg002723l	27.746	4.49E-08	58.2	58
KAB2969448.1_MAG:_NADH-quinone_oxidoreductase_subunit_A_[Thermoanaerobaculia_bacterium]	ptg004143l	40.741	5.29E-13	67.8	62
KAB2969447.1_MAG:_NAD(P)H-dependent_oxidoreductase_subunit_E_[Thermoanaerobaculia_bacterium]	ptg004740l	31.579	0.000000639	52.8	47
KAB2969446.1_MAG:_NADH-quinone_oxidoreductase_subunit_NuoF_[Thermoanaerobaculia_bacterium]	ptg005451l	44.792	3.11E-86	291	89
KAB2969445.1_MAG:_molybdopterin-dependent_oxidoreductase_[Thermoanaerobaculia_bacterium]	ptg005338l	41.27	7.61E-37	116	36
KAB2969444.1_MAG:_NADH-quinone_oxidoreductase_subunit_NuoH_[Thermoanaerobaculia_bacterium]	ptg004143l	37.383	3.94E-61	215	94
KAB2969441.1_MAG:_NADH-quinone_oxidoreductase_subunit_L_[Thermoanaerobaculia_bacterium]	ptg004143l	36.533	1.89E-97	332	95
KAB2969440.1_MAG:_NADH-quinone_oxidoreductase_subunit_M,_partial_[Thermoanaerobaculia_bacterium]	ptg004143l	44.008	2.68E-99	332	93
KAB2969407.1_MAG:_ABC_transporter_ATP-binding_protein_[Thermoanaerobaculia_bacterium]	ptg001659l	49.541	9.98E-65	220	96
KAB2969405.1_MAG:_ABC_transporter_permease_[Thermoanaerobaculia_bacterium]	ptg004986l	26.4	1.01E-20	99	84

KAB2969404.1_MAG:_ABC_transporter_permease_[Thermoanaerobaculia_bacterium]	ptg004986l	28.767	1.38E-13	77	80
KAB2969403.1_MAG:_M48_family_metalloprotease_[Thermoanaerobaculia_bacterium]	ptg004885l	31.858	3.98E-26	117	43
KAB2969396.1_MAG:_NAD(P)-dependent_alcohol_dehydrogenase_[Thermoanaerobaculia_bacterium]	ptg000693l	32.78	6.59E-15	79.3	71
KAB2969334.1_MAG:_hypothetical_protein_F9K18_01500,_partial_[Thermoanaerobaculia_bacterium]	ptg002145l	52.381	1.75E-11	69.3	21
KAB2969333.1_MAG:_DEAD/DEAH_box_helicase_[Thermoanaerobaculia_bacterium]	ptg004186l	26.162	2.24E-27	124	67
KAB2969332.1_MAG:_SAM-dependent_DNA_methyltransferase_[Thermoanaerobaculia_bacterium]	ptg004186l	28.311	1.39E-46	178	81
KAB2969329.1_MAG:_HsdR_family_type_I_site-specific_deoxyribonuclease_[Thermoanaerobaculia_bacterium]	ptg000693l	24.452	3.95E-31	134	81
KAB2969326.1_MAG:_HNH_endonuclease_[Thermoanaerobaculia_bacterium]	ptg003045l	39.716	4.66E-20	94.7	43
KAB2969324.1_MAG:_tetratricopeptide_repeat_protein,_partial_[Thermoanaerobaculia_bacterium]	ptg005121l	25	0.000000798	55.8	40
KAB2969322.1_MAG:_co-chaperone_GroES_[Thermoanaerobaculia_bacterium]	ptg004085l	52.174	9.39E-25	99.8	95
KAB2969321.1_MAG:_chaperonin_GroEL_[Thermoanaerobaculia_bacterium]	ptg002031l	59.924	1.52E-178	562	96
KAB2969320.1_MAG:_sensor_domain-containing_diguanylate_cyclase_[Thermoanaerobaculia_bacterium]	LG04	41.358	7.62E-23	106	32
KAB2969319.1_MAG:_NADH:ubiquinone_oxidoreductase_[Thermoanaerobaculia_bacterium]	ptg005451l	33.579	2.96E-73	260	87
KAB2969318.1_MAG:_2Fe-2S_iron-sulfur_cluster_binding_domain-containing_protein_[Thermoanaerobaculia_bacterium]	ptg005338l	31.897	1.32E-18	77.8	71
KAB2969314.1_MAG:_HAMP_domain-containing_histidine_kinase_[Thermoanaerobaculia_bacterium]	ptg002909l	26.16	2.05E-23	110	37
KAB2969260.1_MAG:_NADP-dependent_oxidoreductase_[Thermoanaerobaculia_bacterium]	ptg001995l	45.614	4.98E-84	281	100
KAB2969248.1_MAG:_bifunctional_salicylyl-CoA_5-hydroxylase/oxidoreductase_[Thermoanaerobaculia_bacterium]	ptg005550l	52.174	6.97E-60	124	46
KAB2969244.1_MAG:_benzoate-CoA_ligase_family_protein_[Thermoanaerobaculia_bacterium]	ptg005087l	26.535	6.87E-35	143	93
KAB2969242.1_MAG:_enoyl-CoA_hydratase/isomerase_family_protein_[Thermoanaerobaculia_bacterium]	ptg002612l	39.216	2.25E-44	163	97
KAB2969241.1_MAG:_glycosyltransferase_[Thermoanaerobaculia_bacterium]	ptg003112l	37.391	2.26E-13	79.3	10
KAB2969202.1_MAG:_signal_peptidase_I_[Thermoanaerobaculia_bacterium]	ptg002902l	33.775	6.8E-15	70.1	64
KAB2969201.1_MAG:_helix-hairpin-helix_domain-containing_protein_[Thermoanaerobaculia_bacterium]	ptg000732l	42.623	3.67E-11	62.4	50
KAB2969199.1_MAG:_divalent-cation_tolerance_protein_CutA_[Thermoanaerobaculia_bacterium]	ptg002612l	40.426	1.65E-14	72.4	70
KAB2969198.1_MAG:_stress_response_translation_initiation_inhibitor_YciH_[Thermoanaerobaculia_bacterium]	ptg005341l	40.278	0.000000023	53.9	62
KAB2969196.1_MAG:_isocitrate_lyase_family_protein_[Thermoanaerobaculia_bacterium]	LG12	61.972	4.18E-19	97.1	17
KAB2969193.1_MAG:_redox-regulated_ATPase_YchF_[Thermoanaerobaculia_bacterium]	ptg004982l	43.014	2.38E-70	242	100
KAB2969148.1_MAG:_peptide_deformylase_[Thermoanaerobaculia_bacterium]	ptg004467l	40.645	2.77E-27	111	84
KAB2969146.1_MAG:_isoleucine--tRNA_ligase_[Thermoanaerobaculia_bacterium]	ptg002867l	38.917	0	763	98
KAB2969145.1_MAG:_phosphomannomutase/phosphoglucomutase_[Thermoanaerobaculia_bacterium]	ptg002505l	35.045	1.37E-64	229	97
KAB2969144.1_MAG:_phospholipid_carrier-dependent_glycosyltransferase_[Thermoanaerobaculia_bacterium]	ptg004500l	38.636	2.32E-15	83.2	27

KAB2969141.1_MAG:_gamma-glutamyltransferase_family_protein_[Thermoanaerobaculia_bacterium]	ptg001595l	29.11	7.31E-41	164	94
KAB2969140.1_MAG:_zinc_metallopeptidase_[Thermoanaerobaculia_bacterium]	ptg000693l	60.849	1.57E-60	208	88
KAB2969096.1_MAG:_SpoIIE_family_protein_phosphatase_[Thermoanaerobaculia_bacterium]	ptg004476l	27.079	6.3E-25	114	88
KAB2969095.1_MAG:_hypothetical_protein_F9K18_01870_[Thermoanaerobaculia_bacterium]	LG12	54.93	1.39E-17	89.4	17
KAB2969094.1_MAG:_AarF/ABC1/UbiB_kinase_family_protein_[Thermoanaerobaculia_bacterium]	ptg003583l	26.984	2.95E-43	170	75
KAB2969093.1_MAG:_S46_family_peptidase_[Thermoanaerobaculia_bacterium]	ptg002992l	34.938	1.61E-103	353	96
KAB2969090.1_MAG:_electron_transfer_flavoprotein-ubiquinone_oxidoreductase_[Thermoanaerobaculia_bacterium]	ptg005554l	37.9	2.97E-51	137	81
KAB2969089.1_MAG:_hypothetical_protein_F9K18_01840,_partial_[Thermoanaerobaculia_bacterium]	ptg000492l	32.157	8.88E-21	99	57
KAB2969048.1_MAG:_2-oxoglutarate_dehydrogenase_E1_component,_partial_[Thermoanaerobaculia_bacterium]	ptg004409l	43.355	1.84E-179	574	99
KAB2969042.1_MAG:_GAF_domain-containing_protein_[Thermoanaerobaculia_bacterium]	ptg004017l	41.84	1.61E-66	247	39
KAB2969039.1_MAG:_TetR_family_transcriptional_regulator_[Thermoanaerobaculia_bacterium]	ptg001473l	34.302	1.07E-26	109	89
KAB2969037.1_MAG:_glutamine--tRNA_ligase/YqeY_domain_fusion_protein_[Thermoanaerobaculia_bacterium]	ptg005010l	60.707	0	660	91
KAB2969036.1_MAG:_glutamate--tRNA_ligase_[Thermoanaerobaculia_bacterium]	ptg004724l	54.709	9.49E-160	507	94
KAB2969000.1_MAG:_RtcB_family_protein,_partial_[Thermoanaerobaculia_bacterium]	LG07	54	0.000000143	53.5	32
KAB2968999.1_MAG:_serine/threonine_protein_kinase,_partial_[Thermoanaerobaculia_bacterium]	ptg004652l	36.364	5.36E-43	161	85
KAB2968998.1_MAG:_SpoIIE_family_protein_phosphatase_[Thermoanaerobaculia_bacterium]	ptg004476l	32.243	3.79E-10	68.6	19
KAB2968995.1_MAG:_molybdopterin-dependent_oxidoreductase_[Thermoanaerobaculia_bacterium]	ptg005556l	25.762	2.81E-10	68.6	40
KAB2968986.1_MAG:_polysulfide_reductase_[Thermoanaerobaculia_bacterium]	ptg002453l	36.788	1.28E-67	238	82
KAB2968985.1_MAG:_4Fe-4S_dicluster_domain-containing_protein_[Thermoanaerobaculia_bacterium]	ptg001690l	39.024	1.36E-44	162	97
KAB2968984.1_MAG:_molybdopterin-dependent_oxidoreductase_[Thermoanaerobaculia_bacterium]	ptg004084l	24.783	1.92E-17	92	52
KAB2968983.1_MAG:_cytochrome_c3_family_protein_[Thermoanaerobaculia_bacterium]	ptg002453l	29.878	7.75E-16	77.4	85
KAB2968980.1_MAG:_SDR_family_oxidoreductase,_partial_[Thermoanaerobaculia_bacterium]	ptg005905l	40.556	1.74E-34	134	71
KAB2968938.1_MAG:_DUF1329_domain-containing_protein_[Thermoanaerobaculia_bacterium]	ptg004403l	56.164	1.63E-119	179	91
KAB2968937.1_MAG:_DUF1302_domain-containing_protein_[Thermoanaerobaculia_bacterium]	ptg004403l	44.118	8.75E-76	156	85
KAB2968936.1_MAG:_ornithine_carbamoyltransferase_[Thermoanaerobaculia_bacterium]	ptg003673l	37.113	5.98E-48	176	87
KAB2968934.1_MAG:_hypothetical_protein_F9K18_02100_[Thermoanaerobaculia_bacterium]	ptg002660l	27.381	8.16E-14	77.8	87
KAB2968933.1_MAG:_ornithine--oxo-acid_transaminase_[Thermoanaerobaculia_bacterium]	ptg003673l	37.629	2.75E-67	235	96
KAB2968931.1_MAG:_DMT_family_transporter_[Thermoanaerobaculia_bacterium]	ptg005680l	29.114	0.00000203	53.5	67
KAB2968929.1_MAG:_hypothetical_protein_F9K18_02075_[Thermoanaerobaculia_bacterium]	ptg002479l	35.979	1.19E-25	106	99
KAB2968889.1_MAG:_HAD_family_hydrolase_[Thermoanaerobaculia_bacterium]	ptg005448l	35.616	2.2E-24	102	79
KAB2968888.1_MAG:_3-deoxy-8-phosphooctulonate_synthase_[Thermoanaerobaculia_bacterium]	ptg003247l	55.042	1.5E-72	245	85
KAB2968887.1_MAG:_KpsF/GutQ_family_sugar-phosphate_isomerase_[Thermoanaerobaculia_bacterium]	ptg005709l	50	8.37E-60	127	88

KAB2968886.1_MAG:_hypothetical_protein_F9K18_02165_[Thermoanaerobaculia_bacterium]	ptg003199l	30.081	0.000000424	57.4	62
KAB2968885.1_MAG:_CTP_synthase_[Thermoanaerobaculia_bacterium]	ptg004256l	56.742	0	611	95
KAB2968884.1_MAG:_3-deoxy-manno-octulosonate_cytidylyltransferase_[Thermoanaerobaculia_bacterium]	ptg005527l	38.288	2.21E-36	127	96
KAB2968883.1_MAG:_polyprenyl_synthetase_family_protein_[Thermoanaerobaculia_bacterium]	ptg002505l	41.346	1.27E-44	167	60
KAB2968881.1_MAG:_YajQ_family_cyclic_di-GMP-binding_protein_[Thermoanaerobaculia_bacterium]	ptg000372l	37.736	1.22E-34	131	98
KAB2968879.1_MAG:_bifunctional_phosphoribosylaminoimidazolecarboxamide_formyltransferase/IMP_cyclohydrolase_[Thermoanaerobaculia_bacterium]	ptg004409l	46.525	9.59E-135	434	99
KAB2968838.1_MAG:_hypothetical_protein_F9K18_02215,_partial_[Thermoanaerobaculia_bacterium]	ptg005108l	41.436	5.36E-113	385	59
KAB2968837.1_MAG:_peptidase_M14_[Thermoanaerobaculia_bacterium]	ptg005108l	50.844	4.64E-161	526	58
KAB2968833.1_MAG:_malate_dehydrogenase_[Thermoanaerobaculia_bacterium]	ptg004470l	64.308	7.44E-125	397	99
KAB2968832.1_MAG:_zinc_ribbon_domain-containing_protein_[Thermoanaerobaculia_bacterium]	ptg004010l	45.455	0.00000224	46.6	58
KAB2968831.1_MAG:_aminodeoxychorismate_synthase,_component_I_[Thermoanaerobaculia_bacterium]	ptg004210l	40.909	1.42E-28	126	32
KAB2968828.1_MAG:_peptide_chain_release_factor_3_[Thermoanaerobaculia_bacterium]	ptg005772l	52.157	2.71E-164	520	96
KAB2968826.1_MAG:_tetratricopeptide_repeat_protein_[Thermoanaerobaculia_bacterium]	ptg002099l	33.553	1.9E-30	134	29
KAB2968787.1_MAG:_AAA_family_ATPase,_partial_[Thermoanaerobaculia_bacterium]	ptg005922l	39.286	2.88E-08	55.8	77
KAB2968785.1_MAG:_GGDEF_domain-containing_protein_[Thermoanaerobaculia_bacterium]	ptg002052l	40.271	1E-40	155	66
KAB2968779.1_MAG:_glycine_C-acetyltransferase_[Thermoanaerobaculia_bacterium]	ptg005614l	53.927	3.4E-130	417	92
KAB2968777.1_MAG:_AMP-binding_protein_[Thermoanaerobaculia_bacterium]	ptg004974l	36.155	2.6E-87	299	96
KAB2968776.1_MAG:_holo-ACP_synthase_[Thermoanaerobaculia_bacterium]	ptg003327l	31.313	0.000000117	52.4	79
KAB2968775.1_MAG:_lipoyl_synthase_[Thermoanaerobaculia_bacterium]	ptg003871l	48.421	3.19E-76	258	88
KAB2968774.1_MAG:_carboxylating_nicotinate-nucleotide_diphosphorylase_[Thermoanaerobaculia_bacterium]	ptg004584l	40.152	4.11E-48	175	92
KAB2968729.1_MAG:_HAMP_domain-containing_histidine_kinase_[Thermoanaerobaculia_bacterium]	ptg001814l	30.916	2.84E-28	119	77
KAB2968728.1_MAG:_response_regulator_transcription_factor_[Thermoanaerobaculia_bacterium]	ptg005010l	38.983	1.27E-50	180	96
KAB2968725.1_MAG:_citrate_synthase_[Thermoanaerobaculia_bacterium]	ptg005518l	57.009	1.29E-153	485	96
KAB2968696.1_MAG:_Glu/Leu/Phe/Val_dehydrogenase_[Thermoanaerobaculia_bacterium]	ptg002313l	39.486	1.47E-88	298	99
KAB2968693.1_MAG:_NADP-specific_glutamate_dehydrogenase_[Thermoanaerobaculia_bacterium]	ptg004340l	58.889	1.3E-170	535	99
KAB2968692.1_MAG:_EAL_domain-containing_protein,_partial_[Thermoanaerobaculia_bacterium]	ptg004457l	33.708	1.32E-24	107	93
KAB2968689.1_MAG:_VWA_domain-containing_protein_[Thermoanaerobaculia_bacterium]	ptg004575l	29.429	1.5E-22	103	81
KAB2968688.1_MAG:_phosphoribosylformylglycinamidine_synthase_subunit_PurL_[Thermoanaerobaculia_bacterium]	ptg005375l	31.242	6.48E-83	291	97
KAB2968687.1_MAG:_MBL_fold_metallo-hydrolase_[Thermoanaerobaculia_bacterium]	ptg004509l	37.447	4.36E-37	142	91
KAB2968686.1_MAG:_hypothetical_protein_F9K18_02460,_partial_[Thermoanaerobaculia_bacterium]	ptg005961l	40.392	7.12E-112	171	90
KAB2968685.1_MAG:_thiamine_pyrophosphate-binding_protein_[Thermoanaerobaculia_bacterium]	ptg002867l	31.122	1E-14	81.3	41
KAB2968684.1_MAG:_4Fe-4S_dicluster_domain-containing_protein_[Thermoanaerobaculia_bacterium]	ptg002102l	31.148	1E-12	74.7	38
KAB2968683.1_MAG:_amidophosphoribosyltransferase_[Thermoanaerobaculia_bacterium]	ptg000484l	37.342	1.27E-84	288	97

KAB2968682.1_MAG:_phosphoribosylformylglycinamidine_synthase_subunit_PurQ_[Thermoanaerobaculia_bacterium]	ptg005375l	31.963	1.01E-20	94.4	84
KAB2968647.1_MAG:_3-hydroxyacyl-ACP_dehydratase_FabZ_[Thermoanaerobaculia_bacterium]	ptg004895l	47.368	1.18E-34	130	92
KAB2968646.1_MAG:_S-methyl-5'-thioadenosine_phosphorylase_[Thermoanaerobaculia_bacterium]	ptg005967l	49.345	9.93E-71	240	75
KAB2968645.1_MAG:_Gfo/Idh/MocA_family_oxidoreductase_[Thermoanaerobaculia_bacterium]	ptg004000l	23.077	0.00000324	52.8	98
KAB2968644.1_MAG:_acyl-ACP--UDP-N-acetylglucosamine_O-acyltransferase_[Thermoanaerobaculia_bacterium]	ptg004895l	42.51	1.92E-59	207	90
KAB2968643.1_MAG:_UDP-3-O-(3-hydroxymyristoyl)glucosamine_N-acyltransferase_[Thermoanaerobaculia_bacterium]	ptg003363l	38.58	9.27E-42	159	94
KAB2968641.1_MAG:_outer_membrane_protein_assembly_factor_BamA_[Thermoanaerobaculia_bacterium]	ptg005656l	25.301	3.25E-33	142	82
KAB2968640.1_MAG:_ATP-dependent_Clp_protease_ATP-binding_subunit_[Thermoanaerobaculia_bacterium]	ptg003199l	44.811	0	702	98
KAB2968639.1_MAG:_ABC_transporter_ATP-binding_protein_[Thermoanaerobaculia_bacterium]	ptg001995l	47.847	3.88E-53	187	92
KAB2968596.1_MAG:_ketoacyl-ACP_synthase_III_[Thermoanaerobaculia_bacterium]	ptg003871l	31.198	5.42E-32	131	93
KAB2968595.1_MAG:_protein_kinase_[Thermoanaerobaculia_bacterium]	ptg003082l	36.691	3.93E-40	164	35
KAB2968594.1_MAG:_protein_kinase_[Thermoanaerobaculia_bacterium]	ptg003082l	37.722	2.43E-42	171	33
KAB2968592.1_MAG:_STAS_domain-containing_protein_[Thermoanaerobaculia_bacterium]	ptg003363l	30.769	0.000000057	52	96
KAB2968591.1_MAG:_sigma-70_family_RNA_polymerase_sigma_factor_[Thermoanaerobaculia_bacterium]	ptg004479l	37.173	2.88E-25	105	99
KAB2968590.1_MAG:_serine/threonine_protein_kinase_[Thermoanaerobaculia_bacterium]	ptg004928l	33.677	7.89E-30	124	80
KAB2968587.1_MAG:_hypothetical_protein_F9K18_02590_[Thermoanaerobaculia_bacterium]	ptg003233l	27.553	1.82E-11	70.9	88
KAB2968586.1_MAG:_sn-glycerol-3-phosphate_ABC_transporter_ATP-binding_protein_UgpC_[Thermoanaerobaculia_bacterium]	ptg005435l	49.724	6.77E-106	345	98
KAB2968585.1_MAG:_isochorismatase_family_protein_[Thermoanaerobaculia_bacterium]	ptg004529l	39.344	1.75E-22	97.8	91
KAB2968582.1_MAG:_transcriptional_repressor_[Thermoanaerobaculia_bacterium]	ptg003304l	28	0.000000432	50.8	93
KAB2968580.1_MAG:_ATP-binding_cassette_domain-containing_protein,_partial_[Thermoanaerobaculia_bacterium]	ptg004026l	34.328	1.02E-14	77.4	71
KAB2968551.1_MAG:_RnfABCDGE_type_electron_transport_complex_subunit_D_[Thermoanaerobaculia_bacterium]	ptg004917l	35.338	5.3E-23	103	81
KAB2968550.1_MAG:_RnfABCDGE_type_electron_transport_complex_subunit_E_[Thermoanaerobaculia_bacterium]	ptg004917l	37.313	8.08E-38	142	92
KAB2968549.1_MAG:_M13_family_metallopeptidase_[Thermoanaerobaculia_bacterium]	ptg001690l	30.573	2.28E-44	163	90
KAB2968547.1_MAG:_electron_transport_complex_subunit_RsxC_[Thermoanaerobaculia_bacterium]	ptg002214l	35	9.62E-73	253	97
KAB2968545.1_MAG:_electron_transport_complex_subunit_A_[Thermoanaerobaculia_bacterium]	ptg002214l	54	6.34E-37	107	96
KAB2968543.1_MAG:_ferredoxin-NADP_reductase_[Thermoanaerobaculia_bacterium]	LG07	40.769	5.34E-17	85.1	42
KAB2968542.1_MAG:_PLP-dependent_transferase_[Thermoanaerobaculia_bacterium]	ptg004895l	46.667	7.73E-97	320	93
KAB2968540.1_MAG:_ATP-binding_cassette_domain-containing_protein,_partial_[Thermoanaerobaculia_bacterium]	ptg001659l	44.444	1.45E-19	87.4	99
KAB2968535.1_MAG:_tetratricopeptide_repeat_protein,_partial_[Thermoanaerobaculia_bacterium]	ptg006047l	26.367	1.52E-11	71.6	53
KAB2968492.1_MAG:_sodium-translocating_pyrophosphatase_[Thermoanaerobaculia_bacterium]	ptg004711l	29.019	3.85E-38	158	87
KAB2968491.1_MAG:_sodium:calcium_symporter_[Thermoanaerobaculia_bacterium]	ptg003960l	28.402	2.93E-32	136	89
KAB2968488.1_MAG:_hypothetical_protein_F9K18_02795,_partial_[Thermoanaerobaculia_bacterium]	ptg002465l	28.293	0.000000003	60.8	82

KAB2968486.1_MAG:_LPS_export_ABC_transporter_ATP-binding_protein_[Thermoanaerobaculia_bacterium]	ptg005709l	55.042	9.15E-83	273	97
KAB2968485.1_MAG:_RNA_polymerase_factor_sigma-54_[Thermoanaerobaculia_bacterium]	ptg003787l	35.58	8.21E-58	211	75
KAB2968483.1_MAG:_RNase_adapter_RapZ_[Thermoanaerobaculia_bacterium]	ptg005611l	41.026	2.26E-53	191	90
KAB2968481.1_MAG:_HPr_family_phosphocarrier_protein_[Thermoanaerobaculia_bacterium]	ptg005611l	44.444	6.24E-16	74.3	99
KAB2968480.1_MAG:_phosphoenolpyruvate--protein_phosphotransferase_[Thermoanaerobaculia_bacterium]	ptg003452l	36.572	6.49E-80	278	95
KAB2968479.1_MAG:_cupin_domain-containing_protein_[Thermoanaerobaculia_bacterium]	ptg002270l	30.508	0.000000123	52	95
KAB2968447.1_MAG:_Rrf2_family_transcriptional_regulator_[Thermoanaerobaculia_bacterium]	ptg005518l	33.051	0.000000056	53.9	84
KAB2968445.1_MAG:_6-oxocyclohex-1-ene-1-carbonyl-CoA_hydratase_[Thermoanaerobaculia_bacterium]	ptg002612l	26.857	3.28E-11	68.9	47
KAB2968438.1_MAG:_glutamine-hydrolyzing_GMP_synthase_[Thermoanaerobaculia_bacterium]	ptg002345l	56.226	2.12E-166	525	99
KAB2968437.1_MAG:_NAD(P)-dependent_glycerol-3-phosphate_dehydrogenase,_partial_[Thermoanaerobaculia_bacterium]	ptg001005l	41.39	1.87E-57	203	100
KAB2968391.1_MAG:_acyl-CoA_thioesterase_[Thermoanaerobaculia_bacterium]	ptg004075l	34.483	1.58E-16	78.2	87
KAB2968388.1_MAG:_iron-sulfur_cluster_assembly_accessory_protein_[Thermoanaerobaculia_bacterium]	ptg000819l	37.903	2.01E-21	91.7	98
KAB2968385.1_MAG:_arsenate_reductase_ArsC_[Thermoanaerobaculia_bacterium]	ptg004266l	44.628	7.55E-23	96.7	81
KAB2968381.1_MAG:_AmpG_family_muropeptide_MFS_transporter,_partial_[Thermoanaerobaculia_bacterium]	ptg002429l	34.211	0.000000663	44.7	68
KAB2968377.1_MAG:_PLP-dependent_aminotransferase_family_protein_[Thermoanaerobaculia_bacterium]	ptg002021l	32.143	9.58E-42	161	85
KAB2968344.1_MAG:_S9_family_peptidase,_partial_[Thermoanaerobaculia_bacterium]	ptg003137l	56.356	2.24E-79	263	99
KAB2968340.1_MAG:_S9_family_peptidase_[Thermoanaerobaculia_bacterium]	ptg004136l	33.921	4.91E-113	379	94
KAB2968339.1_MAG:_threonine/serine_dehydratase_[Thermoanaerobaculia_bacterium]	ptg001814l	36.264	6.96E-24	105	86
KAB2968338.1_MAG:_long-chain_fatty_acid--CoA_ligase,_partial_[Thermoanaerobaculia_bacterium]	ptg005087l	25.075	4.22E-15	82.4	63
KAB2968337.1_MAG:_protease,_partial_[Thermoanaerobaculia_bacterium]	ptg003980l	27.815	3.23E-25	117	74
KAB2968331.1_MAG:_DUF3516_domain-containing_protein_[Thermoanaerobaculia_bacterium]	ptg006020l	32.667	3.27E-59	224	49
KAB2968329.1_MAG:_RluA_family_pseudouridine_synthase_[Thermoanaerobaculia_bacterium]	ptg006031l	29.882	4.02E-30	124	94
KAB2968328.1_MAG:_HAD-IA_family_hydrolase_[Thermoanaerobaculia_bacterium]	ptg005554l	46.875	2.56E-38	124	93
KAB2968294.1_MAG:_sulfur_carrier_protein_ThiS_[Thermoanaerobaculia_bacterium]	ptg003871l	45.161	2.51E-08	51.6	97
KAB2968293.1_MAG:_endopeptidase_La_[Thermoanaerobaculia_bacterium]	ptg005527l	52.493	0	780	96
KAB2968292.1_MAG:_transcription_termination_factor_Rho_[Thermoanaerobaculia_bacterium]	ptg003762l	66.827	0	590	94
KAB2968290.1_MAG:_trigger_factor_[Thermoanaerobaculia_bacterium]	ptg005527l	32.576	3.73E-16	85.1	31
KAB2968289.1_MAG:_ATP-dependent_Clp_endopeptidase_proteolytic_subunit_ClpP_[Thermoanaerobaculia_bacterium]	ptg005527l	60.309	2.82E-78	257	99
KAB2968288.1_MAG:_ATP-dependent_Clp_protease_ATP-binding_subunit_ClpX_[Thermoanaerobaculia_bacterium]	ptg004240l	61.22	2.75E-155	489	98
KAB2968287.1_MAG:_YihA_family_ribosome_biogenesis_GTP-binding_protein_[Thermoanaerobaculia_bacterium]	ptg004532l	34.634	1.47E-24	107	69
KAB2968286.1_MAG:_menaquinone_biosynthesis_protein_[Thermoanaerobaculia_bacterium]	ptg002867l	39	1.92E-24	103	95
KAB2968255.1_MAG:_large-conductance_mechanosensitive_channel_protein_MscL_[Thermoanaerobaculia_bacterium]	ptg003839l	33.577	2.57E-14	71.6	98

KAB2968253.1_MAG:_sigma-54-dependent_Fis_family_transcriptional_regulator_[Thermoanaerobaculia_bacterium]	ptg005150l	45.011	2.9E-123	399	96
KAB2968252.1_MAG:_HAMP_domain-containing_protein_[Thermoanaerobaculia_bacterium]	ptg003137l	33.555	2.51E-35	145	53
KAB2968250.1_MAG:_4Fe-4S_dicluster_domain-containing_protein_[Thermoanaerobaculia_bacterium]	ptg004084l	38.849	1.2E-20	94.7	52
KAB2968247.1_MAG:_SDR_family_oxidoreductase_[Thermoanaerobaculia_bacterium]	ptg005905l	37.073	2E-31	125	80
KAB2968245.1_MAG:_enoyl-CoA_hydratase_family_protein_[Thermoanaerobaculia_bacterium]	ptg001245l	33.465	5.12E-23	102	92
KAB2968211.1_MAG:_peptide_chain_release_factor_2_[Thermoanaerobaculia_bacterium]	ptg004457l	47.546	5.1E-82	275	93
KAB2968207.1_MAG:_apolipoprotein_N-acyltransferase_[Thermoanaerobaculia_bacterium]	ptg004790l	33.974	1.75E-15	77.8	74
KAB2968206.1_MAG:_apolipoprotein_N-acyltransferase_[Thermoanaerobaculia_bacterium]	ptg004790l	33.974	1.75E-15	77.8	74
KAB2968202.1_MAG:_peptide_chain_release_factor_2_[Thermoanaerobaculia_bacterium]	ptg004457l	47.546	5.1E-82	275	93
KAB2968164.1_MAG:_YceI_family_protein_[Thermoanaerobaculia_bacterium]	ptg002928l	27.841	9.05E-09	57.8	88
KAB2968162.1_MAG:_cysteine_synthase_family_protein_[Thermoanaerobaculia_bacterium]	ptg002030l	38.206	2.63E-53	191	92
KAB2968159.1_MAG:_glycosyl_hydrolase_[Thermoanaerobaculia_bacterium]	ptg005375l	45.069	0	905	97
KAB2968157.1_MAG:_hypothetical_protein_F9K18_03215,_partial_[Thermoanaerobaculia_bacterium]	ptg006047l	35.417	0.000000057	54.3	61
KAB2968156.1_MAG:_serine/threonine_protein_kinase,_partial_[Thermoanaerobaculia_bacterium]	ptg005260l	33.206	1.53E-31	127	87
KAB2968155.1_MAG:_response_regulator_transcription_factor_[Thermoanaerobaculia_bacterium]	ptg003829l	32.52	5.32E-27	113	93
KAB2968154.1_MAG:_hypothetical_protein_F9K18_03275_[Thermoanaerobaculia_bacterium]	ptg003829l	32.338	3.95E-29	122	59
KAB2968152.1_MAG:_pyridoxal_phosphate-dependent_aminotransferase_[Thermoanaerobaculia_bacterium]	ptg004905l	28.499	2.01E-41	160	96
KAB2968151.1_MAG:_phosphoenolpyruvate_carboxykinase_(ATP)_[Thermoanaerobaculia_bacterium]	ptg005856l	47.665	6.77E-142	457	93
KAB2968150.1_MAG:_aminopeptidase_P_family_protein_[Thermoanaerobaculia_bacterium]	ptg001995l	26.794	0.00000253	53.9	47
KAB2968114.1_MAG:_hydrogenase_[Thermoanaerobaculia_bacterium]	ptg003918l	27.363	7.19E-16	84.7	81
KAB2968113.1_MAG:_hydrogenase_[Thermoanaerobaculia_bacterium]	ptg004143l	24.033	4.64E-19	95.1	68
KAB2968112.1_MAG:_4Fe-4S_dicluster_domain-containing_protein_[Thermoanaerobaculia_bacterium]	ptg004143l	40.23	1.89E-14	76.6	34
KAB2968076.1_MAG:_SDR_family_oxidoreductase_[Thermoanaerobaculia_bacterium]	ptg005433l	52.564	5.33E-99	323	97
KAB2968075.1_MAG:_UDP-glucose/GDP-mannose_dehydrogenase_family_protein_[Thermoanaerobaculia_bacterium]	ptg001925l	45.786	2.19E-118	384	99
KAB2968074.1_MAG:_NAD-dependent_epimerase/dehydratase_family_protein_[Thermoanaerobaculia_bacterium]	ptg001690l	41.776	1.04E-68	236	94
KAB2968072.1_MAG:_ATP-binding_cassette_domain-containing_protein_[Thermoanaerobaculia_bacterium]	ptg003336l	41.935	1.93E-48	174	94
KAB2968071.1_MAG:_ABC_transporter_permease_[Thermoanaerobaculia_bacterium]	ptg003336l	42.857	2.52E-38	145	80
KAB2968069.1_MAG:_DNA_topoisomerase_III,_partial_[Thermoanaerobaculia_bacterium]	ptg002246l	47.953	8.06E-41	149	100
KAB2968065.1_MAG:_TrbI/VirB10_family_protein_[Thermoanaerobaculia_bacterium]	ptg005137l	35.256	8.58E-23	89	38
KAB2968030.1_MAG:_SOS_response-associated_peptidase_[Thermoanaerobaculia_bacterium]	ptg003566l	29.388	7.06E-12	68.2	99
KAB2968029.1_MAG:_M13_family_metallopeptidase_[Thermoanaerobaculia_bacterium]	ptg001690l	38.872	6.3E-58	216	84
KAB2968028.1_MAG:_site-2_protease_family_protein_[Thermoanaerobaculia_bacterium]	ptg004250l	34.595	6.3E-09	62	42
KAB2968027.1_MAG:_M2_family_metallopeptidase_[Thermoanaerobaculia_bacterium]	ptg003548l	55.944	0	700	93

KAB2968016.1_MAG:_protoporphyrinogen_oxidase,_partial_[Thermoanaerobaculia_bacterium]	ptg003764l	36.879	6.78E-24	102	72
KAB2967958.1_MAG:_S9_family_peptidase_[Thermoanaerobaculia_bacterium]	ptg002902l	49.02	2.48E-136	305	96
KAB2967957.1_MAG:_NADP-dependent_isocitrate_dehydrogenase_[Thermoanaerobaculia_bacterium]	ptg002573l	37.226	4.65E-10	65.9	31
KAB2967956.1_MAG:_glutamine-hydrolyzing_GMP_synthase_[Thermoanaerobaculia_bacterium]	ptg003536l	38.114	3.01E-92	319	67
KAB2967955.1_MAG:_hypothetical_protein_F9K18_03510_[Thermoanaerobaculia_bacterium]	ptg006047l	28.859	9.07E-14	77.8	62
KAB2967952.1_MAG:_heavy_metal_translocating_P-type_ATPase_[Thermoanaerobaculia_bacterium]	ptg003880l	35.5	2.36E-59	226	66
KAB2967950.1_MAG:_efflux_RND_transporter_permease_subunit_[Thermoanaerobaculia_bacterium]	ptg004670l	32.021	8.1E-161	532	99
KAB2967949.1_MAG:_efflux_RND_transporter_periplasmic_adaptor_subunit,_partial_[Thermoanaerobaculia_bacterium]	ptg001766l	29.851	2.48E-33	134	76
KAB2967946.1_MAG:_DnaJ_domain-containing_protein_[Thermoanaerobaculia_bacterium]	ptg005113l	36.875	4.74E-48	176	97
KAB2967945.1_MAG:_ferredoxin_family_protein_[Thermoanaerobaculia_bacterium]	ptg003928l	45.783	7.02E-16	73.9	99
KAB2967905.1_MAG:_ribose-phosphate_pyrophosphokinase_[Thermoanaerobaculia_bacterium]	ptg004982l	51.935	1.66E-108	350	99
KAB2967904.1_MAG:_4-(cytidine_5'-diphospho)-2-C-methyl-D-erythritol_kinase_[Thermoanaerobaculia_bacterium]	ptg003971l	32.404	1.47E-24	107	86
KAB2967903.1_MAG:_septation_regulator_SpoVG_[Thermoanaerobaculia_bacterium]	ptg003363l	32.203	7.62E-13	67	66
KAB2967902.1_MAG:_50S_ribosomal_protein_L25_[Thermoanaerobaculia_bacterium]	ptg004982l	32.749	9.72E-23	99.8	75
KAB2967901.1_MAG:_aminoacyl-tRNA_hydrolase_[Thermoanaerobaculia_bacterium]	ptg005867l	39.683	1.63E-19	89	100
KAB2967900.1_MAG:_30S_ribosomal_protein_S6_[Thermoanaerobaculia_bacterium]	ptg005487l	32.11	9.04E-11	61.6	83
KAB2967899.1_MAG:_30S_ribosomal_protein_S18_[Thermoanaerobaculia_bacterium]	ptg005487l	54.839	2.73E-14	69.7	68
KAB2967898.1_MAG:_50S_ribosomal_protein_L9_[Thermoanaerobaculia_bacterium]	ptg005756l	34.815	2.06E-17	81.3	91
KAB2967896.1_MAG:_PBP1A_family_penicillin-binding_protein_[Thermoanaerobaculia_bacterium]	ptg004827l	35.036	7.79E-102	348	83
KAB2967894.1_MAG:_alpha/beta_fold_hydrolase_[Thermoanaerobaculia_bacterium]	ptg004575l	29.01	2.66E-14	77.4	86
KAB2967893.1_MAG:_GGDEF_domain-containing_protein_[Thermoanaerobaculia_bacterium]	ptg005213l	31.977	2.86E-12	73.6	31
KAB2967890.1_MAG:_sigma-70_family_RNA_polymerase_sigma_factor_[Thermoanaerobaculia_bacterium]	ptg000484l	42.857	0.000000506	52	34
KAB2967857.1_MAG:_YbhB/YbcL_family_Raf_kinase_inhibitor-like_protein_[Thermoanaerobaculia_bacterium]	ptg002270l	38.931	2.98E-13	70.1	70
KAB2967854.1_MAG:_carbamoyl-phosphate_synthase_large_subunit_[Thermoanaerobaculia_bacterium]	ptg003839l	52.451	0	1112	97
KAB2967853.1_MAG:_ribonuclease_HI_[Thermoanaerobaculia_bacterium]	ptg001008l	44.203	3.34E-28	112	84
KAB2967823.1_MAG:_hypothetical_protein_F9K18_03765_[Thermoanaerobaculia_bacterium]	ptg003942l	32.738	2.22E-14	77.4	54
KAB2967822.1_MAG:_IMP_dehydrogenase_[Thermoanaerobaculia_bacterium]	ptg001460l	57.084	8.59E-167	525	99
KAB2967819.1_MAG:_preprotein_translocase_subunit_SecA_[Thermoanaerobaculia_bacterium]	ptg005005l	50.12	0	800	89
KAB2967818.1_MAG:_M23_family_metallopeptidase_[Thermoanaerobaculia_bacterium]	ptg004510l	45.69	1.71E-23	103	40
KAB2967817.1_MAG:_YifB_family_Mg_chelatase-like_AAA_ATPase,_partial_[Thermoanaerobaculia_bacterium]	ptg004355l	50.314	1.42E-93	309	89
KAB2967816.1_MAG:_peptide_chain_release_factor_1_[Thermoanaerobaculia_bacterium]	ptg004982l	49.284	9.79E-82	275	97
KAB2967815.1_MAG:_50S_ribosomal_protein_L31_[Thermoanaerobaculia_bacterium]	ptg004827l	53.125	2.99E-19	82.8	94

KAB2967814.1_MAG:_sigma-54-dependent_Fis_family_transcriptional_regulator_[Thermoanaerobaculia_bacterium]	ptg005150l	42.699	1.23E-113	372	95
KAB2967813.1_MAG:_PAS_domain-containing_protein_[Thermoanaerobaculia_bacterium]	ptg002453l	35	4.54E-25	117	25
KAB2967781.1_MAG:_TerC_family_protein_[Thermoanaerobaculia_bacterium]	LG12	42.529	1.23E-13	75.5	61
KAB2967780.1_MAG:_sigma-70_family_RNA_polymerase_sigma_factor_[Thermoanaerobaculia_bacterium]	ptg004340l	44.079	1.47E-23	100	79
KAB2967779.1_MAG:_aminotransferase_class_I/II-fold_pyridoxal_phosphate-dependent_enzyme_[Thermoanaerobaculia_bacterium]	ptg004905l	28.346	2E-17	89	59
KAB2967777.1_MAG:_aminofutalosine_synthase_MqnE_[Thermoanaerobaculia_bacterium]	ptg001571l	51.667	6.87E-106	345	96
KAB2967775.1_MAG:_deoxynucleoside_kinase_[Thermoanaerobaculia_bacterium]	ptg004203l	26.136	1.47E-08	57.8	72
KAB2967772.1_MAG:_phosphofructokinase_[Thermoanaerobaculia_bacterium]	ptg003223l	34.302	3.51E-50	185	88
KAB2967771.1_MAG:_type_I_glyceraldehyde-3-phosphate_dehydrogenase_[Thermoanaerobaculia_bacterium]	ptg003168l	48.81	3.96E-92	304	99
KAB2967770.1_MAG:_type_I_glyceraldehyde-3-phosphate_dehydrogenase_[Thermoanaerobaculia_bacterium]	ptg003168l	36.452	2.15E-50	184	87
KAB2967769.1_MAG:_LacI_family_transcriptional_regulator_[Thermoanaerobaculia_bacterium]	ptg003973l	32.907	1.97E-36	144	87
KAB2967768.1_MAG:_TonB-dependent_receptor_[Thermoanaerobaculia_bacterium]	ptg001925l	23.953	3.48E-23	111	71
KAB2967741.1_MAG:_Re/Si-specific_NAD(P)(+)_transhydrogenase_subunit_alpha_[Thermoanaerobaculia_bacterium]	ptg004054l	45.205	1.78E-67	234	96
KAB2967737.1_MAG:_thiolase_family_protein_[Thermoanaerobaculia_bacterium]	ptg005527l	44.961	1.13E-67	235	99
KAB2967736.1_MAG:_3-hydroxyacyl-CoA_dehydrogenase,_partial_[Thermoanaerobaculia_bacterium]	ptg002760l	33.945	6.1E-21	102	30
KAB2967707.1_MAG:_SsrA-binding_protein_SmpB_[Thermoanaerobaculia_bacterium]	ptg002723l	40.157	1.37E-21	93.6	81
KAB2967704.1_MAG:_excinuclease_ABC_subunit_UvrA_[Thermoanaerobaculia_bacterium]	ptg004916l	55.781	0	891	98
KAB2967702.1_MAG:_long-chain_fatty_acid--CoA_ligase_[Thermoanaerobaculia_bacterium]	ptg005087l	32.326	7.31E-30	129	53
KAB2967700.1_MAG:_MFS_transporter_[Thermoanaerobaculia_bacterium]	ptg003813l	28.994	1.88E-08	60.8	37
KAB2967666.1_MAG:_GIY-YIG_nuclease_family_protein_[Thermoanaerobaculia_bacterium]	ptg001995l	47.917	0.000000109	50.8	56
KAB2967661.1_MAG:_radical_SAM_protein_[Thermoanaerobaculia_bacterium]	ptg005425l	26.498	3.69E-26	116	66
KAB2967629.1_MAG:_serine/threonine_protein_kinase,_partial_[Thermoanaerobaculia_bacterium]	ptg004564l	40.614	1.36E-55	198	92
KAB2967628.1_MAG:_3-methyl-2-oxobutanoate_hydroxymethyltransferase_[Thermoanaerobaculia_bacterium]	ptg004655l	48.106	1.37E-63	219	93
KAB2967627.1_MAG:_pantoate--beta-alanine_ligase_[Thermoanaerobaculia_bacterium]	LG03	50.267	8.22E-49	177	66
KAB2967626.1_MAG:_aspartate_1-decarboxylase_[Thermoanaerobaculia_bacterium]	ptg004018l	49.63	4.6E-32	122	98
KAB2967624.1_MAG:_4Fe-4S_dicluster_domain-containing_protein_[Thermoanaerobaculia_bacterium]	ptg005913l	45.736	2.29E-32	126	65
KAB2967622.1_MAG:_hypothetical_protein_F9K18_04010,_partial_[Thermoanaerobaculia_bacterium]	ptg001624l	40.476	1.39E-40	160	42
KAB2967591.1_MAG:_D-alanine--D-alanine_ligase_[Thermoanaerobaculia_bacterium]	ptg004510l	32.806	6.36E-21	99	62
KAB2967588.1_MAG:_ABC_transporter_substrate-binding_protein_[Thermoanaerobaculia_bacterium]	ptg003947l	34.52	2.08E-35	138	94
KAB2967587.1_MAG:_HAD-IA_family_hydrolase_[Thermoanaerobaculia_bacterium]	ptg004584l	36.792	6.09E-16	61.6	73
KAB2967585.1_MAG:_hypothetical_protein_F9K18_04110,_partial_[Thermoanaerobaculia_bacterium]	ptg003450l	44.027	2.57E-57	223	50

KAB2967553.1_MAG:_enterochelin_esterase_[Thermoanaerobaculia_bacterium]	ptg004501l	28.866	0.000000139	57.8	41
KAB2967551.1_MAG:_acyl-CoA_thioesterase_[Thermoanaerobaculia_bacterium]	ptg001690l	37.795	4.93E-25	102	91
KAB2967502.1_MAG:_hypothetical_protein_F9K18_04185_[Thermoanaerobaculia_bacterium]	ptg001025l	37.755	2.3E-42	108	45
KAB2967501.1_MAG:_metallophosphoesterase_family_protein_[Thermoanaerobaculia_bacterium]	ptg005702l	33.047	1.92E-19	90.9	98
KAB2967500.1_MAG:_shikimate_kinase_[Thermoanaerobaculia_bacterium]	ptg004564l	36.145	8.11E-21	92	92
KAB2967499.1_MAG:_thiamine_phosphate_synthase_[Thermoanaerobaculia_bacterium]	ptg003199l	45.192	3.44E-10	62.8	48
KAB2967498.1_MAG:_acetyl-CoA_carboxylase_biotin_carboxylase_subunit_[Thermoanaerobaculia_bacterium]	ptg004240l	56.885	1.86E-155	491	98
KAB2967497.1_MAG:_acetyl-CoA_carboxylase_biotin_carboxyl_carrier_protein,_partial_[Thermoanaerobaculia_bacterium]	ptg004240l	48.571	3.9E-14	69.7	69
KAB2967496.1_MAG:_polyphosphate_kinase_1_[Thermoanaerobaculia_bacterium]	ptg005354l	40.028	4.25E-152	492	98
KAB2967494.1_MAG:_inorganic_phosphate_transporter_[Thermoanaerobaculia_bacterium]	ptg004169l	35.882	6.25E-20	95.1	46
KAB2967493.1_MAG:_Ppx/GppA_family_phosphatase_[Thermoanaerobaculia_bacterium]	ptg005354l	37.647	3.91E-28	123	31
KAB2967492.1_MAG:_HAMP_domain-containing_protein_[Thermoanaerobaculia_bacterium]	ptg001595l	30.798	4.37E-49	188	87
KAB2967459.1_MAG:_tRNA_(N6-isopentenyl_adenosine(37)-C2)-methylthiotransferase_MiaB_[Thermoanaerobaculia_bacterium]	ptg005772l	41.798	1.13E-103	344	87
KAB2967458.1_MAG:_ParB/RepB/Spo0J_family_partition_protein_[Thermoanaerobaculia_bacterium]	ptg000372l	39.13	1.59E-24	107	54
KAB2967457.1_MAG:_ParA_family_protein_[Thermoanaerobaculia_bacterium]	ptg001818l	42.857	2.24E-58	203	99
KAB2967456.1_MAG:_bifunctional_nuclease_family_protein_[Thermoanaerobaculia_bacterium]	ptg003247l	38.931	4.6E-21	92.8	75
KAB2967455.1_MAG:_6-carboxytetrahydropterin_synthase_[Thermoanaerobaculia_bacterium]	ptg001897l	40.566	2.47E-15	74.7	76
KAB2967453.1_MAG:_hypothetical_protein_F9K18_04240_[Thermoanaerobaculia_bacterium]	ptg002475l	64.103	2.86E-09	53.9	62
KAB2967425.1_MAG:_DNA-formamidopyrimidine_glycosylase,_partial_[Thermoanaerobaculia_bacterium]	ptg002199l	51.304	9.67E-26	104	85
KAB2967424.1_MAG:_uridine_kinase_[Thermoanaerobaculia_bacterium]	ptg001291l	41.262	4.55E-41	151	95
KAB2967423.1_MAG:_SLC13_family_permease_[Thermoanaerobaculia_bacterium]	ptg005195l	31.399	3.43E-27	121	50
KAB2967418.1_MAG:_redoxin_domain-containing_protein_[Thermoanaerobaculia_bacterium]	ptg004523l	23.077	0.000000131	54.7	80
KAB2967389.1_MAG:_peptidase_S41_[Thermoanaerobaculia_bacterium]	ptg003980l	24.386	3.09E-68	256	94
KAB2967388.1_MAG:_translational_GTPase_TypA_[Thermoanaerobaculia_bacterium]	LG18	50.915	0	617	99
KAB2967386.1_MAG:_amidohydrolase_[Thermoanaerobaculia_bacterium]	ptg006022l	36.066	1.63E-60	215	88
KAB2967384.1_MAG:_DUF1820_family_protein_[Thermoanaerobaculia_bacterium]	ptg004790l	55.814	6.22E-28	109	74
KAB2967383.1_MAG:_ABC_transporter_ATP-binding_protein_[Thermoanaerobaculia_bacterium]	ptg001659l	44.643	1.13E-57	201	92
KAB2967382.1_MAG:_ABC_transporter_permease_[Thermoanaerobaculia_bacterium]	ptg004986l	26.304	6.22E-26	114	99
KAB2967381.1_MAG:_ABC_transporter_permease_[Thermoanaerobaculia_bacterium]	ptg004986l	26.158	7.08E-20	96.3	79
KAB2967378.1_MAG:_N-acetyltransferase_[Thermoanaerobaculia_bacterium]	ptg002992l	36.436	3.09E-56	202	99
KAB2967360.1_MAG:_phosphoglucosamine_mutase,_partial_[Thermoanaerobaculia_bacterium]	ptg004534l	35.507	2.46E-33	133	91
KAB2967359.1_MAG:_hypothetical_protein_F9K18_04400_[Thermoanaerobaculia_bacterium]	ptg002660l	25.664	0.000000235	57.4	71

KAB2967358.1_MAG:_pyridoxine_5'-phosphate_synthase_[Thermoanaerobaculia_bacterium]	ptg003483l	49.112	1.24E-38	127	98
KAB2967356.1_MAG:_sigma-54-dependent_Fis_family_transcriptional_regulator_[Thermoanaerobaculia_bacterium]	ptg005150l	42.228	6.4E-98	327	79
KAB2967327.1_MAG:_hypothetical_protein_F9K18_04435_[Thermoanaerobaculia_bacterium]	ptg004790l	33.333	0.000000766	50.4	74
KAB2967323.1_MAG:_peptide_chain_release_factor_2_[Thermoanaerobaculia_bacterium]	ptg004479l	60.324	1.4E-85	286	70
KAB2967295.1_MAG:_sulfatase,_partial_[Thermoanaerobaculia_bacterium]	ptg000693l	24.471	4E-18	90.9	90
KAB2967292.1_MAG:_bifunctional_3,4-dihydroxy-2-butanone-4-phosphate_synthase/GTP_cyclohydrolase_II_[Thermoanaerobaculia_bacterium]	ptg003075l	50.25	8.86E-107	349	97
KAB2967291.1_MAG:_BamA/TamA_family_outer_membrane_protein,_partial_[Thermoanaerobaculia_bacterium]	ptg005656l	25.373	1.44E-22	108	87
KAB2967290.1_MAG:_hypothetical_protein_F9K18_04485,_partial_[Thermoanaerobaculia_bacterium]	LG07	33.831	3.35E-13	77	32
KAB2967289.1_MAG:_hypothetical_protein_F9K18_04480_[Thermoanaerobaculia_bacterium]	LG07	37.288	3.9E-21	104	68
KAB2967286.1_MAG:_metal-dependent_hydrolase_[Thermoanaerobaculia_bacterium]	ptg005425l	38.397	1.16E-31	126	91
KAB2967258.1_MAG:_HAMP_domain-containing_protein,_partial_[Thermoanaerobaculia_bacterium]	ptg001595l	32.649	1.57E-45	176	96
KAB2967257.1_MAG:_acetate--CoA_ligase_[Thermoanaerobaculia_bacterium]	LG03	42.038	5.98E-156	501	94
KAB2967256.1_MAG:_hypothetical_protein_F9K18_04500_[Thermoanaerobaculia_bacterium]	ptg004885l	28.272	3.43E-08	60.1	40
KAB2967255.1_MAG:_phosphate_signaling_complex_protein_PhoU_[Thermoanaerobaculia_bacterium]	ptg004075l	40.183	1.72E-45	165	98
KAB2967254.1_MAG:_phosphate_ABC_transporter_substrate-binding_protein_[Thermoanaerobaculia_bacterium]	ptg002567l	60.067	1E-112	362	92
KAB2967252.1_MAG:_transposase_[Thermoanaerobaculia_bacterium]	ptg001897l	31.707	3.07E-24	104	80
KAB2967249.1_MAG:_signal_peptide_peptidase_SppA_[Thermoanaerobaculia_bacterium]	ptg004077l	26.813	1.94E-31	134	75
KAB2967223.1_MAG:_DNA_topoisomerase_III_[Thermoanaerobaculia_bacterium]	ptg002246l	38.743	4.76E-101	344	78
KAB2967194.1_MAG:_response_regulator,_partial_[Thermoanaerobaculia_bacterium]	ptg002316l	37.931	2.86E-20	88.6	94
KAB2967193.1_MAG:_cobalamin_B12-binding_domain-containing_protein_[Thermoanaerobaculia_bacterium]	LG30	41.758	6.68E-10	58.9	66
KAB2967192.1_MAG:_DUF664_domain-containing_protein_[Thermoanaerobaculia_bacterium]	ptg003871l	25.157	0.000000395	52.4	87
KAB2967191.1_MAG:_methylmalonyl-CoA_mutase_family_protein_[Thermoanaerobaculia_bacterium]	LG30	52.439	1.03E-14	82	42
KAB2967189.1_MAG:_acyl-CoA_dehydrogenase_[Thermoanaerobaculia_bacterium]	ptg003831l	30.447	1.34E-29	124	82
KAB2967188.1_MAG:_acyl-CoA_dehydrogenase_[Thermoanaerobaculia_bacterium]	ptg004179l	30.141	9.84E-30	125	91
KAB2967167.1_MAG:_translation_initiation_factor_IF-2,_partial_[Thermoanaerobaculia_bacterium]	ptg001690l	44.759	1.62E-140	459	85
KAB2967165.1_MAG:_30S_ribosome-binding_factor_RbfA_[Thermoanaerobaculia_bacterium]	ptg003903l	37.838	1.75E-14	71.6	93
KAB2967164.1_MAG:_bifunctional_oligoribonuclease/PAP_phosphatase_NrnA_[Thermoanaerobaculia_bacterium]	ptg005886l	30.325	5.74E-20	94.7	83
KAB2967163.1_MAG:_tRNA_pseudouridine(55)_synthase_TruB_[Thermoanaerobaculia_bacterium]	ptg003078l	43.458	2.11E-41	156	69
KAB2967162.1_MAG:_30S_ribosomal_protein_S15_[Thermoanaerobaculia_bacterium]	ptg004026l	48.315	2.18E-23	95.5	100
KAB2967161.1_MAG:_polyribonucleotide_nucleotidyltransferase,_partial_[Thermoanaerobaculia_bacterium]	ptg004026l	47.067	0	624	93
KAB2967132.1_MAG:_succinate--CoA_ligase_subunit_alpha,_partial_[Thermoanaerobaculia_bacterium]	ptg002316l	61.278	1.76E-91	299	100
KAB2967131.1_MAG:_ADP-forming_succinate--CoA_ligase_subunit_beta_[Thermoanaerobaculia_bacterium]	ptg002316l	51.07	1.16E-93	310	94

KAB2967130.1_MAG:_PP2C_family_protein-serine/threonine_phosphatase_[Thermoanaerobaculia_bacterium]	ptg004476l	29.114	2.18E-14	82	29
KAB2967129.1_MAG:_cytidine_deaminase_[Thermoanaerobaculia_bacterium]	ptg000909l	67.391	0.000000384	51.2	35
KAB2967128.1_MAG:_M1_family_peptidase,_partial_[Thermoanaerobaculia_bacterium]	ptg004724l	27.689	1.97E-37	155	56
KAB2967127.1_MAG:_DEAD/DEAH_box_helicase,_partial_[Thermoanaerobaculia_bacterium]	ptg003863l	50.535	1.25E-105	347	86
KAB2967126.1_MAG:_response_regulator_[Thermoanaerobaculia_bacterium]	ptg005035l	30.791	8.74E-31	133	87
KAB2967125.1_MAG:_response_regulator_[Thermoanaerobaculia_bacterium]	LG04	35.965	3.69E-19	85.1	92
KAB2967124.1_MAG:_response_regulator_[Thermoanaerobaculia_bacterium]	ptg002909l	37.766	9.74E-58	218	49
KAB2967123.1_MAG:_DinB_family_protein_[Thermoanaerobaculia_bacterium]	ptg004158l	39.535	1.1E-33	129	92
KAB2967099.1_MAG:_GHKL_domain-containing_protein_[Thermoanaerobaculia_bacterium]	ptg002304l	28.251	7.48E-11	69.3	42
KAB2967098.1_MAG:_response_regulator_transcription_factor_[Thermoanaerobaculia_bacterium]	ptg003999l	32.743	4.36E-14	58.9	89
KAB2967096.1_MAG:_hypothetical_protein_F9K18_04715_[Thermoanaerobaculia_bacterium]	ptg002909l	39.175	2.11E-31	123	97
KAB2967095.1_MAG:_ParA_family_protein_[Thermoanaerobaculia_bacterium]	ptg000372l	35.341	4.68E-37	143	86
KAB2967094.1_MAG:_NADPH:quinone_oxidoreductase_family_protein_[Thermoanaerobaculia_bacterium]	ptg004026l	30.702	4.96E-26	112	99
KAB2967068.1_MAG:_beta-ketoacyl_synthase_[Thermoanaerobaculia_bacterium]	ptg002723l	31.278	7.69E-16	85.1	41
KAB2967067.1_MAG:_type_I_glyceraldehyde-3-phosphate_dehydrogenase_[Thermoanaerobaculia_bacterium]	ptg002223l	48.308	7.52E-97	317	95
KAB2967066.1_MAG:_type_I_glyceraldehyde-3-phosphate_dehydrogenase_[Thermoanaerobaculia_bacterium]	ptg005611l	35.608	1.93E-54	196	94
KAB2967064.1_MAG:_hydroxyacid_dehydrogenase,_partial_[Thermoanaerobaculia_bacterium]	ptg001693l	62.069	4.19E-45	132	93
KAB2967036.1_MAG:_isoprenylcysteine_carboxylmethyltransferase_family_protein_[Thermoanaerobaculia_bacterium]	ptg002628l	40.625	5.75E-08	54.7	60
KAB2967035.1_MAG:_pyruvate_dehydrogenase_(acetyl-transferring),_homodimeric_type_[Thermoanaerobaculia_bacterium]	ptg005844l	60.956	0	618	56
KAB2967018.1_MAG:_3-deoxy-7-phosphoheptulonate_synthase_class_II_[Thermoanaerobaculia_bacterium]	ptg003327l	59.41	1.39E-174	546	98
KAB2967017.1_MAG:_serine_hydroxymethyltransferase_[Thermoanaerobaculia_bacterium]	ptg000693l	56.404	2.95E-142	451	98
KAB2967016.1_MAG:_ribose_5-phosphate_isomerase_B_[Thermoanaerobaculia_bacterium]	ptg005354l	50	3.56E-29	114	97
KAB2967015.1_MAG:_sulfatase_[Thermoanaerobaculia_bacterium]	ptg000693l	30.488	8.7E-10	66.2	26
KAB2967014.1_MAG:_acyl-CoA_carboxylase_subunit_beta_[Thermoanaerobaculia_bacterium]	ptg003168l	63.699	0	573	85
KAB2966991.1_MAG:_ATP_synthase_F0_subunit_C_[Thermoanaerobaculia_bacterium]	ptg004470l	75	0.00000232	46.2	42
KAB2966990.1_MAG:_F0F1_ATP_synthase_subunit_B_[Thermoanaerobaculia_bacterium]	ptg004652l	43.976	1.53E-22	97.8	85
KAB2966989.1_MAG:_ATP_synthase_F1_subunit_delta_[Thermoanaerobaculia_bacterium]	ptg002658l	29.299	3.88E-13	70.9	79
KAB2966988.1_MAG:_F0F1_ATP_synthase_subunit_alpha_[Thermoanaerobaculia_bacterium]	ptg004470l	55.903	0	621	98
KAB2966987.1_MAG:_ATP_synthase_F1_subunit_gamma_[Thermoanaerobaculia_bacterium]	ptg004470l	45.085	1.05E-71	243	99
KAB2966986.1_MAG:_F0F1_ATP_synthase_subunit_beta_[Thermoanaerobaculia_bacterium]	ptg004470l	73.65	0	663	96
KAB2966985.1_MAG:_ATP_synthase_F1_subunit_epsilon_[Thermoanaerobaculia_bacterium]	ptg002658l	41.026	7.8E-11	61.6	60
KAB2966960.1_MAG:_Tol-Pal_system_beta_propeller_repeat_protein_TolB,_partial_[Thermoanaerobaculia_bacterium]	ptg005886l	30.435	1.41E-14	80.1	45

KAB2966959.1_MAG:_peptidoglycan-associated_lipoprotein_Pal_[Thermoanaerobaculia_bacterium]	ptg000834l	39.091	0.00000011	54.7	56
KAB2966957.1_MAG:_30S_ribosomal_protein_S20_[Thermoanaerobaculia_bacterium]	ptg002505l	48.485	1.03E-10	59.3	75
KAB2966955.1_MAG:_leucine--tRNA_ligase_[Thermoanaerobaculia_bacterium]	ptg005844l	35.544	8.18E-172	554	99
KAB2966927.1_MAG:_hypothetical_protein_F9K18_04960,_partial_[Thermoanaerobaculia_bacterium]	ptg004470l	40.523	1.32E-19	90.1	71
KAB2966925.1_MAG:_NTP_transferase_domain-containing_protein_[Thermoanaerobaculia_bacterium]	ptg004242l	38.689	2.97E-55	197	83
KAB2966921.1_MAG:_endoglucanase_[Thermoanaerobaculia_bacterium]	ptg005886l	33.333	9.15E-29	126	36
KAB2966899.1_MAG:_GNAT_family_N-acetyltransferase_[Thermoanaerobaculia_bacterium]	ptg001473l	31.336	8.06E-18	89.7	53
KAB2966898.1_MAG:_carbon-nitrogen_hydrolase_family_protein_[Thermoanaerobaculia_bacterium]	ptg001473l	35.842	1.75E-47	173	94
KAB2966896.1_MAG:_efflux_RND_transporter_permease_subunit_[Thermoanaerobaculia_bacterium]	ptg003498l	26.476	9.51E-89	318	95
KAB2966895.1_MAG:_efflux_RND_transporter_periplasmic_adaptor_subunit_[Thermoanaerobaculia_bacterium]	ptg000732l	24.855	3.98E-16	72.8	67
KAB2966873.1_MAG:_phosphoglycerate_mutase,_partial_[Thermoanaerobaculia_bacterium]	ptg002573l	23.684	0.00000231	52	71
KAB2966871.1_MAG:_hypothetical_protein_F9K18_05020_[Thermoanaerobaculia_bacterium]	ptg004114l	25.946	0.00000544	52	56
KAB2966870.1_MAG:_macro_domain-containing_protein_[Thermoanaerobaculia_bacterium]	ptg003471l	38.129	9.35E-16	77.8	73
KAB2966869.1_MAG:_arginine--tRNA_ligase_[Thermoanaerobaculia_bacterium]	ptg005430l	25.294	0.00000177	55.5	24
KAB2966866.1_MAG:_chromate_transporter,_partial_[Thermoanaerobaculia_bacterium]	ptg005301l	37.333	1.15E-08	57	43
KAB2966842.1_MAG:_ABC_transporter_ATP-binding_protein_[Thermoanaerobaculia_bacterium]	ptg005920l	53.695	9.08E-69	232	88
KAB2966840.1_MAG:_FtsX-like_permease_family_protein_[Thermoanaerobaculia_bacterium]	ptg005920l	33.742	5.15E-30	126	70
KAB2966837.1_MAG:_TolC_family_protein_[Thermoanaerobaculia_bacterium]	ptg001814l	25	5.84E-18	92	66
KAB2966816.1_MAG:_nitrate_reductase_catalytic_subunit_NapA_[Thermoanaerobaculia_bacterium]	ptg002844l	42.222	3.94E-90	290	91
KAB2966815.1_MAG:_cysteine_synthase_family_protein_[Thermoanaerobaculia_bacterium]	ptg005554l	42.949	5.18E-57	202	94
KAB2966814.1_MAG:_hypothetical_protein_F9K18_05100_[Thermoanaerobaculia_bacterium]	LG02	33.803	3.87E-16	82.8	94
KAB2966813.1_MAG:_DNA_methyltransferase_[Thermoanaerobaculia_bacterium]	LG07	52.459	1.36E-09	62.8	20
KAB2966787.1_MAG:_efflux_transporter_outer_membrane_subunit_[Thermoanaerobaculia_bacterium]	ptg005487l	31.383	1.75E-14	80.1	40
KAB2966786.1_MAG:_efflux_RND_transporter_permease_subunit_[Thermoanaerobaculia_bacterium]	ptg002381l	53.247	0	426	96
KAB2966785.1_MAG:_efflux_RND_transporter_periplasmic_adaptor_subunit_[Thermoanaerobaculia_bacterium]	ptg002381l	40.964	1.62E-49	183	84
KAB2966760.1_MAG:_2-oxo_acid_dehydrogenase_subunit_E2_[Thermoanaerobaculia_bacterium]	ptg003019l	42.534	3.83E-51	183	80
KAB2966757.1_MAG:_hypothetical_protein_F9K18_05150_[Thermoanaerobaculia_bacterium]	ptg002453l	32.895	9.86E-20	97.4	41
KAB2966756.1_MAG:_deoxyhypusine_synthase_family_protein_[Thermoanaerobaculia_bacterium]	ptg005865l	56.79	2.51E-101	330	99
KAB2966755.1_MAG:_lipoyl(octanoyl)_transferase_LipB_[Thermoanaerobaculia_bacterium]	ptg004799l	48.913	5.37E-13	71.6	64
KAB2966754.1_MAG:_hypothetical_protein_F9K18_05135,_partial_[Thermoanaerobaculia_bacterium]	ptg002634l	42.391	0.00000038	52.8	48
KAB2966730.1_MAG:_response_regulator,_partial_[Thermoanaerobaculia_bacterium]	ptg003548l	52	2.12E-09	57.4	38
KAB2966729.1_MAG:_sensor_histidine_kinase_[Thermoanaerobaculia_bacterium]	ptg003548l	42.157	1.31E-14	79.3	28
KAB2966725.1_MAG:_PAS_domain_S-box_protein,_partial_[Thermoanaerobaculia_bacterium]	ptg001291l	30.038	1.08E-22	108	30
KAB2966701.1_MAG:_CDP-glucose_4,6-dehydratase_[Thermoanaerobaculia_bacterium]	ptg006001l	25.852	2.09E-14	79	86

KAB2966696.1_MAG:_dTDP-4-dehydrorhamnose_3,5-epimerase_[Thermoanaerobaculia_bacterium]	ptg003827l	36.471	1.09E-31	123	94
KAB2966678.1_MAG:_DNA_repair_protein_RadC,_partial_[Thermoanaerobaculia_bacterium]	ptg002199l	35.762	1.8E-24	101	98
KAB2966677.1_MAG:_methionine--tRNA_ligase_[Thermoanaerobaculia_bacterium]	ptg002214l	28.777	2.14E-65	238	99
KAB2966676.1_MAG:_TatD_family_deoxyribonuclease_[Thermoanaerobaculia_bacterium]	ptg005067l	38.824	9.49E-40	153	74
KAB2966674.1_MAG:_methylated-DNA--[protein]-cysteine_S-methyltransferase_[Thermoanaerobaculia_bacterium]	ptg005680l	29.286	1.48E-09	60.1	72
KAB2966673.1_MAG:_hypothetical_protein_F9K18_05245_[Thermoanaerobaculia_bacterium]	ptg003583l	41.667	8.85E-16	75.1	86
KAB2966655.1_MAG:_oxidative_damage_protection_protein_[Thermoanaerobaculia_bacterium]	ptg004779l	31.944	0.00000718	45.8	77
KAB2966653.1_MAG:_hypothetical_protein_F9K18_05290_[Thermoanaerobaculia_bacterium]	ptg002660l	28.533	5.13E-12	72.4	83
KAB2966652.1_MAG:_hypothetical_protein_F9K18_05285_[Thermoanaerobaculia_bacterium]	ptg002660l	26.567	1.1E-10	68.2	79
KAB2966651.1_MAG:_hypothetical_protein_F9K18_05280_[Thermoanaerobaculia_bacterium]	ptg002660l	30.581	3.24E-13	76.3	78
KAB2966650.1_MAG:_hypothetical_protein_F9K18_05275,_partial_[Thermoanaerobaculia_bacterium]	ptg000619l	29.923	1.28E-43	167	89
KAB2966630.1_MAG:_glycosyltransferase_family_4_protein,_partial_[Thermoanaerobaculia_bacterium]	ptg001909l	27.208	2.5E-12	72.8	60
KAB2966629.1_MAG:_undecaprenyl-phosphate_glucose_phosphotransferase_[Thermoanaerobaculia_bacterium]	ptg000819l	35.625	3.8E-48	183	60
KAB2966628.1_MAG:_carbon_starvation_protein_A_[Thermoanaerobaculia_bacterium]	ptg002928l	31.739	2.36E-38	155	71
KAB2966625.1_MAG:_ATP-binding_protein,_partial_[Thermoanaerobaculia_bacterium]	ptg003942l	58.146	2.43E-148	413	95
KAB2966601.1_MAG:_heavy_metal_translocating_P-type_ATPase,_partial_[Thermoanaerobaculia_bacterium]	ptg003880l	31.847	2.16E-08	59.3	49
KAB2966600.1_MAG:_cytochrome-c_oxidase,_cbb3-type_subunit_I_[Thermoanaerobaculia_bacterium]	ptg003901l	73.512	7.87E-154	487	71
KAB2966599.1_MAG:_cytochrome-c_oxidase,_cbb3-type_subunit_II_[Thermoanaerobaculia_bacterium]	ptg006037l	39.32	2.53E-38	143	85
KAB2966597.1_MAG:_cytochrome-c_oxidase,_cbb3-type_subunit_III_[Thermoanaerobaculia_bacterium]	ptg003880l	33.213	6.45E-39	149	86
KAB2966596.1_MAG:_cytochrome_c_oxidase_accessory_protein_CcoG_[Thermoanaerobaculia_bacterium]	ptg003880l	40.594	1.26E-65	233	79
KAB2966591.1_MAG:_tetratricopeptide_repeat_protein,_partial_[Thermoanaerobaculia_bacterium]	LG25	31.609	0.000000187	59.3	19
KAB2966569.1_MAG:_aminotransferase_class_I/II-fold_pyridoxal_phosphate-dependent_enzyme,_partial_[Thermoanaerobaculia_bacterium]	ptg002313l	36.052	4.77E-30	123	78
KAB2966568.1_MAG:_biotin_synthase_BioB_[Thermoanaerobaculia_bacterium]	ptg002313l	53.381	7.76E-87	289	83
KAB2966566.1_MAG:_FtsX-like_permease_family_protein_[Thermoanaerobaculia_bacterium]	ptg004986l	26.42	7.4E-20	95.9	80
KAB2966565.1_MAG:_ABC_transporter_permease_[Thermoanaerobaculia_bacterium]	ptg004986l	24.405	0.0000071	52.4	41
KAB2966564.1_MAG:_ABC_transporter_ATP-binding_protein_[Thermoanaerobaculia_bacterium]	ptg005920l	45.755	2E-57	200	88
KAB2966562.1_MAG:_DUF5117_domain-containing_protein,_partial_[Thermoanaerobaculia_bacterium]	ptg005976l	45.545	2.87E-118	362	65
KAB2966560.1_MAG:_HAMP_domain-containing_protein,_partial_[Thermoanaerobaculia_bacterium]	ptg005886l	27.397	2.57E-17	91.7	24
KAB2966549.1_MAG:_nucleotide_exchange_factor_GrpE_[Thermoanaerobaculia_bacterium]	ptg005922l	39.259	6.86E-15	76.3	62
KAB2966548.1_MAG:_molecular_chaperone_DnaK_[Thermoanaerobaculia_bacterium]	ptg005922l	53.205	0	631	99
KAB2966547.1_MAG:_molecular_chaperone_DnaJ_[Thermoanaerobaculia_bacterium]	ptg003680l	45.946	1.05E-69	241	98
KAB2966524.1_MAG:_magnesium/cobalt_transporter_CorA_[Thermoanaerobaculia_bacterium]	ptg003762l	27.09	1.86E-22	103	79
KAB2966522.1_MAG:_NupC/NupG_family_nucleoside_CNT_transporter_[Thermoanaerobaculia_bacterium]	ptg001925l	47.815	4.92E-98	324	96

KAB2966519.1_MAG:_serine/threonine_protein_kinase,_partial_[Thermoanaerobaculia_bacterium]	ptg001897l	40.8	5.89E-52	186	90
KAB2966517.1_MAG:_zinc-binding_dehydrogenase_[Thermoanaerobaculia_bacterium]	ptg004026l	33.745	3.19E-39	152	68
KAB2966513.1_MAG:_esterase_family_protein_[Thermoanaerobaculia_bacterium]	ptg003764l	25.728	9.78E-09	59.7	72
KAB2966494.1_MAG:_3-phosphoserine/phosphohydroxythreonine_transaminase_[Thermoanaerobaculia_bacterium]	ptg005318l	49.051	8.26E-106	345	100
KAB2966493.1_MAG:_isocitrate/isopropylmalate_dehydrogenase_family_protein_[Thermoanaerobaculia_bacterium]	ptg004623l	33.756	1.56E-45	171	99
KAB2966492.1_MAG:_hydroxymethylglutaryl-CoA_lyase_[Thermoanaerobaculia_bacterium]	ptg000685l	36.823	1.1E-32	131	91
KAB2966491.1_MAG:_CoA_transferase_[Thermoanaerobaculia_bacterium]	ptg004240l	39.74	1.48E-82	279	97
KAB2966490.1_MAG:_3-isopropylmalate_dehydratase_large_subunit_[Thermoanaerobaculia_bacterium]	ptg001715l	33.034	4.32E-49	183	93
KAB2966489.1_MAG:_3-isopropylmalate_dehydratase_[Thermoanaerobaculia_bacterium]	ptg000685l	32.773	2.43E-08	55.8	67
KAB2966468.1_MAG:_ATP-binding_cassette_domain-containing_protein_[Thermoanaerobaculia_bacterium]	ptg003947l	29.976	5.22E-49	188	64
KAB2966464.1_MAG:_peptidase_T_[Thermoanaerobaculia_bacterium]	ptg001690l	44.068	4.6E-113	368	99
KAB2966448.1_MAG:_FtsX-like_permease_family_protein_[Thermoanaerobaculia_bacterium]	ptg005920l	31.195	3.43E-22	103	69
KAB2966447.1_MAG:_ABC_transporter_permease_[Thermoanaerobaculia_bacterium]	ptg005920l	31.153	3.94E-25	112	69
KAB2966446.1_MAG:_ABC_transporter_ATP-binding_protein_[Thermoanaerobaculia_bacterium]	ptg001659l	58.108	3.66E-79	262	93
KAB2966445.1_MAG:_efflux_RND_transporter_periplasmic_adaptor_subunit_[Thermoanaerobaculia_bacterium]	ptg005906l	22.571	0.000000036	60.1	69
KAB2966420.1_MAG:_efflux_RND_transporter_permease_subunit_[Thermoanaerobaculia_bacterium]	ptg004798l	33.398	1.48E-136	459	98
KAB2966396.1_MAG:_cation_transporter_[Thermoanaerobaculia_bacterium]	ptg005301l	28.261	9.38E-16	81.3	77
KAB2966371.1_MAG:_TIGR00730_family_Rossman_fold_protein_[Thermoanaerobaculia_bacterium]	ptg003168l	43.75	4.54E-16	79.3	48
KAB2966370.1_MAG:_S-adenosylmethionine:tRNA_ribosyltransferase-isomerase,_partial_[Thermoanaerobaculia_bacterium]	ptg004256l	60.563	8.66E-23	94	76
KAB2966348.1_MAG:_tRNA_(adenosine(37)-N6)-threonylcarbamoyltransferase_complex_ATPase_subunit_type_1_TsaE_[Thermoanaerobaculia_bacterium]	ptg005375l	42.056	6.76E-16	76.6	75
KAB2966347.1_MAG:_3-hydroxybutyryl-CoA_dehydrogenase_[Thermoanaerobaculia_bacterium]	ptg002612l	39.502	1.69E-59	207	99
KAB2966346.1_MAG:_PhoH_family_protein_[Thermoanaerobaculia_bacterium]	ptg003708l	52.09	7.64E-84	279	95
KAB2966345.1_MAG:_rRNA_maturation_RNase_YbeY_[Thermoanaerobaculia_bacterium]	ptg004790l	34.483	0.000000257	52.4	56
KAB2966344.1_MAG:_HlyC/CorC_family_transporter_[Thermoanaerobaculia_bacterium]	ptg005005l	34.836	5.87E-35	141	56
KAB2966343.1_MAG:_GTPase_Era_[Thermoanaerobaculia_bacterium]	ptg002902l	37.321	1.49E-41	132	84
KAB2966342.1_MAG:_UDP-3-O-[3-hydroxymyristoyl]_N-acetylglucosamine_deacetylase,_partial_[Thermoanaerobaculia_bacterium]	ptg004510l	35.821	1.34E-37	145	84
KAB2966319.1_MAG:_transcriptional_repressor_NrdR_[Thermoanaerobaculia_bacterium]	ptg004151l	43.421	6.24E-25	103	94
KAB2966318.1_MAG:_endopeptidase_La,_partial_[Thermoanaerobaculia_bacterium]	ptg005527l	46.358	0	675	96
KAB2966317.1_MAG:_methionine_synthase_[Thermoanaerobaculia_bacterium]	ptg002021l	37.996	2.96E-161	537	89
KAB2966316.1_MAG:_methylenetetrahydrofolate_reductase_[NAD(P)H]_[Thermoanaerobaculia_bacterium]	ptg002052l	28.981	3.21E-35	139	95
KAB2966296.1_MAG:_hypothetical_protein_F9K18_05785_[Thermoanaerobaculia_bacterium]	LG07	41.423	3.93E-28	127	79
KAB2966275.1_MAG:_alpha/beta_fold_hydrolase_[Thermoanaerobaculia_bacterium]	ptg003863l	48.333	0.000000444	56.6	14

KAB2966273.1_MAG:_NAD-dependent_epimerase/dehydratase_family_protein_[Thermoanaerobaculia_bacterium]	ptg005035l	27.132	0.00000284	52.4	92
KAB2966257.1_MAG:_efflux_RND_transporter_permease_subunit_[Thermoanaerobaculia_bacterium]	ptg000732l	23.218	3.6E-39	163	82
KAB2966254.1_MAG:_winged_helix-turn-helix_transcriptional_regulator_[Thermoanaerobaculia_bacterium]	ptg005046l	42.857	1.44E-08	54.7	59
KAB2966252.1_MAG:_carbon-nitrogen_hydrolase_family_protein_[Thermoanaerobaculia_bacterium]	ptg003452l	31.579	3.39E-24	105	80
KAB2966236.1_MAG:_FAD-binding_oxidoreductase,_partial_[Thermoanaerobaculia_bacterium]	ptg001870l	33.036	0.00000285	49.7	58
KAB2966216.1_MAG:_transcriptional_repressor_[Thermoanaerobaculia_bacterium]	ptg003708l	33.871	5.81E-13	68.9	77
KAB2966214.1_MAG:_citrate_synthase_[Thermoanaerobaculia_bacterium]	ptg005518l	58.824	5.98E-165	517	99
KAB2966210.1_MAG:_ABC_transporter_ATP-binding_protein_[Thermoanaerobaculia_bacterium]	ptg001659l	46.222	6.13E-54	189	97
KAB2966190.1_MAG:_ABC_transporter_permease_subunit_[Thermoanaerobaculia_bacterium]	LG03	30.128	2.51E-14	76.3	59
KAB2966189.1_MAG:_ABC_transporter_ATP-binding_protein_[Thermoanaerobaculia_bacterium]	LG03	44.34	4.98E-51	184	68
KAB2966169.1_MAG:_6-phosphofructokinase_[Thermoanaerobaculia_bacterium]	ptg003223l	48.767	3.35E-75	256	99
KAB2966166.1_MAG:_50S_ribosomal_protein_L21_[Thermoanaerobaculia_bacterium]	ptg001290l	38.158	9.31E-11	60.5	72
KAB2966165.1_MAG:_50S_ribosomal_protein_L27_[Thermoanaerobaculia_bacterium]	ptg002505l	60	1.56E-25	101	99
KAB2966164.1_MAG:_GTPase_ObgE_[Thermoanaerobaculia_bacterium]	ptg002505l	51.916	6.57E-60	211	86
KAB2966163.1_MAG:_nicotinate_(nicotinamide)_nucleotide_adenylyltransferase_[Thermoanaerobaculia_bacterium]	ptg001008l	32.323	4.93E-23	100	86
KAB2966162.1_MAG:_ribosome_silencing_factor_[Thermoanaerobaculia_bacterium]	ptg004055l	38.776	4.4E-16	76.6	78
KAB2966144.1_MAG:_hypothetical_protein_F9K18_06010_[Thermoanaerobaculia_bacterium]	ptg004500l	27.857	0.00000694	54.3	15
KAB2966143.1_MAG:_hypothetical_protein_F9K18_06005_[Thermoanaerobaculia_bacterium]	ptg004500l	33.514	1.36E-14	82.8	20
KAB2966141.1_MAG:_sulfatase_[Thermoanaerobaculia_bacterium]	ptg003090l	39.167	4.33E-13	76.6	22
KAB2966114.1_MAG:_glycosyl_hydrolase,_partial_[Thermoanaerobaculia_bacterium]	ptg005375l	35.789	7.04E-132	444	73
KAB2966094.1_MAG:_type_II_toxin-antitoxin_system_VapC_family_toxin_[Thermoanaerobaculia_bacterium]	ptg003072l	34.351	1.19E-12	67	97
KAB2966093.1_MAG:_site-specific_DNA-methyltransferase,_partial_[Thermoanaerobaculia_bacterium]	ptg005299l	37.975	0.000000154	55.8	31
KAB2966087.1_MAG:_hypothetical_protein_F9K16_00265_[Thermoanaerobaculia_bacterium]	ptg001170l	34.307	1.57E-35	139	86
KAB2966073.1_MAG:_sensor_domain-containing_diguanylate_cyclase_[Thermoanaerobaculia_bacterium]	ptg005550l	41.758	3.74E-27	117	47
KAB2966070.1_MAG:_WYL_domain-containing_protein_[Thermoanaerobaculia_bacterium]	ptg004355l	25.333	6.61E-14	76.3	87
KAB2966069.1_MAG:_ATP-binding_protein_[Thermoanaerobaculia_bacterium]	ptg005375l	32.273	8.43E-23	105	47
KAB2966062.1_MAG:_hypothetical_protein_F9K16_00140_[Thermoanaerobaculia_bacterium]	ptg004670l	27.612	1.92E-35	145	73
KAB2966059.1_MAG:_thermonuclease_family_protein_[Thermoanaerobaculia_bacterium]	ptg002767l	36.17	2.49E-14	75.5	55
KAB2966057.1_MAG:_ParB/RepB/Spo0J_family_partition_protein_[Thermoanaerobaculia_bacterium]	ptg002467l	34.959	4.41E-09	61.6	39
KAB2966056.1_MAG:_TrbI/VirB10_family_protein_[Thermoanaerobaculia_bacterium]	ptg005137l	37.888	8.64E-30	112	37
KAB2966049.1_MAG:_P-type_conjugative_transfer_ATPase_TrbB_[Thermoanaerobaculia_bacterium]	ptg003168l	33.11	3.31E-27	116	80

KAB2966048.1_MAG:_type_IV_secretory_system_conjugative_DNA_transfer_family_protein_[Thermoanaerobaculia_bacterium]	ptg004058l	31.048	1.06E-20	102	57
KAB2966047.1_MAG:_relaxase_domain-containing_protein_[Thermoanaerobaculia_bacterium]	ptg005384l	31.795	2.69E-10	71.2	5
KAB2966039.1_MAG:_single-stranded_DNA-binding_protein_[Thermoanaerobaculia_bacterium]	ptg002505l	45.455	0.000000659	50.8	38
KAB2966036.1_MAG:_hypothetical_protein_F9K16_00005_[Thermoanaerobaculia_bacterium]	ptg004854l	28.231	1.11E-11	74.7	12
KAB2966034.1_MAG:_potassium_transporter_Kup_[Thermoanaerobaculia_bacterium]	ptg005550l	48.286	1.87E-103	266	86
KAB2966033.1_MAG:_potassium_transporter_Kup_[Thermoanaerobaculia_bacterium]	ptg005550l	46.91	2.02E-77	273	93
KAB2966031.1_MAG:_hypothetical_protein_F9K18_06065_[Thermoanaerobaculia_bacterium]	ptg003999l	57.143	1.88E-30	116	83
KAB2966020.1_MAG:_PAS_domain_S-box_protein_[Thermoanaerobaculia_bacterium]	ptg002738l	40.329	9.09E-36	150	31
KAB2966019.1_MAG:_DUF1446_domain-containing_protein_[Thermoanaerobaculia_bacterium]	ptg004500l	55.705	7.53E-168	527	98
KAB2966018.1_MAG:_hypothetical_protein_F9K18_06105_[Thermoanaerobaculia_bacterium]	ptg002612l	50	5.77E-23	95.5	83
KAB2966017.1_MAG:_hypothetical_protein_F9K18_06100_[Thermoanaerobaculia_bacterium]	ptg001245l	33.735	6.81E-15	77.8	62
KAB2966016.1_MAG:_HAD_hydrolase-like_protein_[Thermoanaerobaculia_bacterium]	ptg004085l	35.211	9.81E-25	107	78
KAB2966009.1_MAG:_P-type_conjugative_transfer_ATPase_TrbB_[Thermoanaerobaculia_bacterium]	ptg003168l	34.036	1.28E-35	140	92
KAB2965999.1_MAG:_nucleotidyl_transferase_AbiEii/AbiGii_toxin_family_protein_[Thermoanaerobaculia_bacterium]	ptg005254l	29.956	1.8E-09	62.4	73
KAB2965996.1_MAG:_DUF3732_domain-containing_protein_[Thermoanaerobaculia_bacterium]	ptg000619l	51.351	4.14E-13	77	11
KAB2965993.1_MAG:_DUF1738_domain-containing_protein_[Thermoanaerobaculia_bacterium]	ptg004058l	43.182	5.1E-67	230	96
KAB2965992.1_MAG:_single-stranded_DNA-binding_protein_[Thermoanaerobaculia_bacterium]	ptg005756l	35.345	2.39E-14	71.2	99
KAB2965989.1_MAG:_TraM_recognition_domain-containing_protein_[Thermoanaerobaculia_bacterium]	ptg004058l	27.586	5.87E-20	99.8	49
KAB2965988.1_MAG:_mercury(II)_reductase_[Thermoanaerobaculia_bacterium]	ptg005647l	32.135	3.32E-52	196	79
KAB2965976.1_MAG:_TrbI/VirB10_family_protein_[Thermoanaerobaculia_bacterium]	ptg005137l	42.675	1.67E-32	119	45
KAB2965975.1_MAG:_ParA_family_protein_[Thermoanaerobaculia_bacterium]	ptg001818l	30.508	3.09E-08	57.8	69
KAB2965974.1_MAG:_ParB/RepB/Spo0J_family_partition_protein_[Thermoanaerobaculia_bacterium]	ptg000372l	37.912	8.81E-18	87.4	60
KAB2965973.1_MAG:_cation_transporter_[Thermoanaerobaculia_bacterium]	ptg004758l	47.159	3.84E-21	95.1	78
KAB2965970.1_MAG:_P-II_family_nitrogen_regulator_[Thermoanaerobaculia_bacterium]	ptg003336l	44.248	4.15E-22	93.2	100
KAB2965969.1_MAG:_efflux_RND_transporter_permease_subunit_[Thermoanaerobaculia_bacterium]	ptg004670l	41.993	0	602	99
KAB2965968.1_MAG:_efflux_RND_transporter_periplasmic_adaptor_subunit_[Thermoanaerobaculia_bacterium]	ptg001766l	30.556	9.53E-10	62.8	63
KAB2966013.1_MAG:_sulfatase_[Thermoanaerobaculia_bacterium]	ptg004136l	36.047	1.06E-19	99	21
KAB2965966.1_MAG:_ATP-dependent_DNA_helicase_RecG_[Thermoanaerobaculia_bacterium]	ptg001995l	51.562	7.42E-94	323	54
KAB2965945.1_MAG:_DNA-directed_RNA_polymerase_subunit_beta'_[Thermoanaerobaculia_bacterium]	ptg002723l	50.429	0	1341	98
KAB2965944.1_MAG:_transcription_termination/antitermination_factor_NusG_[Thermoanaerobaculia_bacterium]	ptg002214l	49.143	4.33E-54	187	97
KAB2965943.1_MAG:_serine/threonine_protein_kinase,_partial_[Thermoanaerobaculia_bacterium]	ptg002986l	46.237	6.81E-54	161	81
KAB2965942.1_MAG:_elongation_factor_G_[Thermoanaerobaculia_bacterium]	ptg002658l	34.838	3.53E-121	402	98
KAB2965941.1_MAG:_elongation_factor_P_[Thermoanaerobaculia_bacterium]	LG03	37.569	1.65E-33	129	98

KAB2965940.1_MAG:_50S_ribosomal_protein_L17_[Thermoanaerobaculia_bacterium]	ptg004509l	51.724	5.01E-31	122	59
KAB2965939.1_MAG:_DNA-directed_RNA_polymerase_subunit_alpha_[Thermoanaerobaculia_bacterium]	ptg005964l	45.625	8.75E-72	244	97
KAB2965938.1_MAG:_30S_ribosomal_protein_S4_[Thermoanaerobaculia_bacterium]	ptg004509l	51.905	7.42E-63	214	100
KAB2965937.1_MAG:_30S_ribosomal_protein_S11_[Thermoanaerobaculia_bacterium]	ptg004509l	63.964	1.03E-42	153	80
KAB2965936.1_MAG:_30S_ribosomal_protein_S13_[Thermoanaerobaculia_bacterium]	ptg004509l	58.261	1.09E-39	144	91
KAB2965935.1_MAG:_50S_ribosomal_protein_L36_[Thermoanaerobaculia_bacterium]	ptg004564l	67.568	2.58E-10	55.5	100
KAB2965934.1_MAG:_translation_initiation_factor_IF-1_[Thermoanaerobaculia_bacterium]	ptg003290l	70.909	3.7E-21	88.2	76
KAB2965933.1_MAG:_type_I_methionyl_aminopeptidase_[Thermoanaerobaculia_bacterium]	ptg004895l	46.964	7.94E-69	233	99
KAB2965932.1_MAG:_adenylate_kinase_[Thermoanaerobaculia_bacterium]	ptg005723l	29.787	1.04E-16	80.9	95
KAB2965931.1_MAG:_preprotein_translocase_subunit_SecY_[Thermoanaerobaculia_bacterium]	ptg004509l	44.027	8.02E-109	357	97
KAB2965930.1_MAG:_50S_ribosomal_protein_L15_[Thermoanaerobaculia_bacterium]	ptg005891l	51.02	5.22E-38	140	99
KAB2965929.1_MAG:_50S_ribosomal_protein_L30_[Thermoanaerobaculia_bacterium]	ptg004509l	53.488	7.36E-08	50.1	67
KAB2965928.1_MAG:_30S_ribosomal_protein_S5_[Thermoanaerobaculia_bacterium]	ptg004509l	55.634	3.87E-46	164	86
KAB2965927.1_MAG:_50S_ribosomal_protein_L18_[Thermoanaerobaculia_bacterium]	ptg003734l	42.157	1.16E-17	81.3	80
KAB2965926.1_MAG:_50S_ribosomal_protein_L6_[Thermoanaerobaculia_bacterium]	ptg004509l	41.477	6.8E-38	141	98
KAB2965925.1_MAG:_30S_ribosomal_protein_S8_[Thermoanaerobaculia_bacterium]	ptg005254l	50.781	4.01E-36	134	97
KAB2965924.1_MAG:_type_Z_30S_ribosomal_protein_S14_[Thermoanaerobaculia_bacterium]	ptg005254l	74.074	0.000000997	47	44
KAB2965923.1_MAG:_50S_ribosomal_protein_L5_[Thermoanaerobaculia_bacterium]	ptg005254l	54.237	4.59E-62	211	91
KAB2965922.1_MAG:_50S_ribosomal_protein_L24_[Thermoanaerobaculia_bacterium]	ptg004509l	51.456	1.85E-26	105	96
KAB2965921.1_MAG:_50S_ribosomal_protein_L14_[Thermoanaerobaculia_bacterium]	ptg004509l	63.934	8.84E-37	135	100
KAB2965920.1_MAG:_30S_ribosomal_protein_S17_[Thermoanaerobaculia_bacterium]	ptg002047l	41.026	1.13E-16	76.6	84
KAB2965918.1_MAG:_50S_ribosomal_protein_L16_[Thermoanaerobaculia_bacterium]	ptg001624l	62.121	5.26E-50	174	95
KAB2965917.1_MAG:_30S_ribosomal_protein_S3_[Thermoanaerobaculia_bacterium]	ptg005314l	57.746	1.6E-81	268	98
KAB2965916.1_MAG:_50S_ribosomal_protein_L22_[Thermoanaerobaculia_bacterium]	ptg004509l	47.899	5.43E-23	95.9	99
KAB2965915.1_MAG:_30S_ribosomal_protein_S19_[Thermoanaerobaculia_bacterium]	ptg004509l	57.955	4.35E-28	109	91
KAB2965914.1_MAG:_50S_ribosomal_protein_L2_[Thermoanaerobaculia_bacterium]	ptg005891l	56.934	1.57E-87	288	99
KAB2965913.1_MAG:_50S_ribosomal_protein_L23_[Thermoanaerobaculia_bacterium]	ptg005891l	43.182	2.24E-10	58.9	92
KAB2965912.1_MAG:_50S_ribosomal_protein_L4_[Thermoanaerobaculia_bacterium]	ptg004509l	35.263	8.06E-30	119	92
KAB2965911.1_MAG:_50S_ribosomal_protein_L3_[Thermoanaerobaculia_bacterium]	ptg005314l	48.78	1.29E-54	190	98
KAB2965910.1_MAG:_30S_ribosomal_protein_S10_[Thermoanaerobaculia_bacterium]	ptg005314l	71.287	2.47E-43	153	96
KAB2965909.1_MAG:_elongation_factor_Tu_[Thermoanaerobaculia_bacterium]	ptg002214l	69.586	3.41E-156	491	99
KAB2965908.1_MAG:_elongation_factor_G_[Thermoanaerobaculia_bacterium]	ptg004438l	46.143	8.54E-178	566	98
KAB2965907.1_MAG:_30S_ribosomal_protein_S7_[Thermoanaerobaculia_bacterium]	ptg003722l	46.452	1.23E-45	162	99
KAB2965906.1_MAG:_30S_ribosomal_protein_S12_[Thermoanaerobaculia_bacterium]	ptg003232l	72.131	1.74E-55	189	98

KAB2965905.1_MAG:_DNA-directed_RNA_polymerase_subunit_beta_[Thermoanaerobaculia_bacterium]	ptg002278l	51.696	0	1081	90
KAB2965904.1_MAG:_50S_ribosomal_protein_L7/L12_[Thermoanaerobaculia_bacterium]	ptg002278l	52	2.28E-23	97.4	100
KAB2965903.1_MAG:_50S_ribosomal_protein_L10_[Thermoanaerobaculia_bacterium]	ptg002278l	37.267	3.41E-24	101	93
KAB2965902.1_MAG:_50S_ribosomal_protein_L1_[Thermoanaerobaculia_bacterium]	ptg002278l	49.333	3.07E-69	233	96
KAB2965901.1_MAG:_50S_ribosomal_protein_L11_[Thermoanaerobaculia_bacterium]	ptg003726l	63.121	1.83E-52	181	100
KAB2965899.1_MAG:_hypothetical_protein_F9K16_00515,_partial_[Thermoanaerobaculia_bacterium]	ptg002214l	63.158	1.16E-10	56.6	97
KAB2965898.1_MAG:_DNA_gyrase_subunit_A_[Thermoanaerobaculia_bacterium]	ptg003780l	47.393	0	730	96
KAB2965896.1_MAG:_DNA_topoisomerase_(ATP-hydrolyzing)_subunit_B_[Thermoanaerobaculia_bacterium]	ptg000372l	61.051	0	652	99
KAB2965895.1_MAG:_protein_kinase,_partial_[Thermoanaerobaculia_bacterium]	ptg003378l	28.231	3.22E-12	73.6	56
KAB2965893.1_MAG:_catalase/peroxidase_HPI_[Thermoanaerobaculia_bacterium]	ptg004476l	68.214	0	932	97
KAB2965876.1_MAG:_spermidine_synthase,_partial_[Thermoanaerobaculia_bacterium]	ptg001766l	33.969	1.06E-36	155	44
KAB2965872.1_MAG:_ABC_transporter_ATP-binding_protein_[Thermoanaerobaculia_bacterium]	ptg004997l	40.436	3.74E-112	374	93
KAB2965871.1_MAG:_mercuric_reductase_[Thermoanaerobaculia_bacterium]	ptg003910l	50.495	2.8E-132	427	96
KAB2965870.1_MAG:_TVP38/TMEM64_family_protein_[Thermoanaerobaculia_bacterium]	ptg005647l	33.594	0.000000151	55.8	51
KAB2965869.1_MAG:_aminotransferase_class_V-fold_PLP-dependent_enzyme_[Thermoanaerobaculia_bacterium]	LG16	27.389	0.00000124	55.5	30
KAB2965868.1_MAG:_carboxymuconolactone_decarboxylase_family_protein_[Thermoanaerobaculia_bacterium]	ptg002246l	45.556	1.07E-46	167	95
KAB2965867.1_MAG:_hypothetical_protein_F9K16_00845_[Thermoanaerobaculia_bacterium]	ptg003290l	45.763	1.21E-09	56.2	68
KAB2965864.1_MAG:_RNA_polymerase_sigma_factor_[Thermoanaerobaculia_bacterium]	ptg003441l	29.891	2.8E-12	68.2	90
KAB2965861.1_MAG:_methyltransferase_domain-containing_protein_[Thermoanaerobaculia_bacterium]	ptg001690l	61.919	2.69E-118	381	90
KAB2965860.1_MAG:_radical_SAM/Cys-rich_domain_protein_[Thermoanaerobaculia_bacterium]	ptg005967l	54.826	6.13E-89	295	76
KAB2965859.1_MAG:_DUF2064_domain-containing_protein_[Thermoanaerobaculia_bacterium]	ptg001353l	35.088	1.94E-30	130	42
KAB2965855.1_MAG:_nitrate_reductase_subunit_beta_[Thermoanaerobaculia_bacterium]	ptg002175l	38	1.72E-13	77.4	19
KAB2965851.1_MAG:_acyl-CoA_dehydrogenase_[Thermoanaerobaculia_bacterium]	ptg004179l	28.533	5.3E-37	147	88
KAB2965850.1_MAG:_sigma-70_family_RNA_polymerase_sigma_factor_[Thermoanaerobaculia_bacterium]	ptg002928l	36.145	1.34E-14	74.3	87
KAB2965848.1_MAG:_YceI_family_protein_[Thermoanaerobaculia_bacterium]	ptg002928l	25.909	2.41E-12	68.9	98
KAB2965844.1_MAG:_tetratricopeptide_repeat_protein,_partial_[Thermoanaerobaculia_bacterium]	LG25	33.208	5.65E-18	89.7	92
KAB2965821.1_MAG:_molybdopterin_oxidoreductase_family_protein_[Thermoanaerobaculia_bacterium]	ptg005451l	32.806	1.67E-98	339	90
KAB2965820.1_MAG:_4Fe-4S_dicluster_domain-containing_protein_[Thermoanaerobaculia_bacterium]	ptg002175l	33.758	1.32E-19	90.1	71
KAB2965814.1_MAG:_heavy_metal_translocating_P-type_ATPase_[Thermoanaerobaculia_bacterium]	ptg003880l	33.777	2.07E-57	219	69
KAB2965805.1_MAG:_methyltransferase_domain-containing_protein_[Thermoanaerobaculia_bacterium]	ptg002534l	33.043	8.25E-11	64.7	52
KAB2965799.1_MAG:_efflux_RND_transporter_periplasmic_adaptor_subunit_[Thermoanaerobaculia_bacterium]	ptg001766l	28.046	8.62E-49	184	88
KAB2965798.1_MAG:_efflux_RND_transporter_permease_subunit_[Thermoanaerobaculia_bacterium]	ptg004670l	32.825	1.03E-165	547	98
KAB2965796.1_MAG:_HD_domain-containing_protein_[Thermoanaerobaculia_bacterium]	ptg005839l	33.69	6.71E-20	95.1	53
KAB2965795.1_MAG:_trypsin-like_serine_protease_[Thermoanaerobaculia_bacterium]	ptg004186l	44.904	5.23E-71	246	75

KAB2965794.1_MAG:_ATP-binding_protein_[Thermoanaerobaculia_bacterium]	ptg003334l	32.237	1.06E-21	97.8	58
KAB2965792.1_MAG:_hypothetical_protein_F9K18_06265_[Thermoanaerobaculia_bacterium]	ptg004510l	29.31	1.54E-31	90.1	94
KAB2965790.1_MAG:_DUF1573_domain-containing_protein_[Thermoanaerobaculia_bacterium]	ptg003830l	22.65	0.000000144	57	66
KAB2965789.1_MAG:_ribonuclease_PH_[Thermoanaerobaculia_bacterium]	ptg003928l	50.435	1.84E-25	109	42
KAB2965773.1_MAG:_SDR_family_oxidoreductase,_partial_[Thermoanaerobaculia_bacterium]	ptg003220l	50	1.69E-109	358	98
KAB2965771.1_MAG:_alpha/beta_fold_hydrolase_[Thermoanaerobaculia_bacterium]	ptg005092l	42.254	0.00000609	51.6	23
KAB2965768.1_MAG:_hypothetical_protein_F9K16_01120_[Thermoanaerobaculia_bacterium]	ptg002660l	26.984	0.00000181	54.3	81
KAB2965767.1_MAG:_diguanylate_cyclase_[Thermoanaerobaculia_bacterium]	LG04	40.625	5.59E-23	106	34
KAB2965766.1_MAG:_OmpA_family_protein_[Thermoanaerobaculia_bacterium]	ptg003829l	39.516	3.29E-09	62.4	36
KAB2965765.1_MAG:_EAL_domain-containing_protein_[Thermoanaerobaculia_bacterium]	ptg005265l	37.788	2.11E-61	226	65
KAB2965763.1_MAG:_PAS_domain_S-box_protein_[Thermoanaerobaculia_bacterium]	ptg002738l	32.099	1.68E-49	198	37
KAB2965761.1_MAG:_SUMF1/EgtB/PvdO_family_nonheme_iron_enzyme_[Thermoanaerobaculia_bacterium]	ptg001897l	35.472	2.02E-29	129	54
KAB2965759.1_MAG:_hypothetical_protein_F9K16_01075_[Thermoanaerobaculia_bacterium]	ptg004798l	27.66	5.61E-09	64.7	23
KAB2965749.1_MAG:_azurin_[Thermoanaerobaculia_bacterium]	ptg003378l	32.787	8.61E-14	71.2	74
KAB2965748.1_MAG:_BlaI/MecI/CopY_family_transcriptional_regulator_[Thermoanaerobaculia_bacterium]	ptg004297l	40	0.00000819	47.4	45
KAB2965744.1_MAG:_glycosyltransferase_family_2_protein_[Thermoanaerobaculia_bacterium]	ptg003431l	51.695	2.88E-72	242	97
KAB2965742.1_MAG:_MoxR_family_ATPase_[Thermoanaerobaculia_bacterium]	ptg004759l	46.309	3.35E-64	223	93
KAB2965741.1_MAG:_DUF58_domain-containing_protein,_partial_[Thermoanaerobaculia_bacterium]	ptg002612l	23.636	0.000000186	56.6	48
KAB2965712.1_MAG:_type_I_glyceraldehyde-3-phosphate_dehydrogenase_[Thermoanaerobaculia_bacterium]	ptg002223l	49.401	4.13E-90	298	98
KAB2965711.1_MAG:_glycosyl_hydrolase,_partial_[Thermoanaerobaculia_bacterium]	ptg005375l	35.226	1.11E-134	447	90
KAB2965710.1_MAG:_nuclease_[Thermoanaerobaculia_bacterium]	ptg005341l	27.217	2.96E-20	101	64
KAB2965709.1_MAG:_diguanylate_cyclase_[Thermoanaerobaculia_bacterium]	ptg005550l	49.515	1.11E-23	110	19
KAB2965708.1_MAG:_hypothetical_protein_F9K16_01215_[Thermoanaerobaculia_bacterium]	ptg002660l	23.636	0.00000171	55.8	40
KAB2965707.1_MAG:_flavodoxin-dependent_(E)-4-hydroxy-3-methylbut-2-enyl-diphosphate_synthase_[Thermoanaerobaculia_bacterium]	ptg003703l	36.723	9.72E-46	173	84
KAB2965706.1_MAG:_flavin_reductase_[Thermoanaerobaculia_bacterium]	ptg003548l	30.539	1.14E-08	56.6	97
KAB2965705.1_MAG:_RNA_methyltransferase_[Thermoanaerobaculia_bacterium]	ptg001291l	34.675	1.12E-36	145	82
KAB2965701.1_MAG:_sigma-70_family_RNA_polymerase_sigma_factor_[Thermoanaerobaculia_bacterium]	ptg004340l	40.341	6.81E-23	99	88
KAB2965700.1_MAG:_serine/threonine_protein_kinase_[Thermoanaerobaculia_bacterium]	ptg003839l	38.838	4.07E-40	164	37
KAB2965699.1_MAG:_hypothetical_protein_F9K16_01170_[Thermoanaerobaculia_bacterium]	ptg005443l	26.291	2.17E-10	66.2	57
KAB2965698.1_MAG:_beta-ketoacyl_synthase_[Thermoanaerobaculia_bacterium]	ptg003327l	30.622	7.18E-16	85.1	38
KAB2965697.1_MAG:_type_I_glyceraldehyde-3-phosphate_dehydrogenase_[Thermoanaerobaculia_bacterium]	ptg002223l	36.556	1.13E-63	222	96
KAB2965696.1_MAG:_DUF1015_domain-containing_protein,_partial_[Thermoanaerobaculia_bacterium]	ptg001693l	39.713	6.13E-40	149	89
KAB2965694.1_MAG:_peptide-methionine_(S)-S-oxide_reductase_MsrA,_partial_[Thermoanaerobaculia_bacterium]	ptg003471l	44.554	8.99E-16	75.1	85

KAB2965692.1_MAG:_DUF1295_domain-containing_protein_[Thermoanaerobaculia_bacterium]	ptg002738l	42.478	1.43E-09	62	42
KAB2965691.1_MAG:_GAF_domain-containing_protein_[Thermoanaerobaculia_bacterium]	ptg003863l	31.602	8.96E-23	108	31
KAB2965690.1_MAG:_response_regulator_transcription_factor_[Thermoanaerobaculia_bacterium]	ptg005527l	33.971	4.35E-24	103	99
KAB2965674.1_MAG:_bifunctional_3,4-dihydroxy-2-butanone-4-phosphate_synthase/GTP_cyclohydrolase_II_[Thermoanaerobaculia_bacterium]	ptg003075l	48.75	8.89E-111	360	99
KAB2965673.1_MAG:_NAD(+)/NADH_kinase_[Thermoanaerobaculia_bacterium]	ptg002867l	38.428	2.76E-39	149	79
KAB2965672.1_MAG:_polyprenyl_synthetase_family_protein_[Thermoanaerobaculia_bacterium]	ptg003058l	49.091	1.33E-25	109	84
KAB2965668.1_MAG:_sulfatase_[Thermoanaerobaculia_bacterium]	ptg003382l	33.058	0.00000959	52.4	24
KAB2965667.1_MAG:_sulfatase-like_hydrolase/transferase_[Thermoanaerobaculia_bacterium]	ptg004126l	24.777	3.32E-14	79.3	84
KAB2965665.1_MAG:_sulfatase_[Thermoanaerobaculia_bacterium]	ptg003090l	25.964	3.85E-12	73.2	68
KAB2965664.1_MAG:_BamA/TamA_family_outer_membrane_protein_[Thermoanaerobaculia_bacterium]	ptg002436l	25.97	4.23E-18	94.4	36
KAB2965662.1_MAG:_TlyA_family_RNA_methyltransferase_[Thermoanaerobaculia_bacterium]	ptg004075l	42.798	4.35E-42	156	93
KAB2965660.1_MAG:_1-deoxy-D-xylulose-5-phosphate_synthase_[Thermoanaerobaculia_bacterium]	ptg003058l	43.499	1.43E-165	529	95
KAB2965658.1_MAG:_CDP-glucose_4,6-dehydratase_[Thermoanaerobaculia_bacterium]	ptg006001l	23.81	1.01E-11	70.9	83
KAB2965657.1_MAG:_dTDP-4-keto-6-deoxy-D-glucose_epimerase_[Thermoanaerobaculia_bacterium]	ptg003827l	36.686	3.56E-27	110	93
KAB2965655.1_MAG:_protein_kinase,_partial_[Thermoanaerobaculia_bacterium]	ptg005425l	36.296	3.33E-35	143	58
KAB2965648.1_MAG:_nucleoside_diphosphate_kinase_regulator_[Thermoanaerobaculia_bacterium]	ptg005309l	29.545	0.000000211	52	65
KAB2965646.1_MAG:_HAMP_domain-containing_protein_[Thermoanaerobaculia_bacterium]	ptg004779l	28.864	1.8E-29	127	84
KAB2965645.1_MAG:_response_regulator_[Thermoanaerobaculia_bacterium]	ptg004779l	44.724	1.22E-33	130	89
KAB2965638.1_MAG:_type_I_restriction_endonuclease_subunit_R_[Thermoanaerobaculia_bacterium]	ptg005254l	37.97	1.33E-163	543	78
KAB2965633.1_MAG:_hypothetical_protein_F9K16_01375_[Thermoanaerobaculia_bacterium]	ptg005254l	26.667	3.98E-28	121	75
KAB2965632.1_MAG:_SAM-dependent_DNA_methyltransferase_[Thermoanaerobaculia_bacterium]	ptg005254l	44.612	3.1E-143	465	86
KAB2965631.1_MAG:_serine/threonine_protein_kinase_[Thermoanaerobaculia_bacterium]	ptg003585l	36.066	2.03E-34	139	65
KAB2965630.1_MAG:_tyrosine-type_recombinase/integrase_[Thermoanaerobaculia_bacterium]	ptg005274l	35.211	7.03E-10	61.6	66
KAB2965626.1_MAG:_TonB-dependent_receptor,_partial_[Thermoanaerobaculia_bacterium]	ptg005711l	41.984	1.25E-162	525	98
KAB2965622.1_MAG:_Na+/H+_antiporter_NhaA_[Thermoanaerobaculia_bacterium]	ptg003548l	48.726	5.43E-82	277	79
KAB2965619.1_MAG:_phosphopyruvate_hydratase_[Thermoanaerobaculia_bacterium]	ptg004466l	59.145	8.58E-158	497	97
KAB2965604.1_MAG:_cytochrome_c_[Thermoanaerobaculia_bacterium]	ptg002453l	32.278	1.36E-17	84	67
KAB2965603.1_MAG:_cytochrome_c_oxidase_subunit_3_family_protein_[Thermoanaerobaculia_bacterium]	ptg002453l	48.182	1.4E-20	92	94
KAB2965601.1_MAG:_4Fe-4S_dicluster_domain-containing_protein,_partial_[Thermoanaerobaculia_bacterium]	ptg001690l	55.749	1.08E-96	330	99
KAB2965600.1_MAG:_hydrogenase_[Thermoanaerobaculia_bacterium]	ptg002453l	57.468	1.14E-139	446	85
KAB2965599.1_MAG:_DUF3341_domain-containing_protein_[Thermoanaerobaculia_bacterium]	ptg001690l	35.294	1.13E-21	95.1	100
KAB2965598.1_MAG:_hypothetical_protein_F9K16_01585_[Thermoanaerobaculia_bacterium]	ptg001690l	32.01	6.93E-33	134	93
KAB2965596.1_MAG:_SCO_family_protein_[Thermoanaerobaculia_bacterium]	ptg002453l	29.148	1E-30	124	80

KAB2965595.1_MAG:_cytochrome_c_oxidase_subunit_II_[Thermoanaerobaculia_bacterium]	ptg002453l	35.494	6.47E-52	187	95
KAB2965594.1_MAG:_cytochrome_c_oxidase_subunit_I_[Thermoanaerobaculia_bacterium]	ptg001690l	58.835	0	601	97
KAB2965590.1_MAG:_cob(I)yrinic_acid_a,c-diamide_adenosyltransferase_[Thermoanaerobaculia_bacterium]	ptg003839l	38.983	3.93E-17	82	58
KAB2965588.1_MAG:_type_IV-A_pilus_assembly_ATPase_PilB_[Thermoanaerobaculia_bacterium]	ptg000568l	42.882	2.7E-145	468	100
KAB2965587.1_MAG:_glycosyltransferase_family_9_protein_[Thermoanaerobaculia_bacterium]	ptg004974l	30.114	2.02E-26	114	98
KAB2965586.1_MAG:_adenylyltransferase/cytidyltransferase_family_protein_[Thermoanaerobaculia_bacterium]	ptg002916l	50	4.73E-23	97.8	71
KAB2965585.1_MAG:_sugar_kinase_[Thermoanaerobaculia_bacterium]	ptg002916l	36.697	1.08E-21	100	89
KAB2965584.1_MAG:_sigma-70_family_RNA_polymerase_sigma_factor_[Thermoanaerobaculia_bacterium]	ptg004479l	29.143	1.03E-20	93.2	83
KAB2965581.1_MAG:_peptidoglycan_DD-metalloendopeptidase_family_protein_[Thermoanaerobaculia_bacterium]	ptg000819l	38.785	2.6E-36	146	48
KAB2965579.1_MAG:_ATP-binding_cassette_domain-containing_protein_[Thermoanaerobaculia_bacterium]	ptg003947l	37.179	4.45E-41	162	43
KAB2965578.1_MAG:_glycosyltransferase_family_2_protein_[Thermoanaerobaculia_bacterium]	ptg005234l	28.922	3.27E-12	69.3	82
KAB2965576.1_MAG:_glycosyltransferase_family_2_protein,_partial_[Thermoanaerobaculia_bacterium]	ptg002123l	28.571	0.00000076	53.9	77
KAB2965571.1_MAG:_serine/threonine_protein_kinase,_partial_[Thermoanaerobaculia_bacterium]	ptg004928l	33.852	1.67E-27	119	57
KAB2965570.1_MAG:_sigma-70_family_RNA_polymerase_sigma_factor_[Thermoanaerobaculia_bacterium]	ptg004340l	39.037	4.12E-19	87.8	97
KAB2965568.1_MAG:_VWA_domain-containing_protein,_partial_[Thermoanaerobaculia_bacterium]	ptg004896l	40.196	1.19E-22	87	54
KAB2965548.1_MAG:_LPS_export_ABC_transporter_ATP-binding_protein_[Thermoanaerobaculia_bacterium]	ptg005709l	55.042	4.05E-82	271	97
KAB2965547.1_MAG:_RNA_polymerase_factor_sigma-54_[Thermoanaerobaculia_bacterium]	ptg003787l	34.096	1.75E-63	228	96
KAB2965545.1_MAG:_PTS_sugar_transporter_subunit_IIA_[Thermoanaerobaculia_bacterium]	ptg001366l	39.175	1.59E-17	82	61
KAB2965543.1_MAG:_RNase_adapter_RapZ_[Thermoanaerobaculia_bacterium]	ptg005611l	41.522	1.78E-59	208	96
KAB2965541.1_MAG:_HPr_family_phosphocarrier_protein_[Thermoanaerobaculia_bacterium]	ptg005611l	44.318	1.99E-15	72.8	97
KAB2965540.1_MAG:_phosphoenolpyruvate--protein_phosphotransferase_[Thermoanaerobaculia_bacterium]	ptg002723l	36.907	5.83E-94	320	96
KAB2965539.1_MAG:_cupin_domain-containing_protein_[Thermoanaerobaculia_bacterium]	ptg002270l	31.897	0.000000011	55.1	96
KAB2965537.1_MAG:_tRNA_(N6-isopentenyl_adenosine(37)-C2)-methylthiotransferase_MiaB_[Thermoanaerobaculia_bacterium]	ptg005772l	39.863	2.46E-97	324	93
KAB2965536.1_MAG:_ParB/RepB/Spo0J_family_partition_protein_[Thermoanaerobaculia_bacterium]	ptg001818l	39.459	3.74E-19	91.3	61
KAB2965535.1_MAG:_ParA_family_protein_[Thermoanaerobaculia_bacterium]	ptg003829l	39.6	1.39E-55	195	99
KAB2965534.1_MAG:_bifunctional_nuclease_family_protein_[Thermoanaerobaculia_bacterium]	ptg003247l	42.857	2.42E-24	102	75
KAB2965533.1_MAG:_6-carboxytetrahydropterin_synthase_[Thermoanaerobaculia_bacterium]	ptg002700l	47.368	3.75E-09	57.4	39
KAB2965531.1_MAG:_DNA_polymerase/3'-5'_exonuclease_PolX_[Thermoanaerobaculia_bacterium]	ptg005756l	40.28	8.72E-118	388	97
KAB2965530.1_MAG:_methionine_adenosyltransferase_[Thermoanaerobaculia_bacterium]	ptg005611l	63.271	5.66E-146	461	94
KAB2965527.1_MAG:_sulfatase_[Thermoanaerobaculia_bacterium]	ptg000693l	24.667	1E-11	71.6	81
KAB2965526.1_MAG:_electron_transfer_flavoprotein_subunit_alpha/FixB_family_protein_[Thermoanaerobaculia_bacterium]	LG19	60.87	2.48E-18	89.7	59

KAB2965525.1_MAG:_electron_transfer_flavoprotein_subunit_beta/FixA_family_protein_[Thermoanaerobaculia_bacterium]	ptg005554l	33.761	6.81E-22	98.6	88
KAB2965524.1_MAG:_hypothetical_protein_F9K16_01620_[Thermoanaerobaculia_bacterium]	ptg005341l	41.441	0.000000213	55.5	43
KAB2965523.1_MAG:_protein_TolR_[Thermoanaerobaculia_bacterium]	ptg005341l	38.462	1.82E-20	89.7	89
KAB2965503.1_MAG:_glycosyltransferase_family_2_protein_[Thermoanaerobaculia_bacterium]	ptg002303l	34.731	0.000000568	53.5	71
KAB2965494.1_MAG:_hypothetical_protein_F9K16_01795_[Thermoanaerobaculia_bacterium]	ptg004711l	40.23	7.46E-08	53.9	55
KAB2965490.1_MAG:_DNA_repair_protein_RadC_[Thermoanaerobaculia_bacterium]	ptg002199l	35.211	7.85E-32	126	85
KAB2965485.1_MAG:_hypothetical_protein_F9K16_01750,_partial_[Thermoanaerobaculia_bacterium]	ptg004854l	30.619	3.47E-22	110	6
KAB2965498.1_MAG:_hypothetical_protein_F9K18_06530_[Thermoanaerobaculia_bacterium]	ptg002660l	29.532	2.4E-10	67	81
KAB2965484.1_MAG:_proline--tRNA_ligase_[Thermoanaerobaculia_bacterium]	ptg003673l	37.209	6.17E-67	179	87
KAB2965482.1_MAG:_3-hydroxybutyryl-CoA_dehydrogenase_[Thermoanaerobaculia_bacterium]	ptg002612l	47.857	2.9E-70	239	98
KAB2965455.1_MAG:_NADH-quinone_oxidoreductase_subunit_NuoH_[Thermoanaerobaculia_bacterium]	ptg004143l	37.618	9.08E-63	219	95
KAB2965453.1_MAG:_MBL_fold_metallo-hydrolase,_partial_[Thermoanaerobaculia_bacterium]	ptg003250l	28.488	0.00000262	55.1	25
KAB2965452.1_MAG:_tRNA_(adenosine(37)-N6)-dimethylallyltransferase_MiaA_[Thermoanaerobaculia_bacterium]	ptg003471l	39.51	6.89E-60	209	93
KAB2965449.1_MAG:_CDP-alcohol_phosphatidyltransferase_family_protein_[Thermoanaerobaculia_bacterium]	ptg005527l	32.778	3.05E-14	73.6	95
KAB2965448.1_MAG:_AI-2E_family_transporter_[Thermoanaerobaculia_bacterium]	LG04	28.736	4.62E-10	38.9	38
KAB2965447.1_MAG:_DNA_polymerase_III_subunit_alpha_[Thermoanaerobaculia_bacterium]	ptg003999l	37.087	0	747	99
KAB2965444.1_MAG:_16S_rRNA_(guanine(966)-N(2))-methyltransferase_RsmD_[Thermoanaerobaculia_bacterium]	ptg004885l	34.286	1.04E-17	84	71
KAB2965443.1_MAG:_redox-sensing_transcriptional_repressor_Rex_[Thermoanaerobaculia_bacterium]	ptg005030l	35.498	1.44E-28	117	95
KAB2965442.1_MAG:_NADH-quinone_oxidoreductase_subunit_N_[Thermoanaerobaculia_bacterium]	ptg004143l	34.375	1.23E-35	145	59
KAB2965441.1_MAG:_NADH-quinone_oxidoreductase_subunit_M_[Thermoanaerobaculia_bacterium]	ptg004143l	42.159	2.36E-89	304	90
KAB2965440.1_MAG:_NADH-quinone_oxidoreductase_subunit_L_[Thermoanaerobaculia_bacterium]	ptg005338l	39.934	2.47E-97	332	91
KAB2965439.1_MAG:_NADH-quinone_oxidoreductase_subunit_NuoK_[Thermoanaerobaculia_bacterium]	ptg004143l	40.777	0.000000336	50.1	99
KAB2965437.1_MAG:_molybdopterin-dependent_oxidoreductase_[Thermoanaerobaculia_bacterium]	ptg005338l	42.063	1.87E-37	121	36
KAB2965436.1_MAG:_NADH-quinone_oxidoreductase_subunit_NuoF_[Thermoanaerobaculia_bacterium]	ptg005451l	44.935	2.3E-90	303	89
KAB2965435.1_MAG:_NADH-quinone_oxidoreductase_subunit_A_[Thermoanaerobaculia_bacterium]	ptg005338l	32.967	1.52E-11	63.5	70
KAB2965461.1_MAG:_aldehyde_dehydrogenase_[Thermoanaerobaculia_bacterium]	ptg003168l	40.557	5.02E-55	197	96
KAB2965459.1_MAG:_phosphoglycerate_kinase_[Thermoanaerobaculia_bacterium]	ptg003928l	44.591	3.95E-88	295	94
KAB2965458.1_MAG:_triose-phosphate_isomerase_[Thermoanaerobaculia_bacterium]	ptg003971l	49.59	5.66E-42	156	91
KAB2965417.1_MAG:_glutamine--fructose-6-phosphate_transaminase_(isomerizing)_[Thermoanaerobaculia_bacterium]	ptg002878l	46.624	2.19E-160	512	100

KAB2965416.1_MAG:_AAA_family_ATPase_[Thermoanaerobaculia_bacterium]	ptg005204l	40.787	7.22E-122	406	86
KAB2965415.1_MAG:_GNAT_family_N-acetyltransferase_[Thermoanaerobaculia_bacterium]	ptg002303l	35	0.000000923	52	54
KAB2965414.1_MAG:_hypothetical_protein_F9K18_06595,_partial_[Thermoanaerobaculia_bacterium]	ptg004564l	43.299	2.77E-19	85.9	75
KAB2965408.1_MAG:_phosphoribosylformylglycinamidine_cyclo-ligase_[Thermoanaerobaculia_bacterium]	ptg004520l	41.026	1.29E-73	251	96
KAB2965403.1_MAG:_phosphoribosylglycinamide_formyltransferase_[Thermoanaerobaculia_bacterium]	LG04	44.103	2.69E-34	132	95
KAB2965402.1_MAG:_tryptophanase_[Thermoanaerobaculia_bacterium]	ptg004361l	54.084	6.73E-150	476	98
KAB2965400.1_MAG:_Crp/Fnr_family_transcriptional_regulator_[Thermoanaerobaculia_bacterium]	ptg005331l	25.962	9.95E-18	85.5	89
KAB2965399.1_MAG:_azurin_[Thermoanaerobaculia_bacterium]	ptg003378l	44.444	2.03E-13	70.1	54
KAB2965395.1_MAG:_protoheme_IX_farnesyltransferase_[Thermoanaerobaculia_bacterium]	ptg000340l	40.909	3.58E-28	118	73
KAB2965394.1_MAG:_SCO_family_protein_[Thermoanaerobaculia_bacterium]	ptg000340l	36.667	7.62E-19	87.4	74
KAB2965392.1_MAG:_HD_domain-containing_protein_[Thermoanaerobaculia_bacterium]	ptg004242l	36.242	1.16E-48	179	83
KAB2965412.1_MAG:_peptide_MFS_transporter_[Thermoanaerobaculia_bacterium]	ptg003971l	31.628	3.9E-13	76.3	39
KAB2965410.1_MAG:_acyl-CoA_carboxylase_subunit_beta,_partial_[Thermoanaerobaculia_bacterium]	ptg002612l	48.975	9.86E-129	416	90
KAB2965368.1_MAG:_M20_family_metallopeptidase_[Thermoanaerobaculia_bacterium]	ptg000732l	29.798	1.33E-39	154	98
KAB2965366.1_MAG:_aconitate_hydratase_[Thermoanaerobaculia_bacterium]	ptg000685l	31.84	4.25E-32	137	59
KAB2965362.1_MAG:_D-alanine--D-alanine_ligase_[Thermoanaerobaculia_bacterium]	ptg004510l	32.271	3.29E-19	92.4	73
KAB2965360.1_MAG:_ABC_transporter_substrate-binding_protein_[Thermoanaerobaculia_bacterium]	ptg003947l	35.231	2.45E-37	144	94
KAB2965359.1_MAG:_phosphoglycolate_phosphatase_[Thermoanaerobaculia_bacterium]	ptg004584l	28.369	1.61E-09	49.3	57
KAB2965358.1_MAG:_M1_family_metallopeptidase_[Thermoanaerobaculia_bacterium]	ptg004724l	29.047	2.63E-33	143	49
KAB2965357.1_MAG:_cytidine_deaminase_[Thermoanaerobaculia_bacterium]	ptg000909l	49.612	1.11E-25	104	95
KAB2965356.1_MAG:_PP2C_family_protein-serine/threonine_phosphatase_[Thermoanaerobaculia_bacterium]	ptg004476l	29.536	1.52E-15	85.5	29
KAB2965355.1_MAG:_TerC_family_protein_[Thermoanaerobaculia_bacterium]	LG12	50	1.67E-10	65.9	39
KAB2965354.1_MAG:_ADP-forming_succinate--CoA_ligase_subunit_beta_[Thermoanaerobaculia_bacterium]	ptg002316l	50.263	6.41E-110	357	95
KAB2965353.1_MAG:_succinate--CoA_ligase_subunit_alpha_[Thermoanaerobaculia_bacterium]	ptg002316l	60.554	2.31E-112	360	99
KAB2965351.1_MAG:_peroxiredoxin_[Thermoanaerobaculia_bacterium]	ptg004538l	31.405	6.99E-13	69.3	64
KAB2965350.1_MAG:_nucleoside-diphosphate_kinase_[Thermoanaerobaculia_bacterium]	ptg002916l	47.328	2.41E-34	129	94
KAB2965347.1_MAG:_glycosyltransferase_family_4_protein_[Thermoanaerobaculia_bacterium]	ptg004384l	36.364	2.1E-13	79.7	13
KAB2965346.1_MAG:_crotonase_[Thermoanaerobaculia_bacterium]	ptg002612l	38.095	3.98E-45	165	96
KAB2965345.1_MAG:_TlpA_family_protein_disulfide_reductase_[Thermoanaerobaculia_bacterium]	ptg001266l	32.231	2.49E-08	55.8	72
KAB2965344.1_MAG:_vitamin_B12-dependent_ribonucleotide_reductase_[Thermoanaerobaculia_bacterium]	ptg002723l	23.619	9.63E-29	129	55
KAB2965343.1_MAG:_methylmalonyl-CoA_mutase,_partial_[Thermoanaerobaculia_bacterium]	LG30	61.667	7.85E-13	75.1	31
KAB2965324.1_MAG:_citrate_synthase_[Thermoanaerobaculia_bacterium]	ptg005518l	59.434	9.56E-168	525	99
KAB2965322.1_MAG:_radical_SAM_protein_[Thermoanaerobaculia_bacterium]	ptg003839l	29.63	2.59E-15	78.2	71
KAB2965321.1_MAG:_NUDIX_hydrolase_[Thermoanaerobaculia_bacterium]	ptg003336l	28.571	0.00000432	50.1	56

KAB2965320.1_MAG:_endonuclease_III_[Thermoanaerobaculia_bacterium]	ptg005275l	43.956	4.32E-31	123	83
KAB2965319.1_MAG:_hypothetical_protein_F9K16_02315,_partial_[Thermoanaerobaculia_bacterium]	ptg004538l	32.653	0.000000287	54.7	38
KAB2965318.1_MAG:_ferrous_iron_transporter_B_[Thermoanaerobaculia_bacterium]	ptg003831l	31.959	2.97E-62	229	85
KAB2965316.1_MAG:_transcriptional_repressor_[Thermoanaerobaculia_bacterium]	ptg005922l	35.088	1.89E-11	64.3	73
KAB2965312.1_MAG:_ABC_transporter_ATP-binding_protein_[Thermoanaerobaculia_bacterium]	ptg001659l	50.698	4.51E-61	209	94
KAB2965309.1_MAG:_6-carboxytetrahydropterin_synthase_QueD_[Thermoanaerobaculia_bacterium]	ptg005533l	34.783	4.16E-12	65.1	95
KAB2965308.1_MAG:_NAD-dependent_epimerase/dehydratase_family_protein_[Thermoanaerobaculia_bacterium]	ptg005035l	28.75	9.32E-22	100	92
KAB2965307.1_MAG:_aconitate_hydratase_AcnA_[Thermoanaerobaculia_bacterium]	ptg002123l	55.338	0	421	96
KAB2965305.1_MAG:_YqgE/AlgH_family_protein_[Thermoanaerobaculia_bacterium]	ptg005204l	34.302	6.31E-18	84.7	86
KAB2965304.1_MAG:_pyridoxamine_5'-phosphate_oxidase_[Thermoanaerobaculia_bacterium]	ptg001693l	43.005	2.3E-40	149	98
KAB2965302.1_MAG:_SDR_family_NAD(P)-dependent_oxidoreductase_[Thermoanaerobaculia_bacterium]	ptg003131l	38.462	6.55E-34	132	86
KAB2965298.1_MAG:_sugar_transferase_[Thermoanaerobaculia_bacterium]	ptg004085l	42.739	1.06E-53	198	47
KAB2965326.1_MAG:_DNA_polymerase_III_subunit_alpha_[Thermoanaerobaculia_bacterium]	ptg003999l	37.574	0	769	99
KAB2965325.1_MAG:_CDP-alcohol_phosphatidyltransferase_family_protein_[Thermoanaerobaculia_bacterium]	ptg005527l	31.25	1.97E-09	59.7	93
KAB2965270.1_MAG:_cysteine_synthase_family_protein_[Thermoanaerobaculia_bacterium]	ptg001170l	41.311	8.72E-52	186	99
KAB2965269.1_MAG:_redoxin_domain-containing_protein_[Thermoanaerobaculia_bacterium]	ptg002992l	30.216	4.55E-08	55.5	77
KAB2965267.1_MAG:_alpha/beta_hydrolase_[Thermoanaerobaculia_bacterium]	ptg005092l	25.862	5.36E-09	60.5	80
KAB2965266.1_MAG:_IS110_family_transposase_[Thermoanaerobaculia_bacterium]	ptg003872l	34.911	7.04E-53	191	100
KAB2965265.1_MAG:_IS110_family_transposase_[Thermoanaerobaculia_bacterium]	ptg003872l	35.207	8.56E-54	193	100
KAB2965262.1_MAG:_DinB_family_protein_[Thermoanaerobaculia_bacterium]	ptg002031l	30.719	1.27E-14	75.9	70
KAB2965258.1_MAG:_tryptophan-rich_sensory_protein_[Thermoanaerobaculia_bacterium]	ptg004746l	51.22	1.48E-23	99.8	75
KAB2965249.1_MAG:_hypothetical_protein_F9K16_02400_[Thermoanaerobaculia_bacterium]	ptg000619l	41.379	3.78E-14	73.6	51
KAB2965273.1_MAG:_GGDEF_domain-containing_protein,_partial_[Thermoanaerobaculia_bacterium]	LG04	38.798	6.55E-27	119	36
KAB2965272.1_MAG:_tetratricopeptide_repeat_protein_[Thermoanaerobaculia_bacterium]	LG25	31.017	3.39E-25	117	41
KAB2965271.1_MAG:_monofunctional_biosynthetic_peptidoglycan_transglycosylase_[Thermoanaerobaculia_bacterium]	ptg005647l	41.573	9.37E-29	117	70
KAB2965235.1_MAG:_polysaccharide_biosynthesis_tyrosine_autokinase_[Thermoanaerobaculia_bacterium]	ptg003058l	29.779	1.78E-24	114	35
KAB2965216.1_MAG:_NAD-dependent_epimerase/dehydratase_family_protein_[Thermoanaerobaculia_bacterium]	ptg003768l	32.288	4.07E-36	141	99
KAB2965215.1_MAG:_hypothetical_protein_F9K16_02565_[Thermoanaerobaculia_bacterium]	ptg004372l	27.143	5.64E-14	74.3	60
KAB2965211.1_MAG:_PP2C_family_protein-serine/threonine_phosphatase_[Thermoanaerobaculia_bacterium]	ptg004476l	30.932	1.15E-13	77	56
KAB2965210.1_MAG:_D-alanyl-D-alanine_carboxypeptidase/D-alanyl-D-alanine-endopeptidase_[Thermoanaerobaculia_bacterium]	ptg005301l	27.7	1.59E-29	127	78
KAB2965209.1_MAG:_methyltransferase_domain-containing_protein_[Thermoanaerobaculia_bacterium]	ptg005641l	28.922	1.66E-13	73.6	78
KAB2965207.1_MAG:_NupC/NupG_family_nucleoside_CNT_transporter_[Thermoanaerobaculia_bacterium]	ptg001925l	48.312	7.21E-75	257	95

KAB2965205.1_MAG:_magnesium/cobalt_transporter_CorA_[Thermoanaerobaculia_bacterium]	ptg003762l	26.284	9.45E-26	111	98
KAB2965204.1_MAG:_protein_kinase_[Thermoanaerobaculia_bacterium]	ptg001897l	37.879	2.12E-45	182	30
KAB2965203.1_MAG:_hypothetical_protein_F9K16_02505_[Thermoanaerobaculia_bacterium]	ptg002175l	28.44	0.00000763	50.4	44
KAB2965196.1_MAG:_YceI_family_protein_[Thermoanaerobaculia_bacterium]	ptg002928l	29.605	3.85E-13	70.9	75
KAB2965193.1_MAG:_cysteine_synthase_family_protein_[Thermoanaerobaculia_bacterium]	ptg003871l	42.903	3.29E-43	162	95
KAB2965189.1_MAG:_DEAD/DEAH_box_helicase_[Thermoanaerobaculia_bacterium]	ptg005671l	39.41	2.85E-80	272	88
KAB2965188.1_MAG:_DNA_polymerase_IV_[Thermoanaerobaculia_bacterium]	ptg004053l	43.466	1.33E-65	229	96
KAB2965167.1_MAG:_ParA_family_protein_[Thermoanaerobaculia_bacterium]	ptg004457l	31.939	2.58E-26	111	97
KAB2965150.1_MAG:_EAL_domain-containing_protein_[Thermoanaerobaculia_bacterium]	ptg005727l	31.416	1.02E-27	118	60
KAB2965149.1_MAG:_phosphoribosylaminoimidazolesuccinocarboxamide_synthase_[Thermoanaerobaculia_bacterium]	ptg001255l	52.542	2.99E-32	129	39
KAB2965147.1_MAG:_glycosyltransferase_family_2_protein_[Thermoanaerobaculia_bacterium]	ptg001909l	47.451	8.33E-55	196	76
KAB2965137.1_MAG:_hypothetical_protein_F9K18_06875,_partial_[Thermoanaerobaculia_bacterium]	ptg001925l	23.077	8.63E-11	67.4	91
KAB2965134.1_MAG:_ribonuclease_Y_[Thermoanaerobaculia_bacterium]	ptg005484l	43.371	6.4E-95	320	85
KAB2965133.1_MAG:_TIGR00282_family_metallophosphoesterase_[Thermoanaerobaculia_bacterium]	ptg005484l	42.802	2.67E-57	200	98
KAB2965132.1_MAG:_glycosyltransferase_family_2_protein_[Thermoanaerobaculia_bacterium]	ptg003625l	43.443	2.7E-51	184	83
KAB2965130.1_MAG:_hypothetical_protein_F9K16_02695_[Thermoanaerobaculia_bacterium]	ptg001170l	50	3.17E-22	97.4	38
KAB2965129.1_MAG:_HlyD_family_efflux_transporter_periplasmic_adaptor_subunit_[Thermoanaerobaculia_bacterium]	ptg003290l	33.624	1.26E-13	75.9	69
KAB2965128.1_MAG:_ABC_transporter_ATP-binding_protein_[Thermoanaerobaculia_bacterium]	ptg003466l	34.361	7.83E-44	164	70
KAB2965127.1_MAG:_ABC_transporter_ATP-binding_protein_[Thermoanaerobaculia_bacterium]	ptg004579l	44.493	5.47E-51	185	70
KAB2965125.1_MAG:_sulfate_adenylyltransferase_subunit_CysN_[Thermoanaerobaculia_bacterium]	ptg001062l	52.322	0	694	100
KAB2965124.1_MAG:_sulfate_adenylyltransferase_subunit_CysD_[Thermoanaerobaculia_bacterium]	ptg002453l	65.902	8.3E-137	431	100
KAB2965123.1_MAG:_adenylyl-sulfate_kinase_[Thermoanaerobaculia_bacterium]	ptg003971l	53.403	2.17E-47	170	79
KAB2965120.1_MAG:_hypothetical_protein_F9K16_02715_[Thermoanaerobaculia_bacterium]	ptg000619l	38.028	1.92E-08	62	11
KAB2965119.1_MAG:_nucleotide_pyrophosphatase_[Thermoanaerobaculia_bacterium]	ptg000619l	37.5	0.000000179	59.3	9
KAB2965118.1_MAG:_sulfotransferase_[Thermoanaerobaculia_bacterium]	ptg000619l	41.711	2.25E-40	149	88
KAB2965116.1_MAG:_sulfatase-like_hydrolase/transferase,_partial_[Thermoanaerobaculia_bacterium]	ptg003382l	25.424	1.28E-13	79	49
KAB2965115.1_MAG:_2-oxoacid:acceptor_oxidoreductase_subunit_alpha_[Thermoanaerobaculia_bacterium]	ptg002867l	56.385	0	713	96
KAB2965114.1_MAG:_2-oxoacid:ferredoxin_oxidoreductase_subunit_beta_[Thermoanaerobaculia_bacterium]	ptg002102l	55.357	6.07E-123	393	98
KAB2965113.1_MAG:_hypothetical_protein_F9K18_06880,_partial_[Thermoanaerobaculia_bacterium]	ptg004520l	37.202	2.97E-112	378	87
KAB2965098.1_MAG:_penicillin_acylase_family_protein_[Thermoanaerobaculia_bacterium]	ptg004534l	32.039	7.37E-32	138	55
KAB2965096.1_MAG:_DEAD/DEAH_box_helicase_[Thermoanaerobaculia_bacterium]	ptg003863l	40	8.26E-82	277	94
KAB2965095.1_MAG:_DNA_polymerase_IV_[Thermoanaerobaculia_bacterium]	ptg004053l	43.343	3.19E-66	230	98
KAB2965093.1_MAG:_Rne/Rng_family_ribonuclease_[Thermoanaerobaculia_bacterium]	ptg002760l	40.602	1.68E-107	360	80

KAB2965090.1_MAG:_pyruvate_dehydrogenase_(acetyl-transferring),_homodimeric_type_[Thermoanaerobaculia_bacterium]	ptg005844l	61.373	0	619	56
KAB2965089.1_MAG:_SpoIIE_family_protein_phosphatase_[Thermoanaerobaculia_bacterium]	ptg004476l	27.308	1.23E-09	66.2	29
KAB2965087.1_MAG:_peptide_chain_release_factor_3_[Thermoanaerobaculia_bacterium]	ptg005772l	52.182	1.04E-173	547	99
KAB2965102.1_MAG:_PQQ-dependent_sugar_dehydrogenase_[Thermoanaerobaculia_bacterium]	ptg006020l	35.696	1.4E-40	159	78
KAB2965101.1_MAG:_glycogen_synthase_[Thermoanaerobaculia_bacterium]	ptg003785l	39.394	2.97E-101	337	99
KAB2965100.1_MAG:_glucose-1-phosphate_adenylyltransferase_[Thermoanaerobaculia_bacterium]	ptg002878l	47.897	8.1E-108	353	99
KAB2965085.1_MAG:_response_regulator_transcription_factor,_partial_[Thermoanaerobaculia_bacterium]	ptg005150l	43.077	7.02E-09	55.1	63
KAB2965071.1_MAG:_GGDEF_domain-containing_protein_[Thermoanaerobaculia_bacterium]	ptg003282l	41.935	3.59E-20	97.4	29
KAB2965065.1_MAG:_hypothetical_protein_F9K16_02890_[Thermoanaerobaculia_bacterium]	ptg003112l	29.193	1.99E-26	116	72
KAB2965064.1_MAG:_sigma-70_family_RNA_polymerase_sigma_factor_[Thermoanaerobaculia_bacterium]	ptg004340l	40.415	1.43E-20	93.2	89
KAB2965061.1_MAG:_serine/threonine_protein_kinase_[Thermoanaerobaculia_bacterium]	ptg004340l	37.086	1.83E-38	161	25
KAB2965058.1_MAG:_MOSC_domain-containing_protein_[Thermoanaerobaculia_bacterium]	ptg002052l	36.538	3.44E-33	130	81
KAB2965076.1_MAG:_tetratricopeptide_repeat_protein,_partial_[Thermoanaerobaculia_bacterium]	ptg002123l	30.556	0.00000541	47.8	82
KAB2965075.1_MAG:_TonB-dependent_receptor_[Thermoanaerobaculia_bacterium]	ptg001925l	30.303	0.00000116	57	10
KAB2965074.1_MAG:_ribonuclease_D_[Thermoanaerobaculia_bacterium]	ptg005121l	38.994	3.4E-25	111	42
KAB2965053.1_MAG:_sigma-54-dependent_Fis_family_transcriptional_regulator_[Thermoanaerobaculia_bacterium]	ptg005150l	38.507	3.88E-55	197	96
KAB2965032.1_MAG:_hypothetical_protein_F9K16_03000_[Thermoanaerobaculia_bacterium]	ptg005354l	36.752	2.69E-29	127	43
KAB2965021.1_MAG:_type_III-B_CRISPR-associated_protein_Cas10/Cmr2_[Thermoanaerobaculia_bacterium]	ptg001353l	27	1.58E-08	42.7	28
KAB2965017.1_MAG:_adenylyl-sulfate_kinase_[Thermoanaerobaculia_bacterium]	ptg003971l	56.442	5.01E-40	149	66
KAB2965016.1_MAG:_sulfate_adenylyltransferase_subunit_CysD_[Thermoanaerobaculia_bacterium]	ptg002453l	64.026	3.24E-132	417	100
KAB2965015.1_MAG:_sulfate_adenylyltransferase_subunit_CysN_[Thermoanaerobaculia_bacterium]	ptg001062l	51.777	0	668	100
KAB2965014.1_MAG:_GDP-L-fucose_synthase_[Thermoanaerobaculia_bacterium]	ptg006001l	53.398	3.83E-104	337	98
KAB2965012.1_MAG:_LysM_peptidoglycan-binding_domain-containing_protein_[Thermoanaerobaculia_bacterium]	ptg005398l	23.78	4.93E-21	100	54
KAB2965010.1_MAG:_DUF3857_domain-containing_protein,_partial_[Thermoanaerobaculia_bacterium]	ptg001907l	34.483	1.18E-15	86.3	15
KAB2964997.1_MAG:_proline--tRNA_ligase_[Thermoanaerobaculia_bacterium]	ptg003673l	38.796	2.21E-67	174	91
KAB2964995.1_MAG:_phenylalanine--tRNA_ligase_subunit_alpha_[Thermoanaerobaculia_bacterium]	ptg001925l	49.038	3.72E-94	310	88
KAB2964994.1_MAG:_50S_ribosomal_protein_L20_[Thermoanaerobaculia_bacterium]	ptg001925l	54.237	4.12E-33	125	92
KAB2964992.1_MAG:_translation_initiation_factor_IF-3_[Thermoanaerobaculia_bacterium]	ptg003290l	49.08	3.2E-41	150	89
KAB2964991.1_MAG:_threonine--tRNA_ligase_[Thermoanaerobaculia_bacterium]	ptg004758l	46.016	8.24E-170	541	94
KAB2964990.1_MAG:_class_I_SAM-dependent_rRNA_methyltransferase_[Thermoanaerobaculia_bacterium]	ptg002871l	33.333	3.42E-49	182	94
KAB2964989.1_MAG:_TonB-dependent_receptor_[Thermoanaerobaculia_bacterium]	ptg001925l	27.707	0.000000211	59.7	28
KAB2964987.1_MAG:_HlyC/CorC_family_transporter_[Thermoanaerobaculia_bacterium]	ptg004136l	42.553	1.91E-79	272	90

KAB2964984.1_MAG:_3-hydroxybutyryl-CoA_dehydrogenase_[Thermoanaerobaculia_bacterium]	ptg002612l	49.281	1.58E-77	259	97
KAB2964981.1_MAG:_Fe-S_cluster_assembly_ATPase_SufC_[Thermoanaerobaculia_bacterium]	ptg005265l	65.863	2.31E-108	347	98
KAB2964980.1_MAG:_Fe-S_cluster_assembly_protein_SufB_[Thermoanaerobaculia_bacterium]	ptg005265l	74.737	0	712	98
KAB2964978.1_MAG:_PAS_domain_S-box_protein_[Thermoanaerobaculia_bacterium]	ptg002738l	29.517	3.79E-34	144	54
KAB2964976.1_MAG:_GTP_cyclohydrolase_I_FolE_[Thermoanaerobaculia_bacterium]	ptg002479l	49.451	3.03E-48	172	87
KAB2964974.1_MAG:_3-oxoacyl-ACP_synthase_[Thermoanaerobaculia_bacterium]	ptg003871l	35.795	7.71E-45	168	98
KAB2964950.1_MAG:_type_I_restriction_endonuclease_subunit_R_[Thermoanaerobaculia_bacterium]	ptg000693l	42.667	1.45E-08	57	41
KAB2964949.1_MAG:_SAM-dependent_DNA_methyltransferase_[Thermoanaerobaculia_bacterium]	ptg000693l	30.303	2.52E-54	201	91
KAB2964948.1_MAG:_hypothetical_protein_F9K16_03180_[Thermoanaerobaculia_bacterium]	ptg003098l	31.69	1.04E-15	75.1	42
KAB2964947.1_MAG:_patatin-like_phospholipase_family_protein_[Thermoanaerobaculia_bacterium]	ptg005113l	49.394	1.63E-79	268	96
KAB2964946.1_MAG:_nucleotidyltransferase_[Thermoanaerobaculia_bacterium]	ptg005113l	32.791	2.44E-54	201	69
KAB2964945.1_MAG:_hypothetical_protein_F9K16_03160_[Thermoanaerobaculia_bacterium]	ptg004186l	28.019	1.43E-09	64.7	39
KAB2964943.1_MAG:_type_I_restriction_endonuclease_subunit_R_[Thermoanaerobaculia_bacterium]	ptg000693l	26.316	8.1E-68	254	85
KAB2964942.1_MAG:_cell_filamentation_protein_Fic_[Thermoanaerobaculia_bacterium]	ptg004810l	32.292	1.85E-08	57.8	40
KAB2964938.1_MAG:_transcriptional_regulator_[Thermoanaerobaculia_bacterium]	ptg005254l	44.767	2.75E-44	160	85
KAB2964937.1_MAG:_nucleotidyl_transferase_AbiEii/AbiGii_toxin_family_protein_[Thermoanaerobaculia_bacterium]	ptg005254l	37.778	1.19E-55	197	90
KAB2964936.1_MAG:_DUF3387_domain-containing_protein_[Thermoanaerobaculia_bacterium]	ptg001691l	46.667	1.23E-15	76.3	50
KAB2964956.1_MAG:_molybdopterin-dependent_oxidoreductase,_partial_[Thermoanaerobaculia_bacterium]	ptg005451l	26.853	1.29E-23	110	89
KAB2964955.1_MAG:_4Fe-4S_dicluster_domain-containing_protein_[Thermoanaerobaculia_bacterium]	ptg005913l	48.78	1.99E-36	118	95
KAB2964932.1_MAG:_tRNA_guanosine(34)_transglycosylase_Tgt_[Thermoanaerobaculia_bacterium]	ptg002867l	43.784	1.69E-86	301	55
KAB2964929.1_MAG:_protein_translocase_subunit_SecD_[Thermoanaerobaculia_bacterium]	ptg005746l	37.702	3.02E-73	257	91
KAB2964928.1_MAG:_preprotein_translocase_subunit_YajC_[Thermoanaerobaculia_bacterium]	ptg005746l	43.617	2.46E-10	59.3	87
KAB2964915.1_MAG:_molybdenum_cofactor_biosynthesis_protein_MoaE_[Thermoanaerobaculia_bacterium]	ptg005933l	37.671	2.28E-08	55.5	95
KAB2964913.1_MAG:_hypothetical_protein_F9K16_03245_[Thermoanaerobaculia_bacterium]	ptg001245l	33.206	2.01E-23	104	81
KAB2964912.1_MAG:_DUF1446_domain-containing_protein_[Thermoanaerobaculia_bacterium]	ptg004500l	55.629	5.06E-151	478	99
KAB2964911.1_MAG:_PAS_domain_S-box_protein_[Thermoanaerobaculia_bacterium]	ptg002738l	37.121	6.43E-38	155	41
KAB2964908.1_MAG:_DNA-processing_protein_DprA_[Thermoanaerobaculia_bacterium]	ptg003098l	36.458	1.53E-22	100	75
KAB2964907.1_MAG:_class_I_SAM-dependent_DNA_methyltransferase_[Thermoanaerobaculia_bacterium]	ptg004254l	22.184	2.91E-40	167	72
KAB2964906.1_MAG:_acyl-CoA_carboxylase_subunit_beta_[Thermoanaerobaculia_bacterium]	ptg002612l	50.391	6.46E-151	484	86
KAB2964917.1_MAG:_transketolase,_partial_[Thermoanaerobaculia_bacterium]	ptg005204l	53.16	2E-51	184	96
KAB2964916.1_MAG:_response_regulator_[Thermoanaerobaculia_bacterium]	ptg003903l	38.912	1.46E-28	129	17

KAB2964884.1_MAG:_twin-arginine_translocation_signal_domain-containing_protein,_partial_[Thermoanaerobaculia_bacterium]	ptg001690l	35.374	2.37E-25	112	71
KAB2964883.1_MAG:_cytochrome_c3_family_protein_[Thermoanaerobaculia_bacterium]	ptg001690l	44.388	2.31E-42	155	81
KAB2964881.1_MAG:_FtsX-like_permease_family_protein_[Thermoanaerobaculia_bacterium]	ptg004986l	27.489	1.63E-25	113	100
KAB2964880.1_MAG:_ABC_transporter_permease_[Thermoanaerobaculia_bacterium]	ptg004986l	28.442	1.24E-27	119	97
KAB2964879.1_MAG:_ABC_transporter_ATP-binding_protein_[Thermoanaerobaculia_bacterium]	ptg004986l	57.339	5.63E-77	256	88
KAB2964878.1_MAG:_efflux_RND_transporter_periplasmic_adaptor_subunit_[Thermoanaerobaculia_bacterium]	ptg003498l	22.857	0.00000297	53.5	55
KAB2964873.1_MAG:_CHAT_domain-containing_protein_[Thermoanaerobaculia_bacterium]	LG07	30.526	2.2E-14	82.8	26
KAB2964871.1_MAG:_hypothetical_protein_F9K16_03255,_partial_[Thermoanaerobaculia_bacterium]	ptg003813l	24.782	1.22E-54	211	85
KAB2964870.1_MAG:_DNA_polymerase/3'-5'_exonuclease_PolX_[Thermoanaerobaculia_bacterium]	ptg005756l	39.93	7.37E-124	406	97
KAB2964867.1_MAG:_methionine_adenosyltransferase_[Thermoanaerobaculia_bacterium]	ptg001658l	62.432	5.19E-136	432	94
KAB2964865.1_MAG:_tetratricopeptide_repeat_protein_[Thermoanaerobaculia_bacterium]	ptg005121l	31.452	0.00000054	57.4	18
KAB2964864.1_MAG:_glycosyltransferase_[Thermoanaerobaculia_bacterium]	ptg004384l	29.245	0.000000025	60.5	44
KAB2964863.1_MAG:_glycosyltransferase_family_4_protein_[Thermoanaerobaculia_bacterium]	ptg002534l	24.28	1.97E-09	63.5	60
KAB2964844.1_MAG:_glutamine_synthetase_[Thermoanaerobaculia_bacterium]	ptg004075l	29.897	5.53E-28	121	76
KAB2964842.1_MAG:_M23_family_metallopeptidase_[Thermoanaerobaculia_bacterium]	ptg002270l	45.299	1.92E-19	91.7	43
KAB2964840.1_MAG:_dehypoxanthine_futalosine_cyclase_[Thermoanaerobaculia_bacterium]	ptg002867l	45.96	4.24E-109	356	95
KAB2964848.1_MAG:_ABC_transporter_ATP-binding_protein,_partial_[Thermoanaerobaculia_bacterium]	ptg004026l	59.565	1.9E-87	287	83
KAB2964846.1_MAG:_elongation_factor_4_[Thermoanaerobaculia_bacterium]	ptg004662l	52.862	0	604	99
KAB2964845.1_MAG:_hypothetical_protein_F9K18_07220,_partial_[Thermoanaerobaculia_bacterium]	ptg004361l	33.621	5.89E-08	59.3	27
KAB2964816.1_MAG:_acyl-CoA_thioesterase_[Thermoanaerobaculia_bacterium]	ptg003536l	37.079	4.97E-12	64.7	75
KAB2964815.1_MAG:_gamma_carbonic_anhydrase_family_protein_[Thermoanaerobaculia_bacterium]	ptg004532l	43.353	4.35E-39	144	95
KAB2964814.1_MAG:_histidine--tRNA_ligase_[Thermoanaerobaculia_bacterium]	ptg003703l	51.227	3.75E-86	290	75
KAB2964813.1_MAG:_aspartate--tRNA_ligase_[Thermoanaerobaculia_bacterium]	ptg003078l	46.194	1.29E-128	419	98
KAB2964806.1_MAG:_DinB_family_protein_[Thermoanaerobaculia_bacterium]	ptg004158l	36.31	1.17E-29	117	96
KAB2964801.1_MAG:_sulfatase-like_hydrolase/transferase_[Thermoanaerobaculia_bacterium]	ptg003090l	24.147	1.01E-08	62.8	48
KAB2964789.1_MAG:_hypothetical_protein_F9K18_07275_[Thermoanaerobaculia_bacterium]	ptg000619l	50.427	1.48E-33	129	65
KAB2964788.1_MAG:_4-hydroxy-3-methylbut-2-enyl_diphosphate_reductase_[Thermoanaerobaculia_bacterium]	ptg005010l	34.286	1.74E-38	147	93
KAB2964787.1_MAG:_hypothetical_protein_F9K18_07285_[Thermoanaerobaculia_bacterium]	ptg004584l	52.174	0.000000146	51.2	46
KAB2964786.1_MAG:_phosphoribosylamine--glycine_ligase_[Thermoanaerobaculia_bacterium]	ptg002175l	44.575	6.33E-88	296	92
KAB2964783.1_MAG:_tRNA_(N6-threonylcarbamoyladenosine(37)-N6)-methyltransferase_TrmO,_partial_[Thermoanaerobaculia_bacterium]	ptg005550l	38.806	4.11E-12	66.2	84
KAB2964781.1_MAG:_methyltransferase_domain-containing_protein_[Thermoanaerobaculia_bacterium]	ptg004105l	30.909	5.46E-13	71.2	73
KAB2964780.1_MAG:_TonB-dependent_receptor_[Thermoanaerobaculia_bacterium]	ptg005711l	43.689	9.47E-164	528	93
KAB2964774.1_MAG:_hypothetical_protein_F9K16_03505_[Thermoanaerobaculia_bacterium]	ptg004351l	37.624	7.75E-11	63.9	54

KAB2964772.1_MAG:_(2Fe-2S)-binding_protein_[Thermoanaerobaculia_bacterium]	LG08	58.333	0.00000225	49.7	23
KAB2964770.1_MAG:_ABC_transporter_ATP-binding_protein_[Thermoanaerobaculia_bacterium]	ptg004534l	48.472	3.51E-67	228	89
KAB2964767.1_MAG:_sigma-54-dependent_Fis_family_transcriptional_regulator_[Thermoanaerobaculia_bacterium]	ptg001025l	39.66	5.99E-59	213	77
KAB2964753.1_MAG:_biosynthetic_arginine_decarboxylase_[Thermoanaerobaculia_bacterium]	ptg005430l	29.952	4.64E-08	60.5	31
KAB2964736.1_MAG:_sigma-70_family_RNA_polymerase_sigma_factor_[Thermoanaerobaculia_bacterium]	ptg002465l	34.409	0.000000616	52.4	49
KAB2964735.1_MAG:_amidohydrolase_family_protein_[Thermoanaerobaculia_bacterium]	ptg004242l	32.552	6.47E-34	138	92
KAB2964734.1_MAG:_amidohydrolase_family_protein_[Thermoanaerobaculia_bacterium]	ptg004242l	35.369	1.06E-52	194	88
KAB2964733.1_MAG:_menaquinone_biosynthesis_protein_[Thermoanaerobaculia_bacterium]	ptg002867l	39.149	1.4E-32	129	87
KAB2964732.1_MAG:_transcription_termination_factor_Rho_[Thermoanaerobaculia_bacterium]	ptg003762l	66.826	0	587	92
KAB2964731.1_MAG:_YihA_family_ribosome_biogenesis_GTP-binding_protein_[Thermoanaerobaculia_bacterium]	ptg004532l	33.333	1.12E-23	104	69
KAB2964730.1_MAG:_endopeptidase_La_[Thermoanaerobaculia_bacterium]	ptg005527l	51.323	0	712	94
KAB2964729.1_MAG:_ATP-dependent_Clp_protease_ATP-binding_subunit_ClpX_[Thermoanaerobaculia_bacterium]	ptg004240l	61.707	6.13E-157	494	98
KAB2964728.1_MAG:_ATP-dependent_Clp_endopeptidase_proteolytic_subunit_ClpP_[Thermoanaerobaculia_bacterium]	ptg005527l	61.34	6.75E-73	242	99
KAB2964727.1_MAG:_trigger_factor_[Thermoanaerobaculia_bacterium]	ptg005527l	25.874	1.57E-29	125	96
KAB2964725.1_MAG:_enoyl-CoA_hydratase/isomerase_family_protein_[Thermoanaerobaculia_bacterium]	ptg002612l	33.333	6.93E-29	119	94
KAB2964724.1_MAG:_gamma-glutamyl-gamma-aminobutyrate_hydrolase_family_protein_[Thermoanaerobaculia_bacterium]	ptg005867l	34.146	0.000000537	53.9	49
KAB2964723.1_MAG:_enoyl-CoA_hydratase/isomerase_family_protein,_partial_[Thermoanaerobaculia_bacterium]	ptg002612l	35.912	2.57E-18	85.9	87
KAB2964722.1_MAG:_ABC_transporter_ATP-binding_protein_[Thermoanaerobaculia_bacterium]	ptg004724l	36.471	1.74E-42	160	81
KAB2964720.1_MAG:_ABC_transporter_ATP-binding_protein_[Thermoanaerobaculia_bacterium]	ptg001604l	38.762	8.27E-51	185	86
KAB2964703.1_MAG:_osmotically_inducible_protein_OsmC_[Thermoanaerobaculia_bacterium]	ptg004340l	23.881	0.00000751	47.4	98
KAB2964700.1_MAG:_peptide_deformylase_[Thermoanaerobaculia_bacterium]	ptg003098l	46.667	3.11E-39	144	95
KAB2964699.1_MAG:_cysteine--tRNA_ligase_[Thermoanaerobaculia_bacterium]	ptg002844l	39.627	1.18E-94	317	96
KAB2964698.1_MAG:_YraN_family_protein_[Thermoanaerobaculia_bacterium]	ptg002907l	40.476	2.83E-09	56.6	69
KAB2964697.1_MAG:_response_regulator_transcription_factor_[Thermoanaerobaculia_bacterium]	ptg003863l	34.343	7.08E-31	122	92
KAB2964696.1_MAG:_GAF_domain-containing_sensor_histidine_kinase_[Thermoanaerobaculia_bacterium]	ptg003863l	33.929	7.49E-11	68.6	23
KAB2964695.1_MAG:_replicative_DNA_helicase_[Thermoanaerobaculia_bacterium]	ptg005535l	40.98	1.71E-90	304	98
KAB2964694.1_MAG:_alanine_racemase_[Thermoanaerobaculia_bacterium]	ptg004959l	33.151	3.29E-42	162	88
KAB2964693.1_MAG:_ABC_transporter_permease_[Thermoanaerobaculia_bacterium]	ptg003336l	42.661	2.01E-51	183	87
KAB2964692.1_MAG:_ABC_transporter_ATP-binding_protein_[Thermoanaerobaculia_bacterium]	ptg003336l	43.564	2.32E-44	163	75
KAB2964686.1_MAG:_energy-dependent_translational_throttle_protein_EttA_[Thermoanaerobaculia_bacterium]	ptg003536l	61.58	0	608	99
KAB2964684.1_MAG:_TonB-dependent_receptor,_partial_[Thermoanaerobaculia_bacterium]	ptg004670l	30.27	5.41E-14	80.5	24

KAB2964670.1_MAG:_ATP-binding_protein_[Thermoanaerobaculia_bacterium]	ptg003334l	33.333	7.7E-23	101	58
KAB2964669.1_MAG:_trypsin-like_serine_protease_[Thermoanaerobaculia_bacterium]	ptg004186l	44.586	1.39E-56	204	75
KAB2964668.1_MAG:_HD_domain-containing_protein_[Thermoanaerobaculia_bacterium]	ptg005839l	34.225	5.83E-20	95.1	53
KAB2964666.1_MAG:_efflux_RND_transporter_permease_subunit_[Thermoanaerobaculia_bacterium]	ptg004670l	33.004	2E-167	552	98
KAB2964665.1_MAG:_efflux_RND_transporter_periplasmic_adaptor_subunit_[Thermoanaerobaculia_bacterium]	ptg001766l	28.538	1.37E-46	177	87
KAB2964659.1_MAG:_class_I_SAM-dependent_methyltransferase_[Thermoanaerobaculia_bacterium]	ptg002534l	33.913	1.66E-11	66.6	52
KAB2964653.1_MAG:_EAL_domain-containing_protein_[Thermoanaerobaculia_bacterium]	ptg005865l	41.203	5.69E-120	332	91
KAB2964652.1_MAG:_two-component_sensor_histidine_kinase,_partial_[Thermoanaerobaculia_bacterium]	ptg002909l	29.289	2.2E-19	91.7	81
KAB2964634.1_MAG:_cysteine--tRNA_ligase,_partial_[Thermoanaerobaculia_bacterium]	ptg002844l	44.591	2.2E-96	322	80
KAB2964633.1_MAG:_peptide_deformylase_[Thermoanaerobaculia_bacterium]	ptg003785l	42.466	1.93E-27	110	85
KAB2964630.1_MAG:_heavy_metal_translocating_P-type_ATPase_[Thermoanaerobaculia_bacterium]	ptg003880l	29.138	1.14E-35	149	79
KAB2964625.1_MAG:_DNA_repair_protein_RadC_[Thermoanaerobaculia_bacterium]	ptg002199l	36.923	7.75E-18	83.6	71
KAB2964623.1_MAG:_efflux_RND_transporter_periplasmic_adaptor_subunit_[Thermoanaerobaculia_bacterium]	ptg001766l	26.462	1.52E-18	92.4	75
KAB2964622.1_MAG:_efflux_RND_transporter_permease_subunit_[Thermoanaerobaculia_bacterium]	ptg004670l	41.128	0	651	99
KAB2964621.1_MAG:_P-II_family_nitrogen_regulator_[Thermoanaerobaculia_bacterium]	ptg003336l	33.636	4.1E-15	73.2	97
KAB2964615.1_MAG:_HD_domain-containing_protein,_partial_[Thermoanaerobaculia_bacterium]	ptg006032l	34.304	8.31E-44	165	84
KAB2964601.1_MAG:_transcription_termination_factor_NusA_[Thermoanaerobaculia_bacterium]	ptg003903l	36.446	9.96E-60	217	85
KAB2964599.1_MAG:_Do_family_serine_endopeptidase_[Thermoanaerobaculia_bacterium]	ptg004479l	38.444	2.99E-71	251	81
KAB2964598.1_MAG:_sigma-54-dependent_Fis_family_transcriptional_regulator_[Thermoanaerobaculia_bacterium]	ptg005150l	38.901	2.66E-89	301	96
KAB2964595.1_MAG:_acyl-CoA_dehydrogenase_[Thermoanaerobaculia_bacterium]	ptg004179l	31.085	9.6E-33	134	88
KAB2964594.1_MAG:_acyl-CoA_dehydrogenase_[Thermoanaerobaculia_bacterium]	ptg004179l	29.428	1.32E-25	112	95
KAB2964593.1_MAG:_methylmalonyl-CoA_mutase_family_protein_[Thermoanaerobaculia_bacterium]	LG30	56	5.07E-16	86.3	42
KAB2964592.1_MAG:_DUF664_domain-containing_protein_[Thermoanaerobaculia_bacterium]	ptg003871l	21.528	1.16E-08	56.6	87
KAB2964591.1_MAG:_cobalamin_B12-binding_domain-containing_protein_[Thermoanaerobaculia_bacterium]	LG30	45.055	1E-11	64.3	66
KAB2964590.1_MAG:_response_regulator_[Thermoanaerobaculia_bacterium]	ptg003734l	42.478	2.08E-19	90.5	48
KAB2964589.1_MAG:_valine--tRNA_ligase_[Thermoanaerobaculia_bacterium]	ptg001925l	41.52	0	696	97
KAB2964587.1_MAG:_type_III_pantothenate_kinase_[Thermoanaerobaculia_bacterium]	ptg002278l	28.634	2.18E-11	67.8	84
KAB2964583.1_MAG:_zinc_metalloprotease_[Thermoanaerobaculia_bacterium]	ptg001995l	43.299	6.17E-42	158	63
KAB2964582.1_MAG:_EAL_domain-containing_protein,_partial_[Thermoanaerobaculia_bacterium]	ptg005876l	38.33	5.31E-87	298	82
KAB2964562.1_MAG:_5-formyltetrahydrofolate_cyclo-ligase_[Thermoanaerobaculia_bacterium]	ptg003247l	25.604	8.82E-08	55.8	85
KAB2964561.1_MAG:_ferredoxin_family_protein_[Thermoanaerobaculia_bacterium]	ptg003928l	47.619	6.9E-17	76.6	100
KAB2964560.1_MAG:_PAS_domain_S-box_protein_[Thermoanaerobaculia_bacterium]	ptg005137l	29.268	4.88E-08	55.1	69

KAB2964559.1_MAG:_NAD(P)/FAD-dependent_oxidoreductase_[Thermoanaerobaculia_bacterium]	ptg003483l	33.451	3.39E-26	114	71
KAB2964558.1_MAG:_tryptophan--tRNA_ligase_[Thermoanaerobaculia_bacterium]	ptg003327l	48.758	5.5E-95	311	99
KAB2964557.1_MAG:_NAD(P)/FAD-dependent_oxidoreductase_[Thermoanaerobaculia_bacterium]	ptg005341l	29.381	2.46E-30	128	85
KAB2964554.1_MAG:_2-oxoisovalerate_dehydrogenase_[Thermoanaerobaculia_bacterium]	ptg004010l	31.61	6.8E-65	238	93
KAB2964549.1_MAG:_peroxidase_[Thermoanaerobaculia_bacterium]	ptg004676l	62.5	1.45E-24	98.2	95
KAB2964544.1_MAG:_tyrosine--tRNA_ligase_[Thermoanaerobaculia_bacterium]	ptg000819l	46.914	3.61E-103	339	98
KAB2964542.1_MAG:_phosphomethylpyrimidine_synthase_ThiC_[Thermoanaerobaculia_bacterium]	LG03	64.706	0	411	95
KAB2964534.1_MAG:_acetyl-CoA_C-acyltransferase_[Thermoanaerobaculia_bacterium]	ptg002612l	34.824	2.02E-51	191	89
KAB2964533.1_MAG:_hypothetical_protein_F9K18_07460_[Thermoanaerobaculia_bacterium]	ptg003382l	29.184	3.32E-41	164	81
KAB2964532.1_MAG:_hypothetical_protein_F9K18_07455,_partial_[Thermoanaerobaculia_bacterium]	ptg005274l	40.116	1.41E-10	67.8	36
KAB2964528.1_MAG:_type_IV_pilus_twitching_motility_protein_PilT_[Thermoanaerobaculia_bacterium]	ptg004468l	48.433	4.07E-94	311	94
KAB2964527.1_MAG:_type_II_secretion_system_F_family_protein_[Thermoanaerobaculia_bacterium]	ptg005301l	41.191	6.65E-94	312	99
KAB2964526.1_MAG:_PAS_domain-containing_protein_[Thermoanaerobaculia_bacterium]	ptg005886l	29.339	7.53E-18	91.7	42
KAB2964525.1_MAG:_sigma-54-dependent_Fis_family_transcriptional_regulator_[Thermoanaerobaculia_bacterium]	ptg005150l	43.046	2.82E-110	362	98
KAB2964524.1_MAG:_ribosome_small_subunit-dependent_GTPase_A_[Thermoanaerobaculia_bacterium]	ptg004147l	45.249	4.04E-48	179	55
KAB2964523.1_MAG:_hypothetical_protein_F9K16_04125_[Thermoanaerobaculia_bacterium]	ptg005375l	21.159	0.00000105	56.2	47
KAB2964519.1_MAG:_zinc_ribbon_domain-containing_protein_[Thermoanaerobaculia_bacterium]	ptg004942l	42.373	0.000000783	48.9	55
KAB2964504.1_MAG:_valine--tRNA_ligase_[Thermoanaerobaculia_bacterium]	ptg001925l	41.189	0	663	97
KAB2964502.1_MAG:_type_III_pantothenate_kinase_[Thermoanaerobaculia_bacterium]	ptg002278l	28.696	7.71E-14	75.1	84
KAB2964484.1_MAG:_cystathionine_gamma-synthase,_partial_[Thermoanaerobaculia_bacterium]	ptg004895l	38.112	9.87E-33	132	87
KAB2964482.1_MAG:_isocitrate_lyase_family_protein_[Thermoanaerobaculia_bacterium]	LG12	54.667	8.62E-17	89.7	17
KAB2964480.1_MAG:_hypothetical_protein_F9K16_04200_[Thermoanaerobaculia_bacterium]	ptg002660l	27.557	3.68E-12	72.8	86
KAB2964479.1_MAG:_serine/threonine_protein_kinase_[Thermoanaerobaculia_bacterium]	ptg004054l	39.59	3.11E-44	172	52
KAB2964475.1_MAG:_efflux_RND_transporter_permease_subunit_[Thermoanaerobaculia_bacterium]	ptg005906l	35.34	4.96E-43	176	44
KAB2964474.1_MAG:_efflux_RND_transporter_permease_subunit,_partial_[Thermoanaerobaculia_bacterium]	ptg005906l	42.961	8.65E-75	265	74
KAB2964488.1_MAG:_1-acyl-sn-glycerol-3-phosphate_acyltransferase,_partial_[Thermoanaerobaculia_bacterium]	ptg003625l	51.402	3.31E-26	105	86
KAB2964487.1_MAG:_phosphatidate_cytidylyltransferase_[Thermoanaerobaculia_bacterium]	ptg004810l	46.296	1.83E-66	229	84
KAB2964486.1_MAG:_hypothetical_protein_F9K18_07495_[Thermoanaerobaculia_bacterium]	LG07	38.813	1.61E-22	108	75
KAB2964459.1_MAG:_peptide_chain_release_factor_1_[Thermoanaerobaculia_bacterium]	ptg004982l	57.787	2.01E-80	271	67
KAB2964456.1_MAG:_PAS_domain-containing_protein_[Thermoanaerobaculia_bacterium]	ptg002453l	32.051	1.05E-12	76.6	25
KAB2964455.1_MAG:_sigma-54-dependent_Fis_family_transcriptional_regulator_[Thermoanaerobaculia_bacterium]	ptg005150l	42.731	3E-111	364	98
KAB2964454.1_MAG:_50S_ribosomal_protein_L31_[Thermoanaerobaculia_bacterium]	ptg004827l	49.231	6.03E-19	81.6	96

KAB2964452.1_MAG:_Lrp/AsnC_family_transcriptional_regulator_[Thermoanaerobaculia_bacterium]	ptg003131l	32.847	2.38E-14	73.2	83
KAB2964451.1_MAG:_peptide_chain_release_factor_N(5)-glutamine_methyltransferase_[Thermoanaerobaculia_bacterium]	ptg003336l	34.948	2.98E-34	135	99
KAB2964450.1_MAG:_UDP-N-acetylglucosamine_1-carboxyvinyltransferase_[Thermoanaerobaculia_bacterium]	ptg005611l	52.913	1.03E-94	204	96
KAB2964449.1_MAG:_histidine_triad_nucleotide-binding_protein_[Thermoanaerobaculia_bacterium]	ptg003220l	45.133	2.29E-29	113	100
KAB2964448.1_MAG:_transglycosylase_SLT_domain-containing_protein_[Thermoanaerobaculia_bacterium]	ptg004256l	32.468	1.21E-10	69.3	20
KAB2964447.1_MAG:_methylated-DNA--[protein]-cysteine_S-methyltransferase_[Thermoanaerobaculia_bacterium]	ptg005680l	38.889	6.47E-12	67	49
KAB2964463.1_MAG:_1-acyl-sn-glycerol-3-phosphate_acyltransferase_[Thermoanaerobaculia_bacterium]	ptg002123l	32.044	1.53E-15	80.1	68
KAB2964461.1_MAG:_rhomboid_family_intramembrane_serine_protease_[Thermoanaerobaculia_bacterium]	ptg005092l	43.011	2.44E-31	125	73
KAB2964460.1_MAG:_pyridoxal-phosphate_dependent_enzyme,_partial_[Thermoanaerobaculia_bacterium]	LG31	41.86	1.5E-18	84	93
KAB2964436.1_MAG:_formate_dehydrogenase_accessory_sulfurtransferase_FdhD_[Thermoanaerobaculia_bacterium]	ptg002907l	44.495	4.9E-29	119	75
KAB2964434.1_MAG:_molybdopterin-dependent_oxidoreductase_[Thermoanaerobaculia_bacterium]	ptg004306l	42.063	2.82E-141	458	99
KAB2964431.1_MAG:_HNH_endonuclease_[Thermoanaerobaculia_bacterium]	ptg004981l	55.319	0.000000582	52.4	25
KAB2964430.1_MAG:_ATP-binding_protein_[Thermoanaerobaculia_bacterium]	ptg003045l	48.531	0	1012	100
KAB2964428.1_MAG:_DUF1156_domain-containing_protein_[Thermoanaerobaculia_bacterium]	ptg003045l	46.07	0	827	98
KAB2964426.1_MAG:_DUF262_domain-containing_protein_[Thermoanaerobaculia_bacterium]	ptg003431l	28.908	2.02E-53	200	94
KAB2964425.1_MAG:_DUF3883_domain-containing_protein_[Thermoanaerobaculia_bacterium]	ptg003045l	56.151	0	1177	99
KAB2964418.1_MAG:_AAA_domain-containing_protein_[Thermoanaerobaculia_bacterium]	ptg005527l	28.242	6.61E-18	89.4	91
KAB2964416.1_MAG:_ribose-phosphate_pyrophosphokinase_[Thermoanaerobaculia_bacterium]	ptg005867l	54.313	3.61E-97	319	89
KAB2964415.1_MAG:_aminoacyl-tRNA_hydrolase_[Thermoanaerobaculia_bacterium]	ptg005867l	39.362	3.16E-22	97.1	92
KAB2964414.1_MAG:_tetratricopeptide_repeat_protein,_partial_[Thermoanaerobaculia_bacterium]	ptg004982l	30.579	0.000000189	54.7	55
KAB2964413.1_MAG:_4-(cytidine_5'-diphospho)-2-C-methyl-D-erythritol_kinase_[Thermoanaerobaculia_bacterium]	ptg003971l	31.959	2.18E-17	88.2	71
KAB2964412.1_MAG:_septation_regulator_SpoVG_[Thermoanaerobaculia_bacterium]	ptg003971l	29.851	1.55E-10	60.5	81
KAB2964411.1_MAG:_50S_ribosomal_protein_L25_[Thermoanaerobaculia_bacterium]	ptg004982l	31.551	9.14E-25	105	83
KAB2964410.1_MAG:_30S_ribosomal_protein_S6_[Thermoanaerobaculia_bacterium]	ptg005487l	33.028	1.22E-11	64.7	74
KAB2964409.1_MAG:_30S_ribosomal_protein_S18_[Thermoanaerobaculia_bacterium]	ptg005487l	53.448	8.81E-15	71.2	65
KAB2964408.1_MAG:_50S_ribosomal_protein_L9_[Thermoanaerobaculia_bacterium]	ptg005756l	36.111	5.27E-19	85.9	97
KAB2964406.1_MAG:_PBP1A_family_penicillin-binding_protein_[Thermoanaerobaculia_bacterium]	ptg004827l	35.242	1.09E-92	324	84
KAB2964404.1_MAG:_excinuclease_ABC_subunit_UvrB_[Thermoanaerobaculia_bacterium]	ptg004926l	60.951	0	719	89
KAB2964403.1_MAG:_excinuclease_ABC_subunit_UvrC_[Thermoanaerobaculia_bacterium]	ptg005527l	35.616	1.38E-62	229	89
KAB2964385.1_MAG:_serine--tRNA_ligase_[Thermoanaerobaculia_bacterium]	ptg005746l	48.598	4.75E-99	328	98
KAB2964372.1_MAG:_acyl-CoA_dehydrogenase_[Thermoanaerobaculia_bacterium]	ptg004179l	30.523	3.53E-60	222	91
KAB2964369.1_MAG:_asparagine--tRNA_ligase_[Thermoanaerobaculia_bacterium]	ptg002477l	35.141	1.14E-82	281	97
KAB2964368.1_MAG:_ribonuclease_III_[Thermoanaerobaculia_bacterium]	ptg003583l	35.784	7.16E-23	100	81

KAB2964366.1_MAG:_DNA_mismatch_repair_endonuclease_MutL_[Thermoanaerobaculia_bacterium]	ptg004564l	41.587	9.19E-54	204	44
KAB2964365.1_MAG:_pyruvate,_phosphate_dikinase_[Thermoanaerobaculia_bacterium]	ptg005844l	44.816	0	710	98
KAB2964360.1_MAG:_GAF_domain-containing_protein_[Thermoanaerobaculia_bacterium]	ptg005301l	36.574	1.87E-33	136	55
KAB2964359.1_MAG:_3',5'-cyclic-nucleotide_phosphodiesterase_[Thermoanaerobaculia_bacterium]	ptg003764l	26.415	0.00000839	50.4	61
KAB2964358.1_MAG:_MBL_fold_metallo-hydrolase_[Thermoanaerobaculia_bacterium]	ptg005189l	40.412	7.61E-87	295	94
KAB2964341.1_MAG:_hypothetical_protein_F9K16_04530_[Thermoanaerobaculia_bacterium]	ptg002660l	27.957	2.38E-09	63.5	83
KAB2964338.1_MAG:_VCBS_repeat-containing_protein_[Thermoanaerobaculia_bacterium]	ptg005839l	49.18	0.00000299	55.5	6
KAB2964335.1_MAG:_tetratricopeptide_repeat_protein_[Thermoanaerobaculia_bacterium]	ptg002871l	51.227	1.05E-88	310	42
KAB2964334.1_MAG:_VWA_domain-containing_protein_[Thermoanaerobaculia_bacterium]	ptg003982l	28.767	0.000000126	57.8	53
KAB2964333.1_MAG:_protein-L-isoaspartate(D-aspartate)_O-methyltransferase_[Thermoanaerobaculia_bacterium]	ptg004026l	50	4.66E-56	196	82
KAB2964332.1_MAG:_efflux_RND_transporter_periplasmic_adaptor_subunit_[Thermoanaerobaculia_bacterium]	ptg002381l	42.353	5.2E-54	196	88
KAB2964331.1_MAG:_NAD(P)/FAD-dependent_oxidoreductase_[Thermoanaerobaculia_bacterium]	ptg005341l	27.297	1.38E-36	146	87
KAB2964330.1_MAG:_DNA-3-methyladenine_glycosylase_2_family_protein,_partial_[Thermoanaerobaculia_bacterium]	ptg005680l	36.029	1.14E-41	157	84
KAB2964329.1_MAG:_AAA_domain-containing_protein,_partial_[Thermoanaerobaculia_bacterium]	ptg005150l	43.902	4.14E-66	231	80
KAB2964328.1_MAG:_ATP-binding_protein_[Thermoanaerobaculia_bacterium]	ptg002742l	35.135	7.04E-11	62.4	76
KAB2964326.1_MAG:_SpoIIE_family_protein_phosphatase_[Thermoanaerobaculia_bacterium]	ptg004476l	28.384	6.66E-12	72.4	42
KAB2964324.1_MAG:_efflux_RND_transporter_permease_subunit_[Thermoanaerobaculia_bacterium]	ptg002381l	54.381	0	412	97
KAB2964323.1_MAG:_efflux_transporter_outer_membrane_subunit_[Thermoanaerobaculia_bacterium]	ptg002573l	26.571	4.84E-17	88.2	72
KAB2964322.1_MAG:_PQQ-dependent_sugar_dehydrogenase_[Thermoanaerobaculia_bacterium]	ptg004075l	40.974	9.27E-79	269	82
KAB2964321.1_MAG:_thiolase_family_protein_[Thermoanaerobaculia_bacterium]	ptg005213l	39.557	2.66E-61	133	98
KAB2964313.1_MAG:_acyl-CoA_dehydrogenase_[Thermoanaerobaculia_bacterium]	ptg004179l	27.493	1.16E-23	107	96
KAB2964310.1_MAG:_redox-regulated_ATPase_YchF_[Thermoanaerobaculia_bacterium]	ptg002477l	47.541	7.11E-92	305	99
KAB2964301.1_MAG:_ABC_transporter_ATP-binding_protein_[Thermoanaerobaculia_bacterium]	ptg001659l	57.971	6.01E-72	240	95
KAB2964300.1_MAG:_leucyl_aminopeptidase,_partial_[Thermoanaerobaculia_bacterium]	ptg001925l	46.893	1.34E-59	166	93
KAB2964299.1_MAG:_ABC_transporter_ATP-binding_protein_[Thermoanaerobaculia_bacterium]	ptg005227l	40.19	4.71E-61	214	97
KAB2964298.1_MAG:_ABC_transporter_ATP-binding_protein_[Thermoanaerobaculia_bacterium]	ptg005227l	49.822	1.43E-68	237	85
KAB2964297.1_MAG:_TolC_family_protein_[Thermoanaerobaculia_bacterium]	ptg004500l	27.603	1.53E-26	119	74
KAB2964295.1_MAG:_efflux_RND_transporter_periplasmic_adaptor_subunit_[Thermoanaerobaculia_bacterium]	ptg005867l	26.396	1.37E-08	61.2	66
KAB2964294.1_MAG:_FtsX-like_permease_family_protein_[Thermoanaerobaculia_bacterium]	ptg005920l	30.833	1.92E-19	94.7	79
KAB2964291.1_MAG:_primosomal_protein_N'_[Thermoanaerobaculia_bacterium]	ptg003903l	38.871	8.74E-106	362	86
KAB2964290.1_MAG:_acetyl-CoA_carboxylase_carboxyltransferase_subunit_alpha_[Thermoanaerobaculia_bacterium]	ptg005416l	53.979	8.3E-82	273	91
KAB2964289.1_MAG:_single-stranded-DNA-specific_exonuclease_RecJ,_partial_[Thermoanaerobaculia_bacterium]	ptg002820l	33.539	1.66E-49	189	76

KAB2964280.1_MAG:_dTDP-4-dehydrorhamnose_3,5-epimerase_[Thermoanaerobaculia_bacterium]	ptg003827l	58.286	5.42E-63	213	95
KAB2964279.1_MAG:_hypothetical_protein_F9K18_07670,_partial_[Thermoanaerobaculia_bacterium]	ptg005844l	29.268	0.000000185	54.7	72
KAB2964277.1_MAG:_glucose-1-phosphate_thymidylyltransferase_RfbA_[Thermoanaerobaculia_bacterium]	ptg004111l	59.864	5.51E-118	376	99
KAB2964276.1_MAG:_undecaprenyl-diphosphatase_[Thermoanaerobaculia_bacterium]	ptg003644l	31.373	2.24E-13	73.6	92
KAB2964275.1_MAG:_TerC_family_protein_[Thermoanaerobaculia_bacterium]	LG22	50	1.43E-11	69.3	65
KAB2964274.1_MAG:_dTMP_kinase_[Thermoanaerobaculia_bacterium]	ptg003947l	34.737	9.13E-33	128	88
KAB2964266.1_MAG:_aldehyde_dehydrogenase_family_protein,_partial_[Thermoanaerobaculia_bacterium]	ptg004321l	33.333	0.000000588	54.7	40
KAB2964265.1_MAG:_acyl-CoA_dehydrogenase_[Thermoanaerobaculia_bacterium]	ptg004179l	29.049	1.49E-37	149	92
KAB2964263.1_MAG:_SpoIIE_family_protein_phosphatase_[Thermoanaerobaculia_bacterium]	ptg004476l	25.833	5.47E-09	63.2	45
KAB2964262.1_MAG:_SDR_family_oxidoreductase_[Thermoanaerobaculia_bacterium]	ptg003131l	37.824	1.79E-22	100	71
KAB2964260.1_MAG:_type_II_toxin-antitoxin_system_death-on-curing_family_toxin_[Thermoanaerobaculia_bacterium]	ptg002313l	41.905	1.57E-19	86.7	82
KAB2964258.1_MAG:_hypothetical_protein_F9K16_04700_[Thermoanaerobaculia_bacterium]	ptg004652l	31.29	9.74E-17	90.1	33
KAB2964253.1_MAG:_3-hydroxyacyl-ACP_dehydratase_FabZ_[Thermoanaerobaculia_bacterium]	ptg004895l	48.872	1.44E-35	132	92
KAB2964252.1_MAG:_protein-L-isoaspartate(D-aspartate)_O-methyltransferase_[Thermoanaerobaculia_bacterium]	ptg004026l	45.771	3.62E-45	163	96
KAB2964251.1_MAG:_ABC_transporter_ATP-binding_protein_[Thermoanaerobaculia_bacterium]	ptg001995l	45.238	7.68E-49	174	93
KAB2964250.1_MAG:_ATP-dependent_Clp_protease_ATP-binding_subunit_[Thermoanaerobaculia_bacterium]	ptg003199l	44.707	0	691	96
KAB2964249.1_MAG:_outer_membrane_protein_assembly_factor_BamA_[Thermoanaerobaculia_bacterium]	ptg005656l	25.443	4.51E-32	139	97
KAB2964247.1_MAG:_UDP-3-O-(3-hydroxymyristoyl)glucosamine_N-acyltransferase_[Thermoanaerobaculia_bacterium]	ptg003363l	38.621	1.25E-40	156	81
KAB2964246.1_MAG:_acyl-ACP--UDP-N-acetylglucosamine_O-acyltransferase_[Thermoanaerobaculia_bacterium]	ptg004895l	44.13	1.71E-53	190	90
KAB2964244.1_MAG:_S-methyl-5'-thioadenosine_phosphorylase_[Thermoanaerobaculia_bacterium]	ptg005967l	44.521	1.24E-74	252	95
KAB2964243.1_MAG:_5'/3'-nucleotidase_SurE_[Thermoanaerobaculia_bacterium]	ptg005398l	36.759	1.79E-39	149	92
KAB2964242.1_MAG:_adenine_phosphoribosyltransferase_[Thermoanaerobaculia_bacterium]	ptg004306l	52.941	8.17E-46	163	99
KAB2964240.1_MAG:_thymidine_phosphorylase,_partial_[Thermoanaerobaculia_bacterium]	ptg001353l	47.687	1.05E-59	212	74
KAB2964229.1_MAG:_aconitate_hydratase_[Thermoanaerobaculia_bacterium]	ptg000685l	30.208	1.75E-32	139	67
KAB2964225.1_MAG:_hypothetical_protein_F9K16_04865_[Thermoanaerobaculia_bacterium]	ptg005836l	31.707	0.00000606	53.9	34
KAB2964224.1_MAG:_C4-dicarboxylic_acid_transporter_DauA_[Thermoanaerobaculia_bacterium]	ptg004017l	38.298	0.000004	54.3	24
KAB2964223.1_MAG:_ATP-binding_cassette_domain-containing_protein_[Thermoanaerobaculia_bacterium]	ptg002760l	36.979	8.06E-51	113	97
KAB2964222.1_MAG:_ABC_transporter_permease_[Thermoanaerobaculia_bacterium]	ptg002738l	28.821	4.81E-28	121	98
KAB2964220.1_MAG:_ion_transporter_[Thermoanaerobaculia_bacterium]	ptg002436l	50.661	4.6E-42	157	83
KAB2964219.1_MAG:_DNA_methyltransferase_[Thermoanaerobaculia_bacterium]	LG07	49.153	3.76E-09	61.2	20
KAB2964218.1_MAG:_elongation_factor_4_[Thermoanaerobaculia_bacterium]	ptg004662l	53.367	0	625	99
KAB2964217.1_MAG:_ABC_transporter_ATP-binding_protein_[Thermoanaerobaculia_bacterium]	ptg004026l	52.077	7.89E-94	308	98
KAB2964216.1_MAG:_ABC_transporter_permease,_partial_[Thermoanaerobaculia_bacterium]	ptg004026l	53.061	4.76E-11	58.9	80

KAB2964206.1_MAG:_MBL_fold_metallo-hydrolase_[Thermoanaerobaculia_bacterium]	ptg002475l	39.61	6.41E-99	330	92
KAB2964200.1_MAG:_serine/threonine_protein_kinase,_partial_[Thermoanaerobaculia_bacterium]	ptg006032l	39.033	1.27E-50	183	85
KAB2964192.1_MAG:_J_domain-containing_protein,_partial_[Thermoanaerobaculia_bacterium]	ptg005113l	40.541	3.33E-14	73.2	84
KAB2964191.1_MAG:_ATP-dependent_chaperone_ClpB_[Thermoanaerobaculia_bacterium]	ptg002304l	52.697	0	768	99
KAB2964189.1_MAG:_hypothetical_protein_F9K16_04910_[Thermoanaerobaculia_bacterium]	ptg002313l	34.483	4.74E-16	77.4	93
KAB2964185.1_MAG:_hemolysin_III_family_protein_[Thermoanaerobaculia_bacterium]	ptg004790l	38.372	4.07E-11	65.5	76
KAB2964183.1_MAG:_thioredoxin_family_protein,_partial_[Thermoanaerobaculia_bacterium]	ptg003142l	35.065	0.0000013	55.5	14
KAB2964181.1_MAG:_sulfate_ABC_transporter_permease_subunit_CysW_[Thermoanaerobaculia_bacterium]	ptg003471l	38.235	2.07E-14	77	49
KAB2964180.1_MAG:_sulfate_ABC_transporter_permease_subunit_CysT_[Thermoanaerobaculia_bacterium]	ptg003471l	34.783	0.00000826	50.8	58
KAB2964176.1_MAG:_2Fe-2S_iron-sulfur_cluster_binding_domain-containing_protein_[Thermoanaerobaculia_bacterium]	ptg005451l	37.874	5.98E-46	170	94
KAB2964170.1_MAG:_sensor_domain-containing_diguanylate_cyclase_[Thermoanaerobaculia_bacterium]	LG04	43.125	3.25E-24	110	32
KAB2964169.1_MAG:_chaperonin_GroEL_[Thermoanaerobaculia_bacterium]	ptg002031l	59.546	1.4E-176	556	96
KAB2964168.1_MAG:_co-chaperone_GroES_[Thermoanaerobaculia_bacterium]	ptg004085l	51.087	1.26E-24	99.4	96
KAB2964167.1_MAG:_glycosyltransferase_family_2_protein_[Thermoanaerobaculia_bacterium]	ptg002123l	35.897	1.54E-09	63.2	34
KAB2964154.1_MAG:_dicarboxylate/amino_acid:cation_symporter_[Thermoanaerobaculia_bacterium]	ptg004214l	41.444	1.92E-63	224	89
KAB2964153.1_MAG:_ATP-binding_cassette_domain-containing_protein,_partial_[Thermoanaerobaculia_bacterium]	ptg001571l	45.361	3.3E-19	86.3	68
KAB2964152.1_MAG:_hypothetical_protein_F9K18_07790_[Thermoanaerobaculia_bacterium]	ptg002467l	26.69	2.99E-10	68.6	27
KAB2964140.1_MAG:_UMP_kinase_[Thermoanaerobaculia_bacterium]	ptg002436l	52.119	1.12E-66	226	97
KAB2964138.1_MAG:_ribosome_recycling_factor_[Thermoanaerobaculia_bacterium]	ptg005656l	43.678	1.67E-44	160	95
KAB2964137.1_MAG:_translation_elongation_factor_Ts_[Thermoanaerobaculia_bacterium]	ptg001624l	52.284	2.55E-50	178	90
KAB2964136.1_MAG:_30S_ribosomal_protein_S2_[Thermoanaerobaculia_bacterium]	ptg002436l	49.333	4.27E-66	225	88
KAB2964135.1_MAG:_30S_ribosomal_protein_S9_[Thermoanaerobaculia_bacterium]	ptg005611l	61.682	6.19E-29	113	80
KAB2964134.1_MAG:_50S_ribosomal_protein_L13_[Thermoanaerobaculia_bacterium]	ptg000819l	46.575	5.89E-30	117	98
KAB2964133.1_MAG:_class_I_SAM-dependent_methyltransferase_[Thermoanaerobaculia_bacterium]	ptg003903l	43.534	3.51E-42	156	91
KAB2964132.1_MAG:_bifunctional_methylenetetrahydrofolate_dehydrogenase/methenyltetrahydrofolate_cyclohydrolase_FolD_[Thermoanaerobaculia_bacterium]	ptg002453l	47.855	2.81E-71	243	94
KAB2964131.1_MAG:_dephospho-CoA_kinase_[Thermoanaerobaculia_bacterium]	ptg004510l	31.892	1.37E-16	80.9	92
KAB2964130.1_MAG:_class_I_SAM-dependent_methyltransferase_[Thermoanaerobaculia_bacterium]	ptg003361l	28.481	0.00000843	52.4	33
KAB2964128.1_MAG:_FHA_domain-containing_protein_[Thermoanaerobaculia_bacterium]	ptg005137l	30.846	2.1E-15	84	35
KAB2964127.1_MAG:_adenylosuccinate_lyase_[Thermoanaerobaculia_bacterium]	ptg002345l	34.136	5E-57	208	95
KAB2964126.1_MAG:_septal_ring_lytic_transglycosylase_RlpA_family_protein_[Thermoanaerobaculia_bacterium]	ptg005398l	58.065	1.08E-28	116	44
KAB2964125.1_MAG:_phosphoglycerate_dehydrogenase,_partial_[Thermoanaerobaculia_bacterium]	ptg003603l	40.664	2.26E-74	262	81
KAB2964123.1_MAG:_23S_rRNA_(guanosine(2251)-2'-O)-methyltransferase_RlmB_[Thermoanaerobaculia_bacterium]	ptg005487l	37.419	1.79E-23	102	64

KAB2964122.1_MAG:_2-C-methyl-D-erythritol_2,4-cyclodiphosphate_synthase_[Thermoanaerobaculia_bacterium]	ptg001658l	49.686	5.86E-29	114	99
KAB2964114.1_MAG:_RHS_repeat-associated_core_domain-containing_protein_[Thermoanaerobaculia_bacterium]	ptg002381l	43.75	0.000000118	56.6	22
KAB2964107.1_MAG:_RHS_repeat-associated_core_domain-containing_protein_[Thermoanaerobaculia_bacterium]	ptg004854l	30.263	1.59E-20	101	38
KAB2964106.1_MAG:_hypothetical_protein_F9K16_05075_[Thermoanaerobaculia_bacterium]	ptg002381l	32.836	9.67E-16	87.4	13
KAB2964101.1_MAG:_aminotransferase_class_V-fold_PLP-dependent_enzyme_[Thermoanaerobaculia_bacterium]	ptg003871l	26.866	1.72E-13	77.4	67
KAB2964098.1_MAG:_SDR_family_oxidoreductase_[Thermoanaerobaculia_bacterium]	LG02	34.579	0.00000089	53.5	40
KAB2964096.1_MAG:_SDR_family_oxidoreductase_[Thermoanaerobaculia_bacterium]	ptg003980l	42.273	6.26E-48	172	89
KAB2964093.1_MAG:_ribosome_biogenesis_GTPase_Der_[Thermoanaerobaculia_bacterium]	ptg003703l	39.261	5.06E-86	298	68
KAB2964092.1_MAG:_MerR_family_transcriptional_regulator_[Thermoanaerobaculia_bacterium]	ptg001925l	41.558	6.06E-12	65.5	53
KAB2964091.1_MAG:_6,7-dimethyl-8-ribityllumazine_synthase_[Thermoanaerobaculia_bacterium]	ptg004564l	40.559	6.86E-29	114	91
KAB2964090.1_MAG:_transcription_antitermination_factor_NusB_[Thermoanaerobaculia_bacterium]	ptg002909l	52.055	2.09E-16	79.3	43
KAB2964088.1_MAG:_leucine--tRNA_ligase_[Thermoanaerobaculia_bacterium]	ptg005844l	35.833	4.6E-166	538	99
KAB2964086.1_MAG:_30S_ribosomal_protein_S20_[Thermoanaerobaculia_bacterium]	ptg002505l	45.714	1.65E-08	53.1	80
KAB2964084.1_MAG:_peptidoglycan-associated_lipoprotein_Pal_[Thermoanaerobaculia_bacterium]	ptg000834l	38.679	7.59E-10	61.2	55
KAB2964083.1_MAG:_Tol-Pal_system_beta_propeller_repeat_protein_TolB_[Thermoanaerobaculia_bacterium]	ptg001604l	25	9.4E-09	62	45
KAB2964072.1_MAG:_adenosine_deaminase,_partial_[Thermoanaerobaculia_bacterium]	ptg005754l	26.471	6.17E-09	57.8	60
KAB2964071.1_MAG:_hypothetical_protein_F9K18_07870_[Thermoanaerobaculia_bacterium]	ptg001925l	28.613	5.54E-77	282	96
KAB2964063.1_MAG:_(d)CMP_kinase_[Thermoanaerobaculia_bacterium]	ptg003078l	39.64	4.9E-28	114	99
KAB2964062.1_MAG:_integration_host_factor_subunit_beta_[Thermoanaerobaculia_bacterium]	ptg003072l	45.745	5.11E-21	89	99
KAB2964061.1_MAG:_uracil-DNA_glycosylase_[Thermoanaerobaculia_bacterium]	ptg003644l	50	3.76E-49	176	68
KAB2964060.1_MAG:_Glu/Leu/Phe/Val_dehydrogenase_[Thermoanaerobaculia_bacterium]	ptg002313l	28.289	3.39E-10	65.9	73
KAB2964059.1_MAG:_tRNA_epoxyqueuosine(34)_reductase_QueG_[Thermoanaerobaculia_bacterium]	ptg005839l	39.185	1.46E-56	202	91
KAB2964058.1_MAG:_30S_ribosomal_protein_S1_[Thermoanaerobaculia_bacterium]	ptg001025l	46.341	3.54E-131	427	89
KAB2964056.1_MAG:_lipid-A-disaccharide_synthase_[Thermoanaerobaculia_bacterium]	ptg002436l	45.509	7.61E-53	136	87
KAB2964055.1_MAG:_ABC_transporter_ATP-binding_protein_[Thermoanaerobaculia_bacterium]	ptg003058l	35.928	2.21E-77	273	76
KAB2964054.1_MAG:_lysophospholipid_acyltransferase_family_protein_[Thermoanaerobaculia_bacterium]	ptg003364l	34.568	2.34E-25	107	70
KAB2964053.1_MAG:_YicC_family_protein_[Thermoanaerobaculia_bacterium]	ptg005107l	42.056	3.7E-16	82.4	36
KAB2964052.1_MAG:_guanylate_kinase_[Thermoanaerobaculia_bacterium]	ptg003971l	36.898	2.55E-30	121	84
KAB2964050.1_MAG:_bifunctional_phosphopantothenoylcysteine_decarboxylase/phosphopantothenate--cysteine_ligase_CoaBC_[Thermoanaerobaculia_bacterium]	ptg005430l	42.965	8.78E-61	216	96
KAB2964048.1_MAG:_hypothetical_protein_F9K18_07895,_partial_[Thermoanaerobaculia_bacterium]	ptg003762l	25.735	1.68E-08	56.6	78
KAB2964047.1_MAG:_inorganic_phosphate_transporter_[Thermoanaerobaculia_bacterium]	ptg004169l	37.725	1.92E-15	81.3	50
KAB2964045.1_MAG:_polyphosphate_kinase_1_[Thermoanaerobaculia_bacterium]	ptg001995l	41.988	2.79E-133	438	95
KAB2964031.1_MAG:_RHS_repeat-associated_core_domain-containing_protein_[Thermoanaerobaculia_bacterium]	ptg006050l	50	5.54E-10	65.9	12
KAB2964028.1_MAG:_arsenate_reductase_ArsC_[Thermoanaerobaculia_bacterium]	ptg001034l	39.45	2.61E-17	80.9	68

KAB2964024.1_MAG:_hypothetical_protein_F9K16_05305,_partial_[Thermoanaerobaculia_bacterium]	ptg005772l	24.497	7.83E-25	118	38
KAB2964023.1_MAG:_response_regulator_transcription_factor_[Thermoanaerobaculia_bacterium]	ptg003863l	33.333	1.29E-31	124	95
KAB2964020.1_MAG:_replicative_DNA_helicase_[Thermoanaerobaculia_bacterium]	ptg005535l	42.291	4.7E-89	302	87
KAB2964007.1_MAG:_protein_kinase_[Thermoanaerobaculia_bacterium]	ptg002573l	46.067	5.69E-19	85.9	58
KAB2964005.1_MAG:_response_regulator_transcription_factor_[Thermoanaerobaculia_bacterium]	ptg003863l	33.505	1.06E-17	84	93
KAB2964002.1_MAG:_ABC_transporter_ATP-binding_protein_[Thermoanaerobaculia_bacterium]	ptg004026l	34.266	1.69E-30	126	80
KAB2963998.1_MAG:_class_I_SAM-dependent_methyltransferase_[Thermoanaerobaculia_bacterium]	ptg003839l	36.842	0.00000167	53.5	25
KAB2964012.1_MAG:_2-oxoacid:acceptor_oxidoreductase_subunit_alpha_[Thermoanaerobaculia_bacterium]	ptg002867l	56.86	0	705	96
KAB2964011.1_MAG:_2-oxoacid:ferredoxin_oxidoreductase_subunit_beta_[Thermoanaerobaculia_bacterium]	ptg002102l	55.03	2.34E-123	394	98
KAB2964010.1_MAG:_glycogen_debranching_enzyme,_partial_[Thermoanaerobaculia_bacterium]	ptg004520l	46.565	4.59E-69	240	64
KAB2963988.1_MAG:_RNA_polymerase_sigma_factor_RpoD_[Thermoanaerobaculia_bacterium]	ptg005113l	39.216	4.18E-90	307	87
KAB2963986.1_MAG:_acyltransferase_[Thermoanaerobaculia_bacterium]	ptg005876l	25.538	2.45E-14	81.3	47
KAB2963985.1_MAG:_tryptophan_synthase_subunit_beta_[Thermoanaerobaculia_bacterium]	ptg004214l	57.612	8.48E-108	351	86
KAB2963983.1_MAG:_response_regulator_transcription_factor_[Thermoanaerobaculia_bacterium]	ptg003863l	49.057	9.33E-10	59.3	36
KAB2963981.1_MAG:_tryptophan_synthase_subunit_alpha_[Thermoanaerobaculia_bacterium]	ptg004981l	40.161	3.85E-43	160	89
KAB2963974.1_MAG:_GntR_family_transcriptional_regulator_[Thermoanaerobaculia_bacterium]	ptg004470l	36.735	3.56E-09	57	71
KAB2963973.1_MAG:_ABC_transporter_ATP-binding_protein_[Thermoanaerobaculia_bacterium]	ptg004986l	31.615	1.11E-36	142	96
KAB2963971.1_MAG:_acyltransferase_family_protein_[Thermoanaerobaculia_bacterium]	ptg004026l	38.028	3.54E-38	157	38
KAB2963968.1_MAG:_inositol_monophosphatase_[Thermoanaerobaculia_bacterium]	ptg005265l	49.741	2.68E-28	117	68
KAB2963967.1_MAG:_AAA_family_ATPase,_partial_[Thermoanaerobaculia_bacterium]	ptg004306l	43.796	1.5E-62	217	94
KAB2963965.1_MAG:_aspartate_carbamoyltransferase_catalytic_subunit_[Thermoanaerobaculia_bacterium]	ptg005190l	51.977	1.83E-72	179	85
KAB2963964.1_MAG:_dihydroorotase_[Thermoanaerobaculia_bacterium]	LG04	40.293	5.39E-57	176	89
KAB2963954.1_MAG:_sigma-70_family_RNA_polymerase_sigma_factor_[Thermoanaerobaculia_bacterium]	ptg005318l	34.32	2.59E-20	92	79
KAB2963951.1_MAG:_NAD-dependent_DNA_ligase_LigA,_partial_[Thermoanaerobaculia_bacterium]	ptg005087l	43.091	3.89E-135	438	95
KAB2963950.1_MAG:_S46_family_peptidase_[Thermoanaerobaculia_bacterium]	ptg003057l	34.91	1.35E-100	343	99
KAB2963946.1_MAG:_serine/threonine_protein_kinase_[Thermoanaerobaculia_bacterium]	ptg002871l	39.514	2.41E-40	166	33
KAB2963945.1_MAG:_sigma-70_family_RNA_polymerase_sigma_factor_[Thermoanaerobaculia_bacterium]	ptg005822l	39.241	1.78E-16	80.5	77
KAB2963944.1_MAG:_Crp/Fnr_family_transcriptional_regulator_[Thermoanaerobaculia_bacterium]	ptg005331l	30.601	1.39E-15	80.1	68
KAB2963932.1_MAG:_NADP_oxidoreductase,_partial_[Thermoanaerobaculia_bacterium]	LG02	53.333	0.000000246	57.4	23
KAB2963931.1_MAG:_aspartate-semialdehyde_dehydrogenase_[Thermoanaerobaculia_bacterium]	ptg002820l	30.851	0.00000134	54.3	45
KAB2963930.1_MAG:_aminotransferase_class_V-fold_PLP-dependent_enzyme_[Thermoanaerobaculia_bacterium]	ptg004895l	36.815	8.47E-36	143	95
KAB2963929.1_MAG:_hypothetical_protein_F9K18_08010_[Thermoanaerobaculia_bacterium]	ptg004711l	35.468	1.04E-19	91.7	80

KAB2963928.1_MAG:_methionine_gamma-lyase,_partial_[Thermoanaerobaculia_bacterium]	ptg004895l	50	8.5E-26	103	98
KAB2963924.1_MAG:_patatin_[Thermoanaerobaculia_bacterium]	ptg005087l	37.766	3.54E-39	153	90
KAB2963922.1_MAG:_molybdopterin-dependent_oxidoreductase_[Thermoanaerobaculia_bacterium]	ptg005556l	26.418	5.63E-32	139	82
KAB2963920.1_MAG:_insulinase_family_protein_[Thermoanaerobaculia_bacterium]	ptg005195l	41.341	4.11E-76	277	61
KAB2963919.1_MAG:_M1_family_peptidase_[Thermoanaerobaculia_bacterium]	ptg005913l	23.256	4.41E-16	87	81
KAB2963914.1_MAG:_molecular_chaperone_DnaK_[Thermoanaerobaculia_bacterium]	ptg005922l	63.018	0	741	95
KAB2963902.1_MAG:_alpha/beta_hydrolase_[Thermoanaerobaculia_bacterium]	ptg004434l	27.083	8.98E-11	66.2	99
KAB2963901.1_MAG:_DNA_recombination_protein_RmuC,_partial_[Thermoanaerobaculia_bacterium]	ptg004538l	28.571	5.02E-34	139	82
KAB2963900.1_MAG:_SDR_family_oxidoreductase_[Thermoanaerobaculia_bacterium]	ptg002313l	32.982	5.12E-25	110	74
KAB2963899.1_MAG:_succinate-semialdehyde_dehydrogenase_(NADP(+))_[Thermoanaerobaculia_bacterium]	ptg004321l	29.956	2.86E-47	181	85
KAB2963897.1_MAG:_aminotransferase_class_I/II-fold_pyridoxal_phosphate-dependent_enzyme_[Thermoanaerobaculia_bacterium]	ptg005614l	40.496	2.19E-66	233	87
KAB2963894.1_MAG:_DUF59_domain-containing_protein_[Thermoanaerobaculia_bacterium]	ptg005265l	38.919	1.37E-30	123	70
KAB2963893.1_MAG:_S9_family_peptidase_[Thermoanaerobaculia_bacterium]	ptg003431l	38.34	5.2E-138	453	94
KAB2963892.1_MAG:_SDR_family_oxidoreductase_[Thermoanaerobaculia_bacterium]	ptg002634l	31.638	2.35E-11	68.9	50
KAB2963891.1_MAG:_MBL_fold_metallo-hydrolase,_partial_[Thermoanaerobaculia_bacterium]	ptg002760l	38.034	4.11E-24	104	88
KAB2963890.1_MAG:_bifunctional_hydroxymethylpyrimidine_kinase/phosphomethylpyrimidine_kinase_[Thermoanaerobaculia_bacterium]	ptg002848l	45.902	6.41E-46	167	90
KAB2963888.1_MAG:_YpdA_family_putative_bacillithiol_disulfide_reductase_[Thermoanaerobaculia_bacterium]	ptg003861l	32.877	1.31E-08	60.5	41
KAB2963879.1_MAG:_dipeptidase_[Thermoanaerobaculia_bacterium]	ptg005697l	48.701	9.47E-130	418	97
KAB2963876.1_MAG:_TonB-dependent_receptor_[Thermoanaerobaculia_bacterium]	ptg001925l	29.392	5.21E-16	87.4	32
KAB2963874.1_MAG:_LysM_peptidoglycan-binding_domain-containing_protein_[Thermoanaerobaculia_bacterium]	ptg005398l	37.121	8.02E-22	104	23
KAB2963873.1_MAG:_3',5'-cyclic-nucleotide_phosphodiesterase_[Thermoanaerobaculia_bacterium]	ptg002760l	27.848	3.49E-08	57.8	89
KAB2963872.1_MAG:_GAF_domain-containing_protein_[Thermoanaerobaculia_bacterium]	ptg005301l	36.792	1.02E-32	135	48
KAB2963871.1_MAG:_BamA/TamA_family_outer_membrane_protein_[Thermoanaerobaculia_bacterium]	ptg003980l	25.792	0.000000651	57.8	20
KAB2963854.1_MAG:_magnesium_chelatase_[Thermoanaerobaculia_bacterium]	ptg003226l	37.736	1.3E-93	315	93
KAB2963853.1_MAG:_VWA_domain-containing_protein_[Thermoanaerobaculia_bacterium]	ptg003226l	30.982	6.83E-36	142	81
KAB2963852.1_MAG:_thioredoxin-disulfide_reductase_[Thermoanaerobaculia_bacterium]	ptg004538l	54.43	1.56E-95	313	97
KAB2963851.1_MAG:_ferritin_[Thermoanaerobaculia_bacterium]	ptg001693l	32.335	1.45E-18	86.3	89
KAB2963846.1_MAG:_hypothetical_protein_F9K16_05745_[Thermoanaerobaculia_bacterium]	ptg005375l	25.383	3.96E-27	123	84
KAB2963834.1_MAG:_aminoacetone_oxidase_family_FAD-binding_enzyme_[Thermoanaerobaculia_bacterium]	ptg002612l	32.298	1.53E-42	128	92
KAB2963833.1_MAG:_peptide_deformylase_[Thermoanaerobaculia_bacterium]	ptg004467l	34.524	2.12E-24	102	91
KAB2963831.1_MAG:_isoleucine--tRNA_ligase_[Thermoanaerobaculia_bacterium]	ptg002867l	38.389	0	733	97
KAB2963830.1_MAG:_phosphomannomutase/phosphoglucomutase_[Thermoanaerobaculia_bacterium]	ptg002505l	36.384	9.57E-74	256	95
KAB2963827.1_MAG:_zinc_metallopeptidase_[Thermoanaerobaculia_bacterium]	ptg000693l	55.556	4.52E-61	210	100

KAB2963824.1_MAG:_sigma-70_family_RNA_polymerase_sigma_factor,_partial_[Thermoanaerobaculia_bacterium]	ptg003901l	34.706	2.83E-16	79.7	87
KAB2963798.1_MAG:_amidohydrolase_family_protein_[Thermoanaerobaculia_bacterium]	ptg002699l	50.147	0	696	95
KAB2963792.1_MAG:_hypothetical_protein_F9K16_05835_[Thermoanaerobaculia_bacterium]	ptg003982l	26.95	3.87E-08	55.8	77
KAB2963791.1_MAG:_PAS_domain-containing_protein,_partial_[Thermoanaerobaculia_bacterium]	ptg004534l	25.214	2.3E-19	65.5	74
KAB2963789.1_MAG:_molybdopterin_molybdenumtransferase_MoeA,_partial_[Thermoanaerobaculia_bacterium]	ptg004000l	37.327	2.2E-24	104	91
KAB2963787.1_MAG:_hypothetical_protein_F9K18_08175_[Thermoanaerobaculia_bacterium]	ptg005433l	43.966	2.38E-33	134	69
KAB2963785.1_MAG:_glycosyltransferase_family_2_protein_[Thermoanaerobaculia_bacterium]	ptg003625l	30.769	1.8E-10	64.7	70
KAB2963783.1_MAG:_glycosyltransferase_family_4_protein_[Thermoanaerobaculia_bacterium]	ptg003753l	28.755	8.34E-15	80.5	58
KAB2963781.1_MAG:_glycosyltransferase_[Thermoanaerobaculia_bacterium]	ptg005770l	27.907	0.00000797	52	54
KAB2963780.1_MAG:_glycosyltransferase_family_4_protein_[Thermoanaerobaculia_bacterium]	ptg002534l	24.473	1.93E-12	72.8	59
KAB2963774.1_MAG:_hydroxymethylglutaryl-CoA_reductase_[Thermoanaerobaculia_bacterium]	ptg001917l	42.075	5.16E-84	288	67
KAB2963773.1_MAG:_amidohydrolase_family_protein,_partial_[Thermoanaerobaculia_bacterium]	ptg003746l	29.56	0.000001	54.3	47
KAB2963771.1_MAG:_GntR_family_transcriptional_regulator_[Thermoanaerobaculia_bacterium]	ptg004470l	36.842	3.82E-16	77	84
KAB2963769.1_MAG:_ABC_transporter_ATP-binding_protein_[Thermoanaerobaculia_bacterium]	ptg001571l	37.19	1.78E-33	82.8	82
KAB2963753.1_MAG:_hypothetical_protein_F9K16_05995_[Thermoanaerobaculia_bacterium]	ptg005309l	31.081	5.85E-09	63.9	20
KAB2963751.1_MAG:_CCA_tRNA_nucleotidyltransferase_[Thermoanaerobaculia_bacterium]	ptg004623l	35.714	2.27E-11	70.1	39
KAB2963750.1_MAG:_PilT/PilU_family_type_4a_pilus_ATPase_[Thermoanaerobaculia_bacterium]	ptg004468l	44.828	7.31E-91	306	74
KAB2963749.1_MAG:_transcriptional_repressor_LexA_[Thermoanaerobaculia_bacterium]	ptg002700l	39.901	1.08E-32	127	98
KAB2963748.1_MAG:_insulinase_family_protein_[Thermoanaerobaculia_bacterium]	ptg005195l	45.035	2.24E-77	265	66
KAB2963747.1_MAG:_insulinase_family_protein_[Thermoanaerobaculia_bacterium]	ptg005195l	30.822	1.31E-21	67.4	80
KAB2963739.1_MAG:_FAD-binding_protein,_partial_[Thermoanaerobaculia_bacterium]	ptg003861l	34.862	2.33E-36	155	22
KAB2963726.1_MAG:_ribonuclease_HII_[Thermoanaerobaculia_bacterium]	ptg002436l	42.935	6.96E-24	102	91
KAB2963725.1_MAG:_lytic_transglycosylase_domain-containing_protein_[Thermoanaerobaculia_bacterium]	ptg004256l	36.62	5.06E-15	76.6	57
KAB2963723.1_MAG:_50S_ribosomal_protein_L19_[Thermoanaerobaculia_bacterium]	ptg005431l	57.798	1.63E-34	129	89
KAB2963722.1_MAG:_tRNA_(guanosine(37)-N1)-methyltransferase_TrmD_[Thermoanaerobaculia_bacterium]	ptg005431l	43.22	2.45E-35	137	89
KAB2963719.1_MAG:_30S_ribosomal_protein_S16_[Thermoanaerobaculia_bacterium]	ptg005431l	44.444	3.57E-15	71.6	99
KAB2963718.1_MAG:_signal_recognition_particle_protein_[Thermoanaerobaculia_bacterium]	ptg005431l	45.542	5.71E-102	337	94
KAB2963717.1_MAG:_pyruvate_kinase_[Thermoanaerobaculia_bacterium]	ptg004880l	38.409	7.93E-79	272	88
KAB2963716.1_MAG:_rhomboid_family_intramembrane_serine_protease_[Thermoanaerobaculia_bacterium]	ptg005092l	34.951	4.29E-12	68.2	96
KAB2963714.1_MAG:_CDP-diacylglycerol--glycerol-3-phosphate_3-phosphatidyltransferase_[Thermoanaerobaculia_bacterium]	ptg003511l	36.641	1.8E-16	80.1	67
KAB2963713.1_MAG:_alanine--tRNA_ligase,_partial_[Thermoanaerobaculia_bacterium]	ptg002916l	42.574	6.18E-09	55.8	82
KAB2963711.1_MAG:_chromosome_segregation_protein_SMC,_partial_[Thermoanaerobaculia_bacterium]	ptg005087l	47.642	2.01E-49	184	51
KAB2963705.1_MAG:_aminomethyl-transferring_glycine_dehydrogenase_[Thermoanaerobaculia_bacterium]	ptg005318l	58.038	0	969	99
KAB2963704.1_MAG:_glycine_cleavage_system_protein_GcvH_[Thermoanaerobaculia_bacterium]	ptg005318l	38.333	4.66E-24	99.4	94

KAB2963703.1_MAG:_glycine_cleavage_system_aminomethyltransferase_GcvT_[Thermoanaerobaculia_bacterium]	ptg005299l	41.209	1.34E-76	260	98
KAB2963702.1_MAG:_DUF1501_domain-containing_protein_[Thermoanaerobaculia_bacterium]	ptg004266l	29.143	1.22E-24	111	74
KAB2963701.1_MAG:_DUF1800_domain-containing_protein_[Thermoanaerobaculia_bacterium]	ptg004266l	31.797	3.2E-15	83.6	36
KAB2963699.1_MAG:_carbon-nitrogen_hydrolase_[Thermoanaerobaculia_bacterium]	ptg002304l	31.2	4.35E-27	114	87
KAB2963698.1_MAG:_NAD+_synthase_[Thermoanaerobaculia_bacterium]	ptg002304l	36.652	7.61E-21	95.9	76
KAB2963696.1_MAG:_tRNA_2-thiouridine(34)_synthase_MnmA,_partial_[Thermoanaerobaculia_bacterium]	ptg001571l	52.83	8.27E-09	52.8	90
KAB2963686.1_MAG:_Lrp/AsnC_family_transcriptional_regulator_[Thermoanaerobaculia_bacterium]	ptg003131l	31.818	3.75E-18	84	93
KAB2963685.1_MAG:_peptide_chain_release_factor_N(5)-glutamine_methyltransferase_[Thermoanaerobaculia_bacterium]	ptg003336l	43.363	8.66E-39	150	62
KAB2963684.1_MAG:_UDP-N-acetylglucosamine_1-carboxyvinyltransferase_[Thermoanaerobaculia_bacterium]	ptg005611l	49.515	2.3E-86	172	99
KAB2963683.1_MAG:_HIT_domain-containing_protein_[Thermoanaerobaculia_bacterium]	ptg003220l	49.558	2.25E-19	85.1	100
KAB2963688.1_MAG:_hypothetical_protein_F9K16_06140,_partial_[Thermoanaerobaculia_bacterium]	ptg005929l	31.143	1.76E-12	77.4	16
KAB2963672.1_MAG:_Fic_family_protein_[Thermoanaerobaculia_bacterium]	ptg004254l	33.333	2.96E-32	132	75
KAB2963658.1_MAG:_hypothetical_protein_F9K18_08310,_partial_[Thermoanaerobaculia_bacterium]	ptg005527l	26.667	2.64E-18	93.2	55
KAB2963640.1_MAG:_single-stranded_DNA-binding_protein_[Thermoanaerobaculia_bacterium]	ptg005756l	39.048	1.53E-16	78.6	73
KAB2963636.1_MAG:_SRPBCC_family_protein_[Thermoanaerobaculia_bacterium]	ptg005844l	50	1.86E-30	118	100
KAB2963635.1_MAG:_helix-turn-helix_domain-containing_protein,_partial_[Thermoanaerobaculia_bacterium]	ptg001262l	41.772	2.89E-13	66.2	96
KAB2963634.1_MAG:_fasciclin_domain-containing_protein_[Thermoanaerobaculia_bacterium]	ptg003903l	54.545	2.14E-32	124	81
KAB2963632.1_MAG:_PaaI_family_thioesterase_[Thermoanaerobaculia_bacterium]	ptg003942l	36.496	1.48E-14	73.6	84
KAB2963630.1_MAG:_hypothetical_protein_F9K16_06285_[Thermoanaerobaculia_bacterium]	LG07	37.267	7.21E-13	77.4	69
KAB2963629.1_MAG:_hypothetical_protein_F9K16_06280_[Thermoanaerobaculia_bacterium]	LG07	35.865	2.87E-18	95.1	36
KAB2963628.1_MAG:_hypothetical_protein_F9K16_06275_[Thermoanaerobaculia_bacterium]	LG07	33.728	1.01E-11	73.6	45
KAB2963627.1_MAG:_hypothetical_protein_F9K16_06270_[Thermoanaerobaculia_bacterium]	LG07	45	4.68E-13	77.8	44
KAB2963620.1_MAG:_YebC/PmpR_family_DNA-binding_transcriptional_regulator_[Thermoanaerobaculia_bacterium]	ptg003903l	48.462	4.67E-32	127	54
KAB2963619.1_MAG:_mechanosensitive_ion_channel_family_protein_[Thermoanaerobaculia_bacterium]	ptg004724l	33.88	3.45E-16	84.3	49
KAB2963609.1_MAG:_thioredoxin_family_protein_[Thermoanaerobaculia_bacterium]	ptg002820l	27.174	7.11E-08	55.5	86
KAB2963608.1_MAG:_hypothetical_protein_F9K16_06340_[Thermoanaerobaculia_bacterium]	ptg001460l	41.667	4.05E-09	59.7	41
KAB2963605.1_MAG:_6-carboxytetrahydropterin_synthase_[Thermoanaerobaculia_bacterium]	ptg004575l	42.373	0.000000609	50.4	46
KAB2963604.1_MAG:_SLC13_family_permease_[Thermoanaerobaculia_bacterium]	ptg005195l	31.169	6.8E-20	98.6	47
KAB2963603.1_MAG:_uridine_kinase_[Thermoanaerobaculia_bacterium]	ptg001291l	41.463	6.8E-39	145	95
KAB2963602.1_MAG:_bifunctional_DNA-formamidopyrimidine_glycosylase/DNA-(apurinic_or_apyrimidinic_site)_lyase_[Thermoanaerobaculia_bacterium]	ptg002199l	40.364	2.29E-55	195	100
KAB2963598.1_MAG:_TraR/DksA_family_transcriptional_regulator_[Thermoanaerobaculia_bacterium]	ptg001644l	26.415	2.13E-09	57	88
KAB2963596.1_MAG:_acyl-CoA_dehydrogenase_[Thermoanaerobaculia_bacterium]	ptg004179l	26.748	1.99E-23	106	78

KAB2963595.1_MAG:_class_II_fructose-bisphosphatase_[Thermoanaerobaculia_bacterium]	ptg004284l	48.675	3.98E-66	229	91
KAB2963594.1_MAG:_dCTP_deaminase_[Thermoanaerobaculia_bacterium]	ptg002214l	33.333	1.78E-19	87.8	99
KAB2963589.1_MAG:_ACP_S-malonyltransferase_[Thermoanaerobaculia_bacterium]	ptg004340l	34.014	4.47E-41	155	94
KAB2963588.1_MAG:_3-oxoacyl-[acyl-carrier-protein]_reductase_[Thermoanaerobaculia_bacterium]	ptg003980l	50.413	2.42E-51	182	98
KAB2963587.1_MAG:_acyl_carrier_protein_[Thermoanaerobaculia_bacterium]	ptg003980l	58.667	8.2E-15	70.9	93
KAB2963586.1_MAG:_beta-ketoacyl-ACP_synthase_II_[Thermoanaerobaculia_bacterium]	ptg003980l	43.478	6.46E-83	281	88
KAB2963585.1_MAG:_phospholipase_D_family_protein_[Thermoanaerobaculia_bacterium]	ptg004779l	29.691	1.51E-40	161	86
KAB2963583.1_MAG:_hypothetical_protein_F9K16_06355_[Thermoanaerobaculia_bacterium]	ptg004340l	43.182	2.42E-17	84	60
KAB2963582.1_MAG:_serine/threonine_protein_kinase,_partial_[Thermoanaerobaculia_bacterium]	ptg003839l	38.462	2.57E-27	115	55
KAB2963570.1_MAG:_16S_rRNA_(guanine(966)-N(2))-methyltransferase_RsmD_[Thermoanaerobaculia_bacterium]	ptg004885l	36	5.2E-11	63.9	67
KAB2963568.1_MAG:_redox-sensing_transcriptional_repressor_Rex_[Thermoanaerobaculia_bacterium]	ptg005030l	43.299	1.78E-29	120	73
KAB2963567.1_MAG:_NADH-quinone_oxidoreductase_subunit_N_[Thermoanaerobaculia_bacterium]	ptg004143l	36.09	7.86E-42	163	58
KAB2963559.1_MAG:_lysophospholipid_acyltransferase_family_protein,_partial_[Thermoanaerobaculia_bacterium]	ptg003471l	25.907	5.61E-13	72	74
KAB2963558.1_MAG:_lipopolysaccharide_heptosyltransferase_II_[Thermoanaerobaculia_bacterium]	ptg004856l	31.818	0.0000012	54.3	39
KAB2963556.1_MAG:_peptidase_S41_[Thermoanaerobaculia_bacterium]	ptg003980l	25.815	6.32E-70	261	96
KAB2963554.1_MAG:_phosphopyruvate_hydratase_[Thermoanaerobaculia_bacterium]	ptg004466l	60.142	2.73E-157	495	98
KAB2963551.1_MAG:_MFS_transporter_[Thermoanaerobaculia_bacterium]	ptg003830l	28.197	1.09E-10	67.8	68
KAB2963550.1_MAG:_ABC_transporter_permease_subunit_[Thermoanaerobaculia_bacterium]	ptg005341l	32.71	2.3E-10	61.6	63
KAB2963549.1_MAG:_DUF1501_domain-containing_protein,_partial_[Thermoanaerobaculia_bacterium]	ptg004266l	29.073	1.29E-18	91.7	81
KAB2963547.1_MAG:_carbon-nitrogen_hydrolase_[Thermoanaerobaculia_bacterium]	ptg002304l	28.8	1.05E-20	95.5	89
KAB2963546.1_MAG:_NAD+_synthase_[Thermoanaerobaculia_bacterium]	ptg002304l	37.778	2.9E-30	123	77
KAB2963545.1_MAG:_hypothetical_protein_F9K16_06495,_partial_[Thermoanaerobaculia_bacterium]	ptg003813l	27.476	1.62E-102	361	87
KAB2963544.1_MAG:_hypothetical_protein_F9K16_06490_[Thermoanaerobaculia_bacterium]	ptg005108l	41.966	6.93E-112	379	65
KAB2963542.1_MAG:_malate_dehydrogenase_[Thermoanaerobaculia_bacterium]	ptg004470l	61.846	1.58E-109	353	99
KAB2963537.1_MAG:_SDR_family_oxidoreductase_[Thermoanaerobaculia_bacterium]	ptg003980l	34.4	2.01E-34	135	92
KAB2963535.1_MAG:_thiolase_family_protein_[Thermoanaerobaculia_bacterium]	ptg004745l	41.192	1.3E-63	224	97
KAB2963533.1_MAG:_NAD-dependent_deacylase_[Thermoanaerobaculia_bacterium]	ptg004476l	35.577	9.99E-31	123	77
KAB2963519.1_MAG:_DNA_gyrase_subunit_A_[Thermoanaerobaculia_bacterium]	ptg003780l	47.986	0	740	96
KAB2963517.1_MAG:_sigma-70_family_RNA_polymerase_sigma_factor_[Thermoanaerobaculia_bacterium]	ptg004340l	32.934	6.2E-14	75.1	65
KAB2963515.1_MAG:_acetate--CoA_ligase_[Thermoanaerobaculia_bacterium]	ptg005005l	62.48	0	750	95
KAB2963510.1_MAG:_DNA_topoisomerase_(ATP-hydrolyzing)_subunit_B_[Thermoanaerobaculia_bacterium]	ptg000372l	63.187	0	645	99
KAB2963525.1_MAG:_protein-L-isoaspartate(D-aspartate)_O-methyltransferase_[Thermoanaerobaculia_bacterium]	ptg004026l	44	8.32E-45	162	96
KAB2963524.1_MAG:_5'/3'-nucleotidase_SurE_[Thermoanaerobaculia_bacterium]	ptg005398l	38.976	9.06E-48	173	92
KAB2963522.1_MAG:_adenine_phosphoribosyltransferase_[Thermoanaerobaculia_bacterium]	ptg004306l	52.941	3.91E-47	167	99

KAB2963521.1_MAG:_hypothetical_protein_F9K18_08475_[Thermoanaerobaculia_bacterium]	ptg002660l	27.297	2.45E-15	82.8	84
KAB2963500.1_MAG:_NAD(P)-dependent_oxidoreductase_[Thermoanaerobaculia_bacterium]	ptg001690l	28.319	4.56E-13	73.6	97
KAB2963498.1_MAG:_transketolase,_partial_[Thermoanaerobaculia_bacterium]	ptg004987l	51.232	1.27E-58	206	69
KAB2963497.1_MAG:_glutamate--tRNA_ligase_[Thermoanaerobaculia_bacterium]	ptg004724l	52.918	6.12E-155	493	97
KAB2963496.1_MAG:_glutamine--tRNA_ligase/YqeY_domain_fusion_protein_[Thermoanaerobaculia_bacterium]	ptg005010l	59.055	0	641	91
KAB2963493.1_MAG:_2-oxoglutarate_dehydrogenase_E1_component_[Thermoanaerobaculia_bacterium]	ptg000492l	44.788	0	604	94
KAB2963492.1_MAG:_protoporphyrinogen_oxidase,_partial_[Thermoanaerobaculia_bacterium]	ptg003764l	35.909	9.58E-21	95.1	82
KAB2963490.1_MAG:_hypothetical_protein_F9K18_08535_[Thermoanaerobaculia_bacterium]	ptg005227l	25.269	5.99E-18	92	65
KAB2963489.1_MAG:_Hsp20/alpha_crystallin_family_protein_[Thermoanaerobaculia_bacterium]	ptg002102l	30.952	3.89E-12	66.2	83
KAB2963488.1_MAG:_hypothetical_protein_F9K18_08525_[Thermoanaerobaculia_bacterium]	ptg001025l	33.333	1.08E-10	68.6	18
KAB2963472.1_MAG:_EAL_domain-containing_protein_[Thermoanaerobaculia_bacterium]	ptg002820l	27.54	1.92E-40	166	52
KAB2963471.1_MAG:_phosphoenolpyruvate_carboxykinase_(ATP)_[Thermoanaerobaculia_bacterium]	ptg005856l	51.663	4.37E-158	503	95
KAB2963470.1_MAG:_Glu/Leu/Phe/Val_dehydrogenase_[Thermoanaerobaculia_bacterium]	ptg004284l	47.881	1.4E-139	446	99
KAB2963469.1_MAG:_pentapeptide_repeat-containing_protein_[Thermoanaerobaculia_bacterium]	ptg004266l	31.061	1.06E-11	65.5	79
KAB2963467.1_MAG:_response_regulator,_partial_[Thermoanaerobaculia_bacterium]	ptg001878l	37.662	0.000000507	48.5	93
KAB2963480.1_MAG:_TIGR00730_family_Rossman_fold_protein_[Thermoanaerobaculia_bacterium]	ptg003168l	46.711	1.14E-18	87	77
KAB2963479.1_MAG:_tRNA_preQ1(34)_S-adenosylmethionine_ribosyltransferase-isomerase_QueA_[Thermoanaerobaculia_bacterium]	ptg004256l	45.906	2.33E-67	233	98
KAB2963478.1_MAG:_ABC_transporter_permease_[Thermoanaerobaculia_bacterium]	ptg003131l	39.837	1.51E-21	99.8	36
KAB2963477.1_MAG:_ABC_transporter_permease_[Thermoanaerobaculia_bacterium]	ptg003163l	32.365	2.58E-11	68.6	75
KAB2963455.1_MAG:_glycosyl_transferase_family_36,_partial_[Thermoanaerobaculia_bacterium]	ptg004790l	35	9.03E-19	89.7	56
KAB2963454.1_MAG:_beta-glucosidase_[Thermoanaerobaculia_bacterium]	LG20	45.794	5.57E-19	94	36
KAB2963453.1_MAG:_adenosylhomocysteinase_[Thermoanaerobaculia_bacterium]	ptg002467l	71.138	0	716	99
KAB2963446.1_MAG:_P-type_conjugative_transfer_ATPase_TrbB_[Thermoanaerobaculia_bacterium]	ptg003304l	35.686	4.61E-35	144	45
KAB2963445.1_MAG:_type_IV_secretory_system_conjugative_DNA_transfer_family_protein_[Thermoanaerobaculia_bacterium]	ptg004058l	30.372	3.43E-30	129	63
KAB2963444.1_MAG:_relaxase_domain-containing_protein_[Thermoanaerobaculia_bacterium]	ptg005384l	26.217	5.2E-26	120	62
KAB2963443.1_MAG:_sugar_kinase,_partial_[Thermoanaerobaculia_bacterium]	ptg002916l	33.962	0.000000127	56.6	91
KAB2963442.1_MAG:_sigma-70_family_RNA_polymerase_sigma_factor_[Thermoanaerobaculia_bacterium]	ptg004479l	30.178	9.74E-22	96.3	81
KAB2963439.1_MAG:_M23_family_metallopeptidase_[Thermoanaerobaculia_bacterium]	ptg000819l	33.846	5.46E-42	163	57
KAB2963432.1_MAG:_hypothetical_protein_F9K16_06700_[Thermoanaerobaculia_bacterium]	ptg005318l	30.097	1.13E-10	62.8	61
KAB2963429.1_MAG:_4Fe-4S_dicluster_domain-containing_protein_[Thermoanaerobaculia_bacterium]	ptg004084l	33.951	7.62E-18	87.4	56
KAB2963427.1_MAG:_response_regulator_[Thermoanaerobaculia_bacterium]	ptg001025l	31.034	1.23E-14	80.1	29
KAB2963426.1_MAG:_HAMP_domain-containing_protein_[Thermoanaerobaculia_bacterium]	ptg003137l	31.832	9.06E-41	161	60

KAB2963424.1_MAG:_heme_A_synthase_[Thermoanaerobaculia_bacterium]	ptg000819l	46.364	7.68E-19	82.4	35
KAB2963423.1_MAG:_methylated-DNA--[protein]-cysteine_S-methyltransferase_[Thermoanaerobaculia_bacterium]	ptg005680l	38.854	9.89E-20	94.7	63
KAB2963421.1_MAG:_subclass_B3_metallo-beta-lactamase_[Thermoanaerobaculia_bacterium]	ptg001644l	26.923	1.94E-08	59.3	49
KAB2963419.1_MAG:_hypothetical_protein_F9K18_08620,_partial_[Thermoanaerobaculia_bacterium]	ptg001128l	43.363	1.18E-20	91.3	68
KAB2963411.1_MAG:_selenide,_water_dikinase_SelD_[Thermoanaerobaculia_bacterium]	ptg001871l	37.453	7.11E-17	85.9	75
KAB2963409.1_MAG:_glucose-1-phosphate_adenylyltransferase_[Thermoanaerobaculia_bacterium]	ptg002878l	47.059	1.71E-112	366	99
KAB2963408.1_MAG:_glycogen_synthase_[Thermoanaerobaculia_bacterium]	ptg003785l	38.431	4.55E-105	347	99
KAB2963407.1_MAG:_PQQ-dependent_sugar_dehydrogenase_[Thermoanaerobaculia_bacterium]	ptg006020l	41.333	3.07E-41	162	58
KAB2963406.1_MAG:_4Fe-4S_binding_protein,_partial_[Thermoanaerobaculia_bacterium]	ptg005961l	30.952	0.00000111	53.1	32
KAB2963405.1_MAG:_type_IV_pilus_secretin_PilQ,_partial_[Thermoanaerobaculia_bacterium]	ptg004863l	34.375	5.25E-61	219	89
KAB2963400.1_MAG:_ABC_transporter_ATP-binding_protein_[Thermoanaerobaculia_bacterium]	ptg001659l	45.333	6.17E-59	204	92
KAB2963398.1_MAG:_dipeptidase_PepE_[Thermoanaerobaculia_bacterium]	ptg003603l	54	7.64E-59	204	85
KAB2963397.1_MAG:_PLP-dependent_cysteine_synthase_family_protein_[Thermoanaerobaculia_bacterium]	ptg001170l	35.906	3.2E-40	153	90
KAB2963396.1_MAG:_SDR_family_oxidoreductase_[Thermoanaerobaculia_bacterium]	ptg003980l	36.022	9.83E-23	100	69
KAB2963394.1_MAG:_adenosylhomocysteinase_[Thermoanaerobaculia_bacterium]	ptg002467l	69.556	0	712	100
KAB2963393.1_MAG:_DUF1820_family_protein_[Thermoanaerobaculia_bacterium]	ptg004790l	47.525	2.2E-27	108	86
KAB2963392.1_MAG:_ABC_transporter_permease_[Thermoanaerobaculia_bacterium]	ptg004986l	27.132	7.33E-25	111	85
KAB2963391.1_MAG:_ABC_transporter_permease_[Thermoanaerobaculia_bacterium]	ptg004986l	28.009	9.8E-11	68.2	99
KAB2963383.1_MAG:_hypothetical_protein_F9K18_08670,_partial_[Thermoanaerobaculia_bacterium]	ptg004854l	38.298	2.61E-16	89.4	30
KAB2963379.1_MAG:_CocE/NonD_family_hydrolase_[Thermoanaerobaculia_bacterium]	ptg003785l	34.817	4.68E-94	322	92
KAB2963370.1_MAG:_GDP-mannose_4,6-dehydratase_[Thermoanaerobaculia_bacterium]	ptg006001l	35.78	9.14E-44	164	96
KAB2963369.1_MAG:_dTDP-4-dehydrorhamnose_reductase_[Thermoanaerobaculia_bacterium]	ptg003827l	39.407	4.7E-36	140	79
KAB2963356.1_MAG:_aminotransferase_class_I/II-fold_pyridoxal_phosphate-dependent_enzyme_[Thermoanaerobaculia_bacterium]	ptg004905l	29.134	2.45E-21	100	62
KAB2963355.1_MAG:_protein_kinase,_partial_[Thermoanaerobaculia_bacterium]	ptg003082l	36.331	7.01E-41	169	22
KAB2963352.1_MAG:_deoxynucleoside_kinase_[Thermoanaerobaculia_bacterium]	ptg004203l	27.273	1.71E-10	63.5	72
KAB2963351.1_MAG:_zinc_ribbon_domain-containing_protein_[Thermoanaerobaculia_bacterium]	ptg004010l	38.667	0.00000835	44.7	97
KAB2963350.1_MAG:_aminofutalosine_synthase_MqnE_[Thermoanaerobaculia_bacterium]	ptg003903l	53.495	9.27E-102	333	89
KAB2963359.1_MAG:_FHA_domain-containing_protein_[Thermoanaerobaculia_bacterium]	ptg004055l	32.394	5.94E-18	92	38
KAB2963358.1_MAG:_adenylosuccinate_lyase_[Thermoanaerobaculia_bacterium]	ptg002345l	33.921	8.65E-46	175	94
KAB2963357.1_MAG:_septal_ring_lytic_transglycosylase_RlpA_family_protein,_partial_[Thermoanaerobaculia_bacterium]	ptg005398l	44.531	3.54E-28	114	57
KAB2963330.1_MAG:_hypothetical_protein_F9K16_06920_[Thermoanaerobaculia_bacterium]	ptg005839l	31.757	0.00000194	54.3	29
KAB2963328.1_MAG:_serine/threonine_protein_kinase_[Thermoanaerobaculia_bacterium]	ptg003220l	31.538	1.76E-19	97.4	40

KAB2963325.1_MAG:_hypothetical_protein_F9K16_06895_[Thermoanaerobaculia_bacterium]	ptg002660l	27.344	4.84E-15	81.6	84
KAB2963323.1_MAG:_amino_acid_permease,_partial_[Thermoanaerobaculia_bacterium]	ptg004214l	31.579	6.26E-12	68.6	73
KAB2963322.1_MAG:_ion_transporter_[Thermoanaerobaculia_bacterium]	ptg002436l	51.982	1.63E-51	184	82
KAB2963310.1_MAG:_electron_transport_complex_subunit_RsxC_[Thermoanaerobaculia_bacterium]	ptg002214l	36.219	5.22E-74	256	97
KAB2963309.1_MAG:_RnfABCDGE_type_electron_transport_complex_subunit_D_[Thermoanaerobaculia_bacterium]	ptg002214l	46.269	2.37E-13	75.1	20
KAB2963307.1_MAG:_RnfABCDGE_type_electron_transport_complex_subunit_E_[Thermoanaerobaculia_bacterium]	ptg002192l	37.821	5.83E-26	108	72
KAB2963306.1_MAG:_electron_transport_complex_subunit_RsxA_[Thermoanaerobaculia_bacterium]	ptg004538l	44.737	3.35E-30	119	92
KAB2963305.1_MAG:_UDP-glucose/GDP-mannose_dehydrogenase_family_protein_[Thermoanaerobaculia_bacterium]	ptg001925l	45.147	1.25E-112	367	99
KAB2963304.1_MAG:_SDR_family_oxidoreductase_[Thermoanaerobaculia_bacterium]	ptg005433l	53.846	5.8E-111	357	98
KAB2963303.1_MAG:_TVP38/TMEM64_family_protein_[Thermoanaerobaculia_bacterium]	ptg005017l	27.53	2.43E-08	58.5	87
KAB2963301.1_MAG:_hypothetical_protein_F9K16_06935_[Thermoanaerobaculia_bacterium]	ptg002660l	25.51	0.00000119	55.1	55
KAB2963300.1_MAG:_hypothetical_protein_F9K16_06930,_partial_[Thermoanaerobaculia_bacterium]	ptg005354l	35.26	8.24E-28	113	86
KAB2963313.1_MAG:_1-acyl-sn-glycerol-3-phosphate_acyltransferase_[Thermoanaerobaculia_bacterium]	ptg002123l	31.447	2.28E-14	75.9	63
KAB2963312.1_MAG:_insulinase_family_protein,_partial_[Thermoanaerobaculia_bacterium]	ptg005195l	31.222	5.86E-22	100	67
KAB2963293.1_MAG:_OmpA_family_protein_[Thermoanaerobaculia_bacterium]	ptg003691l	34.454	1.11E-11	68.6	45
KAB2963291.1_MAG:_YgeY_family_selenium_metabolism-linked_hydrolase_[Thermoanaerobaculia_bacterium]	ptg002179l	29.63	6.05E-28	120	90
KAB2963282.1_MAG:_MBL_fold_metallo-hydrolase_[Thermoanaerobaculia_bacterium]	ptg004856l	37.619	9.65E-38	142	97
KAB2963278.1_MAG:_ABC_transporter_ATP-binding_protein_[Thermoanaerobaculia_bacterium]	ptg004986l	32.864	8.35E-25	109	61
KAB2963274.1_MAG:_2-isopropylmalate_synthase_[Thermoanaerobaculia_bacterium]	ptg002871l	31.204	7.71E-35	140	95
KAB2963273.1_MAG:_3-isopropylmalate_dehydrogenase_[Thermoanaerobaculia_bacterium]	ptg001005l	37.38	5.74E-39	151	82
KAB2963272.1_MAG:_YggS_family_pyridoxal_phosphate-dependent_enzyme_[Thermoanaerobaculia_bacterium]	ptg005005l	44.318	2.29E-21	65.9	83
KAB2963268.1_MAG:_lytic_transglycosylase_domain-containing_protein,_partial_[Thermoanaerobaculia_bacterium]	ptg004256l	35.032	3.52E-23	106	38
KAB2963262.1_MAG:_zinc-binding_dehydrogenase_[Thermoanaerobaculia_bacterium]	ptg004026l	31.063	8.62E-29	121	96
KAB2963260.1_MAG:_3-hydroxyacyl-CoA_dehydrogenase_[Thermoanaerobaculia_bacterium]	ptg002612l	44.388	7.15E-49	177	67
KAB2963257.1_MAG:_serine/threonine_protein_kinase_[Thermoanaerobaculia_bacterium]	ptg006032l	28.671	6.42E-23	109	33
KAB2963253.1_MAG:_serine/threonine_protein_kinase,_partial_[Thermoanaerobaculia_bacterium]	ptg002926l	37.597	3.13E-46	169	89
KAB2963252.1_MAG:_response_regulator_transcription_factor_[Thermoanaerobaculia_bacterium]	ptg003829l	31.22	1.02E-19	92	77
KAB2963242.1_MAG:_glycosyltransferase_family_39_protein,_partial_[Thermoanaerobaculia_bacterium]	ptg003471l	30.328	1.46E-14	79.7	80
KAB2963241.1_MAG:_glycosyltransferase_family_2_protein_[Thermoanaerobaculia_bacterium]	ptg004788l	44.105	3.55E-51	182	91
KAB2963239.1_MAG:_NAD-dependent_epimerase/dehydratase_family_protein,_partial_[Thermoanaerobaculia_bacterium]	ptg003768l	31.861	2.79E-34	139	74
KAB2963236.1_MAG:_heavy_metal_translocating_P-type_ATPase_[Thermoanaerobaculia_bacterium]	ptg003880l	33.611	2.68E-62	233	72
KAB2963234.1_MAG:_efflux_RND_transporter_permease_subunit_[Thermoanaerobaculia_bacterium]	ptg004670l	33.005	4.3E-159	528	99
KAB2963233.1_MAG:_efflux_RND_transporter_periplasmic_adaptor_subunit_[Thermoanaerobaculia_bacterium]	ptg001766l	29.6	2.9E-37	148	85
KAB2963228.1_MAG:_hypothetical_protein_F9K18_08865,_partial_[Thermoanaerobaculia_bacterium]	ptg004724l	27.642	1.55E-10	68.2	58

KAB2963227.1_MAG:_PP2C_family_protein-serine/threonine_phosphatase_[Thermoanaerobaculia_bacterium]	ptg004476l	30.899	6.18E-11	68.2	45
KAB2963226.1_MAG:_transcriptional_repressor_[Thermoanaerobaculia_bacterium]	ptg003304l	31.707	0.00000217	48.9	59
KAB2963221.1_MAG:_2-oxoglutarate_oxidoreductase_[Thermoanaerobaculia_bacterium]	ptg002102l	45.327	1.32E-58	206	67
KAB2963220.1_MAG:_2-oxoacid:acceptor_oxidoreductase_subunit_alpha_[Thermoanaerobaculia_bacterium]	ptg002867l	32.222	1.13E-77	273	94
KAB2963218.1_MAG:_NAD-dependent_epimerase/dehydratase_family_protein_[Thermoanaerobaculia_bacterium]	ptg004982l	38.182	4.23E-73	248	97
KAB2963216.1_MAG:_ABC_transporter_ATP-binding_protein_[Thermoanaerobaculia_bacterium]	ptg003336l	38	3.09E-41	154	96
KAB2963215.1_MAG:_MlaE_family_lipid_ABC_transporter_permease_subunit_[Thermoanaerobaculia_bacterium]	ptg003336l	42.781	3.88E-31	135	24
KAB2963214.1_MAG:_Mrp/NBP35_family_ATP-binding_protein,_partial_[Thermoanaerobaculia_bacterium]	ptg002214l	52.133	2.72E-63	221	60
KAB2963213.1_MAG:_acetylglutamate_kinase,_partial_[Thermoanaerobaculia_bacterium]	ptg004476l	37.895	1.52E-14	72.4	73
KAB2963212.1_MAG:_N-acetylornithine_carbamoyltransferase_[Thermoanaerobaculia_bacterium]	ptg003583l	28.571	1.57E-25	111	89
KAB2963211.1_MAG:_aspartate_aminotransferase_family_protein_[Thermoanaerobaculia_bacterium]	ptg002126l	35.739	4.36E-35	141	73
KAB2963210.1_MAG:_N-acetyl-gamma-glutamyl-phosphate_reductase_[Thermoanaerobaculia_bacterium]	ptg004077l	31.136	1.13E-24	111	75
KAB2963205.1_MAG:_VWA_domain-containing_protein_[Thermoanaerobaculia_bacterium]	ptg003226l	31.861	1.01E-33	136	77
KAB2963204.1_MAG:_thioredoxin-disulfide_reductase_[Thermoanaerobaculia_bacterium]	ptg005689l	57.705	1.01E-95	313	95
KAB2963202.1_MAG:_CDP-diacylglycerol--serine_O-phosphatidyltransferase_[Thermoanaerobaculia_bacterium]	ptg002304l	39.189	1.13E-41	155	86
KAB2963190.1_MAG:_DNA_mismatch_repair_protein_MutS_[Thermoanaerobaculia_bacterium]	ptg001460l	43.759	4.97E-136	452	90
KAB2963189.1_MAG:_beta-N-acetylhexosaminidase,_partial_[Thermoanaerobaculia_bacterium]	ptg005474l	30.909	2.44E-09	62	72
KAB2963186.1_MAG:_efflux_RND_transporter_periplasmic_adaptor_subunit_[Thermoanaerobaculia_bacterium]	ptg000732l	24.845	3.85E-14	77.4	47
KAB2963185.1_MAG:_efflux_RND_transporter_permease_subunit_[Thermoanaerobaculia_bacterium]	ptg003168l	30.667	1.36E-104	150	90
KAB2963184.1_MAG:_selenide,_water_dikinase_SelD_[Thermoanaerobaculia_bacterium]	ptg001871l	36.585	1.67E-38	149	98
KAB2963183.1_MAG:_DUF885_domain-containing_protein_[Thermoanaerobaculia_bacterium]	ptg001995l	34.229	5.38E-84	290	91
KAB2963161.1_MAG:_hypothetical_protein_F9K16_07305,_partial_[Thermoanaerobaculia_bacterium]	ptg005527l	38.503	5.46E-26	108	87
KAB2963160.1_MAG:_long-chain_fatty_acid--CoA_ligase_[Thermoanaerobaculia_bacterium]	ptg005087l	31.595	3.15E-29	127	53
KAB2963158.1_MAG:_MFS_transporter_[Thermoanaerobaculia_bacterium]	ptg003971l	28.221	2.53E-09	63.5	37
KAB2963157.1_MAG:_3-phosphoserine/phosphohydroxythreonine_transaminase_[Thermoanaerobaculia_bacterium]	ptg005318l	48.229	1.7E-101	332	99
KAB2963155.1_MAG:_hydroxyacid_dehydrogenase_[Thermoanaerobaculia_bacterium]	ptg001693l	48.555	3.45E-87	290	97
KAB2963154.1_MAG:_DUF1015_domain-containing_protein,_partial_[Thermoanaerobaculia_bacterium]	ptg001693l	53.254	4.49E-51	179	91
KAB2963153.1_MAG:_cupin_domain-containing_protein_[Thermoanaerobaculia_bacterium]	ptg005711l	32.117	3.17E-12	67	81
KAB2963151.1_MAG:_peroxiredoxin_[Thermoanaerobaculia_bacterium]	ptg004538l	34.127	1.65E-15	77	67
KAB2963150.1_MAG:_nucleoside-diphosphate_kinase_[Thermoanaerobaculia_bacterium]	ptg002916l	41.727	6.94E-31	119	99
KAB2963144.1_MAG:_TrbI/VirB10_family_protein_[Thermoanaerobaculia_bacterium]	ptg005137l	37.179	1.21E-23	90.5	38
KAB2963143.1_MAG:_hypothetical_protein_F9K16_07340_[Thermoanaerobaculia_bacterium]	ptg005137l	34.653	0.000000568	55.5	29

KAB2963127.1_MAG:_bifunctional_methionine_sulfoxide_reductase_B/A_protein_[Thermoanaerobaculia_bacterium]	ptg002618l	59.028	8.9E-52	189	81
KAB2963122.1_MAG:_branched-chain_amino_acid_transaminase_[Thermoanaerobaculia_bacterium]	ptg002658l	52.632	2.37E-79	160	95
KAB2963120.1_MAG:_molecular_chaperone_DnaJ_[Thermoanaerobaculia_bacterium]	ptg003680l	44.959	5.72E-61	215	97
KAB2963118.1_MAG:_type_IV_pilus_twitching_motility_protein_PilT_[Thermoanaerobaculia_bacterium]	ptg000568l	45.429	5.99E-78	265	89
KAB2963116.1_MAG:_molecular_chaperone_DnaK_[Thermoanaerobaculia_bacterium]	ptg005922l	53.055	0	642	98
KAB2963115.1_MAG:_nucleotide_exchange_factor_GrpE_[Thermoanaerobaculia_bacterium]	ptg003680l	37.226	4.16E-24	103	61
KAB2963106.1_MAG:_beta-ketoacyl-ACP_synthase_II_[Thermoanaerobaculia_bacterium]	ptg003980l	44.16	5.09E-78	266	84
KAB2963105.1_MAG:_acyl_carrier_protein_[Thermoanaerobaculia_bacterium]	ptg003980l	62.667	6.9E-16	73.9	93
KAB2963104.1_MAG:_3-oxoacyl-[acyl-carrier-protein]_reductase_[Thermoanaerobaculia_bacterium]	ptg005905l	49.174	7.89E-56	196	98
KAB2963100.1_MAG:_acyl-CoA_dehydrogenase_[Thermoanaerobaculia_bacterium]	ptg005087l	49	4.03E-174	551	99
KAB2963097.1_MAG:_hypothetical_protein_F9K16_07410_[Thermoanaerobaculia_bacterium]	ptg001170l	51.163	6.56E-21	104	7
KAB2963081.1_MAG:_tyrosine_recombinase_XerC_[Thermoanaerobaculia_bacterium]	ptg003090l	44.262	2.24E-58	206	97
KAB2963078.1_MAG:_hypothetical_protein_F9K16_07465_[Thermoanaerobaculia_bacterium]	ptg006031l	31.41	7.86E-15	77	62
KAB2963075.1_MAG:_sigma-54-dependent_Fis_family_transcriptional_regulator_[Thermoanaerobaculia_bacterium]	ptg003057l	40.089	4.06E-72	251	98
KAB2963074.1_MAG:_hypothetical_protein_F9K16_07445_[Thermoanaerobaculia_bacterium]	ptg005886l	33.81	8.22E-15	81.6	39
KAB2963073.1_MAG:_cytochrome_c_biogenesis_protein_ResB,_partial_[Thermoanaerobaculia_bacterium]	ptg001658l	30	7.84E-09	62	26
KAB2963066.1_MAG:_ATP-binding_protein_[Thermoanaerobaculia_bacterium]	ptg003864l	27.203	6.92E-13	75.9	42
KAB2963060.1_MAG:_lipid-A-disaccharide_synthase,_partial_[Thermoanaerobaculia_bacterium]	ptg002436l	38.65	1E-38	98.6	85
KAB2963057.1_MAG:_integration_host_factor_subunit_beta_[Thermoanaerobaculia_bacterium]	ptg003072l	46.154	5.86E-15	72.4	85
KAB2963056.1_MAG:_30S_ribosomal_protein_S1_[Thermoanaerobaculia_bacterium]	ptg001025l	47.007	1.82E-130	424	90
KAB2963047.1_MAG:_4-hydroxythreonine-4-phosphate_dehydrogenase_PdxA_[Thermoanaerobaculia_bacterium]	ptg001690l	33.945	1.03E-37	147	92
KAB2963046.1_MAG:_hypothetical_protein_F9K16_07555_[Thermoanaerobaculia_bacterium]	ptg000619l	43.046	2.65E-34	131	83
KAB2963044.1_MAG:_hypothetical_protein_F9K16_07590_[Thermoanaerobaculia_bacterium]	ptg005260l	26.117	1.29E-12	75.9	34
KAB2963043.1_MAG:_prepilin-type_N-terminal_cleavage/methylation_domain-containing_protein_[Thermoanaerobaculia_bacterium]	ptg002871l	29.592	4.09E-08	54.3	67
KAB2963041.1_MAG:_dUTP_diphosphatase_[Thermoanaerobaculia_bacterium]	ptg002199l	45.6	2.81E-25	103	83
KAB2963039.1_MAG:_phosphoribosylamine--glycine_ligase_[Thermoanaerobaculia_bacterium]	ptg002175l	43.632	2.46E-91	306	93
KAB2963038.1_MAG:_phosphate_acetyltransferase,_partial_[Thermoanaerobaculia_bacterium]	ptg004584l	43.624	1.61E-20	94.4	56
KAB2963050.1_MAG:_tryptophanase_[Thermoanaerobaculia_bacterium]	ptg004361l	38.137	3.64E-94	317	89
KAB2963049.1_MAG:_serine/threonine_protein_kinase,_partial_[Thermoanaerobaculia_bacterium]	ptg005260l	43.46	5.16E-46	169	77
KAB2963032.1_MAG:_UDP-3-O-[3-hydroxymyristoyl]_N-acetylglucosamine_deacetylase,_partial_[Thermoanaerobaculia_bacterium]	ptg004510l	36.704	2.23E-39	150	89
KAB2963030.1_MAG:_aminotransferase_class_III-fold_pyridoxal_phosphate-dependent_enzyme_[Thermoanaerobaculia_bacterium]	ptg002773l	41.348	3.41E-106	353	97
KAB2963029.1_MAG:_GTPase_Era_[Thermoanaerobaculia_bacterium]	ptg002902l	41.627	5.85E-45	148	80

KAB2963028.1_MAG:_CBS_domain-containing_protein_[Thermoanaerobaculia_bacterium]	ptg005772l	35.217	2.96E-31	130	53
KAB2963026.1_MAG:_PhoH_family_protein_[Thermoanaerobaculia_bacterium]	ptg003708l	51.495	3.07E-80	269	93
KAB2963025.1_MAG:_3-hydroxybutyryl-CoA_dehydrogenase_[Thermoanaerobaculia_bacterium]	ptg002612l	40.214	6.13E-53	189	99
KAB2963024.1_MAG:_tRNA_(adenosine(37)-N6)-threonylcarbamoyltransferase_complex_ATPase_subunit_type_1_TsaE_[Thermoanaerobaculia_bacterium]	ptg002316l	38.849	9.33E-12	64.7	96
KAB2963023.1_MAG:_NAD(P)H-hydrate_dehydratase,_partial_[Thermoanaerobaculia_bacterium]	ptg001878l	42.105	4.21E-25	106	78
KAB2963014.1_MAG:_thiamine_pyrophosphate-dependent_dehydrogenase_E1_component_subunit_alpha_[Thermoanaerobaculia_bacterium]	ptg004010l	27.068	1.43E-12	72.4	79
KAB2963013.1_MAG:_dihydrolipoyl_dehydrogenase_[Thermoanaerobaculia_bacterium]	ptg003780l	42.188	1.06E-99	332	94
KAB2963012.1_MAG:_alpha-ketoacid_dehydrogenase_subunit_beta_[Thermoanaerobaculia_bacterium]	ptg002612l	31.915	1.96E-38	148	98
KAB2963011.1_MAG:_2-oxoglutarate_dehydrogenase,_partial_[Thermoanaerobaculia_bacterium]	ptg003019l	42.667	1.55E-12	64.7	82
KAB2963009.1_MAG:_hypothetical_protein_F9K16_07685_[Thermoanaerobaculia_bacterium]	ptg002907l	30.27	0.000000191	58.2	32
KAB2963008.1_MAG:_DegT/DnrJ/EryC1/StrS_family_aminotransferase_[Thermoanaerobaculia_bacterium]	ptg003134l	42.737	1.92E-64	226	94
KAB2963005.1_MAG:_M20/M25/M40_family_metallo-hydrolase_[Thermoanaerobaculia_bacterium]	ptg002179l	27.174	8.37E-11	68.9	70
KAB2963004.1_MAG:_methyltransferase_domain-containing_protein_[Thermoanaerobaculia_bacterium]	ptg001460l	36.735	0.000000235	53.9	50
KAB2962995.1_MAG:_glycosyltransferase_family_2_protein_[Thermoanaerobaculia_bacterium]	ptg002999l	42.593	3.52E-46	171	66
KAB2962994.1_MAG:_aminomethyl-transferring_glycine_dehydrogenase,_partial_[Thermoanaerobaculia_bacterium]	ptg005318l	58.842	0	676	99
KAB2962987.1_MAG:_DnaJ_domain-containing_protein,_partial_[Thermoanaerobaculia_bacterium]	ptg003680l	55.072	3.65E-16	78.2	43
KAB2962984.1_MAG:_ferredoxin_family_protein_[Thermoanaerobaculia_bacterium]	ptg003928l	50.667	5.2E-17	77	89
KAB2962976.1_MAG:_AbgT_family_transporter_[Thermoanaerobaculia_bacterium]	ptg003282l	52.459	2.18E-113	373	91
KAB2962967.1_MAG:_NAD-dependent_DNA_ligase_LigA_[Thermoanaerobaculia_bacterium]	ptg005087l	42.202	4.47E-125	413	97
KAB2962965.1_MAG:_acyl-CoA_dehydrogenase_[Thermoanaerobaculia_bacterium]	ptg005087l	48.333	0	592	99
KAB2962964.1_MAG:_3-hydroxyacyl-CoA_dehydrogenase/enoyl-CoA_hydratase_family_protein_[Thermoanaerobaculia_bacterium]	ptg002612l	29.924	2.2E-20	101	50
KAB2962963.1_MAG:_thiolase_family_protein_[Thermoanaerobaculia_bacterium]	ptg005527l	44.961	1.85E-86	289	99
KAB2962961.1_MAG:_TRAP_transporter_large_permease_subunit_[Thermoanaerobaculia_bacterium]	ptg005195l	32.281	8.32E-42	154	67
KAB2962942.1_MAG:_ATP-binding_cassette_domain-containing_protein_[Thermoanaerobaculia_bacterium]	ptg003163l	34.586	4.38E-08	56.2	62
KAB2962940.1_MAG:_cytochrome_C_biogenesis_protein_[Thermoanaerobaculia_bacterium]	ptg005087l	29.545	7.17E-12	67.8	78
KAB2962937.1_MAG:_hypothetical_protein_F9K16_07815_[Thermoanaerobaculia_bacterium]	ptg001025l	39.524	3.98E-41	105	70
KAB2962936.1_MAG:_metallophosphoesterase_family_protein_[Thermoanaerobaculia_bacterium]	ptg005702l	31.25	3.13E-23	67.4	98
KAB2962935.1_MAG:_shikimate_kinase_[Thermoanaerobaculia_bacterium]	ptg004564l	39.634	8.32E-23	98.6	83
KAB2962934.1_MAG:_acetyl-CoA_carboxylase_biotin_carboxylase_subunit_[Thermoanaerobaculia_bacterium]	ptg004240l	54.628	7.35E-152	481	98
KAB2962933.1_MAG:_acetyl-CoA_carboxylase_biotin_carboxyl_carrier_protein_[Thermoanaerobaculia_bacterium]	ptg004240l	47.297	1.31E-12	67.8	46

KAB2962931.1_MAG:_50S_ribosomal_protein_L20_[Thermoanaerobaculia_bacterium]	ptg001925l	51.695	4.15E-30	116	91
KAB2962929.1_MAG:_translation_initiation_factor_IF-3_[Thermoanaerobaculia_bacterium]	ptg001558l	50.641	6.18E-40	147	82
KAB2962928.1_MAG:_threonine--tRNA_ligase_[Thermoanaerobaculia_bacterium]	ptg004758l	46.591	1.25E-168	538	95
KAB2962924.1_MAG:_AarF/ABC1/UbiB_kinase_family_protein_[Thermoanaerobaculia_bacterium]	ptg003583l	28.345	2.05E-47	182	75
KAB2962923.1_MAG:_hydroxymethylglutaryl-CoA_reductase_[Thermoanaerobaculia_bacterium]	ptg001917l	39.423	1.99E-86	295	79
KAB2962922.1_MAG:_adenylosuccinate_synthase_[Thermoanaerobaculia_bacterium]	ptg003090l	44.893	9.28E-98	325	93
KAB2962912.1_MAG:_oxygen-independent_coproporphyrinogen_III_oxidase_[Thermoanaerobaculia_bacterium]	ptg003548l	39.64	1.75E-107	353	95
KAB2962911.1_MAG:_uroporphyrinogen_decarboxylase_[Thermoanaerobaculia_bacterium]	ptg005545l	39.823	2.07E-67	233	98
KAB2962910.1_MAG:_porphobilinogen_synthase_[Thermoanaerobaculia_bacterium]	ptg004997l	51.662	7.95E-94	308	98
KAB2962908.1_MAG:_FHA_domain-containing_protein_[Thermoanaerobaculia_bacterium]	ptg003137l	50	1.97E-20	97.8	30
KAB2962907.1_MAG:_protein_kinase_[Thermoanaerobaculia_bacterium]	ptg004986l	32.192	2.26E-39	159	47
KAB2962903.1_MAG:_thioredoxin_family_protein_[Thermoanaerobaculia_bacterium]	LG15	37.097	1.48E-08	57	33
KAB2962902.1_MAG:_amidohydrolase_family_protein,_partial_[Thermoanaerobaculia_bacterium]	ptg004680l	31.356	0.000000121	57.4	34
KAB2962901.1_MAG:_Fe-S-binding_domain-containing_protein_[Thermoanaerobaculia_bacterium]	ptg004306l	33.219	1.39E-45	167	97
KAB2962900.1_MAG:_NADH-quinone_oxidoreductase_subunit_E,_partial_[Thermoanaerobaculia_bacterium]	ptg005451l	39.402	2.09E-59	215	74
KAB2962897.1_MAG:_phosphoribosylformylglycinamidine_synthase_subunit_PurL_[Thermoanaerobaculia_bacterium]	ptg005375l	31.274	2.42E-88	308	98
KAB2962895.1_MAG:_phosphoribosylformylglycinamidine_synthase_subunit_PurQ_[Thermoanaerobaculia_bacterium]	ptg005375l	31.048	3.49E-21	95.5	92
KAB2962892.1_MAG:_amidophosphoribosyltransferase_[Thermoanaerobaculia_bacterium]	ptg004564l	37.901	4.69E-80	275	95
KAB2962891.1_MAG:_3-methyl-2-oxobutanoate_dehydrogenase_subunit_VorB_[Thermoanaerobaculia_bacterium]	ptg002867l	25.714	1.38E-16	87	61
KAB2962890.1_MAG:_thiamine_pyrophosphate-binding_protein_[Thermoanaerobaculia_bacterium]	ptg004077l	35.135	3.79E-09	63.5	31
KAB2962889.1_MAG:_response_regulator_transcription_factor_[Thermoanaerobaculia_bacterium]	ptg003863l	33.714	2.36E-23	102	73
KAB2962887.1_MAG:_hypothetical_protein_F9K18_09250_[Thermoanaerobaculia_bacterium]	ptg005922l	39.07	6.9E-30	127	46
KAB2962873.1_MAG:_M1_family_metallopeptidase,_partial_[Thermoanaerobaculia_bacterium]	ptg002465l	31.692	2.79E-70	249	79
KAB2962871.1_MAG:_HD_domain-containing_protein_[Thermoanaerobaculia_bacterium]	ptg002928l	41.004	9.75E-42	157	79
KAB2962869.1_MAG:_class_IV_adenylate_cyclase_[Thermoanaerobaculia_bacterium]	ptg004277l	30.065	0.000000651	52.4	70
KAB2962868.1_MAG:_dipeptidase_[Thermoanaerobaculia_bacterium]	ptg005697l	47.669	1.46E-128	414	98
KAB2962860.1_MAG:_ABC_transporter_permease_[Thermoanaerobaculia_bacterium]	ptg003336l	44.954	7.95E-49	175	94
KAB2962858.1_MAG:_ABC_transporter_ATP-binding_protein_[Thermoanaerobaculia_bacterium]	ptg003336l	42.857	7.76E-45	164	78
KAB2962851.1_MAG:_aspartate_ammonia-lyase,_partial_[Thermoanaerobaculia_bacterium]	ptg001693l	50.661	1.53E-55	194	97
KAB2962850.1_MAG:_YebC/PmpR_family_DNA-binding_transcriptional_regulator_[Thermoanaerobaculia_bacterium]	ptg001690l	37.931	5.22E-32	127	96
KAB2962846.1_MAG:_GGDEF_domain-containing_protein_[Thermoanaerobaculia_bacterium]	ptg002030l	42.775	5.01E-28	120	43
KAB2962845.1_MAG:_carbonate_dehydratase_[Thermoanaerobaculia_bacterium]	ptg003910l	33.161	2.69E-23	100	87
KAB2962837.1_MAG:_sigma-70_family_RNA_polymerase_sigma_factor_[Thermoanaerobaculia_bacterium]	ptg003142l	41.53	2.91E-17	82.8	94

KAB2962836.1_MAG:_serine/threonine_protein_kinase,_partial_[Thermoanaerobaculia_bacterium]	ptg004979l	45.413	1.53E-44	166	67
KAB2962831.1_MAG:_aldehyde_dehydrogenase_family_protein_[Thermoanaerobaculia_bacterium]	ptg005596l	67.917	0	659	97
KAB2962830.1_MAG:_ABC-F_family_ATP-binding_cassette_domain-containing_protein,_partial_[Thermoanaerobaculia_bacterium]	ptg003204l	31.452	4.2E-61	221	96
KAB2962829.1_MAG:_dicarboxylate/amino_acid:cation_symporter_[Thermoanaerobaculia_bacterium]	ptg001170l	44.313	1.09E-69	243	95
KAB2962828.1_MAG:_aldehyde_dehydrogenase_[Thermoanaerobaculia_bacterium]	ptg005596l	51.399	5.29E-76	255	99
KAB2962823.1_MAG:_GIY-YIG_nuclease_family_protein_[Thermoanaerobaculia_bacterium]	ptg001995l	40.789	1.27E-08	53.1	90
KAB2962819.1_MAG:_hypothetical_protein_F9K18_09330_[Thermoanaerobaculia_bacterium]	ptg004575l	33.113	4.04E-21	92.8	87
KAB2962812.1_MAG:_SDR_family_oxidoreductase_[Thermoanaerobaculia_bacterium]	ptg005905l	31.507	4.51E-10	63.5	57
KAB2962811.1_MAG:_TonB-dependent_receptor_plug_domain-containing_protein,_partial_[Thermoanaerobaculia_bacterium]	ptg001473l	25.877	3.91E-08	58.2	76
KAB2962808.1_MAG:_hypothetical_protein_F9K16_08180_[Thermoanaerobaculia_bacterium]	ptg002767l	48.039	9.27E-51	179	99
KAB2962807.1_MAG:_nucleotidyl_transferase_AbiEii/AbiGii_toxin_family_protein_[Thermoanaerobaculia_bacterium]	ptg002767l	34.412	6.74E-39	150	97
KAB2962806.1_MAG:_TonB-dependent_receptor_[Thermoanaerobaculia_bacterium]	ptg004982l	28.472	0.000000334	58.5	16
KAB2962805.1_MAG:_GGDEF_domain-containing_protein,_partial_[Thermoanaerobaculia_bacterium]	LG04	40.206	2.51E-15	81.3	28
KAB2962804.1_MAG:_excinuclease_ABC_subunit_UvrB_[Thermoanaerobaculia_bacterium]	ptg004926l	58.333	0	736	98
KAB2962803.1_MAG:_excinuclease_ABC_subunit_UvrC,_partial_[Thermoanaerobaculia_bacterium]	ptg005527l	33.396	1.91E-65	241	68
KAB2962802.1_MAG:_3-deoxy-8-phosphooctulonate_synthase,_partial_[Thermoanaerobaculia_bacterium]	ptg003247l	53.521	1.95E-66	225	93
KAB2962801.1_MAG:_3-deoxy-manno-octulosonate_cytidylyltransferase_[Thermoanaerobaculia_bacterium]	ptg005527l	40.437	2.4E-34	124	76
KAB2962800.1_MAG:_CTP_synthase_[Thermoanaerobaculia_bacterium]	ptg004256l	56.434	0	637	97
KAB2962799.1_MAG:_polyprenyl_synthetase_family_protein_[Thermoanaerobaculia_bacterium]	ptg002505l	39.394	9.49E-33	132	67
KAB2962797.1_MAG:_YajQ_family_cyclic_di-GMP-binding_protein_[Thermoanaerobaculia_bacterium]	ptg000372l	41.509	3.46E-38	141	98
KAB2962795.1_MAG:_bifunctional_phosphoribosylaminoimidazolecarboxamide_formyltransferase/IMP_cyclohydrolase_[Thermoanaerobaculia_bacterium]	ptg004409l	48.069	4.45E-143	458	99
KAB2962786.1_MAG:_2-oxoacid:acceptor_oxidoreductase_subunit_alpha_[Thermoanaerobaculia_bacterium]	ptg002867l	32.701	8.34E-84	291	95
KAB2962785.1_MAG:_2-oxoglutarate_oxidoreductase_[Thermoanaerobaculia_bacterium]	ptg002102l	45.07	5.4E-59	207	66
KAB2962781.1_MAG:_NHLP_family_bacteriocin_export_ABC_transporter_peptidase/permease/ATPase_subunit_[Thermoanaerobaculia_bacterium]	ptg004210l	29.094	2.22E-81	288	90
KAB2962780.1_MAG:_NHLP_bacteriocin_export_ABC_transporter_permease/ATPase_subunit_[Thermoanaerobaculia_bacterium]	ptg004210l	25.565	9.09E-45	181	51
KAB2962777.1_MAG:_FHA_domain-containing_protein,_partial_[Thermoanaerobaculia_bacterium]	ptg001658l	44.286	2.39E-11	63.2	54
KAB2962768.1_MAG:_serine/threonine_protein_kinase,_partial_[Thermoanaerobaculia_bacterium]	ptg003082l	37.748	4.98E-44	164	91
KAB2962767.1_MAG:_hypothetical_protein_F9K16_08305,_partial_[Thermoanaerobaculia_bacterium]	ptg005527l	31.751	2.05E-40	157	83
KAB2962765.1_MAG:_SpoIIE_family_protein_phosphatase_[Thermoanaerobaculia_bacterium]	ptg004476l	27.306	1.02E-35	147	89
KAB2962763.1_MAG:_tetratricopeptide_repeat_protein_[Thermoanaerobaculia_bacterium]	ptg001897l	39.167	3.23E-24	114	26

KAB2962762.1_MAG:_STAS_domain-containing_protein_[Thermoanaerobaculia_bacterium]	ptg002742l	29.63	0.000000246	52.8	49
KAB2962755.1_MAG:_branched-chain_amino_acid_transaminase_[Thermoanaerobaculia_bacterium]	ptg005886l	51.149	3.15E-79	183	98
KAB2962753.1_MAG:_type_IV_pilus_twitching_motility_protein_PilT_[Thermoanaerobaculia_bacterium]	LG13	46.286	3.8E-79	269	89
KAB2962752.1_MAG:_RNA_methyltransferase,_partial_[Thermoanaerobaculia_bacterium]	ptg002679l	46	0.00000221	47	58
KAB2962747.1_MAG:_PqqD_family_protein_[Thermoanaerobaculia_bacterium]	ptg005190l	34.568	1.82E-08	56.6	46
KAB2962744.1_MAG:_radical_SAM_protein_[Thermoanaerobaculia_bacterium]	ptg004147l	37.363	0.000000231	56.6	24
KAB2962743.1_MAG:_methionine_synthase_[Thermoanaerobaculia_bacterium]	ptg002021l	34.171	4.48E-178	585	98
KAB2962742.1_MAG:_methylenetetrahydrofolate_reductase_[NAD(P)H]_[Thermoanaerobaculia_bacterium]	ptg004372l	30.263	1.88E-28	119	94
KAB2962740.1_MAG:_AAA_family_ATPase_[Thermoanaerobaculia_bacterium]	LG26	56	0.000000507	56.6	10
KAB2962738.1_MAG:_superoxide_dismutase_[Thermoanaerobaculia_bacterium]	ptg001604l	54.211	6.09E-55	192	80
KAB2962737.1_MAG:_formylglycine-generating_enzyme_family_protein_[Thermoanaerobaculia_bacterium]	ptg001897l	32.5	4.73E-10	56.2	51
KAB2962736.1_MAG:_hypothetical_protein_F9K16_08370_[Thermoanaerobaculia_bacterium]	ptg003703l	31.066	1.05E-39	161	78
KAB2962732.1_MAG:_hypothetical_protein_F9K16_08350_[Thermoanaerobaculia_bacterium]	ptg005333l	33.229	4.18E-52	148	51
KAB2962731.1_MAG:_HAD-IA_family_hydrolase_[Thermoanaerobaculia_bacterium]	ptg005554l	40.65	2.22E-27	99.8	82
KAB2962725.1_MAG:_S9_family_peptidase_[Thermoanaerobaculia_bacterium]	ptg003431l	39.255	1.23E-141	463	95
KAB2962724.1_MAG:_putative_Fe-S_cluster_assembly_protein_SufT_[Thermoanaerobaculia_bacterium]	ptg005265l	41.379	2.21E-32	128	72
KAB2962717.1_MAG:_WYL_domain-containing_protein,_partial_[Thermoanaerobaculia_bacterium]	ptg002031l	24.444	0.000000121	55.8	72
KAB2962716.1_MAG:_TlpA_family_protein_disulfide_reductase_[Thermoanaerobaculia_bacterium]	ptg001266l	31.579	1.27E-10	69.3	16
KAB2962715.1_MAG:_MATE_family_efflux_transporter_[Thermoanaerobaculia_bacterium]	ptg002992l	25.822	3.23E-16	85.5	93
KAB2962710.1_MAG:_rRNA_pseudouridine_synthase,_partial_[Thermoanaerobaculia_bacterium]	ptg004652l	41.25	1.05E-49	179	89
KAB2962709.1_MAG:_SMC-Scp_complex_subunit_ScpB_[Thermoanaerobaculia_bacterium]	ptg004457l	41.176	1.44E-18	88.6	40
KAB2962708.1_MAG:_segregation/condensation_protein_A_[Thermoanaerobaculia_bacterium]	ptg005938l	29.06	7.64E-14	74.7	85
KAB2962707.1_MAG:_tryptophan--tRNA_ligase_[Thermoanaerobaculia_bacterium]	ptg003327l	38.849	1.48E-52	190	81
KAB2962698.1_MAG:_hypothetical_protein_F9K16_08430,_partial_[Thermoanaerobaculia_bacterium]	ptg005063l	26.316	3.33E-11	73.2	16
KAB2962696.1_MAG:_PilT/PilU_family_type_4a_pilus_ATPase_[Thermoanaerobaculia_bacterium]	ptg004468l	44.253	2.9E-90	304	74
KAB2962695.1_MAG:_transcriptional_repressor_LexA_[Thermoanaerobaculia_bacterium]	ptg002700l	39.512	9.75E-34	130	98
KAB2962694.1_MAG:_insulinase_family_protein_[Thermoanaerobaculia_bacterium]	ptg005195l	50.201	3.95E-76	261	59
KAB2962689.1_MAG:_GDP-mannose_4,6-dehydratase_[Thermoanaerobaculia_bacterium]	ptg006001l	36.792	3.53E-47	174	96
KAB2962688.1_MAG:_NAD-dependent_epimerase/dehydratase_family_protein,_partial_[Thermoanaerobaculia_bacterium]	ptg002634l	34.247	0.000000598	52	77
KAB2962687.1_MAG:_dTDP-4-dehydrorhamnose_reductase_[Thermoanaerobaculia_bacterium]	ptg003827l	34.155	9.67E-39	148	96
KAB2962685.1_MAG:_acetyl-CoA_C-acyltransferase_[Thermoanaerobaculia_bacterium]	ptg004745l	45.753	2.65E-71	246	93
KAB2962684.1_MAG:_Holliday_junction_resolvase_RuvX_[Thermoanaerobaculia_bacterium]	ptg002760l	36.923	2.48E-17	80.9	91

KAB2962682.1_MAG:_protein_kinase,_partial_[Thermoanaerobaculia_bacterium]	ptg001897l	27.925	1.06E-10	68.6	50
KAB2962681.1_MAG:_hypothetical_protein_F9K18_09495_[Thermoanaerobaculia_bacterium]	ptg002723l	28.549	1.89E-52	198	99
KAB2962677.1_MAG:_biosynthetic_arginine_decarboxylase_[Thermoanaerobaculia_bacterium]	ptg005430l	26.132	0.00000036	57.8	43
KAB2962672.1_MAG:_ABC_transporter_ATP-binding_protein_[Thermoanaerobaculia_bacterium]	ptg004026l	37.57	1.4E-74	264	79
KAB2962664.1_MAG:_thioredoxin_domain-containing_protein,_partial_[Thermoanaerobaculia_bacterium]	ptg001290l	45.567	8.71E-109	354	99
KAB2962663.1_MAG:_Fe-S_cluster_assembly_ATPase_SufC_[Thermoanaerobaculia_bacterium]	ptg005265l	65.863	3.67E-108	346	98
KAB2962662.1_MAG:_SUF_system_Fe-S_cluster_assembly_regulator_[Thermoanaerobaculia_bacterium]	ptg005518l	29.231	4.64E-08	54.7	83
KAB2962659.1_MAG:_cysteine_desulfurase_[Thermoanaerobaculia_bacterium]	ptg005265l	44.961	5.98E-104	342	91
KAB2962658.1_MAG:_Fe-S_cluster_assembly_protein_SufD_[Thermoanaerobaculia_bacterium]	ptg005265l	34.646	8.49E-67	235	84
KAB2962657.1_MAG:_Fe-S_cluster_assembly_protein_SufB_[Thermoanaerobaculia_bacterium]	ptg005265l	74.423	0	713	99
KAB2962656.1_MAG:_PAS_domain_S-box_protein,_partial_[Thermoanaerobaculia_bacterium]	ptg005484l	30.851	2.35E-27	122	57
KAB2962651.1_MAG:_type_I_DNA_topoisomerase_[Thermoanaerobaculia_bacterium]	LG04	43.034	1.12E-147	482	81
KAB2962650.1_MAG:_NTP_transferase_domain-containing_protein,_partial_[Thermoanaerobaculia_bacterium]	ptg003813l	48.864	2.23E-15	73.9	75
KAB2962643.1_MAG:_hypothetical_protein_F9K18_09550,_partial_[Thermoanaerobaculia_bacterium]	ptg001995l	32.09	1.66E-08	59.3	48
KAB2962636.1_MAG:_3-deoxy-7-phosphoheptulonate_synthase_class_II,_partial_[Thermoanaerobaculia_bacterium]	ptg003327l	58.257	3.25E-140	448	93
KAB2962633.1_MAG:_methylmalonyl-CoA_carboxyltransferase_[Thermoanaerobaculia_bacterium]	ptg003168l	63.279	0	572	84
KAB2962632.1_MAG:_sulfatase_[Thermoanaerobaculia_bacterium]	ptg006050l	23.227	1E-15	84	83
KAB2962631.1_MAG:_sulfatase_[Thermoanaerobaculia_bacterium]	ptg002760l	25.814	1.05E-17	90.1	82
KAB2962629.1_MAG:_ribose_5-phosphate_isomerase_B_[Thermoanaerobaculia_bacterium]	ptg005354l	45.07	5.25E-27	108	94
KAB2962628.1_MAG:_serine_hydroxymethyltransferase_[Thermoanaerobaculia_bacterium]	ptg002443l	59.459	2.62E-142	452	88
KAB2962627.1_MAG:_hypothetical_protein_F9K18_09565,_partial_[Thermoanaerobaculia_bacterium]	ptg005010l	33.894	2.15E-37	152	66
KAB2962625.1_MAG:_replication-associated_recombination_protein_A,_partial_[Thermoanaerobaculia_bacterium]	ptg001690l	45.61	1.32E-99	330	94
KAB2962623.1_MAG:_molecular_chaperone_DnaK_[Thermoanaerobaculia_bacterium]	ptg005922l	62.706	0	730	94
KAB2962620.1_MAG:_hypothetical_protein_F9K16_08630_[Thermoanaerobaculia_bacterium]	ptg005839l	34.483	7.81E-08	60.5	13
KAB2962614.1_MAG:_3-hydroxyacyl-CoA_dehydrogenase_[Thermoanaerobaculia_bacterium]	ptg002612l	42.347	3.61E-48	175	67
KAB2962613.1_MAG:_NAD(P)/FAD-dependent_oxidoreductase_[Thermoanaerobaculia_bacterium]	ptg002658l	26.829	2.17E-17	90.1	61
KAB2962612.1_MAG:_long-chain_fatty_acid--CoA_ligase,_partial_[Thermoanaerobaculia_bacterium]	ptg005087l	25.333	1.43E-10	66.2	86
KAB2962611.1_MAG:_glycerol-3-phosphate_1-O-acyltransferase_PlsY_[Thermoanaerobaculia_bacterium]	LG04	52.128	1.55E-08	57.8	46
KAB2962608.1_MAG:_glutamine-hydrolyzing_GMP_synthase_[Thermoanaerobaculia_bacterium]	ptg002345l	56.275	0	598	99
KAB2962607.1_MAG:_NAD(P)-dependent_glycerol-3-phosphate_dehydrogenase_[Thermoanaerobaculia_bacterium]	ptg001005l	43.567	4.09E-63	220	99
KAB2962606.1_MAG:_competence/damage-inducible_protein_A_[Thermoanaerobaculia_bacterium]	ptg002699l	37.589	3.49E-21	100	31

KAB2962599.1_MAG:_dTDP-glucose_4,6-dehydratase_[Thermoanaerobaculia_bacterium]	ptg001690l	40	4.81E-81	273	97
KAB2962598.1_MAG:_transketolase,_partial_[Thermoanaerobaculia_bacterium]	ptg004010l	25.292	0.000000125	57.8	68
KAB2962593.1_MAG:_response_regulator_transcription_factor_[Thermoanaerobaculia_bacterium]	ptg003863l	29.808	2.79E-21	95.1	96
KAB2962591.1_MAG:_hypothetical_protein_F9K16_08680_[Thermoanaerobaculia_bacterium]	LG07	35.821	7.91E-29	129	52
KAB2962590.1_MAG:_NADH-quinone_oxidoreductase_subunit_E,_partial_[Thermoanaerobaculia_bacterium]	ptg005451l	40.052	3.27E-68	239	87
KAB2962589.1_MAG:_integration_host_factor_subunit_beta_[Thermoanaerobaculia_bacterium]	ptg005933l	42.169	8.79E-15	70.9	94
KAB2962588.1_MAG:_site-2_protease_family_protein_[Thermoanaerobaculia_bacterium]	ptg001245l	32.51	7.82E-19	89.7	80
KAB2962587.1_MAG:_D-tyrosyl-tRNA(Tyr)_deacylase_[Thermoanaerobaculia_bacterium]	ptg002175l	49.306	6.75E-28	111	95
KAB2962586.1_MAG:_single-stranded_DNA-binding_protein_[Thermoanaerobaculia_bacterium]	ptg005756l	40.777	3.84E-20	89	73
KAB2962585.1_MAG:_TetR/AcrR_family_transcriptional_regulator_[Thermoanaerobaculia_bacterium]	ptg002199l	29.464	0.00000374	50.8	53
KAB2962576.1_MAG:_lytic_transglycosylase_domain-containing_protein_[Thermoanaerobaculia_bacterium]	ptg004256l	30.464	0.00000637	50.4	53
KAB2962574.1_MAG:_phosphotransferase_[Thermoanaerobaculia_bacterium]	ptg004480l	30.325	1.22E-10	67	70
KAB2962572.1_MAG:_23S_rRNA_(adenine(2503)-C(2))-methyltransferase_RlmN_[Thermoanaerobaculia_bacterium]	ptg002634l	40.16	5.17E-69	238	95
KAB2962570.1_MAG:_polyphenol_oxidase_family_protein,_partial_[Thermoanaerobaculia_bacterium]	ptg005689l	37.241	0.00000996	48.5	75
KAB2962569.1_MAG:_DEAD/DEAH_box_helicase,_partial_[Thermoanaerobaculia_bacterium]	ptg002773l	28.108	9.78E-08	60.8	15
KAB2962559.1_MAG:_TlpA_family_protein_disulfide_reductase,_partial_[Thermoanaerobaculia_bacterium]	ptg002992l	29.577	0.000000335	56.2	35
KAB2962557.1_MAG:_6-oxocyclohex-1-ene-1-carbonyl-CoA_hydratase_[Thermoanaerobaculia_bacterium]	ptg002612l	24.37	3.45E-12	72	58
KAB2962551.1_MAG:_Rrf2_family_transcriptional_regulator_[Thermoanaerobaculia_bacterium]	ptg005265l	28.906	9.12E-12	66.6	65
KAB2962550.1_MAG:_M48_family_metalloprotease,_partial_[Thermoanaerobaculia_bacterium]	ptg002738l	38.75	7.02E-08	58.5	22
KAB2962544.1_MAG:_serine/threonine_protein_kinase,_partial_[Thermoanaerobaculia_bacterium]	ptg004979l	45.714	1.38E-40	154	67
KAB2962543.1_MAG:_sigma-70_family_RNA_polymerase_sigma_factor_[Thermoanaerobaculia_bacterium]	ptg004340l	43.094	1.13E-31	123	98
KAB2962534.1_MAG:_replication-associated_recombination_protein_A_[Thermoanaerobaculia_bacterium]	ptg001690l	44.665	4.3E-91	305	93
KAB2962530.1_MAG:_DUF255_domain-containing_protein,_partial_[Thermoanaerobaculia_bacterium]	ptg002021l	38.393	5.32E-19	85.5	75
KAB2962524.1_MAG:_sodium-translocating_pyrophosphatase_[Thermoanaerobaculia_bacterium]	ptg003304l	70.12	0	892	99
KAB2962523.1_MAG:_dehydrogenase_[Thermoanaerobaculia_bacterium]	ptg002612l	33.333	1.04E-41	168	86
KAB2962522.1_MAG:_dCMP_deaminase_family_protein_[Thermoanaerobaculia_bacterium]	ptg005856l	30.168	4.06E-14	72	85
KAB2962521.1_MAG:_sodium:calcium_symporter_[Thermoanaerobaculia_bacterium]	ptg003960l	30.918	7.67E-37	150	75
KAB2962519.1_MAG:_long-chain_fatty_acid--CoA_ligase,_partial_[Thermoanaerobaculia_bacterium]	ptg002844l	28.906	5.88E-11	65.9	47
KAB2962517.1_MAG:_fumarate_hydratase_[Thermoanaerobaculia_bacterium]	LG24	68.182	2.6E-20	99.4	69
KAB2962513.1_MAG:_1-acyl-sn-glycerol-3-phosphate_acyltransferase_[Thermoanaerobaculia_bacterium]	ptg002123l	34.161	1.06E-12	73.6	43
KAB2962509.1_MAG:_AmmeMemoRadiSam_system_protein_B_[Thermoanaerobaculia_bacterium]	LG16	34.715	1.07E-08	59.3	69
KAB2962508.1_MAG:_ABC_transporter_permease,_partial_[Thermoanaerobaculia_bacterium]	ptg004026l	43.554	4.6E-51	184	96
KAB2962504.1_MAG:_adenylosuccinate_synthase,_partial_[Thermoanaerobaculia_bacterium]	ptg003090l	50	2.6E-72	244	86

KAB2962494.1_MAG:_hypothetical_protein_F9K16_08890_[Thermoanaerobaculia_bacterium]	ptg005354l	29.596	5.28E-16	83.6	59
KAB2962488.1_MAG:_MBL_fold_metallo-hydrolase_[Thermoanaerobaculia_bacterium]	ptg002475l	41.722	5.1E-89	300	97
KAB2962481.1_MAG:_AAA_family_ATPase,_partial_[Thermoanaerobaculia_bacterium]	ptg004306l	42.991	7.79E-69	236	98
KAB2962478.1_MAG:_aspartate_carbamoyltransferase_catalytic_subunit_[Thermoanaerobaculia_bacterium]	ptg005190l	53.107	3.22E-72	182	85
KAB2962477.1_MAG:_dihydroorotase_[Thermoanaerobaculia_bacterium]	LG04	37.299	6.49E-46	160	96
KAB2962476.1_MAG:_inositol_monophosphatase_[Thermoanaerobaculia_bacterium]	ptg005265l	51.244	5.7E-40	152	65
KAB2962475.1_MAG:_M23_family_metallopeptidase_[Thermoanaerobaculia_bacterium]	ptg000819l	38.384	1.31E-08	60.5	28
KAB2962471.1_MAG:_asparagine--tRNA_ligase_[Thermoanaerobaculia_bacterium]	ptg002928l	36.264	4.41E-83	282	97
KAB2962467.1_MAG:_sigma-54-dependent_Fis_family_transcriptional_regulator_[Thermoanaerobaculia_bacterium]	ptg005150l	45.269	3.84E-105	347	85
KAB2962466.1_MAG:_PAS_domain_S-box_protein_[Thermoanaerobaculia_bacterium]	ptg002304l	25.07	1.51E-21	103	61
KAB2962465.1_MAG:_type_II_secretion_system_F_family_protein_[Thermoanaerobaculia_bacterium]	ptg005301l	39.348	6.51E-87	291	99
KAB2962464.1_MAG:_type_IV_pilus_twitching_motility_protein_PilT_[Thermoanaerobaculia_bacterium]	ptg000568l	49.284	5.03E-101	331	93
KAB2962463.1_MAG:_ABC_transporter_ATP-binding_protein_[Thermoanaerobaculia_bacterium]	ptg004026l	33.333	8.4E-70	250	78
KAB2962460.1_MAG:_diguanylate_cyclase_[Thermoanaerobaculia_bacterium]	LG04	43.396	5.7E-17	87.8	24
KAB2962458.1_MAG:_Crp/Fnr_family_transcriptional_regulator_[Thermoanaerobaculia_bacterium]	ptg002907l	24.365	5.12E-18	86.3	85
KAB2962455.1_MAG:_MMPL_family_transporter_[Thermoanaerobaculia_bacterium]	ptg003364l	35.088	1.14E-10	65.1	43
KAB2962451.1_MAG:_ABC_transporter_ATP-binding_protein_[Thermoanaerobaculia_bacterium]	ptg001690l	35.714	2.65E-47	174	95
KAB2962445.1_MAG:_M48_family_metalloprotease,_partial_[Thermoanaerobaculia_bacterium]	ptg005195l	40.58	0.000000797	52	35
KAB2962444.1_MAG:_YbjQ_family_protein_[Thermoanaerobaculia_bacterium]	ptg003204l	40.909	4.81E-25	103	92
KAB2962443.1_MAG:_YbjQ_family_protein_[Thermoanaerobaculia_bacterium]	ptg003204l	70.297	9.63E-29	111	97
KAB2962432.1_MAG:_NADH:flavin_oxidoreductase_[Thermoanaerobaculia_bacterium]	ptg005550l	56	1.62E-09	52	38
KAB2962431.1_MAG:_GntR_family_transcriptional_regulator_[Thermoanaerobaculia_bacterium]	ptg003831l	47.826	0.000000281	52	47
KAB2962427.1_MAG:_crossover_junction_endodeoxyribonuclease_RuvC_[Thermoanaerobaculia_bacterium]	ptg005341l	36.508	1.05E-14	74.3	76
KAB2962426.1_MAG:_MotA/TolQ/ExbB_proton_channel_family_protein_[Thermoanaerobaculia_bacterium]	ptg004266l	38.542	7.53E-35	134	83
KAB2962424.1_MAG:_biopolymer_transporter_ExbD_[Thermoanaerobaculia_bacterium]	ptg004266l	29.508	0.00000921	47.8	78
KAB2962423.1_MAG:_energy_transducer_TonB_[Thermoanaerobaculia_bacterium]	ptg005754l	46.753	2.19E-11	67	32
KAB2962414.1_MAG:_AAA_domain-containing_protein,_partial_[Thermoanaerobaculia_bacterium]	ptg000568l	48.357	3.2E-55	194	87
KAB2962412.1_MAG:_glycosyltransferase_family_2_protein_[Thermoanaerobaculia_bacterium]	ptg003431l	52.966	2.22E-68	231	97
KAB2962410.1_MAG:_M20/M25/M40_family_metallo-hydrolase_[Thermoanaerobaculia_bacterium]	ptg002467l	29.518	0.000000174	58.9	25
KAB2962408.1_MAG:_UbiD_family_decarboxylase_[Thermoanaerobaculia_bacterium]	ptg005416l	35.859	1.23E-55	167	85
KAB2962407.1_MAG:_M23_family_metallopeptidase_[Thermoanaerobaculia_bacterium]	ptg004510l	38.298	2.73E-11	70.1	20
KAB2962401.1_MAG:_ParA_family_protein_[Thermoanaerobaculia_bacterium]	ptg001818l	51.19	3.31E-76	254	99
KAB2962400.1_MAG:_ParB/RepB/Spo0J_family_partition_protein_[Thermoanaerobaculia_bacterium]	ptg001818l	48.969	3.34E-43	160	64
KAB2962399.1_MAG:_prohibitin_family_protein_[Thermoanaerobaculia_bacterium]	ptg005920l	27.895	3.54E-14	76.6	64
KAB2962398.1_MAG:_TerC_family_protein_[Thermoanaerobaculia_bacterium]	ptg003226l	47.059	4.62E-36	139	84

KAB2962396.1_MAG:_tRNA_dihydrouridine_synthase_DusB_[Thermoanaerobaculia_bacterium]	ptg004321l	32.353	1.22E-40	155	87
KAB2962394.1_MAG:_ABC_transporter_permease_[Thermoanaerobaculia_bacterium]	ptg002610l	33.186	1.24E-21	102	50
KAB2962393.1_MAG:_ABC_transporter_ATP-binding_protein_[Thermoanaerobaculia_bacterium]	ptg004026l	36.093	2.8E-39	150	95
KAB2962392.1_MAG:_SpoIIE_family_protein_phosphatase,_partial_[Thermoanaerobaculia_bacterium]	ptg004476l	29.861	2.14E-08	60.8	31
KAB2962386.1_MAG:_YjbQ_family_protein_[Thermoanaerobaculia_bacterium]	ptg004959l	28.788	4.09E-09	57.4	88
KAB2962381.1_MAG:_response_regulator_[Thermoanaerobaculia_bacterium]	ptg001170l	43.231	4.33E-42	155	100
KAB2962379.1_MAG:_septum_formation_inhibitor_Maf_[Thermoanaerobaculia_bacterium]	ptg005709l	42.941	2.25E-20	91.7	85
KAB2962374.1_MAG:_aminotransferase_class_V-fold_PLP-dependent_enzyme,_partial_[Thermoanaerobaculia_bacterium]	ptg005756l	58.904	3.75E-16	75.1	82
KAB2962371.1_MAG:_hypothetical_protein_F9K18_09920,_partial_[Thermoanaerobaculia_bacterium]	ptg005545l	40.299	0.000000309	48.9	89
KAB2962370.1_MAG:_30S_ribosomal_protein_S12_methylthiotransferase_RimO_[Thermoanaerobaculia_bacterium]	ptg002871l	40.091	1.04E-69	244	94
KAB2962365.1_MAG:_AAA_domain-containing_protein_[Thermoanaerobaculia_bacterium]	ptg005527l	28.367	1.13E-24	109	87
KAB2962364.1_MAG:_ABC_transporter_ATP-binding_protein_[Thermoanaerobaculia_bacterium]	ptg004017l	45.54	2.5E-45	165	86
KAB2962361.1_MAG:_class_I_SAM-dependent_methyltransferase_[Thermoanaerobaculia_bacterium]	ptg005113l	28.571	0.00000234	54.3	23
KAB2962358.1_MAG:_TetR/AcrR_family_transcriptional_regulator_[Thermoanaerobaculia_bacterium]	ptg005647l	39.286	0.000000428	53.5	26
KAB2962356.1_MAG:_DNA_repair_protein_RecN,_partial_[Thermoanaerobaculia_bacterium]	ptg005922l	32.524	6.93E-19	89.4	81
KAB2962354.1_MAG:_GGDEF_domain-containing_protein_[Thermoanaerobaculia_bacterium]	ptg002052l	40.244	9.04E-48	176	73
KAB2962353.1_MAG:_type_1_glutamine_amidotransferase_[Thermoanaerobaculia_bacterium]	ptg005204l	38.261	1.52E-13	73.9	44
KAB2962349.1_MAG:_lipoyl_synthase_[Thermoanaerobaculia_bacterium]	ptg004680l	44.876	1.41E-68	235	89
KAB2962348.1_MAG:_hypothetical_protein_F9K16_09280,_partial_[Thermoanaerobaculia_bacterium]	ptg000619l	29.452	8.86E-24	107	69
KAB2962345.1_MAG:_nitroreductase_family_protein_[Thermoanaerobaculia_bacterium]	ptg003871l	56.085	6.7E-45	163	84
KAB2962343.1_MAG:_endoglucanase_[Thermoanaerobaculia_bacterium]	ptg005886l	32.234	2.8E-27	106	50
KAB2962337.1_MAG:_phosphate_ABC_transporter_permease_PstA_[Thermoanaerobaculia_bacterium]	ptg002567l	74.194	1.55E-99	333	84
KAB2962336.1_MAG:_phosphate_ABC_transporter_ATP-binding_protein_[Thermoanaerobaculia_bacterium]	ptg002567l	66.275	1.17E-112	360	92
KAB2962335.1_MAG:_ABC_transporter_permease_subunit,_partial_[Thermoanaerobaculia_bacterium]	ptg002567l	54.222	6.05E-64	209	98
KAB2962333.1_MAG:_cyclic_nucleotide-binding_domain-containing_protein_[Thermoanaerobaculia_bacterium]	ptg004457l	36.082	1.27E-08	62.4	16
KAB2962328.1_MAG:_pyridoxal_phosphate-dependent_aminotransferase,_partial_[Thermoanaerobaculia_bacterium]	ptg004905l	31.224	1.2E-17	86.7	84
KAB2962327.1_MAG:_NADH-quinone_oxidoreductase_subunit_I_[Thermoanaerobaculia_bacterium]	ptg004143l	29.94	1.18E-18	87.8	75
KAB2962326.1_MAG:_NADH-quinone_oxidoreductase_subunit_D_[Thermoanaerobaculia_bacterium]	ptg003419l	36.719	7.14E-83	286	68
KAB2962325.1_MAG:_NADH-quinone_oxidoreductase_subunit_B_[Thermoanaerobaculia_bacterium]	ptg004143l	48.322	2.49E-49	173	89
KAB2962324.1_MAG:_SpoIIE_family_protein_phosphatase_[Thermoanaerobaculia_bacterium]	ptg004573l	50.98	7.13E-09	52	13
KAB2962323.1_MAG:_site-specific_tyrosine_recombinase_XerD_[Thermoanaerobaculia_bacterium]	ptg004340l	41.077	1.19E-61	215	93
KAB2962317.1_MAG:_CocE/NonD_family_hydrolase,_partial_[Thermoanaerobaculia_bacterium]	ptg003785l	31.969	4.71E-47	177	88

KAB2962316.1_MAG:_3-oxo-5-alpha-steroid_4-dehydrogenase_[Thermoanaerobaculia_bacterium]	ptg002120l	29.508	1.16E-08	58.9	46
KAB2962315.1_MAG:_murein_transglycosylase,_partial_[Thermoanaerobaculia_bacterium]	ptg005398l	32.645	8.74E-60	149	87
KAB2962311.1_MAG:_ferrous_iron_transport_protein_B_[Thermoanaerobaculia_bacterium]	ptg003831l	40.805	5.91E-24	112	59
KAB2962307.1_MAG:_DNA-binding_protein_[Thermoanaerobaculia_bacterium]	ptg004010l	48.611	6.68E-12	70.5	21
KAB2962304.1_MAG:_amidohydrolase_family_protein,_partial_[Thermoanaerobaculia_bacterium]	ptg004242l	39.779	2.13E-23	102	74
KAB2962303.1_MAG:_serine/threonine_protein_kinase,_partial_[Thermoanaerobaculia_bacterium]	ptg004564l	39.695	7.78E-42	156	99
KAB2962302.1_MAG:_3-methyl-2-oxobutanoate_hydroxymethyltransferase_[Thermoanaerobaculia_bacterium]	ptg002760l	49.237	1.81E-70	239	92
KAB2962301.1_MAG:_pantoate--beta-alanine_ligase_[Thermoanaerobaculia_bacterium]	ptg002465l	43.728	7.1E-55	194	98
KAB2962300.1_MAG:_aspartate_1-decarboxylase_[Thermoanaerobaculia_bacterium]	ptg004018l	51.786	8.15E-31	119	79
KAB2962298.1_MAG:_5-(carboxyamino)imidazole_ribonucleotide_mutase_[Thermoanaerobaculia_bacterium]	ptg002871l	53.416	1.76E-38	142	98
KAB2962297.1_MAG:_response_regulator_[Thermoanaerobaculia_bacterium]	ptg002909l	35.844	1.8E-57	217	50
KAB2962296.1_MAG:_response_regulator_[Thermoanaerobaculia_bacterium]	LG04	41.818	2.77E-21	91.3	95
KAB2962295.1_MAG:_response_regulator_[Thermoanaerobaculia_bacterium]	ptg005035l	31.322	5.12E-43	171	88
KAB2962294.1_MAG:_DEAD/DEAH_box_helicase,_partial_[Thermoanaerobaculia_bacterium]	ptg003863l	50.535	3.01E-109	357	87
KAB2962290.1_MAG:_dCMP_deaminase_family_protein_[Thermoanaerobaculia_bacterium]	ptg005856l	28.655	8.48E-12	65.5	80
KAB2962288.1_MAG:_cupin_domain-containing_protein_[Thermoanaerobaculia_bacterium]	ptg004266l	50.42	6.37E-36	132	99
KAB2962287.1_MAG:_hypothetical_protein_F9K18_10015_[Thermoanaerobaculia_bacterium]	ptg003364l	28.653	3.06E-13	74.3	95
KAB2962284.1_MAG:_prolyl_oligopeptidase_family_serine_peptidase_[Thermoanaerobaculia_bacterium]	ptg002871l	30.137	1.68E-57	217	78
KAB2962283.1_MAG:_electron_transfer_flavoprotein-ubiquinone_oxidoreductase_[Thermoanaerobaculia_bacterium]	ptg005554l	36.652	2.16E-55	152	87
KAB2962282.1_MAG:_SRPBCC_family_protein_[Thermoanaerobaculia_bacterium]	ptg005048l	48.252	5.74E-37	137	99
KAB2962281.1_MAG:_fasciclin_domain-containing_protein_[Thermoanaerobaculia_bacterium]	ptg003903l	55.396	1.16E-45	162	85
KAB2962280.1_MAG:_helix-turn-helix_domain-containing_protein_[Thermoanaerobaculia_bacterium]	ptg005204l	32.231	1.33E-14	77.8	81
KAB2962274.1_MAG:_cyclic_nucleotide-binding_domain-containing_protein_[Thermoanaerobaculia_bacterium]	ptg004457l	37.113	8.16E-09	62.8	16
KAB2962272.1_MAG:_NADP_oxidoreductase,_partial_[Thermoanaerobaculia_bacterium]	LG02	45.714	0.000000003	61.6	34
KAB2962271.1_MAG:_glutaminyl-peptide_cyclotransferase_[Thermoanaerobaculia_bacterium]	ptg005053l	40.171	3.3E-36	139	91
KAB2962270.1_MAG:_RluA_family_pseudouridine_synthase_[Thermoanaerobaculia_bacterium]	ptg002304l	41.275	1.97E-45	169	88
KAB2962269.1_MAG:_TIGR00266_family_protein_[Thermoanaerobaculia_bacterium]	ptg003839l	57.769	1.21E-78	265	74
KAB2962268.1_MAG:_PDZ_domain-containing_protein_[Thermoanaerobaculia_bacterium]	ptg004479l	30.424	6.45E-25	115	85
KAB2962264.1_MAG:_holo-[acyl-carrier-protein]_synthase_[Thermoanaerobaculia_bacterium]	ptg003327l	37.736	1.01E-11	64.3	79
KAB2962261.1_MAG:_1-acyl-sn-glycerol-3-phosphate_acyltransferase_[Thermoanaerobaculia_bacterium]	ptg003708l	32.258	2.38E-15	80.5	48
KAB2962258.1_MAG:_di-trans,poly-cis-decaprenylcistransferase_[Thermoanaerobaculia_bacterium]	ptg005274l	47.303	7.17E-65	223	87
KAB2962257.1_MAG:_CDP-archaeol_synthase_[Thermoanaerobaculia_bacterium]	ptg002436l	40	1.27E-20	95.1	50
KAB2962256.1_MAG:_1-deoxy-D-xylulose-5-phosphate_reductoisomerase_[Thermoanaerobaculia_bacterium]	ptg003090l	53.333	1.46E-55	201	54

KAB2962255.1_MAG:_RIP_metalloprotease_RseP_[Thermoanaerobaculia_bacterium]	ptg002436l	32.057	9.75E-43	164	90
KAB2962252.1_MAG:_ribulose-phosphate_3-epimerase_[Thermoanaerobaculia_bacterium]	ptg004771l	46.948	1.59E-48	173	96
KAB2962246.1_MAG:_NAD(P)-binding_protein,_partial_[Thermoanaerobaculia_bacterium]	ptg002123l	35.463	5.95E-31	136	31
KAB2962244.1_MAG:_type_I_glyceraldehyde-3-phosphate_dehydrogenase_[Thermoanaerobaculia_bacterium]	ptg002223l	41.493	8.22E-67	231	99
KAB2962243.1_MAG:_tRNA_(adenosine(37)-N6)-threonylcarbamoyltransferase_complex_transferase_subunit_TsaD,_partial_[Thermoanaerobaculia_bacterium]	ptg005331l	38.176	4.91E-31	126	97
KAB2962239.1_MAG:_phosphoglycerate_kinase,_partial_[Thermoanaerobaculia_bacterium]	ptg003928l	46.277	2.61E-84	284	94
KAB2962238.1_MAG:_pantetheine-phosphate_adenylyltransferase_[Thermoanaerobaculia_bacterium]	ptg004885l	43.293	9.5E-40	145	98
KAB2962237.1_MAG:_adenosine_deaminase_[Thermoanaerobaculia_bacterium]	ptg005754l	30.435	7.82E-41	156	92
KAB2962230.1_MAG:_ATP-binding_protein,_partial_[Thermoanaerobaculia_bacterium]	ptg005732l	37.349	0.00000532	47.4	70
KAB2962226.1_MAG:_lytic_transglycosylase_domain-containing_protein_[Thermoanaerobaculia_bacterium]	ptg004256l	35.252	1.59E-12	68.2	68
KAB2962225.1_MAG:_SpoIIE_family_protein_phosphatase,_partial_[Thermoanaerobaculia_bacterium]	ptg004476l	30.876	1.47E-10	68.9	32
KAB2962224.1_MAG:_tyrosine_recombinase_XerD,_partial_[Thermoanaerobaculia_bacterium]	ptg004340l	49.573	8.29E-60	206	98
KAB2962216.1_MAG:_acyl-CoA_dehydrogenase_[Thermoanaerobaculia_bacterium]	ptg004179l	27.034	7.97E-27	117	91
KAB2962214.1_MAG:_redox-regulated_ATPase_YchF_[Thermoanaerobaculia_bacterium]	ptg004676l	55.616	1.25E-115	373	99
KAB2962212.1_MAG:_Rne/Rng_family_ribonuclease_[Thermoanaerobaculia_bacterium]	ptg005709l	41.434	4.47E-102	339	99
KAB2962211.1_MAG:_rod_shape-determining_protein_RodA_[Thermoanaerobaculia_bacterium]	ptg004510l	33.969	8.64E-22	100	73
KAB2962206.1_MAG:_hypothetical_protein_F9K16_09715_[Thermoanaerobaculia_bacterium]	ptg000819l	25.862	0.00000492	55.5	15
KAB2962202.1_MAG:_lytic_transglycosylase_domain-containing_protein,_partial_[Thermoanaerobaculia_bacterium]	ptg004256l	35.526	5.9E-16	79.7	71
KAB2962200.1_MAG:_YggS_family_pyridoxal_phosphate-dependent_enzyme_[Thermoanaerobaculia_bacterium]	ptg005677l	35.811	1.49E-22	99.4	63
KAB2962194.1_MAG:_alanine_dehydrogenase_[Thermoanaerobaculia_bacterium]	ptg005554l	42.424	3.07E-68	237	96
KAB2962193.1_MAG:_acetyl-CoA_hydrolase/transferase_family_protein_[Thermoanaerobaculia_bacterium]	LG22	65.909	4.22E-08	59.7	22
KAB2962192.1_MAG:_M3_family_metallopeptidase_[Thermoanaerobaculia_bacterium]	ptg001175l	38.746	7.46E-148	481	97
KAB2962185.1_MAG:_DUF3516_domain-containing_protein_[Thermoanaerobaculia_bacterium]	ptg006020l	33.256	6.27E-56	214	47
KAB2962183.1_MAG:_aminomethyl_transferase_family_protein_[Thermoanaerobaculia_bacterium]	ptg005299l	27.654	3.69E-27	117	88
KAB2962182.1_MAG:_NAD(P)/FAD-dependent_oxidoreductase_[Thermoanaerobaculia_bacterium]	ptg006031l	23.182	0.000000753	56.2	40
KAB2962175.1_MAG:_aminotransferase_class_I/II-fold_pyridoxal_phosphate-dependent_enzyme_[Thermoanaerobaculia_bacterium]	ptg001224l	35.784	4.97E-56	202	98
KAB2962174.1_MAG:_L-threonine_3-dehydrogenase_[Thermoanaerobaculia_bacterium]	ptg003072l	31.875	0.0000042	52.4	46
KAB2962172.1_MAG:_Re/Si-specific_NAD(P)(+)_transhydrogenase_subunit_alpha_[Thermoanaerobaculia_bacterium]	ptg004054l	45.479	6.41E-75	256	95
KAB2962160.1_MAG:_acyl-CoA_thioesterase_[Thermoanaerobaculia_bacterium]	ptg004075l	35.433	1.15E-18	84.3	95
KAB2962158.1_MAG:_sigma-70_family_RNA_polymerase_sigma_factor_[Thermoanaerobaculia_bacterium]	ptg003903l	31.548	1.09E-15	78.2	85
KAB2962153.1_MAG:_ABC_transporter_permease_[Thermoanaerobaculia_bacterium]	ptg004986l	27.107	5.27E-26	114	97

KAB2962152.1_MAG:_ABC_transporter_permease_[Thermoanaerobaculia_bacterium]	ptg004986l	25.918	1.13E-18	92.8	99
KAB2962151.1_MAG:_ABC_transporter_ATP-binding_protein_[Thermoanaerobaculia_bacterium]	ptg001659l	50.226	2.27E-68	231	88
KAB2962147.1_MAG:_Rieske_2Fe-2S_domain-containing_protein_[Thermoanaerobaculia_bacterium]	ptg005150l	32.353	0.00000176	53.9	28
KAB2962144.1_MAG:_UDP-N-acetylmuramoyl-L-alanyl-D-glutamate--2,6-diaminopimelate_ligase_[Thermoanaerobaculia_bacterium]	ptg002660l	31.277	5.27E-45	173	92
KAB2962143.1_MAG:_PASTA_domain-containing_protein_[Thermoanaerobaculia_bacterium]	ptg004827l	23.512	1.43E-09	65.5	43
KAB2962141.1_MAG:_16S_rRNA_(cytosine(1402)-N(4))-methyltransferase_RsmH_[Thermoanaerobaculia_bacterium]	ptg004266l	39.175	2.1E-28	119	94
KAB2962136.1_MAG:_hypothetical_protein_F9K18_10245_[Thermoanaerobaculia_bacterium]	LG19	33.036	0.00000617	54.3	12
KAB2962135.1_MAG:_polyribonucleotide_nucleotidyltransferase,_partial_[Thermoanaerobaculia_bacterium]	ptg004026l	47.807	0	598	92
KAB2962134.1_MAG:_30S_ribosomal_protein_S15_[Thermoanaerobaculia_bacterium]	ptg004026l	51.685	1.29E-24	99	100
KAB2962133.1_MAG:_tRNA_pseudouridine(55)_synthase_TruB_[Thermoanaerobaculia_bacterium]	ptg002420l	39.151	1.76E-33	134	68
KAB2962132.1_MAG:_bifunctional_oligoribonuclease/PAP_phosphatase_NrnA_[Thermoanaerobaculia_bacterium]	ptg004026l	31.868	2.32E-16	84	82
KAB2962131.1_MAG:_30S_ribosome-binding_factor_RbfA_[Thermoanaerobaculia_bacterium]	ptg003903l	36.538	5.41E-19	84.3	87
KAB2962129.1_MAG:_translation_initiation_factor_IF-2_[Thermoanaerobaculia_bacterium]	ptg001690l	44.26	5E-151	489	86
KAB2962123.1_MAG:_AAA_family_ATPase_[Thermoanaerobaculia_bacterium]	ptg004623l	47.308	1.53E-61	213	92
KAB2962115.1_MAG:_tetratricopeptide_repeat_protein,_partial_[Thermoanaerobaculia_bacterium]	ptg000619l	30.493	2.41E-17	89.4	45
KAB2962112.1_MAG:_cytochrome_C_oxidase_subunit_I_[Thermoanaerobaculia_bacterium]	ptg001690l	23.628	0.000000985	56.2	68
KAB2962111.1_MAG:_tetratricopeptide_repeat_protein,_partial_[Thermoanaerobaculia_bacterium]	ptg005121l	25	0.00000237	52	56
KAB2962110.1_MAG:_MBL_fold_metallo-hydrolase_[Thermoanaerobaculia_bacterium]	ptg004509l	35.019	6.49E-38	144	98
KAB2962109.1_MAG:_ferredoxin-NADP_reductase_[Thermoanaerobaculia_bacterium]	LG07	37.692	1.03E-15	81.3	42
KAB2962107.1_MAG:_electron_transport_complex_subunit_A_[Thermoanaerobaculia_bacterium]	ptg002214l	52	2.44E-35	99.8	96
KAB2962106.1_MAG:_RnfABCDGE_type_electron_transport_complex_subunit_E_[Thermoanaerobaculia_bacterium]	ptg002214l	47.287	5.2E-31	122	63
KAB2962104.1_MAG:_RnfABCDGE_type_electron_transport_complex_subunit_D_[Thermoanaerobaculia_bacterium]	ptg002214l	50	3.05E-09	62.4	20
KAB2962103.1_MAG:_electron_transport_complex_subunit_RsxC_[Thermoanaerobaculia_bacterium]	ptg002214l	36.343	2.59E-76	263	97
KAB2962099.1_MAG:_lytic_transglycosylase_domain-containing_protein_[Thermoanaerobaculia_bacterium]	ptg004256l	34.286	2.09E-10	63.9	49
KAB2962098.1_MAG:_hypothetical_protein_F9K18_10290_[Thermoanaerobaculia_bacterium]	ptg005150l	47.917	0.00000987	45.4	51
KAB2962091.1_MAG:_acyl-CoA_thioesterase_[Thermoanaerobaculia_bacterium]	ptg001690l	33.846	2.96E-20	89.4	90
KAB2962089.1_MAG:_enterochelin_esterase_[Thermoanaerobaculia_bacterium]	ptg003764l	24.9	1.45E-12	73.2	59
KAB2962085.1_MAG:_radical_SAM_family_heme_chaperone_HemW,_partial_[Thermoanaerobaculia_bacterium]	ptg005533l	35.561	2.69E-53	193	99
KAB2962084.1_MAG:_hypothetical_protein_F9K18_10310_[Thermoanaerobaculia_bacterium]	ptg002660l	26.582	9.56E-12	71.6	84
KAB2962077.1_MAG:_GTPase_ObgE_[Thermoanaerobaculia_bacterium]	ptg002505l	52.632	4.27E-51	185	86
KAB2962075.1_MAG:_large-conductance_mechanosensitive_channel_protein_MscL_[Thermoanaerobaculia_bacterium]	ptg003839l	36.296	5.48E-16	76.3	97
KAB2962070.1_MAG:_sigma-54-dependent_Fis_family_transcriptional_regulator_[Thermoanaerobaculia_bacterium]	ptg001909l	38.225	6.1E-50	182	80
KAB2962069.1_MAG:_ribosome_silencing_factor_[Thermoanaerobaculia_bacterium]	ptg004055l	34.921	1.43E-15	75.1	92
KAB2962068.1_MAG:_nicotinate_(nicotinamide)_nucleotide_adenylyltransferase_[Thermoanaerobaculia_bacterium]	ptg001008l	33.663	3.87E-26	108	99

KAB2962067.1_MAG:_50S_ribosomal_protein_L27_[Thermoanaerobaculia_bacterium]	ptg002505l	65.333	1.24E-25	101	87
KAB2962066.1_MAG:_50S_ribosomal_protein_L21_[Thermoanaerobaculia_bacterium]	ptg001290l	37.5	1.29E-14	71.2	99
KAB2962079.1_MAG:_metal_ABC_transporter_permease_[Thermoanaerobaculia_bacterium]	ptg004010l	32.37	6.08E-09	60.5	62
KAB2962078.1_MAG:_zinc_ABC_transporter_substrate-binding_protein_[Thermoanaerobaculia_bacterium]	ptg004010l	26.275	1.44E-09	62.8	80
KAB2962064.1_MAG:_helix-turn-helix_domain-containing_protein_[Thermoanaerobaculia_bacterium]	ptg002760l	35.821	1.66E-15	79	55
KAB2962060.1_MAG:_membrane_protein_insertion_efficiency_factor_YidD_[Thermoanaerobaculia_bacterium]	ptg000372l	41.026	4.42E-12	63.2	89
KAB2962059.1_MAG:_DUF4190_domain-containing_protein_[Thermoanaerobaculia_bacterium]	ptg004655l	45.902	1.2E-09	57	58
KAB2962058.1_MAG:_triose-phosphate_isomerase_[Thermoanaerobaculia_bacterium]	ptg003971l	43.2	5.38E-45	165	94
KAB2962056.1_MAG:_peptidyl-prolyl_cis-trans_isomerase_[Thermoanaerobaculia_bacterium]	ptg002976l	59.119	1.34E-58	201	82
KAB2962054.1_MAG:_diguanylate_cyclase_[Thermoanaerobaculia_bacterium]	ptg003673l	41.085	2.92E-22	107	17
KAB2962049.1_MAG:_hypothetical_protein_F9K18_10360,_partial_[Thermoanaerobaculia_bacterium]	ptg002909l	38.028	2.96E-19	98.6	20
KAB2962048.1_MAG:_GGDEF_domain-containing_protein,_partial_[Thermoanaerobaculia_bacterium]	ptg002052l	39.869	5.67E-13	70.5	77
KAB2962047.1_MAG:_prolyl_oligopeptidase_family_serine_peptidase_[Thermoanaerobaculia_bacterium]	ptg002871l	29.723	4.86E-32	139	48
KAB2962044.1_MAG:_RluA_family_pseudouridine_synthase_[Thermoanaerobaculia_bacterium]	ptg002304l	35.616	1.58E-30	125	88
KAB2962043.1_MAG:_asparaginase_[Thermoanaerobaculia_bacterium]	ptg003770l	38.75	1.57E-26	108	95
KAB2962038.1_MAG:_glycosyl_hydrolase,_partial_[Thermoanaerobaculia_bacterium]	ptg005375l	54.857	0	1008	97
KAB2962037.1_MAG:_hypothetical_protein_F9K16_10205_[Thermoanaerobaculia_bacterium]	ptg001353l	26.11	1.1E-09	64.3	89
KAB2962036.1_MAG:_S1_RNA-binding_domain-containing_protein_[Thermoanaerobaculia_bacterium]	ptg001025l	36.311	7.99E-45	170	85
KAB2962035.1_MAG:_Na+/H+_antiporter_NhaC_[Thermoanaerobaculia_bacterium]	ptg005680l	37.188	5.51E-81	278	89
KAB2962034.1_MAG:_carbon_starvation_protein_A_[Thermoanaerobaculia_bacterium]	ptg002928l	31.262	5.23E-50	190	84
KAB2962033.1_MAG:_sulfatase-like_hydrolase/transferase,_partial_[Thermoanaerobaculia_bacterium]	ptg005545l	24.831	1.66E-11	70.9	86
KAB2962029.1_MAG:_RidA_family_protein_[Thermoanaerobaculia_bacterium]	ptg004240l	29.907	0.00000876	47	84
KAB2962028.1_MAG:_hypoxanthine_phosphoribosyltransferase_[Thermoanaerobaculia_bacterium]	ptg000492l	43.662	2.02E-30	120	77
KAB2962026.1_MAG:_purine-nucleoside_phosphorylase,_partial_[Thermoanaerobaculia_bacterium]	ptg002723l	37.129	2.77E-34	134	82
KAB2962018.1_MAG:_hydrogenase_2_operon_protein_HybA_[Thermoanaerobaculia_bacterium]	ptg004084l	33.333	1.15E-13	75.5	52
KAB2962013.1_MAG:_sensor_domain-containing_diguanylate_cyclase_[Thermoanaerobaculia_bacterium]	ptg002052l	35.556	3.89E-14	78.2	47
KAB2962012.1_MAG:_L-lysine_6-transaminase_[Thermoanaerobaculia_bacterium]	ptg002126l	28.431	1.24E-26	117	89
KAB2962009.1_MAG:_hypothetical_protein_F9K16_10260_[Thermoanaerobaculia_bacterium]	ptg004662l	35.652	7.53E-08	61.6	7
KAB2962008.1_MAG:_cyclic_nucleotide-binding_domain-containing_protein_[Thermoanaerobaculia_bacterium]	ptg002907l	23.858	1.62E-15	82	55
KAB2962006.1_MAG:_hypothetical_protein_F9K18_10410,_partial_[Thermoanaerobaculia_bacterium]	ptg000619l	30.034	1.71E-32	134	71
KAB2962005.1_MAG:_tetratricopeptide_repeat_protein,_partial_[Thermoanaerobaculia_bacterium]	ptg001062l	31.935	3.22E-49	190	62
KAB2962004.1_MAG:_EAL_domain-containing_protein_[Thermoanaerobaculia_bacterium]	ptg005727l	31.081	2.34E-27	115	71
KAB2962003.1_MAG:_DNA_repair_protein_RadA,_partial_[Thermoanaerobaculia_bacterium]	LG14	62.295	4.6E-16	84	17
KAB2962002.1_MAG:_phosphoribosylaminoimidazolesuccinocarboxamide_synthase_[Thermoanaerobaculia_bacterium]	ptg001255l	45.69	6.84E-27	114	39

KAB2962000.1_MAG:_glycosyltransferase_family_2_protein_[Thermoanaerobaculia_bacterium]	ptg001909l	46.245	7.79E-64	222	75
KAB2961999.1_MAG:_glycosyltransferase_family_4_protein,_partial_[Thermoanaerobaculia_bacterium]	ptg001909l	28.963	9.84E-23	102	99
KAB2961996.1_MAG:_S8_family_serine_peptidase,_partial_[Thermoanaerobaculia_bacterium]	ptg002612l	28.727	9.79E-08	60.5	29
KAB2961991.1_MAG:_DUF1295_domain-containing_protein_[Thermoanaerobaculia_bacterium]	ptg002738l	43.443	5.12E-19	90.1	46
KAB2961989.1_MAG:_insulinase_family_protein_[Thermoanaerobaculia_bacterium]	ptg002214l	24.823	2.4E-13	78.2	39
KAB2961988.1_MAG:_insulinase_family_protein,_partial_[Thermoanaerobaculia_bacterium]	ptg005195l	35.398	3.99E-27	121	49
KAB2961995.1_MAG:_diguanylate_cyclase,_partial_[Thermoanaerobaculia_bacterium]	ptg005110l	44.375	7.07E-26	120	16
KAB2961987.1_MAG:_aconitate_hydratase_AcnA_[Thermoanaerobaculia_bacterium]	ptg002123l	55.217	0	397	94
KAB2961986.1_MAG:_MotA/TolQ/ExbB_proton_channel_family_protein_[Thermoanaerobaculia_bacterium]	ptg004266l	36.458	5.17E-23	100	83
KAB2961984.1_MAG:_biopolymer_transporter_ExbD_[Thermoanaerobaculia_bacterium]	ptg005754l	28.571	0.00000333	48.9	78
KAB2961983.1_MAG:_energy_transducer_TonB_[Thermoanaerobaculia_bacterium]	ptg005754l	43.902	2.27E-10	63.9	33
KAB2961972.1_MAG:_M20/M25/M40_family_metallo-hydrolase_[Thermoanaerobaculia_bacterium]	ptg002467l	30.128	2.63E-11	70.1	33
KAB2961964.1_MAG:_glutamine-hydrolyzing_carbamoyl-phosphate_synthase_small_subunit_[Thermoanaerobaculia_bacterium]	ptg001693l	49.062	3.46E-91	303	96
KAB2961961.1_MAG:_cation-transporting_P-type_ATPase,_partial_[Thermoanaerobaculia_bacterium]	LG10	53.226	9.11E-09	63.2	9
KAB2961960.1_MAG:_lipase_family_protein_[Thermoanaerobaculia_bacterium]	LG19	29.592	7.25E-08	58.2	55
KAB2961959.1_MAG:_adenosine_deaminase_[Thermoanaerobaculia_bacterium]	ptg005754l	31.677	8.7E-39	150	93
KAB2961958.1_MAG:_pantetheine-phosphate_adenylyltransferase_[Thermoanaerobaculia_bacterium]	ptg004885l	48.077	1.65E-41	151	91
KAB2961953.1_MAG:_DNA_polymerase_III_subunit_beta_[Thermoanaerobaculia_bacterium]	ptg000372l	31.2	1.27E-46	174	100
KAB2961952.1_MAG:_chromosomal_replication_initiator_protein_DnaA_[Thermoanaerobaculia_bacterium]	ptg000372l	51.343	4.4E-108	354	78
KAB2961941.1_MAG:_lysophospholipid_acyltransferase_family_protein_[Thermoanaerobaculia_bacterium]	ptg003364l	33.14	4.72E-24	103	75
KAB2961940.1_MAG:_ATP-binding_cassette_domain-containing_protein_[Thermoanaerobaculia_bacterium]	ptg003058l	36.535	4.81E-86	298	81
KAB2961936.1_MAG:_M1_family_metallopeptidase_[Thermoanaerobaculia_bacterium]	ptg001008l	58.754	0	659	91
KAB2961934.1_MAG:_30S_ribosomal_protein_S12_methylthiotransferase_RimO_[Thermoanaerobaculia_bacterium]	ptg003830l	39.238	8.91E-93	311	95
KAB2961933.1_MAG:_bifunctional_riboflavin_kinase/FAD_synthetase,_partial_[Thermoanaerobaculia_bacterium]	ptg002304l	38.217	4.31E-19	87.4	89
KAB2961928.1_MAG:_endopeptidase_La,_partial_[Thermoanaerobaculia_bacterium]	ptg005527l	53.095	1.72E-140	452	78
KAB2961924.1_MAG:_energy-dependent_translational_throttle_protein_EttA_[Thermoanaerobaculia_bacterium]	ptg003536l	60.721	0	584	99
KAB2961923.1_MAG:_ParA_family_protein_[Thermoanaerobaculia_bacterium]	ptg003829l	33.468	1.03E-38	149	74
KAB2961922.1_MAG:_two-component_sensor_histidine_kinase,_partial_[Thermoanaerobaculia_bacterium]	ptg003967l	34.759	4.68E-11	64.7	96
KAB2961921.1_MAG:_heme_A_synthase,_partial_[Thermoanaerobaculia_bacterium]	ptg000819l	55.556	1.83E-26	114	31
KAB2961912.1_MAG:_PAS_domain-containing_sensor_histidine_kinase,_partial_[Thermoanaerobaculia_bacterium]	ptg004158l	43.902	3.97E-09	57.4	53
KAB2961911.1_MAG:_6-carboxytetrahydropterin_synthase_[Thermoanaerobaculia_bacterium]	ptg004575l	37.333	5.28E-23	97.8	90
KAB2961910.1_MAG:_adenosylmethionine--8-amino-7-oxononanoate_transaminase_[Thermoanaerobaculia_bacterium]	ptg005265l	40.338	4.1E-83	282	95

KAB2961909.1_MAG:_dethiobiotin_synthase_[Thermoanaerobaculia_bacterium]	ptg003831l	36.646	1.31E-12	70.1	66
KAB2961906.1_MAG:_efflux_RND_transporter_permease_subunit_[Thermoanaerobaculia_bacterium]	ptg004798l	26.358	1.19E-43	178	89
KAB2961903.1_MAG:_winged_helix-turn-helix_transcriptional_regulator_[Thermoanaerobaculia_bacterium]	ptg005046l	37.681	0.000000309	50.8	59
KAB2961899.1_MAG:_MBL_fold_metallo-hydrolase_[Thermoanaerobaculia_bacterium]	ptg004856l	33.962	6.11E-32	126	93
KAB2961897.1_MAG:_3-isopropylmalate_dehydrogenase_[Thermoanaerobaculia_bacterium]	ptg001005l	39.414	3.43E-43	164	82
KAB2961885.1_MAG:_30S_ribosomal_protein_S16_[Thermoanaerobaculia_bacterium]	ptg005431l	46.667	3.15E-16	74.7	91
KAB2961884.1_MAG:_signal_recognition_particle_protein_[Thermoanaerobaculia_bacterium]	ptg002916l	45.727	9.36E-102	336	98
KAB2961883.1_MAG:_pyruvate_kinase,_partial_[Thermoanaerobaculia_bacterium]	ptg004880l	43.373	1.33E-67	233	98
KAB2961882.1_MAG:_3-deoxy-D-manno-octulosonic_acid_transferase_[Thermoanaerobaculia_bacterium]	ptg004774l	32.448	3.69E-45	171	82
KAB2961881.1_MAG:_type_II_secretion_system_F_family_protein_[Thermoanaerobaculia_bacterium]	ptg004018l	28.117	1.05E-31	131	93
KAB2961880.1_MAG:_type_II/IV_secretion_system_protein_[Thermoanaerobaculia_bacterium]	ptg005962l	44.272	2.24E-118	389	93
KAB2961873.1_MAG:_FAD:protein_FMN_transferase_[Thermoanaerobaculia_bacterium]	ptg002166l	33.46	9.61E-34	135	73
KAB2961871.1_MAG:_DUF3570_domain-containing_protein_[Thermoanaerobaculia_bacterium]	ptg004779l	22.899	7.59E-12	71.2	77
KAB2961868.1_MAG:_ATP-binding_protein,_partial_[Thermoanaerobaculia_bacterium]	ptg003039l	31.658	6.51E-14	78.6	38
KAB2961867.1_MAG:_Zn-dependent_exopeptidase_M28,_partial_[Thermoanaerobaculia_bacterium]	ptg001897l	29.944	7.65E-12	71.2	40
KAB2961865.1_MAG:_50S_ribosomal_protein_L33_[Thermoanaerobaculia_bacterium]	ptg005430l	60.784	0.00000014	48.5	100
KAB2961859.1_MAG:_SDR_family_NAD(P)-dependent_oxidoreductase,_partial_[Thermoanaerobaculia_bacterium]	ptg003131l	38.249	3.12E-39	147	95
KAB2961855.1_MAG:_sugar_transferase_[Thermoanaerobaculia_bacterium]	ptg004085l	47.34	1.05E-46	178	39
KAB2961854.1_MAG:_response_regulator_transcription_factor_[Thermoanaerobaculia_bacterium]	LG03	34.468	9.76E-35	135	92
KAB2961853.1_MAG:_DUF4118_domain-containing_protein_[Thermoanaerobaculia_bacterium]	LG03	26.19	6.81E-18	89.4	54
KAB2961852.1_MAG:_potassium_transporter_Kup_[Thermoanaerobaculia_bacterium]	ptg005550l	42.197	1.98E-103	180	90
KAB2961851.1_MAG:_type_II/IV_secretion_system_protein_[Thermoanaerobaculia_bacterium]	ptg004468l	39.384	3.88E-125	410	99
KAB2961848.1_MAG:_bifunctional_methionine_sulfoxide_reductase_B/A_protein_[Thermoanaerobaculia_bacterium]	ptg002618l	63.38	7.42E-57	203	82
KAB2961844.1_MAG:_cell_division_protein_FtsZ_[Thermoanaerobaculia_bacterium]	ptg004510l	49.811	3.64E-57	206	61
KAB2961843.1_MAG:_cell_division_protein_FtsA_[Thermoanaerobaculia_bacterium]	ptg004510l	35.802	7.09E-48	152	82
KAB2961841.1_MAG:_UDP-N-acetylmuramate--L-alanine_ligase_[Thermoanaerobaculia_bacterium]	ptg004510l	40.493	5.9E-83	174	95
KAB2961840.1_MAG:_thiamine_pyrophosphate-binding_protein,_partial_[Thermoanaerobaculia_bacterium]	ptg002304l	35.484	2.04E-18	86.3	92
KAB2961839.1_MAG:_TonB-dependent_receptor_[Thermoanaerobaculia_bacterium]	ptg003548l	40.789	0.0000079	53.5	10
KAB2961838.1_MAG:_hypothetical_protein_F9K16_10805,_partial_[Thermoanaerobaculia_bacterium]	ptg001878l	27.407	5.37E-14	75.5	89
KAB2961836.1_MAG:_ATP-binding_cassette_domain-containing_protein_[Thermoanaerobaculia_bacterium]	ptg004055l	49.18	2.05E-52	193	42
KAB2961832.1_MAG:_PDZ_domain-containing_protein_[Thermoanaerobaculia_bacterium]	ptg004479l	42.915	7E-51	188	57
KAB2961831.1_MAG:_hypothetical_protein_F9K18_10675,_partial_[Thermoanaerobaculia_bacterium]	ptg005331l	48.485	6.39E-11	62	51

KAB2961827.1_MAG:_ABC-F_family_ATP-binding_cassette_domain-containing_protein_[Thermoanaerobaculia_bacterium]	ptg005398l	39.658	4.43E-110	368	82
KAB2961825.1_MAG:_Do_family_serine_endopeptidase,_partial_[Thermoanaerobaculia_bacterium]	ptg004479l	36.83	1.35E-64	230	90
KAB2961824.1_MAG:_sigma-54-dependent_Fis_family_transcriptional_regulator_[Thermoanaerobaculia_bacterium]	ptg005150l	40.492	8.8E-102	337	95
KAB2961823.1_MAG:_PLP-dependent_transferase_[Thermoanaerobaculia_bacterium]	ptg004895l	39.51	5.51E-78	265	96
KAB2961819.1_MAG:_signal_peptidase_I_[Thermoanaerobaculia_bacterium]	ptg002902l	33.889	8.11E-21	89.7	74
KAB2961818.1_MAG:_helix-hairpin-helix_domain-containing_protein_[Thermoanaerobaculia_bacterium]	ptg000732l	37.931	7.62E-08	52.8	48
KAB2961816.1_MAG:_divalent-cation_tolerance_protein_CutA_[Thermoanaerobaculia_bacterium]	ptg002612l	38.298	2.04E-12	66.2	73
KAB2961814.1_MAG:_sulfatase,_partial_[Thermoanaerobaculia_bacterium]	ptg001401l	22.487	4.33E-13	77.4	44
KAB2961813.1_MAG:_RidA_family_protein_[Thermoanaerobaculia_bacterium]	ptg004297l	33.835	5.25E-15	73.6	95
KAB2961812.1_MAG:_hypoxanthine_phosphoribosyltransferase_[Thermoanaerobaculia_bacterium]	ptg000492l	38.372	5.42E-27	110	93
KAB2961810.1_MAG:_purine-nucleoside_phosphorylase_[Thermoanaerobaculia_bacterium]	ptg004680l	44.242	1.62E-29	121	69
KAB2961809.1_MAG:_lysine--tRNA_ligase_[Thermoanaerobaculia_bacterium]	ptg004479l	47.894	5.44E-120	392	83
KAB2961807.1_MAG:_hypothetical_protein_F9K18_10700,_partial_[Thermoanaerobaculia_bacterium]	ptg002909l	37.083	1.16E-20	103	23
KAB2961805.1_MAG:_glycosyltransferase_family_39_protein_[Thermoanaerobaculia_bacterium]	ptg003471l	31.549	3.66E-21	102	48
KAB2961804.1_MAG:_glycosyltransferase_family_2_protein_[Thermoanaerobaculia_bacterium]	ptg004788l	43.363	5.6E-48	173	91
KAB2961802.1_MAG:_sulfatase_[Thermoanaerobaculia_bacterium]	ptg003090l	30	3.97E-20	97.8	73
KAB2961798.1_MAG:_carbon-nitrogen_hydrolase_family_protein,_partial_[Thermoanaerobaculia_bacterium]	ptg003452l	29.515	1.71E-22	99.8	89
KAB2961797.1_MAG:_polyphosphate_kinase_2_family_protein_[Thermoanaerobaculia_bacterium]	ptg004579l	47.581	6.27E-69	236	76
KAB2961796.1_MAG:_pyridoxal_phosphate-dependent_aminotransferase_[Thermoanaerobaculia_bacterium]	ptg004905l	27.554	1.91E-18	91.3	84
KAB2961792.1_MAG:_type_II/IV_secretion_system_protein_[Thermoanaerobaculia_bacterium]	ptg004468l	42.48	2.39E-121	397	93
KAB2961791.1_MAG:_type_II_secretion_system_F_family_protein,_partial_[Thermoanaerobaculia_bacterium]	ptg005301l	30.421	4.11E-33	135	75
KAB2961790.1_MAG:_serine/threonine_protein_kinase,_partial_[Thermoanaerobaculia_bacterium]	ptg005260l	38.428	2.48E-32	130	76
KAB2961785.1_MAG:_hypothetical_protein_F9K16_10965,_partial_[Thermoanaerobaculia_bacterium]	ptg005527l	25.356	1.08E-15	83.2	89
KAB2961779.1_MAG:_serine--tRNA_ligase_[Thermoanaerobaculia_bacterium]	ptg005746l	52.815	1.09E-114	373	85
KAB2961774.1_MAG:_transketolase,_partial_[Thermoanaerobaculia_bacterium]	ptg004917l	47.576	7.92E-102	333	91
KAB2961773.1_MAG:_heavy_metal_translocating_P-type_ATPase_[Thermoanaerobaculia_bacterium]	ptg003880l	34.056	5.75E-29	128	66
KAB2961772.1_MAG:_bifunctional_(p)ppGpp_synthetase/guanosine-3',5'-bis(diphosphate)_3'-pyrophosphohydrolase_[Thermoanaerobaculia_bacterium]	ptg002030l	34.294	5.44E-128	424	94
KAB2961771.1_MAG:_protein_translocase_subunit_SecF_[Thermoanaerobaculia_bacterium]	ptg004256l	42.927	2.78E-44	168	51
KAB2961770.1_MAG:_protein_translocase_subunit_SecD_[Thermoanaerobaculia_bacterium]	ptg005746l	38.182	1.2E-74	261	91
KAB2961769.1_MAG:_preprotein_translocase_subunit_YajC_[Thermoanaerobaculia_bacterium]	ptg005746l	43.421	0.000000277	50.4	70
KAB2961768.1_MAG:_tRNA-guanine_transglycosylase,_partial_[Thermoanaerobaculia_bacterium]	ptg004256l	50.282	5.5E-55	192	81
KAB2961383.1_MAG:_dTDP-glucose_4,6-dehydratase_[Thermoanaerobaculia_bacterium]	ptg001690l	40.278	5.89E-81	272	96

KAB2961382.1_MAG:_transketolase_[Thermoanaerobaculia_bacterium]	ptg002612l	30.091	3.25E-29	130	75
KAB2961377.1_MAG:_beta-lactamase_family_protein,_partial_[Thermoanaerobaculia_bacterium]	ptg002123l	27.041	4.1E-11	68.6	51
KAB2961374.1_MAG:_STAS_domain-containing_protein_[Thermoanaerobaculia_bacterium]	ptg003363l	37.705	0.00000313	47.8	55
KAB2961373.1_MAG:_ATP-binding_protein_[Thermoanaerobaculia_bacterium]	ptg004467l	38.211	2.76E-18	84	65
KAB2961370.1_MAG:_acyl-CoA_dehydrogenase_[Thermoanaerobaculia_bacterium]	ptg004179l	25.36	1.47E-22	104	81
KAB2961369.1_MAG:_class_II_fructose-bisphosphatase_[Thermoanaerobaculia_bacterium]	ptg004284l	48.758	5.26E-80	268	96
KAB2961368.1_MAG:_dCTP_deaminase_[Thermoanaerobaculia_bacterium]	ptg002214l	32.804	8.37E-18	82.8	99
KAB2961364.1_MAG:_protein-L-isoaspartate(D-aspartate)_O-methyltransferase_[Thermoanaerobaculia_bacterium]	ptg004026l	43.293	1.01E-29	120	69
KAB2961362.1_MAG:_GNAT_family_N-acetyltransferase_[Thermoanaerobaculia_bacterium]	ptg001473l	33.516	9.54E-22	97.1	76
KAB2961361.1_MAG:_carbon-nitrogen_hydrolase_family_protein_[Thermoanaerobaculia_bacterium]	ptg001473l	35.842	3.89E-51	184	94
KAB2961358.1_MAG:_methylated-DNA--[protein]-cysteine_S-methyltransferase_[Thermoanaerobaculia_bacterium]	ptg005680l	48.299	1.29E-25	105	90
KAB2961357.1_MAG:_DNA-3-methyladenine_glycosylase_2_family_protein,_partial_[Thermoanaerobaculia_bacterium]	ptg005680l	32.37	3.85E-15	77.8	73
KAB2961354.1_MAG:_UDP-N-acetylmuramoyl-L-alanyl-D-glutamate--2,6-diaminopimelate_ligase,_partial_[Thermoanaerobaculia_bacterium]	ptg003536l	30.423	1.26E-23	107	90
KAB2961352.1_MAG:_RHS_repeat-associated_core_domain-containing_protein_[Thermoanaerobaculia_bacterium]	ptg002381l	29.707	8.47E-17	87.8	53
KAB2961348.1_MAG:_cold-shock_protein_[Thermoanaerobaculia_bacterium]	ptg003498l	57.377	6.66E-20	84.3	91
KAB2961347.1_MAG:_cold-shock_protein_[Thermoanaerobaculia_bacterium]	ptg003131l	57.812	2.01E-20	85.9	96
KAB2961346.1_MAG:_RNA_pseudouridine_synthase,_partial_[Thermoanaerobaculia_bacterium]	ptg003213l	33.742	4.65E-19	89.7	67
KAB2961344.1_MAG:_serine/threonine_protein_kinase,_partial_[Thermoanaerobaculia_bacterium]	ptg005905l	38.722	2.96E-35	138	90
KAB2961343.1_MAG:_endopeptidase_La,_partial_[Thermoanaerobaculia_bacterium]	ptg005527l	31.045	7.01E-41	157	93
KAB2961341.1_MAG:_molybdopterin-dependent_oxidoreductase_[Thermoanaerobaculia_bacterium]	LG04	28.195	2.01E-27	87.4	55
KAB2961340.1_MAG:_NAD(P)-dependent_oxidoreductase_[Thermoanaerobaculia_bacterium]	ptg002700l	33.333	2.51E-08	58.5	39
KAB2961330.1_MAG:_bifunctional_(p)ppGpp_synthetase/guanosine-3',5'-bis(diphosphate)_3'-pyrophosphohydrolase,_partial_[Thermoanaerobaculia_bacterium]	ptg002030l	33.787	1.29E-124	414	95
KAB2961329.1_MAG:_50S_ribosomal_protein_L28_[Thermoanaerobaculia_bacterium]	ptg005398l	49.123	0.000000348	48.9	74
KAB2961328.1_MAG:_type_IV_pilus_assembly_protein_PilM_[Thermoanaerobaculia_bacterium]	ptg004863l	33.913	2.69E-47	175	99
KAB2961326.1_MAG:_type_4a_pilus_biogenesis_protein_PilO_[Thermoanaerobaculia_bacterium]	ptg004771l	27.097	0.00000359	50.4	71
KAB2961323.1_MAG:_DUF3488_domain-containing_protein,_partial_[Thermoanaerobaculia_bacterium]	ptg004759l	47.126	6.28E-16	85.9	15
KAB2961322.1_MAG:_DUF58_domain-containing_protein_[Thermoanaerobaculia_bacterium]	ptg000568l	27.778	0.000000127	57.4	43
KAB2961320.1_MAG:_Rne/Rng_family_ribonuclease_[Thermoanaerobaculia_bacterium]	ptg005709l	41	3.77E-100	334	99
KAB2961319.1_MAG:_rod_shape-determining_protein_RodA_[Thermoanaerobaculia_bacterium]	ptg004510l	36.364	1.29E-30	127	68
KAB2961315.1_MAG:_ABC_transporter_permease_[Thermoanaerobaculia_bacterium]	ptg004026l	46.341	1.58E-54	195	75

KAB2961313.1_MAG:_2-isopropylmalate_synthase_[Thermoanaerobaculia_bacterium]	ptg002871l	29.043	1.19E-23	108	68
KAB2961311.1_MAG:_NAD-dependent_epimerase/dehydratase_family_protein,_partial_[Thermoanaerobaculia_bacterium]	ptg003045l	66.667	6.49E-08	50.1	53
KAB2961310.1_MAG:_efflux_RND_transporter_permease_subunit_[Thermoanaerobaculia_bacterium]	ptg004670l	34.522	3.21E-145	485	98
KAB2961306.1_MAG:_protein_kinase_[Thermoanaerobaculia_bacterium]	ptg006032l	36.655	1.8E-42	172	33
KAB2960975.1_MAG:_SDR_family_oxidoreductase_[Thermoanaerobaculia_bacterium]	ptg002700l	36.774	1.14E-23	87	95
KAB2960973.1_MAG:_dTMP_kinase_[Thermoanaerobaculia_bacterium]	ptg003947l	33.871	5.02E-25	106	82
KAB2960972.1_MAG:_TerC_family_protein_[Thermoanaerobaculia_bacterium]	LG22	39.773	6.92E-11	67	63
KAB2960971.1_MAG:_undecaprenyl-diphosphate_phosphatase_[Thermoanaerobaculia_bacterium]	ptg003644l	34.225	2.56E-21	97.1	68
KAB2960970.1_MAG:_glucose-1-phosphate_thymidylyltransferase_RfbA_[Thermoanaerobaculia_bacterium]	ptg004111l	61.246	3.65E-113	362	99
KAB2960969.1_MAG:_dTDP-4-dehydrorhamnose_3,5-epimerase_[Thermoanaerobaculia_bacterium]	ptg003827l	59.429	7.11E-66	221	94
KAB2960967.1_MAG:_ParB/RepB/Spo0J_family_partition_protein,_partial_[Thermoanaerobaculia_bacterium]	ptg001818l	50.588	4.5E-37	140	78
KAB2960966.1_MAG:_ParA_family_protein_[Thermoanaerobaculia_bacterium]	ptg000372l	52.79	1.29E-54	192	92
KAB2960963.1_MAG:_EAL_domain-containing_protein_[Thermoanaerobaculia_bacterium]	ptg005865l	44.018	3.54E-113	383	67
KAB2960959.1_MAG:_alcohol_dehydrogenase_catalytic_domain-containing_protein,_partial_[Thermoanaerobaculia_bacterium]	ptg000693l	36.667	9.91E-19	84	100
KAB2960958.1_MAG:_NAD-dependent_epimerase/dehydratase_family_protein_[Thermoanaerobaculia_bacterium]	ptg002313l	33.948	1.09E-22	103	70
KAB2960957.1_MAG:_succinate-semialdehyde_dehydrogenase_(NADP(+)),_partial_[Thermoanaerobaculia_bacterium]	ptg004321l	30.973	1.2E-52	196	91
KAB2960956.1_MAG:_fumarate_reductase/succinate_dehydrogenase_flavoprotein_subunit_[Thermoanaerobaculia_bacterium]	ptg002316l	65.149	0	784	100
KAB2960955.1_MAG:_succinate_dehydrogenase_cytochrome_b_subunit_[Thermoanaerobaculia_bacterium]	ptg002316l	41.553	1.37E-33	130	94
KAB2960954.1_MAG:_succinate_dehydrogenase/fumarate_reductase_iron-sulfur_subunit_[Thermoanaerobaculia_bacterium]	ptg003304l	59.917	4.97E-100	323	94
KAB2960951.1_MAG:_aminoacyl-tRNA_hydrolase,_partial_[Thermoanaerobaculia_bacterium]	ptg004575l	44.762	4.55E-10	58.5	97
KAB2960949.1_MAG:_site-2_protease_family_protein_[Thermoanaerobaculia_bacterium]	ptg003441l	33.498	1.2E-13	76.6	45
KAB2960948.1_MAG:_SOS_response-associated_peptidase,_partial_[Thermoanaerobaculia_bacterium]	ptg003566l	35.032	1.32E-14	75.9	68
KAB2960947.1_MAG:_M13_family_metallopeptidase_[Thermoanaerobaculia_bacterium]	ptg001690l	39.009	7.16E-50	192	86
KAB2960946.1_MAG:_M2_family_metallopeptidase_[Thermoanaerobaculia_bacterium]	ptg003548l	55.712	0	676	93
KAB2960943.1_MAG:_nucleoside_triphosphate_pyrophosphohydrolase_[Thermoanaerobaculia_bacterium]	ptg002030l	35.938	5.08E-37	143	84
KAB2960942.1_MAG:_arylesterase_[Thermoanaerobaculia_bacterium]	ptg001995l	41.892	1.83E-29	119	60
KAB2960941.1_MAG:_ATP-binding_cassette_domain-containing_protein,_partial_[Thermoanaerobaculia_bacterium]	ptg001995l	53.153	7.48E-25	102	75
KAB2960935.1_MAG:_hypothetical_protein_F9K16_11455_[Thermoanaerobaculia_bacterium]	ptg005297l	34.132	1.11E-45	171	90
KAB2960934.1_MAG:_GTPase_HflX_[Thermoanaerobaculia_bacterium]	ptg004355l	41.304	5.65E-35	87	54
KAB2960933.1_MAG:_TlpA_family_protein_disulfide_reductase_[Thermoanaerobaculia_bacterium]	ptg005108l	27.869	3.45E-08	56.2	63
KAB2960930.1_MAG:_sulfatase-like_hydrolase/transferase_[Thermoanaerobaculia_bacterium]	ptg005776l	27.937	5.31E-21	100	77

KAB2960928.1_MAG:_molybdopterin-dependent_oxidoreductase_[Thermoanaerobaculia_bacterium]	ptg004306l	40.823	5.85E-129	422	99
KAB2960927.1_MAG:_formate_dehydrogenase_accessory_sulfurtransferase_FdhD_[Thermoanaerobaculia_bacterium]	ptg002907l	45.85	5.31E-41	154	86
KAB2960926.1_MAG:_formate--tetrahydrofolate_ligase,_partial_[Thermoanaerobaculia_bacterium]	LG24	51.613	3.35E-08	60.1	14
KAB2960924.1_MAG:_sulfurtransferase_[Thermoanaerobaculia_bacterium]	ptg002618l	28.996	1.61E-34	135	92
KAB2960921.1_MAG:_cytochrome_c_oxidase_subunit_3_family_protein_[Thermoanaerobaculia_bacterium]	ptg002453l	48.182	8.1E-21	92.8	96
KAB2960920.1_MAG:_cytochrome_c_oxidase_subunit_II_[Thermoanaerobaculia_bacterium]	ptg002453l	36.334	8.15E-52	187	92
KAB2960919.1_MAG:_cytochrome_c_oxidase_subunit_I_[Thermoanaerobaculia_bacterium]	ptg001690l	59.81	0	579	96
KAB2960914.1_MAG:_hypothetical_protein_F9K18_10995,_partial_[Thermoanaerobaculia_bacterium]	ptg004075l	37.273	3.48E-14	79.3	24
KAB2960692.1_MAG:_superoxide_dismutase_[Thermoanaerobaculia_bacterium]	ptg001604l	49	1.34E-51	183	84
KAB2960691.1_MAG:_4-hydroxy-3-methylbut-2-enyl_diphosphate_reductase_[Thermoanaerobaculia_bacterium]	ptg005010l	33.571	1.89E-31	127	92
KAB2960686.1_MAG:_bifunctional_methylenetetrahydrofolate_dehydrogenase/methenyltetrahydrofolate_cyclohydrolase_FolD,_partial_[Thermoanaerobaculia_bacterium]	ptg001690l	46.753	1.79E-73	249	99
KAB2960685.1_MAG:_ubiquinone/menaquinone_biosynthesis_methyltransferase_[Thermoanaerobaculia_bacterium]	ptg004457l	44.589	1.29E-50	180	95
KAB2960684.1_MAG:_50S_ribosomal_protein_L13_[Thermoanaerobaculia_bacterium]	ptg000819l	47.26	4.98E-31	120	98
KAB2960683.1_MAG:_30S_ribosomal_protein_S9_[Thermoanaerobaculia_bacterium]	ptg000819l	60.8	5.85E-27	107	96
KAB2960682.1_MAG:_30S_ribosomal_protein_S2_[Thermoanaerobaculia_bacterium]	ptg005656l	50.655	1.93E-67	229	89
KAB2960681.1_MAG:_translation_elongation_factor_Ts_[Thermoanaerobaculia_bacterium]	ptg001624l	49.239	7.14E-47	168	90
KAB2960680.1_MAG:_UMP_kinase_[Thermoanaerobaculia_bacterium]	ptg002436l	52.263	1.98E-66	226	99
KAB2960679.1_MAG:_ribosome_recycling_factor_[Thermoanaerobaculia_bacterium]	ptg005656l	43.333	1.13E-42	155	98
KAB2960676.1_MAG:_cyclic_pyranopterin_monophosphate_synthase_MoaC_[Thermoanaerobaculia_bacterium]	ptg002907l	52.318	2.2E-33	127	93
KAB2960675.1_MAG:_HU_family_DNA-binding_protein_[Thermoanaerobaculia_bacterium]	ptg001170l	45.652	4.65E-15	72	97
KAB2960674.1_MAG:_hypothetical_protein_F9K18_11020,_partial_[Thermoanaerobaculia_bacterium]	ptg003098l	34.815	9.9E-09	57.4	73
KAB2960673.1_MAG:_sulfatase-like_hydrolase/transferase,_partial_[Thermoanaerobaculia_bacterium]	LG10	24.605	3.84E-18	92.8	69
KAB2960672.1_MAG:_TonB-dependent_receptor_[Thermoanaerobaculia_bacterium]	ptg001925l	28.085	1.57E-09	66.2	23
KAB2960670.1_MAG:_50S_ribosomal_protein_L33_[Thermoanaerobaculia_bacterium]	ptg005430l	62.745	0.00000324	44.7	100
KAB2960669.1_MAG:_superoxide_dismutase_[Thermoanaerobaculia_bacterium]	ptg001604l	58.794	4.51E-64	217	99
KAB2960668.1_MAG:_tRNA_lysidine(34)_synthetase_TilS,_partial_[Thermoanaerobaculia_bacterium]	ptg001005l	40.833	7.29E-11	66.6	39
KAB2960666.1_MAG:_arginine--tRNA_ligase_[Thermoanaerobaculia_bacterium]	ptg005430l	22.205	1.04E-21	104	99
KAB2960665.1_MAG:_macro_domain-containing_protein_[Thermoanaerobaculia_bacterium]	ptg003471l	30.597	0.000000265	53.5	70
KAB2960662.1_MAG:_2,3-bisphosphoglycerate-independent_phosphoglycerate_mutase_[Thermoanaerobaculia_bacterium]	ptg002573l	26.615	7.72E-14	77.4	89
KAB2960660.1_MAG:_nucleoside_deaminase_[Thermoanaerobaculia_bacterium]	ptg004856l	53.488	6.52E-30	117	86
KAB2960659.1_MAG:_membrane_protein_insertion_efficiency_factor_YidD_[Thermoanaerobaculia_bacterium]	LG10	44.615	7.61E-08	50.4	96
KAB2960658.1_MAG:_ribonuclease_P_protein_component_[Thermoanaerobaculia_bacterium]	ptg005113l	36.667	5.22E-11	62	93

KAB2960656.1_MAG:_DNA_polymerase_III_subunit_gamma/tau,_partial_[Thermoanaerobaculia_bacterium]	ptg002760l	42.053	2.67E-78	264	88
KAB2960654.1_MAG:_RnfABCDGE_type_electron_transport_complex_subunit_B_[Thermoanaerobaculia_bacterium]	ptg002214l	29.31	9.1E-10	63.2	57
KAB2960653.1_MAG:_DUF420_domain-containing_protein_[Thermoanaerobaculia_bacterium]	ptg002022l	43.925	1.52E-14	72.8	77
KAB2960652.1_MAG:_SCO_family_protein_[Thermoanaerobaculia_bacterium]	ptg000340l	33.548	1.12E-14	77.8	55
KAB2960651.1_MAG:_protoheme_IX_farnesyltransferase_[Thermoanaerobaculia_bacterium]	ptg000340l	42.408	1.13E-18	90.1	64
KAB2960649.1_MAG:_DNA_polymerase_I,_partial_[Thermoanaerobaculia_bacterium]	ptg004532l	44.309	1.37E-114	378	87
KAB2960644.1_MAG:_MBL_fold_metallo-hydrolase_[Thermoanaerobaculia_bacterium]	ptg005920l	43.307	3.73E-58	202	99
KAB2960643.1_MAG:_3-phosphoshikimate_1-carboxyvinyltransferase_[Thermoanaerobaculia_bacterium]	ptg002316l	37.629	3.94E-59	213	86
KAB2960391.1_MAG:_response_regulator,_partial_[Thermoanaerobaculia_bacterium]	ptg003967l	45.794	3.78E-23	96.7	85
KAB2960381.1_MAG:_F0F1_ATP_synthase_subunit_alpha_[Thermoanaerobaculia_bacterium]	ptg001818l	44.602	2.75E-143	462	97
KAB2960380.1_MAG:_ATP_synthase_F1_subunit_gamma_[Thermoanaerobaculia_bacterium]	ptg002658l	42.049	6.21E-60	209	94
KAB2960379.1_MAG:_F0F1_ATP_synthase_subunit_beta_[Thermoanaerobaculia_bacterium]	ptg004470l	74.194	0	670	96
KAB2960378.1_MAG:_ATP_synthase_F1_subunit_epsilon_[Thermoanaerobaculia_bacterium]	ptg002658l	34.921	0.000000134	52.4	95
KAB2960374.1_MAG:_5-formyltetrahydrofolate_cyclo-ligase_[Thermoanaerobaculia_bacterium]	ptg005341l	29.114	5.7E-12	70.9	45
KAB2960372.1_MAG:_RnfABCDGE_type_electron_transport_complex_subunit_B_[Thermoanaerobaculia_bacterium]	ptg002214l	26.163	1.51E-09	62.4	58
KAB2960370.1_MAG:_nicotinate_phosphoribosyltransferase_[Thermoanaerobaculia_bacterium]	ptg005048l	53.32	5.56E-161	509	97
KAB2960369.1_MAG:_ATP-dependent_6-phosphofructokinase_[Thermoanaerobaculia_bacterium]	ptg003223l	47.139	4.58E-91	302	98
KAB2960368.1_MAG:_hypothetical_protein_F9K16_11800,_partial_[Thermoanaerobaculia_bacterium]	ptg005227l	27.985	3.74E-20	96.3	68
KAB2960367.1_MAG:_Hsp20/alpha_crystallin_family_protein_[Thermoanaerobaculia_bacterium]	ptg002102l	34.921	2.38E-15	75.5	83
KAB2960366.1_MAG:_GHKL_domain-containing_protein_[Thermoanaerobaculia_bacterium]	ptg004026l	32.692	2.75E-19	95.5	44
KAB2960363.1_MAG:_divalent_metal_cation_transporter,_partial_[Thermoanaerobaculia_bacterium]	LG10	50	1.29E-20	99	21
KAB2960352.1_MAG:_PP2C_family_protein-serine/threonine_phosphatase_[Thermoanaerobaculia_bacterium]	ptg004476l	31.048	1.01E-16	86.7	59
KAB2960351.1_MAG:_D-alanyl-D-alanine_carboxypeptidase/D-alanyl-D-alanine-endopeptidase,_partial_[Thermoanaerobaculia_bacterium]	ptg005301l	25.606	2.98E-20	98.2	79
KAB2960341.1_MAG:_YpdA_family_putative_bacillithiol_disulfide_reductase_[Thermoanaerobaculia_bacterium]	ptg003861l	27.737	2.61E-12	72.4	69
KAB2960340.1_MAG:_prolipoprotein_diacylglyceryl_transferase_[Thermoanaerobaculia_bacterium]	ptg004916l	29.48	4.9E-09	60.5	62
KAB2960339.1_MAG:_magnesium_transporter_[Thermoanaerobaculia_bacterium]	ptg005611l	33.898	7.88E-55	201	89
KAB2960338.1_MAG:_FAD-binding_oxidoreductase,_partial_[Thermoanaerobaculia_bacterium]	ptg001870l	40.909	0.000000744	48.5	76
KAB2960335.1_MAG:_PhoH_family_protein_[Thermoanaerobaculia_bacterium]	ptg005772l	29.018	6.42E-22	103	49
KAB2960333.1_MAG:_ATP-dependent_metallopeptidase_FtsH/Yme1/Tma_family_protein_[Thermoanaerobaculia_bacterium]	ptg003039l	56.478	2.49E-155	499	77
KAB2960141.1_MAG:_electron_transport_complex_subunit_RsxC,_partial_[Thermoanaerobaculia_bacterium]	ptg002214l	36.649	2.52E-65	229	96
KAB2960140.1_MAG:_RnfABCDGE_type_electron_transport_complex_subunit_D_[Thermoanaerobaculia_bacterium]	ptg002214l	37.688	7.01E-26	89.4	92
KAB2960138.1_MAG:_hypothetical_protein_F9K16_11940_[Thermoanaerobaculia_bacterium]	ptg003077l	50.662	4.9E-101	330	87
KAB2960137.1_MAG:_isoaspartyl_peptidase/L-asparaginase_[Thermoanaerobaculia_bacterium]	ptg002909l	48.822	9.72E-75	251	99

KAB2960135.1_MAG:_ATP-grasp_domain-containing_protein_[Thermoanaerobaculia_bacterium]	ptg004466l	48.657	1.86E-91	302	99
KAB2960133.1_MAG:_hypothetical_protein_F9K18_11270_[Thermoanaerobaculia_bacterium]	ptg005693l	29.63	4.65E-16	82.8	74
KAB2960132.1_MAG:_M20/M25/M40_family_metallo-hydrolase,_partial_[Thermoanaerobaculia_bacterium]	ptg002658l	32.389	3.78E-54	201	85
KAB2960131.1_MAG:_patatin-like_phospholipase_family_protein_[Thermoanaerobaculia_bacterium]	ptg005195l	33.333	2.23E-11	67.4	62
KAB2960130.1_MAG:_acetate--CoA_ligase_[Thermoanaerobaculia_bacterium]	LG03	61.311	0	738	95
KAB2960128.1_MAG:_LemA_family_protein_[Thermoanaerobaculia_bacterium]	ptg000492l	37.158	1.37E-24	104	89
KAB2960127.1_MAG:_dihydrofolate_reductase_[Thermoanaerobaculia_bacterium]	ptg001366l	52.344	1.22E-33	129	73
KAB2960126.1_MAG:_thymidylate_synthase_[Thermoanaerobaculia_bacterium]	ptg004689l	70.076	4.28E-130	409	100
KAB2960125.1_MAG:_ATP-binding_protein,_partial_[Thermoanaerobaculia_bacterium]	ptg004355l	41.935	3.46E-26	109	100
KAB2960119.1_MAG:_HD_domain-containing_protein,_partial_[Thermoanaerobaculia_bacterium]	ptg005035l	34.615	1.84E-32	135	40
KAB2960117.1_MAG:_signal_recognition_particle-docking_protein_FtsY_[Thermoanaerobaculia_bacterium]	ptg004885l	43.369	3.04E-57	202	91
KAB2960114.1_MAG:_glutamine_synthetase_[Thermoanaerobaculia_bacterium]	ptg004075l	29.648	2.05E-28	123	76
KAB2960113.1_MAG:_diguanylate_cyclase_[Thermoanaerobaculia_bacterium]	ptg004532l	38.125	1.43E-18	95.1	23
KAB2960112.1_MAG:_ABC_transporter_permease_subunit_[Thermoanaerobaculia_bacterium]	ptg005110l	31.148	2.19E-20	93.6	96
KAB2960109.1_MAG:_tRNA-guanine_transglycosylase,_partial_[Thermoanaerobaculia_bacterium]	ptg002867l	53.642	9.9E-47	166	86
KAB2959995.1_MAG:_hypothetical_protein_F9K16_12010_[Thermoanaerobaculia_bacterium]	ptg003077l	51.656	1.72E-100	328	87
KAB2959990.1_MAG:_ATP-grasp_domain-containing_protein_[Thermoanaerobaculia_bacterium]	ptg004466l	51.692	2.18E-93	308	95
KAB2959985.1_MAG:_acyl-CoA_thioesterase_[Thermoanaerobaculia_bacterium]	ptg003536l	41.818	9.43E-19	84.7	76
KAB2959984.1_MAG:_gamma_carbonic_anhydrase_family_protein_[Thermoanaerobaculia_bacterium]	ptg004532l	44.509	4.85E-42	152	95
KAB2959983.1_MAG:_histidine--tRNA_ligase_[Thermoanaerobaculia_bacterium]	ptg003703l	48.101	7.11E-84	284	73
KAB2959982.1_MAG:_aspartate--tRNA_ligase_[Thermoanaerobaculia_bacterium]	ptg005341l	49.495	5.58E-141	455	100
KAB2959981.1_MAG:_3-dehydroquinate_synthase,_partial_[Thermoanaerobaculia_bacterium]	ptg004457l	44.586	8.76E-35	140	40
KAB2959980.1_MAG:_1-deoxy-D-xylulose-5-phosphate_reductoisomerase,_partial_[Thermoanaerobaculia_bacterium]	ptg003090l	51.673	9.25E-82	271	98
KAB2959979.1_MAG:_RIP_metalloprotease_RseP_[Thermoanaerobaculia_bacterium]	ptg002436l	31.628	1.05E-41	162	92
KAB2959977.1_MAG:_response_regulator_transcription_factor_[Thermoanaerobaculia_bacterium]	ptg001814l	44.872	8.79E-54	189	96
KAB2959976.1_MAG:_hydroxymethylbilane_synthase,_partial_[Thermoanaerobaculia_bacterium]	ptg001995l	44.398	3.07E-55	196	85
KAB2959975.1_MAG:_HAMP_domain-containing_histidine_kinase_[Thermoanaerobaculia_bacterium]	ptg005010l	33.778	1.06E-18	91.7	62
KAB2959974.1_MAG:_glutamate-1-semialdehyde_2,1-aminomutase_[Thermoanaerobaculia_bacterium]	ptg002871l	49.261	4.63E-87	293	93
KAB2959973.1_MAG:_hypothetical_protein_F9K16_12070_[Thermoanaerobaculia_bacterium]	ptg001290l	27.854	2.04E-13	76.6	49
KAB2959971.1_MAG:_cytochrome_c_[Thermoanaerobaculia_bacterium]	ptg001690l	39.216	8.87E-17	80.9	50
KAB2959970.1_MAG:_4Fe-4S_dicluster_domain-containing_protein,_partial_[Thermoanaerobaculia_bacterium]	ptg001690l	57.422	4.88E-92	308	97
KAB2959969.1_MAG:_hydrogenase_[Thermoanaerobaculia_bacterium]	ptg002453l	55.949	1.21E-135	435	85
KAB2959968.1_MAG:_DUF3341_domain-containing_protein_[Thermoanaerobaculia_bacterium]	ptg001690l	36.634	9.67E-21	92.4	99

KAB2959967.1_MAG:_hypothetical_protein_F9K16_12100_[Thermoanaerobaculia_bacterium]	ptg002453l	30.376	2E-30	127	87
KAB2959847.1_MAG:_iron-sulfur_cluster_assembly_accessory_protein_[Thermoanaerobaculia_bacterium]	ptg000819l	44.34	2E-20	88.2	98
KAB2959844.1_MAG:_3-oxoacyl-ACP_synthase_[Thermoanaerobaculia_bacterium]	ptg003871l	36.286	1.19E-51	188	97
KAB2959842.1_MAG:_GTP_cyclohydrolase_I_FolE_[Thermoanaerobaculia_bacterium]	ptg002479l	47.802	1.07E-46	167	89
KAB2959839.1_MAG:_transcription_termination_factor_NusA_[Thermoanaerobaculia_bacterium]	ptg003903l	38.228	3.12E-60	219	83
KAB2959837.1_MAG:_peptidase_S41_[Thermoanaerobaculia_bacterium]	ptg003980l	25.719	1.15E-65	248	98
KAB2959833.1_MAG:_M28_family_peptidase_[Thermoanaerobaculia_bacterium]	ptg005732l	34.855	1.29E-27	122	42
KAB2959830.1_MAG:_sigma-54-dependent_Fis_family_transcriptional_regulator_[Thermoanaerobaculia_bacterium]	ptg001025l	52.941	9.63E-59	212	53
KAB2959828.1_MAG:_methylmalonyl-CoA_mutase_[Thermoanaerobaculia_bacterium]	LG30	54.93	2.58E-13	77	39
KAB2959822.1_MAG:_IMP_dehydrogenase_[Thermoanaerobaculia_bacterium]	ptg002610l	55.925	3.12E-160	506	98
KAB2959818.1_MAG:_acetyl-CoA_carboxylase_biotin_carboxylase_subunit,_partial_[Thermoanaerobaculia_bacterium]	ptg003168l	47.436	1.09E-106	354	87
KAB2959723.1_MAG:_EAL_domain-containing_protein,_partial_[Thermoanaerobaculia_bacterium]	ptg005865l	41.176	2.22E-45	167	78
KAB2959722.1_MAG:_zinc_metalloprotease_[Thermoanaerobaculia_bacterium]	ptg001995l	38.403	2.91E-46	171	84
KAB2959721.1_MAG:_prephenate_dehydrogenase/arogenate_dehydrogenase_family_protein_[Thermoanaerobaculia_bacterium]	ptg005341l	27.353	1.39E-27	118	90
KAB2959720.1_MAG:_M3_family_metallopeptidase_[Thermoanaerobaculia_bacterium]	ptg001175l	42.5	9.36E-143	466	94
KAB2959719.1_MAG:_lytic_transglycosylase_domain-containing_protein_[Thermoanaerobaculia_bacterium]	ptg004256l	37.795	1.28E-12	69.7	50
KAB2959718.1_MAG:_CDP-diacylglycerol--glycerol-3-phosphate_3-phosphatidyltransferase_[Thermoanaerobaculia_bacterium]	ptg004000l	30.457	1.15E-13	72	89
KAB2959716.1_MAG:_phosphate_ABC_transporter_ATP-binding_protein_[Thermoanaerobaculia_bacterium]	ptg002567l	66.537	9.64E-115	366	90
KAB2959715.1_MAG:_phosphate_ABC_transporter_permease_PstA_[Thermoanaerobaculia_bacterium]	ptg002567l	71.269	1.46E-97	328	86
KAB2959714.1_MAG:_ABC_transporter_permease_subunit,_partial_[Thermoanaerobaculia_bacterium]	ptg002567l	55.051	3.91E-60	201	81
KAB2959703.1_MAG:_DUF1732_domain-containing_protein,_partial_[Thermoanaerobaculia_bacterium]	ptg005107l	44.706	2.32E-17	80.1	66
KAB2959702.1_MAG:_guanylate_kinase_[Thermoanaerobaculia_bacterium]	ptg005533l	38.095	1.68E-34	133	84
KAB2959700.1_MAG:_bifunctional_phosphopantothenoylcysteine_decarboxylase/phosphopantothenate--cysteine_ligase_CoaBC_[Thermoanaerobaculia_bacterium]	ptg005430l	42.222	2.94E-73	253	86
KAB2959697.1_MAG:_ABC_transporter_ATP-binding_protein_[Thermoanaerobaculia_bacterium]	ptg004986l	38.009	1.12E-37	145	73
KAB2959621.1_MAG:_EAL_domain-containing_protein,_partial_[Thermoanaerobaculia_bacterium]	ptg005865l	41.233	1.99E-120	401	76
KAB2959616.1_MAG:_hypothetical_protein_F9K18_11520,_partial_[Thermoanaerobaculia_bacterium]	ptg005431l	33.333	0.000000126	50.1	91
KAB2959614.1_MAG:_slipin_family_protein_[Thermoanaerobaculia_bacterium]	ptg002123l	34.454	8.46E-14	74.3	46
KAB2959611.1_MAG:_hypothetical_protein_F9K16_12360,_partial_[Thermoanaerobaculia_bacterium]	ptg003708l	35.714	3.08E-08	48.1	35
KAB2959609.1_MAG:_ribonuclease_Y_[Thermoanaerobaculia_bacterium]	ptg005484l	50.571	5.26E-96	323	67
KAB2959608.1_MAG:_TIGR00282_family_metallophosphoesterase_[Thermoanaerobaculia_bacterium]	ptg005484l	44.444	8.49E-68	231	99
KAB2959607.1_MAG:_glycosyltransferase_family_2_protein_[Thermoanaerobaculia_bacterium]	ptg003625l	40.081	3.48E-47	172	84
KAB2959600.1_MAG:_tetratricopeptide_repeat_protein,_partial_[Thermoanaerobaculia_bacterium]	LG25	29.839	1.03E-08	62	50

KAB2959513.1_MAG:_polynucleotide_adenylyltransferase_PcnB,_partial_[Thermoanaerobaculia_bacterium]	ptg003220l	37.019	1.62E-29	125	50
KAB2959512.1_MAG:_beta-lactamase_family_protein_[Thermoanaerobaculia_bacterium]	ptg002123l	28.485	4.76E-10	66.2	32
KAB2959511.1_MAG:_transcription-repair_coupling_factor,_partial_[Thermoanaerobaculia_bacterium]	ptg004785l	39.036	0	628	90
KAB2959507.1_MAG:_serine/threonine_protein_kinase,_partial_[Thermoanaerobaculia_bacterium]	ptg003082l	36.678	1.38E-42	159	94
KAB2959505.1_MAG:_TonB-dependent_receptor_[Thermoanaerobaculia_bacterium]	ptg001925l	30.816	3.98E-98	346	89
KAB2959503.1_MAG:_sulfatase-like_hydrolase/transferase_[Thermoanaerobaculia_bacterium]	ptg005545l	24.062	1.45E-15	83.6	87
KAB2959501.1_MAG:_efflux_RND_transporter_permease_subunit_[Thermoanaerobaculia_bacterium]	ptg004798l	33.957	5E-149	496	97
KAB2959500.1_MAG:_efflux_RND_transporter_periplasmic_adaptor_subunit_[Thermoanaerobaculia_bacterium]	ptg000732l	26.531	0.00000101	54.7	50
KAB2959497.1_MAG:_hypothetical_protein_F9K18_11575,_partial_[Thermoanaerobaculia_bacterium]	ptg000619l	37.681	0.000000581	57	11
KAB2959406.1_MAG:_DUF1573_domain-containing_protein_[Thermoanaerobaculia_bacterium]	ptg003830l	23.913	1.27E-08	60.8	60
KAB2959405.1_MAG:_hypothetical_protein_F9K16_12425_[Thermoanaerobaculia_bacterium]	ptg004510l	30.568	2.48E-29	76.6	82
KAB2959403.1_MAG:_prepilin_peptidase_[Thermoanaerobaculia_bacterium]	ptg002916l	35.135	8.89E-14	74.7	33
KAB2959402.1_MAG:_DNA_mismatch_repair_endonuclease_MutL,_partial_[Thermoanaerobaculia_bacterium]	ptg003361l	41.27	3.04E-50	182	99
KAB2959401.1_MAG:_pyruvate,_phosphate_dikinase_[Thermoanaerobaculia_bacterium]	ptg005844l	46.934	0	733	95
KAB2959398.1_MAG:_MATE_family_efflux_transporter,_partial_[Thermoanaerobaculia_bacterium]	ptg004856l	33.443	1.48E-25	111	94
KAB2959396.1_MAG:_type_I_DNA_topoisomerase_[Thermoanaerobaculia_bacterium]	LG04	40.466	3.18E-127	423	86
KAB2959392.1_MAG:_argininosuccinate_lyase_[Thermoanaerobaculia_bacterium]	ptg004584l	41.114	6.57E-49	181	94
KAB2959387.1_MAG:_ATP-binding_cassette_domain-containing_protein_[Thermoanaerobaculia_bacterium]	ptg003511l	48.879	3.58E-64	223	70
KAB2959386.1_MAG:_ABC_transporter_permease_subunit_[Thermoanaerobaculia_bacterium]	ptg005110l	28.75	7.19E-16	80.5	96
KAB2959382.1_MAG:_tRNA_lysidine(34)_synthetase_TilS,_partial_[Thermoanaerobaculia_bacterium]	ptg001005l	32.836	1.01E-22	102	63
KAB2959302.1_MAG:_c-type_cytochrome_[Thermoanaerobaculia_bacterium]	ptg006037l	44.186	4.26E-14	74.7	37
KAB2959300.1_MAG:_acetyl-CoA_hydrolase/transferase_family_protein_[Thermoanaerobaculia_bacterium]	LG22	65.306	5.13E-13	75.1	39
KAB2959296.1_MAG:_fumarate_reductase/succinate_dehydrogenase_flavoprotein_subunit_[Thermoanaerobaculia_bacterium]	ptg002316l	64.207	0	746	100
KAB2959295.1_MAG:_aminoacyl-tRNA_hydrolase_[Thermoanaerobaculia_bacterium]	ptg005110l	46.667	8.17E-12	64.7	66
KAB2959292.1_MAG:_succinate_dehydrogenase/fumarate_reductase_iron-sulfur_subunit_[Thermoanaerobaculia_bacterium]	ptg003304l	59.917	1.26E-99	322	94
KAB2959291.1_MAG:_succinate_dehydrogenase_cytochrome_b_subunit_[Thermoanaerobaculia_bacterium]	ptg002316l	40	2.63E-42	156	99
KAB2959289.1_MAG:_AAA_domain-containing_protein_[Thermoanaerobaculia_bacterium]	ptg004266l	47.826	3.91E-77	260	97
KAB2959288.1_MAG:_DUF58_domain-containing_protein_[Thermoanaerobaculia_bacterium]	ptg003750l	31.222	1.46E-18	90.1	70
KAB2959285.1_MAG:_PAS_domain-containing_protein_[Thermoanaerobaculia_bacterium]	ptg005484l	29.389	1.28E-20	100	50
KAB2959284.1_MAG:_ATP-dependent_protease_subunit_HslV_[Thermoanaerobaculia_bacterium]	ptg004959l	51.429	4.33E-41	150	98
KAB2959283.1_MAG:_ATP-dependent_protease_ATPase_subunit_HslU_[Thermoanaerobaculia_bacterium]	ptg004476l	54.81	1.94E-118	385	97
KAB2959205.1_MAG:_cell_division_ATP-binding_protein_FtsE_[Thermoanaerobaculia_bacterium]	ptg004158l	40.991	8.16E-45	163	92
KAB2959203.1_MAG:_OmpA_family_protein,_partial_[Thermoanaerobaculia_bacterium]	ptg002407l	42.045	3.97E-09	56.6	73

KAB2959193.1_MAG:_sigma-54-dependent_Fis_family_transcriptional_regulator,_partial_[Thermoanaerobaculia_bacterium]	ptg001025l	46	4.87E-29	117	65
KAB2959191.1_MAG:_membrane_protein_insertion_efficiency_factor_YidD_[Thermoanaerobaculia_bacterium]	LG10	39.062	0.00000639	45.1	94
KAB2959190.1_MAG:_nucleoside_deaminase_[Thermoanaerobaculia_bacterium]	ptg001690l	57.609	2.75E-18	84	61
KAB2959188.1_MAG:_membrane_protein_insertase_YidC_[Thermoanaerobaculia_bacterium]	ptg000372l	44.177	9.11E-50	190	42
KAB2959187.1_MAG:_ribonuclease_P_protein_component_[Thermoanaerobaculia_bacterium]	ptg005113l	36.283	3.04E-08	53.9	91
KAB2959185.1_MAG:_DNA_polymerase_III_subunit_gamma/tau,_partial_[Thermoanaerobaculia_bacterium]	ptg003168l	41.622	6.79E-63	222	90
KAB2959183.1_MAG:_DNA_primase_[Thermoanaerobaculia_bacterium]	ptg004856l	30.973	1.43E-49	190	70
KAB2959182.1_MAG:_enoyl-CoA_hydratase/isomerase_family_protein_[Thermoanaerobaculia_bacterium]	ptg004214l	36.97	6.58E-15	77.8	62
KAB2959115.1_MAG:_magnesium_transporter_[Thermoanaerobaculia_bacterium]	ptg005611l	29.288	6.46E-22	102	90
KAB2959114.1_MAG:_TIGR00730_family_Rossman_fold_protein,_partial_[Thermoanaerobaculia_bacterium]	ptg002465l	38.286	2.11E-24	105	63
KAB2959112.1_MAG:_excinuclease_ABC_subunit_UvrA_[Thermoanaerobaculia_bacterium]	ptg004916l	55.662	0	899	99
KAB2959111.1_MAG:_SsrA-binding_protein_SmpB_[Thermoanaerobaculia_bacterium]	ptg004680l	42.748	1.05E-21	94	83
KAB2959108.1_MAG:_serine/threonine_protein_kinase,_partial_[Thermoanaerobaculia_bacterium]	ptg003082l	40.408	6.15E-44	164	81
KAB2959106.1_MAG:_superoxide_dismutase_[Thermoanaerobaculia_bacterium]	ptg001604l	59.799	1.12E-62	213	99
KAB2959103.1_MAG:_hypothetical_protein_F9K16_12730_[Thermoanaerobaculia_bacterium]	ptg004771l	35.61	1.18E-30	121	89
KAB2959101.1_MAG:_hypothetical_protein_F9K18_11740_[Thermoanaerobaculia_bacterium]	ptg005886l	31.1	1.4E-25	109	77
KAB2959100.1_MAG:_sigma-54-dependent_Fis_family_transcriptional_regulator_[Thermoanaerobaculia_bacterium]	ptg003057l	37.061	1.07E-72	253	97
KAB2959098.1_MAG:_MATE_family_efflux_transporter_[Thermoanaerobaculia_bacterium]	ptg003856l	32.753	6.85E-12	72	60
KAB2959096.1_MAG:_single-stranded_DNA-binding_protein_[Thermoanaerobaculia_bacterium]	LG01	71.739	3.23E-13	72.4	19
KAB2959094.1_MAG:_deoxyribose-phosphate_aldolase_[Thermoanaerobaculia_bacterium]	ptg004266l	62.376	6.14E-114	365	100
KAB2959035.1_MAG:_ATP-binding_cassette_domain-containing_protein,_partial_[Thermoanaerobaculia_bacterium]	ptg002760l	49.505	5.31E-48	108	84
KAB2959032.1_MAG:_signal_peptide_peptidase_SppA,_partial_[Thermoanaerobaculia_bacterium]	ptg004077l	33.803	2.13E-35	147	70
KAB2959031.1_MAG:_leucyl_aminopeptidase,_partial_[Thermoanaerobaculia_bacterium]	ptg001925l	47.977	8.28E-43	123	88
KAB2959030.1_MAG:_ABC_transporter_ATP-binding_protein_[Thermoanaerobaculia_bacterium]	ptg005227l	42.945	3.07E-67	232	99
KAB2959029.1_MAG:_ABC_transporter_ATP-binding_protein,_partial_[Thermoanaerobaculia_bacterium]	ptg005227l	50.192	1.05E-64	222	98
KAB2959028.1_MAG:_3,4-dehydroadipyl-CoA_semialdehyde_dehydrogenase,_partial_[Thermoanaerobaculia_bacterium]	ptg004321l	30.052	2.91E-09	62.8	54
KAB2959027.1_MAG:_FKBP-type_peptidyl-prolyl_cis-trans_isomerase_[Thermoanaerobaculia_bacterium]	ptg003237l	39.423	8.05E-34	132	77
KAB2959026.1_MAG:_PAS_domain_S-box_protein_[Thermoanaerobaculia_bacterium]	ptg005484l	34.501	3.53E-47	186	48
KAB2958972.1_MAG:_glycosyltransferase_family_4_protein_[Thermoanaerobaculia_bacterium]	ptg004384l	29.148	6.8E-16	84	54
KAB2958970.1_MAG:_aldehyde_dehydrogenase_family_protein_[Thermoanaerobaculia_bacterium]	ptg004321l	71.55	0	650	99
KAB2958969.1_MAG:_aldehyde_dehydrogenase_[Thermoanaerobaculia_bacterium]	ptg005596l	53.71	1.69E-74	251	99
KAB2958968.1_MAG:_MMPL_family_transporter_[Thermoanaerobaculia_bacterium]	ptg004403l	47.941	2.74E-133	295	94
KAB2958967.1_MAG:_ABC-F_family_ATP-binding_cassette_domain-containing_protein_[Thermoanaerobaculia_bacterium]	ptg005398l	40.797	9.3E-121	399	82

KAB2958963.1_MAG:_deoxyribose-phosphate_aldolase_[Thermoanaerobaculia_bacterium]	ptg004266l	62.414	3.01E-107	346	95
KAB2958961.1_MAG:_CBS_domain-containing_protein_[Thermoanaerobaculia_bacterium]	ptg005487l	33.758	1.02E-12	75.9	22
KAB2958965.1_MAG:_alanine--tRNA_ligase,_partial_[Thermoanaerobaculia_bacterium]	ptg002313l	38.228	3.93E-137	454	90
