# Supplementary material for: Vanadium-Dependent Haloperoxidase Gene Evolution in Brown Algae: Evidence for Horizontal Gene Transfer
Source: Int J Mol Sci. 2025 Jan 16;26(2):716. doi: 10.3390/ijms26020716 (PMC11765636; doi:10.3390/ijms26020716)
Supplement: Supplementary file 1 [file ijms-26-00716-s001.zip › Supplementary figures S1-S3.pdf]

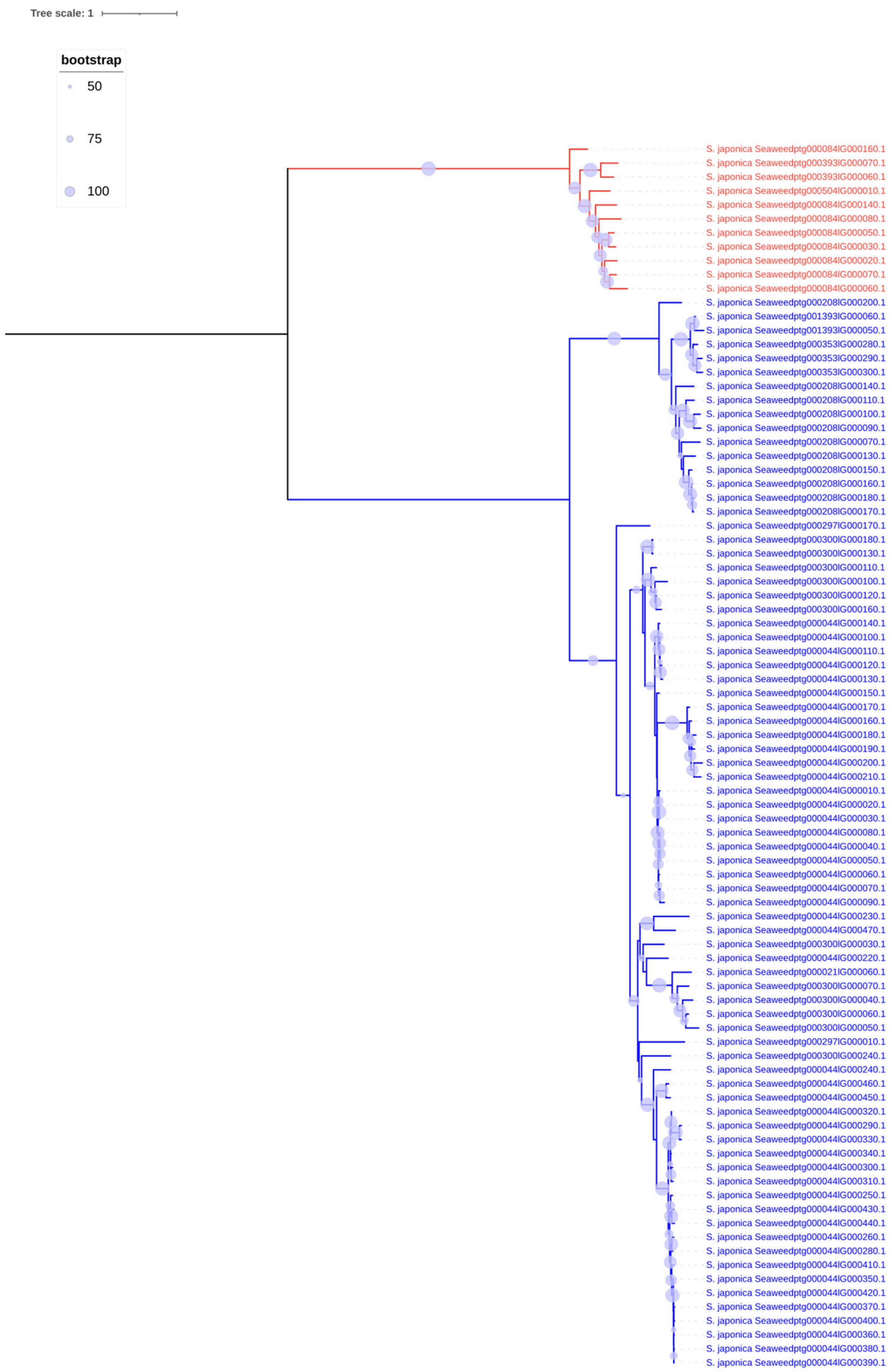

Figure S1. The phylogenetic of V-HPO in Saccharina japonica with bootstrap and branch length. The phylogenetic tree was constructed via maximum likelihood analysis with the WAG+F+G4 substitution model implemented in IQ-TREE 2. The tree was rooted at the midpoint, and the bootstraps are indicated.

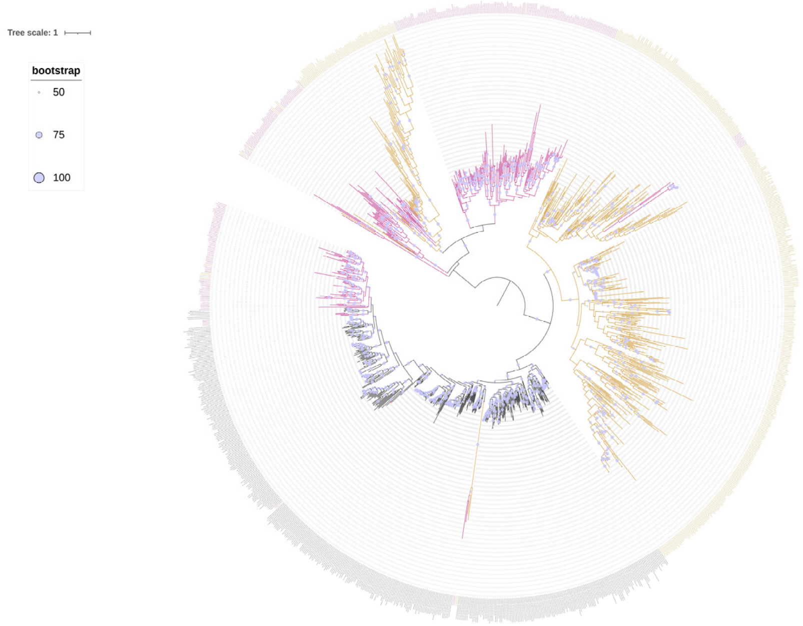

Figure S2. Phylogenetic clade analysis of V-HPO in algae and its relationship with bacteria with bootstrap and branch length. The phylogenetic tree was constructed via maximum likelihood analysis with the VT+R10 substitution model implemented in IQ-TREE 2. The tree was rooted at the midpoint, and the bootstraps are indicated.

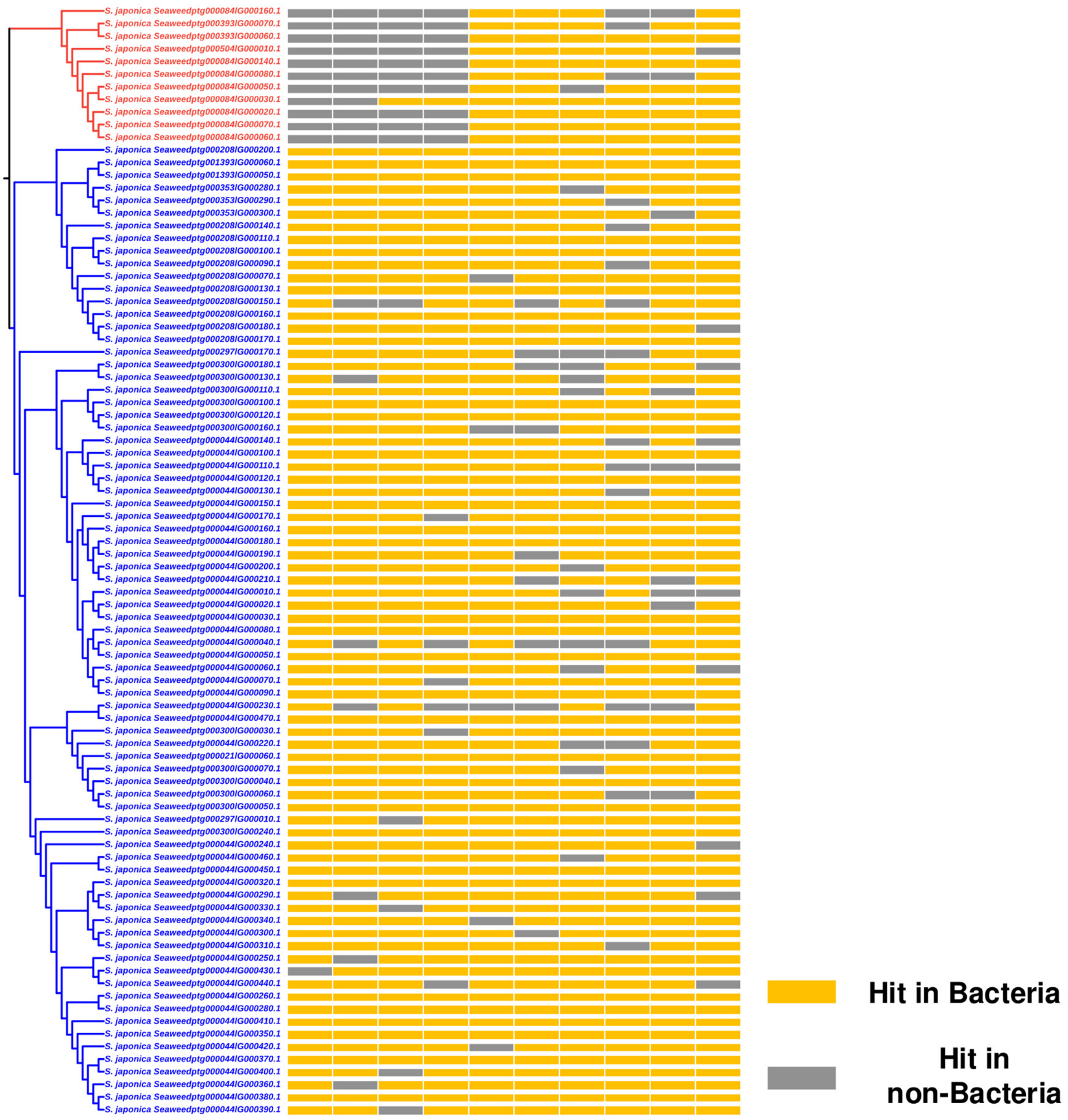

Figure S3. Taxonomic distribution of top 10 hits from V-HPO analysis against the non-redundant database, excluding species from brown and red algae. Bacterial hits are specifically highlighted in orange and the hit in non-bacteria are labeled in grey.
