# Supplementary figures and images for: Vanadium-Dependent Haloperoxidase Gene Evolution in Brown Algae: Evidence for Horizontal Gene Transfer
Source: Int J Mol Sci. 2025 Jan 16;26(2):716. doi: 10.3390/ijms26020716 (PMC11765636; doi:10.3390/ijms26020716)

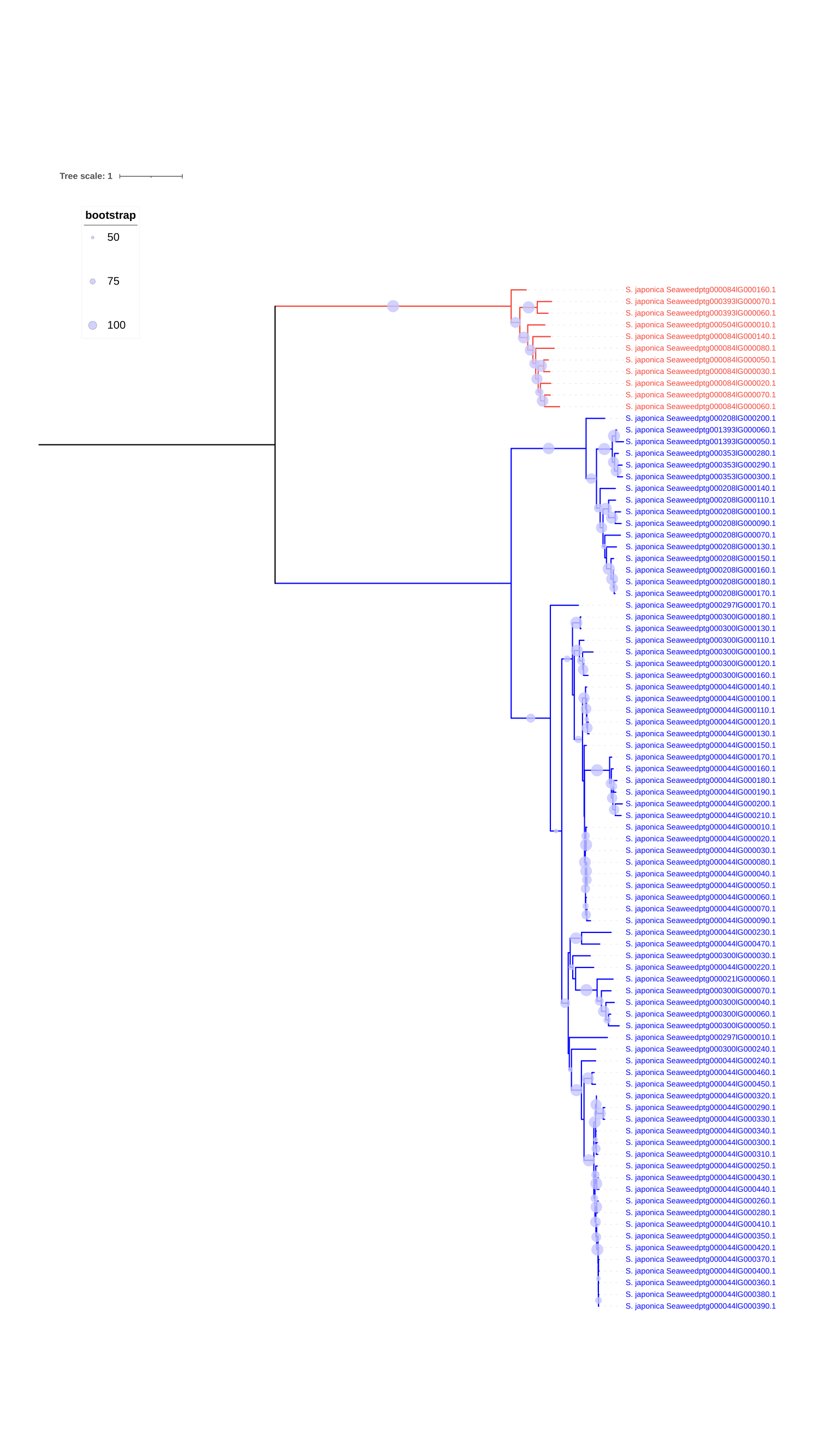

Supplement: Supplementary file 1 [file ijms-26-00716-s001.zip › Supplemental Figure S1. The phylogenetic of V-HPO in Saccharina japonica .tiff]

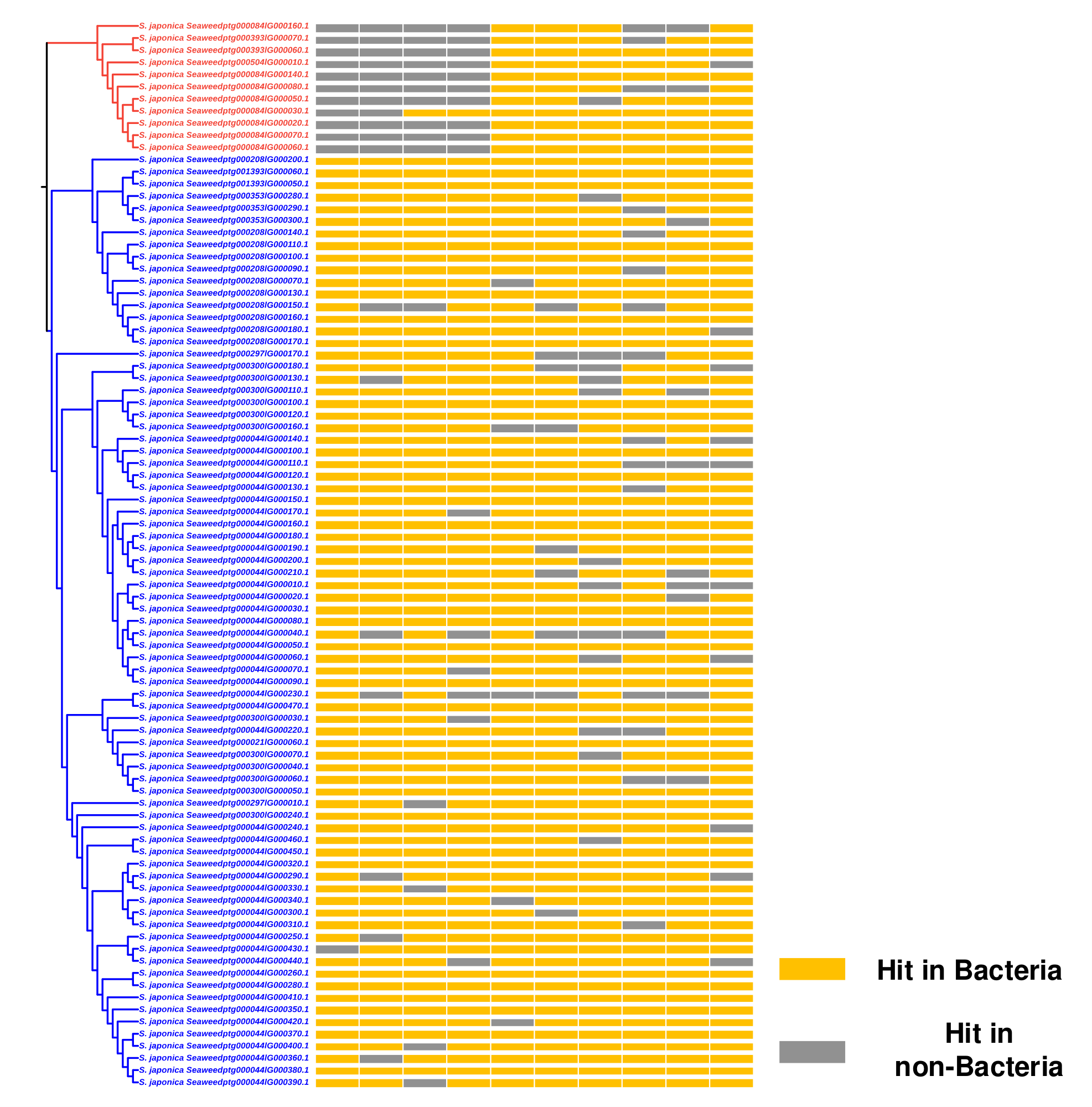

Supplement: Supplementary file 1 [file ijms-26-00716-s001.zip › Supplemental Figure S3. vHPO top hit taxonomy with non Ochrophyta and non-Rhodophyta.tiff]
